# Population genetic analysis of the *Plasmodium falciparum* erythrocyte binding antigen-175 (EBA-175) gene in Equatorial Guinea

**DOI:** 10.1186/s12936-021-03904-x

**Published:** 2021-09-19

**Authors:** Pei-Kui Yang, Xue-Yan Liang, Min Lin, Jiang-Tao Chen, Hui-Ying Huang, Li-Yun Lin, Carlos Salas Ehapo, Urbano Monsuy Eyi, Yu-Zhong Zheng, Dong-De Xie, Jin-Quan He, Huan-Tong Mo, Xin-Yao Chen, Xiang-Zhi Liu, Ying-E. Wu

**Affiliations:** 1 Department of Medical Laboratory, Chaozhou People’s Hospital Affiliated to Shantou University Medical College, Chaozhou, Guangdong Province People’s Republic of China; 2 School of Life Science and Food Engineering, Hanshan Normal University, Chaozhou, People’s Republic of China; 3 Department of Clinical Laboratory, Huizhou Municipal Central Hospital, Huizhou, People’s Republic of China; 4 The Chinese Medical Aid Team to the Republic of Equatorial Guinea, Guangzhou, Guangdong Province People’s Republic of China; 5 Department of Medical Laboratory, Malabo Regional Hospital, Malabo, Equatorial Guinea; 6 Department of Medical Laboratory, First Affiliated Hospital of Shantou University Medical College, Shantou, Guangdong Province People’s Republic of China

**Keywords:** Bioko Island, Bata district, *Plasmodium falciparum*, *Pf*EBA-175, Natural selection, Genetic diversity

## Abstract

**Background:**

*Plasmodium falciparum* erythrocyte binding antigen-175 (*Pf*EBA-175) is a candidate antigen for a blood-stage malaria vaccine, while various polymorphisms and dimorphism have prevented to development of effective vaccines based on this gene. This study aimed to investigate the dimorphism of *Pf*EBA-175 on both the Bioko Island and continent of Equatorial Guinea, as well as the genetic polymorphism and natural selection of global *Pf*EBA-175.

**Methods:**

The allelic dimorphism of *Pf*EBA-175 region II of 297 bloods samples from Equatorial Guinea in 2018 and 2019 were investigated by nested polymerase chain reaction and sequencing. Polymorphic characteristics and the effect of natural selection were analyzed using MEGA 7.0, DnaSP 6.0 and PopART programs. Protein function prediction of new amino acid mutation sites was performed using PolyPhen-2 and Foldx program.

**Results:**

Both Bioko Island and Bata district populations, the frequency of the F-fragment was higher than that of the C-fragment of *Pf*EBA-175 gene. The *Pf*EBA-175 of Bioko Island and Bata district isolates showed a high degree of genetic variability and heterogeneity, with π values of 0.00407 & 0.00411 and Hd values of 0.958 & 0.976 for nucleotide diversity, respectively. The values of Tajima's D of *Pf*EBA-175 on Bata district and Bioko Island were 0.56395 and − 0.27018, respectively. Globally, *Pf*EBA-175 isolates from Asia were more diverse than those from Africa and South America, and genetic differentiation quantified by the fixation index between Asian and South American countries populations was significant (FST > 0.15, *P* < 0.05). A total of 310 global isolates clustered in 92 haplotypes, and only one cluster contained isolates from three continents. The mutations A34T, K109E, D278Y, K301N, L305V and D329N were predicted as probably damaging.

**Conclusions:**

This study demonstrated that the dimorphism of F-fragment *Pf*EBA-175 was remarkably predominant in the study area. The distribution patterns and genetic diversity of *Pf*EBA-175 in Equatorial Guinea isolates were similar another region isolates. And the levels of recombination events suggested that natural selection and intragenic recombination might be the main drivers of genetic diversity in global *Pf*EBA-175. These results have important reference value for the development of blood-stage malaria vaccine based on this antigen.

**Supplementary Information:**

The online version contains supplementary material available at 10.1186/s12936-021-03904-x.

## Background

Malaria, a serious parasitic disease for public health that impacts the lives of millions of people worldwide and is responsible for half a million deaths annually [1]. Malaria is caused by protozoan parasites of the genus *Plasmodium*, which include five species that infect humans, of which *Plasmodium falciparum* has the highest mortality rate [2]. According to the World Malaria Report 2020, total of 229 million malaria cases were reported in 89 malaria endemic regions in 2019, and *P. falciparum* was responsible for approximately 99.7% of malaria cases in the Africa region [1]. Malaria is endemic in Equatorial Guinea, which is located in Central West Africa. Since 2004, the Bioko Island Malaria Control Project (BIMCP) has committed to reducing the burden of malaria on Bioko Island through methods, such as concerted vector control, improved case management, and various educational interventions for the past 15 years. As a result, the BIMCP has reduced malaria prevalence from 43.3% in 2004 to 10.5% in 2016 [3]. Similarly, some successes have been achieved in high-transmission areas in the world. However, malaria parasites with resistance to anti-malarial drugs and mosquito vectors with resistance to insecticides have highlighted the importance of developing a malaria vaccine [4]. Therefore, as a powerful tool for malaria control and prevention, an effective vaccine against malaria remains an important global health priority.

Vaccines are among the most successful and cost-effective interventions in the history of public health [5]. However, development an effective vaccine for *P. falciparum* malaria has been stymied by various issues, such as the extreme complexity of its biology, the diversity of genome organization, the capability of immune evasion and the intricate nature of infection cycle [6]. For instance, the invasion of red blood cells by malaria parasite involves many processes. Multiple interactions between host erythrocyte receptors and parasite ligands are displayed on the merozoite surface during the invasion process [7]. Hence, it is very important to develop a malaria vaccine against molecules which play critical roles in the invasion process into erythrocytes [4]. One of the major antigens of *P. falciparum* merozoite is *Pf*EBA-175, a sialic acid-binding protein ligand, which was the first described invasion ligand of the parasite [8]. *Pf*EBA-175 mainly interacts with glycophorin A located on the erythrocyte surface [9, 10]. The *Pf*EBA-175 gene includes seven regions, and region II contains two cystine-rich segments (F1 and F2) responsible for binding to GPA [11]. It has been shown that immunization with *Pf*EBA-region II could induce significant antiparasitic effects in vivo [12]. Studies have shown that *Pf*EBA-175 region III has a highly dimorphic segment. The dimorphism of region III is an insertion of a 423 base pair segment in the FCR3 strain (F-fragment) or insertion of a 342 base pair segment in the CAMP strain (C-fragment) [8, 13, 14]. However, the relationship between the dimorphism of *Pf*EBA-175 and host–parasite interactions is still unclear [15]. Several studies have indicated that the F or C segment binds to the GPA backbone after *Pf*EBA-175 region II binds to GPA sialic acid residues [16, 17].

As an important *P. falciparum* vaccine candidate gene, *Pf*EBA-175 has been found to show genetic polymorphisms between different isolates [18]. The aims of the present study were to investigate the genetic polymorphism of region II of *Pf*EBA-175 gene of *P. falciparum* on Bioko and Bata, two districts of Equatorial Guinea, as well as to elucidate differences among global *P. falciparum* populations.

## Methods

### Study area and population

**Fig. 1:**
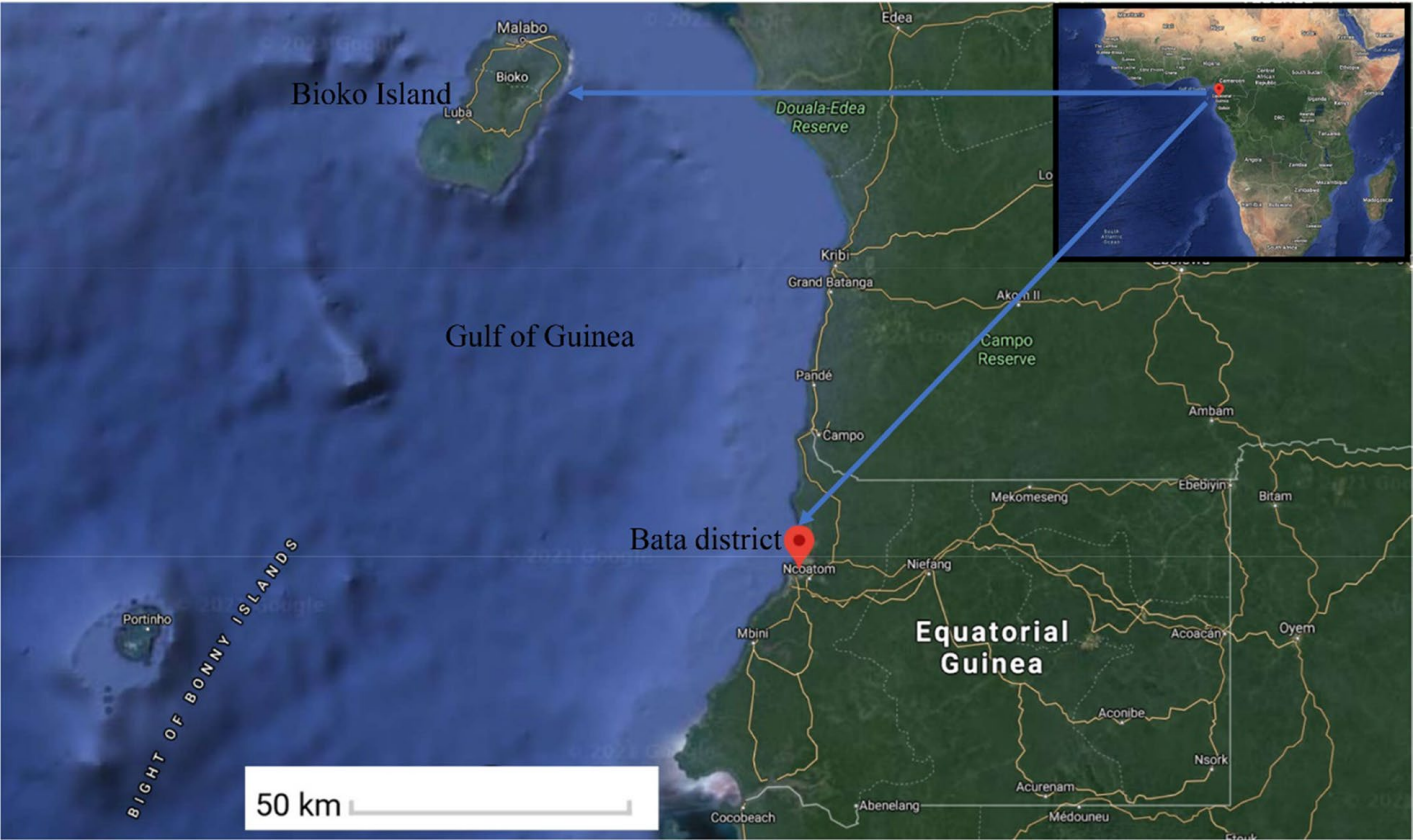
Map of Bioko Island and Bata district of Equatorial Guinea

Equatorial Guinea is located on the west coast of Central Africa at approximately 3° N latitude and 9° E longitude. It is bordered by Cameroon to the north and Gabon to the west and south. This country consists of two parts: a mainland and an insular region (Fig. 1). Bioko is the largest island with a population of 335,048 inhabitants, which is located in the gulf of Guinea, 160 km northwest of the mainland region. Bata is the largest district in the mainland region, with a population of 244,264 inhabitants [19]. Despite the efforts made by the Equatorial Guinea Malaria Control Initiative (EGMCI, 2006–2011), and the Bioko Island Malaria Control Project (BIMCP, 2004–2018), which have had a marked impact on malaria transmission [20–22], malaria is still the major public health problem in the country (www.mcdinternational.org).

### Ethical approval

Verbal informed consent was obtained from all participating subjects or their parents, and this study and the consent process were approved by the Ethics Committee of Malabo Regional Hospital. The Ethical approval letter had been shown as Additional files 1 and 2.

### Study population and blood sample collection

The study was carried out in Malabo Regional Hospital, private diagnostic clinics (PDCs) in different regions of Bioko Island and Bata district, and in a clinic of the Chinese medical aid team to the Republic of Equatorial Guinea. In 2018–2019, *P. falciparum* clinical samples (from individuals 4 months to 80 years of age) were collected from 297 *P. falciparum* malaria cases confirmed by microscopic examination and lateral flow test kit (ICT Diagnostics). Blood samples were collected on filter paper (Whatman 3 mm, GE Healthcare, Pittsburg, USA) for further molecular analysis, air-dried and stored in plastic sealing bags at ambient temperature. Thick blood smears were air dried and stained with 10% fresh Giemsa following standard procedures. After coding and recording the patient medical records, the dried blood filters were stored in plastic sealing bags at − 80 °C.

### DNA extraction

Genomic DNA (gDNA) was extracted from the dried blood spots using 0.5% saponin (Sigma-Aldrich, Taufkirchen, Germany) to free parasites from red blood cells followed by Chelex®100 (Bio-Rad Laboratories, CA, USA) method, as previously described [23], and stored at − 20 °C.

### Allelic genotyping of the *Pf*EBA-175

An improved nested PCR method for genotype determination of *Pf*EBA-175 as described by Touré et al*.* [24] was used. In the first-round PCR, 1 μL of gDNA was amplified with 10 μL 2 × Taq Plus master Mix II (GenStar, Beijing, China), 1 μL of 10 μmol/L forward primer (EBA1: 5′-CAAGAAGCAGTTCCTGAGGAA-3′) and 1 μL of 10 μmol/L reverse primer (EBA2: 5′-TCTCAACATTCATATTAACAATTC-3′), and sterile ultrapure water was added to a final volume of 20 μL. Thermal cycling parameters for PCR were as follows: initial denaturation at 96 °C for 3 min; 30 cycles of 96 °C for 10 s, 57 °C for 10 s, 72 °C for 50 s; and final extension of 72 °C for 10 min. For the second-round PCR, 1 μL of the primary PCR product was amplified with 10 μL 2 × Taq Plus master Mix II (GenStar, Beijing, China), 1 μL of 10 μmol/L forward primer (EBA3: 5′-GAGGAAAACACTGAAATAGCACAC-3′) and 1 μL of 10 μmol/L reverse primer (EBA4: 5′-CAATTCCTCC-AGACTGTTGAACAT-3′), and sterile ultrapure water was added to a final volume of 20 μL. The nested PCR cycling parameters used in the second round were the same as those used for the primary reaction. Nested PCRs were carried out using a LifeECO® PCR System 9700 (Bioer Technology, Binjiang District, China). An allele-specific positive control and DNA negative control were included in each set of reactions.

### Detection of alleles of *Pf*EBA-175

The PCR products were stained with StarGreen nucleic acid gel stain (GenStar, Beijing, China) and resolved by gel electrophoresis in 2% agarose gel. The fragment sizes were determined using a low molecular weight DNA ladder marker (250–5000 bp, Dongsheng Biotech, Guangzhou, China) and photographed with a Tanon 2500/2500R Gel Imaging System (Tanon Science & Technology Co., Ltd., Shanghai, China). Alleles of *Pf*EBA-175 were categorized according to their molecular weights.

### Amplification and sequencing of domains II and III of *Pf*EBA-175

For sequencing domains II and III of *Pf*EBA-175 gene, the samples were amplified by nested PCR. In the first-round PCR, 1 μL of gDNA was amplified with 10 μL PrimeSTAR Max Premix (Takara Bio, Dalian, China), 1 μL of 10 μmol/L forward primer (EBA175F1: 5′-ATTAACGCTGTACGTGTGTCTAG-3′) and 1 μL of 10 μmol/L reverse primer (EBA2: 5′-TCTCAACATTCATATTAACAATTC-3′), 1 μL of DMSO and sterile ultrapure water was added to a final volume of 20 μL. The thermal cycling parameters for PCR were as follows: initial denaturation at 96 °C for 3 min; 30 cycles of 96 °C for 15 s, 57.7 °C for 10 s, 72 °C for 2 min; a final extension of 72 °C for 10 min. For the second-round PCR, 1 μL of the primary PCR product was amplified with 10 μL PrimeSTAR Max Premix (Takara Bioko, Beijing, China), 1 μL of 10 μmol/L forward primer (EBA175F2: 5′-AAGAAATACTTCATCTAATAACG-3′) and 1 μL of 10 μmol/L reverse primer (EBA4: 5′-CAATTCCTCCAGACTGTTGAACAT-3′), and sterile ultrapure water was added to a final volume of 20 μL. The nested PCR cycling parameters of the second round were the same as those of the primary reaction. Nested PCRs were carried out using a LifeECO® PCR System 9700 (Bioer Technology, Binjiang District, China). All PCR products were analysed using 1% agarose gel electrophoresis. Then, a random selection of 70 samples presented only one amplified fragment (F or C) were purified and then sequenced by using an ABI 3730 × L automated sequencer (BGI, Shenzhen, China). The sequencing primers were the same as the primers for the second-round PCR. All sequences were analysed and integrated by BioEdit Sequence Alignment Editor Software version 7.2.5 (BioEdit, California, USA) and MEGA 7.0 [25] software.

### Sequence analysis of domain II of the *Pf*EBA-175

The MEGA 7.0 was used for the analysis of the nucleotide sequence polymorphism, and the amino acid sequences of domain II of *Pf*EBA-175 from Equatorial Guinea were also analysed by this program. The numbers of segregating sites (S), haplotypes (H), haplotype diversity (Hd), nucleotide diversity (π), and average number of pairwise nucleotide differences within a population (K) were estimated by using DnaSP 6.0 [26]. The value of π was calculated to estimate stepwise the diversity of domain II of *Pf*EBA-175 based on a sliding window of 100 bp with a step size of 5 bp. Values of nonsynonymous (dN) and synonymous (dS) substitutions were estimated and compared using the Z test (*P* < 0.05 was considered significant) in the MEGA 7.0 program based on the method of Nei and Gojobori [27] with Jukes and Cantor correction. Tajima’s D value [28] and Fu and Li’s D and F values [29] were analysed using DnaSP 6.0 to evaluate the neutral theory of evolution [26]. Recombination parameters (R), which included the effective population size and probability of recombination between adjacent nucleotides per generation, and the minimum number of recombination events (Rm) were analyzed by DnaSP 6.0. Linkage disequilibrium (LD) between different polymorphic sites was computed based on the R^2^ index using DnaSP 6.0. The nucleotide sequences obtained were aligned and translated into putative amino acid sequences using MEGA 7.0. To examine the phylogenetic relations among 310 *Pf*EBA-175 region II of global *P. falciparum* isolates a neighbor-joining (NJ) tree was constructed. To obtain the neighbor-joining (NJ) tree, the neighbor-joining (NJ) method, applying the nucleotide substitution type and Poisson model and bootstrapping procedure with a minimum of 1000 bootstraps by using MEGA 7.0. The phylogenetic tree was further edited by iTol (https://itol.embl.de/).

### Global sequences acquisition and global diversity analysis

The genetic diversity of *Pf*EBA-175 domain II in other global *P. falciparum* isolates was analysed. Parasite populations from seven countries, including Kenya (*n* = 39, DQ092087–DQ092125), Thailand (Year = 2006, *n* = 48, DQ092039–DQ092086), Thailand (Year = 2015, *n* = 32, LC008232–LC008263), Benin (*n* = 8, KJ419497–KJ419504), Colombia (*n* = 20, KJ419512–KJ419531), Madagascar (*n* = 7, KJ419505–KJ419511), Peru (*n* = 30, KJ419547–KJ419576), Venezuela (*n* = 9, KJ419577–KJ419585), Nigeria (*n* = 30, AJ438799–AJ438828) and two regions of French Guiana, namely French Guiana (Camopi) (*n* = 23, KJ419586–KJ419608) and French Guiana (Maripasoula) (*n* = 15, KJ419532–KJ419546) were included in this analysis. All publicly available sequences covered the domain II of *Pf*EBA-175 (The alignment of all genetic sequences used in the study with FASTA format as Additional file 3 showed). Nucleotide sequence polymorphism analysis and neutrality test were performed for each population using programs DnaSP 6.0 and MEGA 7.0 as described above. Genetic differentiation among parasite populations was calculated based on the fixation index (FST) to estimate pairwise DNA sequence diversity between and within populations using Arlequin 3.5 [30]. To investigate relationships among *Pf*EBA-175 haplotypes, the haplotype network for a total of 310 *Pf*EBA-175 sequences, including 49 Bioko Island and Bata district sequences and the 261 publicly available sequences from Kenya, Thailand, Benin, Colombia, Madagascar, French Guiana, Peru, Venezuela, and Nigeria, was constructed using the Median Joining algorithm of the PopART program [31]. Nucleotide diversity and natural selection of each region were analysed using DnasP 6.0 as described above.

### Prediction of the impact of amino acid change upon protein structure

The potential impact of amino acid substitutions on the structure or function was predicted by the PolyPhen-2 [32] online server and PROVEAN [33]. The FOLDX plugin [34] in YASARA [35] was used to predict the changes in free energy before and after the mutations: ΔΔG(change) = ΔG(mutation)–ΔG(wild-type). Generally, the ΔΔG (change) > 0 to indicate a destabilizing mutation and ΔΔG (change) < 0 to indicate a stabilizing mutation.

### Statistical analysis

All statistical analyses were performed using the software Statistical Package for Social Sciences version 17.0 (SPSS, Inc., Chicago, IL, USA). The chi-square test was used in the univariate analysis to compare proportions. Statistical significance was set at alpha = 0.05 for all tests.

## Results

### Dimorphism of the *Pf*EBA-175 in Equatorial Guinea

**Fig. 2:**
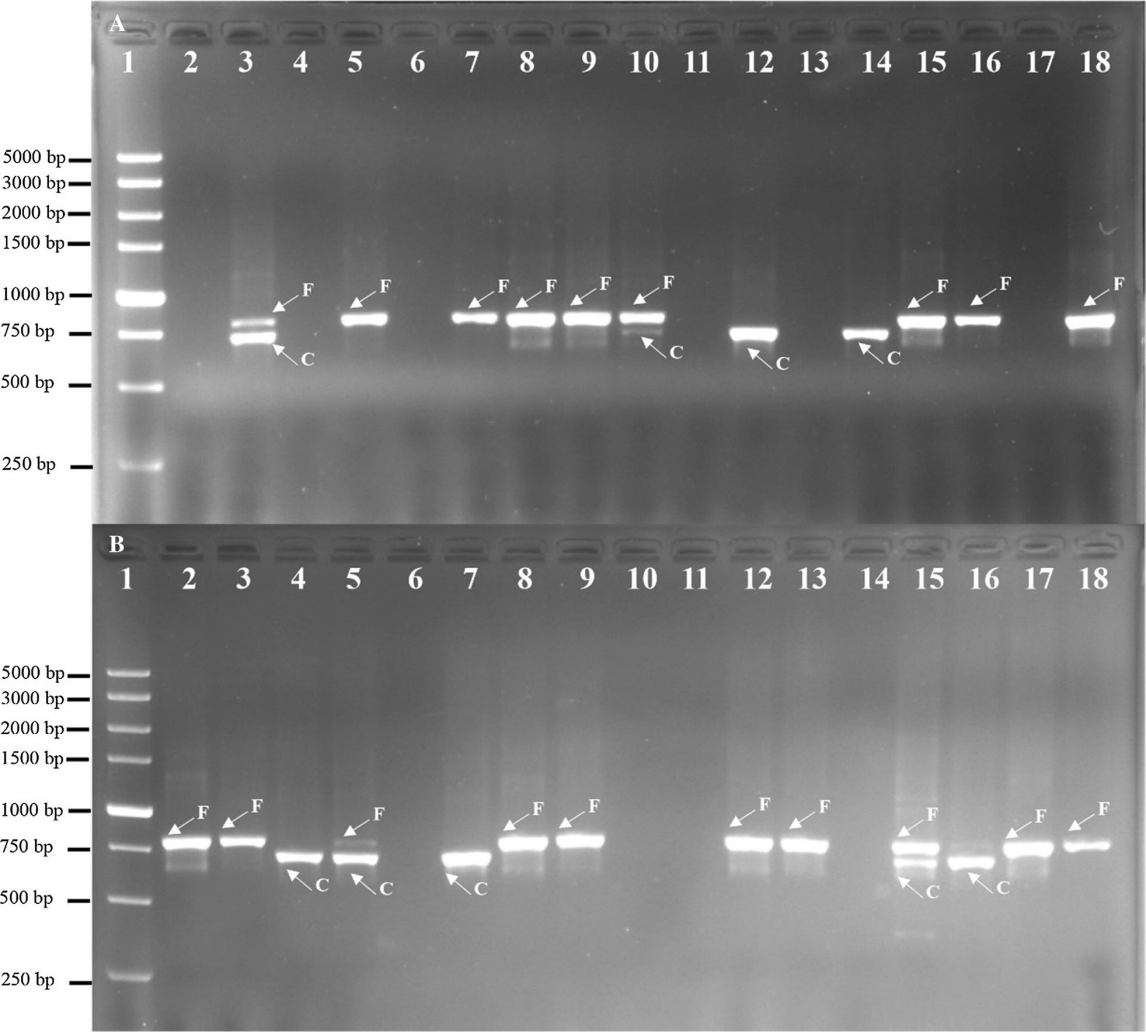
Nested polymerase chain reaction of *Pf*EBA-175. Lane 1 in A, B: 5000 bp molecular ladder; lanes 2–18 in A and B are samples from Bata district and Bioko Island of Equatorial Guinea, respectively. C-fragment of 714 bp; F-fragment of 795 bp; mixed infection (F- and C-fragments)

Of the 297 blood samples extracted from the collections in Bioko Island (*n* = 225) and Bata district (*n* = 72), 254 yielded suitable *Pf*EBA-175 amplicons for allelic genotyping. As shown in Fig. 2, two types of fragments were identified in Bioko and Bata by nested PCR; one was 795 bp, corresponding to the F-fragment, and the other was 714 bp. The vast majority of the *P. falciparum* isolates presented only one amplified fragment with 87.8%, while both fragments were observed in approximately 12.2% of the isolates representing mixed infections (Table 1). The frequency of F-fragment *Pf*EBA-175 in Bioko Island and Bata *P. falciparum* isolates were 71.92% and 56.86%, respectively (Table 1). It is worth noting that the F-fragment was more frequently detected in Bioko Island than on the continent (*P* = 0.024, χ^2^ test).

**Table 1:** Dimorphism of *P. falciparum Pf*EBA-175 in Equatorial Guinea

	Bioko Island *n* = 203	Bata district *n* = 51	Total *n* = 254
Single infection^a^	88.18% (179/203)	86.27% (44/51)	87.80% (223/254)
*F-fragment*	71.92% (146/203)*	56.86% (29/51)*	68.89% (175/254)
*C-fragment*	16.25% (33/203)	29.41% (15/51)	18.90% (48/254)
Mixed infection^b^	11.82% (24/203)	13.73% (7/51)	12.20% (31/254)

^a^Isolates with one fragment

^b^Isolates with two fragments

*P < 0.05 F-fragment *versus* C-fragment

### Sequencing for the region II in Equatorial Guinea *Pf*EBA-175

Of the 69 blood samples from the collections in Bioko (31 samples including 20 F-type and 11 C-type) and Bata (38 samples including 26 F-type and 12 C-type), with suitable F-type or C-type *Pf*EBA-175 amplicons for sequencing, finally, 49 (28 samples from Bioko with 17 F-type and 11 C-type; 21 samples from Bata with 14 F-type and 11 C-type) full-length monoclonal *Pf*EBA-175 region II sequences (532–1932) were analysed in the study. These nucleotide sequences have been deposited at GenBank under Accession Numbers (MW691428–MW691476).

### Genetic polymorphisms and natural selection of the region II in Equatorial Guinea *Pf*EBA-175

The parameters associated with nucleotide diversity and natural selection were also evaluated on the region II of Equatorial Guinea *Pf*EBA-175 (Table 2). The average number of pairwise nucleotide differences (K) values of whole region II (532–1932), F1 domain (532–1275) and F2 domain (1456–1932) were 5.700, 3.677 and 1.891 in Equatorial Guinea *Pf*EBA-175, respectively. The haplotype diversity (Hd) for *Pf*EBA-175 region II was 0.97 ± 0.013. This value of F1 domain (0.959 ± 0.012) was higher than for the F2 domain (0.700 ± 0.067). The π value of *Pf*EBA-175 region II was 0.00409 ± 0.00023. The π values analysis of the F1 and F2 domains revealed that more nucleotide diversity was concentrated in the F1 domain. In order to examine whether natural selection has contributed to the generation of region II diversity in Equatorial Guinea *P. falciparum* populations, the value of dn/ds was estimated using the Nei and Gojobori method. The value of dn/ds for region II was 0.005, suggesting that balancing natural selection might have occurred in region II of the Equatorial Guinea *P. falciparum* populations. Considering high positive dn/ds values for the F1 domain (0.006), these regions might experience more pressure from balancing natural selection forces. The estimated Tajima’s D value of region II was 0.07177 (*P* > 0.10). When Tajima’s D value was analysed for each domain, the F1 domain (0.52076, *P* > 0.10) showed higher positive Tajima’s D values compared to the F2 domain (0.14802, *P* > 0.10).

**Table 2:** DNA sequence polymorphism and tests of neutrality at *Pf*EBA-175 region II of *P. falciparum* isolates in Equatorial Guinea

Fragment	Nt/bp	S	Total no. of mutations	K	H	Hd ± SD	π ± SD	dN-dS	Tajima's D
Region II	532–1932	24	25	5.7	34	0.97 ± 0.013	0.00409 ± 0.00023	0.005	0.07177 (P > 0.10)
F1 domain	532–1275	14	14	3.677	26	0.959 ± 0.012	0.00494 ± 0.00026	0.006	0.52076 (P > 0.10)
F2 domain	1456–1932	7	8	1.891	12	0.700 ± 0.067	0.00348 ± 0.00039	0.004	0.14802 (P > 0.10)

S: segregating sites, K: average number of pairwise nucleotide differences, H: number of haplotypes, Hd: haplotype diversity, π: observed average pairwise nucleotide diversity, dN: rate of nonsynonymous mutations, dS: rate of synonymous mutations

^*^P < 0.05 and **P < 0.02

### Amino acid polymorphism in *Pf*EBA-175 region II from Equatorial Guinea and other global *P. falciparum* isolates

**Fig. 3:**
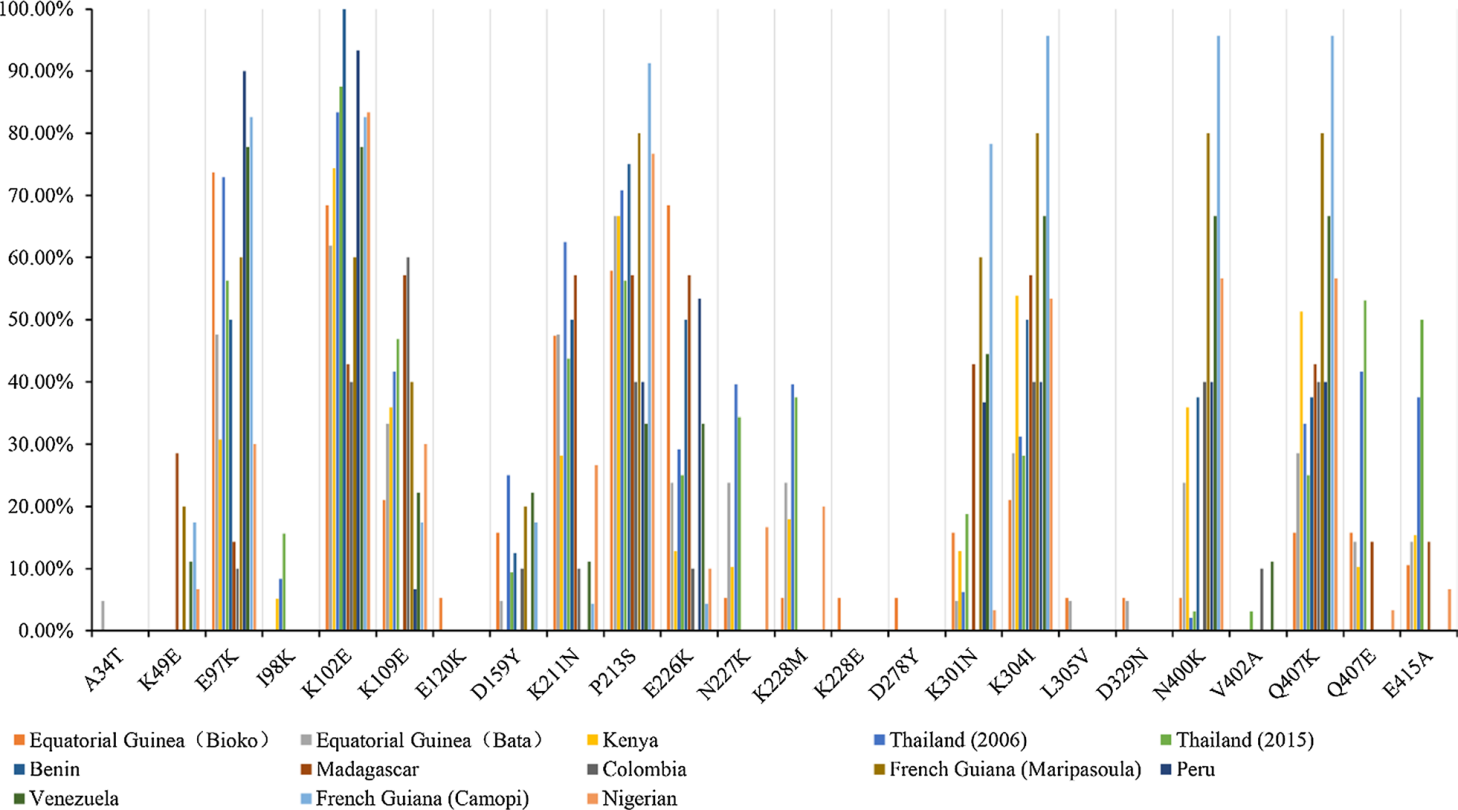
Amino acid polymorphisms of region II of global *Pf*EBA-175. Each region of *Pf*EBA-175 is marked by a different color

The amino acid polymorphisms of *Pf*EBA-175 region II Equatorial Guinea isolates were compared to those from other countries or regions. The results showed that 24 amino acid changes were identified in *Pf*EBA-175 region II from global isolates (Fig. 3, Table 3). There were 14 amino acid changes (A34T, K49E, E97K, I98K, K102E, K109E, E120K, D159Y, K211N, P213S, E226K, N227K, K228M, and N238S) that were found in the F1 domain in *Pf*EBA-175 region II (Fig. 3, Table 3). Of the 9 amino acid changes (K301N, K304I, L305V, D329N, N400K, V402A, Q407K, Q407E, and E415A) were found in the F2 domain in *Pf*EBA-175 region II (Fig. 3, Table 3), whereas D278Y exists in the linking region, which is between the F1 and F2 domain. The frequency of amino acid mutations of Equatorial Guinea *P. falciparum* isolates, whether from Bioko Island or Bata, is lower than that from other parts of the world. The most common amino acid changes is K102E, which frequency in the global isolates were as follows: Bioko (68.42%), Bata, (61.90%), Kenya (74.36%), Thailand (2006) (83.33%), Thailand (2015) (87.5%), Benin (100%), Madagascar (42.86%), Colombia (40%), French Guiana (Maripasoula) (60%), Peru (93.33%), Venezuela (77.78%), French Guiana (Camopi) (82.61%), and Nigeria (83.33%).

**Table 3:** Functional prediction of mutation sites

	Mutation sites	ΔΔG	Poly-Phen-2				PROVEAN	
			Score (HumDiv)^a^	Sensitivity	Specificity	Classification^b^	Score	Prediction^c^
F1 domain	A34T	4.725	0.999	0.14	0.99	Probably damaging	− 1.151	Neutral
	K49E	0.273	0.022	0.95	0.80	Benign	− 1.356	Neutral
	E97K	− 0.043	0.000	1.00	0.00	Benign	0.031	Neutral
	I98K	1.842	0.002	0.95	0.30	Benign	1.265	Neutral
	K102E	− 0.358	0.004	0.97	0.59	Benign	− 0.756	Neutral
	K109E	0.075	0.810	0.84	0.93	Probably damaging	0.781	Neutral
	E120K	− 0.524	0.025	0.95	0.81	Benign	− 0.569	Neutral
	D159Y	− 1.942	0.075	0.93	0.84	Benign	0.377	Neutral
	K211N	0.586	0.000	1.00	0.00	Benign	0.504	Neutral
	P213S	0.693	0.000	1.00	0.00	Benign	0.689	Neutral
	E226K	− 0.685	0.000	1.00	0.00	Benign	0.479	Neutral
	N227K	0.474	0.033	0.95	0.82	Benign	− 0.443	Neutral
	K228M	− 0.164	0.002	0.99	0.30	Benign	− 0.258	Neutral
	N238S	0.012	0.000	1.00	0.00	Benign	− 1.585	Neutral
	D278Y	− 0.434	1.000	0.00	1.00	Probably damaging	− 2.728	Deleterious
F2 domain	K301N	1.225	0.592	0.87	0.91	Probably damaging	0.388	Neutral
	K304I	− 0.007	0.000	1.00	0.00	Benign	− 0.861	Neutral
	L305V	1.551	0.825	0.84	0.93	Probably damaging	− 0.204	Neutral
	D329N	0.021	0.777	0.85	0.92	Probably damaging	− 0.69	Neutral
	N400K	− 0.660	0.001	0.99	0.15	Benign	− 0.002	Neutral
	V402A	− 0.047	0.006	0.97	0.75	Benign	− 0.272	Neutral
	Q407K	0.032	0.001	0.99	0.15	Benign	0.539	Neutral
	Q407E	− 1.657	0.000	1.00	0.00	Benign	0.149	Neutral
	E415A	0.020	0.198	0.92	0.88	Benign	− 1.077	Neutral

^a^HumDiv is the preferred model for evaluating rare alleles, dense mapping of regions identified by genome-wide association studies, and analysis of natural selection

^b^HumDivA >  = 0.953, probably damaging; 0.953 > HumDivB >  = 0.432, possibly damaging; 0.432 > HumDivC >  = 0.0024, benign

^c^The default threshold is − 2.5; that is, variants with a score equal to or below − 2.5 are considered "deleterious" and variants with a score above − 2.5 are considered "neutral."

**Fig. 4:**
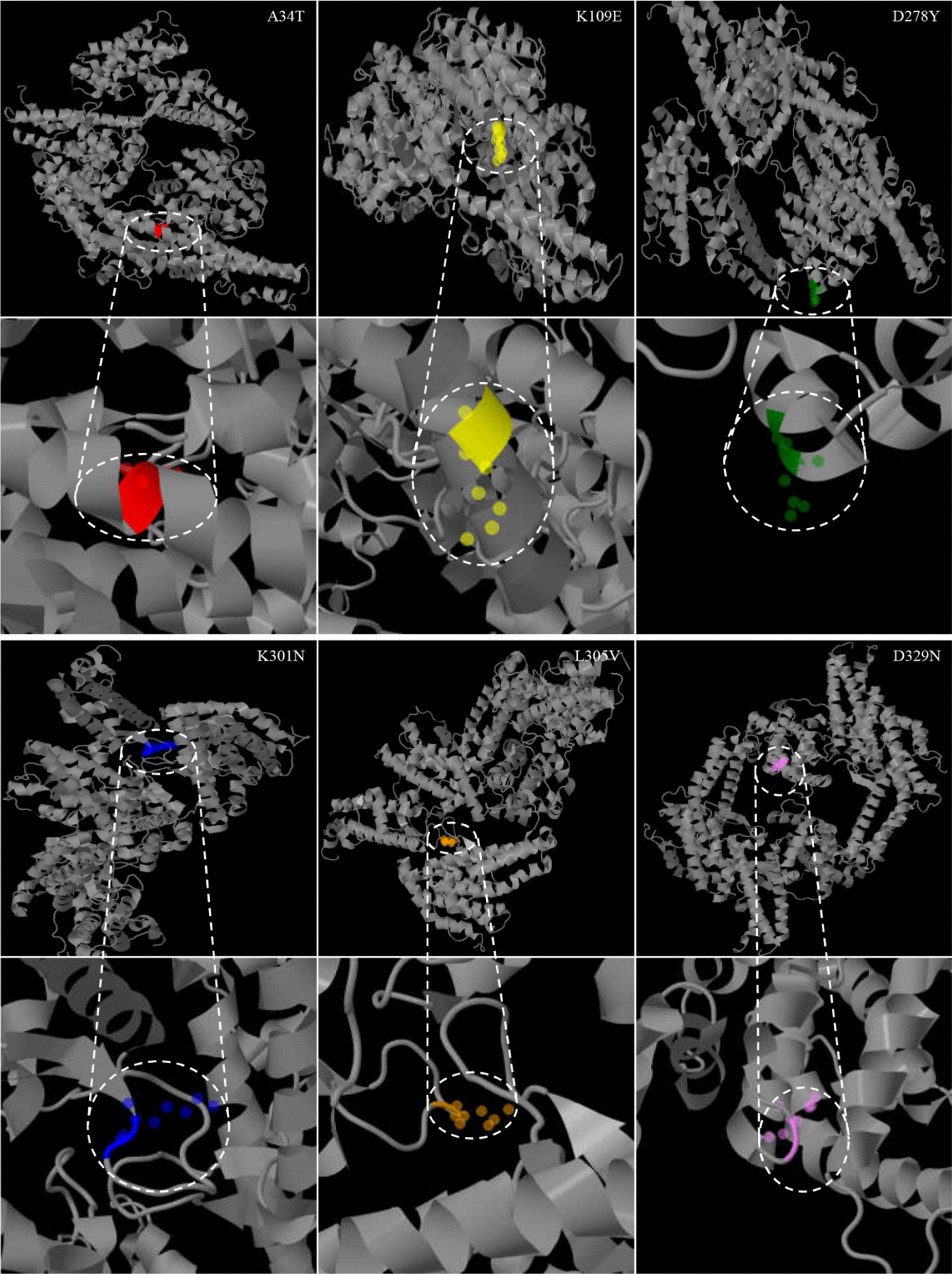
Predicted three-dimensional structure of probably damaging mutation sites in *Pf*EBA-175 region II

Furthermore, mutation effect prediction was conducted among these 24 sites. As shown in Table 3, the mutation D278Y was predicted to be deleterious using the PROVEAN program (score equal to or below − 2.5). According to the HumDiv score predicted by the PolyPhen-2 program, 6 amino acid changes were probably damaging. Among these probably damaging mutants, A34T, K109E, K301N, L305V, and D329N tended to destabilize the protein structure (ΔΔG > 0); D278Y was an exception to this pattern (Table 3). The PolyPhen-2 program was used to predict that the damaging mutation sites probably affect the structure and function of *Pf*EBA-175 region II. The protein structural bioinformatics analysis indicated that these mutation sites were located in the corner area of *Pf*EBA-175 region II (Fig. 4).

### Nucleotide diversity and natural selection of *Pf*EBA-175 region II in global *P. falciparum isolates*

**Fig. 5:**
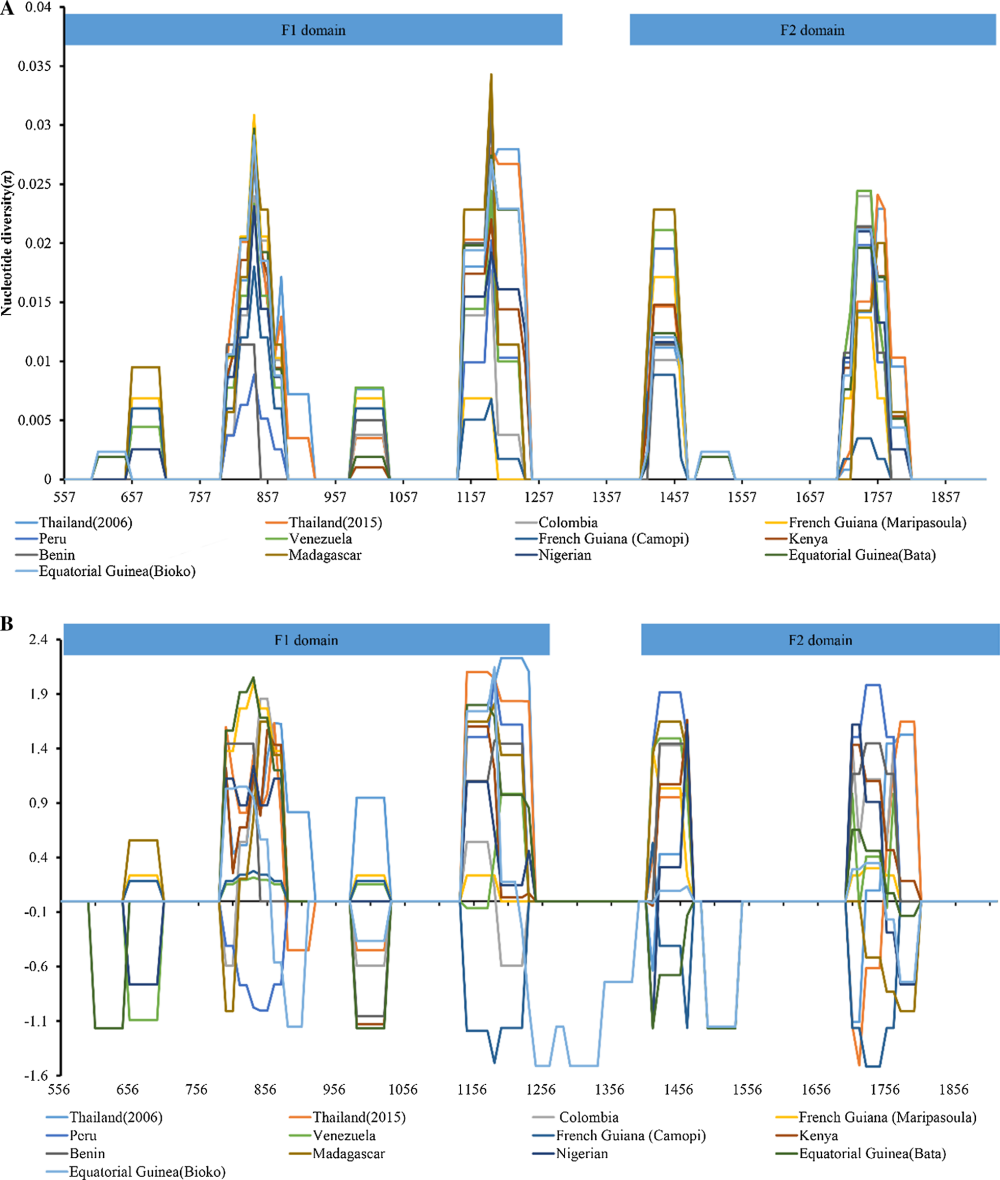
Global nucleotide diversity and natural selection of *Pf*EBA-175 region II. **A** Nucleotide diversity. Sliding window plot analysis shows the nucleotide diversity (π) value across *Pf*EBA-175 region II from different countries. A window size of 100 bp and a step size of 5 bp were used. **B** Natural selection. Sliding window calculation of Tajima’s D statistic was performed for *Pf*EBA-175 region II. A window size of 100 and a step size of 5 were used

Nucleotide diversity of *Pf*EBA-175 region II in global isolates, including samples from Thailand, Colombia, French Guiana (Maripasoula), Peru, Venezuela, French Guiana (Camopi), Bata, Bioko, Kenya, Benin, Madagascar, and Nigeria, were analysed. K values of *Pf*EBA-175 region II from Thailand isolates (Thailand 2006, K = 6.129 and Thailand 2015, K = 6.224) were higher than those from other geographical areas (Table 4). The nucleotide diversities of *Pf*EBA-175 region II from different countries or regions were different by geographical area. The level of nucleotide diversity across the *Pf*EBA-175 region II from Thailand, Bata and Madagascar *P. falciparum* isolates were similarly (Thailand 2006, π = 0.00437; Thailand 2015, π = 0.00444; Bata, π = 0.00411; Madagascar, π = 0.00411) were higher than from other global isolates (Table 4). A sliding window plot of the π values of *Pf*EBA-175 region II from different geographical areas shows that their sequences have similar patterns of nucleotide diversity, and there are peaks in the F1 domain (Fig. 5a). Except for the isolates from the Bioko and French Guiana (Camopi), the sequences of *Pf*EBA-175 regions II from other countries showed positive Tajima’s D values, which suggested a pattern of balancing selection across the majority of *P. falciparum* samples (Table 4). Additionally, the sliding window plot analysis showed that the sequences of global *Pf*EBA-175 region II had different pattern in Tajima’s D among the different geographical origins (Fig. 5b).

**Table 4:** Estimates of DNA sequence polymorphism and tests of neutrality at *Pf*EBA-175 region II among global *P. falciparum* isolates

	Sample number	S	H	K	Hd + SD	π ± SD	dn/ds	Tajima's D	Fu and Li's D	Fu and Li's F
Thailand (2006)	48	16	17	6.129	0.895 ± 0.024	0.00437 ± 0.00016	0.004	1.88575	1.20573	1.7012
Thailand (2015)	32	17	24	6.224	0.976 ± 0.015	0.00444 ± 0.00025	0.005	1.33504	0.90921	1.23017
Colombia	20	11	3	3.979	0.568 ± 0.086	0.00284 ± 0.00052	0.004	1.00475	1.43716	1.52132
French Guiana (Maripasoula)	15	10	3	4.114	0.600 ± 0.109	0.00294 ± 0.00053	0.004	1.28567	1.41829	1.58571
Peru	30	9	4	3.425	0.595 ± 0.057	0.00244 ± 0.00022	0.003	1.57791	1.36748	1.67324
Venezuela	9	13	6	5.278	0.889 ± 0.091	0.00377 ± 0.00065	0.005	0.49334	0.67031	0.70214
French Guiana (Camopi)	23	12	4	2.458	0.498 ± 0.111	0.00175 ± 0.00051	0.002	− 0.84514	− 0.6893	− 0.8564
Equatorial Guinea (Bata)	21	17	17	5.762	0.976 ± 0.023	0.00411 ± 0.00028	0.005	0.56395	0.07543	0.25614
Equatorial Guinea (Bioko)	28	23	20	5.704	0.958 ± 0.025	0.00407 ± 0.00035	0.005	− 0.27018	− 0.41803	− 0.4361
Kenya	39	15	21	5.117	0.895 ± 0.037	0.00365 ± 0.00025	0.005	1.13808	1.17499	1.37173
Benin	8	8	7	4.036	0.964 ± 0.077	0.00288 ± 0.00037	0.004	1.48946	1.04971	1.27432
Madagascar	7	11	7	5.762	1.000 ± 0.076	0.00411 ± 0.00056	0.005	0.96043	0.74709	0.8729
Nigeria	30	14	16	4.625	0.816 ± 0.073	0.0033 ± 0.00035	0.004	0.74635	0.75513	0.88276

S: segregating sites, K: average number of pairwise nucleotide differences, H: number of haplotypes, Hd: haplotype diversity, π: observed average pairwise nucleotide diversity, dN: the number of synonymous substitutions per site, dS: the number of nun-synonymous substitutions per site, *P < 0.05

### Recombination and linkage disequilibrium

The minimum number of recombination events between adjacent polymorphic sites (Rm) of the *Pf*EBA-175 region II from Bioko and Bata were estimated as 7 and 5, respectively (Table 5). The estimate of recombination parameter between adjacent sites (Ra) and per gene (Rb) of Bata isolates were, respectively, 0.0721 and 101, and were higher than those of other global isolates (Table 5). The lowest R values were predicted for the *Pf*EBA-175 region II sequences from Colombia and French Guiana (Camopi). The LD index (R^2^) of global *Pf*EBA-175 genes in the study decrease with increasing distance across this gene (Additional file 4).

**Table 5:** Comparison of recombination events of global *Pf*EBA-175 region II

	Ra	Rb	Rm
Thailand (2006)	0.0166	23.2	4
Thailand (2015)	0.0368	51.5	6
Colombia	0	0.001	0
French Guiana (Maripasoula)	0.0014	2	0
Peru	0.0011	1.6	1
Venezuela	0.0199	27.9	3
French Guiana (Camopi)	0	0.001	0
Equatorial Guinea (Bata)	0.0721	101	5
Equatorial Guinea (Bioko)	0.0511	71.6	7
Kenya	0.0219	30.6	5
Benin	0.0193	27	2
Madagascar	0.0351	49.1	2
Nigeria	0.0094	13.1	5

Ra: estimate of recombination between adjacent sites, Rb: estimate of recombination per gene, Rm: minimum number of recombination events

### Phylogenetic relations among *Pf*EBA-175 region II

The phylogenetic relations among 310 *Pf*EBA-175 region II of global *P. falciparum* isolates (Additional file 4) was assay by a neighbor-joining (NJ) tree. In the tree, *P. falciparum* isolated from Africa formed various clade rooted with Asia or South America *P. falciparum* isolated. But the *P. falciparum* isolated from Asia or South America *P. falciparum* isolated either formed a single clade rooted or formed a cluster with Africa *P. falciparum* isolated. And the *P. falciparum* isolated from Asia or South America was less correlation (Additional file 5), which may suggested different diversity pattern of these geographical areas.

### Nucleotide differentiation among global *Pf*EBA-175 region II

To assay the nucleotide differentiation of global *Pf*EBA-175, the isolates from different geographical areas were evaluated using FST values (Table 6). FST values between different geographical *Pf*EBA-175 populations varied from 0.00033 (*P* > 0.05) between Equatorial Guinea (Bioko) and Equatorial Guinea (Bata) to 0.44629 (*P* < 0.05) between French Guiana (Camopi) and Colombia, excluding negative values. Two negative values appeared in the FST analysis, which might due to the close geographical location of the sample source and the short sequence interval analysed in this study.

**Table 6:** Pairwise FST estimates for *Pf*EBA-175 region II

	Thailand (2006)	Thailand (2015)	Colombia	Maripasoula	Peru	Venezuela	Camopi	Equatorial Guinea (Bata)	Equatorial Guinea (Bioko)	Kenya	Benin	Madagascar	Nigeria
Thailand (2006)		–	+	+	+	+	+	−	+	+	+	+	+
Thailand (2015)	0.00393		+	+	+	+	+	-	+	+	+	+	+
Colombia	0.25314	0.2129		+	+	−	+	+	+	+	+	-	+
French Guiana (Maripasoula)	0.30439	0.28641	0.22479		+	−	−	+	+	+	+	+	+
Peru	0.24558	0.22263	0.27504	0.23791		−	+	+	+	+	-	+	+
Venezuela	0.20447	0.18693	0.15507	0.02166	0.01433		+	+	+	-	-	+	+
French Guiana (Camopi)	0.4156	0.40711	0.44629	0.02594	0.35022	0.10835		+	+	+	+	+	+
Equatorial Guinea(Bata)	0.0468	0.04319	0.08761	0.21439	0.1548	0.11888	0.36412		−	-	−	−	−
Equatorial Guinea(Bioko)	0.06345	0.07981	0.18142	0.22867	0.0884	0.09149	0.34311	0.00033		+	−	−	+
Kenya	0.11567	0.08697	0.06495	0.13286	0.16542	0.07581	0.29154	0.00954	0.06172		−	+	−
Benin	0.14331	0.15873	0.19853	0.24186	0.09564	0.08141	0.361	0.03404	− 0.0166	0.05124		−	−
Madagascar	0.14229	0.11334	0.09085	0.21081	0.21953	0.1371	0.37513	0.04831	0.07368	0.05832	0.11505		+
Nigeria	0.15176	0.13481	0.09872	0.13183	0.18954	0.08867	0.28478	0.03196	0.08477	-0.0118	0.04562	0.11973	

FST values are shown in the lower left quadrant; + indicated statistically significant; − indicated no statistically significant. FST: a measure of genetic differentiation between populations (range from − 1 to + 1)

### Haplotype network analysis

**Fig. 6:**
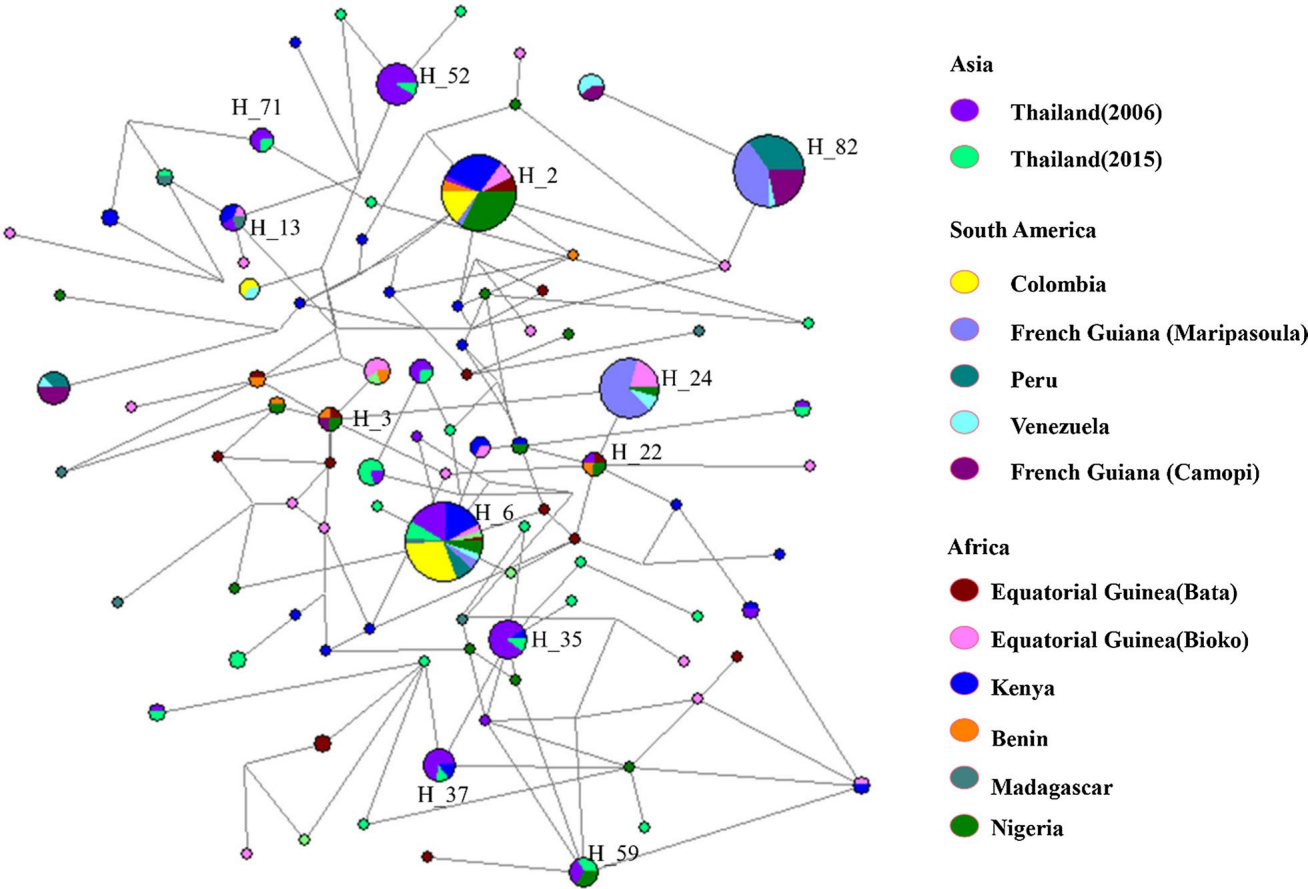
Haplotype network of *Pf*EBA-175 region II globally. Each region of *Pf*EBA-175 is marked by a different color

The haplotypes of *Pf*EBA-175 from the global *P. falciparum* population analysed with the haplotype network showed a complex relationship-dense network (Fig. 6). A total of 92 haplotypes were identified in 310 *Pf*EBA-175 sequences, of which 67.39% (62) were singletons. Haplotype prevalence ranged from 0.32 to 13.14%. The most prevalent haplotype was haplotype 6 (H_6), with a frequency of 13.14%. H_2, H_24, H_35, H_52, and H_82 were other major haplotypes with a high prevalence (3.21 to 12.82%). Only haplotype 6 contained haplotypes from three continents. H_2, H_3, H_13, H_22, H_24, H_35, H_37, H_59, and H_71 were composed of haplotypes from two continents (South American and African populations or African and Asian populations). Haplotypes from Equatorial Guinea (including Bioko Island and Bata district) were mostly scattered with no particular distribution pattern.

## Discussion

Malaria monitoring and evaluation is of great significance for malaria control and assessment of the impact of intervention strategies. In addition, monitoring the genetic diversity of candidate vaccine antigens in global malaria isolates circulating in endemic areas is essential for designing an efficient and protective malaria vaccine [36]. This study provides an initial insight into the *Pf*EBA-175 gene dimorphism of *P. falciparum* in Equatorial Guinea. In the assessment of the frequencies of the dimorphic allele of *Pf*EBA-175 gene on *P. falciparum* merozoites, the F-fragment was predominated, which accounted for 56.86% and 71.92% of samples from Bata and Bioko, respectively, while the C-fragment was only 29.41% and 16.25%, respectively (Table 1). It is obvious that the F-fragment was observed at a higher frequency than the C-fragment. These findings are consistent with those of three independent research groups in geographic areas highly endemic for malaria in Burkina Faso [10], Ghana [13], and Gabon [37], where the F-fragment was also observed to be higher. In contrast, the study results from the Sudan [15] and Brazil [8] showed that the C-fragment was much higher. The different distributions of the F-fragment and C-fragment of *Pf*EBA-175 in *P. falciparum* isolates among geographical areas may be due to random shifts in parasite allele frequencies in different geographic regions [13]. Interestingly, the C-fragment was most common in areas with a lower frequency of mixed infection. The previous study showed that geographic areas where higher F-fragment frequency was observed also had a higher frequency of mixed infections [10, 13, 37], and similar results were also found in the geographic areas of this study. At the same time, the study also revealed the genetic polymorphism and molecular evolution of region II of the *Pf*EBA-175 gene of *P. falciparum* from Equatorial Guinea. The 49 sequences of *Pf*EBA-175 region II from Equatorial Guinea populations (including Bioko Island and Bata district) compared to the *Pf*EBA-175 region II of *P. falciparum* (Gene ID: XM_001349171.2) showed 20 different haplotypes. The results showed that the F1 domain had more mutations than the F2 domain. It was revealed that the sequence conservation was higher in the F2 domain than in the F1 domain, suggesting a greater selective pressure on the F2 domain, which is in accordance with the observation that binding is dependent on the F2 domain to a greater extent than the F1 domain [38]. Although the distribution patterns and genetic diversity of *Pf*EBA-175 found in Bioko Island and Bata district were similar to those of other global isolates, several differences between them were identified in this study.

The π value of global *Pf*EBA-175 isolates ranged from 0.00175 (Camopi of French Guiana) to 0.00444 (Thailand 2006) in the study. Among, the π value of *Pf*EBA-175 isolates from Asia (isolates in Thailand collected in 2006 and 2015) were higher than those isolates from Africa and South America. In fact, the region II of *Pf*EBA-175 is the largest target of the host’s immune system. The high number of genetic polymorphisms of *Pf*EBA-175 region II from Bioko Island and Bata district indicated that these geographical areas are under the selection of host immune pressure during evolution. The previous study showed that IgG1 mouse monoclonal antibodies R217 and R218 recognize the F2 and F1 domains in *Pf*EBA-175 region II, and a combination of R217 and R218 could block *Pf*EBA-175 binding to erythrocytes and inhibit parasite growth [39, 40]. However, in this study, region II of *Pf*EBA-175 showed high levels of polymorphism at the gene and amino acid levels, which were also found in the previous study [41], suggesting that the above region may affect vaccine effectiveness. Interestingly, the distribution patterns of nucleotide and amino acid diversity of *Pf*EBA-175 isolates in this study indicated that *Pf*EBA-175 may have similar genetic diversity across many endemic areas. The dn/ds value of *Pf*EBA-175 region II of global isolates is positive (Table 4), suggesting the involvement of balancing selection. Except for the isolates from Bioko Island and French Guiana (Camopi), *Pf*EBA-175 from most geographical areas had positive Tajima’s D values, indicating that the gene was under balancing selection in these areas. Additionally, the isolates from Bioko Island and French Guiana (Camopi) had similar patterns on the sliding plot analysis of Tajima’s D and showed differences from the *Pf*EBA-175 of other isolates from other areas. The positive value of Tajima’s D for the *Pf*EBA-175 gene indicated isolation with balancing selection, whereas the negative values in some populations can be interpreted as a signature of purifying selection or an expansion in the population size during recent parasite evolutionary history [42]. Similarly to the Tajima’s D values, the Fu and Li’s D and F values were positive in most areas, indicating balancing selection on the *Pf*EBA-175 gene globally, except for Bioko Island and French Guiana (Camopi), where these values were negative. The recombination also contributes to the genetic diversity of *Pf*EBA-175 under natural selection. This study revealed that *Pf*EBA-175 isolates from Bioko Island have a high level of recombination, which is consistent with the previous study [41]. The high recombination level was found in the *Pf*EBA-175 isolates from Bioko Island compared to other geographical areas, which may be attributed to the relatively independent environment of Bioko Island.

The FST value is an important parameter that was used to analysed the overall genetic differentiation. The results indicated that the FST values of *Pf*EBA-175 isolates of Bioko Island had a lower level of genetic differentiation with isolates from Asia or Africa (Table 6). In addition, a moderate level of genetic differentiation was found between Bioko Island and South America, and similar results were found between Bata district and South America (Table 6). However, only lower levels of genetic differentiation were found between Bata district and Asia or Africa. In addition, the results indicated that FST values of *Pf*EBA-175 populations from the same geographical areas were relatively low (Table 6). The negative values of FST, which were also found in the previous study of *Pf*AMA-1 [43], may be attributed to the limited number of samples. Interestingly, the FST values of Bioko Island and Bata district with French Guiana (Camopi) were 0.34311 and 0.36412 (Table 6), which indicated geographical isolation in these areas. The average FST values of *Pf*EBA-175 global isolates showed differently, which indicated that *Pf*EBA-175 has genetic differentiation among the parasite populations of the world.

To assay the haplotype network of *Pf*EBA-175 among the global isolates is important for developing an effective malaria vaccine based on this gene. In this study, 92 different haplotypes were identified from 310 *Pf*EBA-175 sequences. Haplotype network analysis showed that the haplotypes of Bioko Island and Bata district were scattered among other haplotypes from different geographical areas, which was similar to the pattern found for another candidate vaccine gene, *Pf*AMA-1 [43]. In this study, 26 of the 49 isolates from the haplotypes in Equatorial Guinea (including Bioko Island and Bata district) were shared with other isolates from Africa, Asia or South America, which suggested that the Bioko Island and Bata district *Pf*EBA-175 isolates were not independent of other global isolates. On the other hand, there were 62 haplotypes that were limited to singletons (only observed in 1 sequence), which were found in the *Pf*EBA-175 isolates from Africa (Bioko and Bata District 23, Kenya 11, Benin 1, Madagascar 4, Nigeria 8) and Asia (Thailand 15). This finding suggested that the *Pf*EBA-175 of African isolates had higher genetic differentiation and provided an insight for the development of a vaccine based on *Pf*EBA-175 in consideration of its diversity in different areas.

## Conclusions

In this study, the frequencies of dimorphic alleles of *Pf*EBA-175 gene on Bioko Island and in Bata district and the overall pattern of genetic diversity of *Pf*EBA-175 from global isolates were analysed. There is no significant difference in the frequency of dimorphic alleles of the *Pf*EBA-175 gene between the island and the mainland. The distribution patterns and genetic diversity of *Pf*EBA-175 from global isolates showed that *Pf*EBA-175 had a high genetic polymorphism. Compared with global *Pf*EBA-175 isolates, high level of recombination events were observed on Bioko Island and in Bata district, suggesting that natural selection and intragenic recombination might be the main drivers of genetic diversity in global *Pf*EBA-175. Additionally, the high level of nucleotide diversity and natural selection indicated that strong natural selection is occurring under host immune pressure during evolution. This study provides evidence for the continuous monitoring of *Pf*EBA-175 nucleotide and amino acid changes of global *P. falciparum* isolates, and provides useful information for developing an effective malaria vaccine.

## Supplementary Information

**Additional file 1.** Ethical approval letter (Spanish version).

**Additional file 2.** Ethical approval letter (Chinese version).

**Additional file 3.** Recombination events of global *Pf*EBA-175 genes.

**Additional file 4.** Global PfEBA-175 region II acquired from NCBI and sequencing in the study.

**Additional file 5.** Phylogenetic tree analysis of Global PfEBA-175 region II.

## Data Availability

All data generated or analysed during this study are included in this published article [and its additional files].

## Abbreviations

*Pf*EBA-175: *Plasmodium falciparum* Erythrocyte binding antigen-175
MCDI: Medical Care Development International
BIMCP: Bioko Island Malaria Control Project
PDCs: Private diagnostic clinics
PCR: Polymerase chain reaction
Ra: Recombination between adjacent sites
Rb: Recombination of per gene
Rm: The minimum number of recombination events
FST: Fixation Index
*Pf*AMA-1: *Plasmodium falciparum* Apical membrane antigen-1
