# Supplementary figures and images for: Population genetic analysis of the Plasmodium falciparum erythrocyte binding antigen-175 (EBA-175) gene in Equatorial Guinea
Source: Malar J. 2021 Sep 19;20:374. doi: 10.1186/s12936-021-03904-x (PMC8451130; doi:10.1186/s12936-021-03904-x)

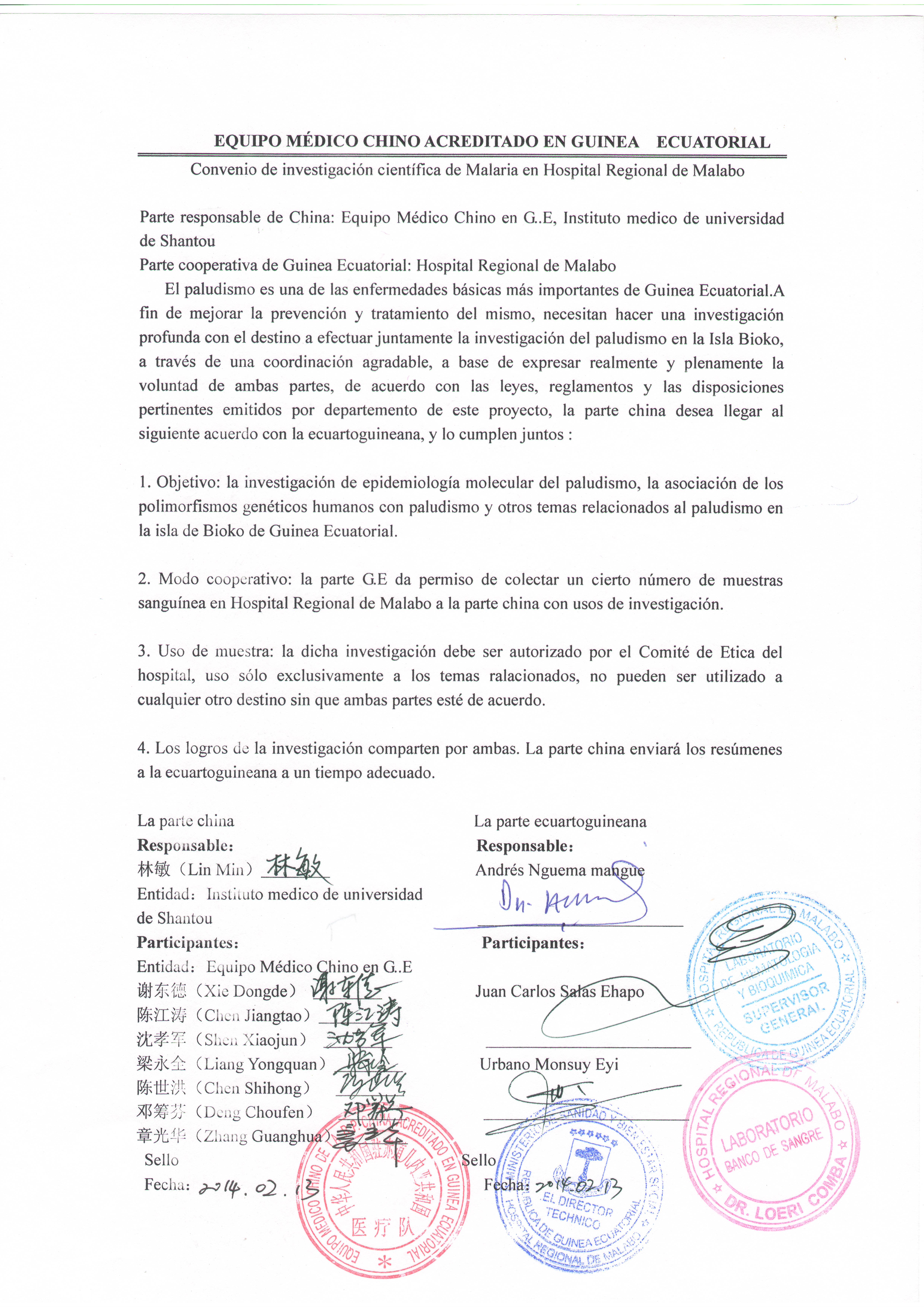

Supplement: Supplementary file 1 — Additional file 1. Ethical approval letter (Spanish version). [file 12936_2021_3904_MOESM1_ESM.jpg]

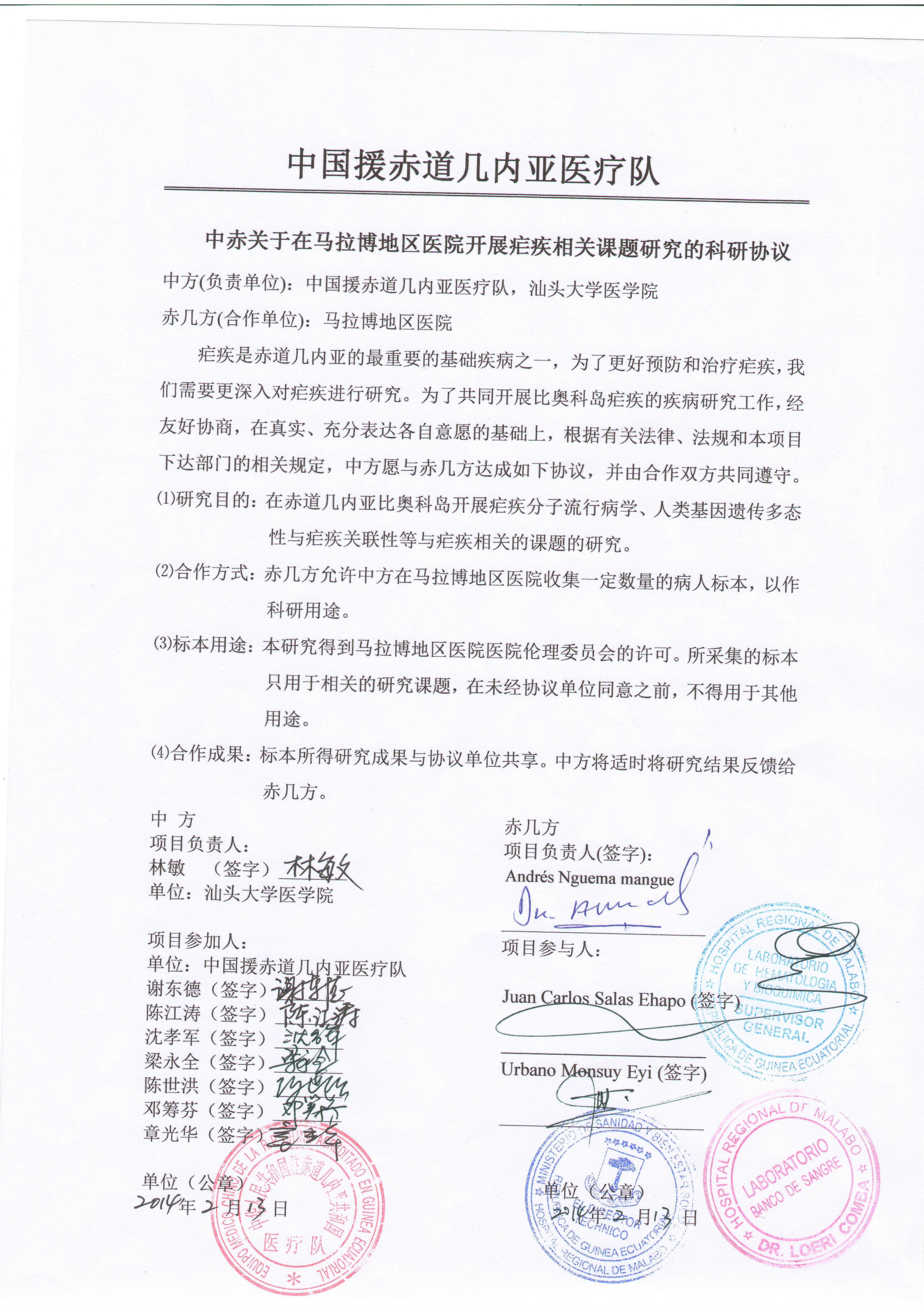

Supplement: Supplementary file 2 — Additional file 2. Ethical approval letter (Chinese version). [file 12936_2021_3904_MOESM2_ESM.jpg]
