# Supplementary material for: Population genetic analysis of the Plasmodium falciparum erythrocyte binding antigen-175 (EBA-175) gene in Equatorial Guinea
Source: Malar J. 2021 Sep 19;20:374. doi: 10.1186/s12936-021-03904-x (PMC8451130; doi:10.1186/s12936-021-03904-x)
Supplement: Supplementary file 3 — Additional file 3. Recombination events of global PfEBA-175 genes. [file 12936_2021_3904_MOESM3_ESM.pdf]

>Plasmodium falciparum 3D7 EBA715_XM_001349171.2
TATGTATGTATTCCTGATCGTAGAATCCAATTATGCATTGTTAATCTTAGCATTATTAAAA
CATATACAAAAGAGACCATGAAGGATCATTTCATTGAAGCCTCTAAAAAAGAATCTCAA
CTTTTGCTTAAAAAAAAATGATAACAAATATAATTCTAAATTTTGTAATGATTTGAAGAATA
GTTTTTTAGATTATGGACATCTTGCTATGGGAAATGATATGGATTTTGGAGGTTATTCAAC
TAAGGCAGAAAACAAAATTCAAGAAGTTTTTAAAGGGGCTCATGGGGAAATAAGTGAAC
ATAAAATTAAAAATTTTAGAAAAAAAATGGTGGAATGAATTTAGAGAGAAACTTTGGGAA
GCTATGTTATCTGAGCATAAAAATAATATAAATAATTGTAAAAATATTCCCCAAGAAGAA
TTACAAATTACTCAATGGATAAAAGAATGGCATGGAGAATTTTTGCTTGAAAGAGATAAT
AGATCAAAATTGCCAAAAAGTAAATGTAAAAATAATACATTATATGAAGCATGTGAGAA
GGAATGTATTGATCCATGTATGAAATATAGAGATTGGATTATTAGAAGTAAATTTGAATG
GCATACGTTATCGAAAGAATATGAAACTCAAAAAGTTCCAAAGGAAAATGCGGAAAATT
ATTTAATCAAAATTTCAGAAAACAAGAATGATGCTAAAGTAAGTTTATTATTGAATAATT
GTGATGCTGAATATTCAAAATATTGTGATTGTAAACATACTACTACTCTCGTTAAAAGCGT
TTTAAATGGTAACGACAATACAATTAAGGAAAAGCGTGAACATATTGATTTAGATGATTT
TTCTAAATTTGGATGTGATAAAAATTCCGTTGATACAAACACAAAGGTGTGGGAATGTAA
AAAACCTTATAAATTATCCACTAAAGATGTATGTGTACCTCCGAGGAGGCAAGAATTATG
TCTTGGAAACATTGATAGAATATACGATAAAAACCTATTAATGATAAAAGAGCATATTCT
TGCTATTGCAATATATGAATCAAGAATATTGAAACGAAAATATAAGAATAAAGATGATAA
AGAAGTTTGTAAAATCATAAATAAAACTTTCGCTGATATAAGAGATATTATAGGAGGTAC
TGATTATTGGAATGATTTGAGCAATAGAAAATTAGTAGGAAAAATTAACACAAATTCAAA
TTATGTTCACAGGAATAAACAAAATGATAAGCTTTTTCGTGATGAGTGGTGGAAAGTTAT
TAAAAAAGATGTATGGAATGTGATATCATGGGTATTCAAGGATAAAACTGTTTGTAAAGA
AGATGATATTGAAAATATACCACAATTCTTCAGATGGTTTAGTGAATGGGGTGATGATTA
TTGCCAGGATAAAACAAAAATG
>Equatorial Guinea (Bata) 2019T19050F_MW691428.1
TATGTATGTATTCCTGATCGTAGAATCCAATTATGCATTGTTAATCTTAGCATTATTAAAA
CATATACAAAAGAGACCATGAAGGATCATTTCATTGAAGCCTCTAAAAAAGAATCTCAA
CTTTTGCTTAAAAAAAAATGATAACAAATATAATTCTAAATTTTGTAATGATTTGAAGAATA
GTTTTTTAGATTATGGACATCTTGCTATGGGAAATGATATGGATTTTGGAGGTTATTCAAC
TAAGGCAGAAAACAAAATTCAAGAAGTTTTTAAAGGGGCTCATGGGGAAATAAGTGAAC
ATAAAATTAAAAATTTTAGAAAAAAAATGGTGGAATGAATTTAGAGAGAAACTTTGGGAA
GCTATGTTATCTGAGCATAAAAATAATATAAATAATTGTAAAAATATTCCCCAAGAAGAA
TTACAAATTACTCAATGGATAAAAGAATGGCATGGAGAATTTTTGCTTGAAAGAGATAAT
AGATCAAAATTGCCAAAAAGTAAATGTAAAAATAATACATTATATGAAGCATGTGAGAA
GGAATGTATTGATCCATGTATGAAATATAGAGATTGGATTATTAGAAGTAAATTTGAATG
GCATACGTTATCGAAAGAATATGAAACTCAAAATGTTTCAAAGGAAAATGCGGAAAATT
ATTTAATCAAAATTTCAAAAAAACAAGAATGATGCTAAAGTAAGTTTATTATTGAATAATTG
TGATGCTGAATATTCAAAATATTGTGATTGTAAACATACTACTACTCTCGTTAAAAGCGTT
TTAAATGGTAACGACAATACAATTAAGGAAAAGCGTGAACATATTGATTTAGATGATTTT
TCTAAATTTGGATGTGATAAAAATTCCGTTGATACAAACACAAAGGTGTGGGAATGTAAA
AAACCTTATAAATTATCCACTAAAGATGTATGTGTACCTCCGAGGAGGCAAGAATTATGT
CTTGGAAACATTGATAGAATATACGATAAAAACCTATTAATGATAAAAGAGCATATTCTT
GCTATTGCAATATATGAATCAAGAATATTGAAACGAAAATATAAGAATAAAGATGATAA

AGAAGTTTGTAAAATCATAAATAAAACTTTCGCTGATATAAGAGATATTATAGGAGGTAC
TGATTATTGGAATGATTTGAGCAATAGAAAATTAGTAGGAAAAATTAACACAAATTCAAA
TTATGTTCACAGGAATAAACAAAATGATAAGCTTTTTCGTGATGAGTGGTGGAAAGTTAT
TAAAAAAGATGTATGGAATGTGATATCATGGGTATTCAAGGATAAAACTGTTTGTAAAGA
AGATGATATTGAAAATATACCACAATTCTTCAGATGGTTTAGTGAATGGGGTGATGATTA
TTGCCAGGATAAAACAAAAATG
>Equatorial Guinea (Bata) 2019T19047F_MW691429.1
TATGTATGTATTCCTGATCGTAGAATCCAATTATGCATTGTTAATCTTAGCATTATTAAAA
CATATACAAAAGAGACCATGAAGGATCATTTCATTGAAGCCTCTAAAAAAGAATCTCAA
CTTTTGCTTAAAAAAAAATGATAACAAATATAATTCTAAATTTTGTAATGATTTGAAGAATA
GTTTTTTAGATTATGGACATCTTGCTATGGGAAATGATATGGATTTTGGAGGTTATTCAAC
TAAGGCAGAAAACAAAATTCAAGAAGTTTTTAAAGGGGCTCATGGGAAAATAAGTGAAC
ATAAAATTAAAAATTTTAGAAAAAAAATGGTGGAATGAATTTAGAGAGAAACTTTGGGAA
GCTATGTTATCTGAGCATAAAAATAATATAAATAATTGTAAAAATATTCCCCAAGAAGAA
TTACAAATTACTCAATGGATAAAAGAATGGCATGGAGAATTTTTGCTTGAAAGAGATAAT
AGATCAAAATTGCCAAAAAGTAAATGTAAAAATAATACATTATATGAAGCATGTGAGAA
GGAATGTATTGATCCATGTATGAAATATAGAGATTGGATTATTAGAAGTAAATTTGAATG
GCATACGTTATCGAAAGAATATGAAACTCAAAATGTTTCAAAGGAAAATGCGGAAAATT
ATTTAATCAAAATTTCAAAAAACAAGAATGATGCTAAAGTAAGTTTATTATTGAATAATTG
TGATGCTGAATATTCAAAATATTGTGATTGTAAACATACTACTACTCTCGTTAAAAGCGTT
TTAAATGGTAACGACAATACAATTAAGGAAAAGCGTGAACATATTGATTTAGATGATTTT
TCTAAATTTGGATGTGATAAAAATTCCGTTGATACAAACACAAAGGTGTGGGAATGTAAA
AAACCTTATAAATTATCCACTAAAGATGTATGTGTACCTCCGAGGAGGCAAGAATTATGT
CTTGGAAACATTGATAGAATATACGATAAAAACCTATTAATGATAAAAGAGCATATTCTT
GCTATTGCAATATATGAATCAAGAATATTGAAACGAAAATATAAGAATAAAGATGATAA
AGAAGTTTGTAAAATCATAAATAAAACTTTCGCTGATATAAGAGATATTATAGGAGGTAC
TGATTATTGGAATGATTTGAGCAATAGAAAATTAGTAGGAAAAATTAACACAAATTCAAA
TTATGTTCACAGGAATAAACAAAATGATAAGCTTTTTCGTGATGAGTGGTGGAAAGTTAT
TAAAAAAGATGTATGGAATGTGATATCATGGGTATTCAAGGATAAAACTGTTTGTAAAGA
AGATGATATTGAAAATATACCACAATTCTTCAGATGGTTTAGTGAATGGGGTGATGATTA
TTGCCAGGATAAAACAAAAATG
>Equatorial Guinea (Bata) 2019T19045F_MW691430.1
TATGTATGTATTCCTGATCGTAGAATCCAATTATGCATTGTTAATCTTAGCATTATTAAAA
CATATACAAAAGAGACCATGAAGGATCATTTCATTGAAGCCTCTAAAAAAGAATCTCAA
CTTTTGCTTAAAAAAAAATGATAACAAATATAATTCTAAATTTTGTAATGATTTGAAGAATA
GTTTTTTAGATTATGGACATCTTGCTATGGGAAATGATATGGATTTTGGAGGTTATTCAAC
TAAGGCAGAAAACAAAATTCAAGAAGTTTTTAAAGGGGCTCATGGGAAAATAAGTGAAC
ATGAAATTAAAAATTTTAGAAAAAAAATGGTGGAATGAATTTAGAGAGAAACTTTGGGAA
GCTATGTTATCTGAGCATAAAAATAATATAAATAATTGTAAAAATATTCCCCAAGAAGAA
TTACAAATTACTCAATGGATAAAAGAATGGCATGGAGAATTTTTGCTTGAAAGAGATAAT
AGATCAAAATTGCCAAAAAGTAAATGTAAAAATAATACATTATATGAAGCATGTGAGAA
GGAATGTATTGATCCATGTATGAAATATAGAGATTGGATTATTAGAAGTAAATTTGAATG
GCATACGTTATCGAAAGAATATGAAACTCAAAATGTTTCAAAGGAAAATGCGGAAAATT
ATTTAATCAAAATTTCAAAAAACAAGAATGATGCTAAAGTAAGTTTATTATTGAATAATTG

TGATGCTGAATATTCAAAATATTGTGATTGTAAACATACTACTACTCTCGTTAAAAGCGTT
TTAAATGGTAACGACAATACAATTAAGGAAAAGCGTGAACATATTGATTTAGATGATTTT
TCTAAATTTGGATGTGATAAAAATTCCGTTGATACAAACACAAAGGTGTGGGAATGTAAA
AAACCTTATATATTATCCACTAAAGATGTATGTGTACCTCCGAGGAGGCAAGAATTATGT
CTTGGAAACATTGATAGAATATACGATAAAAACCTATTAATGATAAAAGAGCATATTCTT
GCTATTGCAATATATGAATCAAGAATATTGAAACGAAAATATAAGAATAAAGATGATAA
AGAAGTTTGTAAAATCATAAATAAAACTTTCGCTGATATAAGAGATATTATAGGAGGTAC
TGATTATTGGAATGATTTGAGCAATAGAAAATTAGTAGGAAAAATTAACACAAATTCAAA
TTATGTTCACAGGAATAAACAAAATGATAAGCTTTTTCGTGATGAGTGGTGGAAAGTTAT
TAAAAAAGATGTATGGAATGTGATATCATGGGTATTCAAGGATAAAACTGTTTGTAAAGA
AGATGATATTGAAAATATACCACAATTCTTCAGATGGTTTAGTGAATGGGGTGATGATTA
TTGCCAGGATAAAACAAAAATG
>Equatorial Guinea (Bata) 2019T19044F_MW691431.1
TATGTATGTATTCCTGATCGTAGAATCCAATTATGCATTGTTAATCTTAGCATTATTAAAA
CATATACAAAAGAGACCATGAAGGATCATTTCATTGAAGCCTCTAAAAAAGAATCTCAA
CTTTTGCTTAAAAAAAATGATAACAAATATAATTCTAAATTTTGTAATGATTTGAAGAATA
GTTTTTTAGATTATGGACATCTTGCTATGGGAAATGATATGGATTTTGGAGGTTATTCAAC
TAAGGCAGAAAACAAAATTCAAGAAGTTTTTAAAGGGGCTCATGGGGAAATAAGTGAAC
ATAAAATTAAAAATTTTAGAAAAGAATGGTGGAATGAATTTAGAGAGAAACTTTGGGAA
GCTATGTTATCTGAGCATAAAAATAATATAAATAATTGTAAAAATATTCCCCAAGAAGAA
TTACAAATTACTCAATGGATAAAAGAATGGCATGGAGAATTTTTGCTTGAAAGAGATAAT
AGATCAAAATTGCCAAAAAGTAAATGTAAAAATAATACATTATATGAAGCATGTGAGAA
GGAATGTATTGATCCATGTATGAAATATAGAGATTGGATTATTAGAAGTAAATTTGAATG
GCATACGTTATCGAAAGAATATGAAACTCAAAAAGTTCCAAAGGAAAATGCGGAAAATT
ATTTAATCAAAATTTCAGAAAACAAGAATGATGCTAAAGTAAGTTTATTATTGAATAATT
GTGATGCTGAATATTCAAAATATTGTGATTGTAAACATACTACTACTCTCGTTAAAAGCGT
TTTAAATGGTAACGACAATACAATTAAGGAAAAGCGTGAACATATTGATTTAGATGATTT
TTCTAAATTTGGATGTGATAAAAATTCCGTTGATACAAACACAAAGGTGTGGGAATGTAA
AAACCCTTATATATTATCCACTAAAGATGTATGTGTACCTCCGAGGAGGCAAGAATTATG
TCTTGGAAACATTGATAGAATATACGATAAAAACCTATTAATGATAAAAGAGCATATTCT
TGCTATTGCAATATATGAATCAAGAATATTGAAACGAAAATATAAGAATAAAGATGATAA
AGAAGTTTGTAAAATCATAAATAAAACTTTCGCTGATATAAGAGATATTATAGGAGGTAC
TGATTATTGGAATGATTTGAGCAATAGAAAATTAGTAGGAAAAATTAACACAAATTCAAA
ATATGTTCACAGGAATAAAAAAAAATGATAAGCTTTTTCGTGATGAGTGGTGGAAAGTTAT
TAAAAAAGATGTATGGAATGTGATATCATGGGTATTCAAGGATAAAACTGTTTGTAAAGA
AGATGATATTGAAAATATACCACAATTCTTCAGATGGTTTAGTGAATGGGGTGATGATTA
TTGCCAGGATAAAACAAAAATG
>Equatorial Guinea (Bata) 2019T19043F_MW691432.1
TATGTATGTATTCCTGATCGTAGAATCCAATTATGCATTGTTAATCTTAGCATTATTAAAA
CATATACAAAAGAGACCATGAAGGATCATTTCATTGAAGCCTCTAAAAAAGAATCTCAA
CTTTTGCTTAAAAAAAATGATAACAAATATAATTCTAAATTTTGTAATGATTTGAAGAATA
GTTTTTTAGATTATGGACATCTTGCTATGGGAAATGATATGGATTTTGGAGGTTATTCAAC
TAAGGCAGAAAACAAAATTCAAGAAGTTTTTAAAGGGGCTCATGGGGAAATAAGTGAAC
ATAAAATTAAAAATTTTAGAAAAAAAATGGTGGAATGAATTTAGAGAGAAACTTTGGGAA

GCTATGTTATCTGAGCATAAAAATAATATAAATAATTGTAAAAATATTCCCCAAGAAGAA
TTACAAATTACTCAATGGATAAAAGAATGGCATGGAGAATTTTTGCTTGAAAGAGATAAT
AGATCAAAATTGCCAAAAAGTAAATGTAAAAATAATACATTATATGAAGCATGTGAGAA
GGAATGTATTGATCCATGTATGAAATATAGAGATTGGATTATTAGAAGTAAATTTGAATG
GCATACGTTATCGAAAGAATATGAAACTCAAAATGTTTCAAAGGAAAATGCGGAAAATT
ATTTAATCAAAATTTCAGAAAAAATGAATGATGCTAAAGTAAGTTTATTATTGAATAATTG
TGATGCTGAATATTCAAAATATTGTGATTGTAAACATACTACTACTCTCGTTAAAAGCGTT
TTAAATGGTAACGACAATACAATTAAGGAAAAGCGTGAACATATTGATTTAGATGATTTT
TCTAAATTTGGATGTGATAAAAAATTCCGTTGATACAAACACAAAGGTGTGGGAATGTAAA
AAACCTTATAAATTATCCACTAAAGATGTATGTGTACCTCCGAGGAGGCAAGAATTATGT
CTTGGAAACATTGATAGAATATACGATAAAAACCTATTAATGATAAAAGAGCATATTCTT
GCTATTGCAATATATGAATCAAGAATATTGAAACGAAAATATAAGAATAAAGATGATAA
AGAAGTTTGTAAAATCATAAATAAAACTTTCGCTGATATAAGAGATATTATAGGAGGTAC
TGATTATTGGAATGATTTGAGCAATAGAAAATTAGTAGGAAAAATTAACACAAATTCAAA
TTATGTTCACAGGAATAAAGAAAATGATAAGCTTTTTCGTGATGCGTGGTGGAAAGTTAT
TAAAAAAGATGTATGGAATGTGATATCATGGGTATTCAAGGATAAAACTGTTTGTAAAGA
AGATGATATTGAAAATATACCACAATTCTTCAGATGGTTTAGTGAATGGGGTGATGATTA
TTGCCAGGATAAAACAAAAATG
>Equatorial Guinea (Bata) 2019T19041F_MW691433.1
TATGTATGTATTCCTGATCGTAGAATCCAATTATGCATTGTTAATCTTAGCATTATTAAAA
CATATACAAAAGAGACCATGAAGGATCATTTCATTGAAGCCTCTAAAAAAGAATCTCAA
CTTTTGCTTAAAAAAAATGATAACAAATATAATTCTAAATTTTGTAATGATTTGAAGAATA
GTTTTTTAGATTATGGACATCTTGCTATGGGAAATGATATGGATTTTGGAGGTTATTCAAC
TAAGGCAGAAAACAAAATTCAAGAAGTTTTTAAAGGGGCTCATGGGGAAATAAGTGAAC
ATAAAATTAAAAATTTTAGAAAAAAATGGTGGAATGAATTTAGAGAGAAACTTTGGGAA
GCTATGTTATCTGAGCATAAAAATAATATAAATAATTGTAAAAATATTCCCCAAGAAGAA
TTACAAATTACTCAATGGATAAAAGAATGGCATGGAGAATTTTTGCTTGAAAGAGATAAT
AGATCAAAATTGCCAAAAAGTAAATGTAAAAATAATACATTATATGAAGCATGTGAGAA
GGAATGTATTGATCCATGTATGAAATATAGAGATTGGATTATTAGAAGTAAATTTGAATG
GCATACGTTATCGAAAGAATATGAAACTCAAAATGTTTCAAAGGAAAATGCGGAAAATT
ATTTAATCAAAATTTCAGAAAAAATGAATGATGCTAAAGTAAGTTTATTATTGAATAATTG
TGATGCTGAATATTCAAAATATTGTGATTGTAAACATACTACTACTCTCGTTAAAAGCGTT
TTAAATGGTAACGACAATACAATTAAGGAAAAGCGTGAACATATTGATTTAGATGATTTT
TCTAAATTTGGATGTGATAAAAAATTCCGTTGATACAAACACAAAGGTGTGGGAATGTAAA
AAACCTTATAAATTATCCACTAAAGATGTATGTGTACCTCCGAGGAGGCAAGAATTATGT
CTTGGAAACATTGATAGAATATACGATAAAAACCTATTAATGATAAAAGAGCATATTCTT
GCTATTGCAATATATGAATCAAGAATATTGAAACGAAAATATAAGAATAAAGATGATAA
AGAAGTTTGTAAAATCATAAATAAAACTTTCGCTGATATAAGAGATATTATAGGAGGTAC
TGATTATTGGAATGATTTGAGCAATAGAAAATTAGTAGGAAAAATTAACACAAATTCAAA
TTATGTTCACAGGAATAAAGAAAATGATAAGCTTTTTCGTGATGCGTGGTGGAAAGTTAT
TAAAAAAGATGTATGGAATGTGATATCATGGGTATTCAAGGATAAAACTGTTTGTAAAGA
AGATGATATTGAAAATATACCACAATTCTTCAGATGGTTTAGTGAATGGGGTGATGATTA
TTGCCAGGATAAAACAAAAATG
>Equatorial Guinea (Bata) 2019T19036F_MW691434.1

TATGTATGTATTCCTGATCGTAGAATCCAATTATGCATTGTTAATCTTAGCATTATTAAAA
CATATACAAAAGAGACCATGAAGGATCATTTCATTGAAGCCTCTAAAAAAGAATCTCAA
CTTTTGCTTAAAAAAAAATGATAACAAATATAATTCTAAATTTTGTAATGATTTGAAGAATA
GTTTTTTAGATTATGGACATCTTGCTATGGGAAATGATATGGATTTTGGAGGTTATTCAAC
TAAGGCAGAAAACAAAATTCAAGAAGTTTTTAAAGGGGCTCATGGGAAAATAAGTGAAC
ATGAAATTAAAAATTTTAGAAAAGAATGGTGGAATGAATTTAGAGAGAAACTTTGGGAA
GCTATGTTATCTGAGCATAAAAATAATATAAATAATTGTAAAAATATTCCCCAAGAAGAA
TTACAAATTACTCAATGGATAAAAGAATGGCATGGAGAATTTTTGCTTGAAAGAGATAAT
AGATCAAAATTGCCAAAAAGTAAATGTAAAAATAATACATTATATGAAGCATGTGAGAA
GGAATGTATTGATCCATGTATGAAATATAGAGATTGGATTATTAGAAGTAAATTTGAATG
GCATACGTTATCGAAAGAATATGAAACTCAAAAAGTTCCAAAGGAAAATGCGGAAAATT
ATTTAATCAAAATTTCAGAAAACAAGAATGATGCTAAAGTAAGTTTATTATTGAATAATT
GTGATGCTGAATATTCAAAATATTGTGATTGTAAACATACTACTACTCTCGTTAAAAGCGT
TTTAAATGGTAACGACAATACAATTAAGGAAAAGCGTGAACATATTGATTTAGATGATTT
TTCTAAATTTGGATGTGATAAAAATTCCGTTGATACAAACACAAAGGTGTGGGAATGTAA
AAAACCTTATAAATTATCCACTAAAGATGTATGTGTACCTCCGAGGAGGCAAGAATTATG
TCTTGGAAACATTGATAGAATATACGATAAAAACCTATTAATGATAAAAGAGCATATTCT
TGCTATTGCAATATATGAATCAAGAATATTGAAACGAAAATATAAGAATAAAGATGATAA
AGAAGTTTGTAAAATCATAAATAAAACTTTCGCTGATATAAGAGATATTATAGGAGGTAC
TGATTATTGGAATGATTTGAGCAATAGAAAATTAGTAGGAAAAATTAACACAAATTCAAA
TTATGTTCACAGGAATAAACAAAATGATAAGCTTTTTCGTGATGAGTGGTGGAAAGTTAT
TAAAAAAGATGTATGGAATGTGATATCATGGGTATTCAAGGATAAAACTGTTTGTAAAGA
AGATGATATTGAAAATATACCACAATTCTTCAGATGGTTTAGTGAATGGGGTGATGATTA
TTGCCAGGATAAAACAAAAATG
>Equatorial Guinea (Bata) 2019T19027F_MW691435.1
TATGTATGTATTCCTGATCGTAGAATCCAATTATGCATTGTTAATCTTAGCATTATTAAAA
CATATACAAAAGAGACCATGAAGGATCATTTCATTGAAACCTCTAAAAAAGAATCTCAA
CTTTTGCTTAAAAAAAAATGATAACAAATATAATTCTAAATTTTGTAATGATTTGAAGAATA
GTTTTTTAGATTATGGACATCTTGCTATGGGAAATGATATGGATTTTGGAGGTTATTCAAC
TAAGGCAGAAAACAAAATTCAAGAAGTTTTTAAAGGGGCTCATGGGAAAATAAGTGAAC
ATGAAATTAAAAATTTTAGAAAAGAATGGTGGAATGAATTTAGAGAGAAACTTTGGGAA
GCTATGTTATCTGAGCATAAAAATAATATAAATAATTGTAAAAATATTCCCCAAGAAGAA
TTACAAATTACTCAATGGATAAAAGAATGGCATGGAGAATTTTTGCTTGAAAGAGATAAT
AGATCAAAATTGCCAAAAAGTAAATGTAAAAATAATACATTATATGAAGCATGTGAGAA
GGAATGTATTGATCCATGTATGAAATATAGAGATTGGATTATTAGAAGTAAATTTGAATG
GCATACGTTATCGAAAGAATATGAAACTCAAAATGTTTCAAAGGAAAATGCGGAAAATT
ATTTAATCAAAATTTCAGAAAAAATGAATGATGCTAAAGTAAGTTTATTATTGAATAATTG
TGATGCTGAATATTCAAAATATTGTGATTGTAAACATACTACTACTCTCGTTAAAAGCGTT
TTAAATGGTAACGACAATACAATTAAGGAAAAGCGTGAACATATTGATTTAGATGATTTT
TCTAAATTTGGATGTGATAAAAATTCCGTTGATACAAACACAAAGGTGTGGGAATGTAAA
AAACCTTATAAATTATCCACTAAAGATGTATGTGTACCTCCGAGGAGGCAAGAATTATGT
CTTGGAAACATTGATAGAATATACGATAAAAACCTATTAATGATAAAAGAGCATATTCTT
GCTATTGCAATATATGAATCAAGAATATTGAAACGAAAATATAAGAATAAAGATGATAA
AGAAGTTTGTAAAATCATAAATAAAACTTTCGCTGATATAAGAGATATTATAGGAGGTAC

TGATTATTGGAATGATTTGAGCAATAGAAAATTAGTAGGAAAAATTAACACAAATTCAAA
TTATGTTCACAGGAATAAACAAAATGATAAGCTTTTTCGTGATGAGTGGTGGAAAGTTAT
TAAAAAAGATGTATGGAATGTGATATCATGGGTATTCAAGGATAAAACTGTTTGTAAAGA
AGATGATATTGAAAATATACCACAATTCTTCAGATGGTTTAGTGAATGGGGTGATGATTA
TTGCCAGGATAAAACAAAAATG
>Equatorial Guinea (Bata) 2019T19026F_MW691436.1
TATGTATGTATTCCTGATCGTAGAATCCAATTATGCATTGTTAATCTTAGCATTATTAAAA
CATATACAAAAGAGACCATGAAGGATCATTTCATTGAAGCCTCTAAAAAAGAATCTCAA
CTTTTGCTTAAAAAAAAATGATAACAAATATAATTCTAAATTTTGTAATGATTTGAAGAATA
GTTTTTTAGATTATGGACATCTTGCTATGGGAAATGATATGGATTTTGGAGGTTATTCAAC
TAAGGCAGAAAACAAAATTCAAGAAGTTTTTAAAGGGGCTCATGGGAAAATAAGTGAAC
ATGAAATTAAAAATTTTAGAAAAAAATGGTGGAATGAATTTAGAGAGAAACTTTGGGAA
GCTATGTTATCTGAGCATAAAAATAATATAAATAATTGTAAAAATATTCCCCAAGAAGAA
TTACAAATTACTCAATGGATAAAAGAATGGCATGGAGAATTTTTGCTTGAAAGAGATAAT
AGATCAAAATTGCCAAAAAGTAAATGTAAAAATAATACATTATATGAAGCATGTGAGAA
GGAATGTATTGATCCATGTATGAAATATAGAGATTGGATTATTAGAAGTAAATTTGAATG
GCATACGTTATCGAAAGAATATGAAACTCAAAAAGTTCCAAAGGAAAATGCGGAAAATT
ATTTAATCAAAATTTCAGAAAACAAGAATGATGCTAAAGTAAGTTTATTATTGAATAATT
GTGATGCTGAATATTCAAAATATTGTGATTGTAAACATACTACTACTCTCGTTAAAAGCGT
TTTAAATGGTAACGACAATACAATTAAGGAAAAGCGTGAACATATTGATTTAGATGATTT
TTCTAAATTTGGATGTGATAAAAATTCCGTTGATACAAACACAAAGGTGTGGGAATGTAA
AAAACCTTATAAATTATCCACTAAAGATGTATGTGTACCTCCGAGGAGGCAAGAATTATG
TCTTGGAAACATTGATAGAATATACGATAAAAACCTATTAATGATAAAAGAGCATATTCT
TGCTATTGCAATATATGAATCAAGAATATTGAAACGAAAATATAAGAATAAAGATGATAA
AGAAGTTTGTAAAATCATAAATAAAACTTTCGCTGATATAAGAGATATTATAGGAGGTAC
TGATTATTGGAATGATTTGAGCAATAGAAAATTAGTAGGAAAAATTAACACAAATTCAAA
TTATGTTCACAGGAATAAACAAAATGATAAGCTTTTTCGTGATGAGTGGTGGAAAGTTAT
TAAAAAAGATGTATGGAATGTGATATCATGGGTATTCAAGGATAAAACTGTTTGTAAAGA
AGATGATATTGAAAATATACCACAATTCTTCAGATGGTTTAGTGAATGGGGTGATGATTA
TTGCCAGGATAAAACAAAAATG
>Equatorial Guinea (Bata) 2019T19009F_MW691437.1
TATGTATGTATTCCTGATCGTAGAATCCAATTATGCATTGTTAATCTTAGCATTATTAAAA
CATATACAAAAGAGACCATGAAGGATCATTTCATTGAAGCCTCTAAAAAAGAATCTCAA
CTTTTGCTTAAAAAAAAATGATAACAAATATAATTCTAAATTTTGTAATGATTTGAAGAATA
GTTTTTTAGATTATGGACATCTTGCTATGGGAAATGATATGGATTTTGGAGGTTATTCAAC
TAAGGCAGAAAACAAAATTCAAGAAGTTTTTAAAGGGGCTCATGGGAAAATAAGTGAAC
ATGAAATTAAAAATTTTAGAAAAAAATGGTGGAATGAATTTAGAGAGAAACTTTGGGAA
GCTATGTTATCTGAGCATAAAAATAATATAAATAATTGTAAAAATATTCCCCAAGAAGAA
TTACAAATTACTCAATGGATAAAAGAATGGCATGGAGAATTTTTGCTTGAAAGAGATAAT
AGATCAAAATTGCCAAAAAGTAAATGTAAAAATAATACATTATATGAAGCATGTGAGAA
GGAATGTATTGATCCATGTATGAAATATAGAGATTGGATTATTAGAAGTAAATTTGAATG
GCATACGTTATCGAAAGAATATGAAACTCAAAAAGTTTCAAAGGAAAATGCGGAAAATT
ATTTAATCAAAATTTCAGAAAACAAGAATGATGCTAAAGTAAGTTTATTATTGAATAATT
GTGATGCTGAATATTCAAAATATTGTGATTGTAAACATACTACTACTCTCGTTAAAAGCGT

TTTAAATGGTAACGACAATACAATTAAGGAAAAGCGTGAACATATTGATTTAGATGATTT
TTCTAAATTTGGATGTGATAAAAATTCCGTTGATACAAACACAAAGGTGTGGGAATGTAA
AAAACCTTATATATTATCCACTAAAGATGTATGTGTACCTCCGAGGAGGCAAGAATTATG
TCTTGGAAACATTGATAGAATATACAATAAAAACCTATTAATGATAAAAGAGCATATTCT
TGCTATTGCAATATATGAATCAAGAATATTGAAACGAAAATATAAGAATAAAGATGATAA
AGAAGTTTGTAAAATCATAAATAAAACTTTCGCTGATATAAGAGATATTATAGGAGGTAC
TGATTATTGGAATGATTTGAGCAATAGAAAATTAGTAGGAAAAATTAACACAAATTCAAA
TTATGTTCACAGGAATAAAAAAAAATGATAAGCTTTTTCGTGATGAGTGGTGGAAAGTTAT
TAAAAAAGATGTATGGAATGTGATATCATGGGTATTCAAGGATAAAACTGTTTGTAAAGA
AGATGATATTGAAAATATACCACAATTCTTCAGATGGTTTAGTGAATGGGGTGATGATTA
TTGCCAGGATAAAACAAAAATG
>Equatorial Guinea (Bioko) 2019T19075F_MW691438.1
TATGTATGTATTCCTGATCGTAGAATCCAATTATGCATTGTTAATCTTAGCATTATTAAAA
CATATACAAAAGAGACCATGAAGGATCATTTCATTGAAGCCTCTAAAAAAGAATCTCAA
CTTTTGCTTAAAAAAAAATGATAACAAATATAATTCTAAATTTTGTAATGATTTGAAGAATA
GTTTTTTAGATTATGGACATCTTGCTATGGGAAATGATATGGATTTTGGAGGTTATTCAAC
TAAGGCAGAAAACAAAATTCAAGAAGTTTTTAAAGGGGCTCATGGGAAAATAAGTGAAC
ATGAAATTAAAAATTTTAGAAAAAAATGGTGGAATGAATTTAGAGAGAAACTTTGGGAA
GCTATGTTATCTGAGCATAAAAATAATATAAATAATTGTAAAAATATTCCCCAAGAAGAA
TTACAAATTACTCAATGGATAAAAGAATGGCATGGAGAATTTTTGCTTGAAAGAGATAAT
AGATCAAAATTGCCAAAAAGTAAATGTAAAAATAATACATTATATGAAGCATGTGAGAA
GGAATGTATTGATCCATGTATGAAATATAGAGATTGGATTATTAGAAGTAAATTTGAATG
GCATACGTTATCGAAAGAATATGAAACTCAAAAAGTTCCAAAGGAAAATGCGGAAAATT
ATTTAATCAAAATTTCAAAAAACAAGAATGATGCTAAAGTAAGTTTATTATTGAATAATTG
TGATGCTGAATATTCAAAATATTGTGATTGTAAACATACTACTACTCTCGTTAAAAGCGTT
TTAAATGGTAACGACAATACAATTAAGGAAAAGCGTGAACATATTGATTTAGATGATTTT
TCTAAATTTGGATGTGATAAAAATTCCGTTGATACAAACACAAAGGTGTGGGAATGTAAA
AAACCTTATAAATTATCCACTAAAGATGTATGTGTACCTCCGAGGAGGCAAGAATTATGT
CTTGGAAACATTGATAGAATATACGATAAAAACCTATTAATGATAAAAGAGCATATTCTT
GCTATTGCAATATATGAATCAAGAATATTGAAACGAAAATATAAGAATAAAGATGATAA
AGAAGTTTGTAAAATCATAAATAAAACTTTCGCTGATATAAGAGATATTATAGGAGGTAC
TGATTATTGGAATGATTTGAGCAATAGAAAATTAGTAGGAAAAATTAACACAAATTCAAA
TTATGTTCACAGGAATAAACAAAATGATAAGCTTTTTCGTGATGAGTGGTGGAAAGTTAT
TAAAAAAGATGTATGGAATGTGATATCATGGGTATTCAAGGATAAAACTGTTTGTAAAGA
AGATGATATTGAAAATATACCACAATTCTTCAGATGGTTTAGTGAATGGGGTGATGATTA
TTGCCAGGATAAAACAAAAATG
>Equatorial Guinea (Bioko) 2019T19070F_MW691439.1
TATGTATGTATTCCTGATCGTAGAATCCAATTATGCATTGTTAATCTTAGCATTATTAAAA
CATATACAAAAGAGACCATGAAGGATCATTTCATTGAAGCCTCTAAAAAAGAATCTCAA
CTTTTGCTTAAAAAAAAATGATAACAAATATAATTCTAAATTTTGTAATGATTTGAAGAATA
GTTTTTTAGATTATGGACATCTTGCTATGGGAAATGATATGGATTTTGGAGGTTATTCAAC
TAAGGCAGAAAACAAAATTCAAGAAGTTTTTAAAGGGGCTCATGGGGAAATAAGTGAAC
ATAAAATTAAAAATTTTAGAAAAGAATGGTGGAATGAATTTAGAGAGAAACTTTGGGAA
GCTATGTTATCTGAGCATAAAAATAATATAAATAATTGTAAAAATATTCCCCAAGAAGAA

TTACAAATTACTCAATGGATAAAAGAATGGCATGGAGAATTTTTGCTTGAAAGAGATAAT
AGATCAAAATTGCCAAAAAGTAAATGTAAAAATAATACATTATATGAAGCATGTGAGAA
GGAATGTATTGATCCATGTATGAAATATAGAGATTGGATTATTAGAAGTAAATTTGAATG
GCATACGTTATCGAAAGAATATGAAACTCAAAATGTTTCAAAGGAAAATGCGGAAAATT
ATTTAATCAAAATTTCAGAAAAAATGAATGATGCTAAAGTAAGTTTATTATTGAATAATTG
TGATGCTGAATATTCAAAATATTGTGATTGTAAACATACTACTACTCTCGTTAAAAGCGTT
TTAAATGGTAACGACAATACAATTAAGGAAAAGCGTGAACATATTGATTTAGATGATTTT
TCTAAATTTGGATGTGATAAAAATTCCGTTGATACAAACACAAAGGTGTGGGAATGTAAA
AAACCTTATAAAGTATCCACTAAAGATGTATGTGTACCTCCGAGGAGGCAAGAATTATGT
CTTGGAAACATTGATAGAATATACGATAAAAACCTATTAATGATAAAAGAGCATATTCTT
GCTATTGCAATATATGAATCAAGAATATTGAAACGAAAATATAAGAATAAAGATGATAA
AGAAGTTTGTAAAATCATAAATAAAACTTTCGCTGATATAAGAGATATTATAGGAGGTAC
TGATTATTGGAATGATTTGAGCAATAGAAAATTAGTAGGAAAAATTAACACAAATTCAAA
TTATGTTCACAGGAATAAAGAAAATGATAAGCTTTTTCGTGATGCGTGGTGGAAAGTTAT
TAAAAAAGATGTATGGAATGTGATATCATGGGTATTCAAGGATAAAACTGTTTGTAAAGA
AGATGATATTGAAAATATACCACAATTCTTCAGATGGTTTAGTGAATGGGGTGATGATTA
TTGCCAGGATAAAACAAAAATG
>Equatorial Guinea (Bioko) 2019T19058F_MW691440.1
TATGTATGTATTCCTGATCGTAGAATCCAATTATGCATTGTTAATCTTAGCATTATTAAAA
CATATACAAAAGAGACCATGAAGGATCATTTCATTGAAGCCTCTAAAAAAGAATCTCAA
CTTTTGCTTAAAAAAAAATGATAACAAATATAATTCTAAATTTTGTAATGATTTGAAGAATA
GTTTTTTAGATTATGGACATCTTGCTATGGGAAATGATATGGATTTTGGAGGTTATTCAAC
TAAGGCAGAAAACAAAATTCAAGAAGTTTTTAAAGGGGCTCATGGGAAAATAAGTGAAC
ATGAAATTAAAAATTTTAGAAAAAAAATGGTGGAATGAATTTAGAGAGAAACTTTGGGAA
GCTATGTTATCTGAGCATAAAAATAATATAAATAATTGTAAAAATATTCCCCAAGAAGAA
TTACAAATTACTCAATGGATAAAAGAATGGCATGGAGAATTTTTGCTTGAAAGAGATAAT
AGATCAAAATTGCCAAAAAGTAAATGTAAAAATAATACATTATATGAAGCATGTGAGAA
GGAATGTATTGATCCATGTATGAAATATAGAGATTGGATTATTAGAAGTAAATTTGAATG
GCATACGTTATCGAAAGAATATGAAACTCAAAAAGTTCCAAAGGAAAATGCGGAAAATT
ATTTAATCAAAATTTCAAAAAACAAGAATGATGCTAAAGTAAGTTTATTATTGAATAATTG
TGATGCTGAATATTCAAAATATTGTGATTGTAAACATACTACTACTCTCGTTAAAAGCGTT
TTAAATGGTAACGACAATACAATTAAGGAAAAGCGTGAACATATTGATTTAGATGATTTT
TCTAAATTTGGATGTGATAAAAATTCCGTTGATACAAACACAAAGGTGTGGGAATGTAAA
AAACCTTATAAATTATCCACTAAAGATGTATGTGTACCTCCGAGGAGGCAAGAATTATGT
CTTGGAAACATTGATAGAATATACGATAAAAACCTATTAATGATAAAAGAGCATATTCTT
GCTATTGCAATATATGAATCAAGAATATTGAAACGAAAATATAAGAATAAAGATGATAA
AGAAGTTTGTAAAATCATAAATAAAACTTTCGCTGATATAAGAGATATTATAGGAGGTAC
TGATTATTGGAATGATTTGAGCAATAGAAAATTAGTAGGAAAAATTAACACAAATTCAAA
TTATGTTCACAGGAATAAACAAAATGATAAGCTTTTTCGTGATGAGTGGTGGAAAGTTAT
TAAAAAAGATGTATGGAATGTGATATCATGGGTATTCAAGGATAAAACTGTTTGTAAAGA
AGATGATATTGAAAATATACCACAATTCTTCAGATGGTTTAGTGAATGGGGTGATGATTA
TTGCCAGGATAAAACAAAAATG
>Equatorial Guinea (Bioko) 2019B19057F_MW691441.1
TATGTATGTATTCCTGATCGTAGAATCCAATTATGCATTGTTAATCTTAGCATTATTAAAA

CATATACAAAAGAGACCATGAAGGATCATTTCATTGAAGCCTCTAAAAAAGAATCTCAA
CTTTTGCTTAAAAAAAAATGATAACAAATATAATTCTAAATTTTGTAATGATTTGAAGAATA
GTTTTTTAGATTATGGACATCTTGCTATGGGAAATGATATGGATTTTGGAGGTTATTCAAC
TAAGGCAGAAAACAAAATTCAAGAAGTTTTTAAAGGGGCTCATGGGGAAATAAGTGAAC
ATAAAATTAAAAATTTTAGAAAAAAATGGTGGAATGAATTTAGAGAGAAACTTTGGGAA
GCTATGTTATCTGAGCATAAAAATAATATAAATAATTGTAAAAATATTCCCCAAGAAGAA
TTACAAATTACTCAATGGATAAAAGAATGGCATGGAGAATTTTTGCTTGAAAGAGATAAT
AGATCAAAATTGCCAAAAAGTAAATGTAAAAATAATACATTATATGAAGCATGTGAGAA
GGAATGTATTGATCCATGTATGAAATATAGAGATTGGATTATTAGAAGTAAATTTGAATG
GCATACGTTATCGAAAGAATATGAAACTCAAAAAGTTTCAAAGGAAAATGCGGAAAATT
ATTTAATCAAAATTTCAGAAAACAAGAATGATGCTAAAGTAAGTTTATTATTGAATAATT
GTGATGCTGAATATTCAAAATATTGTGATTGTAAACATACTACTACTCTCGTTAAAAGCGT
TTTAAATGGTAACGACAATACAATTAAGGAAAAGCGTGAACATATTGATTTAGATGATTT
TTCTAAATTTGGATGTGATAAAAATTCCGTTGATACAAACACAAAGGTGTGGGAATGTAA
AAAACCTTATATATTATCCACTAAAGATGTATGTGTACCTCCGAGGAGGCAAGAATTATG
TCTTGGAAACATTGATAGAATATACAATAAAAACCTATTAATGATAAAAGAGCATATTCT
TGCTATTGCAATATATGAATCAAGAATATTGAAACGAAAATATAAGAATAAAGATGATAA
AGAAGTTTGTAAAATCATAAATAAAACTTTCGCTGATATAAGAGATATTATAGGAGGTAC
TGATTATTGGAATGATTTGAGCAATAGAAAATTAGTAGGAAAAATTAACACAAATTCAAA
TTATGTTCACAGGAATAAAAAAAAATGATAAGCTTTTTCGTGATGAGTGGTGGAAAGTTAT
TAAAAAAGATGTATGGAATGTGATATCATGGGTATTCAAGGATAAAACTGTTTGTAAAGA
AGATGATATTGAAAATATACCACAATTCTTCAGATGGTTTAGTGAATGGGGTGATGATTA
TTGCCAGGATAAAACAAAAATG
>Equatorial Guinea (Bioko) 2019B19055F_MW691442.1
TATGTATGTATTCCTGATCGTAGAATCCAATTATGCATTGTTAATCTTAGCATTATTAAAA
CATATACAAAAGAGACCATGAAGGATCATTTCATTGAAGCCTCTAAAAAAGAATCTCAA
CTTTTGCTTAAAAAAAAATGATAACAAATATAATTCTAAATTTTGTAATGATTTGAAGAATA
GTTTTTTAGATTATGGACATCTTGCTATGGGAAATGATATGGATTTTGGAGGTTATTCAAC
TAAGGCAGAAAACAAAATTCAAGAAGTTTTTAAAGGGGCTCATGGGAAAATAAGTGAAC
ATGAAATTAAAAATTTTAGAAAAAAATGGTGGAATGAATTTAGAGAGAAACTTTGGGAA
GCTATGTTATCTGAGCATAAAAATAATATAAATAATTGTAAAAATATTCCCCAAGAAGAA
TTACAAATTACTCAATGGATAAAAGAATGGCATGGAGAATTTTTGCTTGAAAGAGATAAT
AGATCAAAATTGCCAAAAAGTAAATGTAAAAATAATACATTATATGAAGCATGTGAGAA
GGAATGTATTGATCCATGTATGAAATATAGAGATTGGATTATTAGAAGTAAATTTGAATG
GCATACGTTATCGAAAGAATATGAAACTCAAAAAGTTCCAAAGGAAAATGCGGAAAATT
ATTTAATCAAAATTTCAAAAAACAAGAATGATGCTAAAGTAAGTTTATTATTGAATAATTG
TGATGCTGAATATTCAAAATATTGTGATTGTAAACATACTACTACTCTCGTTAAAAGCGTT
TTAAATGGTAACGACAATACAATTAAGGAAAAGCGTGAACATATTGATTTAGATGATTTT
TCTAAATTTGGATGTGATAAAAATTCCGTTGATACAAACACAAAGGTGTGGGAATGTAAA
AAACCTTATAAATTATCCACTAAAGATGTATGTGTACCTCCGAGGAGGCAAGAATTATGT
CTTGGAAACATTGATAGAATATACGATAAAAACCTATTAATGATAAAAGAGCATATTCTT
GCTATTGCAATATATGAATCAAGAATATTGAAACGAAAATATAAGAATAAAGATGATAA
AGAAGTTTGTAAAATCATAAATAAAACTTTCGCTGATATAAGAGATATTATAGGAGGTAC
TGATTATTGGAATGATTTGAGCAATAGAAAATTAGTAGGAAAAATTAACACAAATTCAAA

TTATGTTCACAGGAATAAACAAAATGATAAGCTTTTTCGTGATGAGTGGTGGAAAGTTAT
TAAAAAAGATGTATGGAATGTGATATCATGGGTATTCAAGGATAAAACTGTTTGTAAAGA
AGATGATATTGAAAATATACCACAATTCTTCAGATGGTTTAGTGAATGGGGTGATGATTA
TTGCCAGGATAAAACAAAAATG
>Equatorial Guinea (Bioko) 2019B19054F_MW691443.1
TATGTATGTATTCCTGATCGTAGAATCCAATTATGCATTGTTAATCTTAGCATTATTAAAA
CATATACAAAAGAGACCATGAAGGATCATTTCATTGAAGCCTCTAAAAAAGAATCTCAA
CTTTTGCTTAAAAAAAAATGATAACAAATATAATTCTAAATTTTGTAATGATTTGAAGAATA
GTTTTTTAGATTATGGACATCTTGCTATGGGAAATGATATGGATTTTGGAGGTTATTCAAC
TAAGGCAGAAAACAAAATTCAAGAAGTTTTTAAAGGGGCTCATGGGAAAATAAGTGAAC
ATGAAATTAAAAATTTTAGAAAAGAATGGTGGAATGAATTTAGAGAGAAACTTTGGGAA
GCTATGTTATCTGAGCATAAAAATAATATAAATAATTGTAAAAATATTCCCCAAGAAGAA
TTACAAATTACTCAATGGATAAAAGAATGGCATGGAGAATTTTTGCTTGAAAGAGATAAT
AGATCAAAATTGCCAAAAAGTAAATGTAAAAATAATACATTATATGAAGCATGTGAGAA
GGAATGTATTGATCCATGTATGAAATATAGAGATTGGATTATTAGAAGTAAATTTGAATG
GCATACGTTATCGAAAGAATATGAAACTCAAAATGTTTCAAAGGAAAATGCGGAAAATT
ATTTAATCAAAATTTCAAAAAACAAGAATGATGCTAAAGTAAGTTTATTATTGAATAATTG
TGATGCTGAATATTCAAAATATTGTGATTGTAAACATACTACTACTCTCGTTAAAAGCGTT
TTAAATGGTAACGACAATACAATTAAGGAAAAGCGTGAACATATTGATTTAGATGATTTT
TCTAAATTTGGATGTGATAAAAATTCCGTTGATACAAACACAAAGGTGTGGGAATGTAAA
AAACCTTATAAATTATCCACTAAAGATGTATGTGTACCTCCGAGGAGGCAAGAATTATGT
CTTGGAAACATTGATAGAATATACGATAAAAACCTATTAATGATAAAAGAGCATATTCTT
GCTATTGCAATATATGAATCAAGAATATTGAAACGAAAATATAAGAATAAAGATGATAA
AGAAGTTTGTAAAATCATAAATAAAACTTTCGCTGATATAAGAGATATTATAGGAGGTAC
TGATTATTGGAATGATTTGAGCAATAGAAAATTAGTAGGAAAAATTAACACAAATTCAAA
TTATGTTCACAGGAATAAACAAAATGATAAGCTTTTTCGTGATGAGTGGTGGAAAGTTAT
TAAAAAAGATGTATGGAATGTGATATCATGGGTATTCAAGGATAAAACTGTTTGTAAAGA
AGATGATATTGAAAATATACCACAATTCTTCAGATGGTTTAGTGAATGGGGTGATGATTA
TTGCCAGGATAAAACAAAAATG
>Equatorial Guinea (Bioko) 2019B19052F_MW691444.1
TATGTATGTATTCCTGATCGTAGAATCCAATTATGCATTGTTAATCTTAGCATTATTAAAA
CATATACAAAAGAGACCATGAAGGATCATTTCATTGAAGCCTCTAAAAAAGAATCTCAA
CTTTTGCTTAAAAAAAAATGATAACAAATATAATTCTAAATTTTGTAATGATTTGAAGAATA
GTTTTTTAGATTATGGACATCTTGCTATGGGAAATGATATGGATTTTGGAGGTTATTCAAC
TAAGGCAGAAAACAAAATTCAAGAAGTTTTTAAAGGGGCTCATGGGAAAATAAGTGAAC
ATGAAATTAAAAATTTTAGAAAAAAATGGTGGAATGAATTTAGAGAGAAACTTTGGGAA
GCTATGTTATCTGAGCATAAAAATAATATAAATAATTGTAAAAATATTCCCCAAGAAGAA
TTACAAATTACTCAATGGATAAAAGAATGGCATGGAGAATTTTTGCTTGAAAGATATAAT
AGATCAAAATTGCCAAAAAGTAAATGTAAAAATAATACATTATATGAAGCATGTGAGAA
GGAATGTATTGATCCATGTATGAAATATAGAGATTGGATTATTAGAAGTAAATTTGAATG
GCATACGTTATCGAAAGAATATGAAACTCAAAATGTTTCAAAGGAAAATGCGGAAAATT
ATTTAATCAAAATTTCAAAAAACAAGAATGATGCTAAAGTAAGTTTATTATTGAATAATTG
TGATGCTGAATATTCAAAATATTGTGATTGTAAACATACTACTACTCTCGTTAAAAGCGTT
TTAAATGGTAACGACAATACAATTAAGGAAAAGCGTGAACATATTGATTTAGATGATTTT

TCTAAATTTGGATGTGATAAAAATTCCGTTGATACAAACACAAAGGTGTGGGAATGTAAA
AAACCTTATAAATTATCCACTAAAGATGTATGTGTACCTCCGAGGAGGCAAGAATTATGT
CTTGGAAACATTGATAGAATATACGATAAAAACCTATTAATGATAAAAGAGCATATTCTT
GCTATTGCAATATATGAATCAAGAATATTGAAACGAAAATATAAGAATAAAGATGATAA
AGAAGTTTGTAAAATCATAAATAAAACTTTCGCTGATATAAGAGATATTATAGGAGGTAC
TGATTATTGGAATGATTTGAGCAATAGAAAATTAGTAGGAAAAATTAACACAAATTCAAA
TTATGTTCACAGGAATAAACAAAATGATAAGCTTTTTCGTGATGAGTGGTGGAAAGTTAT
TAAAAAAGATGTATGGAATGTGATATCATGGGTATTCAAGGATAAAACTGTTTGTAAAGA
AGATGATATTGAAAATATACCACAATTCTTCAGATGGTTTAGTGAATGGGGTGATGATTA
TTGCCAGGATAAAACAAAAATG
>Equatorial Guinea (Bioko) 2019B19051F_MW691445.1
TATGTATGTATTCCTGATCGTAGAATCCAATTATGCATTGTTAATCTTAGCATTATTAAAA
CATATACAAAAGAGACCATGAAGGATCATTTCATTGAAGCCTCTAAAAAAGAATCTCAA
CTTTTGCTTAAAAAAAAATGATAACAAATATAATTCTAAATTTTGTAATGATTTGAAGAATA
GTTTTTTAGATTATGGACATCTTGCTATGGGAAATGATATGGATTTTGGAGGTTATTCAAC
TAAGGCAGAAAACAAAATTCAAGAAGTTTTTAAAGGGGCTCATGGGAAAATAAGTGAAC
ATGAAATTAAAAATTTTAGAAAAAAATGGTGGAATGAATTTAGAGAGAAACTTTGGGAA
GCTATGTTATCTGAGCATAAAAATAATATAAATAATTGTAAAAATATTCCCCAAGAAGAA
TTACAAATTACTCAATGGATAAAAGAATGGCATGGAGAATTTTTGCTTGAAAGATATAAT
AGATCAAAATTGCCAAAAAGTAAATGTAAAAATAATACATTATATGAAGCATGTGAGAA
GGAATGTATTGATCCATGTATGAAATATAGAGATTGGATTATTAGAAGTAAATTTGAATG
GCATACGTTATCGAAAGAATATGAAACTCAAAATGTTTCAAAGGAAAATGCGGAAAATT
ATTTAATCAAAATTTCAAAAAACAAGAATGATGCTAAAGTAAGTTTATTATTGAATAATTG
TGATGCTGAATATTCAAAATATTGTGATTGTAAACATACTACTACTCTCGTTAAAAGCGTT
TTAAATGGTAACGACAATACAATTAAGGAAAAGCGTGAACATATTGATTTAGATGATTTT
TCTAAATTTGGATGTGATAAAAATTCCGTTGATACAAACACAAAGGTGTGGGAATGTAAA
AAACCTTATAAATTATCCACTAAAGATGTATGTGTACCTCCGAGGAGGCAAGAATTATGT
CTTGGAAACATTGATAGAATATACGATAAAAACCTATTAATGATAAAAGAGCATATTCTT
GCTATTGCAATATATGAATCAAGAATATTGAAACGAAAATATAAGAATAAAGATGATAA
AGAAGTTTGTAAAATCATAAATAAAACTTTCGCTGATATAAGAGATATTATAGGAGGTAC
TGATTATTGGAATGATTTGAGCAATAGAAAATTAGTAGGAAAAATTAACACAAATTCAAA
TTATGTTCACAGGAATAAACAAAATGATAAGCTTTTTCGTGATGAGTGGTGGAAAGTTAT
TAAAAAAGATGTATGGAATGTGATATCATGGGTATTCAAGGATAAAACTGTTTGTAAAGA
AGATGATATTGAAAATATACCACAATTCTTCAGATGGTTTAGTGAATGGGGTGATGATTA
TTGCCAGGATAAAACAAAAATG
>Equatorial Guinea (Bioko) 2019B19045F_MW691446.1
TATGTATGTATTCCTGATCGTAGAATCCAATTATGCATTGTTAATCTTAGCATTATTAAAA
CATATACAAAAGAGACCATGAAGGATCATTTCATTGAAGCCTCTAAAAAAGAATCTCAA
CTTTTGCTTAAAAAAAAATGATAACAAATATAATTCTAAATTTTGTAATGATTTGAAGAATA
GTTTTTTAGATTATGGACATCTTGCTATGGGAAATGATATGGATTTTGGAGGTTATTCAAC
TAAGGCAGAAAACAAAATTCAAGAAGTTTTTAAAGGGGCTCATGGGAAAATAAGTGAAC
ATGAAATTAAAAATTTTAGAAAAAAATGGTGGAATGAATTTAGAGAGAAACTTTGGGAA
GCTATGTTATCTGAGCATAAAAATAATATAAATAATTGTAAAAATATTCCCCAAGAAGAA
TTACAAATTACTCAATGGATAAAAGAATGGCATGGAGAATTTTTGCTTGAAAGATATAAT

AGATCAAAATTGCCAAAAAGTAAATGTAAAAATAATACATTATATGAAGCATGTGAGAA
GGAATGTATTGATCCATGTATGAAATATAGAGATTGGATTATTAGAAGTAAATTTGAATG
GCATACGTTATCGAAAGAATATGAAACTCAAAATGTTTCAAAGGAAAATGCGGAAAATT
ATTTAATCAAAATTTCAAAAAACAAGAATGATGCTAAAGTAAGTTTATTATTGAATAATTG
TGATGCTGAATATTCAAAATATTGTGATTGTAAACATACTACTACTCTCGTTAAAAGCGTT
TTAAATGGTAACGACAATACAATTAAGGAAAAGCGTGAACATATTGATTTAGATGATTTT
TCTAAATTTGGATGTGATAAAAATTCCGTTGATACAAACACAAAGGTGTGGGAATGTAAA
AAACCTTATAAATTATCCACTAAAGATGTATGTGTACCTCCGAGGAGGCAAGAATTATGT
CTTGGAAACATTGATAGAATATACGATAAAAACCTATTAATGATAAAAGAGCATATTCTT
GCTATTGCAATATATGAATCAAGAATATTGAAACGAAAATATAAGAATAAAGATGATAA
AGAAGTTTGTAAAATCATAAATAAAACTTTCGCTGATATAAGAGATATTATAGGAGGTAC
TGATTATTGGAATGATTTGAGCAATAGAAAATTAGTAGGAAAAATTAACACAAATTCAAA
TTATGTTCACAGGAATAAACAAAATGATAAGCTTTTTCGTGATGAGTGGTGGAAAGTTAT
TAAAAAAGATGTATGGAATGTGATATCATGGGTATTCAAGGATAAAACTGTTTGTAAAGA
AGATGATATTGAAAATATACCACAATTCTTCAGATGGTTTAGTGAATGGGGTGATGATTA
TTGCCAGGATAAAACAAAAATG
>Equatorial Guinea (Bioko) 2019B19044F_MW691447.1
TATGTATGTATTCCTGATCGTAGAATCCAATTATGCATTGTTAATCTTAGCATTATTAAAA
CATATACAAAAGAGACCATGAAGGATCATTTCATTGAAGCCTCTAAAAAAGAATCTCAA
CTTTTGCTTAAAAAAAATGATAACAAATATAATTCTAAATTTTGTAATGATTTGAAGAATA
GTTTTTTAGATTATGGACATCTTGCTATGGGAAATGATATGGATTTTGGAGGTTATTCAAC
TAAGGCAGAAAACAAAATTCAAGAAGTTTTTAAAGGGGCTCATGGGGAAATAAGTGAAC
ATAAAATTAAAAATTTTAGAAAAGAATGGTGGAATGAATTTAGAGAGAAACTTTGGGAA
GCTATGTTATCTGAGCATAAAAATAATATAAATAATTGTAAAAATATTCCCCAAGAAGAA
TTACAAATTACTCAATGGATAAAAGAATGGCATGGAGAATTTTTGCTTGAAAGAGATAAT
AGATCAAAATTGCCAAAAAGTAAATGTAAAAATAATACATTATATGAAGCATGTGAGAA
GGAATGTATTGATCCATGTATGAAATATAGAGATTGGATTATTAGAAGTAAATTTGAATG
GCATACGTTATCGAAAGAATATGAAACTCAAAAAGTTCCAAAGGAAAATGCGGAAAATT
ATTTAATCAAAATTTCAGAAAACAAGAATGATGCTAAAGTAAGTTTATTATTGAATAATT
GTGATGCTGAATATTCAAAATATTGTGATTGTAAACATACTACTACTCTCGTTAAAAGCGT
TTTAAATGGTAACGACAATACAATTAAGGAAAAGCGTGAACATATTGATTTAGATGATTT
TTCTAAATTTGGATGTGATAAAAATTCCGTTGATACAAACACAAAGGTGTGGGAATGTAA
AAAACCTTATAAATTATCCACTAAAGATGTATGTGTACCTCCGAGGAGGCAAGAATTATG
TCTTGGAAACATTGATAGAATATACGATAAAAACCTATTAATGATAAAAGAGCATATTCT
TGCTATTGCAATATATGAATCAAGAATATTGAAACGAAAATATAAGAATAAAGATGATAA
AGAAGTTTGTAAAATCATAAATAAAACTTTCGCTGATATAAGAGATATTATAGGAGGTAC
TGATTATTGGAATGATTTGAGCAATAGAAAATTAGTAGGAAAAATTAACACAAATTCAAA
TTATGTTCACAGGAATAAACAAAATGATAAGCTTTTTCGTGATGAGTGGTGGAAAGTTAT
TAAAAAAGATGTATGGAATGTGATATCATGGGTATTCAAGGATAAAACTGTTTGTAAAGA
AGATGATATTGAAAATATACCACAATTCTTCAGATGGTTTAGTGAATGGGGTGATGATTA
TTGCCAGGATAAAACAAAAATG
>Equatorial Guinea (Bioko) 2019B19041F_MW691448.1
TATGTATGTATTCCTGATCGTAGAATCCAATTATGCATTGTTAATCTTAGCATTATTAAAA
CATATACAAAAGAGACCATGAAGGATCATTTCATTGAAGCCTCTAAAAAAGAATCTCAA

CTTTTGCTTAAAAAAAAATGATAACAAATATAATTCTAAATTTTGTAATGATTTGAAGAATA
GTTTTTTAGATTATGGACATCTTGCTATGGGAAATGATATGGATTTTGGAGGTTATTCAAC
TAAGGCAGAAAACAAAATTCAAGAAGTTTTTAAAGGGGCTCATGGGAAAATAAGTGAAC
ATGAAATTAAAAATTTTAGAAAAAAATGGTGGAATGAATTTAGAGAGAAACTTTGGGAA
GCTATGTTATCTGAGCATAAAAATAATATAAATAATTGTAAAAATATTCCCCAAGAAGAA
TTACAAATTACTCAATGGATAAAAGAATGGCATGGAGAATTTTTGCTTGAAAGAGATAAT
AGATCAAAATTGCCAAAAAGTAAATGTAAAAATAATACATTATATGAAGCATGTGAGAA
GGAATGTATTGATCCATGTATGAAATATAGAGATTGGATTATTAGAAGTAAATTTGAATG
GCATACGTTATCGAAAGAATATGAAACTCAAAAAGTTTCAAAGGAAAATGCGGAAAATT
ATTTAATCAAAATTTCAGAAAACAAGAATGATGCTAAAGTAAGTTTATTATTGAATAATT
GTGATGCTGAATATTCAAAATATTGTGATTGTAAACATACTACTACTCTCGTTAAAAGCGT
TTTAAATGGTAACGACAATACAATTAAGGAAAAGCGTGAACATATTGATTTAGATGATTT
TTCTAAATTTGGATGTGATAAAAATTCCGTTGATACAAACACAAAGGTGTGGGAATGTAA
AAAACCTTATAAATTATCCACTAAAGATGTATGTGTACCTCCGAGGAGGCAAGAATTATG
TCTTGGAAACATTGATAGAATATACGATAAAAACCTATTAATGATAAAAGAGCATATTCT
TGCTATTGCAATATATGAATCAAGAATATTGAAACGAAAATATAAGAATAAAGATGATAA
AGAAGTTTGTAAAATCATAAATAAAACTTTCGCTGATATAAGAGATATTATAGGAGGTAC
TGATTATTGGAATGATTTGAGCAATAGAAAATTAGTAGGAAAAATTAACACAAATTCAAA
ATATGTTCACAGGAATAAAGAAAATGATAAGCTTTTTCGTGATGAGTGGTGGAAAGTTAT
TAAAAAAGATGTATGGAATGTGATATCATGGGTATTCAAGGATAAAACTGTTTGTAAAGA
AGATGATATTGAAAATATACCACAATTCTTCAGATGGTTTAGTGAATGGGGTGATGATTA
TTGCCAGGATAAAACAAAAATG
>Equatorial Guinea (Bioko) 2019B19040F_MW691449.1
TATGTATGTATTCCTGATCGTAGAATCCAATTATGCATTGTTAATCTTAGCATTATTAAAA
CATATACAAAAGAGACCATGAAGGATCATTTCATTGAAGCCTCTAAAAAAGAATCTCAA
CTTTTGCTTAAAAAAAAATGATAACAAATATAATTCTAAATTTTGTAATGATTTGAAGAATA
GTTTTTTAGATTATGGACATCTTGCTATGGGAAATGATATGGATTTTGGAGGTTATTCAAC
TAAGGCAGAAAACAAAATTCAAGAAGTTTTTAAAGGGGCTCATGGGAAAATAAGTGAAC
ATGAAATTAAAAATTTTAGAAAAAAATGGTGGAATGAATTTAGAGAGAAACTTTGGGAA
GCTATGTTATCTGAGCATAAAAATAATATAAATAATTGTAAAAATATTCCCCAAGAAGAA
TTACAAATTACTCAATGGATAAAAGAATGGCATGGAGAATTTTTGCTTGAAAGAGATAAT
AGATCAAAATTGCCAAAAAGTAAATGTAAAAATAATACATTATATGAAGCATGTGAGAA
GGAATGTATTGATCCATGTATGAAATATAGAGATTGGATTATTAGAAGTAAATTTGAATG
GCATACGTTATCGAAAGAATATGAAACTCAAAAAGTTCCAAAGGAAAATGCGGAAAATT
ATTTAATCAAAATTTCAAAAAACAAGAATGATGCTAAAGTAAGTTTATTATTGAATAATTG
TGATGCTGAATATTCAAAATATTGTGATTGTAAACATACTACTACTCTCGTTAAAAGCGTT
TTAAATGGTAACGACAATACAATTAAGGAAAAGCGTGAACATATTGATTTAGATGATTTT
TCTAAATTTGGATGTGATAAAAATTCCGTTGATACAAACACAAAGGTGTGGGAATGTAAA
AAACCTTATAAATTATCCACTAAAGATGTATGTGTACCTCCGAGGAGGCAAGAATTATGT
CTTGGAAACATTGATAGAATATACGATAAAAACCTATTAATGATAAAAGAGCATATTCTT
GCTATTGCAATATATGAATCAAGAATATTGAAACGAAAATATAAGAATAAAGATGATAA
AGAAGTTTGTAAAATCATAAATAAAACTTTCGCTGATATAAGAGATATTATAGGAGGTAC
TGATTATTGGAATGATTTGAGCAATAGAAAATTAGTAGGAAAAATTAACACAAATTCAAA
TTATGTTCACAGGAATAAACAAAATGATAAGCTTTTTCGTGATGAGTGGTGGAAAGTTAT

TAAAAAAGATGTATGGAATGTGATATCATGGGTATTCAAGGATAAAACTGTTTGTAAAGA
AGATGATATTGAAAATATACCACAATTCTTCAGATGGTTTAGTGAATGGGGTGATGATTA
TTGCCAGGATAAAACAAAAATG
>Equatorial Guinea (Bioko) 2019B19036F_MW691450.1
TATGTATGTATTCCTGATCGTAGAATCCAATTATGCATTGTTAATCTTAGCATTATTAAAA
CATATACAAAAGAGACCATGAAGGATCATTTCATTGAAGCCTCTAAAAAAGAATCTCAA
CTTTTGCTTAAAAAAAAATGATAACAAATATAATTCTAAATTTTGTAATGATTTGAAGAATA
GTTTTTTAGATTATGGACATCTTGCTATGGGAAATGATATGGATTTTGGAGGTTATTCAAC
TAAGGCAGAAAACAAAATTCAAGAAGTTTTTAAAGGGGCTCATGGGAAAATAAGTGAAC
ATGAAATTAAAAATTTTAGAAAAAAATGGTGGAATGAATTTAGAGAGAAACTTTGGGAA
GCTATGTTATCTGAGCATAAAAATAATATAAATAATTGTAAAAATATTCCCCAAGAAGAA
TTACAAATTACTCAATGGATAAAAGAATGGCATGGAGAATTTTTGCTTGAAAGAGATAAT
AGATCAAAATTGCCAAAAAGTAAATGTAAAAATAATACATTATATGAAGCATGTGAGAA
GGAATGTATTGATCCATGTATGAAATATAGAGATTGGATTATTAGAAGTAAATTTGAATG
GCATACGTTATCGAAAGAATATGAAACTCAAAAAGTTCCAAAGGAAAATGCGGAAAATT
ATTTAATCAAAATTTCAGAAAACGAGAATGATGCTAAAGTAAGTTTATTATTGAATAATT
GTGATGCTGAATATTCAAAATATTGTGATTGTAAACATACTACTACTCTCGTTAAAAGCGT
TTTAAATGGTAACGACAATACAATTAAGGAAAAGCGTGAACATATTGATTTATATGATTT
TTCTAAATTTGGATGTGATAAAAATTCCGTTGATACAAACACAAAGGTGTGGGAATGTAA
AAAACCTTATAAATTATCCACTAAAGATGTATGTGTACCTCCGAGGAGGCAAGAATTATG
TCTTGGAAACATTGATAGAATATACGATAAAAACCTATTAATGATAAAAGAGCATATTCT
TGCTATTGCAATATATGAATCAAGAATATTGAAACGAAAATATAAGAATAAAGATGATAA
AGAAGTTTGTAAAATCATAAATAAAACTTTCGCTGATATAAGAGATATTATAGGAGGTAC
TGATTATTGGAATGATTTGAGCAATAGAAAATTAGTAGGAAAAATTAACACAAATTCAAA
TTATGTTCACAGGAATAAACAAAATGATAAGCTTTTTCGTGATGAGTGGTGGAAAGTTAT
TAAAAAAGATGTATGGAATGTGATATCATGGGTATTCAAGGATAAAACTGTTTGTAAAGA
AGATGATATTGAAAATATACCACAATTCTTCAGATGGTTTAGTGAATGGGGTGATGATTA
TTGCCAGGATAAAACAAAAATG
>Equatorial Guinea (Bioko) 2019B19034F_MW691451.1
TATGTATGTATTCCTGATCGTAGAATCCAATTATGCATTGTTAATCTTAGCATTATTAAAA
CATATACAAAAGAGACCATGAAGGATCATTTCATTGAAGCCTCTAAAAAAGAATCTCAA
CTTTTGCTTAAAAAAAAATGATAACAAATATAATTCTAAATTTTGTAATGATTTGAAGAATA
GTTTTTTAGATTATGGACATCTTGCTATGGGAAATGATATGGATTTTGGAGGTTATTCAAC
TAAGGCAGAAAACAAAATTCAAGAAGTTTTTAAAGGGGCTCATGGGAAAATAAGTGAAC
ATGAAATTAAAAATTTTAGAAAAAAATGGTGGAATGAATTTAGAGAGAAACTTTGGGAA
GCTATGTTATCTGAGCATAAAAATAATATAAATAATTGTAAAAATATTCCCCAAGAAGAA
TTACAAATTACTCAATGGATAAAAGAATGGCATGGAGAATTTTTGCTTGAAAGAGATAAT
AGATCAAAATTGCCAAAAAGTAAATGTAAAAATAATACATTATATGAAGCATGTGAGAA
GGAATGTATTGATCCATGTATGAAATATAGAGATTGGATTATTAGAAGTAAATTTGAATG
GCATACGTTATCGAAAGAATATGAAACTCAAAAAGTTCCAAAGGAAAATGCGGAAAATT
ATTTAATCAAAATTTCAAAAAACAAGAATGATGCTAAAGTAAGTTTATTATTGAATAATTG
TGATGCTGAATATTCAAAATATTGTGATTGTAAACATACTACTACTCTCGTTAAAAGCGTT
TTAAATGGTAACGACAATACAATTAAGGAAAAGCGTGAACATATTGATTTAGATGATTTT
TCTAAATTTGGATGTGATAAAAATTCCGTTGATACAAACACAAAGGTGTGGGAATGTAAA

AAACCTTATAAATTATCCACTAAAGATGTATGTGTACCTCCGAGGAGGCAAGAATTATGT
CTTGGAAACATTGATAGAATATACGATAAAAACCTATTAATGATAAAAGAGCATATTCTT
GCTATTGCAATATATGAATCAAGAATATTGAAACGAAAATATAAGAATAAAGATGATAA
AGAAGTTTGTAAAATCATAAATAAAACTTTCGCTGATATAAGAGATATTATAGGAGGTAC
TGATTATTGGAATGATTTGAGCAATAGAAAATTAGTAGGAAAAATTAACACAAATTCAAA
TTATGTTCACAGGAATAAACAAAATGATAAGCTTTTTCGTGATGAGTGGTGGAAAGTTAT
TAAAAAAGATGTATGGAATGTGATATCATGGGTATTCAAGGATAAAACTGTTTGTAAAGA
AGATGATATTGAAAATATACCACAATTCTTCAGATGGTTTAGTGAATGGGGTGATGATTA
TTGCCAGGATAAAACAAAAATG
>Equatorial Guinea (Bioko) 2019B19022F_MW691452.1
TATGTATGTATTCCTGATCGTAGAATCCAATTATGCATTGTTAATCTTAGCATTATTAAAA
CATATACAAAAGAGACCATGAAGGATCATTTCATTGAAGCCTCTAAAAAAGAATCTCAA
CTTTTGCTTAAAAAAAATGATAACAAATATAATTCTAAATTTTGTAATGATTTGAAGAATA
GTTTTTTAGATTATGGACATCTTGCTATGGGAAATGATATGGATTTTGGAGGTTATTCAAC
TAAGGCAGAAAACAAAATTCAAGAAGTTTTTAAAGGGGCTCATGGGAAAATAAGTGAAC
ATAAAATTAAAAATTTTAGAAAAGAATGGTGGAATGAATTTAGAGAGAAACTTTGGGAA
GCTATGTTATCTGAGCATAAAAATAATATAAATAATTGTAAAAATATTCCCCAAGAAGAA
TTACAAATTACTCAATGGATAAAAGAATGGCATGGAGAATTTTTGCTTGAAAGAGATAAT
AGATCAAAATTGCCAAAAAGTAAATGTAAAAATAATACATTATATGAAGCATGTGAGAA
GGAATGTATTGATCCATGTATGAAATATAGAGATTGGATTATTAGAAGTAAATTTGAATG
GCATACGTTATCGAAAGAATATGAAACTCAAAATGTTTCAAAGGAAAATGCGGAAAATT
ATTTAATCAAAATTTCAAAAAACAAGAATGATGCTAAAGTAAGTTTATTATTGAATAATTG
TGATGCTGAATATTCAAAATATTGTGATTGTAAACATACTACTACTCTCGTTAAAAGCGTT
TTAAATGGTAACGACAATACAATTAAGGAAAAGCGTGAACATATTGATTTAGATGATTTT
TCTAAATTTGGATGTGATAAAAATTCCGTTGATACAAACACAAAGGTGTGGGAATGTAAA
AAACCTTATAAATTATCCACTAAAGATGTATGTGTACCTCCGAGGAGGCAAGAATTATGT
CTTGGAAACATTGATAGAATATACGATAAAAACCTATTAATGATAAAAGAGCATATTCTT
GCTATTGCAATATATGAATCAAGAATATTGAAACGAAAATATAAGAATAAAGATGATAA
AGAAGTTTGTAAAATCATAAATAAAACTTTCGCTGATATAAGAGATATTATAGGAGGTAC
TGATTATTGGAATGATTTGAGCAATAGAAAATTAGTAGGAAAAATTAACACAAATTCAAA
TTATGTTCACAGGAATAAACAAAATGATAAGCTTTTTCGTGATGAGTGGTGGAAAGTTAT
TAAAAAAGATGTATGGAATGTGATATCATGGGTATTCAAGGATAAAACTGTTTGTAAAGA
AGATGATATTGAAAATATACCACAATTCTTCAGATGGTTTAGTGAATGGGGTGATGATTA
TTGCCAGGATAAAACAAAAATG
>Equatorial Guinea (Bioko) 2019B19020F_MW691453.1
TATGTATGTATTCCTGATCGTAGAATCCAATTATGCATTGTTAATCTTAGCATTATTAAAA
CATATACAAAAGAGACCATGAAGGATCATTTCATTGAAGCCTCTAAAAAAGAATCTCAA
CTTTTGCTTAAAAAAAATGATAACAAATATAATTCTAAATTTTGTAATGATTTGAAGAATA
GTTTTTTAGATTATGGACATCTTGCTATGGGAAATGATATGGATTTTGGAGGTTATTCAAC
TAAGGCAGAAAACAAAATTCAAGAAGTTTTTAAAGGGGCTCATGGGGAAATAAGTGAAC
ATAAAATTAAAAATTTTAGAAAAAAAATGGTGGAATGAATTTAGAGAGAAACTTTGGGAA
GCTATGTTATCTGAGCATAAAAATAATATAAATAATTGTAAAAATATTCCCCAAGAAGAA
TTACAAATTACTCAATGGATAAAAGAATGGCATGGAGAATTTTTGCTTGAAAGAGATAAT
AGATCAAAATTGCCAAAAAGTAAATGTAAAAATAATACATTATATGAAGCATGTGAGAA

GGAATGTATTGATCCATGTATGAAATATAGAGATTGGATTATTAGAAGTAAATTTGAATG
GCATACGTTATCGAAAGAATATGAAACTCAAAAAGTTCCAAAGGAAAATGCGGAAAATT
ATTTAATCAAAATTTCAGAAAACAAGAATGATGCTAAAGTAAGTTTATTATTGAATAATT
GTGATGCTGAATATTCAAAATATTGTGATTGTAAACATACTACTACTCTCGTTAAAAGCGT
TTTAAATGGTAACGACAATACAATTAAGGAAAAGCGTGAACATATTGATTTAGATGATTT
TTCTAAATTTGGATGTGATAAAAATTCCGTTGATACAAACACAAAGGTGTGGGAATGTAA
AAAACCTTATAAATTATCCACTAAAGATGTATGTGTACCTCCGAGGAGGCAAGAATTATG
TCTTGGAAACATTGATAGAATATACGATAAAAACCTATTAATGATAAAAGAGCATATTCT
TGCTATTGCAATATATGAATCAAGAATATTGAAACGAAAATATAAGAATAAAGATGATAA
AGAAGTTTGTAAAATCATAAATAAAACTTTCGCTGATATAAGAGATATTATAGGAGGTAC
TGATTATTGGAATGATTTGAGCAATAGAAAATTAGTAGGAAAAATTAACACAAATTCAAA
TTATGTTCACAGGAATAAACAAAATGATAAGCTTTTTCGTGATGAGTGGTGGAAAGTTAT
TAAAAAAGATGTATGGAATGTGATATCATGGGTATTCAAGGATAAAACTGTTTGTAAAGA
AGATGATATTGAAAATATACCACAATTCTTCAGATGGTTTAGTGAATGGGGTGATGATTA
TTGCCAGGATAAAACAAAAATG
>Equatorial Guinea (Bioko) 2019B19005F_MW691454.1
TATGTATGTATTCCTGATCGTAGAATCCAATTATGCATTGTTAATCTTAGCATTATTAAAA
CATATACAAAAGAGACCATGAAGGATCATTTCATTGAAGCCTCTAAAAAAGAATCTCAA
CTTTTGCTTAAAAAAAAATGATAACAAATATAATTCTAAATTTTGTAATGATTTGAAGAATA
GTTTTTTAGATTATGGACATCTTGCTATGGGAAATGATATGGATTTTGGAGGTTATTCAAC
TAAGGCAGAAAACAAAATTCAAGAAGTTTTTAAAGGGGCTCATGGGGAAATAAGTGAAC
ATAAAATTAAAAATTTTAGAAAAAAATGGTGGAATGAATTTAGAGAGAAACTTTGGGAA
GCTATGTTATCTGAGCATAAAAATAATATAAATAATTGTAAAAATATTCCCCAAGAAGAA
TTACAAATTACTCAATGGATAAAAGAATGGCATGGAGAATTTTTGCTTGAAAGAGATAAT
AGATCAAAATTGCCAAAAAGTAAATGTAAAAATAATACATTATATGAAGCATGTGAGAA
GGAATGTATTGATCCATGTATGAAATATAGAGATTGGATTATTAGAAGTAAATTTGAATG
GCATACGTTATCGAAAGAATATGAAACTCAAAATGTTTCAAAGGAAAATGCGGAAAATT
ATTTAATCAAAATTTCAAAAAACAAGAATGATGCTAAAGTAAGTTTATTATTGAATAATTG
TGATGCTGAATATTCAAAATATTGTGATTGTAAACATACTACTACTCTCGTTAAAAGCGTT
TTAAATGGTAACGACAATACAATTAAGGAAAAGCGTGAACATATTGATTTAGATGATTTT
TCTAAATTTGGATGTGATAAAAATTCCGTTGATACAAACACAAAGGTGTGGGAATGTAAA
AACCCTTATATATTATCCACTAAAGATGTATGTGTACCTCCGAGGAGGCAAGAATTATGT
CTTGGAAACATTGATAGAATATACGATAAAAACCTATTAATGATAAAAGAGCATATTCTT
GCTATTGCAATATATGAATCAAGAATATTGAAACGAAAATATAAGAATAAAGATGATAA
AGAAGTTTGTAAAATCATAAATAAAACTTTCGCTGATATAAGAGATATTATAGGAGGTAC
TGATTATTGGAATGATTTGAGCAATAGAAAATTAGTAGGAAAAATTAACACAAATTCAAA
TTATGTTCACAGGAATAAAGAAAATGATAAGCTTTTTCGTGATGCGTGGTGGAAAGTTAT
TAAAAAAGATGTATGGAATGTGATATCATGGGTATTCAAGGATAAAACTGTTTGTAAAGA
AGATGATATTGAAAATATACCACAATTCTTCAGATGGTTTAGTGAATGGGGTGATGATTA
TTGCCAGGATAAAACAAAAATG
>Equatorial Guinea (Bata) 201884F_MW691455.1
TATGTATGTATTCCTGATCGTAGAATCCAATTATGCATTGTTAATCTTAGCATTATTAAAA
CATATACAAAAGAGACCATGAAGGATCATTTCATTGAAGCCTCTAAAAAAGAATCTCAA
CTTTTGCTTAAAAAAAAATGATAACAAATATAATTCTAAATTTTGTAATGATTTGAAGAATA

GTTTTTTAGATTATGGACATCTTGCTATGGGAAATGATATGGATTTTGGAGGTTATTCAAC
TAAGGCAGAAAACAAAATTCAAGAAGTTTTTAAAGGGGCTCATGGGGAAATAAGTGAAC
ATAAAATTAAAAATTTTAGAAAAGAATGGTGGAATGAATTTAGAGAGAAACTTTGGGAA
GCTATGTTATCTGAGCATAAAAATAATATAAATAATTGTAAAAATATTCCCCAAGAAGAA
TTACAAATTACTCAATGGATAAAAGAATGGCATGGAGAATTTTTGCTTGAAAGAGATAAT
AGATCAAAATTGCCAAAAAGTAAATGTAAAAATAATACATTATATGAAGCATGTGAGAA
GGAATGTATTGATCCATGTATGAAATATAGAGATTGGATTATTAGAAGTAAATTTGAATG
GCATACGTTATCGAAAGAATATGAAACTCAAAAAGTTCCAAAGGAAAATGCGGAAAATT
ATTTAATCAAAATTTCAGAAAACAAGAATGATGCTAAAGTAAGTTTATTATTGAATAATT
GTGATGCTGAATATTCAAAATATTGTGATTGTAAACATACTACTACTCTCGTTAAAAGCGT
TTTAAATGGTAACGACAATACAATTAAGGAAAAGCGTGAACATATTGATTTAGATGATTT
TTCTAAATTTGGATGTGATAAAAATTCCGTTGATACAAACACAAAGGTGTGGGAATGTAA
AAAACCTTATAAATTATCCACTAAAGATGTATGTGTACCTCCGAGGAGGCAAGAATTATG
TCTTGGAAACATTGATAGAATATACGATAAAAACCTATTAATGATAAAAGAGCATATTCT
TGCTATTGCAATATATGAATCAAGAATATTGAAACGAAAATATAAGAATAAAGATGATAA
AGAAGTTTGTAAAATCATAAATAAAACTTTCGCTGATATAAGAGATATTATAGGAGGTAC
TGATTATTGGAATGATTTGAGCAATAGAAAATTAGTAGGAAAAATTAACACAAATTCAAA
TTATGTTCACAGGAATAAACAAAATGATAAGCTTTTTCGTGATGAGTGGTGGAAAGTTAT
TAAAAAAGATGTATGGAATGTGATATCATGGGTATTCAAGGATAAAACTGTTTGTAAAGA
AGATGATATTGAAAATATACCACAATTCTTCAGATGGTTTAGTGAATGGGGTGATGATTA
TTGCCAGGATAAAACAAAAATG
>Equatorial Guinea (Bata) 201834F_MW691456.1
TATGTATGTATTCCTGATCGTAGAATCCAATTATGCATTGTTAATCTTAGCATTATTAAAA
CATATACAAAAGAGACCATGAAGGATCATTTCATTGAAGCCTCTAAAAAAGAATCTCAA
CTTTTGCTTAAAAAAAAATGATAACAAATATAATTCTAAATTTTGTAATGATTTGAAGAATA
GTTTTTTAGATTATGGACATCTTGCTATGGGAAATGATATGGATTTTGGAGGTTATTCAAC
TAAGGCAGAAAACAAAATTCAAGAAGTTTTTAAAGGGGCTCATGGGGAAATAAGTGAAC
ATAAAATTAAAAATTTTAGAAAAGAATGGTGGAATGAATTTAGAGAGAAACTTTGGGAA
GCTATGTTATCTGAGCATAAAAATAATATAAATAATTGTAAAAATATTCCCCAAGAAGAA
TTACAAATTACTCAATGGATAAAAGAATGGCATGGAGAATTTTTGCTTGAAAGAGATAAT
AGATCAAAATTGCCAAAAAGTAAATGTAAAAATAATACATTATATGAAGCATGTGAGAA
GGAATGTATTGATCCATGTATGAAATATAGAGATTGGATTATTAGAAGTAAATTTGAATG
GCATACGTTATCGAAAGAATATGAAACTCAAAAAGTTCCAAAGGAAAATGCGGAAAATT
ATTTAATCAAAATTTCAGAAAACAAGAATGATGCTAAAGTAAGTTTATTATTGAATAATT
GTGATGCTGAATATTCAAAATATTGTGATTGTAAACATACTACTACTCTCGTTAAAAGCGT
TTTAAATGGTAACGACAATACAATTAAGGAAAAGCGTGAACATATTGATTTAGATGATTT
TTCTAAATTTGGATGTGATAAAAATTCCGTTGATACAAACACAAAGGTGTGGGAATGTAA
AAAACCTTATAAATTATCCACTAAAGATGTATGTGTACCTCCGAGGAGGCAAGAATTATG
TCTTGGAAACATTGATAGAATATACGATAAAAACCTATTAATGATAAAAGAGCATATTCT
TGCTATTGCAATATATGAATCAAGAATATTGAAACGAAAATATAAGAATAAAGATGATAA
AGAAGTTTGTAAAATCATAAATAAAACTTTCGCTGATATAAGAGATATTATAGGAGGTAC
TGATTATTGGAATGATTTGAGCAATAGAAAATTAGTAGGAAAAATTAACACAAATTCAAA
TTATGTTCACAGGAATAAACAAAATGATAAGCTTTTTCGTGATGAGTGGTGGAAAGTTAT
TAAAAAAGATGTATGGAATGTGATATCATGGGTATTCAAGGATAAAACTGTTTGTAAAGA

AGATGATATTGAAAATATACCACAATTCTTCAGATGGTTTAGTGAATGGGGTGATGATTA
TTGCCAGGATAAAACAAAAATG
>Equatorial Guinea (Bata) 201823F_MW691457.1
TATGTATGTATTCCTGATCGTAGAATCCAATTATGCATTGTTAATCTTAGCATTATTAAAA
CATATACAAAAGAGACCATGAAGGATCATTTCATTGAAGCCTCTAAAAAAGAATCTCAA
CTTTTGCTTAAAAAAAATGATAACAAATATAATTCTAAATTTTGTAATGATTTGAAGAATA
GTTTTTTAGATTATGGACATCTTGCTATGGGAAATGATATGGATTTTGGAGGTTATTCAAC
TAAGGCAGAAAACAAAATTCAAGAAGTTTTTAAAGGGGCTCATGGGGAAATAAGTGAAC
ATGAAATTAAAAATTTTAGAAAAAAATGGTGGAATGAATTTAGAGAGAAACTTTGGGAA
GCTATGTTATCTGAGCATAAAAATAATATAAATAATTGTAAAAATATTCCCCAAGAAGAA
TTACAAATTACTCAATGGATAAAAGAATGGCATGGAGAATTTTTGCTTGAAAGAGATAAT
AGATCAAAATTGCCAAAAAGTAAATGTAAAAATAATACATTATATGAAGCATGTGAGAA
GGAATGTATTGATCCATGTATGAAATATAGAGATTGGATTATTAGAAGTAAATTTGAATG
GCATACGTTATCGAAAGAATATGAAACTCAAAATGTTTCAAAGGAAAATGCGGAAAATT
ATTTAATCAAAATTTCAGAAAAAATGAATGATGCTAAAGTAAGTTTATTATTGAATAATTG
TGATGCTGAATATTCAAAATATTGTGATTGTAAACATACTACTACTCTCGTTAAAAGCGTT
TTAAATGGTAACGACAATACAATTAAGGAAAAGCGTGAACATATTGATTTAGATGATTTT
TCTAAATTTGGATGTGATAAAAATTCCGTTGATACAAACACAAAGGTGTGGGAATGTAAA
AAACCTTATAAAGTATCCACTAAAGATGTATGTGTACCTCCGAGGAGGCAAGAATTATGT
CTTGGAAACATTGATAGAATATACGATAAAAACCTATTAATGATAAAAGAGCATATTCTT
GCTATTGCAATATATGAATCAAGAATATTGAAACGAAAATATAAGAATAAAGATGATAA
AGAAGTTTGTAAAATCATAAATAAAACTTTCGCTGATATAAGAGATATTATAGGAGGTAC
TGATTATTGGAATGATTTGAGCAATAGAAAATTAGTAGGAAAAATTAACACAAATTCAAA
TTATGTTCACAGGAATAAAGAAAATGATAAGCTTTTTCGTGATGCGTGGTGGAAAGTTAT
TAAAAAAGATGTATGGAATGTGATATCATGGGTATTCAAGGATAAAACTGTTTGTAAAGA
AGATGATATTGAAAATATACCACAATTCTTCAGATGGTTTAGTGAATGGGGTGATGATTA
TTGCCAGGATAAAACAAAAATG
>Equatorial Guinea (Bata) 201810F_MW691458.1
TATGTATGTATTCCTGATCGTAGAATCCAATTATGCATTGTTAATCTTAGCATTATTAAAA
CATATACAAAAGAGACCATGAAGGATCATTTCATTGAAGCCTCTAAAAAAGAATCTCAA
CTTTTGCTTAAAAAAAATGATAACAAATATAATTCTAAATTTTGTAATGATTTGAAGAATA
GTTTTTTAGATTATGGACATCTTGCTATGGGAAATGATATGGATTTTGGAGGTTATTCAAC
TAAGGCAGAAAACAAAATTCAAGAAGTTTTTAAAGGGGCTCATGGGAAAATAAGTGAAC
ATGAAATTAAAAATTTTAGAAAAAAATGGTGGAATGAATTTAGAGAGAAACTTTGGGAA
GCTATGTTATCTGAGCATAAAAATAATATAAATAATTGTAAAAATATTCCCCAAGAAGAA
TTACAAATTACTCAATGGATAAAAGAATGGCATGGAGAATTTTTGCTTGAAAGATATAAT
AGATCAAAATTGCCAAAAAGTAAATGTAAAAATAATACATTATATGAAGCATGTGAGAA
GGAATGTATTGATCCATGTATGAAATATAGAGATTGGATTATTAGAAGTAAATTTGAATG
GCATACGTTATCGAAAGAATATGAAACTCAAAATGTTTCAAAGGAAAATGCGGAAAATT
ATTTAATCAAAATTTCAAAAAACAAGAATGATGCTAAAGTAAGTTTATTATTGAATAATTG
TGATGCTGAATATTCAAAATATTGTGATTGTAAACATACTACTACTCTCGTTAAAAGCGTT
TTAAATGGTAACGACAATACAATTAAGGAAAAGCGTGAACATATTGATTTAGATGATTTT
TCTAAATTTGGATGTGATAAAAATTCCGTTGATACAAACACAAAGGTGTGGGAATGTAAA
AAACCTTATAAATTATCCACTAAAGATGTATGTGTACCTCCGAGGAGGCAAGAATTATGT

CTTGGAAACATTGATAGAATATACGATAAAAACCTATTAATGATAAAAGAGCATATTCTT
GCTATTGCAATATATGAATCAAGAATATTGAAACGAAAATATAAGAATAAAGATGATAA
AGAAGTTTGTAAAATCATAAATAAAACTTTCGCTGATATAAGAGATATTATAGGAGGTAC
TGATTATTGGAATGATTTGAGCAATAGAAAATTAGTAGGAAAAATTAACACAAATTCAAA
TTATGTTCACAGGAATAAACAAAATGATAAGCTTTTTCGTGATGAGTGGTGGAAAGTTAT
TAAAAAAGATGTATGGAATGTGATATCATGGGTATTCAAGGATAAAACTGTTTGTAAAGA
AGATGATATTGAAAATATACCACAATTCTTCAGATGGTTTAGTGAATGGGGTGATGATTA
TTGCCAGGATAAAACAAAAATG
>Equatorial Guinea (Bata) 2019T19053C_MW691459.1
TATGTATGTATTCCTGATCGTAGAATCCAATTATGCATTGTTAATCTTAGCATTATTAAAA
CATATACAAAAGAGACCATGAAGGATCATTTCATTGAAGCCTCTAAAAAAGAATCTCAA
CTTTTGCTTAAAAAAAATGATAACAAATATAATTCTAAATTTTGTAATGATTTGAAGAATA
GTTTTTTAGATTATGGACATCTTGCTATGGGAAATGATATGGATTTTGGAGGTTATTCAAC
TAAGGCAGAAAACAAAATTCAAGAAGTTTTTAAAGGGGCTCATGGGGAAATAAGTGAAC
ATGAAATTAAAAATTTTAGAAAAAAAATGGTGGAATGAATTTAGAGAGAAACTTTGGGAA
GCTATGTTATCTGAGCATAAAAATAATATAAATAATTGTAAAAATATTCCCCAAGAAGAA
TTACAAATTACTCAATGGATAAAAGAATGGCATGGAGAATTTTTGCTTGAAAGAGATAAT
AGATCAAAATTGCCAAAAAGTAAATGTAAAAATAATACATTATATGAAGCATGTGAGAA
GGAATGTATTGATCCATGTATGAAATATAGAGATTGGATTATTAGAAGTAAATTTGAATG
GCATACGTTATCGAAAGAATATGAAACTCAAAAAGTTTCAAAGGAAAATGCGGAAAATT
ATTTAATCAAAATTTCAGAAAACAAGAATGATGCTAAAGTAAGTTTATTATTGAATAATT
GTGATGCTGAATATTCAAAATATTGTGATTGTAAACATACTACTACTCTCGTTAAAAGCGT
TTTAAATGGTAACGACAATACAATTAAGGAAAAGCGTGAACATATTGATTTAGATGATTT
TTCTAAATTTGGATGTGATAAAAATTCCGTTGATACAAACACAAAGGTGTGGGAATGTAA
AAAACCTTATATATTATCCACTAAAGATGTATGTGTACCTCCGAGGAGGCAAGAATTATG
TCTTGGAAACATTGATAGAATATACGATAAAAACCTATTAATGATAAAAGAGCATATTCT
TGCTATTGCAATATATGAATCAAGAATATTGAAACGAAAATATAAGAATAAAGATGATAA
AGAAGTTTGTAAAATCATAAATAAAACTTTCGCTGATATAAGAGATATTATAGGAGGTAC
TGATTATTGGAATGATTTGAGCAATAGAAAATTAGTAGGAAAAATTAACACAAATTCAAA
ATATGTTCACAGGAATAAAAAAAAATGATAAGCTTTTTCGTGATGAGTGGTGGAAAGTTAT
TAAAAAAGATGTATGGAATGTGATATCATGGGTATTCAAGGATAAAACTGTTTGTAAAGA
AGATGATATTGAAAATATACCACAATTCTTCAGATGGTTTAGTGAATGGGGTGATGATTA
TTGCCAGGATAAAACAAAAATG
>Equatorial Guinea (Bata) 2019T19058C_MW691460.1
TATGTATGTATTCCTGATCGTAGAATCCAATTATGCATTGTTAATCTTAGCATTATTAAAA
CATATACAAAAGAGACCATGAAGGATCATTTCATTGAAGCCTCTAAAAAAGAATCTCAA
CTTTTGCTTAAAAAAAATGATAACAAATATAATTCTAAATTTTGTAATGATTTGAAGAATA
GTTTTTTAGATTATGGACATCTTGCTATGGGAAATGATATGGATTTTGGAGGTTATTCAAC
TAAGGCAGAAAACAAAATTCAAGAAGTTTTTAAAGGGGCTCATGGGGAAATAAGTGAAC
ATGAAATTAAAAATTTTAGAAAAAAAATGGTGGAATGAATTTAGAGAGAAACTTTGGGAA
GCTATGTTATCTGAGCATAAAAATAATATAAATAATTGTAAAAATATTCCCCAAGAAGAA
TTACAAATTACTCAATGGATAAAAGAATGGCATGGAGAATTTTTGCTTGAAAGAGATAAT
AGATCAAAATTGCCAAAAAGTAAATGTAAAAATAATACATTATATGAAGCATGTGAGAA
GGAATGTATTGATCCATGTATGAAATATAGAGATTGGATTATTAGAAGTAAATTTGAATG

GCATACGTTATCGAAAGAATATGAAACTCAAAAAGTTTCAAAGGAAAATGCGGAAAATT
ATTTAATCAAAATTTCAGAAAACAAGAATGATGCTAAAGTAAGTTTATTATTGAATAATT
GTGATGCTGAATATTCAAAATATTGTGATTGTAAACATACTACTACTCTCGTTAAAAGCGT
TTTAAATGGTAACGACAATACAATTAAGGAAAAGCGTGAACATATTGATTTAGATGATTT
TTCTAAATTTGGATGTGATAAAAATTCCGTTGATACAAACACAAAGGTGTGGGAATGTAA
AAAACCTTATATATTATCCACTAAAGATGTATGTGTACCTCCGAGGAGGCAAGAATTATG
TCTTGGAAACATTGATAGAATATACGATAAAAACCTATTAATGATAAAAGAGCATATTCT
TGCTATTGCAATATATGAATCAAGAATATTGAAACGAAAATATAAGAATAAAGATGATAA
AGAAGTTTGTAAAATCATAAATAAAACTTTCGCTGATATAAGAGATATTATAGGAGGTAC
TGATTATTGGAATGATTTGAGCAATAGAAAATTAGTAGGAAAAATTAACACAAATTCAAA
ATATGTTCACAGGAATAAAAAAAATGATAAGCTTTTTCGTGATGAGTGGTGGAAAGTTAT
TAAAAAAGATGTATGGAATGTGATATCATGGGTATTCAAGGATAAAACTGTTTGTAAAGA
AGATGATATTGAAAATATACCACAATTCTTCAGATGGTTTAGTGAATGGGGTGATGATTA
TTGCCAGGATAAAACAAAAATG
>Equatorial Guinea (Bata) 2019T19034C_MW691461.1
TATGTATGTATTCCTGATCGTAGAATCCAATTATGCATTGTTAATCTTAGCATTATTAAAA
CATATACAAAAGAGACCATGAAGGATCATTTCATTGAAGCCTCTAAAAAAGAATCTCAA
CTTTTGCTTAAAAAAAATGATAACAAATATAATTCTAAATTTTGTAATGATTTGAAGAATA
GTTTTTTAGATTATGGACATCTTGCTATGGGAAATGATATGGATTTTGGAGGTTATTCAAC
TAAGGCAGAAAACAAAATTCAAGAAGTTTTTAAAGGGGCTCATGGGAAAATAAGTGAAC
ATGAAATTAAAAATTTTAGAAAAAAATGGTGGAATGAATTTAGAGAGAAACTTTGGGAA
GCTATGTTATCTGAGCATAAAAATAATATAAATAATTGTAAAAATATTCCCCAAGAAGAA
TTACAAATTACTCAATGGATAAAAGAATGGCATGGAGAATTTTTGCTTGAAAGAGATAAT
AGATCAAAATTGCCAAAAAGTAAATGTAAAAATAATACATTATATGAAGCATGTGAGAA
GGAATGTATTGATCCATGTATGAAATATAGAGATTGGATTATTAGAAGTAAATTTGAATG
GCATACGTTATCGAAAGAATATGAAACTCAAAATGTTTCAAAGGAAAATGCGGAAAATT
ATTTAATCAAAATTTCAAAAAACAAGAATGATGCTAAAGTAAGTTTATTATTGAATAATTG
TGATGCTGAATATTCAAAATATTGTGATTGTAAACATACTACTACTCTCGTTAAAAGCGTT
TTAAATGGTAACGACAATACAATTAAGGAAAAGCGTGAACATATTGATTTAGATGATTTT
TCTAAATTTGGATGTGATAAAAATTCCGTTGATACAAACACAAAGGTGTGGGAATGTAAA
AAACCTTATAAATTATCCACTAAAGATGTATGTGTACCTCCGAGGAGGCAAGAATTATGT
CTTGGAAACATTGATAGAATATACGATAAAAACCTATTAATGATAAAAGAGCATATTCTT
GCTATTGCAATATATGAATCAAGAATATTGAAACGAAAATATAAGAATAAAGATGATAA
AGAAGTTTGTAAAATCATAAATAAAACTTTCGCTGATATAAGAGATATTATAGGAGGTAC
TGATTATTGGAATGATTTGAGCAATAGAAAATTAGTAGGAAAAATTAACACAAATTCAAA
TTATGTTCACAGGAATAAACAAAATGATAAGCTTTTTCGTGATGAGTGGTGGAAAGTTAT
TAAAAAAGATGTATGGAATGTGATATCATGGGTATTCAAGGATAAAACTGTTTGTAAAGA
AGATGATATTGAAAATATACCACAATTCTTCAGATGGTTTAGTGAATGGGGTGATGATTA
TTGCCAGGATAAAACAAAAATG
>Equatorial Guinea (Bata) 2019T19033C_MW691462.1
TATGTATGTATTCCTGATCGTAGAATCCAATTATGCATTGTTAATCTTAGCATTATTAAAA
CATATACAAAAGAGACCATGAAGGATCATTTCATTGAAGCCTCTAAAAAAGAATCTCAA
CTTTTGCTTAAAAAAAATGATAACAAATATAATTCTAAATTTTGTAATGATTTGAAGAATA
GTTTTTTAGATTATGGACATCTTGCTATGGGAAATGATATGGATTTTGGAGGTTATTCAAC

TAAGGCAGAAAACAAAATTCAAGAAGTTTTTAAAGGGGCTCATGGGGAAATAAGTGAAC
ATGAAATTAAAAATTTTAGAAAAAAATGGTGGAATGAATTTAGAGAGAAACTTTGGGAA
GCTATGTTATCTGAGCATAAAAATAATATAAATAATTGTAAAAATATTCCCCAAGAAGAA
TTACAAATTACTCAATGGATAAAAGAATGGCATGGAGAATTTTTGCTTGAAAGAGATAAT
AGATCAAAATTGCCAAAAAGTAAATGTAAAAATAATACATTATATGAAGCATGTGAGAA
GGAATGTATTGATCCATGTATGAAATATAGAGATTGGATTATTAGAAGTAAATTTGAATG
GCATACGTTATCGAAAGAATATGAAACTCAAAAAGTTTCAAAGGAAAATGCGGAAAATT
ATTTAATCAAAATTTCAGAAAACAAGAATGATGCTAAAGTAAGTTTATTATTGAATAATT
GTGATGCTGAATATTCAAAATATTGTGATTGTAAACATACTACTACTCTCGTTAAAAGCGT
TTTAAATGGTAACGACAATACAATTAAGGAAAAGCGTGAACATATTGATTTAGATGATTT
TTCTAAATTTGGATGTGATAAAAATTCCGTTGATACAAACACAAAGGTGTGGGAATGTAA
AAAACCTTATATATTATCCACTAAAGATGTATGTGTACCTCCGAGGAGGCAAGAATTATG
TCTTGGAAACATTGATAGAATATACGATAAAAACCTATTAATGATAAAAGAGCATATTCT
TGCTATTGCAATATATGAATCAAGAATATTGAAACGAAAATATAAGAATAAAGATGATAA
AGAAGTTTGTAAAATCATAAATAAAACTTTCGCTGATATAAGAGATATTATAGGAGGTAC
TGATTATTGGAATGATTTGAGCAATAGAAAATTAGTAGGAAAAATTAACACAAATTCAAA
ATATGTTCACAGGAATAAAAAAAAATGATAAGCTTTTTCGTGATGAGTGGTGGAAAGTTAT
TAAAAAAGATGTATGGAATGTGATATCATGGGTATTCAAGGATAAAACTGTTTGTAAAGA
AGATGATATTGAAAATATACCACAATTCTTCAGATGGTTTAGTGAATGGGGTGATGATTA
TTGCCAGGATAAAACAAAAATG
>Equatorial Guinea (Bata) 2019T19022C_MW691463.1
TATGTATGTATTCCTGATCGTAGAATCCAATTATGCATTGTTAATCTTAGCATTATTAAAA
CATATACAAAAGAGACCATGAAGGATCATTTCATTGAAGCCTCTAAAAAAGAATCTCAA
CTTTTGCTTAAAAAAAAATGATAACAAATATAATTCTAAATTTTGTAATGATTTGAAGAATA
GTTTTTTAGATTATGGACATCTTGCTATGGGAAATGATATGGATTTTGGAGGTTATTCAAC
TAAGGCAGAAAACAAAATTCAAGAAGTTTTTAAAGGGGCTCATGGGGAAATAAGTGAAC
ATGAAATTAAAAATTTTAGAAAAGAATGGTGGAATGAATTTAGAGAGAAACTTTGGGAA
GCTATGTTATCTGAGCATAAAAATAATATAAATAATTGTAAAAATATTCCCCAAGAAGAA
TTACAAATTACTCAATGGATAAAAGAATGGCATGGAGAATTTTTGCTTGAAAGAGATAAT
AGATCAAAATTGCCAAAAAGTAAATGTAAAAATAATACATTATATGAAGCATGTGAGAA
GGAATGTATTGATCCATGTATGAAATATAGAGATTGGATTATTAGAAGTAAATTTGAATG
GCATACGTTATCGAAAGAATATGAAACTCAAAAAGTTCCAAAGGAAAATGCGGAAAATT
ATTTAATCAAAATTTCAGAAAACAAGAATGATGCTAAAGTAAGTTTATTATTGAATAATT
GTGATGCTGAATATTCAAAATATTGTGATTGTAAACATACTACTACTCTCGTTAAAAGCGT
TTTAAATGGTAACGACAATACAATTAAGGAAAAGCGTGAACATATTGATTTAGATGATTT
TTCTAAATTTGGATGTGATAAAAATTCCGTTGATACAAACACAAAGGTGTGGGAATGTAA
AAAACCTTATAAATTATCCACTAAAGATGTATGTGTACCTCCGAGGAGGCAAGAATTATG
TCTTGGAAACATTGATAGAATATACGATAAAAACCTATTAATGATAAAAGAGCATATTCT
TGCTATTGCAATATATGAATCAAGAATATTGAAACGAAAATATAAGAATAAAGATGATAA
AGAAGTTTGTAAAATCATAAATAAAACTTTCGCTGATATAAGAGATATTATAGGAGGTAC
TGATTATTGGAATGATTTGAGCAATAGAAAATTAGTAGGAAAAATTAACACAAATTCAAA
TTATGTTCACAGGAATAAACAAAATGATAAGCTTTTTCGTGATGAGTGGTGGAAAGTTAT
TAAAAAAGATGTATGGAATGTGATATCATGGGTATTCAAGGATAAAACTGTTTGTAAAGA
AGATGATATTGAAAATATACCACAATTCTTCAGATGGTTTAGTGAATGGGGTGATGATTA

TTGCCAGGATAAAACAAAAATG
>Equatorial Guinea (Bata) 2019T19019C_MW691464.1
TATGTATGTATTCCTGATCGTAGAATCCAATTATGCATTGTTAATCTTAGCATTATTAAAA
CATATACAAAAGAGACCATGAAGGATCATTTCATTGAAGCCTCTAAAAAAGAATCTCAA
CTTTTGCTTAAAAAAAATGATAACAAATATAATTCTAAATTTTGTAATGATTTGAAGAATA
GTTTTTTAGATTATGGACATCTTGCTATGGGAAATGATATGGATTTTGGAGGTTATTCAAC
TAAGGCAGAAAACAAAATTCAAGAAGTTTTTAAAGGGGCTCATGGGAAAATAAGTGAAC
ATGAAATTAAAAATTTTAGAAAAAAATGGTGGAATGAATTTAGAGAGAAACTTTGGGAA
GCTATGTTATCTGAGCATAAAAATAATATAAATAATTGTAAAAATATTCCCCAAGAAGAA
TTACAAATTACTCAATGGATAAAAGAATGGCATGGAGAATTTTTGCTTGAAAGAGATAAT
AGATCAAAATTGCCAAAAAGTAAATGTAAAAATAATACATTATATGAAGCATGTGAGAA
GGAATGTATTGATCCATGTATGAAATATAGAGATTGGATTATTAGAAGTAAATTTGAATG
GCATACGTTATCGAAAGAATATGAAACTCAAAATGTTTCAAAGGAAAATGCGGAAAATT
ATTTAATCAAAATTTCAGAAAAAATGAATGATGCTAAAGTAAGTTTATTATTGAATAATTG
TGATGCTGAATATTCAAAATATTGTGATTGTAAACATACTACTACTCTCGTTAAAAGCGTT
TTAAATGGTAACGACAATACAATTAAGGAAAAGCGTGAACATATTGATTTAGATGATTTT
TCTAAATTTGGATGTGATAAAAATTCCGTTGATACAAACACAAAGGTGTGGGAATGTAAA
AAACCTTATAAATTATCCACTAAAGATGTATGTGTACCTCCGAGGAGGCAAGAATTATGT
CTTGGAAACATTGATAGAATATACGATAAAAACCTATTAATGATAAAAGAGCATATTCTT
GCTATTGCAATATATGAATCAAGAATATTGAAACGAAAATATAAGAATAAAGATGATAA
AGAAGTTTGTAAAATCATAAATAAAACTTTCGCTGATATAAGAGATATTATAGGAGGTAC
TGATTATTGGAATGATTTGAGCAATAGAAAATTAGTAGGAAAAATTAACACAAATTCAAA
TTATGTTCACAGGAATAAAAAAAATGATAAGCTTTTTCGTGATGAGTGGTGGAAAGTTAT
TAAAAAAGATGTATGGAATGTGATATCATGGGTATTCAAGGATAAAACTGTTTGTAAAGA
AGATGATATTGAAAATATACCACAATTCTTCAGATGGTTTAGTGAATGGGGTGATGATTA
TTGCCAGGATAAAACAAAAATG
>Equatorial Guinea (Bata) 2019T19016C_MW691465.1
TATGTATGTATTCCTGATCGTAGAATCCAATTATGCATTGTTAATCTTAGCATTATTAAAA
CATATACAAAAGAGACCATGAAGGATCATTTCATTGAAGCCTCTAAAAAAGAATCTCAA
CTTTTGCTTAAAAAAAATGATAACAAATATAATTCTAAATTTTGTAATGATTTGAAGAATA
GTTTTTTAGATTATGGACATCTTGCTATGGGAAATGATATGGATTTTGGAGGTTATTCAAC
TAAGGCAGAAAACAAAATTCAAGAAGTTTTTAAAGGGGCTCATGGGGAAATAAGTGAAC
ATAAAATTAAAAATTTTAGAAAAGAATGGTGGAATGAATTTAGAGAGAAACTTTGGGAA
GCTATGTTATCTGAGCATAAAAATAATATAAATAATTGTAAAAATATTCCCCAAGAAGAA
TTACAAATTACTCAATGGATAAAAGAATGGCATGGAGAATTTTTGCTTGAAAGAGATAAT
AGATCAAAATTGCCAAAAAGTAAATGTAAAAATAATACATTATATGAAGCATGTGAGAA
GGAATGTATTGATCCATGTATGAAATATAGAGATTGGATTATTAGAAGTAAATTTGAATG
GCATACGTTATCGAAAGAATATGAAACTCAAAAAGTTCCAAAGGAAAATGCGGAAAATT
ATTTAATCAAAATTTCAGAAAACAAGAATGATGCTAAAGTAAGTTTATTATTGAATAATT
GTGATGCTGAATATTCAAAATATTGTGATTGTAAACATACTACTACTCTCGTTAAAAGCGT
TTTAAATGGTAACGACAATACAATTAAGGAAAAGCGTGAACATATTGATTTAGATGATTT
TTCTAAATTTGGATGTGATAAAAATTCCGTTGATACAAACACAAAGGTGTGGGAATGTAA
AAAACCTTATAAATTATCCACTAAAGATGTATGTGTACCTCCGAGGAGGCAAGAATTATG
TCTTGGAAACATTGATAGAATATACGATAAAAACCTATTAATGATAAAAGAGCATATTCT

TGCTATTGCAATATATGAATCAAGAATATTGAAACGAAAATATAAGAATAAAGATGATAA
AGAAGTTTGTAAAATCATAAATAAAACTTTCGCTGATATAAGAGATATTATAGGAGGTAC
TGATTATTGGAATGATTTGAGCAATAGAAAATTAGTAGGAAAAATTAACACAAATTCAAA
TTATGTTCACAGGAATAAACAAAATGATAAGCTTTTTCGTGATGAGTGGTGGAAAGTTAT
TAAAAAAGATGTATGGAATGTGATATCATGGGTATTCAAGGATAAAACTGTTTGTAAAGA
AGATGATATTGAAAATATACCACAATTCTTCAGATGGTTTAGTGAATGGGGTGATGATTA
TTGCCAGGATAAAACAAAAATG
>Equatorial Guinea (Bioko) 2019B19072C_MW691466.1
TATGTATGTATTCCTGATCGTAGAATCCAATTATGCATTGTTAATCTTAGCATTATTAAAA
CATATACAAAAGAGACCATGAAGGATCATTTCATTGAAGCCTCTAAAAAAGAATCTCAA
CTTTTGCTTAAAAAAAAATGATAACAAATATAATTCTAAATTTTGTAATGATTTGAAGAATA
GTTTTTTAGATTATGGACATCTTGCTATGGGAAATGATATGGATTTTGGAGGTTATTCAAC
TAAGGCAGAAAACAAAATTCAAGAAGTTTTTAAAGGGGCTCATGGGGAAATAAGTGAAC
ATGAAATTAAAAATTTTAGAAAAAAATGGTGGAATGAATTTAGAGAGAAACTTTGGGAA
GCTATGTTATCTGAGCATAAAAATAATATAAATAATTGTAAAAATATTCCCCAAGAAGAA
TTACAAATTACTCAATGGATAAAAGAATGGCATGGAGAATTTTTGCTTGAAAGAGATAAT
AGATCAAAATTGCCAAAAAGTAAATGTAAAAATAATACATTATATGAAGCATGTGAGAA
GGAATGTATTGATCCATGTATGAAATATAGAGATTGGATTATTAGAAGTAAATTTGAATG
GCATACGTTATCGAAAGAATATGAAACTCAAAAAGTTTCAAAGGAAAATGCGGAAAATT
ATTTAATCAAAATTTCAGAAAACAAGAATGATGCTAAAGTAAGTTTATTATTGAATAATT
GTGATGCTGAATATTCAAAATATTGTGATTGTAAACATACTACTACTCTCGTTAAAAGCGT
TTTAAATGGTAACGACAATACAATTAAGGAAAAGCGTGAACATATTGATTTAGATGATTT
TTCTAAATTTGGATGTGATAAAAATTCCGTTGATACAAACACAAAGGTGTGGGAATGTAA
AAAACCTTATATATTATCCACTAAAGATGTATGTGTACCTCCGAGGAGGCAAGAATTATG
TCTTGGAAACATTGATAGAATATACGATAAAAACCTATTAATGATAAAAGAGCATATTCT
TGCTATTGCAATATATGAATCAAGAATATTGAAACGAAAATATAAGAATAAAGATGATAA
AGAAGTTTGTAAAATCATAAATAAAACTTTCGCTGATATAAGAGATATTATAGGAGGTAC
TGATTATTGGAATGATTTGAGCAATAGAAAATTAGTAGGAAAAATTAACACAAATTCAAA
ATATGTTCACAGGAATAAAAAAAAATGATAAGCTTTTTCGTGATGAGTGGTGGAAAGTTAT
TAAAAAAGATGTATGGAATGTGATATCATGGGTATTCAAGGATAAAACTGTTTGTAAAGA
AGATGATATTGAAAATATACCACAATTCTTCAGATGGTTTAGTGAATGGGGTGATGATTA
TTGCCAGGATAAAACAAAAATG
>Equatorial Guinea (Bioko) 2019B19065C_MW691467.1
TATGTATGTATTCCTGATCGTAGAATCCAATTATGCATTGTTAATCTTAGCATTATTAAAA
CATATACAAAAGAGACCATGAAGGATCATTTCATTGAAGCCTCTAAAAAAGAATCTCAA
CTTTTGCTTAAAAAAAAATGATAACAAATATAATTCTAAATTTTGTAATGATTTGAAGAATA
GTTTTTTAGATTATGGACATCTTGCTATGGGAAATGATATGGATTTTGGAGGTTATTCAAC
TAAGGCAGAAAACAAAATTCAAGAAGTTTTTAAAGGGGCTCATGGGAAAATAAGTGAAC
ATGAAATTAAAAATTTTAGAAAAAAATGGTGGAATGAATTTAGAGAGAAACTTTGGGAA
GCTATGTTATCTGAGCATAAAAATAATATAAATAATTGTAAAAATATTCCCCAAGAAGAA
TTACAAATTACTCAATGGATAAAAGAATGGCATGGAGAATTTTTGCTTGAAAGAGATAAT
AGATCAAAATTGCCAAAAAGTAAATGTAAAAATAATACATTATATGAAGCATGTGAGAA
GGAATGTATTGATCCATGTATGAAATATAGAGATTGGATTATTAGAAGTAAATTTGAATG
GCATACGTTATCGAAAGAATATGAAACTCAAAATGTTTCAAAGGAAAATGCGGAAAATT

ATTTAATCAAAATTTCAGAAAAAATGAATGATGCTAAAGTAAGTTTATTATTGAATAATTG
TGATGCTGAATATTCAAAATATTGTGATTGTAAACATACTACTACTCTCGTTAAAAGCGTT
TTAAATGGTAACGACAATACAATTAAGGAAAAGCGTGAACATATTGATTTAGATGATTTT
TCTAAATTTGGATGTGATAAAAATTCCGTTGATACAAACACAAAGGTGTGGGAATGTAAA
AAACCTTATAAATTATCCACTAAAGATGTATGTGTACCTCCGAGGAGGCAAGAATTATGT
CTTGGAAACATTGATAGAATATACGATAAAAACCTATTAATGATAAAAGAGCATATTCTT
GCTATTGCAATATATGAATCAAGAATATTGAAACGAAAATATAAGAATAAAGATGATAA
AGAAGTTTGTAAAATCATAAATAAAACTTTCGCTGATATAAGAGATATTATAGGAGGTAC
TGATTATTGGAATGATTTGAGCAATAGAAAATTAGTAGGAAAAATTAACACAAATTCAAA
TTATGTTCACAGGAATAAACAAAATGATAAGCTTTTTCGTGATGAGTGGTGGAAAGTTAT
TAAAAAAGATGTATGGAATGTGATATCATGGGTATTCAAGGATAAAACTGTTTGTAAAGA
AGATGATATTGAAAATATACCACAATTCTTCAGATGGTTTAGTGAATGGGGTGATGATTA
TTGCCAGGATAAAACAAAAATG
>Equatorial Guinea (Bioko) 2019B19064C_MW691468.1
TATGTATGTATTCCTGATCGTAGAATCCAATTATGCATTGTTAATCTTAGCATTATTAAAA
CATATACAAAAGAGACCATGAAGGATCATTTCATTGAAGCCTCTAAAAAAGAATCTCAA
CTTTTGCTTAAAAAAAAATGATAACAAATATAATTCTAAATTTTGTAATGATTTGAAGAATA
GTTTTTTAGATTATGGACATCTTGCTATGGGAAATGATATGGATTTTGGAGGTTATTCAAC
TAAGGCAGAAAACAAAATTCAAGAAGTTTTTAAAGGGGCTCATGGGAAAATAAGTGAAC
ATGAAATTAAAAATTTTAGAAAAAAATGGTGGAATGAATTTAGAGAGAAACTTTGGGAA
GCTATGTTATCTGAGCATAAAAATAATATAAATAATTGTAAAAATATTCCCCAAGAAGAA
TTACAAATTACTCAATGGATAAAAGAATGGCATGGAGAATTTTTGCTTGAAAGAGATAAT
AGATCAAAATTGCCAAAAAGTAAATGTAAAAATAATACATTATATGAAGCATGTGAGAA
GGAATGTATTGATCCATGTATGAAATATAGAGATTGGATTATTAGAAGTAAATTTGAATG
GCATACGTTATCGAAAGAATATGAAACTCAAAAAGTTTCAAAGGAAAATGCGGAAAATT
ATTTAATCAAAATTTCAGAAAACAAGAATGATGCTAAAGTAAGTTTATTATTGAATAATT
GTGATGCTGAATATTCAAAATATTGTGATTGTAAACATACTACTACTCTCGTTAAAAGCGT
TTTAAATGGTAACGACAATACAATTAAGGAAAAGCGTGAACATATTGATTTAGATGATTT
TTCTAAATTTGGATGTGATAAAAATTCCGTTGATACAAACACAAAGGTGTGGGAATGTAA
AAAACCTTATATATTATCCACTAAAGATGTATGTGTACCTCCGAGGAGGCAAGAATTATG
TCTTGGAAACATTGATAGAATATACGATAAAAACCTATTAATGATAAAAGAGCATATTCT
TGCTATTGCAATATATGAATCAAGAATATTGAAACGAAAATATAAGAATAAAGATGATAA
AGAAGTTTGTAAAATCATAAATAAAACTTTCGCTGATATAAGAGATATTATAGGAGGTAC
TGATTATTGGAATGATTTGAGCAATAGAAAATTAGTAGGAAAAATTAACACAAATTCAAA
ATATGTTCACAGGAATAAAAAAAATGATAAGCTTTTTCGTGATGAGTGGTGGAAAGTTAT
TAAAAAAGATGTATGGAATGTGATATCATGGGTATTCAAGGATAAAACTGTTTGTAAAGA
AGATGATATTGAAAATATACCACAATTCTTCAGATGGTTTAGTGAATGGGGTGATGATTA
TTGCCAGGATAAAACAAAAATG
>Equatorial Guinea (Bioko) 2019B19060C_MW691469.1
TATGTATGTATTCCTGATCGTAGAATCCAATTATGCATTGTTAATCTTAGCATTATTAAAA
CATATACAAAAGAGACCATGAAGGATCATTTCATTGAAGCCTCTAAAAAAGAATCTCAA
CTTTTGCTTAAAAAAAAATGATAACAAATATAATTCTAAATTTTGTAATGATTTGAAGAATA
GTTTTTTAGATTATGGACATCTTGCTATGGGAAATGATATGGATTTTGGAGGTTATTCAAC
TAAGGCAGAAAACAAAATTCAAGAAGTTTTTAAAGGGGCTCATGGGAAAATAAGTGAAC

ATGAAATTAAAAATTTTAGAAAAAAATGGTGGAATGAATTTAGAGAGAAACTTTGGGAA
GCTATGTTATCTGAGCATAAAAATAATATAAATAATTGTAAAAATATTCCCCAAGAAGAA
TTACAAATTACTCAATGGATAAAAGAATGGCATGGAGAATTTTTGCTTGAAAGAGATAAT
AGATCAAAATTGCCAAAAAGTAAATGTAAAAATAATACATTATATGAAGCATGTGAGAA
GGAATGTATTGATCCATGTATGAAATATAGAGATTGGATTATTAGAAGTAAATTTGAATG
GCATACGTTATCGAAAGAATATGAAACTCAAAATGTTTCAAAGGAAAATGCGGAAAATT
ATTTAATCAAAATTTCAGAAAACAAGAATGATGCTAAAGTAAGTTTATTATTGAATAATT
GTGATGCTGAATATTCAAAATATTGTGATTGTAAACATACTACTACTCTCGTTAAAAGCGT
TTTAAATGGTAACGACAATACAATTAAGGAAAAGCGTGAACATATTGATTTAGATGATTT
TTCTAAATTTGGATGTGATAAAAATTCCGTTGATACAAACACAAAGGTGTGGGAATGTAA
AAAACCTTATAAATTATCCACTAAAGATGTATGTGTACCTCCGAGGAGGCAAGAATTATG
TCTTGGAAACATTGATAGAATATACGATAAAAACCTATTAATGATAAAAGAGCATATTCT
TGCTATTGCAATATATGAATCAAGAATATTGAAACGAAAATATAAGAATAAAGATGATAA
AGAAGTTTGTAAAATCATAAATAAAACTTTCGCTGATATAAGAGATATTATAGGAGGTAC
TGATTATTGGAATGATTTGAGCAATAGAAAATTAGTAGGAAAAATTAACACAAATTCAAA
TTATGTTCACAGGAATAAACAAAATGATAAGCTTTTTCGTGATGAGTGGTGGAAAGTTAT
TAAAAAAGATGTATGGAATGTGATATCATGGGTATTCAAGGATAAAACTGTTTGTAAAGA
AGATGATATTGAAAATATACCACAATTCTTCAGATGGTTTAGTGAATGGGGTGATGATTA
TTGCCAGGATAAAACAAAAATG
>Equatorial Guinea (Bioko) 2019B19059C_MW691470.1
TATGTATGTATTCCTGATCGTAGAATCCAATTATGCATTGTTAATCTTAGCATTATTAAAA
CATATACAAAAGAGACCATGAAGGATCATTTCATTGAAGCCTCTAAAAAAGAATCTCAA
CTTTTGCTTAAAAAAAAATGATAACAAATATAATTCTAAATTTTGTAATGATTTGAAGAATA
GTTTTTTAGATTATGGACATCTTGCTATGGGAAATGATATGGATTTTGGAGGTTATTCAAC
TAAGGCAGAAAACAAAATTCAAGAAGTTTTTAAAGGGGCTCATGGGAAAATAAGTGAAC
ATGAAATTAAAAATTTTAGAAAAAAATGGTGGAATGAATTTAGAGAGAAACTTTGGGAA
GCTATGTTATCTGAGCATAAAAATAATATAAATAATTGTAAAAATATTCCCCAAGAAGAA
TTACAAATTACTCAATGGATAAAAGAATGGCATGGAGAATTTTTGCTTGAAAGAGATAAT
AGATCAAAATTGCCAAAAAGTAAATGTAAAAATAATACATTATATGAAGCATGTGAGAA
GGAATGTATTGATCCATGTATGAAATATAGAGATTGGATTATTAGAAGTAAATTTGAATG
GCATACGTTATCGAAAGAATATGAAACTCAAAATGTTTCAAAGGAAAATGCGGAAAATT
ATTTAATCAAAATTTCAGAAAAAAATGAATGATGCTAAAGTAAGTTTATTATTGAATAATTG
TGATGCTGAATATTCAAAATATTGTGATTGTAAACATACTACTACTCTCGTTAAAAGCGTT
TTAAATGGTAACGACAATACAATTAAGGAAAAGCGTGAACATATTGATTTAGATGATTTT
TCTAAATTTGGATGTGATAAAAATTCCGTTGATACAAACACAAAGGTGTGGGAATGTAAA
AAACCTTATAAATTATCCACTAAAGATGTATGTGTACCTCCGAGGAGGCAAGAATTATGT
CTTGGAAACATTGATAGAATATACGATAAAAACCTATTAATGATAAAAGAGCATATTCTT
GCTATTGCAATATATGAATCAAGAATATTGAAACGAAAATATAAGAATAAAGATGATAA
AGAAGTTTGTAAAATCATAAATAAAACTTTCGCTGATATAAGAGATATTATAGGAGGTAC
TGATTATTGGAATGATTTGAGCAATAGAAAATTAGTAGGAAAAATTAACACAAATTCAAA
TTATGTTCACAGGAATAAAAAAAAATGATAAGCTTTTTCGTGATGAGTGGTGGAAAGTTAT
TAAAAAAGATGTATGGAATGTGATATCATGGGTATTCAAGGATAAAACTGTTTGTAAAGA
AGATGATATTGAAAATATACCACAATTCTTCAGATGGTTTAGTGAATGGGGTGATGATTA
TTGCCAGGATAAAACAAAAATG

>Equatorial Guinea (Bioko) 2019B19049C_MW691471.1
TATGTATGTATTCCTGATCGTAGAATCCAATTATGCATTGTTAATCTTAGCATTATTAAAA
CATATACAAAAGAGACCATGAAGGATCATTTCATTGAAGCCTCTAAAAAAGAATCTCAA
CTTTTGCTTAAAAAAAAATGATAACAAATATAATTCTAAATTTTGTAATGATTTGAAGAATA
GTTTTTTAGATTATGGACATCTTGCTATGGGAAATGATATGGATTTTGGAGGTTATTCAAC
TAAGGCAGAAAACAAAATTCAAGAAGTTTTTAAAGGGGCTCATGGGAAAATAAGTGAAC
ATGAAATTAAAAATTTTAGAAAAGAATGGTGGAATGAATTTAGAGAGAAACTTTGGGAA
GCTATGTTATCTGAGCATAAAAATAATATAAATAATTGTAAAAATATTCCCCAAGAAGAA
TTACAAATTACTCAATGGATAAAAGAATGGCATGGAGAATTTTTGCTTGAAAGAGATAAT
AGATCAAAATTGCCAAAAAGTAAATGTAAAAATAATACATTATATGAAGCATGTGAGAA
GGAATGTATTGATCCATGTATGAAATATAGAGATTGGATTATTAGAAGTAAATTTGAATG
GCATACGTTATCGAAAGAATATGAAACTCAAAATGTTTCAAAGGAAAATGCGGAAAATT
ATTTAATCAAAATTTCAGAAAAAATGAATGATGCTAAAGTAAGTTTATTATTGAATAATTG
TGATGCTGAATATTCAAAATATTGTGATTGTAAACATACTACTACTCTCGTTAAAAGCGTT
TTAAATGGTAACGACAATACAATTAAGGAAAAGCGTGAACATATTGATTTAGATGATTTT
TCTAAATTTGGATGTGATAAAAAATTCCGTTGATACAAACACAAAGGTGTGGGAATGTAAA
AACCCTTATATATTATCCACTAAAGATGTATGTGTACCTCCGAGGAGGCAAGAATTATGT
CTTGGAAACATTGATAGAATATACGATAAAAACCTATTAATGATAAAAGAGCATATTCTT
GCTATTGCAATATATGAATCAAGAATATTGAAACGAAAATATAAGAATAAAGATGATAA
AGAAGTTTGTAAAATCATAAATAAAACTTTCGCTGATATAAGAGATATTATAGGAGGTAC
TGATTATTGGAATGATTTGAGCAATAGAAAATTAGTAGGAAAAATTAACACAAATTCAAA
TTATGTTCACAGGAATAAAAAAAATGATAAGCTTTTTCGTGATGAGTGGTGGAAAGTTAT
TAAAAAAGATGTATGGAATGTGATATCATGGGTATTCAAGGATAAAACTGTTTGTAAAGA
AGATGATATTGAAAATATACCACAATTCTTCAGATGGTTTAGTGAATGGGGTGATGATTA
TTGCCAGGATAAAACAAAAATG
>Equatorial Guinea (Bioko) 2019B19046C_MW691472.1
TATGTATGTATTCCTGATCGTAGAATCCAATTATGCATTGTTAATCTTAGCATTATTAAAA
CATATACAAAAGAGACCATGAAGGATCATTTCATTGAAGCCTCTAAAAAAGAATCTCAA
CTTTTGCTTAAAAAAAAATGATAACAAATATAATTCTAAATTTTGTAATGATTTGAAGAATA
GTTTTTTAGATTATGGACATCTTGCTATGGGAAATGATATGGATTTTGGAGGTTATTCAAC
TAAGGCAGAAAACAAAATTCAAGAAGTTTTTAAAGGGGCTCATGGGAAAATAAGTGAAC
ATGAAATTAAAAATTTTAGAAAAAAAATGGTGGAATGAATTTAGAGAGAAACTTTGGGAA
GCTATGTTATCTGAGCATAAAAATAATATAAATAATTGTAAAAATATTCCCCAAGAAGAA
TTACAAATTACTCAATGGATAAAAGAATGGCATGGAGAATTTTTGCTTGAAAGAGATAAT
AGATCAAAATTGCCAAAAAGTAAATGTAAAAATAATACATTATATGAAGCATGTGAGAA
GGAATGTATTGATCCATGTATGAAATATAGAGATTGGATTATTAGAAGTAAATTTGAATG
GCATACGTTATCGAAAGAATATGAAACTCAAAATGTTTCAAAGGAAAATGCGGAAAATT
ATTTAATCAAAATTTCAAAAAAACAAGAATGATGCTAAAGTAAGTTTATTATTGAGTAATT
GTGATGCTGAATATTCAAGATATTGTGATTGTAAACATACTACTACTCTCGTTAAAAGCGT
TGTAAATGGCAACGACAATACAATTAAGGAAAAGCGTGAACATATTGATTTATATGATTT
TTCTAAATTTGGATGTGATAAAAAATTCCGTTGATACAAACACAAAGGTGTGGGAATGTAA
AAAACCTTATATATTATCCACTAAAGATGTATGTGTACCTCCGAGGAGGCAAGAATTATG
TCTTGGAAACATTGATAGAATATACGATAAAAACCTATTAATGATAAAAGAGCATATTCT
TGCTATTGCAATATATGAATCAAGAATATTGAAACGAAAATATAAGAATAAAGATGATAA

AGAAGTTTGTAAAATCATAAATAAAACTTTCGCTGATATAAGAGATATTATAGGAGGTAC
TGATTATTGGAATGATTTGAGCAATAGAAAATTAGTAGGAAAAATTAACACAAATTCAAA
TTATGTTCACAGGAATAAACAAAATGATAAGCTTTTTCGTGATGAGTGGTGGAAAGTTAT
TAAAAAAGATGTATGGAATGTGATATCATGGGTATTCAAGGATAAAACTGTTTGTAAAGA
AGATGATATTGAAAATATACCACAATTCTTCAGATGGTTTAGTGAATGGGGTGATGATTA
TTGCCAGGATAAAACAAAAATG
>Equatorial Guinea (Bioko) 2019B19037C_MW691473.1
TATGTATGTATTCCTGATCGTAGAATCCAATTATGCATTGTTAATCTTAGCATTATTAAAA
CATATACAAAAGAGACCATGAAGGATCATTTCATTGAAGCCTCTAAAAAAGAATCTCAA
CTTTTGCTTAAAAAAAAATGATAACAAATATAATTCTAAATTTTGTAATGATTTGAAGAATA
GTTTTTTAGATTATGGACATCTTGCTATGGGAAATGATATGGATTTTGGAGGTTATTCAAC
TAAGGCAGAAAACAAAATTCAAGAAGTTTTTAAAGGGGCTCATGGGGAAATAAGTGAAC
ATGAAATTAAAAATTTTAGAAAAAAATGGTGGAATGAATTTAGAGAGAAACTTTGGGAA
GCTATGTTATCTGAGCATAAAAATAATATAAATAATTGTAAAAATATTCCCCAAGAAGAA
TTACAAATTACTCAATGGATAAAAGAATGGCATGGAGAATTTTTGCTTGAAAGAGATAAT
AGATCAAAATTGCCAAAAAGTAAATGTAAAAATAATACATTATATGAAGCATGTGAGAA
GGAATGTATTGATCCATGTATGAAATATAGAGATTGGATTATTAGAAGTAAATTTGAATG
GCATACGTTATCGAAAGAATATGAAACTCAAAAAGTTTCAAAGGAAAATGCGGAAAATT
ATTTAATCAAAATTTCAGAAAACAAGAATGATGCTAAAGTAAGTTTATTATTGAATAATT
GTGATGCTGAATATTCAAAATATTGTGATTGTAAACATACTACTACTCTCGTTAAAAGCGT
TTTAAATGGTAACGACAATACAATTAAGGAAAAGCGTGAACATATTGATTTAGATGATTT
TTCTAAATTTGGATGTGATAAAAATTCCGTTGATACAAACACAAAGGTGTGGGAATGTAA
AAAACCTTATATATTATCCACTAAAGATGTATGTGTACCTCCGAGGAGGCAAGAATTATG
TCTTGGAAACATTGATAGAATATACGATAAAAACCTATTAATGATAAAAGAGCATATTCT
TGCTATTGCAATATATGAATCAAGAATATTGAAACGAAAATATAAGAATAAAGATGATAA
AGAAGTTTGTAAAATCATAAATAAAACTTTCGCTGATATAAGAGATATTATAGGAGGTAC
TGATTATTGGAATGATTTGAGCAATAGAAAATTAGTAGGAAAAATTAACACAAATTCAAA
ATATGTTCACAGGAATAAAAAAAAATGATAAGCTTTTTCGTGATGAGTGGTGGAAAGTTAT
TAAAAAAGATGTATGGAATGTGATATCATGGGTATTCAAGGATAAAACTGTTTGTAAAGA
AGATGATATTGAAAATATACCACAATTCTTCAGATGGTTTAGTGAATGGGGTGATGATTA
TTGCCAGGATAAAACAAAAATG
>Equatorial Guinea (Bioko) 2019B19029C_MW691474.1
TATGTATGTATTCCTGATCGTAGAATCCAATTATGCATTGTTAATCTTAGCATTATTAAAA
CATATACAAAAGAGACCATGAAGGATCATTTCATTGAAGCCTCTAAAAAAGAATCTCAA
CTTTTGCTTAAAAAAAAATGATAACAAATATAATTCTAAATTTTGTAATGATTTGAAGAATA
GTTTTTTAGATTATGGACATCTTGCTATGGGAAATGATATGGATTTTGGAGGTTATTCAAC
TAAGGCAGAAAACAAAATTCAAGAAGTTTTTAAAGGGGCTCATGGGGAAATAAGTGAAC
ATGAAATTAAAAATTTTAGAAAAAAATGGTGGAATGAATTTAGAGAGAAACTTTGGGAA
GCTATGTTATCTGAGCATAAAAATAATATAAATAATTGTAAAAATATTCCCCAAGAAGAA
TTACAAATTACTCAATGGATAAAAGAATGGCATGGAGAATTTTTGCTTGAAAGAGATAAT
AGATCAAAATTGCCAAAAAGTAAATGTAAAAATAATACATTATATGAAGCATGTGAGAA
GGAATGTATTGATCCATGTATGAAATATAGAGATTGGATTATTAGAAGTAAATTTGAATG
GCATACGTTATCGAAAGAATATGAAACTCAAAAAGTTTCAAAGGAAAATGCGGAAAATT
ATTTAATCAAAATTTCAGAAAACAAGAATGATGCTAAAGTAAGTTTATTATTGAATAATT

GTGATGCTGAATATTCAAAATATTGTGATTGTAAACATACTACTACTCTCGTTAAAAGCGT
TTTAAATGGTAACGACAATACAATTAAGGAAAAGCGTGAACATATTGATTTAGATGATTT
TTCTAAATTTGGATGTGATAAAAATTCCGTTGATACAAACACAAAGGTGTGGGAATGTAA
AAAACCTTATATATTATCCACTAAAGATGTATGTGTACCTCCGAGGAGGCAAGAATTATG
TCTTGGAAACATTGATAGAATATACGATAAAAACCTATTAATGATAAAAGAGCATATTCT
TGCTATTGCAATATATGAATCAAGAATATTGAAACGAAAATATAAGAATAAAGATGATAA
AGAAGTTTGTAAAATCATAAATAAAACTTTCGCTGATATAAGAGATATTATAGGAGGTAC
TGATTATTGGAATGATTTGAGCAATAGAAAATTAGTAGGAAAAATTAACACAAATTCAAA
ATATGTTCACAGGAATAAAAAAAATGATAAGCTTTTTCGTGATGAGTGGTGGAAAGTTAT
TAAAAAAGATGTATGGAATGTGATATCATGGGTATTCAAGGATAAAACTGTTTGTAAAGA
AGATGATATTGAAAATATACCACAATTCTTCAGATGGTTTAGTGAATGGGGTGATGATTA
TTGCCAGGATAAAACAAAAATG
>Equatorial Guinea (Bioko) 2019B19026C_MW691475.1
TATGTATGTATTCCTGATCGTAGAATCCAATTATGCATTGTTAATCTTAGCATTATTAAAA
CATATACAAAAGAGACCATGAAGGATCATTTCATTGAAGCCTCTAAAAAAGAATCTCAA
CTTTTGCTTAAAAAAAATGATAACAAATATAATTCTAAATTTTGTAATGATTTGAAGAATA
GTTTTTTAGATTATGGACATCTTGCTATGGGAAATGATATGGATTTTGGAGGTTATTCAAC
TAAGGCAGAAAACAAAATTCAAGAAGTTTTTAAAGGGGCTCATGGGAAAATAAGTGAAC
ATGAAATTAAAAATTTTAGAAAAAAATGGTGGAATGAATTTAGAGAGAAACTTTGGGAA
GCTATGTTATCTGAGCATAAAAATAATATAAATAATTGTAAAAATATTCCCCAAGAAGAA
TTACAAATTACTCAATGGATAAAAGAATGGCATGGAGAATTTTTGCTTGAAAGAGATAAT
AGATCAAAATTGCCAAAAAGTAAATGTAAAAATAATACATTATATGAAGCATGTGAGAA
GGAATGTATTGATCCATGTATGAAATATAGAGATTGGATTATTAGAAGTAAATTTGAATG
GCATACGTTATCGAAAGAATATGAAACTCAAAATGTTTCAAAGGAAAATGCGGAAAATT
ATTTAATCAAAATTTCAAAAAACAAGAATGATGCTAAAGTAAGTTTATTATTGAATAATTG
TGATGCTGAATATTCAAAATATTGTGATTGTAAACATACTACTACTCTCGTTAAAAGCGTT
TTAAATGGTAACGACAATACAATTAAGGAAAAGCGTGAACATATTGATTTAGATGATTTT
TCTAAATTTGGATGTGATAAAAATTCCGTTGATACAAACACAAAGGTGTGGGAATGTAAA
AACCCTTATATATTATCCACTAAAGATGTATGTGTACCTCCGAGGAGGCAAGAATTATGT
CTTGGAAACATTGATAGAATATACGATAAAAACCTATTAATGATAAAAGAGCATATTCTT
GCTATTGCAATATATGAATCAAGAATATTGAAACGAAAATATAAGAATAAAGATGATAA
AGAAGTTTGTAAAATCATAAATAAAACTTTCGCTGATATAAGAGATATTATAGGAGGTAC
TGATTATTGGAATGATTTGAGCAATAGAAAATTAGTAGGAAAAATTAACACAAATTCAAA
TTATGTTCACAGGAATAAAAAAAATGATAAGCTTTTTCGTGATGAGTGGTGGAAAGTTAT
TAAAAAAGATGTATGGAATGTGATATCATGGGTATTCAAGGATAAAACTGTTTGTAAAGA
AGATGATATTGAAAATATACCACAATTCTTCAGATGGTTTAGTGAATGGGGTGATGATTA
TTGCCAGGATAAAACAAAAATG
>Equatorial Guinea (Bioko) 2019B19008C_MW691476.1
TATGTATGTATTCCTGATCGTAGAATCCAATTATGCATTGTTAATCTTAGCATTATTAAAA
CATATACAAAAGAGACCATGAAGGATCATTTCATTGAAGCCTCTAAAAAAGAATCTCAA
CTTTTGCTTAAAAAAAATGATAACAAATATAATTCTAAATTTTGTAATGATTTGAAGAATA
GTTTTTTAGATTATGGACATCTTGCTATGGGAAATGATATGGATTTTGGAGGTTATTCAAC
TAAGGCAGAAAACAAAATTCAAGAAGTTTTTAAAGGGGCTCATGGGAAAATAAGTGAAC
ATGAAATTAAAAATTTTAGAAAAAAATGGTGGAATGAATTTAGAGAGAAACTTTGGAAA

GCTATGTTATCTGAGCATAAAAATAATATAAATAATTGTAAAAATATTCCCCAAGAAGAA
TTACAAATTACTCAATGGATAAAAGAATGGCATGGAGAATTTTTGCTTGAAAGAGATAAT
AGATCAAAATTGCCAAAAAGTAAATGTAAAAATAATACATTATATGAAGCATGTGAGAA
GGAATGTATTGATCCATGTATGAAATATAGAGATTGGATTATTAGAAGTAAATTTGAATG
GCATACGTTATCGAAAGAATATGAAACTCAAAATGTTTCAAAGGAAAATGCGGAAAATT
ATTTAATCAAAATTTCAAAAAACAAGAATGATGCTAAAGTAAGTTTATTATTGAATAATTG
TGATGCTGAATATTCAAAATATTGTGATTGTAAACATACTACTACTCTCGTTAAAAGCGTT
TTAAATGGTAACGACAATACAATTAAGGAAAAGCGTGAACATATTGATTTAGATGATTTT
TCTAAATTTGGATGTGATAAAAATTCCGTTGATACAAACACAAAGGTGTGGGAATGTAAA
AACCCTTATATATTATCCACTAAAGATGTATGTGTACCTCCGAGGAGGCAAGAATTATGT
CTTGGAAACATTGATAGAATATACGATAAAAACCTATTAATGATAAAAGAGCATATTCTT
GCTATTGCAATATATGAATCAAGAATATTGAAACGAAAATATAAGAATAAAGATGATAA
AGAAGTTTGTAAAATCATAAATAAAACTTTCGCTGATATAAGAGATATTATAGGAGGTAC
TGATTATTGGAATGATTTGAGCAATAGAAAATTAGTAGGAAAAATTAACACAAATTCAAA
TTATGTTCACAGGAATAAAAAAAATGATAAGCTTTTTCGTGATGAGTGGTGGAAAGTTAT
TAAAAAAGATGTATGGAATGTGATATCATGGGTATTCAAGGATAAAACTGTTTGTAAAGA
AGATGATATTGAAAATATACCACAATTCTTCAGATGGTTTAGTGAATGGGGTGATGATTA
TTGCCAGGATAAAACAAAAATG
>Kenya C843_EBA-175DQ092125.1
TATGTATGTATTCCTGATCGTAGAATCCAATTATGCATTGTTAATCTTAGCATTATTAAAA
CATATACAAAAGAGACCATGAAGGATCATTTCATTGAAGCCTCTAAAAAAGAATCTCAA
CTTTTGCTTAAAAAAAAATGATAACAAATATAATTCTAAATTTTGTAATGATTTGAAGAATA
GTTTTTTAGATTATGGACATCTTGCTATGGGAAATGATATGGATTTTGGAGGTTATTCAAC
TAAGGCAGAAAACAAAATTCAAGAAGTTTTTAAAGGGGCTCATGGGAAAATAAGTGAAC
ATGAAATTAAAAATTTTAGAAAAGAATGGTGGAATGAATTTAGAGAGAAACTTTGGGAA
GCTATGTTATCTGAGCATAAAAATAATATAAATAATTGTAAAAATATTCCCCAAGAAGAA
TTACAAATTACTCAATGGATAAAAGAATGGCATGGAGAATTTTTGCTTGAAAGAGATAAT
AGATCAAAATTGCCAAAAAGTAAATGTAAAAATAATACATTATATGAAGCATGTGAGAA
GGAATGTATTGATCCATGTATGAAATATAGAGATTGGATTATTAGAAGTAAATTTGAATG
GCATACGTTATCGAAAGAATATGAAACTCAAAATGTTTCAAAGGAAAATGCGGAAAATT
ATTTAATCAAAATTTCAGAAAAAATGAATGATGCTAAAGTAAGTTTATTATTGAATAATTG
TGATGCTGAATATTCAAAATATTGTGATTGTAAACATACTACTACTCTCGTTAAAAGCGTT
TTAAATGGTAACGACAATACAATTAAGGAAAAGCGTGAACATATTGATTTAGATGATTTT
TCTAAATTTGGATGTGATAAAAATTCCGTTGATACAAACACAAAGGTGTGGGAATGTAAA
AAACCTTATAAATTATCCACTAAAGATGTATGTGTACCTCCGAGGAGGCAAGAATTATGT
CTTGGAAACATTGATAGAATATACGATAAAAACCTATTAATGATAAAAGAGCATATTCTT
GCTATTGCAATATATGAATCAAGAATATTGAAACGAAAATATAAGAATAAAGATGATAA
AGAAGTTTGTAAAATCATAAATAAAACTTTCGCTGATATAAGAGATATTATAGGAGGTAC
TGATTATTGGAATGATTTGAGCAATAGAAAATTAGTAGGAAAAATTAACACAAATTCAAA
TTATGTTCACAGGAATAAAGAAAATGATAAGCTTTTTCGTGATGCGTGGTGGAAAGTTAT
TAAAAAAGATGTATGGAATGTGATATCATGGGTATTCAAGGATAAAACTGTTTGTAAAGA
AGATGATATTGAAAATATACCACAATTCTTCAGATGGTTTAGTGAATGGGGTGATGATTA
TTGCCAGGATAAAACAAAAATG
>Kenya C619_EBA-175DQ092124.1

TATGTATGTATTCCTGATCGTAGAATCCAATTATGCATTGTTAATCTTAGCATTATTAAAA
CATATACAAAAGAGACCATGAAGGATCATTTCATTGAAGCCTCTAAAAAAGAATCTCAA
CTTTTGCTTAAAAAAAAATGATAACAAATATAATTCTAAATTTTGTAATGATTTGAAGAATA
GTTTTTTAGATTATGGACATCTTGCTATGGGAAATGATATGGATTTTGGAGGTTATTCAAC
TAAGGCAGAAAACAAAATTCAAGAAGTTTTTAAAGGGGCTCATGGGAAAATAAGTGAAC
ATGAAATTAAAAATTTTAGAAAAAAATGGTGGAATGAATTTAGAGAGAAACTTTGGGAA
GCTATGTTATCTGAGCATAAAAATAATATAAATAATTGTAAAAATATTCCCCAAGAAGAA
TTACAAATTACTCAATGGATAAAAGAATGGCATGGAGAATTTTTGCTTGAAAGAGATAAT
AGATCAAAATTGCCAAAAAGTAAATGTAAAAATAATACATTATATGAAGCATGTGAGAA
GGAATGTATTGATCCATGTATGAAATATAGAGATTGGATTATTAGAAGTAAATTTGAATG
GCATACGTTATCGAAAGAATATGAAACTCAAAATGTTTCAAAGGAAAATGCGGAAAATT
ATTTAATCAAAATTTCAGAAAAAATGAATGATGCTAAAGTAAGTTTATTATTGAATAATTG
TGATGCTGAATATTCAAAATATTGTGATTGTAAACATACTACTACTCTCGTTAAAAGCGTT
TTAAATGGTAACGACAATACAATTAAGGAAAAGCGTGAACATATTGATTTAGATGATTTT
TCTAAATTTGGATGTGATAAAAATTCCGTTGATACAAACACAAAGGTGTGGGAATGTAAA
AAACCTTATAAATTATCCACTAAAGATGTATGTGTACCTCCGAGGAGGCAAGAATTATGT
CTTGGAAACATTGATAGAATATACGATAAAAACCTATTAATGATAAAAGAGCATATTCTT
GCTATTGCAATATATGAATCAAGAATATTGAAACGAAAATATAAGAATAAAGATGATAA
AGAAGTTTGTAAAATCATAAATAAAACTTTCGCTGATATAAGAGATATTATAGGAGGTAC
TGATTATTGGAATGATTTGAGCAATAGAAAATTAGTAGGAAAAATTAACACAAATTCAAA
TTATGTTCACAGGAATAAAGAAAATGATAAGCTTTTTCGTGATGCGTGGTGGAAAGTTAT
TAAAAAAGATGTATGGAATGTGATATCATGGGTATTCAAGGATAAAACTGTTTGTAAAGA
AGATGATATTGAAAATATACCACAATTCTTCAGATGGTTTAGTGAATGGGGTGATGATTA
TTGCCAGGATAAAACAAAAATG
>Kenya C244_EBA-175DQ092123.1
TATGTATGTATTCCTGATCGTAGAATCCAATTATGCATTGTTAATCTTAGCATTATTAAAA
CATATACAAAAGAGACCATGAAGGATCATTTCATTGAAGCCTCTAAAAAAGAATCTCAA
CTTTTGCTTAAAAAAAAATGATAACAAATATAATTCTAAATTTTGTAATGATTTGAAGAATA
GTTTTTTAGATTATGGACATCTTGCTATGGGAAATGATATGGATTTTGGAGGTTATTCAAC
TAAGGCAGAAAACAAAATTCAAGAAGTTTTTAAAGGGGCTCATGGGAAAATAAGTGAAC
ATGAAATTAAAAATTTTAGAAAAAAATGGTGGAATGAATTTAGAGAGAAACTTTGGGAA
GCTATGTTATCTGAGCATAAAAATAATATAAATAATTGTAAAAATATTCCCCAAGAAGAA
TTACAAATTACTCAATGGATAAAAGAATGGCATGGAGAATTTTTGCTTGAAAGAGATAAT
AGATCAAAATTGCCAAAAAGTAAATGTAAAAATAATACATTATATGAAGCATGTGAGAA
GGAATGTATTGATCCATGTATGAAATATAGAGATTGGATTATTAGAAGTAAATTTGAATG
GCATACGTTATCGAAAGAATATGAAACTCAAAAAGTTTCAAAGGAAAATGCGGAAAATT
ATTTAATCAAAATTTCAGAAAAAATGAATGATGCTAAAGTAAGTTTATTATTGAATAATTG
TGATGCTGAATATTCAAAATATTGTGATTGTAAACATACTACTACTCTCGTTAAAAGCGTT
TTAAATGGTAACGACAATACAATTAAGGAAAAGCGTGAACATATTGATTTAGATGATTTT
TCTAAATTTGGATGTGATAAAAATTCCGTTGATACAAACACAAAGGTGTGGGAATGTAAA
AAACCTTATAAATTATCCACTAAAGATGTATGTGTACCTCCGAGGAGGCAAGAATTATGT
CTTGGAAACATTGATAGAATATACGATAAAAACCTATTAATGATAAAAGAGCATATTCTT
GCTATTGCAATATATGAATCAAGAATATTGAAACGAAAATATAAGAATAAAGATGATAA
AGAAGTTTGTAAAATCATAAATAAAACTTTCGCTGATATAAGAGATATTATAGGAGGTAC

TGATTATTGGAATGATTTGAGCAATAGAAAATTAGTAGGAAAAATTAACACAAATTCAAA
TTATGTTCACAGGAATAAACAAAATGATAAGCTTTTTCGTGATGAGTGGTGGAAAGTTAT
TAAAAAAGATGTATGGAATGTGATATCATGGGTATTCAAGGATAAAACTGTTTGTAAAGA
AGATGATATTGAAAATATACCACAATTCTTCAGATGGTTTAGTGAATGGGGTGATGATTA
TTGCCAGGATAAAACAAAAATG
>Kenya C754_EBA-175DQ092122.1
TATGTATGTATTCCTGATCGTAGAATCCAATTATGCATTGTTAATCTTAGCATTATTAAAA
CATATACAAAAGAGACCATGAAGGATCATTTCATTGAAGCCTCTAAAAAAGAATCTCAA
CTTTTGCTTAAAAAAAAATGATAACAAATATAATTCTAAATTTTGTAATGATTTGAAGAATA
GTTTTTTAGATTATGGACATCTTGCTATGGGAAATGATATGGATTTTGGAGGTTATTCAAC
TAAGGCAGAAAACAAAATTCAAGAAGTTTTTAAAGGGGCTCATGGGGAAATAAGTGAAC
ATGAAATTAAAAATTTTAGAAAAAAATGGTGGAATGAATTTAGAGAGAAACTTTGGGAA
GCTATGTTATCTGAGCATAAAAATAATATAAATAATTGTAAAAATATTCCCCAAGAAGAA
TTACAAATTACTCAATGGATAAAAGAATGGCATGGAGAATTTTTGCTTGAAAGAGATAAT
AGATCAAAATTGCCAAAAAGTAAATGTAAAAATAATACATTATATGAAGCATGTGAGAA
GGAATGTATTGATCCATGTATGAAATATAGAGATTGGATTATTAGAAGTAAATTTGAATG
GCATACGTTATCGAAAGAATATGAAACTCAAAAAGTTTCAAAGGAAAATGCGGAAAATT
ATTTAATCAAAATTTCAGAAAACAAGAATGATGCTAAAGTAAGTTTATTATTGAATAATT
GTGATGCTGAATATTCAAAATATTGTGATTGTAAACATACTACTACTCTCGTTAAAAGCGT
TTTAAATGGTAACGACAATACAATTAAGGAAAAGCGTGAACATATTGATTTAGATGATTT
TTCTAAATTTGGATGTGATAAAAATTCCGTTGATACAAACACAAAGGTGTGGGAATGTAA
AAAACCTTATATATTATCCACTAAAGATGTATGTGTACCTCCGAGGAGGCAAGAATTATG
TCTTGGAAACATTGATAGAATATACGATAAAAACCTATTAATGATAAAAGAGCATATTCT
TGCTATTGCAATATATGAATCAAGAATATTGAAACGAAAATATAAGAATAAAGATGATAA
AGAAGTTTGTAAAATCATAAATAAAACTTTCGCTGATATAAGAGATATTATAGGAGGTAC
TGATTATTGGAATGATTTGAGCAATAGAAAATTAGTAGGAAAAATTAACACAAATTCAAA
ATATGTTCACAGGAATAAAAAAAAATGATAAGCTTTTTCGTGATGAGTGGTGGAAAGTTAT
TAAAAAAGATGTATGGAATGTGATATCATGGGTATTCAAGGATAAAACTGTTTGTAAAGA
AGATGATATTGAAAATATACCACAATTCTTCAGATGGTTTAGTGAATGGGGTGATGATTA
TTGCCAGGATAAAACAAAAATG
>Kenya C725_EBA-175DQ092121.1
TATGTATGTATTCCTGATCGTAGAATCCAATTATGCATTGTTAATCTTAGCATTATTAAAA
CATATACAAAAGAGACCATGAAGGATCATTTCATTGAAGCCTCTAAAAAAGAATCTCAA
CTTTTGCTTAAAAAAAAATGATAACAAATATAATTCTAAATTTTGTAATGATTTGAAGAATA
GTTTTTTAGATTATGGACATCTTGCTATGGGAAATGATATGGATTTTGGAGGTTATTCAAC
TAAGGCAGAAAACAAAATTCAAGAAGTTTTTAAAGGGGCTCATGGGAAAATAAGTGAAC
ATGAAATTAAAAATTTTAGAAAAGAATGGTGGAATGAATTTAGAGAGAAACTTTGGGAA
GCTATGTTATCTGAGCATAAAAATAATATAAATAATTGTAAAAATATTCCCCAAGAAGAA
TTACAAATTACTCAATGGATAAAAGAATGGCATGGAGAATTTTTGCTTGAAAGAGATAAT
AGATCAAAATTGCCAAAAAGTAAATGTAAAAATAATACATTATATGAAGCATGTGAGAA
GGAATGTATTGATCCATGTATGAAATATAGAGATTGGATTATTAGAAGTAAATTTGAATG
GCATACGTTATCGAAAGAATATGAAACTCAAAATGTTTCAAAGGAAAATGCGGAAAATT
ATTTAATCAAAATTTCAGAAAACAAGAATGATGCTAAAGTAAGTTTATTATTGAATAATT
GTGATGCTGAATATTCAAAATATTGTGATTGTAAACATACTACTACTCTCGTTAAAAGCGT

TTTAAATGGTAACGACAATACAATTAAGGAAAAGCGTGAACATATTGATTTAGATGATTT
TTCTAAATTTGGATGTGATAAAAATTCCGTTGATACAAACACAAAGGTGTGGGAATGTAA
AAAACCTTATAAATTATCCACTAAAGATGTATGTGTACCTCCGAGGAGGCAAGAATTATG
TCTTGGAAACATTGATAGAATATACGATAAAAACCTATTAATGATAAAAGAGCATATTCT
TGCTATTGCAATATATGAATCAAGAATATTGAAACGAAAATATAAGAATAAAGATGATAA
AGAAGTTTGTAAAATCATAAATAAAACTTTCGCTGATATAAGAGATATTATAGGAGGTAC
TGATTATTGGAATGATTTGAGCAATAGAAAATTAGTAGGAAAAATTAACACAAATTCAAA
TTATGTTCACAGGAATAAACAAAATGATAAGCTTTTTCGTGATGAGTGGTGGAAAGTTAT
TAAAAAAGATGTATGGAATGTGATATCATGGGTATTCAAGGATAAAACTGTTTGTAAAGA
AGATGATATTGAAAATATACCACAATTCTTCAGATGGTTTAGTGAATGGGGTGATGATTA
TTGCCAGGATAAAACAAAAATG
>Kenya C568_EBA-175DQ092120.1
TATGTATGTATTCCTGATCGTAGAATCCAATTATGCATTGTTAATCTTAGCATTATTAAAA
CATATACAAAAGAGACCATGAAGGATCATTTCATTGAAGCCTCTAAAAAAGAATCTCAA
CTTTTGCTTAAAAAAAAATGATAACAAATATAATTCTAAATTTTGTAATGATTTGAAGAATA
GTTTTTTAGATTATGGACATCTTGCTATGGGAAATGATATGGATTTTGGAGGTTATTCAAC
TAAGGCAGAAAACAAAATTCAAGAAGTTTTTAAAGGGGCTCATGGGGAAATAAGTGAAC
ATAAAATTAAAAATTTTAGAAAAAAATGGTGGAATGAATTTAGAGAGAAACTTTGGGAA
GCTATGTTATCTGAGCATAAAAATAATATAAATAATTGTAAAAATATTCCCCAAGAAGAA
TTACAAATTACTCAATGGATAAAAGAATGGCATGGAGAATTTTTGCTTGAAAGAGATAAT
AGATCAAAATTGCCAAAAAGTAAATGTAAAAATAATACATTATATGAAGCATGTGAGAA
GGAATGTATTGATCCATGTATGAAATATAGAGATTGGATTATTAGAAGTAAATTTGAATG
GCATACGTTATCGAAAGAATATGAAACTCAAAAAGTTCCAAAGGAAAATGCGGAAAATT
ATTTAATCAAAATTTCAGAAAACAAGAATGATGCTAAAGTAAGTTTATTATTGAATAATT
GTGATGCTGAATATTCAAAATATTGTGATTGTAAACATACTACTACTCTCGTTAAAAGCGT
TTTAAATGGTAACGACAATACAATTAAGGAAAAGCGTGAACATATTGATTTAGATGATTT
TTCTAAATTTGGATGTGATAAAAATTCCGTTGATACAAACACAAAGGTGTGGGAATGTAA
AAAACCTTATATATTATCCACTAAAGATGTATGTGTACCTCCGAGGAGGCAAGAATTATG
TCTTGGAAACATTGATAGAATATACGATAAAAACCTATTAATGATAAAAGAGCATATTCT
TGCTATTGCAATATATGAATCAAGAATATTGAAACGAAAATATAAGAATAAAGATGATAA
AGAAGTTTGTAAAATCATAAATAAAACTTTCGCTGATATAAGAGATATTATAGGAGGTAC
TGATTATTGGAATGATTTGAGCAATAGAAAATTAGTAGGAAAAATTAACACAAATTCAAA
ATATGTTCACAGGAATAAAAAAAAATGATAAGCTTTTTCGTGATGAGTGGTGGAAAGTTAT
TAAAAAAGATGTATGGAATGTGATATCATGGGTATTCAAGGATAAAACTGTTTGTAAAGA
AGATGATATTGAAAATATACCACAATTCTTCAGATGGTTTAGTGAATGGGGTGATGATTA
TTGCCAGGATAAAACAAAAATG
>Kenya C323_EBA-175DQ092119.1
TATGTATGTATTCCTGATCGTAGAATCCAATTATGCATTGTTAATCTTAGCATTATTAAAA
CATATACAAAAGAGACCATGAAGGATCATTTCATTGAAGCCTCTAAAAAAGAATCTCAA
CTTTTGCTTAAAAAAAAATGATAACAAATATAATTCTAAATTTTGTAATGATTTGAAGAATA
GTTTTTTAGATTATGGACATCTTGCTATGGGAAATGATATGGATTTTGGAGGTTATTCAAC
TAAGGCAGAAAACAAAATTCAAGAAGTTTTTAAAGGGGCTCATGGGAAAATAAGTGAAC
ATGAAATTAAAAATTTTAGAAAAAAATGGTGGAATGAATTTAGAGAGAAACTTTGGGAA
GCTATGTTATCTGAGCATAAAAATAATATAAATAATTGTAAAAATATTCCCCAAGAAGAA

TTACAAATTACTCAATGGATAAAAGAATGGCATGGAGAATTTTTGCTTGAAAGAGATAAT
AGATCAAAATTGCCAAAAAGTAAATGTAAAAATAATACATTATATGAAGCATGTGAGAA
GGAATGTATTGATCCATGTATGAAATATAGAGATTGGATTATTAGAAGTAAATTTGAATG
GCATACGTTATCGAAAGAATATGAAACTCAAAATGTTTCAAAGGAAAATGCGGAAAATT
ATTTAATCAAAATTTCAGAAAAAATGAATGATGCTAAAGTAAGTTTATTATTGAATAATTG
TGATGCTGAATATTCAAAATATTGTGATTGTAAACATACTACTACTCTCGTTAAAAGCGTT
TTAAATGGTAACGACAATACAATTAAGGAAAAGCGTGAACATATTGATTTAGATGATTTT
TCTAAATTTGGATGTGATAAAAATTCCGTTGATACAAACACAAAGGTGTGGGAATGTAAA
AAACCTTATAAATTATCCACTAAAGATGTATGTGTACCTCCGAGGAGGCAAGAATTATGT
CTTGGAAACATTGATAGAATATACGATAAAAACCTATTAATGATAAAAGAGCATATTCTT
GCTATTGCAATATATGAATCAAGAATATTGAAACGAAAATATAAGAATAAAGATGATAA
AGAAGTTTGTAAAATCATAAATAAAACTTTCGCTGATATAAGAGATATTATAGGAGGTAC
TGATTATTGGAATGATTTGAGCAATAGAAAATTAGTAGGAAAAATTAACACAAATTCAAA
TTATGTTCACAGGAATAAACAAAATGATAAGCTTTTTCGTGATGAGTGGTGGAAAGTTAT
TAAAAAAGATGTATGGAATGTGATATCATGGGTATTCAAGGATAAAACTGTTTGTAAAGA
AGATGATATTGAAAATATACCACAATTCTTCAGATGGTTTAGTGAATGGGGTGATGATTA
TTGCCAGGATAAAACAAAAATG
>Kenya C309_EBA-175DQ092118.1
TATGTATGTATTCCTGATCGTAGAATCCAATTATGCATTGTTAATCTTAGCATTATTAAAA
CATATACAAAAGAGACCATGAAGGATCATTTCATTGAAGCCTCTAAAAAAGAATCTCAA
CTTTTGCTTAAAAAAAAATGATAACAAATATAATTCTAAATTTTGTAATGATTTGAAGAATA
GTTTTTTAGATTATGGACATCTTGCTATGGGAAATGATATGGATTTTGGAGGTTATTCAAC
TAAGGCAGAAAACAAAATTCAAGAAGTTTTTAAAGGGGCTCATGGGGAAATAAGTGAAC
ATGAAATTAAAAATTTTAGAAAAAAATGGTGGAATGAATTTAGAGAGAAACTTTGGGAA
GCTATGTTATCTGAGCATAAAAATAATATAAATAATTGTAAAAATATTCCCCAAGAAGAA
TTACAAATTACTCAATGGATAAAAGAATGGCATGGAGAATTTTTGCTTGAAAGAGATAAT
AGATCAAAATTGCCAAAAAGTAAATGTAAAAATAATACATTATATGAAGCATGTGAGAA
GGAATGTATTGATCCATGTATGAAATATAGAGATTGGATTATTAGAAGTAAATTTGAATG
GCATACGTTATCGAAAGAATATGAAACTCAAAAAGTTTCAAAGGAAAATGCGGAAAATT
ATTTAATCAAAATTTCAGAAAACAAGAATGATGCTAAAGTAAGTTTATTATTGAATAATT
GTGATGCTGAATATTCAAAATATTGTGATTGTAAACATACTACTACTCTCGTTAAAAGCGT
TTTAAATGGTAACGACAATACAATTAAGGAAAAGCGTGAACATATTGATTTAGATGATTT
TTCTAAATTTGGATGTGATAAAAATTCCGTTGATACAAACACAAAGGTGTGGGAATGTAA
AAAACCTTATATATTATCCACTAAAGATGTATGTGTACCTCCGAGGAGGCAAGAATTATG
TCTTGGAAACATTGATAGAATATACGATAAAAACCTATTAATGATAAAAGAGCATATTCT
TGCTATTGCAATATATGAATCAAGAATATTGAAACGAAAATATAAGAATAAAGATGATAA
AGAAGTTTGTAAAATCATAAATAAAACTTTCGCTGATATAAGAGATATTATAGGAGGTAC
TGATTATTGGAATGATTTGAGCAATAGAAAATTAGTAGGAAAAATTAACACAAATTCAAA
ATATGTTCACAGGAATAAAAAAAAATGATAAGCTTTTTCGTGATGAGTGGTGGAAAGTTAT
TAAAAAAGATGTATGGAATGTGATATCATGGGTATTCAAGGATAAAACTGTTTGTAAAGA
AGATGATATTGAAAATATACCACAATTCTTCAGATGGTTTAGTGAATGGGGTGATGATTA
TTGCCAGGATAAAACAAAAATG
>Kenya C182_EBA-175DQ092117.1
TATGTATGTATTCCTGATCGTAGAATCCAATTATGCATTGTTAATCTTAGCATTATTAAAA

CATATACAAAAGAGACCATGAAGGATCATTTCATTGAAGCCTCTAAAAAAGAATCTCAA
CTTTTGCTTAAAAAAAAATGATAACAAATATAATTCTAAATTTTGTAATGATTTGAAGAATA
GTTTTTTAGATTATGGACATCTTGCTATGGGAAATGATATGGATTTTGGAGGTTATTCAAC
TAAGGCAGAAAACAAAATTCAAGAAGTTTTTAAAGGGGCTCATGGGGAAATAAGTGAAC
ATGAAATTAAAAATTTTAGAAAAAAATGGTGGAATGAATTTAGAGAGAAACTTTGGGAA
GCTATGTTATCTGAGCATAAAAATAATATAAATAATTGTAAAAATATTCCCCAAGAAGAA
TTACAAATTACTCAATGGATAAAAGAATGGCATGGAGAATTTTTGCTTGAAAGAGATAAT
AGATCAAAATTGCCAAAAAGTAAATGTAAAAATAATACATTATATGAAGCATGTGAGAA
GGAATGTATTGATCCATGTATGAAATATAGAGATTGGATTATTAGAAGTAAATTTGAATG
GCATACGTTATCGAAAGAATATGAAACTCAAAAAGTTTCAAAGGAAAATGCGGAAAATT
ATTTAATCAAAATTTCAGAAAACAAGAATGATGCTAAAGTAAGTTTATTATTGAATAATT
GTGATGCTGAATATTCAAAATATTGTGATTGTAAACATACTACTACTCTCGTTAAAAGCGT
TTTAAATGGTAACGACAATACAATTAAGGAAAAGCGTGAACATATTGATTTAGATGATTT
TTCTAAATTTGGATGTGATAAAAATTCCGTTGATACAAACACAAAGGTGTGGGAATGTAA
AAAACCTTATATATTATCCACTAAAGATGTATGTGTACCTCCGAGGAGGCAAGAATTATG
TCTTGGAAACATTGATAGAATATACGATAAAAACCTATTAATGATAAAAGAGCATATTCT
TGCTATTGCAATATATGAATCAAGAATATTGAAACGAAAATATAAGAATAAAGATGATAA
AGAAGTTTGTAAAATCATAAATAAAACTTTCGCTGATATAAGAGATATTATAGGAGGTAC
TGATTATTGGAATGATTTGAGCAATAGAAAATTAGTAGGAAAAATTAACACAAATTCAAA
ATATGTTCACAGGAATAAAAAAAATGATAAGCTTTTTCGTGATGAGTGGTGGAAAGTTAT
TAAAAAAGATGTATGGAATGTGATATCATGGGTATTCAAGGATAAAACTGTTTGTAAAGA
AGATGATATTGAAAATATACCACAATTCTTCAGATGGTTTAGTGAATGGGGTGATGATTA
TTGCCAGGATAAAACAAAAATG
>Kenya C176_EBA-175DQ092116.1
TATGTATGTATTCCTGATCGTAGAATCCAATTATGCATTGTTAATCTTAGCATTATTAAAA
CATATACAAAAGAGACCATGAAGGATCATTTCATTGAAGCCTCTAAAAAAGAATCTCAA
CTTTTGCTTAAAAAAAAATGATAACAAATATAATTCTAAATTTTGTAATGATTTGAAGAATA
GTTTTTTAGATTATGGACATCTTGCTATGGGAAATGATATGGATTTTGGAGGTTATTCAAC
TAAGGCAGAAAACAAAATTCAAGAAGTTTTTAAAGGGGCTCATGGGGAAATAAGTGAAC
ATGAAATTAAAAATTTTAGAAAAAAATGGTGGAATGAATTTAGAGAGAAACTTTGGGAA
GCTATGTTATCTGAGCATAAAAATAATATAAATAATTGTAAAAATATTCCCCAAGAAGAA
TTACAAATTACTCAATGGATAAAAGAATGGCATGGAGAATTTTTGCTTGAAAGAGATAAT
AGATCAAAATTGCCAAAAAGTAAATGTAAAAATAATACATTATATGAAGCATGTGAGAA
GGAATGTATTGATCCATGTATGAAATATAGAGATTGGATTATTAGAAGTAAATTTGAATG
GCATACGTTATCGAAAGAATATGAAACTCAAAAAGTTTCAAAGGAAAATGCGGAAAATT
ATTTAATCAAAATTTCAGAAAACAAGAATGATGCTAAAGTAAGTTTATTATTGAATAATT
GTGATGCTGAATATTCAAAATATTGTGATTGTAAACATACTACTACTCTCGTTAAAAGCGT
TTTAAATGGTAACGACAATACAATTAAGGAAAAGCGTGAACATATTGATTTAGATGATTT
TTCTAAATTTGGATGTGATAAAAATTCCGTTGATACAAACACAAAGGTGTGGGAATGTAA
AAAACCTTATATATTATCCACTAAAGATGTATGTGTACCTCCGAGGAGGCAAGAATTATG
TCTTGGAAACATTGATAGAATATACGATAAAAACCTATTAATGATAAAAGAGCATATTCT
TGCTATTGCAATATATGAATCAAGAATATTGAAACGAAAATATAAGAATAAAGATGATAA
AGAAGTTTGTAAAATCATAAATAAAACTTTCGCTGATATAAGAGATATTATAGGAGGTAC
TGATTATTGGAATGATTTGAGCAATAGAAAATTAGTAGGAAAAATTAACACAAATTCAAA

ATATGTTCACAGGAATAAAAAAAAATGATAAGCTTTTTCGTGATGAGTGGTGGAAAGTTAT
TAAAAAAGATGTATGGAATGTGATATCATGGGTATTCAAGGATAAAACTGTTTGTAAAGA
AGATGATATTGAAAATATACCACAATTCTTCAGATGGTTTAGTGAATGGGGTGATGATTA
TTGCCAGGATAAAACAAAAATG
>Kenya C100_EBA-175DQ092115.1
TATGTATGTATTCCTGATCGTAGAATCCAATTATGCATTGTTAATCTTAGCATTATTAAAA
CATATACAAAAGAGACCATGAAGGATCATTTCATTGAAGCCTCTAAAAAAGAATCTCAA
CTTTTGCTTAAAAAAAAATGATAACAAATATAATTCTAAATTTTGTAATGATTTGAAGAATA
GTTTTTTAGATTATGGACATCTTGCTATGGGAAATGATATGGATTTTGGAGGTTATTCAAC
TAAGGCAGAAAACAAAATTCAAGAAGTTTTTAAAGGGGCTCATGGGGAAATAAGTGAAC
ATAAAATTAAAAATTTTAGAAAAAAAATGGTGGAATGAATTTAGAGAGAAACTTTGGGAA
GCTATGTTATCTGAGCATAAAAATAATATAAATAATTGTAAAAATATTCCCCAAGAAGAA
TTACAAATTACTCAATGGATAAAAGAATGGCATGGAGAATTTTTGCTTGAAAGAGATAAT
AGATCAAAATTGCCAAAAAGTAAATGTAAAAATAATACATTATATGAAGCATGTGAGAA
GGAATGTATTGATCCATGTATGAAATATAGAGATTGGATTATTAGAAGTAAATTTGAATG
GCATACGTTATCGAAAGAATATGAAACTCAAAAAGTTCCAAAGGAAAATGCGGAAAATT
ATTTAATCAAAATTTCAGAAAACAAGAATGATGCTAAAGTAAGTTTATTATTGAATAATT
GTGATGCTGAATATTCAAAATATTGTGATTGTAAACATACTACTACTCTCGTTAAAAGCGT
TTTAAATGGTAACGACAATACAATTAAGGAAAAGCGTGAACATATTGATTTAGATGATTT
TTCTAAATTTGGATGTGATAAAAATTCCGTTGATACAAACACAAAGGTGTGGGAATGTAA
AAAACCTTATAAATTATCCACTAAAGATGTATGTGTACCTCCGAGGAGGCAAGAATTATG
TCTTGGAAACATTGATAGAATATACGATAAAAACCTATTAATGATAAAAGAGCATATTCT
TGCTATTGCAATATATGAATCAAGAATATTGAAACGAAAATATAAGAATAAAGATGATAA
AGAAGTTTGTAAAATCATAAATAAAACTTTCGCTGATATAAGAGATATTATAGGAGGTAC
TGATTATTGGAATGATTTGAGCAATAGAAAATTAGTAGGAAAAATTAACACAAATTCAAA
TTATGTTCACAGGAATAAACAAAATGATAAGCTTTTTCGTGATGAGTGGTGGAAAGTTAT
TAAAAAAGATGTATGGAATGTGATATCATGGGTATTCAAGGATAAAACTGTTTGTAAAGA
AGATGATATTGAAAATATACCACAATTCTTCAGATGGTTTAGTGAATGGGGTGATGATTA
TTGCCAGGATAAAACAAAAATG
>Kenya C067_EBA-175DQ092114.1
TATGTATGTATTCCTGATCGTAGAATCCAATTATGCATTGTTAATCTTAGCATTATTAAAA
CATATACAAAAGAGACCATGAAGGATCATTTCATTGAAGCCTCTAAAAAAGAATCTCAA
CTTTTGCTTAAAAAAAAATGATAACAAATATAATTCTAAATTTTGTAATGATTTGAAGAATA
GTTTTTTAGATTATGGACATCTTGCTATGGGAAATGATATGGATTTTGGAGGTTATTCAAC
TAAGGCAGAAAACAAAATTCAAGAAGTTTTTAAAGGGGCTCATGGGGAAAAAAGTGAA
CATGAAATTAAAAATTTTAGAAAAAAAATGGTGGAATGAATTTAGAGAGAAACTTTGGGA
AGCTATGTTATCTGAGCATAAAAATAATATAAATAATTGTAAAAATATTCCCCAAGAAGA
ATTACAAATTACTCAATGGATAAAAGAATGGCATGGAGAATTTTTGCTTGAAAGAGATAA
TAGATCAAAATTGCCAAAAAGTAAATGTAAAAATAATACATTATATGAAGCATGTGAGA
AGGAATGTATTGATCCATGTATGAAATATAGAGATTGGATTATTAGAAGTAAATTTGAAT
GGCATACGTTATCGAAAGAATATGAAACTCAAAATGTTTCAAAGGAAAATGCGGAAAAT
TATTTAATCAAAATTTCAAAAAACAAGAATGATGCTAAAGTAAGTTTATTATTGAATAATT
GTGATGCTGAATATTCAAAATATTGTGATTGTAAACATACTACTACTCTCGTTAAAAGCGT
TTTAAATGGTAACGACAATACAATTAAGGAAAAGCGTGAACATATTGATTTAGATGATTT

TTCTAAATTTGGATGTGATAAAAATTCCGTTGATACAAACACAAAGGTGTGGGAATGTAA
AAACCCTTATATATTATCCACTAAAGATGTATGTGTACCTCCGAGGAGGCAAGAATTATG
TCTTGGAAACATTGATAGAATATACGATAAAAACCTATTAATGATAAAAGAGCATATTCT
TGCTATTGCAATATATGAATCAAGAATATTGAAACGAAAATATAAGAATAAAGATGATAA
AGAAGTTTGTAAAATCATAAATAAAACTTTCGCTGATATAAGAGATATTATAGGAGGTAC
TGATTATTGGAATGATTTGAGCAATAGAAAATTAGTAGGAAAAATTAACACAAATTCAAA
TTATGTTCACAGGAATAAAAAAAATGATAAGCTTTTTCGTGATGCGTGGTGGAAAGTTAT
TAAAAAAGATGTATGGAATGTGATATCATGGGTATTCAAGGATAAAACTGTTTGTAAAGA
AGATGATATTGAAAATATACCACAATTCTTCAGATGGTTTAGTGAATGGGGTGATGATTA
TTGCCAGGATAAAACAAAAATG
>Kenya C031_EBA-175DQ092113.1
TATGTATGTATTCCTGATCGTAGAATCCAATTATGCATTGTTAATCTTAGCATTATTAAAA
CATATACAAAAGAGACCATGAAGGATCATTTCATTGAAGCCTCTAAAAAAGAATCTCAA
CTTTTGCTTAAAAAAAATGATAACAAATATAATTCTAAATTTTGTAATGATTTGAAGAATA
GTTTTTTAGATTATGGACATCTTGCTATGGGAAATGATATGGATTTTGGAGGTTATTCAAC
TAAGGCAGAAAACAAAATTCAAGAAGTTTTTAAAGGGGCTCATGGGGAAATAAGTGAAC
ATGAAATTAAAAATTTTAGAAAAGAATGGTGGAATGAATTTAGAGAGAAACTTTGGGAA
GCTATGTTATCTGAGCATAAAAATAATATAAATAATTGTAAAAATATTCCCCAAGAAGAA
TTACAAATTACTCAATGGATAAAAGAATGGCATGGAGAATTTTTGCTTGAAAGAGATAAT
AGATCAAAATTGCCAAAAAGTAAATGTAAAAATAATACATTATATGAAGCATGTGAGAA
GGAATGTATTGATCCATGTATGAAATATAGAGATTGGATTATTAGAAGTAAATTTGAATG
GCATACGTTATCGAAAGAATATGAAACTCAAAAAGTTTCAAAGGAAAATGCGGAAAATT
ATTTAATCAAAATTTCAGAAAACAAGAATGATGCTAAAGTAAGTTTATTATTGAATAATT
GTGATGCTGAATATTCAAAATATTGTGATTGTAAACATACTACTACTCTCGTTAAAAGCGT
TTTAAATGGTAACGACAATACAATTAAGGAAAAGCGTGAACATATTGATTTAGATGATTT
TTCTAAATTTGGATGTGATAAAAATTCCGTTGATACAAACACAAAGGTGTGGGAATGTAA
AAAACCTTATAAATTATCCACTAAAGATGTATGTGTACCTCCGAGGAGGCAAGAATTATG
TCTTGGAAACATTGATAGAATATACGATAAAAACCTATTAATGATAAAAGAGCATATTCT
TGCTATTGCAATATATGAATCAAGAATATTGAAACGAAAATATAAGAATAAAGATGATAA
AGAAGTTTGTAAAATCATAAATAAAACTTTCGCTGATATAAGAGATATTATAGGAGGTAC
TGATTATTGGAATGATTTGAGCAATAGAAAATTAGTAGGAAAAATTAACACAAATTCAAA
ATATGTTCACAGGAATAAAAAAAATGATAAGCTTTTTCGTGATGAGTGGTGGAAAGTTAT
TAAAAAAGATGTATGGAATGTGATATCATGGGTATTCAAGGATAAAACTGTTTGTAAAGA
AGATGATATTGAAAATATACCACAATTCTTCAGATGGTTTAGTGAATGGGGTGATGATTA
TTGCCAGGATAAAACAAAAATG
>Kenya C457_EBA-175DQ092112.1
TATGTATGTATTCCTGATCGTAGAATCCAATTATGCATTGTTAATCTTAGCATTATTAAAA
CATATACAAAAGAGACCATGAAGGATCATTTCATTGAAGCCTCTAAAAAAGAATCTCAA
CTTTTGCTTAAAAAAAATGATAACAAATATAATTCTAAATTTTGTAATGATTTGAAGAATA
GTTTTTTAGATTATGGACATCTTGCTATGGGAAATGATATGGATTTTGGAGGTTATTCAAC
TAAGGCAGAAAACAAAATTCAAGAAGTTTTTAAAGGGGCTCATGGGAAAATAAGTGAAC
ATGAAATTAAAAATTTTAGAAAAAAAATGGTGGAATGAATTTAGAGAGAAACTTTGGGAA
GCTATGTTATCTGAGCATAAAAATAATATAAATAATTGTAAAAATATTCCCCAAGAAGAA
TTACAAATTACTCAATGGATAAAAGAATGGCATGGAGAATTTTTGCTTGAAAGAGATAAT

AGATCAAAATTGCCAAAAAGTAAATGTAAAAATAATACATTATATGAAGCATGTGAGAA
GGAATGTATTGATCCATGTATGAAATATAGAGATTGGATTATTAGAAGTAAATTTGAATG
GCATACGTTATCGAAAGAATATGAAACTCAAAATGTTTCAAAGGAAAATGCGGAAAATT
ATTTAATCAAAATTTCAAAAAACAAGAATGATGCTAAAGTAAGTTTATTATTGAATAATTG
TGATGCTGAATATTCAAAATATTGTGATTGTAAACATACTACTACTCTCGTTAAAAGCGTT
TTAAATGGTAACGACAATACAATTAAGGAAAAGCGTGAACATATTGATTTAGATGATTTT
TCTAAATTTGGATGTGATAAAAATTCCGTTGATACAAACACAAAGGTGTGGGAATGTAAA
AACCCTTATATATTATCCACTAAAGATGTATGTGTACCTCCGAGGAGGCAAGAATTATGT
CTTGGAAACATTGATAGAATATACGATAAAAACCTATTAATGATAAAAGAGCATATTCTT
GCTATTGCAATATATGAATCAAGAATATTGAAACGAAAATATAAGAATAAAGATGATAA
AGAAGTTTGTAAAATCATAAATAAAACTTTCGCTGATATAAGAGATATTATAGGAGGTAC
TGATTATTGGAATGATTTGAGCAATAGAAAATTAGTAGGAAAAATTAACACAAATTCAAA
TTATGTTCACAGGAATAAAAAAAATGATAAGCTTTTTCGTGATGAGTGGTGGAAAGTTAT
TAAAAAAGATGTATGGAATGTGATATCATGGGTATTCAAGGATAAAACTGTTTGTAAAGA
AGATGATATTGAAAATATACCACAATTCTTCAGATGGTTTAGTGAATGGGGTGATGATTA
TTGCCAGGATAAAACAAAAATG
>Kenya C444_EBA-175DQ092111.1
TATGTATGTATTCCTGATCGTAGAATCCAATTATGCATTGTTAATCTTAGCATTATTAAAA
CATATACAAAAGAGACCATGAAGGATCATTTCATTGAAGCCTCTAAAAAAGAATCTCAA
CTTTTGCTTAAAAAAAATGATAACAAATATAATTCTAAATTTTGTAATGATTTGAAGAATA
GTTTTTTAGATTATGGACATCTTGCTATGGGAAATGATATGGATTTTGGAGGTTATTCAAC
TAAGGCAGAAAACAAAATTCAAGAAGTTTTTAAAGGGGCTCATGGGGAAATAAGTGAAC
ATAAAATTAAAAATTTTAGAAAAGAATGGTGGAATGAATTTAGAGAGAAACTTTGGGAA
GCTATGTTATCTGAGCATAAAAATAATATAAATAATTGTAAAAATATTCCCCAAGAAGAA
TTACAAATTACTCAATGGATAAAAGAATGGCATGGAGAATTTTTGCTTGAAAGAGATAAT
AGATCAAAATTGCCAAAAAGTAAATGTAAAAATAATACATTATATGAAGCATGTGAGAA
GGAATGTATTGATCCATGTATGAAATATAGAGATTGGATTATTAGAAGTAAATTTGAATG
GCATACGTTATCGAAAGAATATGAAACTCAAAAAGTTTCAAAGGAAAATGCGGAAAATT
ATTTAATCAAAATTTCAGAAAACAAGAATGATGCTAAAGTAAGTTTATTATTGAATAATT
GTGATGCTGAATATTCAAAATATTGTGATTGTAAACATACTACTACTCTCGTTAAAAGCGT
TTTAAATGGTAACGACAATACAATTAAGGAAAAGCGTGAACATATTGATTTAGATGATTT
TTCTAAATTTGGATGTGATAAAAATTCCGTTGATACAAACACAAAGGTGTGGGAATGTAA
AAAACCTTATATATTATCCACTAAAGATGTATGTGTACCTCCGAGGAGGCAAGAATTATG
TCTTGGAAACATTGATAGAATATACGATAAAAACCTATTAATGATAAAAGAGCATATTCT
TGCTATTGCAATATATGAATCAAGAATATTGAAACGAAAATATAAGAATAAAGATGATAA
AGAAGTTTGTAAAATCATAAATAAAACTTTCGCTGATATAAGAGATATTATAGGAGGTAC
TGATTATTGGAATGATTTGAGCAATAGAAAATTAGTAGGAAAAATTAACACAAATTCAAA
ATATGTTCACAGGAATAAAAAAAATGATAAGCTTTTTCGTGATGAGTGGTGGAAAGTTAT
TAAAAAAGATGTATGGAATGTGATATCATGGGTATTCAAGGATAAAACTGTTTGTAAAGA
AGATGATATTGAAAATATACCACAATTCTTCAGATGGTTTAGTGAATGGGGTGATGATTA
TTGCCAGGATAAAACAAAAATG
>Kenya C443_EBA-175DQ092110.1
TATGTATGTATTCCTGATCGTAGAATCCAATTATGCATTGTTAATCTTAGCATTATTAAAA
CATATACAAAAGAGACCATGAAGGATCATTTCATTGAAGCCTCTAAAAAAGAATCTCAA

CTTTTGCTTAAAAAAAAATGATAACAAATATAATTCTAAATTTTGTAATGATTTGAAGAATA
GTTTTTTAGATTATGGACATCTTGCTATGGGAAATGATATGGATTTTGGAGGTTATTCAAC
TAAGGCAGAAAACAAAATTCAAGAAGTTTTTAAAGGGGCTCATGGGGAAATAAGTGAAC
ATAAAATTAAAAATTTTAGAAAAGAATGGTGGAATGAATTTAGAGAGAAACTTTGGGAA
GCTATGTTATCTGAGCATAAAAATAATATAAATAATTGTAAAAATATTCCCCAAGAAGAA
TTACAAATTACTCAATGGATAAAAGAATGGCATGGAGAATTTTTGCTTGAAAGAGATAAT
AGATCAAAATTGCCAAAAAGTAAATGTAAAAATAATACATTATATGAAGCATGTGAGAA
GGAATGTATTGATCCATGTATGAAATATAGAGATTGGATTATTAGAAGTAAATTTGAATG
GCATACGTTATCGAAAGAATATGAAACTCAAAAAGTTCCAAAGGAAAATGCGGAAAATT
ATTTAATCAAAATTTCAGAAAACAAGAATGATGCTAAAGTAAGTTTATTATTGAATAATT
GTGATGCTGAATATTCAAAATATTGTGATTGTAAACATACTACTACTCTCGTTAAAAGCGT
TTTAAATGGTAACGACAATACAATTAAGGAAAAGCGTGAACATATTGATTTAGATGATTT
TTCTAAATTTGGATGTGATAAAAATTCCGTTGATACAAACACAAAGGTGTGGGAATGTAA
AAAACCTTATAAATTATCCACTAAAGATGTATGTGTACCTCCGAGGAGGCAAGAATTATG
TCTTGGAAACATTGATAGAATATACGATAAAAACCTATTAATGATAAAAGAGCATATTCT
TGCTATTGCAATATATGAATCAAGAATATTGAAACGAAAATATAAGAATAAAGATGATAA
AGAAGTTTGTAAAATCATAAATAAAACTTTCGCTGATATAAGAGATATTATAGGAGGTAC
TGATTATTGGAATGATTTGAGCAATAGAAAATTAGTAGGAAAAATTAACACAAATTCAAA
TTATGTTCACAGGAATAAACAAAATGATAAGCTTTTTCGTGATGAGTGGTGGAAAGTTAT
TAAAAAAGATGTATGGAATGTGATATCATGGGTATTCAAGGATAAAACTGTTTGTAAAGA
AGATGATATTGAAAATATACCACAATTCTTCAGATGGTTTAGTGAATGGGGTGATGATTA
TTGCCAGGATAAAACAAAAATG
>Kenya C426_EBA-175DQ092109.1
TATGTATGTATTCCTGATCGTAGAATCCAATTATGCATTGTTAATCTTAGCATTATTAAAA
CATATACAAAAGAGACCATGAAGGATCATTTCATTGAAGCCTCTAAAAAAGAATCTCAA
CTTTTGCTTAAAAAAAAATGATAACAAATATAATTCTAAATTTTGTAATGATTTGAAGAATA
GTTTTTTAGATTATGGACATCTTGCTATGGGAAATGATATGGATTTTGGAGGTTATTCAAC
TAAGGCAGAAAACAAAATTCAAGAAGTTTTTAAAGGGGCTCATGGGGAAATAAGTGAAC
ATAAAATTAAAAATTTTAGAAAAGAATGGTGGAATGAATTTAGAGAGAAACTTTGGGAA
GCTATGTTATCTGAGCATAAAAATAATATAAATAATTGTAAAAATATTCCCCAAGAAGAA
TTACAAATTACTCAATGGATAAAAGAATGGCATGGAGAATTTTTGCTTGAAAGAGATAAT
AGATCAAAATTGCCAAAAAGTAAATGTAAAAATAATACATTATATGAAGCATGTGAGAA
GGAATGTATTGATCCATGTATGAAATATAGAGATTGGATTATTAGAAGTAAATTTGAATG
GCATACGTTATCGAAAGAATATGAAACTCAAAAAGTTCCAAAGGAAAATGCGGAAAATT
ATTTAATCAAAATTTCAGAAAACAAGAATGATGCTAAAGTAAGTTTATTATTGAATAATT
GTGATGCTGAATATTCAAAATATTGTGATTGTAAACATACTACTACTCTCGTTAAAAGCGT
TTTAAATGGTAACGACAATACAATTAAGGAAAAGCGTGAACATATTGATTTAGATGATTT
TTCTAAATTTGGATGTGATAAAAATTCCGTTGATACAAACACAAAGGTGTGGGAATGTAA
AAAACCTTATAAATTATCCACTAAAGATGTATGTGTACCTCCGAGGAGGCAAGAATTATG
TCTTGGAAACATTGATAGAATATACGATAAAAACCTATTAATGATAAAAGAGCATATTCT
TGCTATTGCAATATATGAATCAAGAATATTGAAACGAAAATATAAGAATAAAGATGATAA
AGAAGTTTGTAAAATCATAAATAAAACTTTCGCTGATATAAGAGATATTATAGGAGGTAC
TGATTATTGGAATGATTTGAGCAATAGAAAATTAGTAGGAAAAATTAACACAAATTCAAA
TTATGTTCACAGGAATAAACAAAATGATAAGCTTTTTCGTGATGAGTGGTGGAAAGTTAT

TAAAAAAGATGTATGGAATGTGATATCATGGGTATTCAAGGATAAAACTGTTTGTAAAGA
AGATGATATTGAAAATATACCACAATTCTTCAGATGGTTTAGTGAATGGGGTGATGATTA
TTGCCAGGATAAAACAAAAATG
>Kenya C425_EBA-175DQ092108.1
TATGTATGTATTCCTGATCGTAGAATCCAATTATGCATTGTTAATCTTAGCATTATTAAAA
CATATACAAAAGAGACCATGAAGGATCATTTCATTGAAGCCTCTAAAAAAGAATCTCAA
CTTTTGCTTAAAAAAAATGATAACAAATATAATTCTAAATTTTGTAATGATTTGAAGAATA
GTTTTTTAGATTATGGACATCTTGCTATGGGAAATGATATGGATTTTGGAGGTTATTCAAC
TAAGGCAGAAAACAAAATTCAAGAAGTTTTTAAAGGGGCTCATGGGGAAATAAGTGAAC
ATGAAATTAAAAATTTTAGAAAAAAATGGTGGAATGAATTTAGAGAGAAACTTTGGGAA
GCTATGTTATCTGAGCATAAAAATAATATAAATAATTGTAAAAATATTCCCCAAGAAGAA
TTACAAATTACTCAATGGATAAAAGAATGGCATGGAGAATTTTTGCTTGAAAGAGATAAT
AGATCAAAATTGCCAAAAAGTAAATGTAAAAATAATACATTATATGAAGCATGTGAGAA
GGAATGTATTGATCCATGTATGAAATATAGAGATTGGATTATTAGAAGTAAATTTGAATG
GCATACGTTATCGAAAGAATATGAAACTCAAAAAGTTTCAAAGGAAAATGCGGAAAATT
ATTTAATCAAAATTTCAGAAAACAAGAATGATGCTAAAGTAAGTTTATTATTGAATAATT
GTGATGCTGAATATTCAAAATATTGTGATTGTAAACATACTACTACTCTCGTTAAAAGCGT
TTTAAATGGTAACGACAATACAATTAAGGAAAAGCGTGAACATATTGATTTAGATGATTT
TTCTAAATTTGGATGTGATAAAAATTCCGTTGATACAAACACAAAGGTGTGGGAATGTAA
AAAACCTTATATATTATCCACTAAAGATGTATGTGTACCTCCGAGGAGGCAAGAATTATG
TCTTGGAAACATTGATAGAATATACGATAAAAACCTATTAATGATAAAAGAGCATATTCT
TGCTATTGCAATATATGAATCAAGAATATTGAAACGAAAATATAAGAATAAAGATGATAA
AGAAGTTTGTAAAATCATAAATAAAACTTTCGCTGATATAAGAGATATTATAGGAGGTAC
TGATTATTGGAATGATTTGAGCAATAGAAAATTAGTAGGAAAAATTAACACAAATTCAAA
ATATGTTCACAGGAATAAAAAAAATGATAAGCTTTTTCGTGATGAGTGGTGGAAAGTTAT
TAAAAAAGATGTATGGAATGTGATATCATGGGTATTCAAGGATAAAACTGTTTGTAAAGA
AGATGATATTGAAAATATACCACAATTCTTCAGATGGTTTAGTGAATGGGGTGATGATTA
TTGCCAGGATAAAACAAAAATG
>Kenya C417_EBA-175DQ092107.1
TATGTATGTATTCCTGATCGTAGAATCCAATTATGCATTGTTAATCTTAGCATTATTAAAA
CATATACAAAAGAGACCATGAAGGATCATTTCATTGAAGCCTCTAAAAAAGAATCTCAA
CTTTTGCTTAAAAAAAATGATAACAAATATAATTCTAAATTTTGTAATGATTTGAAGAATA
GTTTTTTAGATTATGGACATCTTGCTATGGGAAATGATATGGATTTTGGAGGTTATTCAAC
TAAGGCAGAAAACAAAATTCAAGAAGTTTTTAAAGGGGCTCATGGGAAAATAAGTGAAC
ATGAAATTAAAAATTTTAGAAAAGAATGGTGGAATGAATTTAGAGAGAAACTTTGGGAA
GCTATGTTATCTGAGCATAAAAATAATATAAATAATTGTAAAAATATTCCCCAAGAAGAA
TTACAAATTACTCAATGGATAAAAGAATGGCATGGAGAATTTTTGCTTGAAAGAGATAAT
AGATCAAAATTGCCAAAAAGTAAATGTAAAAATAATACATTATATGAAGCATGTGAGAA
GGAATGTATTGATCCATGTATGAAATATAGAGATTGGATTATTAGAAGTAAATTTGAATG
GCATACGTTATCGAAAGAATATGAAACTCAAAATGTTTCAAAGGAAAATGCGGAAAATT
ATTTAATCAAAATTTCAAAAAACAAGAATGATGCTAAAGTAAGTTTATTATTGAATAATTG
TGATGCTGAATATTCAAAATATTGTGATTGTAAACATACTACTACTCTCGTTAAAAGCGTT
TTAAATGGTAACGACAATACAATTAAGGAAAAGCGTGAACATATTGATTTAGATGATTTT
TCTAAATTTGGATGTGATAAAAATTCCGTTGATACAAACACAAAGGTGTGGGAATGTAAA

AAACCTTATAAATTATCCACTAAAGATGTATGTGTACCTCCGAGGAGGCAAGAATTATGT
CTTGGAAACATTGATAGAATATACGATAAAAACCTATTAATGATAAAAGAGCATATTCTT
GCTATTGCAATATATGAATCAAGAATATTGAAACGAAAATATAAGAATAAAGATGATAA
AGAAGTTTGTAAAATCATAAATAAAACTTTCGCTGATATAAGAGATATTATAGGAGGTAC
TGATTATTGGAATGATTTGAGCAATAGAAAATTAGTAGGAAAAATTAACACAAATTCAAA
TTATGTTCACAGGAATAAAGAAAATGATAAGCTTTTTCGTGATGCGTGGTGGAAAGTTAT
TAAAAAAGATGTATGGAATGTGATATCATGGGTATTCAAGGATAAAACTGTTTGTAAAGA
AGATGATATTGAAAATATACCACAATTCTTCAGATGGTTTAGTGAATGGGGTGATGATTA
TTGCCAGGATAAAACAAAAATG
>Kenya C405_EBA-175DQ092106.1
TATGTATGTATTCCTGATCGTAGAATCCAATTATGCATTGTTAATCTTAGCATTATTAAAA
CATATACAAAAGAGACCATGAAGGATCATTTCATTGAAGCCTCTAAAAAAGAATCTCAA
CTTTTGCTTAAAAAAAAATGATAACAAATATAATTCTAAATTTTGTAATGATTTGAAGAATA
GTTTTTTAGATTATGGACATCTTGCTATGGGAAATGATATGGATTTTGGAGGTTATTCAAC
TAAGGCAGAAAACAAAATTCAAGAAGTTTTTAAAGGGGCTCATGGGGAAAAAAGTGAA
CATGAAATTAAAAATTTTAGAAAAAAATGGTGGAATGAATTTAGAGAGAAACTTTGGGA
AGCTATGTTATCTGAGCATAAAAATAATATAAATAATTGTAAAAATATTCCCCAAGAAGA
ATTACAAATTACTCAATGGATAAAAGAATGGCATGGAGAATTTTTGCTTGAAAGAGATAA
TAGATCAAAATTGCCAAAAAGTAAATGTAAAAATAATACATTATATGAAGCATGTGAGA
AGGAATGTATTGATCCATGTATGAAATATAGAGATTGGATTATTAGAAGTAAATTTGAAT
GGCATACGTTATCGAAAGAATATGAAACTCAAAATGTTTCAAAGGAAAATGCGGAAAAT
TATTTAATCAAAATTTCAAAAAACAAGAATGATGCTAAAGTAAGTTTATTATTGAATAATT
GTGATGCTGAATATTCAAAATATTGTGATTGTAAACATACTACTACTCTCGTTAAAAGCGT
TTTAAATGGTAACGACAATACAATTAAGGAAAAGCGTGAACATATTGATTTAGATGATTT
TTCTAAATTTGGATGTGATAAAAATTCCGTTGATACAAACACAAAGGTGTGGGAATGTAA
AAACCCTTATATATTATCCACTAAAGATGTATGTGTACCTCCGAGGAGGCAAGAATTATG
TCTTGGAAACATTGATAGAATATACGATAAAAACCTATTAATGATAAAAGAGCATATTCT
TGCTATTGCAATATATGAATCAAGAATATTGAAACGAAAATATAAGAATAAAGATGATAA
AGAAGTTTGTAAAATCATAAATAAAACTTTCGCTGATATAAGAGATATTATAGGAGGTAC
TGATTATTGGAATGATTTGAGCAATAGAAAATTAGTAGGAAAAATTAACACAAATTCAAA
TTATGTTCACAGGAATAAAAAAAAATGATAAGCTTTTTCGTGATGCGTGGTGGAAAGTTAT
TAAAAAAGATGTATGGAATGTGATATCATGGGTATTCAAGGATAAAACTGTTTGTAAAGA
AGATGATATTGAAAATATACCACAATTCTTCAGATGGTTTAGTGAATGGGGTGATGATTA
TTGCCAGGATAAAACAAAAATG
>Kenya C404_EBA-175DQ092105.1
TATGTATGTATTCCTGATCGTAGAATCCAATTATGCATTGTTAATCTTAGCATTATTAAAA
CATATACAAAAGAGACCATGAAGGATCATTTCATTGAAGCCTCTAAAAAAGAATCTCAA
CTTTTGCTTAAAAAAAAATGATAACAAATATAATTCTAAATTTTGTAATGATTTGAAGAATA
GTTTTTTAGATTATGGACATCTTGCTATGGGAAATGATATGGATTTTGGAGGTTATTCAAC
TAAGGCAGAAAACAAAATTCAAGAAGTTTTTAAAGGGGCTCATGGGGAAATAAGTGAAC
ATGAAATTAAAAATTTTAGAAAAAAATGGTGGAATGAATTTAGAGAGAAACTTTGGGAA
GCTATGTTATCTGAGCATAAAAATAATATAAATAATTGTAAAAATATTCCCCAAGAAGAA
TTACAAATTACTCAATGGATAAAAGAATGGCATGGAGAATTTTTGCTTGAAAGAGATAAT
AGATCAAAATTGCCAAAAAGTAAATGTAAAAATAATACATTATATGAAGCATGTGAGAA

GGAATGTATTGATCCATGTATGAAATATAGAGATTGGATTATTAGAAGTAAATTTGAATG
GCATACGTTATCGAAAGAATATGAAACTCAAAAAGTTTCAAAGGAAAATGCGGAAAATT
ATTTAATCAAAATTTCAGAAAACAAGAATGATGCTAAAGTAAGTTTATTATTGAATAATT
GTGATGCTGAATATTCAAAATATTGTGATTGTAAACATACTACTACTCTCGTTAAAAGCGT
TTTAAATGGTAACGACAATACAATTAAGGAAAAGCGTGAACATATTGATTTAGATGATTT
TTCTAAATTTGGATGTGATAAAAATTCCGTTGATACAAACACAAAGGTGTGGGAATGTAA
AAAACCTTATATATTATCCACTAAAGATGTATGTGTACCTCCGAGGAGGCAAGAATTATG
TCTTGGAAACATTGATAGAATATACGATAAAAACCTATTAATGATAAAAGAGCATATTCT
TGCTATTGCAATATATGAATCAAGAATATTGAAACGAAAATATAAGAATAAAGATGATAA
AGAAGTTTGTAAAATCATAAATAAAACTTTCGCTGATATAAGAGATATTATAGGAGGTAC
TGATTATTGGAATGATTTGAGCAATAGAAAATTAGTAGGAAAAATTAACACAAATTCAAA
ATATGTTCACAGGAATAAAAAAAATGATAAGCTTTTTCGTGATGAGTGGTGGAAAGTTAT
TAAAAAAGATGTATGGAATGTGATATCATGGGTATTCAAGGATAAAACTGTTTGTAAAGA
AGATGATATTGAAAATATACCACAATTCTTCAGATGGTTTAGTGAATGGGGTGATGATTA
TTGCCAGGATAAAACAAAAATG
>Kenya C375_EBA-175DQ092104.1
TATGTATGTATTCCTGATCGTAGAATCCAATTATGCATTGTTAATCTTAGCATTATTAAAA
CATATACAAAAGAGACCATGAAGGATCATTTCATTGAAGCCTCTAAAAAAGAATCTCAA
CTTTTGCTTAAAAAAAATGATAACAAATATAATTCTAAATTTTGTAATGATTTGAAGAATA
GTTTTTTAGATTATGGACATCTTGCTATGGGAAATGATATGGATTTTGGAGGTTATTCAAC
TAAGGCAGAAAACAAAATTCAAGAAGTTTTTAAAGGGGCTCATGGGGAAATAAGTGAAC
ATGAAATTAAAAATTTTAGAAAAAAATGGTGGAATGAATTTAGAGAGAAACTTTGGGAA
GCTATGTTATCTGAGCATAAAAATAATATAAATAATTGTAAAAATATTCCCCAAGAAGAA
TTACAAATTACTCAATGGATAAAAGAATGGCATGGAGAATTTTTGCTTGAAAGAGATAAT
AGATCAAAATTGCCAAAAAGTAAATGTAAAAATAATACATTATATGAAGCATGTGAGAA
GGAATGTATTGATCCATGTATGAAATATAGAGATTGGATTATTAGAAGTAAATTTGAATG
GCATACGTTATCGAAAGAATATGAAACTCAAAAAGTTCCAAAGGAAAATGCGGAAAATT
ATTTAATCAAAATTTCAGAAAACAAGAATGATGCTAAAGTAAGTTTATTATTGAATAATT
GTGATGCTGAATATTCAAAATATTGTGATTGTAAACATACTACTACTCTCGTTAAAAGCGT
TTTAAATGGTAACGACAATACAATTAAGGAAAAGCGTGAACATATTGATTTAGATGATTT
TTCTAAATTTGGATGTGATAAAAATTCCGTTGATACAAACACAAAGGTGTGGGAATGTAA
AAAACCTTATAAATTATCCACTAAAGATGTATGTGTACCTCCGAGGAGGCAAGAATTATG
TCTTGGAAACATTGATAGAATATACGATAAAAACCTATTAATGATAAAAGAGCATATTCT
TGCTATTGCAATATATGAATCAAGAATATTGAAACGAAAATATAAGAATAAAGATGATAA
AGAAGTTTGTAAAATCATAAATAAAACTTTCGCTGATATAAGAGATATTATAGGAGGTAC
TGATTATTGGAATGATTTGAGCAATAGAAAATTAGTAGGAAAAATTAACACAAATTCAAA
TTATGTTCACAGGAATAAACAAAATGATAAGCTTTTTCGTGATGAGTGGTGGAAAGTTAT
TAAAAAAGATGTATGGAATGTGATATCATGGGTATTCAAGGATAAAACTGTTTGTAAAGA
AGATGATATTGAAAATATACCACAATTCTTCAGATGGTTTAGTGAATGGGGTGATGATTA
TTGCCAGGATAAAACAAAAATG
>Kenya C372_EBA-175DQ092103.1
TATGTATGTATTCCTGATCGTAGAATCCAATTATGCATTGTTAATCTTAGCATTATTAAAA
CATATACAAAAGAGACCATGAAGGATCATTTCATTGAAGCCTCTAAAAAAGAATCTCAA
CTTTTGCTTAAAAAAAATGATAACAAATATAATTCTAAATTTTGTAATGATTTGAAGAATA

GTTTTTTAGATTATGGACATCTTGCTATGGGAAATGATATGGATTTTGGAGGTTATTCAAC
TAAGGCAGAAAACAAAATTCAAGAAGTTTTTAAAGGGGCTCATGGGGAAATAAGTGAAC
ATAAAATTAAAAATTTTAGAAAAGAATGGTGGAATGAATTTAGAGAGAAACTTTGGGAA
GCTATGTTATCTGAGCATAAAAATAATATAAATAATTGTAAAAATATTCCCCAAGAAGAA
TTACAAATTACTCAATGGATAAAAGAATGGCATGGAGAATTTTTGCTTGAAAGAGATAAT
AGATCAAAATTGCCAAAAAGTAAATGTAAAAATAATACATTATATGAAGCATGTGAGAA
GGAATGTATTGATCCATGTATGAAATATAGAGATTGGATTATTAGAAGTAAATTTGAATG
GCATACGTTATCGAAAGAATATGAAACTCAAAAAGTTCCAAAGGAAAATGCGGAAAATT
ATTTAATCAAAATTTCAGAAAACAAGAATGATGCTAAAGTAAGTTTATTATTGAATAATT
GTGATGCTGAATATTCAAAATATTGTGATTGTAAACATACTACTACTCTCGTTAAAAGCGT
TTTAAATGGTAACGACAATACAATTAAGGAAAAGCGTGAACATATTGATTTAGATGATTT
TTCTAAATTTGGATGTGATAAAAATTCCGTTGATACAAACACAAAGGTGTGGGAATGTAA
AAAACCTTATAAATTATCCACTAAAGATGTATGTGTACCTCCGAGGAGGCAAGAATTATG
TCTTGGAAACATTGATAGAATATACGATAAAAACCTATTAATGATAAAAGAGCATATTCT
TGCTATTGCAATATATGAATCAAGAATATTGAAACGAAAATATAAGAATAAAGATGATAA
AGAAGTTTGTAAAATCATAAATAAAACTTTCGCTGATATAAGAGATATTATAGGAGGTAC
TGATTATTGGAATGATTTGAGCAATAGAAAATTAGTAGGAAAAATTAACACAAATTCAAA
TTATGTTCACAGGAATAAACAAAATGATAAGCTTTTTCGTGATGAGTGGTGGAAAGTTAT
TAAAAAAGATGTATGGAATGTGATATCATGGGTATTCAAGGATAAAACTGTTTGTAAAGA
AGATGATATTGAAAATATACCACAATTCTTCAGATGGTTTAGTGAATGGGGTGATGATTA
TTGCCAGGATAAAACAAAAATG
>Kenya C346_EBA-175DQ092102.1
TATGTATGTATTCCTGATCGTAGAATCCAATTATGCATTGTTAATCTTAGCATTATTAAAA
CATATACAAAAGAGACCATGAAGGATCATTTCATTGAAGCCTCTAAAAAAGAATCTCAA
CTTTTGCTTAAAAAAAAATGATAACAAATATAATTCTAAATTTTGTAATGATTTGAAGAATA
GTTTTTTAGATTATGGACATCTTGCTATGGGAAATGATATGGATTTTGGAGGTTATTCAAC
TAAGGCAGAAAACAAAATTCAAGAAGTTTTTAAAGGGGCTCATGGGGAAATAAGTGAAC
ATGAAATTAAAAATTTTAGAAAAAAAATGGTGGAATGAATTTAGAGAGAAACTTTGGGAA
GCTATGTTATCTGAGCATAAAAATAATATAAATAATTGTAAAAATATTCCCCAAGAAGAA
TTACAAATTACTCAATGGATAAAAGAATGGCATGGAGAATTTTTGCTTGAAAGAGATAAT
AGATCAAAATTGCCAAAAAGTAAATGTAAAAATAATACATTATATGAAGCATGTGAGAA
GGAATGTATTGATCCATGTATGAAATATAGAGATTGGATTATTAGAAGTAAATTTGAATG
GCATACGTTATCGAAAGAATATGAAACTCAAAAAGTTCCAAAGGAAAATGCGGAAAATT
ATTTAATCAAAATTTCAGAAAACAAGAATGATGCTAAAGTAAGTTTATTATTGAATAATT
GTGATGCTGAATATTCAAAATATTGTGATTGTAAACATACTACTACTCTCGTTAAAAGCGT
TTTAAATGGTAACGACAATACAATTAAGGAAAAGCGTGAACATATTGATTTAGATGATTT
TTCTAAATTTGGATGTGATAAAAATTCCGTTGATACAAACACAAAGGTGTGGGAATGTAA
AAAACCTTATATATTATCCACTAAAGATGTATGTGTACCTCCGAGGAGGCAAGAATTATG
TCTTGGAAACATTGATAGAATATACGATAAAAACCTATTAATGATAAAAGAGCATATTCT
TGCTATTGCAATATATGAATCAAGAATATTGAAACGAAAATATAAGAATAAAGATGATAA
AGAAGTTTGTAAAATCATAAATAAAACTTTCGCTGATATAAGAGATATTATAGGAGGTAC
TGATTATTGGAATGATTTGAGCAATAGAAAATTAGTAGGAAAAATTAACACAAATTCAAA
TTATGTTCACAGGAATAAAGAAAATGATAAGCTTTTTCGTGATGAGTGGTGGAAAGTTAT
TAAAAAAGATGTATGGAATGTGATATCATGGGTATTCAAGGATAAAACTGTTTGTAAAGA

AGATGATATTGAAAATATACCACAATTCTTCAGATGGTTTAGTGAATGGGGTGATGATTA
TTGCCAGGATAAAACAAAAATG
>Kenya C346_EBA-175DQ092101.1
TATGTATGTATTCCTGATCGTAGAATCCAATTATGCATTGTTAATCTTAGCATTATTAAAA
CATATACAAAAGAGACCATGAAGGATCATTTCATTGAAGCCTCTAAAAAAGAATCTCAA
CTTTTGCTTAAAAAAAATGATAACAAATATAATTCTAAATTTTGTAATGATTTGAAGAATA
GTTTTTTAGATTATGGACATCTTGCTATGGGAAATGATATGGATTTTGGAGGTTATTCAAC
TAAGGCAGAAAACAAAATTCAAGAAGTTTTTAAAGGGGCTCATGGGGAAATAAGTGAAC
ATAAAATTAAAAATTTTAGAAAAGAATGGTGGAATGAATTTAGAGAGAAACTTTGGGAA
GCTATGTTATCTGAGCATAAAAATAATATAAATAATTGTAAAAATATTCCCCAAGAAGAA
TTACAAATTACTCAATGGATAAAAGAATGGCATGGAGAATTTTTGCTTGAAAGAGATAAT
AGATCAAAATTGCCAAAAAGTAAATGTAAAAATAATACATTATATGAAGCATGTGAGAA
GGAATGTATTGATCCATGTATGAAATATAGAGATTGGATTATTAGAAGTAAATTTGAATG
GCATACGTTATCGAAAGAATATGAAACTCAAAAAGTTCCAAAGGAAAATGCGGAAAATT
ATTTAATCAAAATTTCAGAAAACAAGAATGATGCTAAAGTAAGTTTATTATTGAATAATT
GTGATGCTGAATATTCAAAATATTGTGATTGTAAACATACTACTACTCTCGTTAAAAGCGT
TTTAAATGGTAACGACAATACAATTAAGGAAAAGCGTGAACATATTGATTTAGATGATTT
TTCTAAATTTGGATGTGATAAAAATTCCGTTGATACAAACACAAAGGTGTGGGAATGTAA
AAAACCTTATAAATTATCCACTAAAGATGTATGTGTACCTCCGAGGAGGCAAGAATTATG
TCTTGGAAACATTGATAGAATATACGATAAAAACCTATTAATGATAAAAGAGCATATTCT
TGCTATTGCAATATATGAATCAAGAATATTGAAACGAAAATATAAGAATAAAGATGATAA
AGAAGTTTGTAAAATCATAAATAAAACTTTCGCTGATATAAGAGATATTATAGGAGGTAC
TGATTATTGGAATGATTTGAGCAATAGAAAATTAGTAGGAAAAATTAACACAAATTCAAA
TTATGTTCACAGGAATAAACAAAATGATAAGCTTTTTCGTGATGAGTGGTGGAAAGTTAT
TAAAAAAGATGTATGGAATGTGATATCATGGGTATTCAAGGATAAAACTGTTTGTAAAGA
AGATGATATTGAAAATATACCACAATTCTTCAGATGGTTTAGTGAATGGGGTGATGATTA
TTGCCAGGATAAAACAAAAATG
>Kenya C338_EBA-175DQ092100.1
TATGTATGTATTCCTGATCGTAGAATCCAATTATGCATTGTTAATCTTAGCATTATTAAAA
CATATACAAAAGAGACCATGAAGGATCATTTCATTGAAGCCTCTAAAAAAGAATCTCAA
CTTTTGCTTAAAAAAAATGATAACAAATATAATTCTAAATTTTGTAATGATTTGAAGAATA
GTTTTTTAGATTATGGACATCTTGCTATGGGAAATGATATGGATTTTGGAGGTTATTCAAC
TAAGGCAGAAAACAAAATTCAAGAAGTTTTTAAAGGGGCTCATGGGAAAATAAGTGAAC
ATGAAATTAAAAATTTTAGAAAAAAAATGGTGGAATGAATTTAGAGAGAAACTTTGGGAA
GCTATGTTATCTGAGCATAAAAATAATATAAATAATTGTAAAAATATTCCCCAAGAAGAA
TTACAAATTACTCAATGGATAAAAGAATGGCATGGAGAATTTTTGCTTGAAAGATATAAT
AGATCAAAATTGCCAAAAAGTAAATGTAAAAATAATACATTATATGAAGCATGTGAGAA
GGAATGTATTGATCCATGTATGAAATATAGAGATTGGATTATTAGAAGTAAATTTGAATG
GCATACGTTATCGAAAGAATATGAAACTCAAAATGTTTCAAAGGAAAATGCGGAAAATT
ATTTAATCAAAATTTCAGAAAACATGAATGATGCTAAAGTAAGTTTATTATTGAATAATTG
TGATGCTGAATATTCAAAATATTGTGATTGTAAACATACTACTACTCTCGTTAAAAGCGTT
TTAAATGGTAACGACAATACAATTAAGGAAAAGCGTGAACATATTGATTTAGATGATTTT
TCTAAATTTGGATGTGATAAAAATTCCGTTGATACAAACACAAAGGTGTGGGAATGTAAA
AACCCTTATATATTATCCACTAAAGATGTATGTGTACCTCCGAGGAGGCAAGAATTATGT

CTTGGAAACATTGATAGAATATACGATAAAAACCTATTAATGATAAAAGAGCATATTCTT
GCTATTGCAATATATGAATCAAGAATATTGAAACGAAAATATAAGAATAAAGATGATAA
AGAAGTTTGTAAAATCATAAATAAAACTTTCGCTGATATAAGAGATATTATAGGAGGTAC
TGATTATTGGAATGATTTGAGCAATAGAAAATTAGTAGGAAAAATTAACACAAATTCAAA
TTATGTTCACAGGAATAAAAAAAATGATAAGCTTTTTCGTGATGAGTGGTGGAAAGTTAT
TAAAAAAGATGTATGGAATGTGATATCATGGGTATTCAAGGATAAAACTGTTTGTAAAGA
AGATGATATTGAAAATATACCACAATTCTTCAGATGGTTTAGTGAATGGGGTGATGATTA
TTGCCAGGATAAAACAAAAATG
>Kenya C325_EBA-175DQ092099.1
TATGTATGTATTCCTGATCGTAGAATCCAATTATGCATTGTTAATCTTAGCATTATTAAAA
CATATACAAAAGAGACCATGAAGGATCATTTCATTGAAGCCTCTAAAAAAGAATCTCAA
CTTTTGCTTAAAAAAAATGATAACAAATATAATTCTAAATTTTGTAATGATTTGAAGAATA
GTTTTTTAGATTATGGACATCTTGCTATGGGAAATGATATGGATTTTGGAGGTTATTCAAC
TAAGGCAGAAAACAAAATTCAAGAAGTTTTTAAAGGGGCTCATGGGGAAATAAGTGAAC
ATGAAATTAAAAATTTTAGAAAAAAATGGTGGAATGAATTTAGAGAGAAACTTTGGGAA
GCTATGTTATCTGAGCATAAAAATAATATAAATAATTGTAAAAATATTCCCCAAGAAGAA
TTACAAATTACTCAATGGATAAAAGAATGGCATGGAGAATTTTTGCTTGAAAGAGATAAT
AGATCAAAATTGCCAAAAAGTAAATGTAAAAATAATACATTATATGAAGCATGTGAGAA
GGAATGTATTGATCCATGTATGAAATATAGAGATTGGATTATTAGAAGTAAATTTGAATG
GCATACGTTATCGAAAGAATATGAAACTCAAAAAGTTTCAAAGGAAAATGCGGAAAATT
ATTTAATCAAAATTTCAGAAAACAAGAATGATGCTAAAGTAAGTTTATTATTGAATAATT
GTGATGCTGAATATTCAAAATATTGTGATTGTAAACATACTACTACTCTCGTTAAAAGCGT
TTTAAATGGTAACGACAATACAATTAAGGAAAAGCGTGAACATATTGATTTAGATGATTT
TTCTAAATTTGGATGTGATAAAAATTCCGTTGATACAAACACAAAGGTGTGGGAATGTAA
AAAACCTTATATATTATCCACTAAAGATGTATGTGTACCTCCGAGGAGGCAAGAATTATG
TCTTGGAAACATTGATAGAATATACGATAAAAACCTATTAATGATAAAAGAGCATATTCT
TGCTATTGCAATATATGAATCAAGAATATTGAAACGAAAATATAAGAATAAAGATGATAA
AGAAGTTTGTAAAATCATAAATAAAACTTTCGCTGATATAAGAGATATTATAGGAGGTAC
TGATTATTGGAATGATTTGAGCAATAGAAAATTAGTAGGAAAAATTAACACAAATTCAAA
ATATGTTCACAGGAATAAAAAAAATGATAAGCTTTTTCGTGATGAGTGGTGGAAAGTTAT
TAAAAAAGATGTATGGAATGTGATATCATGGGTATTCAAGGATAAAACTGTTTGTAAAGA
AGATGATATTGAAAATATACCACAATTCTTCAGATGGTTTAGTGAATGGGGTGATGATTA
TTGCCAGGATAAAACAAAAATG
>Kenya C304_EBA-175DQ092098.1
TATGTATGTATTCCTGATCGTAGAATCCAATTATGCATTGTTAATCTTAGCATTATTAAAA
CATATACAAAAGAGACCATGAAGGATCATTTCATTGAAGCCTCTAAAAAAGAATCTCAA
CTTTTGCTTAAAAAAAATGATAACAAATATAATTCTAAATTTTGTAATGATTTGAAGAATA
GTTTTTTAGATTATGGACATCTTGCTATGGGAAATGATATGGATTTTGGAGGTTATTCAAC
TAAGGCAGAAAACAAAATTCAAGAAGTTTTTAAAGGGGCTCATGGGAAAATAAGTGAAC
ATGAAATTAAAAATTTTAGAAAAAAATGGTGGAATGAATTTAGAGAGAAACTTTGGGAA
GCTATGTTATCTGAGCATAAAAATAATATAAATAATTGTAAAAATATTCCCCAAGAAGAA
TTACAAATTACTCAATGGATAAAAGAATGGCATGGAGAATTTTTGCTTGAAAGAGATAAT
AGATCAAAATTGCCAAAAAGTAAATGTAAAAATAATACATTATATGAAGCATGTGAGAA
GGAATGTATTGATCCATGTATGAAATATAGAGATTGGATTATTAGAAGTAAATTTGAATG

GCATACGTTATCGAAAGAATATGAAACTCAAAATGTTTCAAAGGAAAATGCGGAAAATT
ATTTAATCAAAATTTCAAAAAACAAGAATGATGCTAAAGTAAGTTTATTATTGAATAATTG
TGATGCTGAATATTCAAAATATTGTGATTGTAAACATACTACTACTCTCGTTAAAAGCGTT
TTAAATGGTAACGACAATACAATTAAGGAAAAGCGTGAACATATTGATTTAGATGATTTT
TCTAAATTTGGATGTGATAAAAATTCCGTTGATACAAACACAAAGGTGTGGGAATGTAAA
AACCCTTATATATTATCCACTAAAGATGTATGTGTACCTCCGAGGAGGCAAGAATTATGT
CTTGGAAACATTGATAGAATATACGATAAAAACCTATTAATGATAAAAGAGCATATTCTT
GCTATTGCAATATATGAATCAAGAATATTGAAACGAAAATATAAGAATAAAGATGATAA
AGAAGTTTGTAAAATCATAAATAAAACTTTCGCTGATATAAGAGATATTATAGGAGGTAC
TGATTATTGGAATGATTTGAGCAATAGAAAATTAGTAGGAAAAATTAACACAAATTCAAA
TTATGTTCACAGGAATAAAAAAAATGATAAGCTTTTTCGTGATGAGTGGTGGAAAGTTAT
TAAAAAAGATGTATGGAATGTGATATCATGGGTATTCAAGGATAAAACTGTTTGTAAAGA
AGATGATATTGAAAATATACCACAATTCTTCAGATGGTTTAGTGAATGGGGTGATGATTA
TTGCCAGGATAAAACAAAAATG
>Kenya C298_EBA-175DQ092097.1
TATGTATGTATTCCTGATCGTAGAATCCAATTATGCATTGTTAATCTTAGCATTATTAAAA
CATATACAAAAGAGACCATGAAGGATCATTTCATTGAAGCCTCTAAAAAAGAATCTCAA
CTTTTGCTTAAAAAAAAATGATAACAAATATAATTCTAAATTTTGTAATGATTTGAAGAATA
GTTTTTTAGATTATGGACATCTTGCTATGGGAAATGATATGGATTTTGGAGGTTATTCAAC
TAAGGCAGAAAACAAAATTCAAGAAGTTTTTAAAGGGGCTCATGGGGAAATAAGTGAAC
ATAAAATTAAAAATTTTAGAAAAGAATGGTGGAATGAATTTAGAGAGAAACTTTGGGAA
GCTATGTTATCTGAGCATAAAAATAATATAAATAATTGTAAAAATATTCCCCAAGAAGAA
TTACAAATTACTCAATGGATAAAAGAATGGCATGGAGAATTTTTGCTTGAAAGAGATAAT
AGATCAAAATTGCCAAAAAGTAAATGTAAAAATAATACATTATATGAAGCATGTGAGAA
GGAATGTATTGATCCATGTATGAAATATAGAGATTGGATTATTAGAAGTAAATTTGAATG
GCATACGTTATCGAAAGAATATGAAACTCAAAAAGTTCCAAAGGAAAATGCGGAAAATT
ATTTAATCAAAATTTCAGAAAACAAGAATGATGCTAAAGTAAGTTTATTATTGAATAATT
GTGATGCTGAATATTCAAAATATTGTGATTGTAAACATACTACTACTCTCGTTAAAAGCGT
TTTAAATGGTAACGACAATACAATTAAGGAAAAGCGTGAACATATTGATTTAGATGATTT
TTCTAAATTTGGATGTGATAAAAATTCCGTTGATACAAACACAAAGGTGTGGGAATGTAA
AAAACCTTATAAATTATCCACTAAAGATGTATGTGTACCTCCGAGGAGGCAAGAATTATG
TCTTGGAAACATTGATAGAATATACGATAAAAACCTATTAATGATAAAAGAGCATATTCT
TGCTATTGCAATATATGAATCAAGAATATTGAAACGAAAATATAAGAATAAAGATGATAA
AGAAGTTTGTAAAATCATAAATAAAACTTTCGCTGATATAAGAGATATTATAGGAGGTAC
TGATTATTGGAATGATTTGAGCAATAGAAAATTAGTAGGAAAAATTAACACAAATTCAAA
TTATGTTCACAGGAATAAACAAAATGATAAGCTTTTTCGTGATGAGTGGTGGAAAGTTAT
TAAAAAAGATGTATGGAATGTGATATCATGGGTATTCAAGGATAAAACTGTTTGTAAAGA
AGATGATATTGAAAATATACCACAATTCTTCAGATGGTTTAGTGAATGGGGTGATGATTA
TTGCCAGGATAAAACAAAAATG
>Kenya C289_EBA-175DQ092096.1
TATGTATGTATTCCTGATCGTAGAATCCAATTATGCATTGTTAATCTTAGCATTATTAAAA
CATATACAAAAGAGACCATGAAGGATCATTTCATTGAAGCCTCTAAAAAAGAATCTCAA
CTTTTGCTTAAAAAAAAATGATAACAAATATAATTCTAAATTTTGTAATGATTTGAAGAATA
GTTTTTTAGATTATGGACATCTTGCTATGGGAAATGATATGGATTTTGGAGGTTATTCAAC

TAAGGCAGAAAACAAAATTCAAGAAGTTTTTAAAGGGGCTCATGGGAAAATAAGTGAAC
ATGAAATTAAAAATTTTAGAAAAAAATGGTGGAATGAATTTAGAGAGAAACTTTGGGAA
GCTATGTTATCTGAGCATAAAAATAATATAAATAATTGTAAAAATATTCCCCAAGAAGAA
TTACAAATTACTCAATGGATAAAAGAATGGCATGGAGAATTTTTGCTTGAAAGAGATAAT
AGATCAAAATTGCCAAAAAGTAAATGTAAAAATAATACATTATATGAAGCATGTGAGAA
GGAATGTATTGATCCATGTATGAAATATAGAGATTGGATTATTAGAAGTAAATTTGAATG
GCATACGTTATCGAAAGAATATGAAACTCAAAAAGTTCCAAAGGAAAATGCGGAAAATT
ATTTAATCAAAATTTCAGAAAACATGAATGATGCTAAAGTAAGTTTATTATTGAATAATTG
TGATGCTGAATATTCAAAATATTGTGATTGTAAACATACTACTACTCTCGTTAAAAGCGTT
TTAAATGGTAACGACAATACAATTAAGGAAAAGCGTGAACATATTGATTTAGATGATTTT
TCTAAATTTGGATGTGATAAAAATTCCGTTGATACAAACACAAAGGTGTGGGAATGTAAA
AAACCTTATAAATTATCCACTAAAGATGTATGTGTACCTCCGAGGAGGCAAGAATTATGT
CTTGGAAACATTGATAGAATATACGATAAAAACCTATTAATGATAAAAGAGCATATTCTT
GCTATTGCAATATATGAATCAAGAATATTGAAACGAAAATATAAGAATAAAGATGATAA
AGAAGTTTGTAAAATCATAAATAAAACTTTCGCTGATATAAGAGATATTATAGGAGGTAC
TGATTATTGGAATGATTTGAGCAATAGAAAATTAGTAGGAAAAATTAACACAAATTCAAA
TTATGTTCACAGGAATAAACAAAATGATAAGCTTTTTCGTGATGAGTGGTGGAAAGTTAT
TAAAAAAGATGTATGGAATGTGATATCATGGGTATTCAAGGATAAAACTGTTTGTAAAGA
AGATGATATTGAAAATATACCACAATTCTTCAGATGGTTTAGTGAATGGGGTGATGATTA
TTGCCAGGATAAAACAAAAATG
>Kenya C282_EBA-175DQ092095.1
TATGTATGTATTCCTGATCGTAGAATCCAATTATGCATTGTTAATCTTAGCATTATTAAAA
CATATACAAAAGAGACCATGAAGGATCATTTCATTGAAGCCTCTAAAAAAGAATCTCAA
CTTTTGCTTAAAAAAAAATGATAACAAATATAATTCTAAATTTTGTAATGATTTGAAGAATA
GTTTTTTAGATTATGGACATCTTGCTATGGGAAATGATATGGATTTTGGAGGTTATTCAAC
TAAGGCAGAAAACAAAATTCAAGAAGTTTTTAAAGGGGCTCATGGGAAAATAAGTGAAC
ATGAAATTAAAAATTTTAGAAAAGAATGGTGGAATGAATTTAGAGAGAAACTTTGGGAA
GCTATGTTATCTGAGCATAAAAATAATATAAATAATTGTAAAAATATTCCCCAAGAAGAA
TTACAAATTACTCAATGGATAAAAGAATGGCATGGAGAATTTTTGCTTGAAAGAGATAAT
AGATCAAAATTGCCAAAAAGTAAATGTAAAAATAATACATTATATGAAGCATGTGAGAA
GGAATGTATTGATCCATGTATGAAATATAGAGATTGGATTATTAGAAGTAAATTTGAATG
GCATACGTTATCGAAAGAATATGAAACTCAAAATGTTTCAAAGGAAAATGCGGAAAATT
ATTTAATCAAAATTTCAGAAAACAAGAATGATGCTAAAGTAAGTTTATTATTGAATAATT
GTGATGCTGAATATTCAAAATATTGTGATTGTAAACATACTACTACTCTCGTTAAAAGCGT
TTTAAATGGTAACGACAATACAATTAAGGAAAAGCGTGAACATATTGATTTAGATGATTT
TTCTAAATTTGGATGTGATAAAAATTCCGTTGATACAAACACAAAGGTGTGGGAATGTAA
AAAACCTTATAAATTATCCACTAAAGATGTATGTGTACCTCCGAGGAGGCAAGAATTATG
TCTTGGAAACATTGATAGAATATACGATAAAAACCTATTAATGATAAAAGAGCATATTCT
TGCTATTGCAATATATGAATCAAGAATATTGAAACGAAAATATAAGAATAAAGATGATAA
AGAAGTTTGTAAAATCATAAATAAAACTTTCGCTGATATAAGAGATATTATAGGAGGTAC
TGATTATTGGAATGATTTGAGCAATAGAAAATTAGTAGGAAAAATTAACACAAATTCAAA
TTATGTTCACAGGAATAAACAAAATGATAAGCTTTTTCGTGATGCGTGGTGGAAAGTTAT
TAAAAAAGATGTATGGAATGTGATATCATGGGTATTCAAGGATAAAACTGTTTGTAAAGA
AGATGATATTGAAAATATACCACAATTCTTCAGATGGTTTAGTGAATGGGGTGATGATTA

TTGCCAGGATAAAACAAAAATG
>Kenya C256_EBA-175DQ092094.1
TATGTATGTATTCCTGATCGTAGAATCCAATTATGCATTGTTAATCTTAGCATTATTAAAA
CATATACAAAAGAGACCATGAAGGATCATTTCATTGAAGCCTCTAAAAAAGAATCTCAA
CTTTTGCTTAAAAAAAATGATAACAAATATAATTCTAAATTTTGTAATGATTTGAAGAATA
GTTTTTTAGATTATGGACATCTTGCTATGGGAAATGATATGGATTTTGGAGGTTATTCAAC
TAAGGCAGAAAACAAAATTCAAGAAGTTTTTAAAGGGGCTCATGGGGAAATAAGTGAAC
ATAAAATTAAAAATTTTAGAAAAGAATGGTGGAATGAATTTAGAGAGAAACTTTGGGAA
GCTATGTTATCTGAGCATAAAAATAATATAAATAATTGTAAAAATATTCCCCAAGAAGAA
TTACAAATTACTCAATGGATAAAAGAATGGCATGGAGAATTTTTGCTTGAAAGAGATAAT
AGATCAAAATTGCCAAAAAGTAAATGTAAAAATAATACATTATATGAAGCATGTGAGAA
GGAATGTATTGATCCATGTATGAAATATAGAGATTGGATTATTAGAAGTAAATTTGAATG
GCATACGTTATCGAAAGAATATGAAACTCAAAAAGTTCCAAAGGAAAATGCGGAAAATT
ATTTAATCAAAATTTCAGAAAACAAGAATGATGCTAAAGTAAGTTTATTATTGAATAATT
GTGATGCTGAATATTCAAAATATTGTGATTGTAAACATACTACTACTCTCGTTAAAAGCGT
TTTAAATGGTAACGACAATACAATTAAGGAAAAGCGTGAACATATTGATTTAGATGATTT
TTCTAAATTTGGATGTGATAAAAATTCCGTTGATACAAACACAAAGGTGTGGGAATGTAA
AAAACCTTATAAATTATCCACTAAAGATGTATGTGTACCTCCGAGGAGGCAAGAATTATG
TCTTGGAAACATTGATAGAATATACGATAAAAACCTATTAATGATAAAAGAGCATATTCT
TGCTATTGCAATATATGAATCAAGAATATTGAAACGAAAATATAAGAATAAAGATGATAA
AGAAGTTTGTAAAATCATAAATAAAACTTTCGCTGATATAAGAGATATTATAGGAGGTAC
TGATTATTGGAATGATTTGAGCAATAGAAAATTAGTAGGAAAAATTAACACAAATTCAAA
TTATGTTCACAGGAATAAACAAAATGATAAGCTTTTTCGTGATGAGTGGTGGAAAGTTAT
TAAAAAAGATGTATGGAATGTGATATCATGGGTATTCAAGGATAAAACTGTTTGTAAAGA
AGATGATATTGAAAATATACCACAATTCTTCAGATGGTTTAGTGAATGGGGTGATGATTA
TTGCCAGGATAAAACAAAAATG
>Kenya C229_EBA-175DQ092093.1
TATGTATGTATTCCTGATCGTAGAATCCAATTATGCATTGTTAATCTTAGCATTATTAAAA
CATATACAAAAGAGACCATGAAGGATCATTTCATTGAAGCCTCTAAAAAAGAATCTCAA
CTTTTGCTTAAAAAAAATGATAACAAATATAATTCTAAATTTTGTAATGATTTGAAGAATA
GTTTTTTAGATTATGGACATCTTGCTATGGGAAATGATATGGATTTTGGAGGTTATTCAAC
TAAGGCAGAAAACAAAATTCAAGAAGTTTTTAAAGGGGCTCATGGGGAAATAAGTGAAC
ATGAAATTAAAAATTTTAGAAAAAAATGGTGGAATGAATTTAGAGAGAAACTTTGGGAA
GCTATGTTATCTGAGCATAAAAATAATATAAATAATTGTAAAAATATTCCCCAAGAAGAA
TTACAAATTACTCAATGGATAAAAGAATGGCATGGAGAATTTTTGCTTGAAAGAGATAAT
AGATCAAAATTGCCAAAAAGTAAATGTAAAAATAATACATTATATGAAGCATGTGAGAA
GGAATGTATTGATCCATGTATGAAATATAGAGATTGGATTATTAGAAGTAAATTTGAATG
GCATACGTTATCGAAAGAATATGAAACTCAAAAAGTTTCAAAGGAAAATGCGGAAAATT
ATTTAATCAAAATTTCAGAAAACAAGAATGATGCTAAAGTAAGTTTATTATTGAATAATT
GTGATGCTGAATATTCAAAATATTGTGATTGTAAACATACTACTACTCTCGTTAAAAGCGT
TTTAAATGGTAACGACAATACAATTAAGGAAAAGCGTGAACATATTGATTTAGATGATTT
TTCTAAATTTGGATGTGATAAAAATTCCGTTGATACAAACACAAAGGTGTGGGAATGTAA
AAAACCTTATATATTATCCACTAAAGATGTATGTGTACCTCCGAGGAGGCAAGAATTATG
TCTTGGAAACATTGATAGAATATACGATAAAAACCTATTAATGATAAAAGAGCATATTCT

TGCTATTGCAATATATGAATCAAGAATATTGAAACGAAAATATAAGAATAAAGATGATAA
AGAAGTTTGTAAAATCATAAATAAAACTTTCGCTGATATAAGAGATATTATAGGAGGTAC
TGATTATTGGAATGATTTGAGCAATAGAAAATTAGTAGGAAAAATTAACACAAATTCAAA
ATATGTTCACAGGAATAAAAAAAATGATAAGCTTTTTCGTGATGAGTGGTGGAAAGTTAT
TAAAAAAGATGTATGGAATGTGATATCATGGGTATTCAAGGATAAAACTGTTTGTAAAGA
AGATGATATTGAAAATATACCACAATTCTTCAGATGGTTTAGTGAATGGGGTGATGATTA
TTGCCAGGATAAAACAAAAATG
>Kenya C226_EBA-175DQ092092.1
TATGTATGTATTCCTGATCGTAGAATCCAATTATGCATTGTTAATCTTAGCATTATTAAAA
CATATACAAAAGAGACCATGAAGGATCATTTCATTGAAGCCTCTAAAAAAGAATCTCAA
CTTTTGCTTAAAAAAAAATGATAACAAATATAATTCTAAATTTTGTAATGATTTGAAGAATA
GTTTTTTAGATTATGGACATCTTGCTATGGGAAATGATATGGATTTTGGAGGTTATTCAAC
TAAGGCAGAAAACAAAATTCAAGAAGTTTTTAAAGGGGCTCATGGGGAAATAAGTGAAC
ATGAAATTAAAAATTTTAGAAAAAAATGGTGGAATGAATTTAGAGAGAAACTTTGGGAA
GCTATGTTATCTGAGCATAAAAATAATATAAATAATTGTAAAAATATTCCCCAAGAAGAA
TTACAAATTACTCAATGGATAAAAGAATGGCATGGAGAATTTTTGCTTGAAAGAGATAAT
AGATCAAAATTGCCAAAAAGTAAATGTAAAAATAATACATTATATGAAGCATGTGAGAA
GGAATGTATTGATCCATGTATGAAATATAGAGATTGGATTATTAGAAGTAAATTTGAATG
GCATACGTTATCGAAAGAATATGAAACTCAAAAAGTTTCAAAGGAAAATGCGGAAAATT
ATTTAATCAAAATTTCAGAAAACAAGAATGATGCTAAAGTAAGTTTATTATTGAATAATT
GTGATGCTGAATATTCAAAATATTGTGATTGTAAACATACTACTACTCTCGTTAAAAGCGT
TTTAAATGGTAACGACAATACAATTAAGGAAAAGCGTGAACATATTGATTTAGATGATTT
TTCTAAATTTGGATGTGATAAAAATTCCGTTGATACAAACACAAAGGTGTGGGAATGTAA
AAAACCTTATATATTATCCACTAAAGATGTATGTGTACCTCCGAGGAGGCAAGAATTATG
TCTTGGAAACATTGATAGAATATACGATAAAAACCTATTAATGATAAAAGAGCATATTCT
TGCTATTGCAATATATGAATCAAGAATATTGAAACGAAAATATAAGAATAAAGATGATAA
AGAAGTTTGTAAAATCATAAATAAAACTTTCGCTGATATAAGAGATATTATAGGAGGTAC
TGATTATTGGAATGATTTGAGCAATAGAAAATTAGTAGGAAAAATTAACACAAATTCAAA
ATATGTTCACAGGAATAAAAAAAATGATAAGCTTTTTCGTGATGAGTGGTGGAAAGTTAT
TAAAAAAGATGTATGGAATGTGATATCATGGGTATTCAAGGATAAAACTGTTTGTAAAGA
AGATGATATTGAAAATATACCACAATTCTTCAGATGGTTTAGTGAATGGGGTGATGATTA
TTGCCAGGATAAAACAAAAATG
>Kenya C171_EBA-175DQ092091.1
TATGTATGTATTCCTGATCGTAGAATCCAATTATGCATTGTTAATCTTAGCATTATTAAAA
CATATACAAAAGAGACCATGAAGGATCATTTCATTGAAGCCTCTAAAAAAGAATCTCAA
CTTTTGCTTAAAAAAAAATGATAACAAATATAATTCTAAATTTTGTAATGATTTGAAGAATA
GTTTTTTAGATTATGGACATCTTGCTATGGGAAATGATATGGATTTTGGAGGTTATTCAAC
TAAGGCAGAAAACAAAATTCAAGAAGTTTTTAAAGGGGCTCATGGGGAAATAAGTGAAC
ATGAAATTAAAAATTTTAGAAAAAAATGGTGGAATGAATTTAGAGAGAAACTTTGGGAA
GCTATGTTATCTGAGCATAAAAATAATATAAATAATTGTAAAAATATTCCCCAAGAAGAA
TTACAAATTACTCAATGGATAAAAGAATGGCATGGAGAATTTTTGCTTGAAAGAGATAAT
AGATCAAAATTGCCAAAAAGTAAATGTAAAAATAATACATTATATGAAGCATGTGAGAA
GGAATGTATTGATCCATGTATGAAATATAGAGATTGGATTATTAGAAGTAAATTTGAATG
GCATACGTTATCGAAAGAATATGAAACTCAAAAAGTTTCAAAGGAAAATGCGGAAAATT

ATTTAATCAAAATTTCAGAAAACAAGAATGATGCTAAAGTAAGTTTATTATTGAATAATT
GTGATGCTGAATATTCAAAATATTGTGATTGTAAACATACTACTACTCTCGTTAAAAGCGT
TTTAAATGGTAACGACAATACAATTAAGGAAAAGCGTGAACATATTGATTTAGATGATTT
TTCTAAATTTGGATGTGATAAAAATTCCGTTGATACAAACACAAAGGTGTGGGAATGTAA
AAAACCTTATATATTATCCACTAAAGATGTATGTGTACCTCCGAGGAGGCAAGAATTATG
TCTTGGAAACATTGATAGAATATACGATAAAAACCTATTAATGATAAAAGAGCATATTCT
TGCTATTGCAATATATGAATCAAGAATATTGAAACGAAAATATAAGAATAAAGATGATAA
AGAAGTTTGTAAAATCATAAATAAAACTTTCGCTGATATAAGAGATATTATAGGAGGTAC
TGATTATTGGAATGATTTGAGCAATAGAAAATTAGTAGGAAAAATTAACACAAATTCAAA
ATATGTTCACAGGAATAAAAAAAATGATAAGCTTTTTCGTGATGAGTGGTGGAAAGTTAT
TAAAAAAGATGTATGGAATGTGATATCATGGGTATTCAAGGATAAAACTGTTTGTAAAGA
AGATGATATTGAAAATATACCACAATTCTTCAGATGGTTTAGTGAATGGGGTGATGATTA
TTGCCAGGATAAAACAAAAATG
>Kenya C157_EBA-175DQ092090.1
TATGTATGTATTCCTGATCGTAGAATCCAATTATGCATTGTTAATCTTAGCATTATTAAAA
CATATACAAAAGAGACCATGAAGGATCATTTCATTGAAGCCTCTAAAAAAGAATCTCAA
CTTTTGCTTAAAAAAAAATGATAACAAATATAATTCTAAATTTTGTAATGATTTGAAGAATA
GTTTTTTAGATTATGGACATCTTGCTATGGGAAATGATATGGATTTTGGAGGTTATTCAAC
TAAGGCAGAAAACAAAATTCAAGAAGTTTTTAAAGGGGCTCATGGGGAAATAAGTGAAC
ATGAAATTAAAAATTTTAGAAAAAAATGGTGGAATGAATTTAGAGAGAAACTTTGGGAA
GCTATGTTATCTGAGCATAAAAATAATATAAATAATTGTAAAAATATTCCCCAAGAAGAA
TTACAAATTACTCAATGGATAAAAGAATGGCATGGAGAATTTTTGCTTGAAAGAGATAAT
AGATCAAAATTGCCAAAAAGTAAATGTAAAAATAATACATTATATGAAGCATGTGAGAA
GGAATGTATTGATCCATGTATGAAATATAGAGATTGGATTATTAGAAGTAAATTTGAATG
GCATACGTTATCGAAAGAATATGAAACTCAAAAAGTTTCAAAGGAAAATGCGGAAAATT
ATTTAATCAAAATTTCAGAAAACAAGAATGATGCTAAAGTAAGTTTATTATTGAATAATT
GTGATGCTGAATATTCAAAATATTGTGATTGTAAACATACTACTACTCTCGTTAAAAGCGT
TTTAAATGGTAACGACAATACAATTAAGGAAAAGCGTGAACATATTGATTTAGATGATTT
TTCTAAATTTGGATGTGATAAAAATTCCGTTGATACAAACACAAAGGTGTGGGAATGTAA
AAAACCTTATATATTATCCACTAAAGATGTATGTGTACCTCCGAGGAGGCAAGAATTATG
TCTTGGAAACATTGATAGAATATACGATAAAAACCTATTAATGATAAAAGAGCATATTCT
TGCTATTGCAATATATGAATCAAGAATATTGAAACGAAAATATAAGAATAAAGATGATAA
AGAAGTTTGTAAAATCATAAATAAAACTTTCGCTGATATAAGAGATATTATAGGAGGTAC
TGATTATTGGAATGATTTGAGCAATAGAAAATTAGTAGGAAAAATTAACACAAATTCAAA
TTATGTTCACAGGAATAAAAAAAATGATAAGCTTTTTCGTGATGAGTGGTGGAAAGTTAT
TAAAAAAGATGTATGGAATGTGATATCATGGGTATTCAAGGATAAAACTGTTTGTAAAGA
AGATGATATTGAAAATATACCACAATTCTTCAGATGGTTTAGTGAATGGGGTGATGATTA
TTGCCAGGATAAAACAAAAATG
>Kenya C066_EBA-175DQ092089.1
TATGTATGTATTCCTGATCGTAGAATCCAATTATGCATTGTTAATCTTAGCATTATTAAAA
CATATACAAAAGAGACCATGAAGGATCATTTCATTGAAGCCTCTAAAAAAGAATCTCAA
CTTTTGCTTAAAAAAAAATGATAACAAATATAATTCTAAATTTTGTAATGATTTGAAGAATA
GTTTTTTAGATTATGGACATCTTGCTATGGGAAATGATATGGATTTTGGAGGTTATTCAAC
TAAGGCAGAAAACAAAATTCAAGAAGTTTTTAAAGGGGCTCATGGAAAATAAGTGAAC

ATGAAATTAAAAATTTTAGAAAAGAATGGTGGAATGAATTTAGAGAGAAACTTTGGGAA
GCTATGTTATCTGAGCATAAAAATAATATAAATAATTGTAAAAATATTCCCCAAGAAGAA
TTACAAATTACTCAATGGATAAAAGAATGGCATGGAGAATTTTTGCTTGAAAGAGATAAT
AGATCAAAATTGCCAAAAAGTAAATGTAAAAATAATACATTATATGAAGCATGTGAGAA
GGAATGTATTGATCCATGTATGAAATATAGAGATTGGATTATTAGAAGTAAATTTGAATG
GCATACGTTATCGAAAGAATATGAAACTCAAAAAGTTCCAAAGGAAAATGCGGAAAATT
ATTTAATCAAAATTTCAGAAAACATGAATGATGCTAAAGTAAGTTTATTATTGAATAATTG
TGATGCTGAATATTCAAAATATTGTGATTGTAAACATACTACTACTCTCGTTAAAAGCGTT
TTAAATGGTAACGACAATACAATTAAGGAAAAGCGTGAACATATTGATTTAGATGATTTT
TCTAAATTTGGATGTGATAAAAATTCCGTTGATACAAACACAAAGGTGTGGGAATGTAAA
AAACCTTATATATTATCCACTAAAGATGTATGTGTACCTCCGAGGAGGCAAGAATTATGT
CTTGGAAACATTGATAGAATATACGATAAAAACCTATTAATGATAAAAGAGCATATTCTT
GCTATTGCAATATATGAATCAAGAATATTGAAACGAAAATATAAGAATAAAGATGATAA
AGAAGTTTGTAAAATCATAAATAAAACTTTCGCTGATATAAGAGATATTATAGGAGGTAC
TGATTATTGGAATGATTTGAGCAATAGAAAATTAGTAGGAAAAATTAACACAAATTCAAA
TTATGTTCACAGGAATAAACAAAATGATAAGCTTTTTCGTGATGAGTGGTGGAAAGTTAT
TAAAAAAGATGTATGGAATGTGATATCATGGGTATTCAAGGATAAAACTGTTTGTAAAGA
AGATGATATTGAAAATATACCACAATTCTTCAGATGGTTTAGTGAATGGGGTGATGATTA
TTGCCAGGATAAAACAAAAATG
>Kenya C057_EBA-175DQ092088.1
TATGTATGTATTCCTGATCGTAGAATCCAATTATGCATTGTTAATCTTAGCATTATTAAAA
CATATACAAAAGAGACCATGAAGGATCATTTCATTGAAGCCTCTAAAAAAGAATCTCAA
CTTTTGCTTAAAAAAAAATGATAACAAATATAATTCTAAATTTTGTAATGATTTGAAGAATA
GTTTTTTAGATTATGGACATCTTGCTATGGGAAATGATATGGATTTTGGAGGTTATTCAAC
TAAGGCAGAAAACAAAATTCAAGAAGTTTTTAAAGGGGCTCATGGGGAAATAAGTGAAC
ATGAAATTAAAAATTTTAGAAAAAAAATGGTGGAATGAATTTAGAGAGAAACTTTGGGAA
GCTATGTTATCTGAGCATAAAAATAATATAAATAATTGTAAAAATATTCCCCAAGAAGAA
TTACAAATTACTCAATGGATAAAAGAATGGCATGGAGAATTTTTGCTTGAAAGAGATAAT
AGATCAAAATTGCCAAAAAGTAAATGTAAAAATAATACATTATATGAAGCATGTGAGAA
GGAATGTATTGATCCATGTATGAAATATAGAGATTGGATTATTAGAAGTAAATTTGAATG
GCATACGTTATCGAAAGAATATGAAACTCAAAAAGTTTCAAAGGAAAATGCGGAAAATT
ATTTAATCAAAATTTCAGAAAACAAGAATGATGCTAAAGTAAGTTTATTATTGAATAATT
GTGATGCTGAATATTCAAAATATTGTGATTGTAAACATACTACTACTCTCGTTAAAAGCGT
TTTAAATGGTAACGACAATACAATTAAGGAAAAGCGTGAACATATTGATTTAGATGATTT
TTCTAAATTTGGATGTGATAAAAATTCCGTTGATACAAACACAAAGGTGTGGGAATGTAA
AAAACCTTATATATTATCCACTAAAGATGTATGTGTACCTCCGAGGAGGCAAGAATTATG
TCTTGGAAACATTGATAGAATATACGATAAAAACCTATTAATGATAAAAGAGCATATTCT
TGCTATTGCAATATATGAATCAAGAATATTGAAACGAAAATATAAGAATAAAGATGATAA
AGAAGTTTGTAAAATCATAAATAAAACTTTCGCTGATATAAGAGATATTATAGGAGGTAC
TGATTATTGGAATGATTTGAGCAATAGAAAATTAGTAGGAAAAATTAACACAAATTCAAA
ATATGTTCACAGGAATAAAAAAAAATGATAAGCTTTTTCGTGATGAGTGGTGGAAAGTTAT
TAAAAAAGATGTATGGAATGTGATATCATGGGTATTCAAGGATAAAACTGTTTGTAAAGA
AGATGATATTGAAAATATACCACAATTCTTCAGATGGTTTAGTGAATGGGGTGATGATTA
TTGCCAGGATAAAACAAAAATG

>Kenya C007_EBA175DQ092087.1
TATGTATGTATTCCTGATCGTAGAATCCAATTATGCATTGTTAATCTTAGCATTATTAAAA
CATATACAAAAGAGACCATGAAGGATCATTTCATTGAAGCCTCTAAAAAAGAATCTCAA
CTTTTGCTTAAAAAAAAATGATAACAAATATAATTCTAAATTTTGTAATGATTTGAAGAATA
GTTTTTTAGATTATGGACATCTTGCTATGGGAAATGATATGGATTTTGGAGGTTATTCAAC
TAAGGCAGAAAACAAAATTCAAGAAGTTTTTAAAGGGGCTCATGGGGAAATAAGTGAAC
ATAAAATTAAAAATTTTAGAAAAGAATGGTGGAATGAATTTAGAGAGAAACTTTGGGAA
GCTATGTTATCTGAGCATAAAAATAATATAAATAATTGTAAAAATATTCCCCAAGAAGAA
TTACAAATTACTCAATGGATAAAAGAATGGCATGGAGAATTTTTGCTTGAAAGAGATAAT
AGATCAAAATTGCCAAAAAGTAAATGTAAAAATAATACATTATATGAAGCATGTGAGAA
GGAATGTATTGATCCATGTATGAAATATAGAGATTGGATTATTAGAAGTAAATTTGAATG
GCATACGTTATCGAAAGAATATGAAACTCAAAAAGTTCCAAAGGAAAATGCGGAAAATT
ATTTAATCAAAATTTCAGAAAACAAGAATGATGCTAAAGTAAGTTTATTATTGAATAATT
GTGATGCTGAATATTCAAAATATTGTGATTGTAAACATACTACTACTCTCGTTAAAAGCGT
TTTAAATGGTAACGACAATACAATTAAGGAAAAGCGTGAACATATTGATTTAGATGATTT
TTCTAAATTTGGATGTGATAAAAATTCCGTTGATACAAACACAAAGGTGTGGGAATGTAA
AAAACCTTATAAATTATCCACTAAAGATGTATGTGTACCTCCGAGGAGGCAAGAATTATG
TCTTGGAAACATTGATAGAATATACGATAAAAACCTATTAATGATAAAAGAGCATATTCT
TGCTATTGCAATATATGAATCAAGAATATTGAAACGAAAATATAAGAATAAAGATGATAA
AGAAGTTTGTAAAATCATAAATAAAACTTTCGCTGATATAAGAGATATTATAGGAGGTAC
TGATTATTGGAATGATTTGAGCAATAGAAAATTAGTAGGAAAAATTAACACAAATTCAAA
TTATGTTCACAGGAATAAACAAAATGATAAGCTTTTTCGTGATGAGTGGTGGAAAGTTAT
TAAAAAAGATGTATGGAATGTGATATCATGGGTATTCAAGGATAAAACTGTTTGTAAAGA
AGATGATATTGAAAATATACCACAATTCTTCAGATGGTTTAGTGAATGGGGTGATGATTA
TTGCCAGGATAAAACAAAAATG
>Thailand 2006 Th100_EBA-175DQ092086.1
TATGTATGTATTCCTGATCGTAGAATCCAATTATGCATTGTTAATCTTAGCATTATTAAAA
CATATACAAAAGAGACCATGAAGGATCATTTCATTGAAGCCTCTAAAAAAGAATCTCAA
CTTTTGCTTAAAAAAAAATGATAACAAATATAATTCTAAATTTTGTAATGATTTGAAGAATA
GTTTTTTAGATTATGGACATCTTGCTATGGGAAATGATATGGATTTTGGAGGTTATTCAAC
TAAGGCAGAAAACAAAATTCAAGAAGTTTTTAAAGGGGCTCATGGGAAAATAAGTGAAC
ATGAAATTAAAAATTTTAGAAAAGAATGGTGGAATGAATTTAGAGAGAAACTTTGGGAA
GCTATGTTATCTGAGCATAAAAATAATATAAATAATTGTAAAAATATTCCCCAAGAAGAA
TTACAAATTACTCAATGGATAAAAGAATGGCATGGAGAATTTTTGCTTGAAAGAGATAAT
AGATCAAAATTGCCAAAAAGTAAATGTAAAAATAATACATTATATGAAGCATGTGAGAA
GGAATGTATTGATCCATGTATGAAATATAGAGATTGGATTATTAGAAGTAAATTTGAATG
GCATACGTTATCGAAAGAATATGAAACTCAAAAAGTTCCAAAGGAAAATGCGGAAAATT
ATTTAATCAAAATTTCAGAAAACAAGAATGATGCTAAAGTAAGTTTATTATTGAATAATT
GTGATGCTGAATATTCAAAATATTGTGATTGTAAACATACTACTACTCTCGTTAAAAGCGT
TTTAAATGGTAACGACAATACAATTAAGGAAAAGCGTGAACATATTGATTTAGATGATTT
TTCTAAATTTGGATGTGATAAAAATTCCGTTGATACAAACACAAAGGTGTGGGAATGTAA
AAAACCTTATAAATTATCCACTAAAGATGTATGTGTACCTCCGAGGAGGCAAGAATTATG
TCTTGGAAACATTGATAGAATATACGATAAAAACCTATTAATGATAAAAGAGCATATTCT
TGCTATTGCAATATATGAATCAAGAATATTGAAACGAAAATATAAGAATAAAGATGATAA

AGAAGTTTGTAAAATCATAAATAAAACTTTCGCTGATATAAGAGATATTATAGGAGGTAC
TGATTATTGGAATGATTTGAGCAATAGAAAATTAGTAGGAAAAATTAACACAAATTCAAA
TTATGTTCACAGGAATAAAGAAAATGATAAGCTTTTTCGTGATGCGTGGTGGAAAGTTAT
TAAAAAAGATGTATGGAATGTGATATCATGGGTATTCAAGGATAAAACTGTTTGTAAAGA
AGATGATATTGAAAATATACCACAATTCTTCAGATGGTTTAGTGAATGGGGTGATGATTA
TTGCCAGGATAAAACAAAAATG
>Thailand 2006 Th99_EBA-175DQ092085.1
TATGTATGTATTCCTGATCGTAGAATCCAATTATGCATTGTTAATCTTAGCATTATTAAAA
CATATACAAAAGAGACCATGAAGGATCATTTCATTGAAGCCTCTAAAAAAGAATCTCAA
CTTTTGCTTAAAAAAAAATGATAACAAATATAATTCTAAATTTTGTAATGATTTGAAGAATA
GTTTTTTAGATTATGGACATCTTGCTATGGGAAATGATATGGATTTTGGAGGTTATTCAAC
TAAGGCAGAAAACAAAATTCAAGAAGTTTTTAAAGGGGCTCATGGGAAAATAAGTGAAC
ATGAAATTAAAAATTTTAGAAAAGAATGGTGGAATGAATTTAGAGAGAAACTTTGGGAA
GCTATGTTATCTGAGCATAAAAATAATATAAATAATTGTAAAAATATTCCCCAAGAAGAA
TTACAAATTACTCAATGGATAAAAGAATGGCATGGAGAATTTTTGCTTGAAAGAGATAAT
AGATCAAAATTGCCAAAAAGTAAATGTAAAAATAATACATTATATGAAGCATGTGAGAA
GGAATGTATTGATCCATGTATGAAATATAGAGATTGGATTATTAGAAGTAAATTTGAATG
GCATACGTTATCGAAAGAATATGAAACTCAAAATGTTTCAAAGGAAAATGCGGAAAATT
ATTTAATCAAAATTTCAGAAAAAATGAATGATGCTAAAGTAAGTTTATTATTGAATAATTG
TGATGCTGAATATTCAAAATATTGTGATTGTAAACATACTACTACTCTCGTTAAAAGCGTT
TTAAATGGTAACGACAATACAATTAAGGAAAAGCGTGAACATATTGATTTAGATGATTTT
TCTAAATTTGGATGTGATAAAAATTCCGTTGATACAAACACAAAGGTGTGGGAATGTAAA
AAACCTTATAAATTATCCACTAAAGATGTATGTGTACCTCCGAGGAGGCAAGAATTATGT
CTTGGAAACATTGATAGAATATACGATAAAAACCTATTAATGATAAAAGAGCATATTCTT
GCTATTGCAATATATGAATCAAGAATATTGAAACGAAAATATAAGAATAAAGATGATAA
AGAAGTTTGTAAAATCATAAATAAAACTTTCGCTGATATAAGAGATATTATAGGAGGTAC
TGATTATTGGAATGATTTGAGCAATAGAAAATTAGTAGGAAAAATTAACACAAATTCAAA
TTATGTTCACAGGAATAAAGAAAATGATAAGCTTTTTCGTGATGCGTGGTGGAAAGTTAT
TAAAAAAGATGTATGGAATGTGATATCATGGGTATTCAAGGATAAAACTGTTTGTAAAGA
AGATGATATTGAAAATATACCACAATTCTTCAGATGGTTTAGTGAATGGGGTGATGATTA
TTGCCAGGATAAAACAAAAATG
>Thailand 2006 Th98_EBA-175DQ092084.1
TATGTATGTATTCCTGATCGTAGAATCCAATTATGCATTGTTAATCTTAGCATTATTAAAA
CATATACAAAAGAGACCATGAAGGATCATTTCATTGAAGCCTCTAAAAAAGAATCTCAA
CTTTTGCTTAAAAAAAAATGATAACAAATATAATTCTAAATTTTGTAATGATTTGAAGAATA
GTTTTTTAGATTATGGACATCTTGCTATGGGAAATGATATGGATTTTGGAGGTTATTCAAC
TAAGGCAGAAAACAAAATTCAAGAAGTTTTTAAAGGGGCTCATGGGAAAATAAGTGAAC
ATGAAATTAAAAATTTTAGAAAAAAAATGGTGGAATGAATTTAGAGAGAAACTTTGGGAA
GCTATGCTATCTGAGCATAAAAATAATATAAATAATTGTAAAAATATTCCCCAAGAAGAA
TTACAAATTACTCAATGGATAAAAGAATGGCATGGAGAATTTTTGCTTGAAAGATATAAT
AGATCAAAATTGCCAAAAAGTAAATGTAAAAATAATACATTATATGAAGCATGTGAGAA
GGAATGTATTGATCCATGTATGAAATATAGAGATTGGATTATTAGAAGTAAATTTGAATG
GCATACGTTATCGAAAGAATATGAAACTCAAAATGTTTCAAAGGAAAATGCGGAAAATT
ATTTAATCAAAATTTCAAAAAAACAAGAATGATGCTAAAGTAAGTTTATTATTGAATAATTG

TGATGCTGAATATTCAAAATATTGTGATTGTAAACATACTACTACTCTCGTTAAAAGCGTT
TTAAATGGTAACGACAATACAATTAAGGAAAAGCGTGAACATATTGATTTAGATGATTTT
TCTAAATTTGGATGTGATAAAAATTCCGTTGATACAAACACAAAGGTGTGGGAATGTAAA
AAACCTTATATATTATCCACTAAAGATGTATGTGTACCTCCGAGGAGGCAAGAATTATGT
CTTGGAAACATTGATAGAATATACGATAAAAACCTATTAATGATAAAAGAGCATATTCTT
GCTATTGCAATATATGAATCAAGAATATTGAAACGAAAATATAAGAATAAAGATGATAA
AGAAGTTTGTAAAATCATAAATAAAACTTTCGCTGATATAAGAGATATTATAGGAGGTAC
TGATTATTGGAATGATTTGAGCAATAGAAAATTAGTAGGAAAAATTAACACAAATTCAAA
TTATGTTCACAGGAATAAAAAAAATGATAAGCTTTTTCGTGATGAGTGGTGGAAAGTTAT
TAAAAAAGATGTATGGAATGTGATATCATGGGTATTCAAGGATAAAACTGTTTGTAAAGA
AGATGATATTGAAAATATACCACAATTCTTCAGATGGTTTAGTGAATGGGGTGATGATTA
TTGCCAGGATAAAACAAAAATG
>Thailand 2006 Th97_EBA-175DQ092083.1
TATGTATGTATTCCTGATCGTAGAATCCAATTATGCATTGTTAATCTTAGCATTATTAAAA
CATATACAAAAGAGACCATGAAGGATCATTTCATTGAAGCCTCTAAAAAAGAATCTCAA
CTTTTGCTTAAAAAAAATGATAACAAATATAATTCTAAATTTTGTAATGATTTGAAGAATA
GTTTTTTAGATTATGGACATCTTGCTATGGGAAATGATATGGATTTTGGAGGTTATTCAAC
TAAGGCAGAAAACAAAATTCAAGAAGTTTTTAAAGGGGCTCATGGGGAAATAAGTGAAC
ATGAAATTAAAAATTTTAGAAAAAAATGGTGGAATGAATTTAGAGAGAAACTTTGGGAA
GCTATGTTATCTGAGCATAAAAATAATATAAATAATTGTAAAAATATTCCCCAAGAAGAA
TTACAAATTACTCAATGGATAAAAGAATGGCATGGAGAATTTTTGCTTGAAAGAGATAAT
AGATCAAAATTGCCAAAAAGTAAATGTAAAAATAATACATTATATGAAGCATGTGAGAA
GGAATGTATTGATCCATGTATGAAATATAGAGATTGGATTATTAGAAGTAAATTTGAATG
GCATACGTTATCGAAAGAATATGAAACTCAAAAAGTTTCAAAGGAAAATGCGGAAAATT
ATTTAATCAAAATTTCAGAAAACAAGAATGATGCTAAAGTAAGTTTATTATTGAATAATT
GTGATGCTGAATATTCAAAATATTGTGATTGTAAACATACTACTACTCTCGTTAAAAGCGT
TTTAAATGGTAACGACAATACAATTAAGGAAAAGCGTGAACATATTGATTTAGATGATTT
TTCTAAATTTGGATGTGATAAAAATTCCGTTGATACAAACACAAAGGTGTGGGAATGTAA
AAAACCTTATATATTATCCACTAAAGATGTATGTGTACCTCCGAGGAGGCAAGAATTATG
TCTTGGAAACATTGATAGAATATACGATAAAAACCTATTAATGATAAAAGAGCATATTCT
TGCTATTGCAATATATGAATCAAGAATATTGAAACGAAAATATAAGAATAAAGATGATAA
AGAAGTTTGTAAAATCATAAATAAAACTTTCGCTGATATAAGAGATATTATAGGAGGTAC
TGATTATTGGAATGATTTGAGCAATAGAAAATTAGTAGGAAAAATTAACACAAATTCAAA
ATATGTTCACAGGAATAAAAAAAATGATAAGCTTTTTCGTGATGAGTGGTGGAAAGTTAT
TAAAAAAGATGTATGGAATGTGATATCATGGGTATTCAAGGATAAAACTGTTTGTAAAGA
AGATGATATTGAAAATATACCACAATTCTTCAGATGGTTTAGTGAATGGGGTGATGATTA
TTGCCAGGATAAAACAAAAATG
>Thailand 2006 Th95_EBA-175DQ092082.1
TATGTATGTATTCCTGATCGTAGAATCCAATTATGCATTGTTAATCTTAGCATTATTAAAA
CATATACAAAAGAGACCATGAAGGATCATTTCATTGAAGCCTCTAAAAAAGAATCTCAA
CTTTTGCTTAAAAAAAATGATAACAAATATAATTCTAAATTTTGTAATGATTTGAAGAATA
GTTTTTTAGATTATGGACATCTTGCTATGGGAAATGATATGGATTTTGGAGGTTATTCAAC
TAAGGCAGAAAACAAAATTCAAGAAGTTTTTAAAGGGGCTCATGGGAAAATAAGTGAAC
ATGAAATTAAAAATTTTAGAAAAGAATGGTGGAATGAATTTAGAGAGAAACTTTGGGAA

GCTATGTTATCTGAGCATAAAAATAATATAAATAATTGTAAAAATATTCCCCAAGAAGAA
TTACAAATTACTCAATGGATAAAAGAATGGCATGGAGAATTTTTGCTTGAAAGAGATAAT
AGATCAAAATTGCCAAAAAGTAAATGTAAAAATAATACATTATATGAAGCATGTGAGAA
GGAATGTATTGATCCATGTATGAAATATAGAGATTGGATTATTAGAAGTAAATTTGAATG
GCATACGTTATCGAAAGAATATGAAACTCAAAATGTTTCAAAGGAAAATGCGGAAAATT
ATTTAATCAAAATTTCAGAAAAAATGAATGATGCTAAAGTAAGTTTATTATTGAATAATTG
TGATGCTGAATATTCAAAATATTGTGATTGTAAACATACTACTACTCTCGTTAAAAGCGTT
TTAAATGGTAACGACAATACAATTAAGGAAAAGCGTGAACATATTGATTTAGATGATTTT
TCTAAATTTGGATGTGATAAAAATTCCGTTGATACAAACACAAAGGTGTGGGAATGTAAA
AAACCTTATAAATTATCCACTAAAGATGTATGTGTACCTCCGAGGAGGCAAGAATTATGT
CTTGGAAACATTGATAGAATATACGATAAAAACCTATTAATGATAAAAGAGCATATTCTT
GCTATTGCAATATATGAATCAAGAATATTGAAACGAAAATATAAGAATAAAGATGATAA
AGAAGTTTGTAAAATCATAAATAAAACTTTCGCTGATATAAGAGATATTATAGGAGGTAC
TGATTATTGGAATGATTTGAGCAATAGAAAATTAGTAGGAAAAATTAACACAAATTCAAA
TTATGTTCACAGGAATAAAGAAAATGATAAGCTTTTTCGTGATGCGTGGTGGAAAGTTAT
TAAAAAAGATGTATGGAATGTGATATCATGGGTATTCAAGGATAAAACTGTTTGTAAAGA
AGATGATATTGAAAATATACCACAATTCTTCAGATGGTTTAGTGAATGGGGTGATGATTA
TTGCCAGGATAAAACAAAAATG
>Thailand 2006 Th94_EBA-175DQ092081.1
TATGTATGTATTCCTGATCGTAGAATCCAATTATGCATTGTTAATCTTAGCATTATTAAAA
CATATACAAAAGAGACCATGAAGGATCATTTCATTGAAGCCTCTAAAAAAGAATCTCAA
CTTTTGCTTAAAAAAAATGATAACAAATATAATTCTAAATTTTGTAATGATTTGAAGAATA
GTTTTTTAGATTATGGACATCTTGCTATGGGAAATGATATGGATTTTGGAGGTTATTCAAC
TAAGGCAGAAAACAAAATTCAAGAAGTTTTTAAAGGGGCTCATGGGGAAAAAAGTGAA
CATGAAATTAAAAATTTTAGAAAAAAATGGTGGAATGAATTTAGAGAGAAACTTTGGGA
AGCTATGTTATCTGAGCATAAAAATAATATAAATAATTGTAAAAATATTCCCCAAGAAGA
ATTACAAATTACTCAATGGATAAAAGAATGGCATGGAGAATTTTTGCTTGAAAGAGATAA
TAGATCAAAATTGCCAAAAAGTAAATGTAAAAATAATACATTATATGAAGCATGTGAGA
AGGAATGTATTGATCCATGTATGAAATATAGAGATTGGATTATTAGAAGTAAATTTGAAT
GGCATACGTTATCGAAAGAATATGAAACTCAAAAAGTTCCAAAGGAAAATGCGGAAAAT
TATTTAATCAAAATTTCAGAAAACAAGAATGATGCTAAAGTAAGTTTATTATTGAATAATT
GTGATGCTGAATATTCAAAATATTGTGATTGTAAACATACTACTACTCTCGTTAAAAGCGT
TTTAAATGGTAACGACAATACAATTAAGGAAAAGCGTGAACATATTGATTTAGATGATTT
TTCTAAATTTGGATGTGATAAAAATTCCGTTGATACAAACACAAAGGTGTGGGAATGTAA
AAAACCTTATAAATTATCCACTAAAGATGTATGTGTACCTCCGAGGAGGCAAGAATTATG
TCTTGGAAACATTGATAGAATATACGATAAAAACCTATTAATGATAAAAGAGCATATTCT
TGCTATTGCAATATATGAATCAAGAATATTGAAACGAAAATATAAGAATAAAGATGATAA
AGAAGTTTGTAAAATCATAAATAAAACTTTCGCTGATATAAGAGATATTATAGGAGGTAC
TGATTATTGGAATGATTTGAGCAATAGAAAATTAGTAGGAAAAATTAACACAAATTCAAA
TTATGTTCACAGGAATAAACAAAATGATAAGCTTTTTCGTGATGAGTGGTGGAAAGTTAT
TAAAAAAGATGTATGGAATGTGATATCATGGGTATTCAAGGATAAAACTGTTTGTAAAGA
AGATGATATTGAAAATATACCACAATTCTTCAGATGGTTTAGTGAATGGGGTGATGATTA
TTGCCAGGATAAAACAAAAATG
>Thailand 2006 Th93_EBA-175DQ092080.1

TATGTATGTATTCCTGATCGTAGAATCCAATTATGCATTGTTAATCTTAGCATTATTAAAA
CATATACAAAAGAGACCATGAAGGATCATTTCATTGAAGCCTCTAAAAAAGAATCTCAA
CTTTTGCTTAAAAAAAAATGATAACAAATATAATTCTAAATTTTGTAATGATTTGAAGAATA
GTTTTTTAGATTATGGACATCTTGCTATGGGAAATGATATGGATTTTGGAGGTTATTCAAC
TAAGGCAGAAAACAAAATTCAAGAAGTTTTTAAAGGGGCTCATGGGAAAATAAGTGAAC
ATGAAATTAAAAATTTTAGAAAAAAAATGGTGGAATGAATTTAGAGAGAAACTTTGGGAA
GCTATGTTATCTGAGCATAAAAATAATATAAATAATTGTAAAAATATTCCCCAAGAAGAA
TTACAAATTACTCAATGGATAAAAGAATGGCATGGAGAATTTTTGCTTGAAAGAGATAAT
AGATCAAAATTGCCAAAAAGTAAATGTAAAAATAATACATTATATGAAGCATGTGAGAA
GGAATGTATTGATCCATGTATGAAATATAGAGATTGGATTATTAGAAGTAAATTTGAATG
GCATACGTTATCGAAAGAATATGAAACTCAAAATGTTTCAAAGGAAAATGCGGAAAATT
ATTTAATCAAAATTTCAGAAAAAAATGAATGATGCTAAAGTAAGTTTATTATTGAATAATTG
TGATGCTGAATATTCAAAATATTGTGATTGTAAACATACTACTACTCTCGTTAAAAGCGTT
TTAAATGGTAACGACAATACAATTAAGGAAAAGCGTGAACATATTGATTTAGATGATTTT
TCTAAATTTGGATGTGATAAAAATTCCGTTGATACAAACACAAAGGTGTGGGAATGTAAA
AAACCTTATAAATTATCCACTAAAGATGTATGTGTACCTCCGAGGAGGCAAGAATTATGT
CTTGGAAACATTGATAGAATATACGATAAAAACCTATTAATGATAAAAGAGCATATTCTT
GCTATTGCAATATATGAATCAAGAATATTGAAACGAAAATATAAGAATAAAGATGATAA
AGAAGTTTGTAAAATCATAAATAAAACTTTCGCTGATATAAGAGATATTATAGGAGGTAC
TGATTATTGGAATGATTTGAGCAATAGAAAATTAGTAGGAAAAATTAACACAAATTCAAA
TTATGTTCACAGGAATAAAGAAAATGATAAGCTTTTTCGTGATGCGTGGTGGAAAGTTAT
TAAAAAAGATGTATGGAATGTGATATCATGGGTATTCAAGGATAAAACTGTTTGTAAAGA
AGATGATATTGAAAATATACCACAATTCTTCAGATGGTTTAGTGAATGGGGTGATGATTA
TTGCCAGGATAAAACAAAAATG
>Thailand 2006 Th91_EBA-175DQ092079.1
TATGTATGTATTCCTGATCGTAGAATCCAATTATGCATTGTTAATCTTAGCATTATTAAAA
CATATACAAAAGAGACCATGAAGGATCATTTCATTGAAGCCTCTAAAAAAGAATCTCAA
CTTTTGCTTAAAAAAAAATGATAACAAATATAATTCTAAATTTTGTAATGATTTGAAGAATA
GTTTTTTAGATTATGGACATCTTGCTATGGGAAATGATATGGATTTTGGAGGTTATTCAAC
TAAGGCAGAAAACAAAATTCAAGAAGTTTTTAAAGGGGCTCATGGGAAAATAAGTGAAC
ATGAAATTAAAAATTTTAGAAAAAAAATGGTGGAATGAATTTAGAGAGAAACTTTGGGAA
GCTATGTTATCTGAGCATAAAAATAATATAAATAATTGTAAAAATATTCCCCAAGAAGAA
TTACAAATTACTCAATGGATAAAAGAATGGCATGGAGAATTTTTGCTTGAAAGAGATAAT
AGATCAAAATTGCCAAAAAGTAAATGTAAAAATAATACATTATATGAAGCATGTGAGAA
GGAATGTATTGATCCATGTATGAAATATAGAGATTGGATTATTAGAAGTAAATTTGAATG
GCATACGTTATCGAAAGAATATGAAACTCAAAAAGTTCCAAAGGAAAATGCGGAAAATT
ATTTAATCAAAATTTCAGAAAACAAGAATGATGCTAAAGTAAGTTTATTATTGAATAATT
GTGATGCTGAATATTCAAAATATTGTGATTGTAAACATACTACTACTCTCGTTAAAAGCGT
TTTAAATGGTAACGACAATACAATTAAGGAAAAGCGTGAACATATTGATTTAGATGATTT
TTCTAAATTTGGATGTGATAAAAATTCCGTTGATACAAACACAAAGGTGTGGGAATGTAA
AAAACCTTATAAATTATCCACTAAAGATGTATGTGTACCTCCGAGGAGGCAAGAATTATG
TCTTGGAAACATTGATAGAATATACGATAAAAACCTATTAATGATAAAAGAGCATATTCT
TGCTATTGCAATATATGAATCAAGAATATTGAAACGAAAATATAAGAATAAAGATGATAA
AGAAGTTTGTAAAATCATAAATAAAACTTTCGCTGATATAAGAGATATTATAGGAGGTAC

TGATTATTGGAATGATTTGAGCAATAGAAAATTAGTAGGAAAAATTAACACAAATTCAAA
TTATGTTCACAGGAATAAAGAAAATGATAAGCTTTTTCGTGATGCGTGGTGGAAAGTTAT
TAAAAAAGATGTATGGAATGTGATATCATGGGTATTCAAGGATAAAACTGTTTGTAAAGA
AGATGATATTGAAAATATACCACAATTCTTCAGATGGTTTAGTGAATGGGGTGATGATTA
TTGCCAGGATAAAACAAAAATG
>Thailand 2006 Th89_EBA-175DQ092078.1
TATGTATGTATTCCTGATCGTAGAATCCAATTATGCATTGTTAATCTTAGCATTATTAAAA
CATATACAAAAGAGACCATGAAGGATCATTTCATTGAAGCCTCTAAAAAAGAATCTCAA
CTTTTGCTTAAAAAAAAATGATAACAAATATAATTCTAAATTTTGTAATGATTTGAAGAATA
GTTTTTTAGATTATGGACATCTTGCTATGGGAAATGATATGGATTTTGGAGGTTATTCAAC
TAAGGCAGAAAACAAAATTCAAGAAGTTTTTAAAGGGGCTCATGGGAAAATAAGTGAAC
ATGAAATTAAAAATTTTAGAAAAAAATGGTGGAATGAATTTAGAGAGAAACTTTGGGAA
GCTATGTTATCTGAGCATAAAAATAATATAAATAATTGTAAAAATATTCCCCAAGAAGAA
TTACAAATTACTCAATGGATAAAAGAATGGCATGGAGAATTTTTGCTTGAAAGAGATAAT
AGATCAAAATTGCCAAAAAGTAAATGTAAAAATAATACATTATATGAAGCATGTGAGAA
GGAATGTATTGATCCATGTATGAAATATAGAGATTGGATTATTAGAAGTAAATTTGAATG
GCATACGTTATCGAAAGAATATGAAACTCAAAATGTTTCAAAGGAAAATGCGGAAAATT
ATTTAATCAAAATTTCAGAAAAAATGAATGATGCTAAAGTAAGTTTATTATTGAATAATTG
TGATGCTGAATATTCAAAATATTGTGATTGTAAACATACTACTACTCTCGTTAAAAGCGTT
TTAAATGGTAACGACAATACAATTAAGGAAAAGCGTGAACATATTGATTTAGATGATTTT
TCTAAATTTGGATGTGATAAAAATTCCGTTGATACAAACACAAAGGTGTGGGAATGTAAA
AAACCTTATAAATTATCCACTAAAGATGTATGTGTACCTCCGAGGAGGCAAGAATTATGT
CTTGGAAACATTGATAGAATATACGATAAAAACCTATTAATGATAAAAGAGCATATTCTT
GCTATTGCAATATATGAATCAAGAATATTGAAACGAAAATATAAGAATAAAGATGATAA
AGAAGTTTGTAAAATCATAAATAAAACTTTCGCTGATATAAGAGATATTATAGGAGGTAC
TGATTATTGGAATGATTTGAGCAATAGAAAATTAGTAGGAAAAATTAACACAAATTCAAA
TTATGTTCACAGGAATAAAGAAAATGATAAGCTTTTTCGTGATGCGTGGTGGAAAGTTAT
TAAAAAAGATGTATGGAATGTGATATCATGGGTATTCAAGGATAAAACTGTTTGTAAAGA
AGATGATATTGAAAATATACCACAATTCTTCAGATGGTTTAGTGAATGGGGTGATGATTA
TTGCCAGGATAAAACAAAAATG
>Thailand 2006 Th86_EBA-175DQ092077.1
TATGTATGTATTCCTGATCGTAGAATCCAATTATGCATTGTTAATCTTAGCATTATTAAAA
CATATACAAAAGAGACCATGAAGGATCATTTCATTGAAGCCTCTAAAAAAGAATCTCAA
CTTTTGCTTAAAAAAAAATGATAACAAATATAATTCTAAATTTTGTAATGATTTGAAGAATA
GTTTTTTAGATTATGGACATCTTGCTATGGGAAATGATATGGATTTTGGAGGTTATTCAAC
TAAGGCAGAAAACAAAATTCAAGAAGTTTTTAAAGGGGCTCATGGGGAAAAAAGTGAA
CATGAAATTAAAAATTTTAGAAAAAAATGGTGGAATGAATTTAGAGAGAAACTTTGGGA
AGCTATGTTATCTGAGCATAAAAATAATATAAATAATTGTAAAAATATTCCCCAAGAAGA
ATTACAAATTACTCAATGGATAAAAGAATGGCATGGAGAATTTTTGCTTGAAAGAGATAA
TAGATCAAAATTGCCAAAAAGTAAATGTAAAAATAATACATTATATGAAGCATGTGAGA
AGGAATGTATTGATCCATGTATGAAATATAGAGATTGGATTATTAGAAGTAAATTTGAAT
GGCATACGTTATCGAAAGAATATGAAACTCAAAATGTTTCAAAGGAAAATGCGGAAAAT
TATTTAATCAAAATTTCAGAAAAAATGAATGATGCTAAAGTAAGTTTATTATTGAATAATT
GTGATGCTGAATATTCAAAATATTGTGATTGTAAACATACTACTACTCTCGTTAAAAGCGT

TTTAAATGGTAACGACAATACAATTAAGGAAAAGCGTGAACATATTGATTTAGATGATTT
TTCTAAATTTGGATGTGATAAAAATTCCGTTGATACAAACACAAAGGTGTGGGAATGTAA
AAAACCTTATAAATTATCCACTAAAGATGTATGTGTACCTCCGAGGAGGCAAGAATTATG
TCTTGGAAACATTGATAGAATATACGATAAAAACCTATTAATGATAAAAGAGCATATTCT
TGCTATTGCAATATATGAATCAAGAATATTGAAACGAAAATATAAGAATAAAGATGATAA
AGAAGTTTGTAAAATCATAAATAAAACTTTCGCTGATATAAGAGATATTATAGGAGGTAC
TGATTATTGGAATGATTTGAGCAATAGAAAATTAGTAGGAAAAATTAACACAAATTCAAA
TTATGTTCACAGGAATAAAGAAAATGATAAGCTTTTTCGTGATGCGTGGTGGAAAGTTAT
TAAAAAAGATGTATGGAATGTGATATCATGGGTATTCAAGGATAAAACTGTTTGTAAAGA
AGATGATATTGAAAATATACCACAATTCTTCAGATGGTTTAGTGAATGGGGTGATGATTA
TTGCCAGGATAAAACAAAAATG
>Thailand 2006 Th85_EBA-175DQ092076.1
TATGTATGTATTCCTGATCGTAGAATCCAATTATGCATTGTTAATCTTAGCATTATTAAAA
CATATACAAAAGAGACCATGAAGGATCATTTCATTGAAGCCTCTAAAAAAGAATCTCAA
CTTTTGCTTAAAAAAAAATGATAACAAATATAATTCTAAATTTTGTAATGATTTGAAGAATA
GTTTTTTAGATTATGGACATCTTGCTATGGGAAATGATATGGATTTTGGAGGTTATTCAAC
TAAGGCAGAAAACAAAATTCAAGAAGTTTTTAAAGGGGCTCATGGGAAAATAAGTGAAC
ATGAAATTAAAAATTTTAGAAAAAAATGGTGGAATGAATTTAGAGAGAAACTTTGGGAA
GCTATGTTATCTGAGCATAAAAATAATATAAATAATTGTAAAAATATTCCCCAAGAAGAA
TTACAAATTACTCAATGGATAAAAGAATGGCATGGAGAATTTTTGCTTGAAAGAGATAAT
AGATCAAAATTGCCAAAAAGTAAATGTAAAAATAATACATTATATGAAGCATGTGAGAA
GGAATGTATTGATCCATGTATGAAATATAGAGATTGGATTATTAGAAGTAAATTTGAATG
GCATACGTTATCGAAAGAATATGAAACTCAAAAAGTTCCAAAGGAAAATGCGGAAAATT
ATTTAATCAAAATTTCAGAAAACAAGAATGATGCTAAAGTAAGTTTATTATTGAATAATT
GTGATGCTGAATATTCAAAATATTGTGATTGTAAACATACTACTACTCTCGTTAAAAGCGT
TTTAAATGGTAACGACAATACAATTAAGGAAAAGCGTGAACATATTGATTTAGATGATTT
TTCTAAATTTGGATGTGATAAAAATTCCGTTGATACAAACACAAAGGTGTGGGAATGTAA
AAAACCTTATAAATTATCCACTAAAGATGTATGTGTACCTCCGAGGAGGCAAGAATTATG
TCTTGGAAACATTGATAGAATATACGATAAAAACCTATTAATGATAAAAGAGCATATTCT
TGCTATTGCAATATATGAATCAAGAATATTGAAACGAAAATATAAGAATAAAGATGATAA
AGAAGTTTGTAAAATCATAAATAAAACTTTCGCTGATATAAGAGATATTATAGGAGGTAC
TGATTATTGGAATGATTTGAGCAATAGAAAATTAGTAGGAAAAATTAACACAAATTCAAA
TTATGTTCACAGGAATAAACAAAATGATAAGCTTTTTCGTGATGAGTGGTGGAAAGTTAT
TAAAAAAGATGTATGGAATGTGATATCATGGGTATTCAAGGATAAAACTGTTTGTAAAGA
AGATGATATTGAAAATATACCACAATTCTTCAGATGGTTTAGTGAATGGGGTGATGATTA
TTGCCAGGATAAAACAAAAATG
>Thailand 2006 Th83_EBA-175DQ092075.1
TATGTATGTATTCCTGATCGTAGAATCCAATTATGCATTGTTAATCTTAGCATTATTAAAA
CATATACAAAAGAGACCATGAAGGATCATTTCATTGAAGCCTCTAAAAAAGAATCTCAA
CTTTTGCTTAAAAAAAAATGATAACAAATATAATTCTAAATTTTGTAATGATTTGAAGAATA
GTTTTTTAGATTATGGACATCTTGCTATGGGAAATGATATGGATTTTGGAGGTTATTCAAC
TAAGGCAGAAAACAAAATTCAAGAAGTTTTTAAAGGGGCTCATGGGAAAATAAGTGAAC
ATGAAATTAAAAATTTTAGAAAAAAATGGTGGAATGAATTTAGAGAGAAACTTTGGGAA
GCTATGTTATCTGAGCATAAAAATAATATAAATAATTGTAAAAATATTCCCCAAGAAGAA

TTACAAATTACTCAATGGATAAAAGAATGGCATGGAGAATTTTTGCTTGAAAGAGATAAT
AGATCAAAATTGCCAAAAAGTAAATGTAAAAATAATACATTATATGAAGCATGTGAGAA
GGAATGTATTGATCCATGTATGAAATATAGAGATTGGATTATTAGAAGTAAATTTGAATG
GCATACGTTATCGAAAGAATATGAAACTCAAAAAGTTCCAAAGGAAAATGCGGAAAATT
ATTTAATCAAAATTTCAGAAAACAAGAATGATGCTAAAGTAAGTTTATTATTGAATAATT
GTGATGCTGAATATTCAAAATATTGTGATTGTAAACATACTACTACTCTCGTTAAAAGCGT
TTTAAATGGTAACGACAATACAATTAAGGAAAAGCGTGAACATATTGATTTAGATGATTT
TTCTAAATTTGGATGTGATAAAAAATTCCGTTGATACAAACACAAAGGTGTGGGAATGTAA
AAAACCTTATAAATTATCCACTAAAGATGTATGTGTACCTCCGAGGAGGCAAGAATTATG
TCTTGGAAACATTGATAGAATATACGATAAAAACCTATTAATGATAAAAGAGCATATTCT
TGCTATTGCAATATATGAATCAAGAATATTGAAACGAAAATATAAGAATAAAGATGATAA
AGAAGTTTGTAAAATCATAAATAAAACTTTCGCTGATATAAGAGATATTATAGGAGGTAC
TGATTATTGGAATGATTTGAGCAATAGAAAATTAGTAGGAAAAATTAACACAAATTCAAA
TTATGTTCACAGGAATAAAGAAAATGATAAGCTTTTTCGTGATGCGTGGTGGAAAGTTAT
TAAAAAAGATGTATGGAATGTGATATCATGGGTATTCAAGGATAAAACTGTTTGTAAAGA
AGATGATATTGAAAATATACCACAATTCTTCAGATGGTTTAGTGAATGGGGTGATGATTA
TTGCCAGGATAAAACAAAAATG
>Thailand 2006 Th80_EBA-175DQ092074.1
TATGTATGTATTCCTGATCGTAGAATCCAATTATGCATTGTTAATCTTAGCATTATTAAAA
CATATACAAAAGAGACCATGAAGGATCATTTCATTGAAGCCTCTAAAAAAGAATCTCAA
CTTTTGCTTAAAAAAAAATGATAACAAATATAATTCTAAATTTTGTAATGATTTGAAGAATA
GTTTTTTAGATTATGGACATCTTGCTATGGGAAATGATATGGATTTTGGAGGTTATTCAAC
TAAGGCAGAAAACAAAATTCAAGAAGTTTTTAAAGGGGCTCATGGGAAAATAAGTGAAC
ATGAAATTAAAAATTTTAGAAAAAAAATGGTGGAATGAATTTAGAGAGAAACTTTGGGAA
GCTATGCTATCTGAGCATAAAAATAATATAAATAATTGTAAAAATATTCCCCAAGAAGAA
TTACAAATTACTCAATGGATAAAAGAATGGCATGGAGAATTTTTGCTTGAAAGATATAAT
AGATCAAAATTGCCAAAAAGTAAATGTAAAAATAATACATTATATGAAGCATGTGAGAA
GGAATGTATTGATCCATGTATGAAATATAGAGATTGGATTATTAGAAGTAAATTTGAATG
GCATACGTTATCGAAAGAATATGAAACTCAAAATGTTTCAAAGGAAAATGCGGAAAATT
ATTTAATCAAAATTTCAAAAAACAAGAATGATGCTAAAGTAAGTTTATTATTGAATAATTG
TGATGCTGAATATTCAAAATATTGTGATTGTAAACATACTACTACTCTCGTTAAAAGCGTT
TTAAATGGTAACGACAATACAATTAAGGAAAAGCGTGAACATATTGATTTAGATGATTTT
TCTAAATTTGGATGTGATAAAAAATTCCGTTGATACAAACACAAAGGTGTGGGAATGTAAA
AAACCTTATATATTATCCACTAAAGATGTATGTGTACCTCCGAGGAGGCAAGAATTATGT
CTTGGAAACATTGATAGAATATACGATAAAAACCTATTAATGATAAAAGAGCATATTCTT
GCTATTGCAATATATGAATCAAGAATATTGAAACGAAAATATAAGAATAAAGATGATAA
AGAAGTTTGTAAAATCATAAATAAAACTTTCGCTGATATAAGAGATATTATAGGAGGTAC
TGATTATTGGAATGATTTGAGCAATAGAAAATTAGTAGGAAAAATTAACACAAATTCAAA
TTATGTTCACAGGAATAAAAAAAATGATAAGCTTTTTCGTGATGAGTGGTGGAAAGTTAT
TAAAAAAGATGTATGGAATGTGATATCATGGGTATTCAAGGATAAAACTGTTTGTAAAGA
AGATGATATTGAAAATATACCACAATTCTTCAGATGGTTTAGTGAATGGGGTGATGATTA
TTGCCAGGATAAAACAAAAATG
>Thailand 2006 Th74_EBA-175DQ092073.1
TATGTATGTATTCCTGATCGTAGAATCCAATTATGCATTGTTAATCTTAGCATTATTAAAA

CATATACAAAAGAGACCATGAAGGATCATTTCATTGAAGCCTCTAAAAAAGAATCTCAA
CTTTTGCTTAAAAAAAAATGATAACAAATATAATTCTAAATTTTGTAATGATTTGAAGAATA
GTTTTTTAGATTATGGACATCTTGCTATGGGAAATGATATGGATTTTGGAGGTTATTCAAC
TAAGGCAGAAAACAAAATTCAAGAAGTTTTTAAAGGGGCTCATGGGGAAATAAGTGAAC
ATAAAATTAAAAATTTTAGAAAAGAATGGTGGAATGAATTTAGAGAGAAACTTTGGGAA
GCTATGTTATCTGAGCATAAAAATAATATAAATAATTGTAAAAATATTCCCCAAGAAGAA
TTACAAATTACTCAATGGATAAAAGAATGGCATGGAGAATTTTTGCTTGAAAGAGATAAT
AGATCAAAATTGCCAAAAAGTAAATGTAAAAATAATACATTATATGAAGCATGTGAGAA
GGAATGTATTGATCCATGTATGAAATATAGAGATTGGATTATTAGAAGTAAATTTGAATG
GCATACGTTATCGAAAGAATATGAAACTCAAAAAGTTCCAAAGGAAAATGCGGAAAATT
ATTTAATCAAAATTTCAGAAAACAAGAATGATGCTAAAGTAAGTTTATTATTGAATAATT
GTGATGCTGAATATTCAAAATATTGTGATTGTAAACATACTACTACTCTCGTTAAAAGCGT
TTTAAATGGTAACGACAATACAATTAAGGAAAAGCGTGAACATATTGATTTAGATGATTT
TTCTAAATTTGGATGTGATAAAAATTCCGTTGATACAAACACAAAGGTGTGGGAATGTAA
AAAACCTTATAAATTATCCACTAAAGATGTATGTGTACCTCCGAGGAGGCAAGAATTATG
TCTTGGAAACATTGATAGAATATACGATAAAAACCTATTAATGATAAAAGAGCATATTCT
TGCTATTGCAATATATGAATCAAGAATATTGAAACGAAAATATAAGAATAAAGATGATAA
AGAAGTTTGTAAAATCATAAATAAAACTTTCGCTGATATAAGAGATATTATAGGAGGTAC
TGATTATTGGAATGATTTGAGCAATAGAAAATTAGTAGGAAAAATTAACACAAATTCAAA
TTATGTTCACAGGAATAAACAAAATGATAAGCTTTTTCGTGATGAGTGGTGGAAAGTTAT
TAAAAAAGATGTATGGAATGTGATATCATGGGTATTCAAGGATAAAACTGTTTGTAAAGA
AGATGATATTGAAAATATACCACAATTCTTCAGATGGTTTAGTGAATGGGGTGATGATTA
TTGCCAGGATAAAACAAAAATG
>Thailand 2006 Th72_EBA-175DQ092072.1
TATGTATGTATTCCTGATCGTAGAATCCAATTATGCATTGTTAATCTTAGCATTATTAAAA
CATATACAAAAGAGACCATGAAGGATCATTTCATTGAAGCCTCTAAAAAAGAATCTCAA
CTTTTGCTTAAAAAAAAATGATAACAAATATAATTCTAAATTTTGTAATGATTTGAAGAATA
GTTTTTTAGATTATGGACATCTTGCTATGGGAAATGATATGGATTTTGGAGGTTATTCAAC
TAAGGCAGAAAACAAAATTCAAGAAGTTTTTAAAGGGGCTCATGGGGAAAAAAGTGAA
CATGAAATTAAAAATTTTAGAAAAAAAATGGTGGAATGAATTTAGAGAGAAACTTTGGGA
AGCTATGTTATCTGAGCATAAAAATAATATAAATAATTGTAAAAATATTCCCCAAGAAGA
ATTACAAATTACTCAATGGATAAAAGAATGGCATGGAGAATTTTTGCTTGAAAGAGATAA
TAGATCAAAATTGCCAAAAAGTAAATGTAAAAATAATACATTATATGAAGCATGTGAGA
AGGAATGTATTGATCCATGTATGAAATATAGAGATTGGATTATTAGAAGTAAATTTGAAT
GGCATACGTTATCGAAAGAATATGAAACTCAAAAAGTTTCAAAGGAAAATGCGGAAAAT
TATTTAATCAAAATTTCAAAAAACAAGAATGATGCTAAAGTAAGTTTATTATTGAATAATT
GTGATGCTGAATATTCAAAATATTGTGATTGTAAACATACTACTACTCTCGTTAAAAGCGT
TTTAAATGGTAACGACAATACAATTAAGGAAAAGCGTGAACATATTGATTTAGATGATTT
TTCTAAATTTGGATGTGATAAAAATTCCGTTGATACAAACACAAAGGTGTGGGAATGTAA
AAACCCTTATATATTATCCACTAAAGATGTATGTGTACCTCCGAGGAGGCAAGAATTATG
TCTTGGAAACATTGATAGAATATACGATAAAAACCTATTAATGATAAAAGAGCATATTCT
TGCTATTGCAATATATGAATCAAGAATATTGAAACGAAAATATAAGAATAAAGATGATAA
AGAAGTTTGTAAAATCATAAATAAAACTTTCGCTGATATAAGAGATATTATAGGAGGTAC
TGATTATTGGAATGATTTGAGCAATAGAAAATTAGTAGGAAAAATTAACACAAATTCAAA

TTATGTTCACAGGAATAAAAAAAATGATAAGCTTTTTCGTGATGAGTGGTGGAAAGTTAT
TAAAAAAGATGTATGGAATGTGATATCATGGGTATTCAAGGATAAAACTGTTTGTAAAGA
AGATGATATTGAAAATATACCACAATTCTTCAGATGGTTTAGTGAATGGGGTGATGATTA
TTGCCAGGATAAAACAAAAATG
>Thailand 2006 Th71_EBA-175DQ092071.1
TATGTATGTATTCCTGATCGTAGAATCCAATTATGCATTGTTAATCTTAGCATTATTAAAA
CATATACAAAAGAGACCATGAAGGATCATTTCATTGAAGCCTCTAAAAAAGAATCTCAA
CTTTTGCTTAAAAAAAAATGATAACAAATATAATTCTAAATTTTGTAATGATTTGAAGAATA
GTTTTTTAGATTATGGACATCTTGCTATGGGAAATGATATGGATTTTGGAGGTTATTCAAC
TAAGGCAGAAAACAAAATTCAAGAAGTTTTTAAAGGGGCTCATGGGAAAATAAGTGAAC
ATGAAATTAAAAATTTTAGAAAAGAATGGTGGAATGAATTTAGAGAGAAACTTTGGGAA
GCTATGTTATCTGAGCATAAAAATAATATAAATAATTGTAAAAATATTCCCCAAGAAGAA
TTACAAATTACTCAATGGATAAAAGAATGGCATGGAGAATTTTTGCTTGAAAGAGATAAT
AGATCAAAATTGCCAAAAAGTAAATGTAAAAATAATACATTATATGAAGCATGTGAGAA
GGAATGTATTGATCCATGTATGAAATATAGAGATTGGATTATTAGAAGTAAATTTGAATG
GCATACGTTATCGAAAGAATATGAAACTCAAAATGTTTCAAAGGAAAATGCGGAAAATT
ATTTAATCAAAATTTCAGAAAAAATGAATGATGCTAAAGTAAGTTTATTATTGAATAATTG
TGATGCTGAATATTCAAAATATTGTGATTGTAAACATACTACTACTCTCGTTAAAAGCGTT
TTAAATGGTAACGACAATACAATTAAGGAAAAGCGTGAACATATTGATTTAGATGATTTT
TCTAAATTTGGATGTGATAAAAATTCCGTTGATACAAACACAAAGGTGTGGGAATGTAAA
AAACCTTATAAATTATCCACTAAAGATGTATGTGTACCTCCGAGGAGGCAAGAATTATGT
CTTGGAAACATTGATAGAATATACGATAAAAACCTATTAATGATAAAAGAGCATATTCTT
GCTATTGCAATATATGAATCAAGAATATTGAAACGAAAATATAAGAATAAAGATGATAA
AGAAGTTTGTAAAATCATAAATAAAACTTTCGCTGATATAAGAGATATTATAGGAGGTAC
TGATTATTGGAATGATTTGAGCAATAGAAAATTAGTAGGAAAAATTAACACAAATTCAAA
TTATGTTCACAGGAATAAAGAAAATGATAAGCTTTTTCGTGATGCGTGGTGGAAAGTTAT
TAAAAAAGATGTATGGAATGTGATATCATGGGTATTCAAGGATAAAACTGTTTGTAAAGA
AGATGATATTGAAAATATACCACAATTCTTCAGATGGTTTAGTGAATGGGGTGATGATTA
TTGCCAGGATAAAACAAAAATG
>Thailand 2006 Th65_EBA-175DQ092070.1
TATGTATGTATTCCTGATCGTAGAATCCAATTATGCATTGTTAATCTTAGCATTATTAAAA
CATATACAAAAGAGACCATGAAGGATCATTTCATTGAAGCCTCTAAAAAAGAATCTCAA
CTTTTGCTTAAAAAAAAATGATAACAAATATAATTCTAAATTTTGTAATGATTTGAAGAATA
GTTTTTTAGATTATGGACATCTTGCTATGGGAAATGATATGGATTTTGGAGGTTATTCAAC
TAAGGCAGAAAACAAAATTCAAGAAGTTTTTAAAGGGGCTCATGGGAAAATAAGTGAAC
ATGAAATTAAAAATTTTAGAAAAAAAATGGTGGAATGAATTTAGAGAGAAACTTTGGGAA
GCTATGTTATCTGAGCATAAAAATAATATAAATAATTGTAAAAATATTCCCCAAGAAGAA
TTACAAATTACTCAATGGATAAAAGAATGGCATGGAGAATTTTTGCTTGAAAGAGATAAT
AGATCAAAATTGCCAAAAAGTAAATGTAAAAATAATACATTATATGAAGCATGTGAGAA
GGAATGTATTGATCCATGTATGAAATATAGAGATTGGATTATTAGAAGTAAATTTGAATG
GCATACGTTATCGAAAGAATATGAAACTCAAAAAGTTTCAAAGGAAAATGCGGAAAATT
ATTTAATCAAAATTTCAGAAAAAATGAATGATGCTAAAGTAAGTTTATTATTGAATAATTG
TGATGCTGAATATTCAAAATATTGTGATTGTAAACATACTACTACTCTCGTTAAAAGCGTT
TTAAATGGTAACGACAATACAATTAAGGAAAAGCGTGAACATATTGATTTAGATGATTTT

TCTAAATTTGGATGTGATAAAAATTCCGTTGATACAAACACAAAGGTGTGGGAATGTAAA
AAACCTTATAAATTATCCACTAAAGATGTATGTGTACCTCCGAGGAGGCAAGAATTATGT
CTTGGAAACATTGATAGAATATACGATAAAAACCTATTAATGATAAAAGAGCATATTCTT
GCTATTGCAATATATGAATCAAGAATATTGAAACGAAAATATAAGAATAAAGATGATAA
AGAAGTTTGTAAAATCATAAATAAAACTTTCGCTGATATAAGAGATATTATAGGAGGTAC
TGATTATTGGAATGATTTGAGCAATAGAAAATTAGTAGGAAAAATTAACACAAATTCAAA
TTATGTTCACAGGAATAAACAAAATGATAAGCTTTTTCGTGATGAGTGGTGGAAAGTTAT
TAAAAAAGATGTATGGAATGTGATATCATGGGTATTCAAGGATAAAACTGTTTGTAAAGA
AGATGATATTGAAAATATACCACAATTCTTCAGATGGTTTAGTGAATGGGGTGATGATTA
TTGCCAGGATAAAACAAAAATG
>Thailand 2006 Th58_EBA-175DQ092069.1
TATGTATGTATTCCTGATCGTAGAATCCAATTATGCATTGTTAATCTTAGCATTATTAAAA
CATATACAAAAGAGACCATGAAGGATCATTTCATTGAAGCCTCTAAAAAAGAATCTCAA
CTTTTGCTTAAAAAAAATGATAACAAATATAATTCTAAATTTTGTAATGATTTGAAGAATA
GTTTTTTAGATTATGGACATCTTGCTATGGGAAATGATATGGATTTTGGAGGTTATTCAAC
TAAGGCAGAAAACAAAATTCAAGAAGTTTTTAAAGGGGCTCATGGGAAAATAAGTGAAC
ATGAAATTAAAAATTTTAGAAAAAAATGGTGGAATGAATTTAGAGAGAAACTTTGGGAA
GCTATGCTATCTGAGCATAAAAATAATATAAATAATTGTAAAAATATTCCCCAAGAAGAA
TTACAAATTACTCAATGGATAAAAGAATGGCATGGAGAATTTTTGCTTGAAAGATATAAT
AGATCAAAATTGCCAAAAAGTAAATGTAAAAATAATACATTATATGAAGCATGTGAGAA
GGAATGTATTGATCCATGTATGAAATATAGAGATTGGATTATTAGAAGTAAATTTGAATG
GCATACGTTATCGAAAGAATATGAAACTCAAAATGTTTCAAAGGAAAATGCGGAAAATT
ATTTAATCAAAATTTCAAAAAACAAGAATGATGCTAAAGTAAGTTTATTATTGAATAATTG
TGATGCTGAATATTCAAAATATTGTGATTGTAAACATACTACTACTCTCGTTAAAAGCGTT
TTAAATGGTAACGACAATACAATTAAGGAAAAGCGTGAACATATTGATTTAGATGATTTT
TCTAAATTTGGATGTGATAAAAATTCCGTTGATACAAACACAAAGGTGTGGGAATGTAAA
AAACCTTATATATTATCCACTAAAGATGTATGTGTACCTCCGAGGAGGCAAGAATTATGT
CTTGGAAACATTGATAGAATATACGATAAAAACCTATTAATGATAAAAGAGCATATTCTT
GCTATTGCAATATATGAATCAAGAATATTGAAACGAAAATATAAGAATAAAGATGATAA
AGAAGTTTGTAAAATCATAAATAAAACTTTCGCTGATATAAGAGATATTATAGGAGGTAC
TGATTATTGGAATGATTTGAGCAATAGAAAATTAGTAGGAAAAATTAACACAAATTCAAA
TTATGTTCACAGGAATAAAAAAAAATGATAAGCTTTTTCGTGATGAGTGGTGGAAAGTTAT
TAAAAAAGATGTATGGAATGTGATATCATGGGTATTCAAGGATAAAACTGTTTGTAAAGA
AGATGATATTGAAAATATACCACAATTCTTCAGATGGTTTAGTGAATGGGGTGATGATTA
TTGCCAGGATAAAACAAAAATG
>Thailand 2006 Th56_EBA-175DQ092068.1
TATGTATGTATTCCTGATCGTAGAATCCAATTATGCATTGTTAATCTTAGCATTATTAAAA
CATATACAAAAGAGACCATGAAGGATCATTTCATTGAAGCCTCTAAAAAAGAATCTCAA
CTTTTGCTTAAAAAAAATGATAACAAATATAATTCTAAATTTTGTAATGATTTGAAGAATA
GTTTTTTAGATTATGGACATCTTGCTATGGGAAATGATATGGATTTTGGAGGTTATTCAAC
TAAGGCAGAAAACAAAATTCAAGAAGTTTTTAAAGGGGCTCATGGGGAAATAAGTGAAC
ATAAAATTAAAAATTTTAGAAAAGAATGGTGGAATGAATTTAGAGAGAAACTTTGGGAA
GCTATGTTATCTGAGCATAAAAATAATATAAATAATTGTAAAAATATTCCCCAAGAAGAA
TTACAAATTACTCAATGGATAAAAGAATGGCATGGAGAATTTTTGCTTGAAAGAGATAAT

AGATCAAAATTGCCAAAAAGTAAATGTAAAAATAATACATTATATGAAGCATGTGAGAA
GGAATGTATTGATCCATGTATGAAATATAGAGATTGGATTATTAGAAGTAAATTTGAATG
GCATACGTTATCGAAAGAATATGAAACTCAAAAAGTTCCAAAGGAAAATGCGGAAAATT
ATTTAATCAAAATTTCAGAAAACAAGAATGATGCTAAAGTAAGTTTATTATTGAATAATT
GTGATGCTGAATATTCAAAATATTGTGATTGTAAACATACTACTACTCTCGTTAAAAGCGT
TTTAAATGGTAACGACAATACAATTAAGGAAAAGCGTGAACATATTGATTTAGATGATTT
TTCTAAATTTGGATGTGATAAAAATTCCGTTGATACAAACACAAAGGTGTGGGAATGTAA
AAAACCTTATAAATTATCCACTAAAGATGTATGTGTACCTCCGAGGAGGCAAGAATTATG
TCTTGGAAACATTGATAGAATATACGATAAAAACCTATTAATGATAAAAGAGCATATTCT
TGCTATTGCAATATATGAATCAAGAATATTGAAACGAAAATATAAGAATAAAGATGATAA
AGAAGTTTGTAAAATCATAAATAAAACTTTCGCTGATATAAGAGATATTATAGGAGGTAC
TGATTATTGGAATGATTTGAGCAATAGAAAATTAGTAGGAAAAATTAACACAAATTCAAA
TTATGTTCACAGGAATAAACAAAATGATAAGCTTTTTCGTGATGAGTGGTGGAAAGTTAT
TAAAAAAGATGTATGGAATGTGATATCATGGGTATTCAAGGATAAAACTGTTTGTAAAGA
AGATGATATTGAAAATATACCACAATTCTTCAGATGGTTTAGTGAATGGGGTGATGATTA
TTGCCAGGATAAAACAAAAATG
>Thailand 2006 Th55_EBA-175DQ092067.1
TATGTATGTATTCCTGATCGTAGAATCCAATTATGCATTGTTAATCTTAGCATTATTAAAA
CATATACAAAAGAGACCATGAAGGATCATTTCATTGAAGCCTCTAAAAAAGAATCTCAA
CTTTTGCTTAAAAAAAATGATAACAAATATAATTCTAAATTTTGTAATGATTTGAAGAATA
GTTTTTTAGATTATGGACATCTTGCTATGGGAAATGATATGGATTTTGGAGGTTATTCAAC
TAAGGCAGAAAACAAAATTCAAGAAGTTTTTAAAGGGGCTCATGGGGAAATAAGTGAAC
ATAAAATTAAAAATTTTAGAAAAGAATGGTGGAATGAATTTAGAGAGAAACTTTGGGAA
GCTATGTTATCTGAGCATAAAAATAATATAAATAATTGTAAAAATATTCCCCAAGAAGAA
TTACAAATTACTCAATGGATAAAAGAATGGCATGGAGAATTTTTGCTTGAAAGAGATAAT
AGATCAAAATTGCCAAAAAGTAAATGTAAAAATAATACATTATATGAAGCATGTGAGAA
GGAATGTATTGATCCATGTATGAAATATAGAGATTGGATTATTAGAAGTAAATTTGAATG
GCATACGTTATCGAAAGAATATGAAACTCAAAAAGTTCCAAAGGAAAATGCGGAAAATT
ATTTAATCAAAATTTCAGAAAACAAGAATGATGCTAAAGTAAGTTTATTATTGAATAATT
GTGATGCTGAATATTCAAAATATTGTGATTGTAAACATACTACTACTCTCGTTAAAAGCGT
TTTAAATGGTAACGACAATACAATTAAGGAAAAGCGTGAACATATTGATTTAGATGATTT
TTCTAAATTTGGATGTGATAAAAATTCCGTTGATACAAACACAAAGGTGTGGGAATGTAA
AAAACCTTATAAATTATCCACTAAAGATGTATGTGTACCTCCGAGGAGGCAAGAATTATG
TCTTGGAAACATTGATAGAATATACGATAAAAACCTATTAATGATAAAAGAGCATATTCT
TGCTATTGCAATATATGAATCAAGAATATTGAAACGAAAATATAAGAATAAAGATGATAA
AGAAGTTTGTAAAATCATAAATAAAACTTTCGCTGATATAAGAGATATTATAGGAGGTAC
TGATTATTGGAATGATTTGAGCAATAGAAAATTAGTAGGAAAAATTAACACAAATTCAAA
TTATGTTCACAGGAATAAACAAAATGATAAGCTTTTTCGTGATGAGTGGTGGAAAGTTAT
TAAAAAAGATGTATGGAATGTGATATCATGGGTATTCAAGGATAAAACTGTTTGTAAAGA
AGATGATATTGAAAATATACCACAATTCTTCAGATGGTTTAGTGAATGGGGTGATGATTA
TTGCCAGGATAAAACAAAAATG
>Thailand 2006 Th54_EBA-175DQ092066.1
TATGTATGTATTCCTGATCGTAGAATCCAATTATGCATTGTTAATCTTAGCATTATTAAAA
CATATACAAAAGAGACCATGAAGGATCATTTCATTGAAGCCTCTAAAAAAGAATCTCAA

CTTTTGCTTAAAAAAAAATGATAACAAATATAATTCTAAATTTTGTAATGATTTGAAGAATA
GTTTTTTAGATTATGGACATCTTGCTATGGGAAATGATATGGATTTTGGAGGTTATTCAAC
TAAGGCAGAAAACAAAATTCAAGAAGTTTTTAAAGGGGCTCATGGGAAAATAAGTGAAC
ATGAAATTAAAAATTTTAGAAAAAAAATGGTGGAATGAATTTAGAGAGAAACTTTGGGAA
GCTATGTTATCTGAGCATAAAAATAATATAAATAATTGTAAAAATATTCCCCAAGAAGAA
TTACAAATTACTCAATGGATAAAAGAATGGCATGGAGAATTTTTGCTTGAAAGAGATAAT
AGATCAAAATTGCCAAAAAGTAAATGTAAAAATAATACATTATATGAAGCATGTGAGAA
GGAATGTATTGATCCATGTATGAAATATAGAGATTGGATTATTAGAAGTAAATTTGAATG
GCATACGTTATCGAAAGAATATGAAACTCAAAATGTTTCAAAGGAAAATGCGGAAAATT
ATTTAATCAAAATTTCAGAAAAAATGAATGATGCTAAAGTAAGTTTATTATTGAATAATTG
TGATGCTGAATATTCAAAATATTGTGATTGTAAACATACTACTACTCTCGTTAAAAGCGTT
TTAAATGGTAACGACAATACAATTAAGGAAAAGCGTGAACATATTGATTTAGATGATTTT
TCTAAATTTGGATGTGATAAAAAATTCCGTTGATACAAACACAAAGGTGTGGGAATGTAAA
AAACCTTATAAATTATCCACTAAAGATGTATGTGTACCTCCGAGGAGGCAAGAATTATGT
CTTGGAAACATTGATAGAATATACGATAAAAACCTATTAATGATAAAAGAGCATATTCTT
GCTATTGCAATATATGAATCAAGAATATTGAAACGAAAATATAAGAATAAAGATGATAA
AGAAGTTTGTAAAATCATAAATAAAACTTTCGCTGATATAAGAGATATTATAGGAGGTAC
TGATTATTGGAATGATTTGAGCAATAGAAAATTAGTAGGAAAAATTAACACAAATTCAAA
TTATGTTCACAGGAATAAAGAAAATGATAAGCTTTTTCGTGATGCGTGGTGGAAAGTTAT
TAAAAAAGATGTATGGAATGTGATATCATGGGTATTCAAGGATAAAACTGTTTGTAAAGA
AGATGATATTGAAAATATACCACAATTCTTCAGATGGTTTAGTGAATGGGGTGATGATTA
TTGCCAGGATAAAACAAAAATG
>Thailand 2006 Th50_EBA-175DQ092065.1
TATGTATGTATTCCTGATCGTAGAATCCAATTATGCATTGTTAATCTTAGCATTATTAAAA
CATATACAAAAGAGACCATGAAGGATCATTTCATTGAAGCCTCTAAAAAAGAATCTCAA
CTTTTGCTTAAAAAAAAATGATAACAAATATAATTCTAAATTTTGTAATGATTTGAAGAATA
GTTTTTTAGATTATGGACATCTTGCTATGGGAAATGATATGGATTTTGGAGGTTATTCAAC
TAAGGCAGAAAACAAAATTCAAGAAGTTTTTAAAGGGGCTCATGGGAAAATAAGTGAAC
ATGAAATTAAAAATTTTAGAAAAGAATGGTGGAATGAATTTAGAGAGAAACTTTGGGAA
GCTATGTTATCTGAGCATAAAAATAATATAAATAATTGTAAAAATATTCCCCAAGAAGAA
TTACAAATTACTCAATGGATAAAAGAATGGCATGGAGAATTTTTGCTTGAAAGAGATAAT
AGATCAAAATTGCCAAAAAGTAAATGTAAAAATAATACATTATATGAAGCATGTGAGAA
GGAATGTATTGATCCATGTATGAAATATAGAGATTGGATTATTAGAAGTAAATTTGAATG
GCATACGTTATCGAAAGAATATGAAACTCAAAATGTTTCAAAGGAAAATGCGGAAAATT
ATTTAATCAAAATTTCAGAAAAAATGAATGATGCTAAAGTAAGTTTATTATTGAATAATTG
TGATGCTGAATATTCAAAATATTGTGATTGTAAACATACTACTACTCTCGTTAAAAGCGTT
TTAAATGGTAACGACAATACAATTAAGGAAAAGCGTGAACATATTGATTTAGATGATTTT
TCTAAATTTGGATGTGATAAAAAATTCCGTTGATACAAACACAAAGGTGTGGGAATGTAAA
AAACCTTATAAATTATCCACTAAAGATGTATGTGTACCTCCGAGGAGGCAAGAATTATGT
CTTGGAAACATTGATAGAATATACGATAAAAACCTATTAATGATAAAAGAGCATATTCTT
GCTATTGCAATATATGAATCAAGAATATTGAAACGAAAATATAAGAATAAAGATGATAA
AGAAGTTTGTAAAATCATAAATAAAACTTTCGCTGATATAAGAGATATTATAGGAGGTAC
TGATTATTGGAATGATTTGAGCAATAGAAAATTAGTAGGAAAAATTAACACAAATTCAAA
TTATGTTCACAGGAATAAAGAAAATGATAAGCTTTTTCGTGATGCGTGGTGGAAAGTTAT

TAAAAAAGATGTATGGAATGTGATATCATGGGTATTCAAGGATAAAACTGTTTGTAAAGA
AGATGATATTGAAAATATACCACAATTCTTCAGATGGTTTAGTGAATGGGGTGATGATTA
TTGCCAGGATAAAACAAAAATG
>Thailand 2006 Th48_EBA-175DQ092064.1
TATGTATGTATTCCTGATCGTAGAATCCAATTATGCATTGTTAATCTTAGCATTATTAAAA
CATATACAAAAGAGACCATGAAGGATCATTTCATTGAAGCCTCTAAAAAAGAATCTCAA
CTTTTGCTTAAAAAAAAATGATAACAAATATAATTCTAAATTTTGTAATGATTTGAAGAATA
GTTTTTTAGATTATGGACATCTTGCTATGGGAAATGATATGGATTTTGGAGGTTATTCAAC
TAAGGCAGAAAACAAAATTCAAGAAGTTTTTAAAGGGGCTCATGGGAAAATAAGTGAAC
ATGAAATTAAAAATTTTAGAAAAAAATGGTGGAATGAATTTAGAGAGAAACTTTGGGAA
GCTATGCTATCTGAGCATAAAAATAATATAAATAATTGTAAAAATATTCCCCAAGAAGAA
TTACAAATTACTCAATGGATAAAAGAATGGCATGGAGAATTTTTGCTTGAAAGATATAAT
AGATCAAAATTGCCAAAAAGTAAATGTAAAAATAATACATTATATGAAGCATGTGAGAA
GGAATGTATTGATCCATGTATGAAATATAGAGATTGGATTATTAGAAGTAAATTTGAATG
GCATACGTTATCGAAAGAATATGAAACTCAAAATGTTTCAAAGGAAAATGCGGAAAATT
ATTTAATCAAAATTTCAAAAAACAAGAATGATGCTAAAGTAAGTTTATTATTGAATAATTG
TGATGCTGAATATTCAAAATATTGTGATTGTAAACATACTACTACTCTCGTTAAAAGCGTT
TTAAATGGTAACGACAATACAATTAAGGAAAAGCGTGAACATATTGATTTAGATGATTTT
TCTAAATTTGGATGTGATAAAAATTCCGTTGATACAAACACAAAGGTGTGGGAATGTAAA
AAACCTTATATATTATCCACTAAAGATGTATGTGTACCTCCGAGGAGGCAAGAATTATGT
CTTGGAAACATTGATAGAATATACGATAAAAACCTATTAATGATAAAAGAGCATATTCTT
GCTATTGCAATATATGAATCAAGAATATTGAAACGAAAATATAAGAATAAAGATGATAA
AGAAGTTTGTAAAATCATAAATAAAACTTTCGCTGATATAAGAGATATTATAGGAGGTAC
TGATTATTGGAATGATTTGAGCAATAGAAAATTAGTAGGAAAAATTAACACAAATTCAAA
TTATGTTCACAGGAATAAAAAAAATGATAAGCTTTTTCGTGATGAGTGGTGGAAAGTTAT
TAAAAAAGATGTATGGAATGTGATATCATGGGTATTCAAGGATAAAACTGTTTGTAAAGA
AGATGATATTGAAAATATACCACAATTCTTCAGATGGTTTAGTGAATGGGGTGATGATTA
TTGCCAGGATAAAACAAAAATG
>Thailand 2006 Th43_EBA-175DQ092063.1
TATGTATGTATTCCTGATCGTAGAATCCAATTATGCATTGTTAATCTTAGCATTATTAAAA
CATATACAAAAGAGACCATGAAGGATCATTTCATTGAAGCCTCTAAAAAAGAATCTCAA
CTTTTGCTTAAAAAAAAATGATAACAAATATAATTCTAAATTTTGTAATGATTTGAAGAATA
GTTTTTTAGATTATGGACATCTTGCTATGGGAAATGATATGGATTTTGGAGGTTATTCAAC
TAAGGCAGAAAACAAAATTCAAGAAGTTTTTAAAGGGGCTCATGGGAAAATAAGTGAAC
ATGAAATTAAAAATTTTAGAAAAAAATGGTGGAATGAATTTAGAGAGAAACTTTGGGAA
GCTATGTTATCTGAGCATAAAAATAATATAAATAATTGTAAAAATATTCCCCAAGAAGAA
TTACAAATTACTCAATGGATAAAAGAATGGCATGGAGAATTTTTGCTTGAAAGAGATAAT
AGATCAAAATTGCCAAAAAGTAAATGTAAAAATAATACATTATATGAAGCATGTGAGAA
GGAATGTATTGATCCATGTATGAAATATAGAGATTGGATTATTAGAAGTAAATTTGAATG
GCATACGTTATCGAAAGAATATGAAACTCAAAATGTTTCAAAGGAAAATGCGGAAAATT
ATTTAATCAAAATTTCAGAAAAAATGAATGATGCTAAAGTAAGTTTATTATTGAATAATTG
TGATGCTGAATATTCAAAATATTGTGATTGTAAACATACTACTACTCTCGTTAAAAGCGTT
TTAAATGGTAACGACAATACAATTAAGGAAAAGCGTGAACATATTGATTTAGATGATTTT
TCTAAATTTGGATGTGATAAAAATTCCGTTGATACAAACACAAAGGTGTGGGAATGTAAA

AAACCTTATAAATTATCCACTAAAGATGTATGTGTACCTCCGAGGAGGCAAGAATTATGT
CTTGGAAACATTGATAGAATATACGATAAAAACCTATTAATGATAAAAGAGCATATTCTT
GCTATTGCAATATATGAATCAAGAATATTGAAACGAAAATATAAGAATAAAGATGATAA
AGAAGTTTGTAAAATCATAAATAAAACTTTCGCTGATATAAGAGATATTATAGGAGGTAC
TGATTATTGGAATGATTTGAGCAATAGAAAATTAGTAGGAAAAATTAACACAAATTCAAA
TTATGTTCACAGGAATAAAGAAAATGATAAGCTTTTTCGTGATGCGTGGTGGAAAGTTAT
TAAAAAAGATGTATGGAATGTGATATCATGGGTATTCAAGGATAAAACTGTTTGTAAAGA
AGATGATATTGAAAATATACCACAATTCTTCAGATGGTTTAGTGAATGGGGTGATGATTA
TTGCCAGGATAAAACAAAAATG
>Thailand 2006 Th42_EBA-175DQ092062.1
TATGTATGTATTCCTGATCGTAGAATCCAATTATGCATTGTTAATCTTAGCATTATTAAAA
CATATACAAAAGAGACCATGAAGGATCATTTCATTGAAGCCTCTAAAAAAGAATCTCAA
CTTTTGCTTAAAAAAAAATGATAACAAATATAATTCTAAATTTTGTAATGATTTGAAGAATA
GTTTTTTAGATTATGGACATCTTGCTATGGGAAATGATATGGATTTTGGAGGTTATTCAAC
TAAGGCAGAAAACAAAATTCAAGAAGTTTTTAAAGGGGCTCATGGGGAAATAAGTGAAC
ATAAAATTAAAAATTTTAGAAAAGAATGGTGGAATGAATTTAGAGAGAAACTTTGGGAA
GCTATGTTATCTGAGCATAAAAATAATATAAATAATTGTAAAAATATTCCCCAAGAAGAA
TTACAAATTACTCAATGGATAAAAGAATGGCATGGAGAATTTTTGCTTGAAAGAGATAAT
AGATCAAAATTGCCAAAAAGTAAATGTAAAAATAATACATTATATGAAGCATGTGAGAA
GGAATGTATTGATCCATGTATGAAATATAGAGATTGGATTATTAGAAGTAAATTTGAATG
GCATACGTTATCGAAAGAATATGAAACTCAAAAAGTTCCAAAGGAAAATGCGGAAAATT
ATTTAATCAAAATTTCAGAAAACAAGAATGATGCTAAAGTAAGTTTATTATTGAATAATT
GTGATGCTGAATATTCAAAATATTGTGATTGTAAACATACTACTACTCTCGTTAAAAGCGT
TTTAAATGGTAACGACAATACAATTAAGGAAAAGCGTGAACATATTGATTTAGATGATTT
TTCTAAATTTGGATGTGATAAAAATTCCGTTGATACAAACACAAAGGTGTGGGAATGTAA
AAAACCTTATAAATTATCCACTAAAGATGTATGTGTACCTCCGAGGAGGCAAGAATTATG
TCTTGGAAACATTGATAGAATATACGATAAAAACCTATTAATGATAAAAGAGCATATTCT
TGCTATTGCAATATATGAATCAAGAATATTGAAACGAAAATATAAGAATAAAGATGATAA
AGAAGTTTGTAAAATCATAAATAAAACTTTCGCTGATATAAGAGATATTATAGGAGGTAC
TGATTATTGGAATGATTTGAGCAATAGAAAATTAGTAGGAAAAATTAACACAAATTCAAA
TTATGTTCACAGGAATAAACAAAATGATAAGCTTTTTCGTGATGAGTGGTGGAAAGTTAT
TAAAAAAGATGTATGGAATGTGATATCATGGGTATTCAAGGATAAAACTGTTTGTAAAGA
AGATGATATTGAAAATATACCACAATTCTTCAGATGGTTTAGTGAATGGGGTGATGATTA
TTGCCAGGATAAAACAAAAATG
>Thailand 2006 Th40_EBA-175DQ092061.1
TATGTATGTATTCCTGATCGTAGAATCCAATTATGCATTGTTAATCTTAGCATTATTAAAA
CATATACAAAAGAGACCATGAAGGATCATTTCATTGAAGCCTCTAAAAAAGAATCTCAA
CTTTTGCTTAAAAAAAAATGATAACAAATATAATTCTAAATTTTGTAATGATTTGAAGAATA
GTTTTTTAGATTATGGACATCTTGCTATGGGAAATGATATGGATTTTGGAGGTTATTCAAC
TAAGGCAGAAAACAAAATTCAAGAAGTTTTTAAAGGGGCTCATGGGGAAATAAGTGAAC
ATAAAATTAAAAATTTTAGAAAAGAATGGTGGAATGAATTTAGAGAGAAACTTTGGGAA
GCTATGTTATCTGAGCATAAAAATAATATAAATAATTGTAAAAATATTCCCCAAGAAGAA
TTACAAATTACTCAATGGATAAAAGAATGGCATGGAGAATTTTTGCTTGAAAGAGATAAT
AGATCAAAATTGCCAAAAAGTAAATGTAAAAATAATACATTATATGAAGCATGTGAGAA

GGAATGTATTGATCCATGTATGAAATATAGAGATTGGATTATTAGAAGTAAATTTGAATG
GCATACGTTATCGAAAGAATATGAAACTCAAAAAGTTCCAAAGGAAAATGCGGAAAATT
ATTTAATCAAAATTTCAGAAAACAAGAATGATGCTAAAGTAAGTTTATTATTGAATAATT
GTGATGCTGAATATTCAAAATATTGTGATTGTAAACATACTACTACTCTCGTTAAAAGCGT
TTTAAATGGTAACGACAATACAATTAAGGAAAAGCGTGAACATATTGATTTAGATGATTT
TTCTAAATTTGGATGTGATAAAAATTCCGTTGATACAAACACAAAGGTGTGGGAATGTAA
AAAACCTTATAAATTATCCACTAAAGATGTATGTGTACCTCCGAGGAGGCAAGAATTATG
TCTTGGAAACATTGATAGAATATACGATAAAAACCTATTAATGATAAAAGAGCATATTCT
TGCTATTGCAATATATGAATCAAGAATATTGAAACGAAAATATAAGAATAAAGATGATAA
AGAAGTTTGTAAAATCATAAATAAAACTTTCGCTGATATAAGAGATATTATAGGAGGTAC
TGATTATTGGAATGATTTGAGCAATAGAAAATTAGTAGGAAAAATTAACACAAATTCAAA
TTATGTTCACAGGAATAAACAAAATGATAAGCTTTTTCGTGATGAGTGGTGGAAAGTTAT
TAAAAAAGATGTATGGAATGTGATATCATGGGTATTCAAGGATAAAACTGTTTGTAAAGA
AGATGATATTGAAAATATACCACAATTCTTCAGATGGTTTAGTGAATGGGGTGATGATTA
TTGCCAGGATAAAACAAAAATG
>Thailand 2006 Th36_EBA-175DQ092060.1
TATGTATGTATTCCTGATCGTAGAATCCAATTATGCATTGTTAATCTTAGCATTATTAAAA
CATATACAAAAGAGACCATGAAGGATCATTTCATTGAAGCCTCTAAAAAAGAATCTCAA
CTTTTGCTTAAAAAAAATGATAACAAATATAATTCTAAATTTTGTAATGATTTGAAGAATA
GTTTTTTAGATTATGGACATCTTGCTATGGGAAATGATATGGATTTTGGAGGTTATTCAAC
TAAGGCAGAAAACAAAATTCAAGAAGTTTTTAAAGGGGCTCATGGGAAAATAAGTGAAC
ATGAAATTAAAAATTTTAGAAAAAAATGGTGGAATGAATTTAGAGAGAAACTTTGGGAA
GCTATGCTATCTGAGCATAAAAATAATATAAATAATTGTAAAAATATTCCCCAAGAAGAA
TTACAAATTACTCAATGGATAAAAGAATGGCATGGAGAATTTTTGCTTGAAAGATATAAT
AGATCAAAATTGCCAAAAAGTAAATGTAAAAATAATACATTATATGAAGCATGTGAGAA
GGAATGTATTGATCCATGTATGAAATATAGAGATTGGATTATTAGAAGTAAATTTGAATG
GCATACGTTATCGAAAGAATATGAAACTCAAAATGTTTCAAAGGAAAATGCGGAAAATT
ATTTAATCAAAATTTCAAAAAACAAGAATGATGCTAAAGTAAGTTTATTATTGAATAATTG
TGATGCTGAATATTCAAAATATTGTGATTGTAAACATACTACTACTCTCGTTAAAAGCGTT
TTAAATGGTAACGACAATACAATTAAGGAAAAGCGTGAACATATTGATTTAGATGATTTT
TCTAAATTTGGATGTGATAAAAATTCCGTTGATACAAACACAAAGGTGTGGGAATGTAAA
AAACCTTATATATTATCCACTAAAGATGTATGTGTACCTCCGAGGAGGCAAGAATTATGT
CTTGGAAACATTGATAGAATATACGATAAAAACCTATTAATGATAAAAGAGCATATTCTT
GCTATTGCAATATATGAATCAAGAATATTGAAACGAAAATATAAGAATAAAGATGATAA
AGAAGTTTGTAAAATCATAAATAAAACTTTCGCTGATATAAGAGATATTATAGGAGGTAC
TGATTATTGGAATGATTTGAGCAATAGAAAATTAGTAGGAAAAATTAACACAAATTCAAA
TTATGTTCACAGGAATAAAAAAAAATGATAAGCTTTTTCGTGATGAGTGGTGGAAAGTTAT
TAAAAAAGATGTATGGAATGTGATATCATGGGTATTCAAGGATAAAACTGTTTGTAAAGA
AGATGATATTGAAAATATACCACAATTCTTCAGATGGTTTAGTGAATGGGGTGATGATTA
TTGCCAGGATAAAACAAAAATG
>Thailand 2006 Th34_EBA-175DQ092059.1
TATGTATGTATTCCTGATCGTAGAATCCAATTATGCATTGTTAATCTTAGCATTATTAAAA
CATATACAAAAGAGACCATGAAGGATCATTTCATTGAAGCCTCTAAAAAAGAATCTCAA
CTTTTGCTTAAAAAAAATGATAACAAATATAATTCTAAATTTTGTAATGATTTGAAGAATA

GTTTTTTAGATTATGGACATCTTGCTATGGGAAATGATATGGATTTTGGAGGTTATTCAAC
TAAGGCAGAAAACAAAATTCAAGAAGTTTTTAAAGGGGCTCATGGGAAAATAAGTGAAC
ATGAAATTAAAAATTTTAGAAAAAAAATGGTGGAATGAATTTAGAGAGAAACTTTGGGAA
GCTATGTTATCTGAGCATAAAAATAATATAAATAATTGTAAAAATATTCCCCAAGAAGAA
TTACAAATTACTCAATGGATAAAAGAATGGCATGGAGAATTTTTGCTTGAAAGAGATAAT
AGATCAAAATTGCCAAAAAGTAAATGTAAAAATAATACATTATATGAAGCATGTGAGAA
GGAATGTATTGATCCATGTATGAAATATAGAGATTGGATTATTAGAAGTAAATTTGAATG
GCATACGTTATCGAAAGAATATGAAACTCAAAATGTTTCAAAGGAAAATGCGGAAAATT
ATTTAATCAAAATTTCAAAAAACAAGAATGATGCTAAAGTAAGTTTATTATTGAATAATTG
TGATGCTGAATATTCAAAATATTGTGATTGTAAACATACTACTACTCTCGTTAAAAGCGTT
TTAAATGGTAACGACAATACAATTAAGGAAAAGCGTGAACATATTGATTTAGATGATTTT
TCTAAATTTGGATGTGATAAAAATTCCGTTGATACAAACACAAAGGTGTGGGAATGTAAA
AACCCTTATATATTATCCACTAAAGATGTATGTGTACCTCCGAGGAGGCAAGAATTATGT
CTTGGAAACATTGATAGAATATACGATAAAAACCTATTAATGATAAAAGAGCATATTCTT
GCTATTGCAATATATGAATCAAGAATATTGAAACGAAAATATAAGAATAAAGATGATAA
AGAAGTTTGTAAAATCATAAATAAAACTTTCGCTGATATAAGAGATATTATAGGAGGTAC
TGATTATTGGAATGATTTGAGCAATAGAAAATTAGTAGGAAAAATTAACACAAATTCAAA
TTATGTTCACAGGAATAAAAAAAATGATAAGCTTTTTCGTGATGAGTGGTGGAAAGTTAT
TAAAAAAGATGTATGGAATGTGATATCATGGGTATTCAAGGATAAAACTGTTTGTAAAGA
AGATGATATTGAAAATATACCACAATTCTTCAGATGGTTTAGTGAATGGGGTGATGATTA
TTGCCAGGATAAAACAAAAATG
>Thailand 2006 Th30_EBA-175DQ092058.1
TATGTATGTATTCCTGATCGTAGAATCCAATTATGCATTGTTAATCTTAGCATTATTAAAA
CATATACAAAAGAGACCATGAAGGATCATTTCATTGAAGCCTCTAAAAAAGAATCTCAA
CTTTTGCTTAAAAAAAAATGATAACAAATATAATTCTAAATTTTGTAATGATTTGAAGAATA
GTTTTTTAGATTATGGACATCTTGCTATGGGAAATGATATGGATTTTGGAGGTTATTCAAC
TAAGGCAGAAAACAAAATTCAAGAAGTTTTTAAAGGGGCTCATGGGAAAATAAGTGAAC
ATGAAATTAAAAATTTTAGAAAAGAATGGTGGAATGAATTTAGAGAGAAACTTTGGGAA
GCTATGTTATCTGAGCATAAAAATAATATAAATAATTGTAAAAATATTCCCCAAGAAGAA
TTACAAATTACTCAATGGATAAAAGAATGGCATGGAGAATTTTTGCTTGAAAGAGATAAT
AGATCAAAATTGCCAAAAAGTAAATGTAAAAATAATACATTATATGAAGCATGTGAGAA
GGAATGTATTGATCCATGTATGAAATATAGAGATTGGATTATTAGAAGTAAATTTGAATG
GCATACGTTATCGAAAGAATATGAAACTCAAAATGTTTCAAAGGAAAATGCGGAAAATT
ATTTAATCAAAATTTCAGAAAAAATGAATGATGCTAAAGTAAGTTTATTATTGAATAATTG
TGATGCTGAATATTCAAAATATTGTGATTGTAAACATACTACTACTCTCGTTAAAAGCGTT
TTAAATGGTAACGACAATACAATTAAGGAAAAGCGTGAACATATTGATTTAGATGATTTT
TCTAAATTTGGATGTGATAAAAATTCCGTTGATACAAACACAAAGGTGTGGGAATGTAAA
AAACCTTATAAATTATCCACTAAAGATGTATGTGTACCTCCGAGGAGGCAAGAATTATGT
CTTGGAAACATTGATAGAATATACGATAAAAACCTATTAATGATAAAAGAGCATATTCTT
GCTATTGCAATATATGAATCAAGAATATTGAAACGAAAATATAAGAATAAAGATGATAA
AGAAGTTTGTAAAATCATAAATAAAACTTTCGCTGATATAAGAGATATTATAGGAGGTAC
TGATTATTGGAATGATTTGAGCAATAGAAAATTAGTAGGAAAAATTAACACAAATTCAAA
TTATGTTCACAGGAATAAAGAAAATGATAAGCTTTTTCGTGATGAGTGGTGGAAAGTTAT
TAAAAAAGATGTATGGAATGTGATATCATGGGTATTCAAGGATAAAACTGTTTGTAAAGA

AGATGATATTGAAAATATACCACAATTCTTCAGATGGTTTAGTGAATGGGGTGATGATTA
TTGCCAGGATAAAACAAAAATG
>Thailand 2006 Th27_EBA-175DQ092057.1
TATGTATGTATTCCTGATCGTAGAATCCAATTATGCATTGTTAATCTTAGCATTATTAAAA
CATATACAAAAGAGACCATGAAGGATCATTTCATTGAAGCCTCTAAAAAAGAATCTCAA
CTTTTGCTTAAAAAAAATGATAACAAATATAATTCTAAATTTTGTAATGATTTGAAGAATA
GTTTTTTAGATTATGGACATCTTGCTATGGGAAATGATATGGATTTTGGAGGTTATTCAAC
TAAGGCAGAAAACAAAATTCAAGAAGTTTTTAAAGGGGCTCATGGGAAAATAAGTGAAC
ATGAAATTAAAAATTTTAGAAAAAAATGGTGGAATGAATTTAGAGAGAAACTTTGGGAA
GCTATGCTATCTGAGCATAAAAATAATATAAATAATTGTAAAAATATTCCCCAAGAAGAA
TTACAAATTACTCAATGGATAAAAGAATGGCATGGAGAATTTTTGCTTGAAAGATATAAT
AGATCAAAATTGCCAAAAAGTAAATGTAAAAATAATACATTATATGAAGCATGTGAGAA
GGAATGTATTGATCCATGTATGAAATATAGAGATTGGATTATTAGAAGTAAATTTGAATG
GCATACGTTATCGAAAGAATATGAAACTCAAAATGTTTCAAAGGAAAATGCGGAAAATT
ATTTAATCAAAATTTCAAAAAACAAGAATGATGCTAAAGTAAGTTTATTATTGAATAATTG
TGATGCTGAATATTCAAAATATTGTGATTGTAAACATACTACTACTCTCGTTAAAAGCGTT
TTAAATGGTAACGACAATACAATTAAGGAAAAGCGTGAACATATTGATTTAGATGATTTT
TCTAAATTTGGATGTGATAAAAATTCCGTTGATACAAACACAAAGGTGTGGGAATGTAAA
AAACCTTATATATTATCCACTAAAGATGTATGTGTACCTCCGAGGAGGCAAGAATTATGT
CTTGGAAACATTGATAGAATATACGATAAAAACCTATTAATGATAAAAGAGCATATTCTT
GCTATTGCAATATATGAATCAAGAATATTGAAACGAAAATATAAGAATAAAGATGATAA
AGAAGTTTGTAAAATCATAAATAAAACTTTCGCTGATATAAGAGATATTATAGGAGGTAC
TGATTATTGGAATGATTTGAGCAATAGAAAATTAGTAGGAAAAATTAACACAAATTCAAA
TTATGTTCACAGGAATAAAAAAAATGATAAGCTTTTTCGTGATGAGTGGTGGAAAGTTAT
TAAAAAAGATGTATGGAATGTGATATCATGGGTATTCAAGGATAAAACTGTTTGTAAAGA
AGATGATATTGAAAATATACCACAATTCTTCAGATGGTTTAGTGAATGGGGTGATGATTA
TTGCCAGGATAAAACAAAAATG
>Thailand 2006 Th26_EBA-175DQ092056.1
TATGTATGTATTCCTGATCGTAGAATCCAATTATGCATTGTTAATCTTAGCATTATTAAAA
CATATACAAAAGAGACCATGAAGGATCATTTCATTGAAGCCTCTAAAAAAGAATCTCAA
CTTTTGCTTAAAAAAAATGATAACAAATATAATTCTAAATTTTGTAATGATTTGAAGAATA
GTTTTTTAGATTATGGACATCTTGCTATGGGAAATGATATGGATTTTGGAGGTTATTCAAC
TAAGGCAGAAAACAAAATTCAAGAAGTTTTTAAAGGGGCTCATGGGAAAATAAGTGAAC
ATGAAATTAAAAATTTTAGAAAAAAATGGTGGAATGAATTTAGAGAGAAACTTTGGGAA
GCTATGCTATCTGAGCATAAAAATAATATAAATAATTGTAAAAATATTCCCCAAGAAGAA
TTACAAATTACTCAATGGATAAAAGAATGGCATGGAGAATTTTTGCTTGAAAGATATAAT
AGATCAAAATTGCCAAAAAGTAAATGTAAAAATAATACATTATATGAAGCATGTGAGAA
GGAATGTATTGATCCATGTATGAAATATAGAGATTGGATTATTAGAAGTAAATTTGAATG
GCATACGTTATCGAAAGAATATGAAACTCAAAATGTTTCAAAGGAAAATGCGGAAAATT
ATTTAATCAAAATTTCAAAAAACAAGAATGATGCTAAAGTAAGTTTATTATTGAATAATTG
TGATGCTGAATATTCAAAATATTGTGATTGTAAACATACTACTACTCTCGTTAAAAGCGTT
TTAAATGGTAACGACAATACAATTAAGGAAAAGCGTGAACATATTGATTTAGATGATTTT
TCTAAATTTGGATGTGATAAAAATTCCGTTGATACAAACACAAAGGTGTGGGAATGTAAA
AAACCTTATATATTATCCACTAAAGATGTATGTGTACCTCCGAGGAGGCAAGAATTATGT

CTTGGAAACATTGATAGAATATACGATAAAAACCTATTAATGATAAAAGAGCATATTCTT
GCTATTGCAATATATGAATCAAGAATATTGAAACGAAAATATAAGAATAAAGATGATAA
AGAAGTTTGTAAAATCATAAATAAAACTTTCGCTGATATAAGAGATATTATAGGAGGTAC
TGATTATTGGAATGATTTGAGCAATAGAAAATTAGTAGGAAAAATTAACACAAATTCAAA
TTATGTTCACAGGAATAAAAAAAATGATAAGCTTTTTCGTGATGAGTGGTGGAAAGTTAT
TAAAAAAGATGTATGGAATGTGATATCATGGGTATTCAAGGATAAAACTGTTTGTAAAGA
AGATGATATTGAAAATATACCACAATTCTTCAGATGGTTTAGTGAATGGGGTGATGATTA
TTGCCAGGATAAAACAAAAATG
>Thailand 2006 Th25_EBA-175DQ092055.1
TATGTATGTATTCCTGATCGTAGAATCCAATTATGCATTGTTAATCTTAGCATTATTAAAA
CATATACAAAAGAGACCATGAAGGATCATTTCATTGAAGCCTCTAAAAAAGAATCTCAA
CTTTTGCTTAAAAAAAATGATAACAAATATAATTCTAAATTTTGTAATGATTTGAAGAATA
GTTTTTTAGATTATGGACATCTTGCTATGGGAAATGATATGGATTTTGGAGGTTATTCAAC
TAAGGCAGAAAACAAAATTCAAGAAGTTTTTAAAGGGGCTCATGGGGAAATAAGTGAAC
ATAAAATTAAAAATTTTAGAAAAGAATGGTGGAATGAATTTAGAGAGAAACTTTGGGAA
GCTATGTTATCTGAGCATAAAAATAATATAAATAATTGTAAAAATATTCCCCAAGAAGAA
TTACAAATTACTCAATGGATAAAAGAATGGCATGGAGAATTTTTGCTTGAAAGAGATAAT
AGATCAAAATTGCCAAAAAGTAAATGTAAAAATAATACATTATATGAAGCATGTGAGAA
GGAATGTATTGATCCATGTATGAAATATAGAGATTGGATTATTAGAAGTAAATTTGAATG
GCATACGTTATCGAAAGAATATGAAACTCAAAAAGTTCCAAAGGAAAATGCGGAAAATT
ATTTAATCAAAATTTCAGAAAACAAGAATGATGCTAAAGTAAGTTTATTATTGAATAATT
GTGATGCTGAATATTCAAAATATTGTGATTGTAAACATACTACTACTCTCGTTAAAAGCGT
TTTAAATGGTAACGACAATACAATTAAGGAAAAGCGTGAACATATTGATTTAGATGATTT
TTCTAAATTTGGATGTGATAAAAATTCCGTTGATACAAACACAAAGGTGTGGGAATGTAA
AAAACCTTATAAATTATCCACTAAAGATGTATGTGTACCTCCGAGGAGGCAAGAATTATG
TCTTGGAAACATTGATAGAATATACGATAAAAACCTATTAATGATAAAAGAGCATATTCT
TGCTATTGCAATATATGAATCAAGAATATTGAAACGAAAATATAAGAATAAAGATGATAA
AGAAGTTTGTAAAATCATAAATAAAACTTTCGCTGATATAAGAGATATTATAGGAGGTAC
TGATTATTGGAATGATTTGAGCAATAGAAAATTAGTAGGAAAAATTAACACAAATTCAAA
TTATGTTCACAGGAATAAAAAAAATGATAAGCTTTTTCGTGATGAGTGGTGGAAAGTTAT
TAAAAAAGATGTATGGAATGTGATATCATGGGTATTCAAGGATAAAACTGTTTGTAAAGA
AGATGATATTGAAAATATACCACAATTCTTCAGATGGTTTAGTGAATGGGGTGATGATTA
TTGCCAGGATAAAACAAAAATG
>Thailand 2006 Th24_EBA-175DQ092054.1
TATGTATGTATTCCTGATCGTAGAATCCAATTATGCATTGTTAATCTTAGCATTATTAAAA
CATATACAAAAGAGACCATGAAGGATCATTTCATTGAAGCCTCTAAAAAAGAATCTCAA
CTTTTGCTTAAAAAAAATGATAACAAATATAATTCTAAATTTTGTAATGATTTGAAGAATA
GTTTTTTAGATTATGGACATCTTGCTATGGGAAATGATATGGATTTTGGAGGTTATTCAAC
TAAGGCAGAAAACAAAATTCAAGAAGTTTTTAAAGGGGCTCATGGGAAAATAAGTGAAC
ATGAAATTAAAAATTTTAGAAAAGAATGGTGGAATGAATTTAGAGAGAAACTTTGGGAA
GCTATGTTATCTGAGCATAAAAATAATATAAATAATTGTAAAAATATTCCCCAAGAAGAA
TTACAAATTACTCAATGGATAAAAGAATGGCATGGAGAATTTTTGCTTGAAAGAGATAAT
AGATCAAAATTGCCAAAAAGTAAATGTAAAAATAATACATTATATGAAGCATGTGAGAA
GGAATGTATTGATCCATGTATGAAATATAGAGATTGGATTATTAGAAGTAAATTTGAATG

GCATACGTTATCGAAAGAATATGAAACTCAAAATGTTTCAAAGGAAAATGCGGAAAATT
ATTTAATCAAAATTTCAGAAAAAATGAATGATGCTAAAGTAAGTTTATTATTGAATAATTG
TGATGCTGAATATTCAAAATATTGTGATTGTAAACATACTACTACTCTCGTTAAAAGCGTT
TTAAATGGTAACGACAATACAATTAAGGAAAAGCGTGAACATATTGATTTAGATGATTTT
TCTAAATTTGGATGTGATAAAAATTCCGTTGATACAAACACAAAGGTGTGGGAATGTAAA
AAACCTTATAAATTATCCACTAAAGATGTATGTGTACCTCCGAGGAGGCAAGAATTATGT
CTTGGAAACATTGATAGAATATACGATAAAAACCTATTAATGATAAAAGAGCATATTCTT
GCTATTGCAATATATGAATCAAGAATATTGAAACGAAAATATAAGAATAAAGATGATAA
AGAAGTTTGTAAAATCATAAATAAAACTTTCGCTGATATAAGAGATATTATAGGAGGTAC
TGATTATTGGAATGATTTGAGCAATAGAAAATTAGTAGGAAAAATTAACACAAATTCAAA
TTATGTTCACAGGAATAAAGAAAATGATAAGCTTTTTCGTGATGCGTGGTGGAAAGTTAT
TAAAAAAGATGTATGGAATGTGATATCATGGGTATTCAAGGATAAAACTGTTTGTAAAGA
AGATGATATTGAAAATATACCACAATTCTTCAGATGGTTTAGTGAATGGGGTGATGATTA
TTGCCAGGATAAAACAAAAATG
>Thailand 2006 Th23_EBA-175DQ092053.1
TATGTATGTATTCCTGATCGTAGAATCCAATTATGCATTGTTAATCTTAGCATTATTAAAA
CATATACAAAAGAGACCATGAAGGATCATTTCATTGAAGCCTCTAAAAAAGAATCTCAA
CTTTTGCTTAAAAAAAATGATAACAAATATAATTCTAAATTTTGTAATGATTTGAAGAATA
GTTTTTTAGATTATGGACATCTTGCTATGGGAAATGATATGGATTTTGGAGGTTATTCAAC
TAAGGCAGAAAACAAAATTCAAGAAGTTTTTAAAGGGGCTCATGGGGAAATAAGTGAAC
ATAAAATTAAAAATTTTAGAAAAGAATGGTGGAATGAATTTAGAGAGAAACTTTGGGAA
GCTATGTTATCTGAGCATAAAAATAATATAAATAATTGTAAAAATATTCCCCAAGAAGAA
TTACAAATTACTCAATGGATAAAAGAATGGCATGGAGAATTTTTGCTTGAAAGAGATAAT
AGATCAAAATTGCCAAAAAGTAAATGTAAAAATAATACATTATATGAAGCATGTGAGAA
GGAATGTATTGATCCATGTATGAAATATAGAGATTGGATTATTAGAAGTAAATTTGAATG
GCATACGTTATCGAAAGAATATGAAACTCAAAAAGTTCCAAAGGAAAATGCGGAAAATT
ATTTAATCAAAATTTCAGAAAACAAGAATGATGCTAAAGTAAGTTTATTATTGAATAATT
GTGATGCTGAATATTCAAAATATTGTGATTGTAAACATACTACTACTCTCGTTAAAAGCGT
TTTAAATGGTAACGACAATACAATTAAGGAAAAGCGTGAACATATTGATTTAGATGATTT
TTCTAAATTTGGATGTGATAAAAATTCCGTTGATACAAACACAAAGGTGTGGGAATGTAA
AAAACCTTATAAATTATCCACTAAAGATGTATGTGTACCTCCGAGGAGGCAAGAATTATG
TCTTGGAAACATTGATAGAATATACGATAAAAACCTATTAATGATAAAAGAGCATATTCT
TGCTATTGCAATATATGAATCAAGAATATTGAAACGAAAATATAAGAATAAAGATGATAA
AGAAGTTTGTAAAATCATAAATAAAACTTTCGCTGATATAAGAGATATTATAGGAGGTAC
TGATTATTGGAATGATTTGAGCAATAGAAAATTAGTAGGAAAAATTAACACAAATTCAAA
TTATGTTCACAGGAATAAACAAAATGATAAGCTTTTTCGTGATGAGTGGTGGAAAGTTAT
TAAAAAAGATGTATGGAATGTGATATCATGGGTATTCAAGGATAAAACTGTTTGTAAAGA
AGATGATATTGAAAATATACCACAATTCTTCAGATGGTTTAGTGAATGGGGTGATGATTA
TTGCCAGGATAAAACAAAAATG
>Thailand 2006 Th22_EBA-175DQ092052.1
TATGTATGTATTCCTGATCGTAGAATCCAATTATGCATTGTTAATCTTAGCATTATTAAAA
CATATACAAAAGAGACCATGAAGGATCATTTCATTGAAGCCTCTAAAAAAGAATCTCAA
CTTTTGCTTAAAAAAAATGATAACAAATATAATTCTAAATTTTGTAATGATTTGAAGAATA
GTTTTTTAGATTATGGACATCTTGCTATGGGAAATGATATGGATTTTGGAGGTTATTCAAC

TAAGGCAGAAAACAAAATTCAAGAAGTTTTTAAAGGGGCTCATGGGAAAATAAGTGAAC
ATGAAATTAAAAATTTTAGAAAAGAATGGTGGAATGAATTTAGAGAGAAACTTTGGGAA
GCTATGTTATCTGAGCATAAAAATAATATAAATAATTGTAAAAATATTCCCCAAGAAGAA
TTACAAATTACTCAATGGATAAAAGAATGGCATGGAGAATTTTTGCTTGAAAGAGATAAT
AGATCAAAATTGCCAAAAAGTAAATGTAAAAATAATACATTATATGAAGCATGTGAGAA
GGAATGTATTGATCCATGTATGAAATATAGAGATTGGATTATTAGAAGTAAATTTGAATG
GCATACGTTATCGAAAGAATATGAAACTCAAAATGTTTCAAAGGAAAATGCGGAAAATT
ATTTAATCAAAATTTCAGAAAAAATGAATGATGCTAAAGTAAGTTTATTATTGAATAATTG
TGATGCTGAATATTCAAAATATTGTGATTGTAAACATACTACTACTCTCGTTAAAAGCGTT
TTAAATGGTAACGACAATACAATTAAGGAAAAGCGTGAACATATTGATTTAGATGATTTT
TCTAAATTTGGATGTGATAAAAATTCCGTTGATACAAACACAAAGGTGTGGGAATGTAAA
AAACCTTATAAATTATCCACTAAAGATGTATGTGTACCTCCGAGGAGGCAAGAATTATGT
CTTGGAAACATTGATAGAATATACGATAAAAACCTATTAATGATAAAAGAGCATATTCTT
GCTATTGCAATATATGAATCAAGAATATTGAAACGAAAATATAAGAATAAAGATGATAA
AGAAGTTTGTAAAATCATAAATAAAACTTTCGCTGATATAAGAGATATTATAGGAGGTAC
TGATTATTGGAATGATTTGAGCAATAGAAAATTAGTAGGAAAAATTAACACAAATTCAAA
TTATGTTCACAGGAATAAACAAAATGATAAGCTTTTTCGTGATGAGTGGTGGAAAGTTAT
TAAAAAAGATGTATGGAATGTGATATCATGGGTATTCAAGGATAAAACTGTTTGTAAAGA
AGATGATATTGAAAATATACCACAATTCTTCAGATGGTTTAGTGAATGGGGTGATGATTA
TTGCCAGGATAAAACAAAAATG
>Thailand 2006 Th21_EBA-175DQ092051.1
TATGTATGTATTCCTGATCGTAGAATCCAATTATGCATTGTTAATCTTAGCATTATTAAAA
CATATACAAAAGAGACCATGAAGGATCATTTCATTGAAGCCTCTAAAAAAGAATCTCAA
CTTTTGCTTAAAAAAAAATGATAACAAATATAATTCTAAATTTTGTAATGATTTGAAGAATA
GTTTTTTAGATTATGGACATCTTGCTATGGGAAATGATATGGATTTTGGAGGTTATTCAAC
TAAGGCAGAAAACAAAATTCAAGAAGTTTTTAAAGGGGCTCATGGGAAAATAAGTGAAC
ATGAAATTAAAAATTTTAGAAAAGAATGGTGGAATGAATTTAGAGAGAAACTTTGGGAA
GCTATGTTATCTGAGCATAAAAATAATATAAATAATTGTAAAAATATTCCCCAAGAAGAA
TTACAAATTACTCAATGGATAAAAGAATGGCATGGAGAATTTTTGCTTGAAAGAGATAAT
AGATCAAAATTGCCAAAAAGTAAATGTAAAAATAATACATTATATGAAGCATGTGAGAA
GGAATGTATTGATCCATGTATGAAATATAGAGATTGGATTATTAGAAGTAAATTTGAATG
GCATACGTTATCGAAAGAATATGAAACTCAAAATGTTTCAAAGGAAAATGCGGAAAATT
ATTTAATCAAAATTTCAGAAAAAATGAATGATGCTAAAGTAAGTTTATTATTGAATAATTG
TGATGCTGAATATTCAAAATATTGTGATTGTAAACATACTACTACTCTCGTTAAAAGCGTT
TTAAATGGTAACGACAATACAATTAAGGAAAAGCGTGAACATATTGATTTAGATGATTTT
TCTAAATTTGGATGTGATAAAAATTCCGTTGATACAAACACAAAGGTGTGGGAATGTAAA
AAACCTTATAAATTATCCACTAAAGATGTATGTGTACCTCCGAGGAGGCAAGAATTATGT
CTTGGAAACATTGATAGAATATACGATAAAAACCTATTAATGATAAAAGAGCATATTCTT
GCTATTGCAATATATGAATCAAGAATATTGAAACGAAAATATAAGAATAAAGATGATAA
AGAAGTTTGTAAAATCATAAATAAAACTTTCGCTGATATAAGAGATATTATAGGAGGTAC
TGATTATTGGAATGATTTGAGCAATAGAAAATTAGTAGGAAAAATTAACACAAATTCAAA
TTATGTTCACAGGAATAAACAAAATGATAAGCTTTTTCGTGATGAGTGGTGGAAAGTTAT
TAAAAAAGATGTATGGAATGTGATATCATGGGTATTCAAGGATAAAACTGTTTGTAAAGA
AGATGATATTGAAAATATACCACAATTCTTCAGATGGTTTAGTGAATGGGGTGATGATTA

TTGCCAGGATAAAACAAAAATG
>Thailand 2006 Th19_EBA-175DQ092050.1
TATGTATGTATTCCTGATCGTAGAATCCAATTATGCATTGTTAATCTTAGCATTATTAAAA
CATATACAAAAGAGACCATGAAGGATCATTTCATTGAAGCCTCTAAAAAAGAATCTCAA
CTTTTGCTTAAAAAAAAATGATAACAAATATAATTCTAAATTTTGTAATGATTTGAAGAATA
GTTTTTTAGATTATGGACATCTTGCTATGGGAAATGATATGGATTTTGGAGGTTATTCAAC
TAAGGCAGAAAACAAAATTCAAGAAGTTTTTAAAGGGGCTCATGGGAAAATAAGTGAAC
ATGAAATTAAAAATTTTAGAAAAGAATGGTGGAATGAATTTAGAGAGAAACTTTGGGAA
GCTATGTTATCTGAGCATAAAAATAATATAAATAATTGTAAAAATATTCCCCAAGAAGAA
TTACAAATTACTCAATGGATAAAAGAATGGCATGGAGAATTTTTGCTTGAAAGAGATAAT
AGATCAAAATTGCCAAAAAGTAAATGTAAAAATAATACATTATATGAAGCATGTGAGAA
GGAATGTATTGATCCATGTATGAAATATAGAGATTGGATTATTAGAAGTAAATTTGAATG
GCATACGTTATCGAAAGAATATGAAACTCAAAATGTTTCAAAGGAAAATGCGGAAAATT
ATTTAATCAAAATTTCAGAAAAAATGAATGATGCTAAAGTAAGTTTATTATTGAATAATTG
TGATGCTGAATATTCAAAATATTGTGATTGTAAACATACTACTACTCTCGTTAAAAGCGTT
TTAAATGGTAACGACAATACAATTAAGGAAAAGCGTGAACATATTGATTTAGATGATTTT
TCTAAATTTGGATGTGATAAAAATTCCGTTGATACAAACACAAAGGTGTGGGAATGTAAA
AAACCTTATAAATTATCCACTAAAGATGTATGTGTACCTCCGAGGAGGCAAGAATTATGT
CTTGGAAACATTGATAGAATATACGATAAAAACCTATTAATGATAAAAGAGCATATTCTT
GCTATTGCAATATATGAATCAAGAATATTGAAACGAAAATATAAGAATAAAGATGATAA
AGAAGTTTGTAAAATCATAAATAAAACTTTCGCTGATATAAGAGATATTATAGGAGGTAC
TGATTATTGGAATGATTTGAGCAATAGAAAATTAGTAGGAAAAATTAACACAAATTCAAA
TTATGTTCACAGGAATAAAGAAAATGATAAGCTTTTTCGTGATGCGTGGTGGAAAGTTAT
TAAAAAAGATGTATGGAATGTGATATCATGGGTATTCAAGGATAAAACTGTTTGTAAAGA
AGATGATATTGAAAATATACCACAATTCTTCAGATGGTTTAGTGAATGGGGTGATGATTA
TTGCCAGGATAAAACAAAAATG
>Thailand 2006 Th18_EBA-175DQ092049.1
TATGTATGTATTCCTGATCGTAGAATCCAATTATGCATTGTTAATCTTAGCATTATTAAAA
CATATACAAAAGAGACCATGAAGGATCATTTCATTGAAGCCTCTAAAAAAGAATCTCAA
CTTTTGCTTAAAAAAAAATGATAACAAATATAATTCTAAATTTTGTAATGATTTGAAGAATA
GTTTTTTAGATTATGGACATCTTGCTATGGGAAATGATATGGATTTTGGAGGTTATTCAAC
TAAGGCAGAAAACAAAATTCAAGAAGTTTTTAAAGGGGCTCATGGGAAAATAAGTGAAC
ATGAAATTAAAAATTTTAGAAAAAAAATGGTGGAATGAATTTAGAGAGAAACTTTGGGAA
GCTATGCTATCTGAGCATAAAAATAATATAAATAATTGTAAAAATATTCCCCAAGAAGAA
TTACAAATTACTCAATGGATAAAAGAATGGCATGGAGAATTTTTGCTTGAAAGATATAAT
AGATCAAAATTGCCAAAAAGTAAATGTAAAAATAATACATTATATGAAGCATGTGAGAA
GGAATGTATTGATCCATGTATGAAATATAGAGATTGGATTATTAGAAGTAAATTTGAATG
GCATACGTTATCGAAAGAATATGAAACTCAAAATGTTTCAAAGGAAAATGCGGAAAATT
ATTTAATCAAAATTTCAAAAAACAAGAATGATGCTAAAGTAAGTTTATTATTGAATAATTG
TGATGCTGAATATTCAAAATATTGTGATTGTAAACATACTACTACTCTCGTTAAAAGCGTT
TTAAATGGTAACGACAATACAATTAAGGAAAAGCGTGAACATATTGATTTAGATGATTTT
TCTAAATTTGGATGTGATAAAAATTCCGTTGATACAAACACAAAGGTGTGGGAATGTAAA
AAACCTTATATATTATCCACTAAAGATGTATGTGTACCTCCGAGGAGGCAAGAATTATGT
CTTGGAAACATTGATAGAATATACGATAAAAACCTATTAATGATAAAAGAGCATATTCTT

GCTATTGCAATATATGAATCAAGAATATTGAAACGAAAATATAAGAATAAAGATGATAA
AGAAGTTTGTAAAATCATAAATAAAACTTTCGCTGATATAAGAGATATTATAGGAGGTAC
TGATTATTGGAATGATTTGAGCAATAGAAAATTAGTAGGAAAAATTAACACAAATTCAAA
TTATGTTCACAGGAATAAAAAAAATGATAAGCTTTTTCGTGATGAGTGGTGGAAAGTTAT
TAAAAAAGATGTATGGAATGTGATATCATGGGTATTCAAGGATAAAACTGTTTGTAAAGA
AGATGATATTGAAAATATACCACAATTCTTCAGATGGTTTAGTGAATGGGGTGATGATTA
TTGCCAGGATAAAACAAAAATG
>Thailand 2006 Th17_EBA-175DQ092048.1
TATGTATGTATTCCTGATCGTAGAATCCAATTATGCATTGTTAATCTTAGCATTATTAAAA
CATATACAAAAGAGACCATGAAGGATCATTTCATTGAAGCCTCTAAAAAAGAATCTCAA
CTTTTGCTTAAAAAAAAATGATAACAAATATAATTCTAAATTTTGTAATGATTTGAAGAATA
GTTTTTTAGATTATGGACATCTTGCTATGGGAAATGATATGGATTTTGGAGGTTATTCAAC
TAAGGCAGAAAACAAAATTCAAGAAGTTTTTAAAGGGGCTCATGGGGAAATAAGTGAAC
ATAAAATTAAAAATTTTAGAAAAGAATGGTGGAATGAATTTAGAGAGAAACTTTGGGAA
GCTATGTTATCTGAGCATAAAAATAATATAAATAATTGTAAAAATATTCCCCAAGAAGAA
TTACAAATTACTCAATGGATAAAAGAATGGCATGGAGAATTTTTGCTTGAAAGAGATAAT
AGATCAAAATTGCCAAAAAGTAAATGTAAAAATAATACATTATATGAAGCATGTGAGAA
GGAATGTATTGATCCATGTATGAAATATAGAGATTGGATTATTAGAAGTAAATTTGAATG
GCATACGTTATCGAAAGAATATGAAACTCAAAAAGTTCCAAAGGAAAATGCGGAAAATT
ATTTAATCAAAATTTCAGAAAACAAGAATGATGCTAAAGTAAGTTTATTATTGAATAATT
GTGATGCTGAATATTCAAAATATTGTGATTGTAAACATACTACTACTCTCGTTAAAAGCGT
TTTAAATGGTAACGACAATACAATTAAGGAAAAGCGTGAACATATTGATTTAGATGATTT
TTCTAAATTTGGATGTGATAAAAATTCCGTTGATACAAACACAAAGGTGTGGGAATGTAA
AAAACCTTATAAATTATCCACTAAAGATGTATGTGTACCTCCGAGGAGGCAAGAATTATG
TCTTGGAAACATTGATAGAATATACGATAAAAACCTATTAATGATAAAAGAGCATATTCT
TGCTATTGCAATATATGAATCAAGAATATTGAAACGAAAATATAAGAATAAAGATGATAA
AGAAGTTTGTAAAATCATAAATAAAACTTTCGCTGATATAAGAGATATTATAGGAGGTAC
TGATTATTGGAATGATTTGAGCAATAGAAAATTAGTAGGAAAAATTAACACAAATTCAAA
TTATGTTCACAGGAATAAACAAAATGATAAGCTTTTTCGTGATGAGTGGTGGAAAGTTAT
TAAAAAAGATGTATGGAATGTGATATCATGGGTATTCAAGGATAAAACTGTTTGTAAAGA
AGATGATATTGAAAATATACCACAATTCTTCAGATGGTTTAGTGAATGGGGTGATGATTA
TTGCCAGGATAAAACAAAAATG
>Thailand 2006 Th16_EBA-175DQ092047.1
TATGTATGTATTCCTGATCGTAGAATCCAATTATGCATTGTTAATCTTAGCATTATTAAAA
CATATACAAAAGAGACCATGAAGGATCATTTCATTGAAGCCTCTAAAAAAGAATCTCAA
CTTTTGCTTAAAAAAAAATGATAACAAATATAATTCTAAATTTTGTAATGATTTGAAGAATA
GTTTTTTAGATTATGGACATCTTGCTATGGGAAATGATATGGATTTTGGAGGTTATTCAAC
TAAGGCAGAAAACAAAATTCAAGAAGTTTTTAAAGGGGCTCATGGGAAAATAAGTGAAC
ATGAAATTAAAAATTTTAGAAAAAAAATGGTGGAATGAATTTAGAGAGAAACTTTGGGAA
GCTATGTTATCTGAGCATAAAAATAATATAAATAATTGTAAAAATATTCCCCAAGAAGAA
TTACAAATTACTCAATGGATAAAAGAATGGCATGGAGAATTTTTGCTTGAAAGATATAAT
AGATCAAAATTGCCAAAAAGTAAATGTAAAAATAATACATTATATGAAGCATGTGAGAA
GGAATGTATTGATCCATGTATGAAATATAGAGATTGGATTATTAGAAGTAAATTTGAATG
GCATACGTTATCGAAAGAATATGAAACTCAAAATGTTTCAAAGGAAAATGCGGAAAATT

ATTTAATCAAAATTTCAGAAAAAATGAATGATGCTAAAGTAAGTTTATTATTGAATAATTG
TGATGCTGAATATTCAAAATATTGTGATTGTAAACATACTACTACTCTCGTTAAAAGCGTT
TTAAATGGTAACGACAATACAATTAAGGAAAAGCGTGAACATATTGATTTAGATGATTTT
TCTAAATTTGGATGTGATAAAAATTCCGTTGATACAAACACAAAGGTGTGGGAATGTAAA
AAACCTTATAAATTATCCACTAAAGATGTATGTGTACCTCCGAGGAGGCAAGAATTATGT
CTTGGAAACATTGATAGAATATACGATAAAAACCTATTAATGATAAAAGAGCATATTCTT
GCTATTGCAATATATGAATCAAGAATATTGAAACGAAAATATAAGAATAAAGATGATAA
AGAAGTTTGTAAAATCATAAATAAAACTTTCGCTGATATAAGAGATATTATAGGAGGTAC
TGATTATTGGAATGATTTGAGCAATAGAAAATTAGTAGGAAAAATTAACACAAATTCAAA
TTATGTTCACAGGAATAAAGAAAATGATAAGCTTTTTCGTGATGAGTGGTGGAAAGTTAT
TAAAAAAGATGTATGGAATGTGATATCATGGGTATTCAAGGATAAAACTGTTTGTAAAGA
AGATGATATTGAAAATATACCACAATTCTTCAGATGGTTTAGTGAATGGGGTGATGATTA
TTGCCAGGATAAAACAAAAATG
>Thailand 2006 Th13_EBA-175DQ092046.1
TATGTATGTATTCCTGATCGTAGAATCCAATTATGCATTGTTAATCTTAGCATTATTAAAA
CATATACAAAAGAGACCATGAAGGATCATTTCATTGAAGCCTCTAAAAAAGAATCTCAA
CTTTTGCTTAAAAAAAAATGATAACAAATATAATTCTAAATTTTGTAATGATTTGAAGAATA
GTTTTTTAGATTATGGACATCTTGCTATGGGAAATGATATGGATTTTGGAGGTTATTCAAC
TAAGGCAGAAAACAAAATTCAAGAAGTTTTTAAAGGGGCTCATGGGAAAATAAGTGAAC
ATGAAATTAAAAATTTTAGAAAAAAATGGTGGAATGAATTTAGAGAGAAACTTTGGGAA
GCTATGTTATCTGAGCATAAAAATAATATAAATAATTGTAAAAATATTCCCCAAGAAGAA
TTACAAATTACTCAATGGATAAAAGAATGGCATGGAGAATTTTTGCTTGAAAGAGATAAT
AGATCAAAATTGCCAAAAAGTAAATGTAAAAATAATACATTATATGAAGCATGTGAGAA
GGAATGTATTGATCCATGTATGAAATATAGAGATTGGATTATTAGAAGTAAATTTGAATG
GCATACGTTATCGAAAGAATATGAAACTCAAAATGTTTCAAAGGAAAATGCGGAAAATT
ATTTAATCAAAATTTCAGAAAAAATGAATGATGCTAAAGTAAGTTTATTATTGAATAATTG
TGATGCTGAATATTCAAAATATTGTGATTGTAAACATACTACTACTCTCGTTAAAAGCGTT
TTAAATGGTAACGACAATACAATTAAGGAAAAGCGTGAACATATTGATTTAGATGATTTT
TCTAAATTTGGATGTGATAAAAATTCCGTTGATACAAACACAAAGGTGTGGGAATGTAAA
AAACCTTATAAATTATCCACTAAAGATGTATGTGTACCTCCGAGGAGGCAAGAATTATGT
CTTGGAAACATTGATAGAATATACGATAAAAACCTATTAATGATAAAAGAGCATATTCTT
GCTATTGCAATATATGAATCAAGAATATTGAAACGAAAATATAAGAATAAAGATGATAA
AGAAGTTTGTAAAATCATAAATAAAACTTTCGCTGATATAAGAGATATTATAGGAGGTAC
TGATTATTGGAATGATTTGAGCAATAGAAAATTAGTAGGAAAAATTAACACAAATTCAAA
TTATGTTCACAGGAATAAAGAAAATGATAAGCTTTTTCGTGATGCGTGGTGGAAAGTTAT
TAAAAAAGATGTATGGAATGTGATATCATGGGTATTCAAGGATAAAACTGTTTGTAAAGA
AGATGATATTGAAAATATACCACAATTCTTCAGATGGTTTAGTGAATGGGGTGATGATTA
TTGCCAGGATAAAACAAAAATG
>Thailand 2006 Th11_EBA-175DQ092045.1
TATGTATGTATTCCTGATCGTAGAATCCAATTATGCATTGTTAATCTTAGCATTATTAAAA
CATATACAAAAGAGACCATGAAGGATCATTTCATTGAAGCCTCTAAAAAAGAATCTCAA
CTTTTGCTTAAAAAAAAATGATAACAAATATAATTCTAAATTTTGTAATGATTTGAAGAATA
GTTTTTTAGATTATGGACATCTTGCTATGGGAAATGATATGGATTTTGGAGGTTATTCAAC
TAAGGCAGAAAACAAAATTCAAGAAGTTTTTAAAGGGGCTCATGGGAAAATAAGTGAAC

ATGAAATTAAAAATTTTAGAAAAAAATGGTGGAATGAATTTAGAGAGAAACTTTGGGAA
GCTATGCTATCTGAGCATAAAAATAATATAAATAATTGTAAAAATATTCCCCAAGAAGAA
TTACAAATTACTCAATGGATAAAAGAATGGCATGGAGAATTTTTGCTTGAAAGATATAAT
AGATCAAAATTGCCAAAAAGTAAATGTAAAAATAATACATTATATGAAGCATGTGAGAA
GGAATGTATTGATCCATGTATGAAATATAGAGATTGGATTATTAGAAGTAAATTTGAATG
GCATACGTTATCGAAAGAATATGAAACTCAAAATGTTTCAAAGGAAAATGCGGAAAATT
ATTTAATCAAAATTTCAAAAAACAAGAATGATGCTAAAGTAAGTTTATTATTGAATAATTG
TGATGCTGAATATTCAAAATATTGTGATTGTAAACATACTACTACTCTCGTTAAAAGCGTT
TTAAATGGTAACGACAATACAATTAAGGAAAAGCGTGAACATATTGATTTAGATGATTTT
TCTAAATTTGGATGTGATAAAAATTCCGTTGATACAAACACAAAGGTGTGGGAATGTAAA
AAACCTTATATATTATCCACTAAAGATGTATGTGTACCTCCGAGGAGGCAAGAATTATGT
CTTGGAAACATTGATAGAATATACGATAAAAACCTATTAATGATAAAAGAGCATATTCTT
GCTATTGCAATATATGAATCAAGAATATTGAAACGAAAATATAAGAATAAAGATGATAA
AGAAGTTTGTAAAATCATAAATAAAACTTTCGCTGATATAAGAGATATTATAGGAGGTAC
TGATTATTGGAATGATTTGAGCAATAGAAAATTAGTAGGAAAAATTAACACAAATTCAAA
TTATGTTCACAGGAATAAAAAAAATGATAAGCTTTTTCGTGATGAGTGGTGGAAAGTTAT
TAAAAAAGATGTATGGAATGTGATATCATGGGTATTCAAGGATAAAACTGTTTGTAAAGA
AGATGATATTGAAAATATACCACAATTCTTCAGATGGTTTAGTGAATGGGGTGATGATTA
TTGCCAGGATAAAACAAAAATG
>Thailand 2006 Th10_EBA-175DQ092044.1
TATGTATGTATTCCTGATCGTAGAATCCAATTATGCATTGTTAATCTTAGCATTATTAAAA
CATATACAAAAGAGACCATGAAGGATCATTTCATTGAAGCCTCTAAAAAAGAATCTCAA
CTTTTGCTTAAAAAAAAATGATAACAAATATAATTCTAAATTTTGTAATGATTTGAAGAATA
GTTTTTTAGATTATGGACATCTTGCTATGGGAAATGATATGGATTTTGGAGGTTATTCAAC
TAAGGCAGAAAACAAAATTCAAGAAGTTTTTAAAGGGGCTCATGGGAAAATAAGTGAAC
ATGAAATTAAAAATTTTAGAAAAGAATGGTGGAATGAATTTAGAGAGAAACTTTGGGAA
GCTATGTTATCTGAGCATAAAAATAATATAAATAATTGTAAAAATATTCCCCAAGAAGAA
TTACAAATTACTCAATGGATAAAAGAATGGCATGGAGAATTTTTGCTTGAAAGAGATAAT
AGATCAAAATTGCCAAAAAGTAAATGTAAAAATAATACATTATATGAAGCATGTGAGAA
GGAATGTATTGATCCATGTATGAAATATAGAGATTGGATTATTAGAAGTAAATTTGAATG
GCATACGTTATCGAAAGAATATGAAACTCAAAATGTTTCAAAGGAAAATGCGGAAAATT
ATTTAATCAAAATTTCAGAAAAAATGAATGATGCTAAAGTAAGTTTATTATTGAATAATTG
TGATGCTGAATATTCAAAATATTGTGATTGTAAACATACTACTACTCTCGTTAAAAGCGTT
TTAAATGGTAACGACAATACAATTAAGGAAAAGCGTGAACATATTGATTTAGATGATTTT
TCTAAATTTGGATGTGATAAAAATTCCGTTGATACAAACACAAAGGTGTGGGAATGTAAA
AAACCTTATAAATTATCCACTAAAGATGTATGTGTACCTCCGAGGAGGCAAGAATTATGT
CTTGGAAACATTGATAGAATATACGATAAAAACCTATTAATGATAAAAGAGCATATTCTT
GCTATTGCAATATATGAATCAAGAATATTGAAACGAAAATATAAGAATAAAGATGATAA
AGAAGTTTGTAAAATCATAAATAAAACTTTCGCTGATATAAGAGATATTATAGGAGGTAC
TGATTATTGGAATGATTTGAGCAATAGAAAATTAGTAGGAAAAATTAACACAAATTCAAA
TTATGTTCACAGGAATAAAGAAAATGATAAGCTTTTTCGTGATGCGTGGTGGAAAGTTAT
TAAAAAAGATGTATGGAATGTGATATCATGGGTATTCAAGGATAAAACTGTTTGTAAAGA
AGATGATATTGAAAATATACCACAATTCTTCAGATGGTTTAGTGAATGGGGTGATGATTA
TTGCCAGGATAAAACAAAAATG

>Thailand 2006 Th09_EBA-175DQ092043.1
TATGTATGTATTCCTGATCGTAGAATCCAATTATGCATTGTTAATCTTAGCATTATTAAAA
CATATACAAAAGAGACCATGAAGGATCATTTCATTGAAGCCTCTAAAAAAGAATCTCAA
CTTTTGCTTAAAAAAAAATGATAACAAATATAATTCTAAATTTTGTAATGATTTGAAGAATA
GTTTTTTAGATTATGGACATCTTGCTATGGGAAATGATATGGATTTTGGAGGTTATTCAAC
TAAGGCAGAAAACAAAATTCAAGAAGTTTTTAAAGGGGCTCATGGGAAAATAAGTGAAC
ATGAAATTAAAAATTTTAGAAAAAAAATGGTGGAATGAATTTAGAGAGAAACTTTGGGAA
GCTATGCTATCTGAGCATAAAAATAATATAAATAATTGTAAAAATATTCCCCAAGAAGAA
TTACAAATTACTCAATGGATAAAAGAATGGCATGGAGAATTTTTGCTTGAAAGATATAAT
AGATCAAAATTGCCAAAAAGTAAATGTAAAAATAATACATTATATGAAGCATGTGAGAA
GGAATGTATTGATCCATGTATGAAATATAGAGATTGGATTATTAGAAGTAAATTTGAATG
GCATACGTTATCGAAAGAATATGAAACTCAAAATGTTTCAAAGGAAAATGCGGAAAATT
ATTTAATCAAAATTTCAAAAAACAAGAATGATGCTAAAGTAAGTTTATTATTGAATAATTG
TGATGCTGAATATTCAAAATATTGTGATTGTAAACATACTACTACTCTCGTTAAAAGCGTT
TTAAATGGTAACGACAATACAATTAAGGAAAAGCGTGAACATATTGATTTAGATGATTTT
TCTAAATTTGGATGTGATAAAAATTCCGTTGATACAAACACAAAGGTGTGGGAATGTAAA
AAACCTTATATATTATCCACTAAAGATGTATGTGTACCTCCGAGGAGGCAAGAATTATGT
CTTGGAAACATTGATAGAATATACGATAAAAACCTATTAATGATAAAAGAGCATATTCTT
GCTATTGCAATATATGAATCAAGAATATTGAAACGAAAATATAAGAATAAAGATGATAA
AGAAGTTTGTAAAATCATAAATAAAACTTTCGCTGATATAAGAGATATTATAGGAGGTAC
TGATTATTGGAATGATTTGAGCAATAGAAAATTAGTAGGAAAAATTAACACAAATTCAAA
TTATGTTCACAGGAATAAAAAAAATGATAAGCTTTTTCGTGATGAGTGGTGGAAAGTTAT
TAAAAAAGATGTATGGAATGTGATATCATGGGTATTCAAGGATAAAACTGTTTGTAAAGA
AGATGATATTGAAAATATACCACAATTCTTCAGATGGTTTAGTGAATGGGGTGATGATTA
TTGCCAGGATAAAACAAAAATG
>Thailand 2006 Th08_EBA-175DQ092042.1
TATGTATGTATTCCTGATCGTAGAATCCAATTATGCATTGTTAATCTTAGCATTATTAAAA
CATATACAAAAGAGACCATGAAGGATCATTTCATTGAAGCCTCTAAAAAAGAATCTCAA
CTTTTGCTTAAAAAAAAATGATAACAAATATAATTCTAAATTTTGTAATGATTTGAAGAATA
GTTTTTTAGATTATGGACATCTTGCTATGGGAAATGATATGGATTTTGGAGGTTATTCAAC
TAAGGCAGAAAACAAAATTCAAGAAGTTTTTAAAGGGGCTCATGGGGAAAAAAGTGAA
CATGAAATTAAAAATTTTAGAAAAAAAATGGTGGAATGAATTTAGAGAGAAACTTTGGGA
AGCTATGTTATCTGAGCATAAAAATAATATAAATAATTGTAAAAATATTCCCCAAGAAGA
ATTACAAATTACTCAATGGATAAAAGAATGGCATGGAGAATTTTTGCTTGAAAGAGATAA
TAGATCAAAATTGCCAAAAAGTAAATGTAAAAATAATACATTATATGAAGCATGTGAGA
AGGAATGTATTGATCCATGTATGAAATATAGAGATTGGATTATTAGAAGTAAATTTGAAT
GGCATACGTTATCGAAAGAATATGAAACTCAAAAAGTTTCAAAGGAAAATGCGGAAAAT
TATTTAATCAAAATTTCAAAAAACAAGAATGATGCTAAAGTAAGTTTATTATTGAATAATT
GTGATGCTGAATATTCAAAATATTGTGATTGTAAACATACTACTACTCTCGTTAAAAGCGT
TTTAAATGGTAACGACAATACAATTAAGGAAAAGCGTGAACATATTGATTTAGATGATTT
TTCTAAATTTGGATGTGATAAAAATTCCGTTGATACAAACACAAAGGTGTGGGAATGTAA
AAACCCTTATATATTATCCACTAAAGATGTATGTGTACCTCCGAGGAGGCAAGAATTATG
TCTTGGAAACATTGATAGAATATACGATAAAAACCTATTAATGATAAAAGAGCATATTCT
TGCTATTGCAATATATGAATCAAGAATATTGAAACGAAAATATAAGAATAAAGATGATAA

AGAAGTTTGTAAAATCATAAATAAAACTTTCGCTGATATAAGAGATATTATAGGAGGTAC
TGATTATTGGAATGATTTGAGCAATAGAAAATTAGTAGGAAAAATTAACACAAATTCAAA
TTATGTTCACAGGAATAAAAAAAATGATAAGCTTTTTCGTGATGAGTGGTGGAAAGTTAT
TAAAAAAGATGTATGGAATGTGATATCATGGGTATTCAAGGATAAAACTGTTTGTAAAGA
AGATGATATTGAAAATATACCACAATTCTTCAGATGGTTTAGTGAATGGGGTGATGATTA
TTGCCAGGATAAAACAAAAATG
>Thailand 2006 Th07_EBA-175DQ092041.1
TATGTATGTATTCCTGATCGTAGAATCCAATTATGCATTGTTAATCTTAGCATTATTAAAA
CATATACAAAAGAGACCATGAAGGATCATTTCATTGAAGCCTCTAAAAAAGAATCTCAA
CTTTTGCTTAAAAAAAAATGATAACAAATATAATTCTAAATTTTGTAATGATTTGAAGAATA
GTTTTTTAGATTATGGACATCTTGCTATGGGAAATGATATGGATTTTGGAGGTTATTCAAC
TAAGGCAGAAAACAAAATTCAAGAAGTTTTTAAAGGGGCTCATGGGAAAATAAGTGAAC
ATGAAATTAAAAATTTTAGAAAAAAATGGTGGAATGAATTTAGAGAGAAACTTTGGGAA
GCTATGCTATCTGAGCATAAAAATAATATAAATAATTGTAAAAATATTCCCCAAGAAGAA
TTACAAATTACTCAATGGATAAAAGAATGGCATGGAGAATTTTTGCTTGAAAGATATAAT
AGATCAAAATTGCCAAAAAGTAAATGTAAAAATAATACATTATATGAAGCATGTGAGAA
GGAATGTATTGATCCATGTATGAAATATAGAGATTGGATTATTAGAAGTAAATTTGAATG
GCATACGTTATCGAAAGAATATGAAACTCAAAATGTTTCAAAGGAAAATGCGGAAAATT
ATTTAATCAAAATTTCAAAAAACAAGAATGATGCTAAAGTAAGTTTATTATTGAATAATTG
TGATGCTGAATATTCAAAATATTGTGATTGTAAACATACTACTACTCTCGTTAAAAGCGTT
TTAAATGGTAACGACAATACAATTAAGGAAAAGCGTGAACATATTGATTTAGATGATTTT
TCTAAATTTGGATGTGATAAAAATTCCGTTGATACAAACACAAAGGTGTGGGAATGTAAA
AAACCTTATATATTATCCACTAAAGATGTATGTGTACCTCCGAGGAGGCAAGAATTATGT
CTTGGAAACATTGATAGAATATACGATAAAAACCTATTAATGATAAAAGAGCATATTCTT
GCTATTGCAATATATGAATCAAGAATATTGAAACGAAAATATAAGAATAAAGATGATAA
AGAAGTTTGTAAAATCATAAATAAAACTTTCGCTGATATAAGAGATATTATAGGAGGTAC
TGATTATTGGAATGATTTGAGCAATAGAAAATTAGTAGGAAAAATTAACACAAATTCAAA
TTATGTTCACAGGAATAAAAAAAATGATAAGCTTTTTCGTGATGAGTGGTGGAAAGTTAT
TAAAAAAGATGTATGGAATGTGATATCATGGGTATTCAAGGATAAAACTGTTTGTAAAGA
AGATGATATTGAAAATATACCACAATTCTTCAGATGGTTTAGTGAATGGGGTGATGATTA
TTGCCAGGATAAAACAAAAATG
>Thailand 2006 Th04_EBA-175DQ092040.1
TATGTATGTATTCCTGATCGTAGAATCCAATTATGCATTGTTAATCTTAGCATTATTAAAA
CATATACAAAAGAGACCATGAAGGATCATTTCATTGAAGCCTCTAAAAAAGAATCTCAA
CTTTTGCTTAAAAAAAAATGATAACAAATATAATTCTAAATTTTGTAATGATTTGAAGAATA
GTTTTTTAGATTATGGACATCTTGCTATGGGAAATGATATGGATTTTGGAGGTTATTCAAC
TAAGGCAGAAAACAAAATTCAAGAAGTTTTTAAAGGGGCTCATGGGAAAATAAGTGAAC
ATGAAATTAAAAATTTTAGAAAAAAATGGTGGAATGAATTTAGAGAGAAACTTTGGGAA
GCTATGTTATCTGAGCATAAAAATAATATAAATAATTGTAAAAATATTCCCCAAGAAGAA
TTACAAATTACTCAATGGATAAAAGAATGGCATGGAGAATTTTTGCTTGAAAGAGATAAT
AGATCAAAATTGCCAAAAAGTAAATGTAAAAATAATACATTATATGAAGCATGTGAGAA
GGAATGTATTGATCCATGTATGAAATATAGAGATTGGATTATTAGAAGTAAATTTGAATG
GCATACGTTATCGAAAGAATATGAAACTCAAAAAGTTCCAAAGGAAAATGCGGAAAATT
ATTTAATCAAAATTTCAGAAAACAAGAATGATGCTAAAGTAAGTTTATTATTGAATAATT

GTGATGCTGAATATTCAAAATATTGTGATTGTAAACATACTACTACTCTCGTTAAAAGCGT
TTTAAATGGTAACGACAATACAATTAAGGAAAAGCGTGAACATATTGATTTAGATGATTT
TTCTAAATTTGGATGTGATAAAAATTCCGTTGATACAAACACAAAGGTGTGGGAATGTAA
AAAACCTTATAAATTATCCACTAAAGATGTATGTGTACCTCCGAGGAGGCAAGAATTATG
TCTTGGAAACATTGATAGAATATACGATAAAAACCTATTAATGATAAAAGAGCATATTCT
TGCTATTGCAATATATGAATCAAGAATATTGAAACGAAAATATAAGAATAAAGATGATAA
AGAAGTTTGTAAAATCATAAATAAAACTTTCGCTGATATAAGAGATATTATAGGAGGTAC
TGATTATTGGAATGATTTGAGCAATAGAAAATTAGTAGGAAAAATTAACACAAATTCAAA
TTATGTTCACAGGAATAAAGAAAATGATAAGCTTTTTCGTGATGCGTGGTGGAAAGTTAT
TAAAAAAGATGTATGGAATGTGATATCATGGGTATTCAAGGATAAAACTGTTTGTAAAGA
AGATGATATTGAAAATATACCACAATTCTTCAGATGGTTTAGTGAATGGGGTGATGATTA
TTGCCAGGATAAAACAAAAATG
>Thailand 2006 Th01_EBA-175DQ092039.1
TATGTATGTATTCCTGATCGTAGAATCCAATTATGCATTGTTAATCTTAGCATTATTAAAA
CATATACAAAAGAGACCATGAAGGATCATTTCATTGAAGCCTCTAAAAAAGAATCTCAA
CTTTTGCTTAAAAAAAAATGATAACAAATATAATTCTAAATTTTGTAATGATTTGAAGAATA
GTTTTTTAGATTATGGACATCTTGCTATGGGAAATGATATGGATTTTGGAGGTTATTCAAC
TAAGGCAGAAAACAAAATTCAAGAAGTTTTTAAAGGGGCTCATGGGAAAATAAGTGAAC
ATGAAATTAAAAATTTTAGAAAAGAATGGTGGAATGAATTTAGAGAGAAACTTTGGGAA
GCTATGTTATCTGAGCATAAAAATAATATAAATAATTGTAAAAATATTCCCCAAGAAGAA
TTACAAATTACTCAATGGATAAAAGAATGGCATGGAGAATTTTTGCTTGAAAGAGATAAT
AGATCAAAATTGCCAAAAAGTAAATGTAAAAATAATACATTATATGAAGCATGTGAGAA
GGAATGTATTGATCCATGTATGAAATATAGAGATTGGATTATTAGAAGTAAATTTGAATG
GCATACGTTATCGAAAGAATATGAAACTCAAAATGTTTCAAAGGAAAATGCGGAAAATT
ATTTAATCAAAATTTCAGAAAAAATGAATGATGCTAAAGTAAGTTTATTATTGAATAATTG
TGATGCTGAATATTCAAAATATTGTGATTGTAAACATACTACTACTCTCGTTAAAAGCGTT
TTAAATGGTAACGACAATACAATTAAGGAAAAGCGTGAACATATTGATTTAGATGATTTT
TCTAAATTTGGATGTGATAAAAATTCCGTTGATACAAACACAAAGGTGTGGGAATGTAAA
AAACCTTATAAATTATCCACTAAAGATGTATGTGTACCTCCGAGGAGGCAAGAATTATGT
CTTGGAAACATTGATAGAATATACGATAAAAACCTATTAATGATAAAAGAGCATATTCTT
GCTATTGCAATATATGAATCAAGAATATTGAAACGAAAATATAAGAATAAAGATGATAA
AGAAGTTTGTAAAATCATAAATAAAACTTTCGCTGATATAAGAGATATTATAGGAGGTAC
TGATTATTGGAATGATTTGAGCAATAGAAAATTAGTAGGAAAAATTAACACAAATTCAAA
TTATGTTCACAGGAATAAAGAAAATGATAAGCTTTTTCGTGATGCGTGGTGGAAAGTTAT
TAAAAAAGATGTATGGAATGTGATATCATGGGTATTCAAGGATAAAACTGTTTGTAAAGA
AGATGATATTGAAAATATACCACAATTCTTCAGATGGTTTAGTGAATGGGGTGATGATTA
TTGCCAGGATAAAACAAAAATG
>Thailand 2015 Th-36_EBA-175LC008257.1
TATGTATGTATTCCTGATCGTAGAATCCAATTATGCATTGTTAATCTTAGCATTATTAAAA
CATATACAAAAGAGACCATGAAGGATCATTTCATTGAAGCCTCTAAAAAAGAATCTCAA
CTTTTGCTTAAAAAAAAATGATAACAAATATAATTCTAAATTTTGTAATGATTTGAAGAATA
GTTTTTTAGATTATGGACATCTTGCTATGGGAAATGATATGGATTTTGGAGGTTATTCAAC
TAAGGCAGAAAACAAAATTCAAGAAGTTTTTAAAGGGGCTCATGGGAAAATAAGTGAAC
ATGAAATTAAAAATTTTAGAAAAGAATGGTGGAATGAATTTAGAGAGAAACTTTGGGAA

GCTATGTTATCTGAGCATAAAAATAATATAAATAATTGTAAAAATATTCCCCAAGAAGAA
TTACAAATTACTCAATGGATAAAAGAATGGCATGGAGAATTTTTGCTTGAAAGAGATAAT
AGATCAAAATTGCCAAAAAGTAAATGTAAAAATAATACATTATATGAAGCATGTGAGAA
GGAATGTATTGATCCATGTATGAAATATAGAGATTGGATTATTAGAAGTAAATTTGAATG
GCATACGTTATCGAAAGAATATGAAACTCAAAATGTTTCAAAGGAAAATGCGGAAAATT
ATTTAATCAAAATTTCAGAAAAAATGAATGATGCTAAAGTAAGTTTATTATTGAATAATTG
TGATGCTGAATATTCAAAATATTGTGATTGTAAACATACTACTACTCTCGTTAAAAGCGTT
TTAAATGGTAACGACAATACAATTAAGGAAAAGCGTGAACATATTGATTTAGATGATTTT
TCTAAATTTGGATGTGATAAAAATTCCGTTGATACAAACACAAAGGTGTGGGAATGTAAA
AAACCTTATATATTATCCACTAAAGATGTATGTGTACCTCCGAGGAGGCAAGAATTATGT
CTTGGAAACATTGATAGAATATACGATAAAAACCTATTAATGATAAAAGAGCATATTCTT
GCTATTGCAATATATGAATCAAGAATATTGAAACGAAAATATAAGAATAAAGATGATAA
AGAAGTTTGTAAAATCATAAATAAAACTTTCGCTGATATAAGAGATATTATAGGAGGTAC
TGATTATTGGAATGATTTGAGCAATAGAAAATTAGTAGGAAAAATTAACACAAATTCAAA
TTATGTTCACAGGAATAAAGAAAATGATAAGCTTTTTCGTGATGCGTGGTGGAAAGTTAT
TAAAAAAGATGTATGGAATGTGATATCATGGGTATTCAAGGATAAAACTGTTTGTAAAGA
AGATGATATTGAAAATATACCACAATTCTTCAGATGGTTTAGTGAATGGGGTGATGATTA
TTGCCAGGATAAAACAAAAATG
>Thailand 2015 Th-32_EBA-175LC008263.1
TATGTATGTATTCCTGATCGTAGAATCCAATTATGCATTGTTAATCTTAGCATTATTAAAA
CATATACAAAAGAGACCATGAAGGATCATTTCATTGAAGCCTCTAAAAAAGAATCTCAA
CTTTTGCTTAAAAAAAATGATAACAAATATAATTCTAAATTTTGTAATGATTTGAAGAATA
GTTTTTTAGATTATGGACATCTTGCTATGGGAAATGATATGGATTTTGGAGGTTATTCAAC
TAAGGCAGAAAACAAAATTCAAGAAGTTTTTAAAGGGGCTCATGGGGAAATAAGTGAAC
ATAAAATTAAAAATTTTAGAAAAGAATGGTGGAATGAATTTAGAGAGAAACTTTGGGAA
GCTATGTTATCTGAGCATAAAAATAATATAAATAATTGTAAAAATATTCCCCAAGAAGAA
TTACAAATTACTCAATGGATAAAAGAATGGCATGGAGAATTTTTGCTTGAAAGAGATAAT
AGATCAAAATTGCCAAAAAGTAAATGTAAAAATAATACATTATATGAAGCATGTGAGAA
GGAATGTATTGATCCATGTATGAAATATAGAGATTGGATTATTAGAAGTAAATTTGAATG
GCATACGTTATCGAAAGAATATGAAACTCAAAAAGTTCCAAAGGAAAATGCGGAAAATT
ATTTAATCAAAATTTCAGAAAACATGAATGATGCTAAAGTAAGTTTATTATTGAATAATTG
TGATGCTGAATATTCAAAATATTGTGATTGTAAACATACTACTACTCTCGTTAAAAGCGTT
TTAAATGGTAACGACAATACAATTAAGGAAAAGCGTGAACATATTGATTTAGATGATTTT
TCTAAATTTGGATGTGATAAAAATTCCGTTGATACAAACACAAAGGTGTGGGAATGTAAA
AAACCTTATAAATTATCCACTAAAGATGTATGTGTACCTCCGAGGAGGCAAGAATTATGT
CTTGGAAACATTGATAGAATATACGATAAAAACCTATTAATGATAAAAGAGCATATTCTT
GCTATTGCAATATATGAATCAAGAATATTGAAACGAAAATATAAGAATAAAGATGATAA
AGAAGTTTGTAAAATCATAAATAAAACTTTCGCTGATATAAGAGATATTATAGGAGGTAC
TGATTATTGGAATGATTTGAGCAATAGAAAATTAGTAGGAAAAATTAACACAAATTCAAA
TTATGTTCACAGGAATAAACAAAATGATAAGCTTTTTCGTGATGAGTGGTGGAAAGTTAT
TAAAAAAGATGTATGGAATGTGATATCATGGGTATTCAAGGATAAAACTGTTTGTAAAGA
AGATGATATTGAAAATATACCACAATTCTTCAGATGGTTTAGTGAATGGGGTGATGATTA
TTGCCAGGATAAAACAAAAATG
>Thailand 2015 Th-31_EBA-175LC008262.1

TATGTATGTATTCCTGATCGTAGAATCCAATTATGCATTGTTAATCTTAGCATTATTAAAA
CATATACAAAAGAGACCATGAAGGATCATTTCATTGAAGCCTCTAAAAAAGAATCTCAA
CTTTTGCTTAAAAAAAAATGATAACAAATATAATTCTAAATTTTGTAATGATTTGAAGAATA
GTTTTTTAGATTATGGACATCTTGCTATGGGAAATGATATGGATTTTGGAGGTTATTCAAC
TAAGGCAGAAAACAAAATTCAAGAAGTTTTTAAAGGGGCTCATGGGAAAATAAGTGAAC
ATGAAATTAAAAATTTTAGAAAAGAATGGTGGAATGAATTTAGAGAGAAACTTTGGGAA
GCTATGTTATCTGAGCATAAAAATAATATAAATAATTGTAAAAATATTCCCCAAGAAGAA
TTACAAATTACTCAATGGATAAAAGAATGGCATGGAGAATTTTTGCTTGAAAGAGATAAT
AGATCAAAATTGCCAAAAAGTAAATGTAAAAATAATACATTATATGAAGCATGTGAGAA
GGAATGTATTGATCCATGTATGAAATATAGAGATTGGATTATTAGAAGTAAATTTGAATG
GCATACGTTATCGAAAGAATATGAAACTCAAAATGTTTCAAAGGAAAATGCGGAAAATT
ATTTAATCAAAATTTCAGAAAAAATGAATGATGCTAAAGTAAGTTTATTATTGAATAATTG
TGATGCTGAATATTCAAAATATTGTGATTGTAAACATACTACTACTCTCGTTAAAAGCGTT
TTAAATGGTAACGACAATACAATTAAGGAAAAGCGTGAACATATTGATTTAGATGATTTT
TCTAAATTTGGATGTGATAAAAATTCCGTTGATACAAACACAAAGGTGTGGGAATGTAAA
AAACCTTATAAATTATCCACTAAAGATGTATGTGTACCTCCGAGGAGGCAAGAATTATGT
CTTGGAAACATTGATAGAATATACGATAAAAACCTATTAATGATAAAAGAGCATATTCTT
GCTATTGCAATATATGAATCAAGAATATTGAAACGAAAATATAAGAATAAAGATGATAA
AGAAGTTTGTAAAATCATAAATAAAACTTTCGCTGATATAAGAGATATTATAGGAGGTAC
TGATTATTGGAATGATTTGAGCAATAGAAAATTAGTAGGAAAAATTAACACAAATTCAAA
TTATGTTCACAGGAATAAACAAAATGATAAGCTTTTTCGTGATGAGTGGTGGAAAGTTAT
TAAAAAAGATGTATGGAATGTGATATCATGGGTATTCAAGGATAAAACTGTTTGTAAAGA
AGATGATATTGAAAATATACCACAATTCTTCAGATGGTTTAGTGAATGGGGTGATGATTA
TTGCCAGGATAAAACAAAAATG
>Thailand 2015 Th-30_EBA-175LC008261.1
TATGTATGTATTCCTGATCGTAGAATCCAATTATGCATTGTTAATCTTAGCATTATTAAAA
CATATACAAAAGAGACCATGAAGGATCATTTCATTGAAGCCTCTAAAAAAGAATCTCAA
CTTTTGCTTAAAAAAAAATGATAACAAATATAATTCTAAATTTTGTAATGATTTGAAGAATA
GTTTTTTAGATTATGGACATCTTGCTATGGGAAATGATATGGATTTTGGAGGTTATTCAAC
TAAGGCAGAAAACAAAATTCAAGAAGTTTTTAAAGGGGCTCATGGGGAAATAAGTGAAC
ATAAAATTAAAAATTTTAGAAAAGAATGGTGGAATGAATTTAGAGAGAAACTTTGGGAA
GCTATGTTATCTGAGCATAAAAATAATATAAATAATTGTAAAAATATTCCCCAAGAAGAA
TTACAAATTACTCAATGGATAAAAGAATGGCATGGAGAATTTTTGCTTGAAAGAGATAAT
AGATCAAAATTGCCAAAAAGTAAATGTAAAAATAATACATTATATGAAGCATGTGAGAA
GGAATGTATTGATCCATGTATGAAATATAGAGATTGGATTATTAGAAGTAAATTTGAATG
GCATACGTTATCGAAAGAATATGAAACTCAAAAAGTTCCAAAGGAAAATGCGGAAAATT
ATTTAATCAAAATTTCAGAAAACAAGAATGATGCTAAAGTAAGTTTATTATTGAATAATT
GTGATGCTGAATATTCAAAATATTGTGATTGTAAACATACTACTACTCTCGTTAAAAGCGT
TTTAAATGGTAACGACAATACAATTAAGGAAAAGCGTGAACATATTGATTTAGATGATTT
TTCTAAATTTGGATGTGATAAAAATTCCGTTGATACAAACACAAAGGTGTGGGAATGTAA
AAAACCTTATAAATTATCCACTAAAGATGTATGTGTACCTCCGAGGAGGCAAGAATTATG
TCTTGGAAACATTGATAGAATATACGATAAAAACCTATTAATGATAAAAGAGCATATTCT
TGCTATTGCAATATATGAATCAAGAATATTGAAACGAAAATATAAGAATAAAGATGATAA
AGAAGTTTGTAAAATCATAAATAAAACTTTCGCTGATATAAGAGATATTATAGGAGGTAC

TGATTATTGGAATGATTTGAGCAATAGAAAATTAGTAGGAAAAATTAACACAAATTCAAA
TTATGTTCACAGGAATAAACAAAATGATAAGCTTTTTCGTGATGAGTGGTGGAAAGTTAT
TAAAAAAGATGTATGGAATGTGATATCATGGGTATTCAAGGATAAAACTGTTTGTAAAGA
AGATGATATTGAAAATATACCACAATTCTTCAGATGGTTTAGTGAATGGGGTGATGATTA
TTGCCAGGATAAAACAAAAATG
>Thailand 2015 Th-29_EBA-175LC008260.1
TATGTATGTATTCCTGATCGTAGAATCCAATTATGCATTGTTAATCTTAGCATTATTAAAA
CATATACAAAAGAGACCATGAAGGATCATTTCATTGAAGCCTCTAAAAAAGAATCTCAA
CTTTTGCTTAAAAAAAAATGATAACAAATATAATTCTAAATTTTGTAATGATTTGAAGAATA
GTTTTTTAGATTATGGACATCTTGCTATGGGAAATGATATGGATTTTGGAGGTTATTCAAC
TAAGGCAGAAAACAAAATTCAAGAAGTTTTTAAAGGGGCTCATGGGGAAAAAAGTGAA
CATGAAATTAAAAATTTTAGAAAAAAAATGGTGGAATGAATTTAGAGAGAAACTTTGGGA
AGCTATGTTATCTGAGCATAAAAATAATATAAATAATTGTAAAAATATTCCCCAAGAAGA
ATTACAAATTACTCAATGGATAAAAGAATGGCATGGAGAATTTTTGCTTGAAAGAGATAA
TAGATCAAAATTGCCAAAAAGTAAATGTAAAAATAATACATTATATGAAGCATGTGAGA
AGGAATGTATTGATCCATGTATGAAATATAGAGATTGGATTATTAGAAGTAAATTTGAAT
GGCATACGTTATCGAAAGAATATGAAACTCAAAAAGTTCCAAAGGAAAATGCGGAAAAT
TATTTAATCAAAATTTCAGAAAACAAGAATGATGCTAAAGTAAGTTTATTATTGAATAATT
GTGATGCTGAATATTCAAAATATTGTGATTGTAAACATACTACTACTCTCGTTAAAAGCGT
TTTAAATGGTAACGACAATACAATTAAGGAAAAGCGTGAACATATTGATTTAGATGATTT
TTCTAAATTTGGATGTGATAAAAATTCCGTTGATACAAACACAAAGGTGTGGGAATGTAA
AAAACCTTATAAATTATCCACTAAAGATGTATGTGTACCTCCGAGGAGGCAAGAATTATG
TCTTGGAAACATTGATAGAATATACGATAAAAACCTATTAATGATAAAAGAGCATATTCT
TGCTATTGCAATATATGAATCAAGAATATTGAAACGAAAATATAAGAATAAAGATGATAA
AGAAGTTTGTAAAATCATAAATAAAACTTTCGCTGATATAAGAGATATTATAGGAGGTAC
TGATTATTGGAATGATTTGAGCAATAGAAAATTAGTAGGAAAAATTAACACAAATTCAAA
TTATGTTCACAGGAATAAACAAAATGATAAGCTTTTTCGTGATGAGTGGTGGAAAGTTAT
TAAAAAAGATGTATGGAATGTGATATCATGGGTATTCAAGGATAAAACTGTTTGTAAAGA
AGATGATATTGAAAATATACCACAATTCTTCAGATGGTTTAGTGAATGGGGTGATGATTA
TTGCCAGGATAAAACAAAAATG
>Thailand 2015 Th-28_EBA-175LC008259.1
TATGTATGTATTCCTGATCGTAGAATCCAATTATGCATTGTTAATCTTAGCATTATTAAAA
CATATACAAAAGAGACCATGAAGGATCATTTCATTGAAGCCTCTAAAAAAGAATCTCAA
CTTTTGCTTAAAAAAAAATGATAACAAATATAATTCTAAATTTTGTAATGATTTGAAGAATA
GTTTTTTAGATTATGGACATCTTGCTATGGGAAATGATATGGATTTTGGAGGTTATTCAAC
TAAGGCAGAAAACAAAATTCAAGAAGTTTTTAAAGGGGCTCATGGGGAAAAAAGTGAA
CATGAAATTAAAAATTTTAGAAAAAAAATGGTGGAATGAATTTAGAGAGAAACTTTGGGA
AGCTATGTTATCTGAGCATAAAAATAATATAAATAATTGTAAAAATATTCCCCAAGAAGA
ATTACAAATTACTCAATGGATAAAAGAATGGCATGGAGAATTTTTGCTTGAAAGAGATAA
TAGATCAAAATTGCCAAAAAGTAAATGTAAAAATAATACATTATATGAAGCATGTGAGA
AGGAATGTATTGATCCATGTATGAAATATAGAGATTGGATTATTAGAAGTAAATTTGAAT
GGCATACGTTATCGAAAGAATATGAAACTCAAAAAGTTTCAAAGGAAAATGCGGAAAAT
TATTTAATCAAAATTTCAAAAAACAAGAATGATGCTAAAGTAAGTTTATTATTGAATAATT
GTGATGCTGAATATTCAAAATATTGTGATTGTAAACATACTACTACTCTCGTTAAAAGCGT

TTTAAATGGTAACGACAATACAATTAAGGAAAAGCGTGAACATATTGATTTAGATGATTT
TTCTAAATTTGGATGTGATAAAAATTCCGTTGATACAAACACAAAGGTGTGGGAATGTAA
AAACCCTTATATATTATCCACTAAAGATGTATGTGTACCTCCGAGGAGGCAAGAATTATG
TCTTGGAAACATTGATAGAATATACGATAAAAACCTATTAATGATAAAAGAGCATATTCT
TGCTATTGCAATATATGAATCAAGAATATTGAAACGAAAATATAAGAATAAAGATGATAA
AGAAGTTTGTAAAATCATAAATAAAACTTTCGCTGATATAAGAGATATTATAGGAGGTAC
TGATTATTGGAATGATTTGAGCAATAGAAAATTAGTAGGAAAAATTAACACAAATTCAAA
TTATGTTCACAGGAATAAAAAAAAATGATAAGCTTTTTCGTGATGAGTGGTGGAAAGTTAT
TAAAAAAGATGTATGGAATGTGATATCATGGGTATTCAAGGATAAAACTGTTTGTAAAGA
AGATGATATTGAAAATATACCACAATTCTTCAGATGGTTTAGTGAATGGGGTGATGATTA
TTGCCAGGATAAAACAAAAATG
>Thailand 2015 Th-27_EBA-175LC008258.1
TATGTATGTATTCCTGATCGTAGAATCCAATTATGCATTGTTAATCTTAGCATTATTAAAA
CATATACAAAAGAGACCATGAAGGATCATTTCATTGAAGCCTCTAAAAAAGAATCTCAA
CTTTTGCTTAAAAAAAAATGATAACAAATATAATTCTAAATTTTGTAATGATTTGAAGAATA
GTTTTTTAGATTATGGACATCTTGCTATGGGAAATGATATGGATTTTGGAGGTTATTCAAC
TAAGGCAGAAAACAAAATTCAAGAAGTTTTTAAAGGGGCTCATGGGGAAATAAGTGAAC
ATAAAATTAAAAATTTTAGAAAAGAATGGTGGAATGAATTTAGAGAGAAACTTTGGGAA
GCTATGTTATCTGAGCATAAAAATAATATAAATAATTGTAAAAATATTCCCCAAGAAGAA
TTACAAATTACTCAATGGATAAAAGAATGGCATGGAGAATTTTTGCTTGAAAGAGATAAT
AGATCAAAATTGCCAAAAAGTAAATGTAAAAATAATACATTATATGAAGCATGTGAGAA
GGAATGTATTGATCCATGTATGAAATATAGAGATTGGATTATTAGAAGTAAATTTGAATG
GCATACGTTATCGAAAGAATATGAAACTCAAAAAGTTCCAAAGGAAAATGCGGAAAATT
ATTTAATCAAAATTTCAGAAAACAAGAATGATGCTAAAGTAAGTTTATTATTGAATAATT
GTGATGCTGAATATTCAAAATATTGTGATTGTAAACATACTACTACTCTCGTTAAAAGCGT
TTTAAATGGTAACGACAATACAATTAAGGAAAAGCGTGAACATATTGATTTAGATGATTT
TTCTAAATTTGGATGTGATAAAAATTCCGTTGATACAAACACAAAGGTGTGGGAATGTAA
AAAACCTTATAAATTATCCACTAAAGATGTATGTGTACCTCCGAGGAGGCAAGAATTATG
TCTTGGAAACATTGATAGAATATACGATAAAAACCTATTAATGATAAAAGAGCATATTCT
TGCTATTGCAATATATGAATCAAGAATATTGAAACGAAAATATAAGAATAAAGATGATAA
AGAAGTTTGTAAAATCATAAATAAAACTTTCGCTGATATAAGAGATATTATAGGAGGTAC
TGATTATTGGAATGATTTGAGCAATAGAAAATTAGTAGGAAAAATTAACACAAATTCAAA
TTATGTTCACAGGAATAAACAAAATGATAAGCTTTTTCGTGATGAGTGGTGGAAAGTTAT
TAAAAAAGATGTATGGAATGTGATATCATGGGTATTCAAGGATAAAACTGTTTGTAAAGA
AGATGATATTGAAAATATACCACAATTCTTCAGATGGTTTAGTGAATGGGGTGATGATTA
TTGCCAGGATAAAACAAAAATG
>Thailand 2015 Th-25_EBA-175LC008256.1
TATGTATGTATTCCTGATCGTAGAATCCAATTATGCATTGTTAATCTTAGCATTATTAAAA
CATATACAAAAGAGACCATGAAGGATCATTTCATTGAAGCCTCTAAAAAAGAATCTCAA
CTTTTGCTTAAAAAAAAATGATAACAAATATAATTCTAAATTTTGTAATGATTTGAAGAATA
GTTTTTTAGATTATGGACATCTTGCTATGGGAAATGATATGGATTTTGGAGGTTATTCAAC
TAAGGCAGAAAACAAAATTCAAGAAGTTTTTAAAGGGGCTCATGGGAAAATAAGTGAAC
ATGAAATTAAAAATTTTAGAAAAGAATGGTGGAATGAATTTAGAGAGAAACTTTGGGAA
GCTATGTTATCTGAGCATAAAAATAATATAAATAATTGTAAAAATATTCCCCAAGAAGAA

TTACAAATTACTCAATGGATAAAAGAATGGCATGGAGAATTTTTGCTTGAAAGAGATAAT
AGATCAAAATTGCCAAAAAGTAAATGTAAAAATAATACATTATATGAAGCATGTGAGAA
GGAATGTATTGATCCATGTATGAAATATAGAGATTGGATTATTAGAAGTAAATTTGAATG
GCATACGTTATCGAAAGAATATGAAACTCAAAATGTTTCAAAGGAAAATGCGGAAAATT
ATTTAATCAAAATTTCAGAAAAAAATGAATGATGCTAAAGTAAGTTTATTATTGAATAATTG
TGATGCTGAATATTCAAAATATTGTGATTGTAAACATACTACTACTCTCGTTAAAAGCGTT
TTAAATGGTAACGACAATACAATTAAGGAAAAGCGTGAACATATTGATTTAGATGATTTT
TCTAAATTTGGATGTGATAAAAATTCCGTTGATACAAACACAAAGGTGTGGGAATGTAAA
AAACCTTATAAATTATCCACTAAAGATGTATGTGTACCTCCGAGGAGGCAAGAATTATGT
CTTGGAAACATTGATAGAATATACGATAAAAACCTATTAATGATAAAAGAGCATATTCTT
GCTATTGCAATATATGAATCAAGAATATTGAAACGAAAATATAAGAATAAAGATGATAA
AGAAGTTTGTAAAATCATAAATAAAACTTTCGCTGATATAAGAGATATTATAGGAGGTAC
TGATTATTGGAATGATTTGAGCAATAGAAAATTAGTAGGAAAAATTAACACAAATTCAAA
TTATGTTCACAGGAATAAAGAAAATGATAAGCTTTTTCGTGATGCGTGGTGGAAAGTTAT
TAAAAAAGATGTATGGAATGTGATATCATGGGTATTCAAGGATAAAACTGTTTGTAAAGA
AGATGATATTGAAAATATACCACAATTCTTCAGATGGTTTAGTGAATGGGGTGATGATTA
TTGCCAGGATAAAACAAAAATG
>Thailand 2015 Th-24_EBA-175LC008255.1
TATGTATGTATTCCTGATCGTAGAATCCAATTATGCATTGTTAATCTTAGCATTATTAAAA
CATATACAAAAGAGACCATGAAGGATCATTTCATTGAAGCCTCTAAAAAAGAATCTCAA
CTTTTGCTTAAAAAAAAATGATAACAAATATAATTCTAAATTTTGTAATGATTTGAAGAATA
GTTTTTTAGATTATGGACATCTTGCTATGGGAAATGATATGGATTTTGGAGGTTATTCAAC
TAAGGCAGAAAACAAAATTCAAGAAGTTTTTAAAGGGGCTCATGGGGAAATAAGTGAAC
ATGAAATTAAAAATTTTAGAAAAAAATGGTGGAATGAATTTAGAGAGAAACTTTGGGAA
GCTATGTTATCTGAGCATAAAAATAATATAAATAATTGTAAAAATATTCCCCAAGAAGAA
TTACAAATTACTCAATGGATAAAAGAATGGCATGGAGAATTTTTGCTTGAAAGAGATAAT
AGATCAAAATTGCCAAAAAGTAAATGTAAAAATAATACATTATATGAAGCATGTGAGAA
GGAATGTATTGATCCATGTATGAAATATAGAGATTGGATTATTAGAAGTAAATTTGAATG
GCATACGTTATCGAAAGAATATGAAACTCAAAATGTTTCAAAGGAAAATGCGGAAAATT
ATTTAATCAAAATTTCAGAAAAAAATGAATGATGCTAAAGTAAGTTTATTATTGAATAATTG
TGATGCTGAATATTCAAAATATTGTGATTGTAAACATACTACTACTCTCGTTAAAAGCGTT
TTAAATGGTAACGACAATACAATTAAGGAAAAGCGTGAACATATTGATTTAGATGATTTT
TCTAAATTTGGATGTGATAAAAATTCCGTTGATACAAACACAAAGGTGTGGGAATGTAAA
AAACCTTATAAATTATCCACTAAAGATGTATGTGTACCTCCGAGGAGGCAAGAATTATGT
CTTGGAAACATTGATAGAATATACGATAAAAACCTATTAATGATAAAAGAGCATATTCTT
GCTATTGCAATATATGAATCAAGAATATTGAAACGAAAATATAAGAATAAAGATGATAA
AGAAGTTTGTAAAATCATAAATAAAACTTTCGCTGATATAAGAGATATTATAGGAGGTAC
TGATTATTGGAATGATTTGAGCAATAGAAAATTAGTAGGAAAAATTAACACAAATTCAAA
TTATGTTCACAGGAATAAAGAAAATGATAAGCTTTTTCGTGATGCGTGGTGGAAAGTTAT
TAAAAAAGATGTATGGAATGTGATATCATGGGTATTCAAGGATAAAACTGTTTGTAAAGA
AGATGATATTGAAAATATACCACAATTCTTCAGATGGTTTAGTGAATGGGGTGATGATTA
TTGCCAGGATAAAACAAAAATG
>Thailand 2015 Th-23_EBA-175LC008254.1
TATGTATGTATTCCTGATCGTAGAATCCAATTATGCATTGTTAATCTTAGCATTATTAAAA

CATATACAAAAGAGACCATGAAGGATCATTTCATTGAAGCCTCTAAAAAAGAATCTCAA
CTTTTGCTTAAAAAAAAATGATAACAAATATAATTCTAAATTTTGTAATGATTTGAAGAATA
GTTTTTTAGATTATGGACATCTTGCTATGGGAAATGATATGGATTTTGGAGGTTATTCAAC
TAAGGCAGAAAACAAAATTCAAGAAGTTTTTAAAGGGGCTCATGGGGAAAAAAGTGAA
CATGAAATTAAAAATTTTAGAAAAAAAATGGTGGAATGAATTTAGAGAGAAACTTTGGGA
AGCTATGTTATCTGAGCATAAAAATAATATAAATAATTGTAAAAATATTCCCCAAGAAGA
ATTACAAATTACTCAATGGATAAAAGAATGGCATGGAGAATTTTTGCTTGAAAGAGATAA
TAGATCAAAATTGCCAAAAAGTAAATGTAAAAATAATACATTATATGAAGCATGTGAGA
AGGAATGTATTGATCCATGTATGAAATATAGAGATTGGATTATTAGAAGTAAATTTGAAT
GGCATACGTTATCGAAAGAATATGAAACTCAAAATGTTTCAAAGGAAAATGCGGAAAAT
TATTTAATCAAAATTTCAGAAAAAATGAATGATGCTAAAGTAAGTTTATTATTGAATAATT
GTGATGCTGAATATTCAAAATATTGTGATTGTAAACATACTACTACTCTCGTTAAAAGCGT
TTTAAATGGTAACGACAATACAATTAAGGAAAAGCGTGAACATATTGATTTAGATGATTT
TTCTAAATTTGGATGTGATAAAAATTCCGTTGATACAAACACAAAGGTGTGGGAATGTAA
AAAACCTTATAAATTATCCACTAAAGATGTATGTGTACCTCCGAGGAGGCAAGAATTATG
TCTTGGAAACATTGATAGAATATACGATAAAAACCTATTAATGATAAAAGAGCATATTCT
TGCTATTGCAATATATGAATCAAGAATATTGAAACGAAAATATAAGAATAAAGATGATAA
AGAAGTTTGTAAAATCATAAATAAAACTTTCGCTGATATAAGAGATATTATAGGAGGTAC
TGATTATTGGAATGATTTGAGCAATAGAAAATTAGTAGGAAAAATTAACACAAATTCAAA
TTATGTTCACAGGAATAAAGAAAATGATAAGCTTTTTCGTGATGCGTGGTGGAAAGTTAT
TAAAAAAGATGTATGGAATGTGATATCATGGGTATTCAAGGATAAAACTGTTTGTAAAGA
AGATGATATTGAAAATATACCACAATTCTTCAGATGGTTTAGTGAATGGGGTGATGATTA
TTGCCAGGATAAAACAAAAATG
>Thailand 2015 Th-22_EBA-175LC008253.1
TATGTATGTATTCCTGATCGTAGAATCCAATTATGCATTGTTAATCTTAGCATTATTAAAA
CATATACAAAAGAGACCATGAAGGATCATTTCATTGAAGCCTCTAAAAAAGAATCTCAA
CTTTTGCTTAAAAAAAAATGATAACAAATATAATTCTAAATTTTGTAATGATTTGAAGAATA
GTTTTTTAGATTATGGACATCTTGCTATGGGAAATGATATGGATTTTGGAGGTTATTCAAC
TAAGGCAGAAAACAAAATTCAAGAAGTTTTTAAAGGGGCTCATGGGAAAATAAGTGAAC
ATGAAATTAAAAATTTTAGAAAAGAATGGTGGAATGAATTTAGAGAGAAACTTTGGGAA
GCTATGTTATCTGAGCATAAAAATAATATAAATAATTGTAAAAATATTCCCCAAGAAGAA
TTACAAATTACTCAATGGATAAAAGAATGGCATGGAGAATTTTTGCTTGAAAGAGATAAT
AGATCAAAATTGCCAAAAAGTAAATGTAAAAATAATACATTATATGAAGCATGTGAGAA
GGAATGTATTGATCCATGTATGAAATATAGAGATTGGATTATTAGAAGTAAATTTGAATG
GCATACGTTATCGAAAGAATATGAAACTCAAAAAGTTTCAAAGGAAAATGCGGAAAATT
ATTTAATCAAAATTTCAGAAAAAATGAATGATGCTAAAGTAAGTTTATTATTGAATAATTG
TGATGCTGAATATTCAAAATATTGTGATTGTAAACATACTACTACTCTCGTTAAAAGCGTT
TTAAATGGTAACGACAATACAATTAAGGAAAAGCGTGAACATATTGATTTAGATGATTTT
TCTAAATTTGGATGTGATAAAAATTCCGTTGATACAAACACAAAGGTGTGGGAATGTAAA
AAACCTTATAAATTATCCACTAAAGATGTATGTGTACCTCCGAGGAGGCAAGAATTATGT
CTTGGAAACATTGATAGAATATACGATAAAAACCTATTAATGATAAAAGAGCATATTCTT
GCTATTGCAATATATGAATCAAGAATATTGAAACGAAAATATAAGAATAAAGATGATAA
AGAAGTTTGTAAAATCATAAATAAAACTTTCGCTGATATAAGAGATATTATAGGAGGTAC
TGATTATTGGAATGATTTGAGCAATAGAAAATTAGTAGGAAAAATTAACACAAATTCAAA

TTATGTTCACAGGAATAAAGAAAATGATAAGCTTTTTCGTGATGCGTGGTGGAAAGTTAT
TAAAAAAGATGTATGGAATGTGATATCATGGGTATTCAAGGATAAAACTGTTTGTAAAGA
AGATGATATTGAAAATATACCACAATTCTTCAGATGGTTTAGTGAATGGGGTGATGATTA
TTGCCAGGATAAAACAAAAATG
>Thailand 2015 Th-21_EBA-175LC008252.1
TATGTATGTATTCCTGATCGTAGAATCCAATTATGCATTGTTAATCTTAGCATTATTAAAA
CATATACAAAAGAGACCATGAAGGATCATTTCATTGAAGCCTCTAAAAAAGAATCTCAA
CTTTTGCTTAAAAAAAAATGATAACAAATATAATTCTAAATTTTGTAATGATTTGAAGAATA
GTTTTTTAGATTATGGACATCTTGCTATGGGAAATGATATGGATTTTGGAGGTTATTCAAC
TAAGGCAGAAAACAAAATTCAAGAAGTTTTTAAAGGGGCTCATGGGAAAATAAGTGAAC
ATGAAATTAAAAATTTTAGAAAAGAATGGTGGAATGAATTTAGAGAGAAACTTTGGGAA
GCTATGTTATCTGAGCATAAAAATAATATAAATAATTGTAAAAATATTCCCCAAGAAGAA
TTACAAATTACTCAATGGATAAAAGAATGGCATGGAGAATTTTTGCTTGAAAGAGATAAT
AGATCAAAATTGCCAAAAAGTAAATGTAAAAATAATACATTATATGAAGCATGTGAGAA
GGAATGTATTGATCCATGTATGAAATATAGAGATTGGATTATTAGAAGTAAATTTGAATG
GCATACGTTATCGAAAGAATATGAAACTCAAAATGTTCCAAAGGAAAATGCGGAAAATT
ATTTAATCAAAATTTCAGAAAAAATGAATGATGCTAAAGTAAGTTTATTATTGAATAATTG
TGATGCTGAATATTCAAAATATTGTGATTGTAAACATACTACTACTCTCGTTAAAAGCGTT
TTAAATGGTAACGACAATACAATTAAGGAAAAGCGTGAACATATTGATTTAGATGATTTT
TCTAAATTTGGATGTGATAAAAATTCCGTTGATACAAACACAAAGGTGTGGGAATGTAAA
AAACCTTATAAATTATCCACTAAAGATGTATGTGTACCTCCGAGGAGGCAAGAATTATGT
CTTGGAAACATTGATAGAATATACGATAAAAACCTATTAATGATAAAAGAGCATATTCTT
GCTATTGCAATATATGAATCAAGAATATTGAAACGAAAATATAAGAATAAAGATGATAA
AGAAGTTTGTAAAATCATAAATAAAACTTTCGCTGATATAAGAGATATTATAGGAGGTAC
TGATTATTGGAATGATTTGAGCAATAGAAAATTAGTAGGAAAAATTAACACAAATTCAAA
TTATGTTCACAGGAATAAAGAAAATGATAAGCTTTTTCGTGATGCGTGGTGGAAAGTTAT
TAAAAAAGATGTATGGAATGTGATATCATGGGTATTCAAGGATAAAACTGTTTGTAAAGA
AGATGATATTGAAAATATACCACAATTCTTCAGATGGTTTAGTGAATGGGGTGATGATTA
TTGCCAGGATAAAACAAAAATG
>Thailand 2015 Th-20_EBA-175LC008251.1
TATGTATGTATTCCTGATCGTAGAATCCAATTATGCATTGTTAATCTTAGCATTATTAAAA
CATATACAAAAGAGACCATGAAGGATCATTTCATTGAAGCCTCTAAAAAAGAATCTCAA
CTTTTGCTTAAAAAAAAATGATAACAAATATAATTCTAAATTTTGTAATGATTTGAAGAATA
GTTTTTTAGATTATGGACATCTTGCTATGGGAAATGATATGGATTTTGGAGGTTATTCAAC
TAAGGCAGAAAACAAAATTCAAGAAGTTTTTAAAGGGGCTCATGGGAAAATAAGTGAAC
ATGAAATTAAAAATTTTAGAAAAGAATGGTGGAATGAATTTAGAGAGAAACTTTGGGAA
GCTATGTTATCTGAGCATAAAAATAATATAAATAATTGTAAAAATATTCCCCAAGAAGAA
TTACAAATTACTCAATGGATAAAAGAATGGCATGGAGAATTTTTGCTTGAAAGAGATAAT
AGATCAAAATTGCCAAAAAGTAAATGTAAAAATAATACATTATATGAAGCATGTGAGAA
GGAATGTATTGATCCATGTATGAAATATAGAGATTGGATTATTAGAAGTAAATTTGAATG
GCATACGTTATCGAAAGAATATGAAACTCAAAAAGTTCCAAAGGAAAATGCGGAAAATT
ATTTAATCAAAATTTCAGAAAACAAGAATGATGCTAAAGTAAGTTTATTATTGAATAATT
GTGATGCTGAATATTCAAAATATTGTGATTGTAAACATACTACTACTCTCGTTAAAAGCGT
TTTAAATGGTAACGACAATACAATTAAGGAAAAGCGTGAACATATTGATTTAGATGATTT

TTCTAAATTTGGATGTGATAAAAATTCCGTTGATACAAACACAAAGGTGTGGGAATGTAA
AAAACCTTATAAATTATCCACTAAAGATGTATGTGTACCTCCGAGGAGGCAAGAATTATG
TCTTGGAAACATTGATAGAATATACGATAAAAACCTATTAATGATAAAAGAGCATATTCT
TGCTATTGCAATATATGAATCAAGAATATTGAAACGAAAATATAAGAATAAAGATGATAA
AGAAGTTTGTAAAATCATAAATAAAACTTTCGCTGATATAAGAGATATTATAGGAGGTAC
TGATTATTGGAATGATTTGAGCAATAGAAAATTAGTAGGAAAAATTAACACAAATTCAAA
TTATGTTCACAGGAATAAAGAAAATGATAAGCTTTTTCGTGATGCGTGGTGGAAAGTTAT
TAAAAAAGATGTATGGAATGTGATATCATGGGTATTCAAGGATAAAACTGTTTGTAAAGA
AGATGATATTGAAAATATACCACAATTCTTCAGATGGTTTAGTGAATGGGGTGATGATTA
TTGCCAGGATAAAACAAAAATG
>Thailand 2015 Th-19_EBA-175LC008250.1
TATGTATGTATTCCTGATCGTAGAATCCAATTATGCATTGTTAATCTTAGCATTATTAAAA
CATATACAAAAGAGACCATGAAGGATCATTTCATTGAAGCCTCTAAAAAAGAATCTCAA
CTTTTGCTTAAAAAAAATGATAACAAATATAATTCTAAATTTTGTAATGATTTGAAGAATA
GTTTTTTAGATTATGGACATCTTGCTATGGGAAATGATATGGATTTTGGAGGTTATTCAAC
TAAGGCAGAAAACAAAATTCAAGAAGTTTTTAAAGGGGCTCATGGGAAAATAAGTGAAC
ATGAAATTAAAAATTTTAGAAAAAAATGGTGGAATGAATTTAGAGAGAAACTTTGGGAA
GCTATGTTATCTGAGCATAAAAATAATATAAATAATTGTAAAAATATTCCCCAAGAAGAA
TTACAAATTACTCAATGGATAAAAGAATGGCATGGAGAATTTTTGCTTGAAAGAGATAAT
AGATCAAAATTGCCAAAAAGTAAATGTAAAAATAATACATTATATGAAGCATGTGAGAA
GGAATGTATTGATCCATGTATGAAATATAGAGATTGGATTATTAGAAGTAAATTTGAATG
GCATACGTTATCGAAAGAATATGAAACTCAAAAAGTTCCAAAGGAAAATGCGGAAAATT
ATTTAATCAAAATTTCAGAAAACAAGAATGATGCTAAAGTAAGTTTATTATTGAATAATT
GTGATGCTGAATATTCAAAATATTGTGATTGTAAACATACTACTACTCTCGTTAAAAGCGT
TTTAAATGGTAACGACAATACAATTAAGGAAAAGCGTGAACATATTGATTTAGATGATTT
TTCTAAATTTGGATGTGATAAAAATTCCGTTGATACAAACACAAAGGTGTGGGAATGTAA
AAAACCTTATAAATTATCCACTAAAGATGTATGTGTACCTCCGAGGAGGCAAGAATTATG
TCTTGGAAACATTGATAGAATATACGATAAAAACCTATTAATGATAAAAGAGCATATTCT
TGCTATTGCAATATATGAATCAAGAATATTGAAACGAAAATATAAGAATAAAGATGATAA
AGAAGTTTGTAAAATCATAAATAAAACTTTCGCTGATATAAGAGATATTATAGGAGGTAC
TGATTATTGGAATGATTTGAGCAATAGAAAATTAGTAGGAAAAATTAACACAAATTCAAA
TTATGTTCACAGGAATAAAGAAAATGATAAGCTTTTTCGTGATGCGTGGTGGAAAGTTAT
TAAAAAAGATGTATGGAATGTGATATCATGGGTATTCAAGGATAAAACTGTTTGTAAAGA
AGATGATATTGAAAATATACCACAATTCTTCAGATGGTTTAGTGAATGGGGTGATGATTA
TTGCCAGGATAAAACAAAAATG
>Thailand 2015 Th-18_EBA-175LC008249.1
TATGTATGTATTCCTGATCGTAGAATCCAATTATGCATTGTTAATCTTAGCATTATTAAAA
CATATACAAAAGAGACCATGAAGGATCATTTCATTGAAGCCTCTAAAAAAGAATCTCAA
CTTTTGCTTAAAAAAAATGATAACAAATATAATTCTAAATTTTGTAATGATTTGAAGAATA
GTTTTTTAGATTATGGACATCTTGCTATGGGAAATGATATGGATTTTGGAGGTTATTCAAC
TAAGGCAGAAAACAAAATTCAAGAAGTTTTTAAAGGGGCTCATGGGAAAATAAGTGAAC
ATGAAATTAAAAATTTTAGAAAAAAATGGTGGAATGAATTTAGAGAGAAACTTTGGGAA
GCTATGTTATCTGAGCATAAAAATAATATAAATAATTGTAAAAATATTCCCCAAGAAGAA
TTACAAATTACTCAATGGATAAAAGAATGGCATGGAGAATTTTTGCTTGAAAGAGATAAT

AGATCAAAATTGCCAAAAAGTAAATGTAAAAATAATACATTATATGAAGCATGTGAGAA
GGAATGTATTGATCCATGTATGAAATATAGAGATTGGATTATTAGAAGTAAATTTGAATG
GCATACGTTATCGAAAGAATATGAAACTCAAAATGTTTCAAAGGAAAATGCGGAAAATT
ATTTAATCAAAATTTCAAAAAACAAGAATGATGCTAAAGTAAGTTTATTATTGAATAATTG
TGATGCTGAATATTCAAAATATTGTGATTGTAAACATACTACTACTCTCGTTAAAAGCGTT
TTAAATGGTAACGACAATACAATTAAGGAAAAGCGTGAACATATTGATTTAGATGATTTT
TCTAAATTTGGATGTGATAAAAATTCCGTTGATACAAACACAAAGGTGTGGGAATGTAAA
AAACCTTATAAATTATCCACTAAAGATGTATGTGTACCTCCGAGGAGGCAAGAATTATGT
CTTGGAAACATTGATAGAATATACGATAAAAACCTATTAATGATAAAAGAGCATATTCTT
GCTATTGCAATATATGAATCAAGAATATTGAAACGAAAATATAAGAATAAAGATGATAA
AGAAGTTTGTAAAATCATAAATAAAACTTTCGCTGATATAAGAGATATTATAGGAGGTAC
TGATTATTGGAATGATTTGAGCAATAGAAAATTAGTAGGAAAAATTAACACAAATTCAAA
TTATGTTCACAGGAATAAAGAAAATGATAAGCTTTTTCGTGATGCGTGGTGGAAAGTTAT
TAAAAAAGATGTATGGAATGTGATATCATGGGTATTCAAGGATAAAACTGTTTGTAAAGA
AGATGATATTGAAAATATACCACAATTCTTCAGATGGTTTAGTGAATGGGGTGATGATTA
TTGCCAGGATAAAACAAAAATG
>Thailand 2015 Th-17_EBA-175LC008248.1
TATGTATGTATTCCTGATCGTAGAATCCAATTATGCATTGTTAATCTTAGCATTATTAAAA
CATATACAAAAGAGACCATGAAGGATCATTTCATTGAAGCCTCTAAAAAAGAATCTCAA
CTTTTGCTTAAAAAAAATGATAACAAATATAATTCTAAATTTTGTAATGATTTGAAGAATA
GTTTTTTAGATTATGGACATCTTGCTATGGGAAATGATATGGATTTTGGAGGTTATTCAAC
TAAGGCAGAAAACAAAATTCAAGAAGTTTTTAAAGGGGCTCATGGGAAAATAAGTGAAC
ATGAAATTAAAAATTTTAGAAAAGAATGGTGGAATGAATTTAGAGAGAAACTTTGGGAA
GCTATGTTATCTGAGCATAAAAATAATATAAATAATTGTAAAAATATTCCCCAAGAAGAA
TTACAAATTACTCAATGGATAAAAGAATGGCATGGAGAATTTTTGCTTGAAAGAGATAAT
AGATCAAAATTGCCAAAAAGTAAATGTAAAAATAATACATTATATGAAGCATGTGAGAA
GGAATGTATTGATCCATGTATGAAATATAGAGATTGGATTATTAGAAGTAAATTTGAATG
GCATACGTTATCGAAAGAATATGAAACTCAAAAAGTTTCAAAGGAAAATGCGGAAAATT
ATTTAATCAAAATTTCAGAAAAAATGAATGATGCTAAAGTAAGTTTATTATTGAATAATTG
TGATGCTGAATATTCAAAATATTGTGATTGTAAACATACTACTACTCTCGTTAAAAGCGTT
TTAAATGGTAACGACAATACAATTAAGGAAAAGCGTGAACATATTGATTTAGATGATTTT
TCTAAATTTGGATGTGATAAAAATTCCGTTGATACAAACACAAAGGTGTGGGAATGTAAA
AAACCTTATAAATTATCCACTAAAGATGTATGTGTACCTCCGAGGAGGCAAGAATTATGT
CTTGGAAACATTGATAGAATATACGATAAAAACCTATTAATGATAAAAGAGCATATTCTT
GCTATTGCAATATATGAATCAAGAATATTGAAACGAAAATATAAGAATAAAGATGATAA
AGAAGTTTGTAAAATCATAAATAAAACTTTCGCTGATATAAGAGATATTATAGGAGGTAC
TGATTATTGGAATGATTTGAGCAATAGAAAATTAGTAGGAAAAATTAACACAAATTCAAA
ATATGCTCACAGGAATAAAGAAAATGATAAGCTTTTTCGTGATGCGTGGTGGAAAGTTAT
TAAAAAAGATGTATGGAATGTGATATCATGGGTATTCAAGGATAAAACTGTTTGTAAAGA
AGATGATATTGAAAATATACCACAATTCTTCAGATGGTTTAGTGAATGGGGTGATGATTA
TTGCCAGGATAAAACAAAAATG
>Thailand 2015 Th-16_EBA-175LC008247.1
TATGTATGTATTCCTGATCGTAGAATCCAATTATGCATTGTTAATCTTAGCATTATTAAAA
CATATACAAAAGAGACCATGAAGGATCATTTCATTGAAGCCTCTAAAAAAGAATCTCAA

CTTTTGCTTAAAAAAAATGATAACAAATATAATTCTAAATTTTGTAATGATTTGAAGAATA
GTTTTTTAGATTATGGACATCTTGCTATGGGAAATGATATGGATTTTGGAGGTTATTCAAC
TAAGGCAGAAAACAAAATTCAAGAAGTTTTTAAAGGGGCTCATGGGGAAATAAGTGAAC
ATGAAATTAAAAATTTTAGAAAAAAATGGTGGAATGAATTTAGAGAGAAACTTTGGGAA
GCTATGTTATCTGAGCATAAAAATAATATAAATAATTGTAAAAATATTCCCCAAGAAGAA
TTACAAATTACTCAATGGATAAAAGAATGGCATGGAGAATTTTTGCTTGAAAGAGATAAT
AGATCAAAATTGCCAAAAAGTAAATGTAAAAATAATACATTATATGAAGCATGTGAGAA
GGAATGTATTGATCCATGTATGAAATATAGAGATTGGATTATTAGAAGTAAATTTGAATG
GCATACGTTATCGAAAGAATATGAAACTCAAAATGTTTCAAAGGAAAATGCGGAAAATT
ATTTAATCAAAATTTCAGAAAAAATGAATGATGCTAAAGTAAGTTTATTATTGAATAATTG
TGATGCTGAATATTCAAAATATTGTGATTGTAAACATACTACTACTCTCGTTAAAAGCGTT
TTAAATGGTAACGACAATACAATTAAGGAAAAGCGTGAACATATTGATTTAGATGATTTT
TCTAAATTTGGATGTGATAAAAATTCCGTTGATACAAACACAAAGGTGTGGGAATGTAAA
AAACCTTATAAATTATCCACTAAAGATGTATGTGTACCTCCGAGGAGGCAAGAATTATGT
CTTGGAAACATTGATAGAATATACGATAAAAACCTATTAATGATAAAAGAGCATATTCTT
GCTATTGCAATATATGAATCAAGAATATTGAAACGAAAATATAAGAATAAAGATGATAA
AGAAGTTTGTAAAATCATAAATAAAACTTTCGCTGATATAAGAGATATTATAGGAGGTAC
TGATTATTGGAATGATTTGAGCAATAGAAAATTAGTAGGAAAAATTAACACAAATTCAAA
TTATGTTCACAGGAATAAAGAAAATGATAAGCTTTTTCGTGATGAGTGGTGGAAAGTTAT
TAAAAAAGATGTATGGAATGTGATATCATGGGTATTCAAGGATAAAACTGTTTGTAAAGA
AGATGATATTGAAAATATACCACAATTCTTCAGATGGTTTAGTGAATGGGGTGATGATTA
TTGCCAGGATAAAACAAAAATG
>Thailand 2015 Th-15_EBA-175LC008246.1
TATGTATGTATTCCTGATCGTAGAATCCAATTATGCATTGTTAATCTTAGCATTATTAAAA
CATATACAAAAGAGACCATGAAGGATCATTTCATTGAAGCCTCTAAAAAAGAATCTCAA
CTTTTGCTTAAAAAAAATGATAACAAATATAATTCTAAATTTTGTAATGATTTGAAGAATA
GTTTTTTAGATTATGGACATCTTGCTATGGGAAATGATATGGATTTTGGAGGTTATTCAAC
TAAGGCAGAAAACAAAATTCAAGAAGTTTTTAAAGGGGCTCATGGGAAAATAAGTGAAC
ATGAAATTAAAAATTTTAGAAAAGAATGGTGGAATGAATTTAGAGAGAAACTTTGGGAA
GCTATGTTATCTGAGCATAAAAATAATATAAATAATTGTAAAAATATTCCCCAAGAAGAA
TTACAAATTACTCAATGGATAAAAGAATGGCATGGAGAATTTTTGCTTGAAAGAGATAAT
AGATCAAAATTGCCAAAAAGTAAATGTAAAAATAATACATTATATGAAGCATGTGAGAA
GGAATGTATTGATCCATGTATGAAATATAGAGATTGGATTATTAGAAGTAAATTTGAATG
GCATACGTTATCGAAAGAATATGAAACTCAAAAAGTTCCAAAGGAAAATGCGGAAAATT
ATTTAATCAAAATTTCAGAAAACAAGAATGATGCTAAAGTAAGTTTATTATTGAATAATT
GTGATGCTGAATATTCAAAATATTGTGATTGTAAACATACTACTACTCTCGTTAAAAGCGT
TTTAAATGGTAACGACAATACAATTAAGGAAAAGCGTGAACATATTGATTTAGATGATTT
TTCTAAATTTGGATGTGATAAAAATTCCGTTGATACAAACACAAAGGTGTGGGAATGTAA
AAAACCTTATAAATTATCCACTAAAGATGTATGTGTACCTCCGAGGAGGCAAGAATTATG
TCTTGGAAACATTGATAGAATATACGATAAAAACCTATTAATGATAAAAGAGCATATTCT
TGCTATTGCAATATATGAATCAAGAATATTGAAACGAAAATATAAGAATAAAGATGATAA
AGAAGTTTGTAAAATCATAAATAAAACTTTCGCTGATATAAGAGATATTATAGGAGGTAC
TGATTATTGGAATGATTTGAGCAATAGAAAATTAGTAGGAAAAATTAACACAAATTCAAA
TTATGTTCACAGGAATAAAGAAAATGATAAGCTTTTTCGTGATGCGTGGTGGAAAGTTAT

TAAAAAAGATGTATGGAATGTGATATCATGGGTATTCAAGGATAAAACTGTTTGTAAAGA
AGATGATATTGAAAATATACCACAATTCTTCAGATGGTTTAGTGAATGGGGTGATGATTA
TTGCCAGGATAAAACAAAAATG
>Thailand 2015 Th-14_EBA-175LC008245.1
TATGTATGTATTCCTGATCGTAGAATCCAATTATGCATTGTTAATCTTAGCATTATTAAAA
CATATACAAAAGAGACCATGAAGGATCATTTCATTGAAGCCTCTAAAAAAGAATCTCAA
CTTTTGCTTAAAAAAAAATGATAACAAATATAATTCTAAATTTTGTAATGATTTGAAGAATA
GTTTTTTAGATTATGGACATCTTGCTATGGGAAATGATATGGATTTTGGAGGTTATTCAAC
TAAGGCAGAAAACAAAATTCAAGAAGTTTTTAAAGGGGCTCATGGGAAAATAAGTGAAC
ATGAAATTAAAAATTTTAGAAAAGAATGGTGGAATGAATTTAGAGAGAAACTTTGGGAA
GCTATGTTATCTGAGCATAAAAATAATATAAATAATTGTAAAAATATTCCCCAAGAAGAA
TTACAAATTACTCAATGGATAAAAGAATGGCATGGAGAATTTTTGCTTGAAAGAGATAAT
AGATCAAAATTGCCAAAAAGTAAATGTAAAAATAATACATTATATGAAGCATGTGAGAA
GGAATGTATTGATCCATGTATGAAATATAGAGATTGGATTATTAGAAGTAAATTTGAATG
GCATACGTTATCGAAAGAATATGAAACTCAAAAAGTTCCAAAGGAAAATGCGGAAAATT
ATTTAATCAAAATTTCAGAAAACAAGAATGATGCTAAAGTAAGTTTATTATTGAATAATT
GTGATGCTGAATATTCAAAATATTGTGATTGTAAACATACTACTACTCTCGTTAAAAGCGT
TTTAAATGGTAACGACAATACAATTAAGGAAAAGCGTGAACATATTGATTTAGATGATTT
TTCTAAATTTGGATGTGATAAAAATTCCGTTGATACAAACACAAAGGTGTGGGAATGTAA
AAAACCTTATAAATTATCCACTAAAGATGTATGTGTACCTCCGAGGAGGCAAGAATTATG
TCTTGGAAACATTGATAGAATATACGATAAAAACCTATTAATGATAAAAGAGCATATTCT
TGCTATTGCAATATATGAATCAAGAATATTGAAACGAAAATATAAGAATAAAGATGATAA
AGAAGTTTGTAAAATCATAAATAAAACTTTCGCTGATATAAGAGATATTATAGGAGGTAC
TGATTATTGGAATGATTTGAGCAATAGAAAATTAGTAGGAAAAATTAACACAAATTCAAA
TTATGTTCACAGGAATAAAGAAAATGATAAGCTTTTTCGTGATGCGTGGTGGAAAGTTAT
TAAAAAAGATGTATGGAATGTGATATCATGGGTATTCAAGGATAAAACTGTTTGTAAAGA
AGATGATATTGAAAATATACCACAATTCTTCAGATGGTTTAGTGAATGGGGTGATGATTA
TTGCCAGGATAAAACAAAAATG
>Thailand 2015 Th-13_EBA-175LC008244.1
TATGTATGTATTCCTGATCGTAGAATCCAATTATGCATTGTTAATCTTAGCATTATTAAAA
CATATACAAAAGAGACCATGAAGGATCATTTCATTGAAGCCTCTAAAAAAGAATCTCAA
CTTTTGCTTAAAAAAAAATGATAACAAATATAATTCTAAATTTTGTAATGATTTGAAGAATA
GTTTTTTAGATTATGGACATCTTGCTATGGGAAATGATATGGATTTTGGAGGTTATTCAAC
TAAGGCAGAAAACAAAATTCAAGAAGTTTTTAAAGGGGCTCATGGGAAAATAAGTGAAC
ATGAAATTAAAAATTTTAGAAAAGAATGGTGGAATGAATTTAGAGAGAAACTTTGGGAA
GCTATGTTATCTGAGCATAAAAATAATATAAATAATTGTAAAAATATTCCCCAAGAAGAA
TTACAAATTACTCAATGGATAAAAGAATGGCATGGAGAATTTTTGCTTGAAAGAGATAAT
AGATCAAAATTGCCAAAAAGTAAATGTAAAAATAATACATTATATGAAGCATGTGAGAA
GGAATGTATTGATCCATGTATGAAATATAGAGATTGGATTATTAGAAGTAAATTTGAATG
GCATACGTTATCGAAAGAATATGAAACTCAAAAAGTTCCAAAGGAAAATGCGGAAAATT
ATTTAATCAAAATTTCAGAAAACAAGAATGATGCTAAAGTAAGTTTATTATTGAATAATT
GTGATGCTGAATATTCAAAATATTGTGATTGTAAACATACTACTACTCTCGTTAAAAGCGT
TTTAAATGGTAACGACAATACAATTAAGGAAAAGCGTGAACATATTGATTTAGATGATTT
TTCTAAATTTGGATGTGATAAAAATTCCGTTGATACAAACACAAAGGTGTGGGAATGTAA

AAAACCTTATAAATTATCCACTAAAGATGTATGTGTACCTCCGAGGAGGCAAGAATTATG
TCTTGGAAACATTGATAGAATATACGATAAAAACCTATTAATGATAAAAGAGCATATTCT
TGCTATTGCAATATATGAATCAAGAATATTGAAACGAAAATATAAGAATAAAGATGATAA
AGAAGTTTGTAAAATCATAAATAAAACTTTCGCTGATATAAGAGATATTATAGGAGGTAC
TGATTATTGGAATGATTTGAGCAATAGAAAATTAGTAGGAAAAATTAACACAAATTCAAA
TTATGTTCACAGGAATAAAGAAAATGATAAGCTTTTTCGTGATGCGTGGTGGAAAGTTAT
TAAAAAAGATGTATGGAATGTGATATCATGGGTATTCAAGGATAAAACTGTTTGTAAAGA
AGATGATATTGAAAATATACCACAATTCTTCAGATGGTTTAGTGAATGGGGTGATGATTA
TTGCCAGGATAAAACAAAAATG
>Thailand 2015 Th-12_EBA-175LC008243.1
TATGTATGTATTCCTGATCGTAGAATCCAATTATGCATTGTTAATCTTAGCATTATTAAAA
CATATACAAAAGAGACCATGAAGGATCATTTCATTGAAGCCTCTAAAAAAGAATCTCAA
CTTTTGCTTAAAAAAAAATGATAACAAATATAATTCTAAATTTTGTAATGATTTGAAGAATA
GTTTTTTAGATTATGGACATCTTGCTATGGGAAATGATATGGATTTTGGAGGTTATTCAAC
TAAGGCAGAAAACAAAATTCAAGAAGTTTTTAAAGGGGCTCATGGGAAAATAAGTGAAC
ATGAAATTAAAAATTTTAGAAAAAAATGGTGGAATGAATTTAGAGAGAAACTTTGGGAA
GCTATGTTATCTGAGCATAAAAATAATATAAATAATTGTAAAAATATTCCCCAAGAAGAA
TTACAAATTACTCAATGGATAAAAGAATGGCATGGAGAATTTTTGCTTGAAAGAGATAAT
AGATCAAAATTGCCAAAAAGTAAATGTAAAAATAATACATTATATGAAGCATGTGAGAA
GGAATGTATTGATCCATGTATGAAATATAGAGATTGGATTATTAGAAGTAAATTTGAATG
GCATACGTTATCGAAAGAATATGAAACTCAAAATGTTTCAAAGGAAAATGCGGAAAATT
ATTTAATCAAAATTTCAAAAAACAAGAATGATGCTAAAGTAAGTTTATTATTGAATAATTG
TGATGCTGAATATTCAAAATATTGTGATTGTAAACATACTACTACTCTCGTTAAAAGCGTT
TTAAATGGTAACGACAATACAATTAAGGAAAAGCGTGAACATATTGATTTAGATGATTTT
TCTAAATTTGGATGTGATAAAAATTCCGTTGATACAAACACAAAGGTGTGGGAATGTAAA
AAACCTTATAAATTATCCACTAAAGATGTATGTGTACCTCCGAGGAGGCAAGAATTATGT
CTTGGAAACATTGATAGAATATACGATAAAAACCTATTAATGATAAAAGAGCATATTCTT
GCTATTGCAATATATGAATCAAGAATATTGAAACGAAAATATAAGAATAAAGATGATAA
AGAAGTTTGTAAAATCATAAATAAAACTTTCGCTGATATAAGAGATATTATAGGAGGTAC
TGATTATTGGAATGATTTGAGCAATAGAAAATTAGTAGGAAAAATTAACACAAATTCAAA
TTATGTTCACAGGAATAAAGAAAATGATAAGCTTTTTCGTGATGCGTGGTGGAAAGTTAT
TAAAAAAGATGTATGGAATGTGATATCATGGGTATTCAAGGATAAAACTGTTTGTAAAGA
AGATGATATTGAAAATATACCACAATTCTTCAGATGGTTTAGTGAATGGGGTGATGATTA
TTGCCAGGATAAAACAAAAATG
>Thailand 2015 Th-11_EBA-175LC008242.1
TATGTATGTATTCCTGATCGTAGAATCCAATTATGCATTGTTAATCTTAGCATTATTAAAA
CATATACAAAAGAGACCATGAAGGATCATTTCATTGAAGCCTCTAAAAAAGAATCTCAA
CTTTTGCTTAAAAAAAAATGATAACAAATATAATTCTAAATTTTGTAATGATTTGAAGAATA
GTTTTTTAGATTATGGACATCTTGCTATGGGAAATGATATGGATTTTGGAGGTTATTCAAC
TAAGGCAGAAAACAAAATTCAAGAAGTTTTTAAAGGGGCTCATGGGGAAATAAGTGAAC
ATAAAATTAAAAATTTTAGAAAAGAATGGTGGAATGAATTTAGAGAGAAACTTTGGGAA
GCTATGTTATCTGAGCATAAAAATAATATAAATAATTGTAAAAATATTCCCCAAGAAGAA
TTACAAATTACTCAATGGATAAAAGAATGGCATGGAGAATTTTTGCTTGAAAGAGATAAT
AGATCAAAATTGCCAAAAAGTAAATGTAAAAATAATACATTATATGAAGCATGTGAGAA

GGAATGTATTGATCCATGTATGAAATATAGAGATTGGATTATTAGAAGTAAATTTGAATG
GCATACGTTATCGAAAGAATATGAAACTCAAAAAGTTCCAAAGGAAAATGCGGAAAATT
ATTTAATCAAAATTTCAGAAAACAAGAATGATGCTAAAGTAAGTTTATTATTGAATAATT
GTGATGCTGAATATTCAAAATATTGTGATTGTAAACATACTACTACTCTCGTTAAAAGCGT
TTTAAATGGTAACGACAATACAATTAAGGAAAAGCGTGAACATATTGATTTAGATGATTT
TTCTAAATTTGGATGTGATAAAAATTCCGTTGATACAAACACAAAGGTGTGGGAATGTAA
AAAACCTTATAAATTATCCACTAAAGATGTATGTGTACCTCCGAGGAGGCAAGAATTATG
TCTTGGAAACATTGATAGAATATACGATAAAAACCTATTAATGATAAAAGAGCATATTCT
TGCTATTGCAATATATGAATCAAGAATATTGAAACGAAAATATAAGAATAAAGATGATAA
AGAAGTTTGTAAAATCATAAATAAAACTTTCGCTGATATAAGAGATATTATAGGAGGTAC
TGATTATTGGAATGATTTGAGCAATAGAAAATTAGTAGGAAAAATTAACACAAATTCAAA
TTATGTTCACAGGAATAAACAAAATGATAAGCTTTTTCGTGATGAGTGGTGGAAAGTTAT
TAAAAAAGATGTATGGAATGTGATATCATGGGTATTCAAGGATAAAACTGTTTGTAAAGA
AGATGATATTGAAAATATACCACAATTCTTCAGATGGTTTAGTGAATGGGGTGATGATTA
TTGCCAGGATAAAACAAAAATG
>Thailand 2015 Th-10_EBA-175LC008241.1
TATGTATGTATTCCTGATCGTAGAATCCAATTATGCATTGTTAATCTTAGCATTATTAAAA
CATATACAAAAGAGACCATGAAGGATCATTTCATTGAAGCCTCTAAAAAAGAATCTCAA
CTTTTGCTTAAAAAAAAATGATAACAAATATAATTCTAAATTTTGTAATGATTTGAAGAATA
GTTTTTTAGATTATGGACATCTTGCTATGGGAAATGATATGGATTTTGGAGGTTATTCAAC
TAAGGCAGAAAACAAAATTCAAGAAGTTTTTAAAGGGGCTCATGGGGAAATAAGTGAAC
ATGAAATTAAAAATTTTAGAAAAAAATGGTGGAATGAATTTAGAGAGAAACTTTGGGAA
GCTATGTTATCTGAGCATAAAAATAATATAAATAATTGTAAAAATATTCCCCAAGAAGAA
TTACAAATTACTCAATGGATAAAAGAATGGCATGGAGAATTTTTGCTTGAAAGAGATAAT
AGATCAAAATTGCCAAAAAGTAAATGTAAAAATAATACATTATATGAAGCATGTGAGAA
GGAATGTATTGATCCATGTATGAAATATAGAGATTGGATTATTAGAAGTAAATTTGAATG
GCATACGTTATCGAAAGAATATGAAACTCAAAAAGTTCCAAAGGAAAATGCGGAAAATT
ATTTAATCAAAATTTCAGAAAACAAGAATGATGCTAAAGTAAGTTTATTATTGAATAATT
GTGATGCTGAATATTCAAAATATTGTGATTGTAAACATACTACTACTCTCGTTAAAAGCGT
TTTAAATGGTAACGACAATACAATTAAGGAAAAGCGTGAACATATTGATTTAGATGATTT
TTCTAAATTTGGATGTGATAAAAATTCCGTTGATACAAACACAAAGGTGTGGGAATGTAA
AAACCCTTATATATTATCCACTAAAGATGTATGTGTACCTCCGAGGAGGCAAGAATTATG
TCTTGGAAACATTGATAGAATATACGATAAAAACCTATTAATGATAAAAGAGCATATTCT
TGCTATTGCAATATATGAATCAAGAATATTGAAACGAAAATATAAGAATAAAGATGATAA
AGAAGTTTGTAAAATCATAAATAAAACTTTCGCTGATATAAGAGATATTATAGGAGGTAC
TGATTATTGGAATGATTTGAGCAATAGAAAATTAGTAGGAAAAATTAACACAAATTCAAA
TTATGTTCACAGGAATAAAAAAAATGATAAGCTTTTTCGTGATGAGTGGTGGAAAGTTAT
TAAAAAAGATGTATGGAATGTGATATCATGGGTATTCAAGGATAAAACTGTTTGTAAAGA
AGATGATATTGAAAATATACCACAATTCTTCAGATGGTTTAGTGAATGGGGTGATGATTA
TTGCCAGGATAAAACAAAAATG
>Thailand 2015 Th-9_EBA-175LC008240.1
TATGTATGTATTCCTGATCGTAGAATCCAATTATGCATTGTTAATCTTAGCATTATTAAAA
CATATACAAAAGAGACCATGAAGGATCATTTCATTGAAGCCTCTAAAAAAGAATCTCAA
CTTTTGCTTAAAAAAAAATGATAACAAATATAATTCTAAATTTTGTAATGATTTGAAGAATA

GTTTTTTAGATTATGGACATCTTGCTATGGGAAATGATATGGATTTTGGAGGTTATTCAAC
TAAGGCAGAAAACAAAATTCAAGAAGTTTTTAAAGGGGCTCATGGGGAAAAAAGTGAA
CATGAAATTAAAAATTTTAGAAAAAAATGGTGGAATGAATTTAGAGAGAAACTTTGGGA
AGCTATGTTATCTGAGCATAAAAATAATATAAATAATTGTAAAAATATTCCCCAAGAAGA
ATTACAAATTACTCAATGGATAAAAGAATGGCATGGAGAATTTTTGCTTGAAAGAGATAA
TAGATCAAAATTGCCAAAAAGTAAATGTAAAAATAATACATTATATGAAGCATGTGAGA
AGGAATGTATTGATCCATGTATGAAATATAGAGATTGGATTATTAGAAGTAAATTTGAAT
GGCATACGTTATCGAAAGAATATGAAACTCAAAAAGTTCCAAAGGAAAATGCGGAAAAT
TATTTAATCAAAATTTCAGAAAACAAGAATGATGCTAAAGTAAGTTTATTATTGAATAATT
GTGATGCTGAATATTCAAAATATTGTGATTGTAAACATACTACTACTCTCGTTAAAAGCGT
TTTAAATGGTAACGACAATACAATTAAGGAAAAGCGTGAACATATTGATTTAGATGATTT
TTCTAAATTTGGATGTGATAAAAATTCCGTTGATACAAACACAAAGGTGTGGGAATGTAA
AAACCCTTATATATTATCCACTAAAGATGTATGTGTACCTCCGAGGAGGCAAGAATTATG
TCTTGGAAACATTGATAGAATATACGATAAAAACCTATTAATGATAAAAGAGCATATTCT
TGCTATTGCAATATATGAATCAAGAATATTGAAACGAAAATATAAGAATAAAGATGATAA
AGAAGTTTGTAAAATCATAAATAAAACTTTCGCTGATATAAGAGATATTATAGGAGGTAC
TGATTATTGGAATGATTTGAGCAATAGAAAATTAGTAGGAAAAATTAACACAAATTCAAA
TTATGTTCACAGGAATAAAAAAAATGATAAGCTTTTTCGTGATGAGTGGTGGAAAGTTAT
TAAAAAAGATGTATGGAATGTGATATCATGGGTATTCAAGGATAAAACTGTTTGTAAAGA
AGATGATATTGAAAATATACCACAATTCTTCAGATGGTTTAGTGAATGGGGTGATGATTA
TTGCCAGGATAAAACAAAAATG
>Thailand 2015 Th-8_EBA-175LC008239.1
TATGTATGTATTCCTGATCGTAGAATCCAATTATGCATTGTTAATCTTAGCATTATTAAAA
CATATACAAAAGAGACCATGAAGGATCATTTCATTGAAGCCTCTAAAAAAGAATCTCAA
CTTTTGCTTAAAAAAAATGATAACAAATATAATTCTAAATTTTGTAATGATTTGAAGAATA
GTTTTTTAGATTATGGACATCTTGCTATGGGAAATGATATGGATTTTGGAGGTTATTCAAC
TAAGGCAGAAAACAAAATTCAAGAAGTTTTTAAAGGGGCTCATGGGGAAATAAGTGAAC
ATGAAATTAAAAATTTTAGAAAAAAATGGTGGAATGAATTTAGAGAGAAACTTTGGGAA
GCTATGTTATCTGAGCATAAAAATAATATAAATAATTGTAAAAATATTCCCCAAGAAGAA
TTACAAATTACTCAATGGATAAAAGAATGGCATGGAGAATTTTTGCTTGAAAGAGATAAT
AGATCAAAATTGCCAAAAAGTAAATGTAAAAATAATACATTATATGAAGCATGTGAGAA
GGAATGTATTGATCCATGTATGAAATATAGAGATTGGATTATTAGAAGTAAATTTGAATG
GCATACGTTATCGAAAGAATATGAAACTCAAAATGTTTCAAAGGAAAATGCGGAAAATT
ATTTAATCAAAATTTCAAAAAACAAGAATGATGCTAAAGTAAGTTTATTATTGAATAATTG
TGATGCTGAATATTCAAAATATTGTGATTGTAAACATACTACTACTCTCGTTAAAAGCGTT
TTAAATGGTAACGACAATACAATTAAGGAAAAGCGTGAACATATTGATTTAGATGATTTT
TCTAAATTTGGATGTGATAAAAATTCCGTTGATACAAACACAAAGGTGTGGGAATGTAAA
AACCCTTATATATTATCCACTAAAGATGTATGTGTACCTCCGAGGAGGCAAGAATTATGT
CTTGGAAACATTGATAGAATATACGATAAAAACCTATTAATGATAAAAGAGCATATTCTT
GCTATTGCAATATATGAATCAAGAATATTGAAACGAAAATATAAGAATAAAGATGATAA
AGAAGTTTGTAAAATCATAAATAAAACTTTCGCTGATATAAGAGATATTATAGGAGGTAC
TGATTATTGGAATGATTTGAGCAATAGAAAATTAGTAGGAAAAATTAACACAAATTCAAA
TTATGTTCACAGGAATAAAAAAAATGATAAGCTTTTTCGTGATGAGTGGTGGAAAGTTAT
TAAAAAAGATGTATGGAATGTGATATCATGGGTATTCAAGGATAAAACTGTTTGTAAAGA

AGATGATATTGAAAATATACCACAATTCTTCAGATGGTTTAGTGAATGGGGTGATGATTA
TTGCCAGGATAAAACAAAAATG
>Thailand 2015 Th-7_EBA-175LC008238.1
TATGTATGTATTCCTGATCGTAGAATCCAATTATGCATTGTTAATCTTAGCATTATTAAAA
CATATACAAAAGAGACCATGAAGGATCATTTCATTGAAGCCTCTAAAAAAGAATCTCAA
CTTTTGCTTAAAAAAAATGATAACAAATATAATTCTAAATTTTGTAATGATTTGAAGAATA
GTTTTTTAGATTATGGACATCTTGCTATGGGAAATGATATGGATTTTGGAGGTTATTCAAC
TAAGGCAGAAAACAAAATTCAAGAAGTTTTTAAAGGGGCTCATGGGAAAATAAGTGAAC
ATGAAATTAAAAATTTTAGAAAAAAATGGTGGAATGAATTTAGAGAGAAACTTTGGGAA
GCTATGCTATCTGAGCATAAAAATAATATAAATAATTGTAAAAATATTCCCCAAGAAGAA
TTACAAATTACTCAATGGATAAAAGAATGGCATGGAGAATTTTTGCTTGAAAGATATAAT
AGATCAAAATTGCCAAAAAGTAAATGTAAAAATAATACATTATATGAAGCATGTGAGAA
GGAATGTATTGATCCATGTATGAAATATAGAGATTGGATTATTAGAAGTAAATTTGAATG
GCATACGTTATCGAAAGAATATGAAACTCAAAATGTTTCAAAGGAAAATGCGGAAAATT
ATTTAATCAAAATTTCAAAAAACAAGAATGATGCTAAAGTAAGTTTATTATTGAATAATTG
TGATGCTGAATATTCAAAATATTGTGATTGTAAACATACTACTACTCTCGTTAAAAGCGTT
TTAAATGGTAACGACAATACAATTAAGGAAAAGCGTGAACATATTGATTTAGATGATTTT
TCTAAATTTGGATGTGATAAAAATTCCGTTGATACAAACACAAAGGTGTGGGAATGTAAA
AACCCTTATATATTATCCACTAAAGATGTATGTGTACCTCCGAGGAGGCAAGAATTATGT
CTTGGAAACATTGATAGAATATACGATAAAAACCTATTAATGATAAAAGAGCATATTCTT
GCTATTGCAATATATGAATCAAGAATATTGAAACGAAAATATAAGAATAAAGATGATAA
AGAAGTTTGTAAAATCATAAATAAAACTTTCGCTGATATAAGAGATATTATAGGAGGTAC
TGATTATTGGAATGATTTGAGCAATAGAAAATTAGTAGGAAAAATTAACACAAATTCAAA
TTATGTTCACAGGAATAAAAAAAATGATAAGCTTTTTCGTGATGAGTGGTGGAAAGTTAT
TAAAAAAGATGTATGGAATGTGATATCATGGGTATTCAAGGATAAAACTGTTTGTAAAGA
AGATGATATTGAAAATATACCACAATTCTTCAGATGGTTTAGTGAATGGGGTGATGATTA
TTGCCAGGATAAAACAAAAATG
>Thailand 2015 Th-4_EBA-175LC008237.1
TATGTATGTATTCCTGATCGTAGAATCCAATTATGCATTGTTAATCTTAGCATTATTAAAA
CATATACAAAAGAGACCATGAAGGATCATTTCATTGAAGCCTCTAAAAAAGAATCTCAA
CTTTTGCTTAAAAAAAATGATAACAAATATAATTCTAAATTTTGTAATGATTTGAAGAATA
GTTTTTTAGATTATGGACATCTTGCTATGGGAAATGATATGGATTTTGGAGGTTATTCAAC
TAAGGCAGAAAACAAAATTCAAGAAGTTTTTAAAGGGGCTCATGGGGAAAAAAGTGAA
CATGAAATTAAAAATTTTAGAAAAAAATGGTGGAATGAATTTAGAGAGAAACTTTGGGA
AGCTATGTTATCTGAGCATAAAAATAATATAAATAATTGTAAAAATATTCCCCAAGAAGA
ATTACAAATTACTCAATGGATAAAAGAATGGCATGGAGAATTTTTGCTTGAAAGAGATAA
TAGATCAAAATTGCCAAAAAGTAAATGTAAAAATAATACATTATATGAAGCATGTGAGA
AGGAATGTATTGATCCATGTATGAAATATAGAGATTGGATTATTAGAAGTAAATTTGAAT
GGCATACGTTATCGAAAGAATATGAAACTCAAAAAGTTTCAAAGGAAAATGCGGAAAAT
TATTTAATCAAAATTTCAAAAAACAAGAATGATGCTAAAGTAAGTTTATTATTGAATAATT
GTGATGCTGAATATTCAAAATATTGTGATTGTAAACATACTACTACTCTCGTTAAAAGCGT
TTTAAATGGTAACGACAATACAATTAAGGAAAAGCGTGAACATATTGATTTAGATGATTT
TTCTAAATTTGGATGTGATAAAAATTCCGTTGATACAAACACAAAGGTGTGGGAATGTAA
AAACCCTTATATATTATCCACTAAAGATGTATGTGTACCTCCGAGGAGGCAAGAATTATG

TCTTGGAAACATTGATAGAATATACGATAAAAACCTATTAATGATAAAAGAGCATATTCT
TGCTATTGCAATATATGAATCAAGAATATTGAAACGAAAATATAAGAATAAAGATGATAA
AGAAGTTTGTAAAATCATAAATAAAACTTTCGCTGATATAAGAGATATTATAGGAGGTAC
TGATTATTGGAATGATTTGAGCAATAGAAAATTAGTAGGAAAAATTAACACAAATTCAAA
TTATGTTCACAGGAATAAAAAAAATGATAAGCTTTTTCGTGATGAGTGGTGGAAAGTTAT
TAAAAAAGATGTATGGAATGTGATATCATGGGTATTCAAGGATAAAACTGTTTGTAAAGA
AGATGATATTGAAAATATACCACAATTCTTCAGATGGTTTAGTGAATGGGGTGATGATTA
TTGCCAGGATAAAACAAAAATG
>Thailand 2015 Th-5_EBA-175LC008236.1
TATGTATGTATTCCTGATCGTAGAATCCAATTATGCATTGTTAATCTTAGCATTATTAAAA
CATATACAAAAGAGACCATGAAGGATCATTTCATTGAAGCCTCTAAAAAAGAATCTCAA
CTTTTGCTTAAAAAAAATGATAACAAATATAATTCTAAATTTTGTAATGATTTGAAGAATA
GTTTTTTAGATTATGGACATCTTGCTATGGGAAATGATATGGATTTTGGAGGTTATTCAAC
TAAGGCAGAAAACAAAATTCAAGAAGTTTTTAAAGGGGCTCATGGAAAATAAGTGAAC
ATGAAATTAAAAATTTTAGAAAAAAATGGTGGAATGAATTTAGAGAGAAACTTTGGGAA
GCTATGCTATCTGAGCATAAAAATAATATAAATAATTGTAAAAATATTCCCCAAGAAGAA
TTACAAATTACTCAATGGATAAAAGAATGGCATGGAGAATTTTTGCTTGAAAGATATAAT
AGATCAAAATTGCCAAAAAGTAAATGTAAAAATAATACATTATATGAAGCATGTGAGAA
GGAATGTATTGATCCATGTATGAAATATAGAGATTGGATTATTAGAAGTAAATTTGAATG
GCATACGTTATCGAAAGAATATGAAACTCAAAAAGTTTCAAAGGAAAATGCGGAAAATT
ATTTAATCAAAATTTCAAAAAACAAGAATGATGCTAAAGTAAGTTTATTATTGAATAATTG
TGATGCTGAATATTCAAAATATTGTGATTGTAAACATACTACTACTCTCGTTAAAAGCGTT
TTAAATGGTAACGACAATACAATTAAGGAAAAGCGTGAACATATTGATTTAGATGATTTT
TCTAAATTTGGATGTGATAAAAATTCCGTTGATACAAACACAAAGGTGTGGGAATGTAAA
AAACCTTATATATTATCCACTAAAGATGTATGTGTACCTCCGAGGAGGCAAGAATTATGT
CTTGGAAACATTGATAGAATATACGATAAAAACCTATTAATGATAAAAGAGCATATTCTT
GCTATTGCAATATATGAATCAAGAATATTGAAACGAAAATATAAGAATAAAGATGATAA
AGAAGTTTGTAAAATCATAAATAAAACTTTCGCTGATATAAGAGATATTATAGGAGGTAC
TGATTATTGGAATGATTTGAGCAATAGAAAATTAGTAGGAAAAATTAACACAAATTCAAA
TTATGTTCACAGGAATAAAAAAAATGATAAGCTTTTTCGTGATGAGTGGTGGAAAGTTAT
TAAAAAAGATGTATGGAATGTGATATCATGGGTATTCAAGGATAAAACTGTTTGTAAAGA
AGATGATATTGAAAATATACCACAATTCTTCAGATGGTTTAGTGAATGGGGTGATGATTA
TTGCCAGGATAAAACAAAAATG
>Thailand 2015 Th-4_EBA-175LC008235.1
TATGTATGTATTCCTGATCGTAGAATCCAATTATGCATTGTTAATCTTAGCATTATTAAAA
CATATACAAAAGAGACCATGAAGGATCATTTCATTGAAGCCTCTAAAAAAGAATCTCAA
CTTTTGCTTAAAAAAAATGATAACAAATATAATTCTAAATTTTGTAATGATTTGAAGAATA
GTTTTTTAGATTATGGACATCTTGCTATGGGAAATGATATGGATTTTGGAGGTTATTCAAC
TAAGGCAGAAAACAAAATTCAAGAAGTTTTTAAAGGGGCTCATGGAAAATAAGTGAAC
ATGAAATTAAAAATTTTAGAAAAAAATGGTGGAATGAATTTAGAGAGAAACTTTGGGAA
GCTATGCTATCTGAGCATAAAAATAATATAAATAATTGTAAAAATATTCCCCAAGAAGAA
TTACAAATTACTCAATGGATAAAAGAATGGCATGGAGAATTTTTGCTTGAAAGATATAAT
AGATCAAAATTGCCAAAAAGTAAATGTAAAAATAATACATTATATGAAGCATGTGAGAA
GGAATGTATTGATCCATGTATGAAATATAGAGATTGGATTATTAGAAGTAAATTTGAATG

GCATACGTTATCGAAAGAATATGAAACTCAAAATGTTTCAAAGGAAAATGCGGAAAATT
ATTTAATCAAAATTTCAAAAAACAAGAATGATGCTAAAGTAAGTTTATTATTGAATAATTG
TGATGCTGAATATTCAAAATATTGTGATTGTAAACATACTACTACTCTCGTTAAAAGCGTT
TTAAATGGTAACGACAATACAATTAAGGAAAAGCGTGAACATATTGATTTAGATGATTTT
TCTAAATTTGGATGTGATAAAAATTCCGTTGATACAAACACAAAGGTGTGGGAATGTAAA
AAACCTTATATATTATCCACTAAAGATGTATGTGTACCTCCGAGGAGGCAAGAATTATGT
CTTGGAAACATTGATAGAATATACGATAAAAACCTATTAATGATAAAAGAGCATATTCTT
GCTATTGCAATATATGAATCAAGAATATTGAAACGAAAATATAAGAATAAAGATGATAA
AGAAGTTTGTAAAATCATAAATAAAACTTTCGCTGATATAAGAGATATTATAGGAGGTAC
TGATTATTGGAATGATTTGAGCAATAGAAAATTAGTAGGAAAAATTAACACAAATTCAAA
TTATGTTCACAGGAATAAAAAAAATGATAAGCTTTTTCGTGATGAGTGGTGGAAAGTTAT
TAAAAAAGATGTATGGAATGTGATATCATGGGTATTCAAGGATAAAACTGTTTGTAAAGA
AGATGATATTGAAAATATACCACAATTCTTCAGATGGTTTAGTGAATGGGGTGATGATTA
TTGCCAGGATAAAACAAAAATG
>Thailand 2015 Th-3_EBA-175LC008234.1
TATGTATGTATTCCTGATCGTAGAATCCAATTATGCATTGTTAATCTTAGCATTATTAAAA
CATATACAAAAGAGACCATGAAGGATCATTTCATTGAAGCCTCTAAAAAAGAATCTCAA
CTTTTGCTTAAAAAAAATGATAACAAATATAATTCTAAATTTTGTAATGATTTGAAGAATA
GTTTTTTAGATTATGGACATCTTGCTATGGGAAATGATATGGATTTTGGAGGTTATTCAAC
TAAGGCAGAAAACAAAATTCAAGAAGTTTTTAAAGGGGCTCATGGGGAAATAAGTGAAC
ATGAAATTAAAAATTTTAGAAAAAAATGGTGGAATGAATTTAGAGAGAAACTTTGGGAA
GCTATGTTATCTGAGCATAAAAATAATATAAATAATTGTAAAAATATTCCCCAAGAAGAA
TTACAAATTACTCAATGGATAAAAGAATGGCATGGAGAATTTTTGCTTGAAAGAGATAAT
AGATCAAAATTGCCAAAAAGTAAATGTAAAAATAATACATTATATGAAGCATGTGAGAA
GGAATGTATTGATCCATGTATGAAATATAGAGATTGGATTATTAGAAGTAAATTTGAATG
GCATACGTTATCGAAAGAATATGAAACTCAAAAAGTTCCAAAGGAAAATGCGGAAAATT
ATTTAATCAAAATTTCAGAAAACAAGAATGATGCTAAAGTAAGTTTATTATTGAATAATT
GTGATGCTGAATATTCAAAATATTGTGATTGTAAACATACTACTACTCTCGTTAAAAGCGT
TTTAAATGGTAACGACAATACAATTAAGGAAAAGCGTGAACATATTGATTTAGATGATTT
TTCTAAATTTGGATGTGATAAAAATTCCGTTGATACAAACACAAAGGTGTGGGAATGTAA
AAAACCTTATAAATTATCCACTAAAGATGTATGTGTACCTCCGAGGAGGCAAGAATTATG
TCTTGGAAACATTGATAGAATATACGATAAAAACCTATTAATGATAAAAGAGCATATTCT
TGCTATTGCAATATATGAATCAAGAATATTGAAACGAAAATATAAGAATAAAGATGATAA
AGAAGTTTGTAAAATCATAAATAAAACTTTCGCTGATATAAGAGATATTATAGGAGGTAC
TGATTATTGGAATGATTTGAGCAATAGAAAATTAGTAGGAAAAATTAACACAAATTCAAA
TTATGTTCACAGGAATAAAGAAAATGATAAGCTTTTTCGTGATGCGTGGTGGAAAGTTAT
TAAAAAAGATGTATGGAATGTGATATCATGGGTATTCAAGGATAAAACTGTTTGTAAAGA
AGATGATATTGAAAATATACCACAATTCTTCAGATGGTTTAGTGAATGGGGTGATGATTA
TTGCCAGGATAAAACAAAAATG
>Thailand 2015 Th-2_EBA-175LC008233.1
TATGTATGTATTCCTGATCGTAGAATCCAATTATGCATTGTTAATCTTAGCATTATTAAAA
CATATACAAAAGAGACCATGAAGGATCATTTCATTGAAGCCTCTAAAAAAGAATCTCAA
CTTTTGCTTAAAAAAAATGATAACAAATATAATTCTAAATTTTGTAATGATTTGAAGAATA
GTTTTTTAGATTATGGACATCTTGCTATGGGAAATGATATGGATTTTGGAGGTTATTCAAC

TAAGGCAGAAAACAAAATTCAAGAAGTTTTTAAAGGGGCTCATGGGAAAATAAGTGAAC
ATGAAATTAAAAATTTTAGAAAAAAATGGTGGAATGAATTTAGAGAGAAACTTTGGGAA
GCTATGTTATCTGAGCATAAAAATAATATAAATAATTGTAAAAATATTCCCCAAGAAGAA
TTACAAATTACTCAATGGATAAAAGAATGGCATGGAGAATTTTTGCTTGAAAGAGATAAT
AGATCAAAATTGCCAAAAAGTAAATGTAAAAATAATACATTATATGAAGCATGTGAGAA
GGAATGTATTGATCCATGTATGAAATATAGAGATTGGATTATTAGAAGTAAATTTGAATG
GCATACGTTATCGAAAGAATATGAAACTCAAAATGTTTCAAAGGAAAATGCGGAAAATT
ATTTAATCAAAATTTCAGAAAAAATGAATGATGCTAAAGTAAGTTTATTATTGAATAATTG
TGATGCTGAATATTCAAAATATTGTGATTGTAAACATACTACTACTCTCGTTAAAAGCGTT
TTAAATGGTAACGACAATACAATTAAGGAAAAGCGTGAACATATTGATTTAGATGATTTT
TCTAAATTTGGATGTGATAAAAATTCCGTTGATACAAACACAAAGGTGTGGGAATGTAAA
AAACCTTATAAATTATCCACTAAAGATGTATGTGTACCTCCGAGGAGGCAAGAATTATGT
CTTGGAAACATTGATAGAATATACGATAAAAACCTATTAATGATAAAAGAGCATATTCTT
GCTATTGCAATATATGAATCAAGAATATTGAAACGAAAATATAAGAATAAAGATGATAA
AGAAGTTTGTAAAATCATAAATAAAACTTTCGCTGATATAAGAGATATTATAGGAGGTAC
TGATTATTGGAATGATTTGAGCAATAGAAAATTAGTAGGAAAAATTAACACAAATTCAAA
TTATGTTCACAGGAATAAAGAAAATGATAAGCTTTTTCGTGATGCGTGGTGGAAAGTTAT
TAAAAAAGATGTATGGAATGTGATATCATGGGTATTCAAGGATAAAACTGTTTGTAAAGA
AGATGATATTGAAAATATACCACAATTCTTCAGATGGTTTAGTGAATGGGGTGATGATTA
TTGCCAGGATAAAACAAAAATG
>Thailand 2015 Th-1_EBA-175LC008232.1
TATGTATGTATTCCTGATCGTAGAATCCAATTATGCATTGTTAATCTTAGCATTATTAAAA
CATATACAAAAGAGACCATGAAGGATCATTTCATTGAAGCCTCTAAAAAAGAATCTCAA
CTTTTGCTTAAAAAAAAATGATAACAAATATAATTCTAAATTTTGTAATGATTTGAAGAATA
GTTTTTTAGATTATGGACATCTTGCTATGGGAAATGATATGGATTTTGGAGGTTATTCAAC
TAAGGCAGAAAACAAAATTCAAGAAGTTTTTAAAGGGGCTCATGGGAAAATAAGTGAAC
ATGAAATTAAAAATTTTAGAAAAGAATGGTGGAATGAATTTAGAGAGAAACTTTGGGAA
GCTATGTTATCTGAGCATAAAAATAATATAAATAATTGTAAAAATATTCCCCAAGAAGAA
TTACAAATTACTCAATGGATAAAAGAATGGCATGGAGAATTTTTGCTTGAAAGAGATAAT
AGATCAAAATTGCCAAAAAGTAAATGTAAAAATAATACATTATATGAAGCATGTGAGAA
GGAATGTATTGATCCATGTATGAAATATAGAGATTGGATTATTAGAAGTAAATTTGAATG
GCATACGTTATCGAAAGAATATGAAACTCAAAATGTTTCAAAGGAAAATGCGGAAAATT
ATTTAATCAAAATTTCAGAAAAAATGAATGATGCTAAAGTAAGTTTATTATTGAATAATTG
TGATGCTGAATATTCAAAATATTGTGATTGTAAACATACTACTACTCTCGTTAAAAGCGTT
TTAAATGGTAACGACAATACAATTAAGGAAAAGCGTGAACATATTGATTTAGATGATTTT
TCTAAATTTGGATGTGATAAAAATTCCGTTGATACAAACACAAAGGTGTGGGAATGTAAA
AAACCTTATAAATTATCCACTAAAGATGTATGTGTACCTCCGAGGAGGCAAGAATTATGT
CTTGGAAACATTGATAGAATATACGATAAAAACCTATTAATGATAAAAGAGCATATTCTT
GCTATTGCAATATATGAATCAAGAATATTGAAACGAAAATATAAGAATAAAGATGATAA
AGAAGTTTGTAAAATCATAAATAAAACTTTCGCTGATATAAGAGATATTATAGGAGGTAC
TGATTATTGGAATGATTTGAGCAATAGAAAATTAGTAGGAAAAATTAACACAAATTCAAA
TTATGTTCACAGGAATAAACAAAATGATAAGCTTTTTCGTGATGAGTGGTGGAAAGTTAT
TAAAAAAGATGTATGGAATGTGATATCATGGGTATTCAAGGATAAAACTGTTTGTAAAGA
AGATGATATTGAAAATATACCACAATTCTTCAGATGGTTTAGTGAATGGGGTGATGATTA

TTGCCAGGATAAAACAAAAATG
>Benin Ben26_EBA-175KJ419497.1
TATGTATGTATTCCTGATCGTAGAATCCAATTATGCATTGTTAATCTTAGCATTATTAAAA
CATATACAAAAGAGACCATGAAGGATCATTTCATTGAAGCCTCTAAAAAAGAATCTCAA
CTTTTGCTTAAAAAAAAATGATAACAAATATAATTCTAAATTTTGTAATGATTTGAAGAATA
GTTTTTTAGATTATGGACATCTTGCTATGGGAAATGATATGGATTTTGGAGGTTATTCAAC
TAAGGCAGAAAACAAAATTCAAGAAGTTTTTAAAGGGGCTCATGGGAAAATAAGTGAAC
ATGAAATTAAAAATTTTAGAAAAAAATGGTGGAATGAATTTAGAGAGAAACTTTGGGAA
GCTATGTTATCTGAGCATAAAAATAATATAAATAATTGTAAAAATATTCCCCAAGAAGAA
TTACAAATTACTCAATGGATAAAAGAATGGCATGGAGAATTTTTGCTTGAAAGAGATAAT
AGATCAAAATTGCCAAAAAGTAAATGTAAAAATAATACATTATATGAAGCATGTGAGAA
GGAATGTATTGATCCATGTATGAAATATAGAGATTGGATTATTAGAAGTAAATTTGAATG
GCATACGTTATCGAAAGAATATGAAACTCAAAATGTTTCAAAGGAAAATGCGGAAAATT
ATTTAATCAAAATTTCAAAAAACAAGAATGATGCTAAAGTAAGTTTATTATTGAATAATTG
TGATGCTGAATATTCAAAATATTGTGATTGTAAACATACTACTACTCTCGTTAAAAGCGTT
TTAAATGGTAACGACAATACAATTAAGGAAAAGCGTGAACATATTGATTTAGATGATTTT
TCTAAATTTGGATGTGATAAAAATTCCGTTGATACAAACACAAAGGTGTGGGAATGTAAA
AAACCTTATAAATTATCCACTAAAGATGTATGTGTACCTCCGAGGAGGCAAGAATTATGT
CTTGGAAACATTGATAGAATATACGATAAAAACCTATTAATGATAAAAGAGCATATTCTT
GCTATTGCAATATATGAATCAAGAATATTGAAACGAAAATATAAGAATAAAGATGATAA
AGAAGTTTGTAAAATCATAAATAAAACTTTCGCTGATATAAGAGATATTATAGGAGGTAC
TGATTATTGGAATGATTTGAGCAATAGAAAATTAGTAGGAAAAATTAACACAAATTCAAA
TTATGTTCACAGGAATAAACAAAATGATAAGCTTTTTCGTGATGAGTGGTGGAAAGTTAT
TAAAAAAGATGTATGGAATGTGATATCATGGGTATTCAAGGATAAAACTGTTTGTAAAGA
AGATGATATTGAAAATATACCACAATTCTTCAGATGGTTTAGTGAATGGGGTGATGATTA
TTGCCAGGATAAAACAAAAATG
>Benin Ben28_EBA-175KJ419498.1
TATGTATGTATTCCTGATCGTAGAATCCAATTATGCATTGTTAATCTTAGCATTATTAAAA
CATATACAAAAGAGACCATGAAGGATCATTTCATTGAAGCCTCTAAAAAAGAATCTCAA
CTTTTGCTTAAAAAAAAATGATAACAAATATAATTCTAAATTTTGTAATGATTTGAAGAATA
GTTTTTTAGATTATGGACATCTTGCTATGGGAAATGATATGGATTTTGGAGGTTATTCAAC
TAAGGCAGAAAACAAAATTCAAGAAGTTTTTAAAGGGGCTCATGGGGAAATAAGTGAAC
ATGAAATTAAAAATTTTAGAAAAAAATGGTGGAATGAATTTAGAGAGAAACTTTGGGAA
GCTATGTTATCTGAGCATAAAAATAATATAAATAATTGTAAAAATATTCCCCAAGAAGAA
TTACAAATTACTCAATGGATAAAAGAATGGCATGGAGAATTTTTGCTTGAAAGAGATAAT
AGATCAAAATTGCCAAAAAGTAAATGTAAAAATAATACATTATATGAAGCATGTGAGAA
GGAATGTATTGATCCATGTATGAAATATAGAGATTGGATTATTAGAAGTAAATTTGAATG
GCATACGTTATCGAAAGAATATGAAACTCAAAAAGTTTCAAAGGAAAATGCGGAAAATT
ATTTAATCAAAATTTCAGAAAACAAGAATGATGCTAAAGTAAGTTTATTATTGAATAATT
GTGATGCTGAATATTCAAAATATTGTGATTGTAAACATACTACTACTCTCGTTAAAAGCGT
TTTAAATGGTAACGACAATACAATTAAGGAAAAGCGTGAACATATTGATTTAGATGATTT
TTCTAAATTTGGATGTGATAAAAATTCCGTTGATACAAACACAAAGGTGTGGGAATGTAA
AAAACCTTATATATTATCCACTAAAGATGTATGTGTACCTCCGAGGAGGCAAGAATTATG
TCTTGGAAACATTGATAGAATATACGATAAAAACCTATTAATGATAAAAGAGCATATTCT

TGCTATTGCAATATATGAATCAAGAATATTGAAACGAAAATATAAGAATAAAGATGATAA
AGAAGTTTGTAAAATCATAAATAAAACTTTCGCTGATATAAGAGATATTATAGGAGGTAC
TGATTATTGGAATGATTTGAGCAATAGAAAATTAGTAGGAAAAATTAACACAAATTCAAA
ATATGTTCACAGGAATAAAAAAAATGATAAGCTTTTTCGTGATGAGTGGTGGAAAGTTAT
TAAAAAAGATGTATGGAATGTGATATCATGGGTATTCAAGGATAAAACTGTTTGTAAAGA
AGATGATATTGAAAATATACCACAATTCTTCAGATGGTTTAGTGAATGGGGTGATGATTA
TTGCCAGGATAAAACAAAAATG
>Benin Ben32_EBA-175KJ419499.1
TATGTATGTATTCCTGATCGTAGAATCCAATTATGCATTGTTAATCTTAGCATTATTAAAA
CATATACAAAAGAGACCATGAAGGATCATTTCATTGAAGCCTCTAAAAAAGAATCTCAA
CTTTTGCTTAAAAAAAATGATAACAAATATAATTCTAAATTTTGTAATGATTTGAAGAATA
GTTTTTTAGATTATGGACATCTTGCTATGGGAAATGATATGGATTTTGGAGGTTATTCAAC
TAAGGCAGAAAACAAAATTCAAGAAGTTTTTAAAGGGGCTCATGGGAAAATAAGTGAAC
ATGAAATTAAAAATTTTAGAAAAAAATGGTGGAATGAATTTAGAGAGAAACTTTGGGAA
GCTATGTTATCTGAGCATAAAAATAATATAAATAATTGTAAAAATATTCCCCAAGAAGAA
TTACAAATTACTCAATGGATAAAAGAATGGCATGGAGAATTTTTGCTTGAAAGAGATAAT
AGATCAAAATTGCCAAAAAGTAAATGTAAAAATAATACATTATATGAAGCATGTGAGAA
GGAATGTATTGATCCATGTATGAAATATAGAGATTGGATTATTAGAAGTAAATTTGAATG
GCATACGTTATCGAAAGAATATGAAACTCAAAATGTTTCAAAGGAAAATGCGGAAAATT
ATTTAATCAAAATTTCAAAAAACAAGAATGATGCTAAAGTAAGTTTATTATTGAATAATTG
TGATGCTGAATATTCAAAATATTGTGATTGTAAACATACTACTACTCTCGTTAAAAGCGTT
TTAAATGGTAACGACAATACAATTAAGGAAAAGCGTGAACATATTGATTTAGATGATTTT
TCTAAATTTGGATGTGATAAAAATTCCGTTGATACAAACACAAAGGTGTGGGAATGTAAA
AAACCTTATATATTATCCACTAAAGATGTATGTGTACCTCCGAGGAGGCAAGAATTATGT
CTTGGAAACATTGATAGAATATACGATAAAAACCTATTAATGATAAAAGAGCATATTCTT
GCTATTGCAATATATGAATCAAGAATATTGAAACGAAAATATAAGAATAAAGATGATAA
AGAAGTTTGTAAAATCATAAATAAAACTTTCGCTGATATAAGAGATATTATAGGAGGTAC
TGATTATTGGAATGATTTGAGCAATAGAAAATTAGTAGGAAAAATTAACACAAATTCAAA
TTATGTTCACAGGAATAAACAAAATGATAAGCTTTTTCGTGATGAGTGGTGGAAAGTTAT
TAAAAAAGATGTATGGAATGTGATATCATGGGTATTCAAGGATAAAACTGTTTGTAAAGA
AGATGATATTGAAAATATACCACAATTCTTCAGATGGTTTAGTGAATGGGGTGATGATTA
TTGCCAGGATAAAACAAAAATG
>Benin Ben30_EBA-175KJ419500.1
TATGTATGTATTCCTGATCGTAGAATCCAATTATGCATTGTTAATCTTAGCATTATTAAAA
CATATACAAAAGAGACCATGAAGGATCATTTCATTGAAGCCTCTAAAAAAGAATCTCAA
CTTTTGCTTAAAAAAAATGATAACAAATATAATTCTAAATTTTGTAATGATTTGAAGAATA
GTTTTTTAGATTATGGACATCTTGCTATGGGAAATGATATGGATTTTGGAGGTTATTCAAC
TAAGGCAGAAAACAAAATTCAAGAAGTTTTTAAAGGGGCTCATGGGGAAATAAGTGAAC
ATGAAATTAAAAATTTTAGAAAAAAATGGTGGAATGAATTTAGAGAGAAACTTTGGGAA
GCTATGTTATCTGAGCATAAAAATAATATAAATAATTGTAAAAATATTCCCCAAGAAGAA
TTACAAATTACTCAATGGATAAAAGAATGGCATGGAGAATTTTTGCTTGAAAGAGATAAT
AGATCAAAATTGCCAAAAAGTAAATGTAAAAATAATACATTATATGAAGCATGTGAGAA
GGAATGTATTGATCCATGTATGAAATATAGAGATTGGATTATTAGAAGTAAATTTGAATG
GCATACGTTATCGAAAGAATATGAAACTCAAAAAGTTCCAAAGGAAAATGCGGAAAATT

ATTTAATCAAAATTTCAGAAAACAAGAATGATGCTAAAGTAAGTTTATTATTGAATAATT
GTGATGCTGAATATTCAAAATATTGTGATTGTAAACATACTACTACTCTCGTTAAAAGCGT
TTTAAATGGTAACGACAATACAATTAAGGAAAAGCGTGAACATATTGATTTAGATGATTT
TTCTAAATTTGGATGTGATAAAAATTCCGTTGATACAAACACAAAGGTGTGGGAATGTAA
AAAACCTTATATATTATCCACTAAAGATGTATGTGTACCTCCGAGGAGGCAAGAATTATG
TCTTGGAAACATTGATAGAATATACGATAAAAACCTATTAATGATAAAAGAGCATATTCT
TGCTATTGCAATATATGAATCAAGAATATTGAAACGAAAATATAAGAATAAAGATGATAA
AGAAGTTTGTAAAATCATAAATAAAACTTTCGCTGATATAAGAGATATTATAGGAGGTAC
TGATTATTGGAATGATTTGAGCAATAGAAAATTAGTAGGAAAAATTAACACAAATTCAAA
ATATGTTCACAGGAATAAAAAAAATGATAAGCTTTTTCGTGATGAGTGGTGGAAAGTTAT
TAAAAAAGATGTATGGAATGTGATATCATGGGTATTCAAGGATAAAACTGTTTGTAAAGA
AGATGATATTGAAAATATACCACAATTCTTCAGATGGTTTAGTGAATGGGGTGATGATTA
TTGCCAGGATAAAACAAAAATG
>Benin Ben38_EBA-175KJ419501.1
TATGTATGTATTCCTGATCGTAGAATCCAATTATGCATTGTTAATCTTAGCATTATTAAAA
CATATACAAAAGAGACCATGAAGGATCATTTCATTGAAGCCTCTAAAAAAGAATCTCAA
CTTTTGCTTAAAAAAAAATGATAACAAATATAATTCTAAATTTTGTAATGATTTGAAGAATA
GTTTTTTAGATTATGGACATCTTGCTATGGGAAATGATATGGATTTTGGAGGTTATTCAAC
TAAGGCAGAAAACAAAATTCAAGAAGTTTTTAAAGGGGCTCATGGGGAAATAAGTGAAC
ATGAAATTAAAAATTTTAGAAAAAAAATGGTGGAATGAATTTAGAGAGAAACTTTGGGAA
GCTATGTTATCTGAGCATAAAAATAATATAAATAATTGTAAAAATATTCCCCAAGAAGAA
TTACAAATTACTCAATGGATAAAAGAATGGCATGGAGAATTTTTGCTTGAAAGAGATAAT
AGATCAAAATTGCCAAAAAGTAAATGTAAAAATAATACATTATATGAAGCATGTGAGAA
GGAATGTATTGATCCATGTATGAAATATAGAGATTGGATTATTAGAAGTAAATTTGAATG
GCATACGTTATCGAAAGAATATGAAACTCAAAATGTTTCAAAGGAAAATGCGGAAAATT
ATTTAATCAAAATTTCAAAAAACAAGAATGATGCTAAAGTAAGTTTATTATTGAATAATTG
TGATGCTGAATATTCAAAATATTGTGATTGTAAACATACTACTACTCTCGTTAAAAGCGTT
TTAAATGGTAACGACAATACAATTAAGGAAAAGCGTGAACATATTGATTTAGATGATTTT
TCTAAATTTGGATGTGATAAAAATTCCGTTGATACAAACACAAAGGTGTGGGAATGTAAA
AAACCTTATAAATTATCCACTAAAGATGTATGTGTACCTCCGAGGAGGCAAGAATTATGT
CTTGGAAACATTGATAGAATATACGATAAAAACCTATTAATGATAAAAGAGCATATTCTT
GCTATTGCAATATATGAATCAAGAATATTGAAACGAAAATATAAGAATAAAGATGATAA
AGAAGTTTGTAAAATCATAAATAAAACTTTCGCTGATATAAGAGATATTATAGGAGGTAC
TGATTATTGGAATGATTTGAGCAATAGAAAATTAGTAGGAAAAATTAACACAAATTCAAA
TTATGTTCACAGGAATAAACAAAATGATAAGCTTTTTCGTGATGAGTGGTGGAAAGTTAT
TAAAAAAGATGTATGGAATGTGATATCATGGGTATTCAAGGATAAAACTGTTTGTAAAGA
AGATGATATTGAAAATATACCACAATTCTTCAGATGGTTTAGTGAATGGGGTGATGATTA
TTGCCAGGATAAAACAAAAATG
>Benin Ben44_EBA-175KJ419502.1
TATGTATGTATTCCTGATCGTAGAATCCAATTATGCATTGTTAATCTTAGCATTATTAAAA
CATATACAAAAGAGACCATGAAGGATCATTTCATTGAAGCCTCTAAAAAAGAATCTCAA
CTTTTGCTTAAAAAAAAATGATAACAAATATAATTCTAAATTTTGTAATGATTTGAAGAATA
GTTTTTTAGATTATGGACATCTTGCTATGGGAAATGATATGGATTTTGGAGGTTATTCAAC
TAAGGCAGAAAACAAAATTCAAGAAGTTTTTAAAGGGGCTCATGGGAAAATAAGTGAAC

ATGAAATTAAAAATTTTAGAAAAAAATGGTGGAATGAATTTAGAGAGAAACTTTGGGAA
GCTATGTTATCTGAGCATAAAAATAATATAAATAATTGTAAAAATATTCCCCAAGAAGAA
TTACAAATTACTCAATGGATAAAAGAATGGCATGGAGAATTTTTGCTTGAAAGAGATAAT
AGATCAAAATTGCCAAAAAGTAAATGTAAAAATAATACATTATATGAAGCATGTGAGAA
GGAATGTATTGATCCATGTATGAAATATAGAGATTGGATTATTAGAAGTAAATTTGAATG
GCATACGTTATCGAAAGAATATGAAACTCAAAAAGTTCCAAAGGAAAATGCGGAAAATT
ATTTAATCAAAATTTCAGAAAACAAGAATGATGCTAAAGTAAGTTTATTATTGAATAATT
GTGATGCTGAATATTCAAAATATTGTGATTGTAAACATACTACTACTCTCGTTAAAAGCGT
TTTAAATGGTAACGACAATACAATTAAGGAAAAGCGTGAACATATTGATTTAGATGATTT
TTCTAAATTTGGATGTGATAAAAATTCCGTTGATACAAACACAAAGGTGTGGGAATGTAA
AAAACCTTATAAATTATCCACTAAAGATGTATGTGTACCTCCGAGGAGGCAAGAATTATG
TCTTGGAAACATTGATAGAATATACGATAAAAACCTATTAATGATAAAAGAGCATATTCT
TGCTATTGCAATATATGAATCAAGAATATTGAAACGAAAATATAAGAATAAAGATGATAA
AGAAGTTTGTAAAATCATAAATAAAACTTTCGCTGATATAAGAGATATTATAGGAGGTAC
TGATTATTGGAATGATTTGAGCAATAGAAAATTAGTAGGAAAAATTAACACAAATTCAAA
TTATGTTCACAGGAATAAACAAAATGATAAGCTTTTTCGTGATGAGTGGTGGAAAGTTAT
TAAAAAAGATGTATGGAATGTGATATCATGGGTATTCAAGGATAAAACTGTTTGTAAAGA
AGATGATATTGAAAATATACCACAATTCTTCAGATGGTTTAGTGAATGGGGTGATGATTA
TTGCCAGGATAAAACAAAAATG
>Benin Ben47_EBA-175KJ419503.1
TATGTATGTATTCCTGATCGTAGAATCCAATTATGCATTGTTAATCTTAGCATTATTAAAA
CATATACAAAAGAGACCATGAAGGATCATTTCATTGAAGCCTCTAAAAAAGAATCTCAA
CTTTTGCTTAAAAAAAAATGATAACAAATATAATTCTAAATTTTGTAATGATTTGAAGAATA
GTTTTTTAGATTATGGACATCTTGCTATGGGAAATGATATGGATTTTGGAGGTTATTCAAC
TAAGGCAGAAAACAAAATTCAAGAAGTTTTTAAAGGGGCTCATGGGAAAATAAGTGAAC
ATGAAATTAAAAATTTTAGAAAAAAATGGTGGAATGAATTTAGAGAGAAACTTTGGGAA
GCTATGTTATCTGAGCATAAAAATAATATAAATAATTGTAAAAATATTCCCCAAGAAGAA
TTACAAATTACTCAATGGATAAAAGAATGGCATGGAGAATTTTTGCTTGAAAGATATAAT
AGATCAAAATTGCCAAAAAGTAAATGTAAAAATAATACATTATATGAAGCATGTGAGAA
GGAATGTATTGATCCATGTATGAAATATAGAGATTGGATTATTAGAAGTAAATTTGAATG
GCATACGTTATCGAAAGAATATGAAACTCAAAATGTTTCAAAGGAAAATGCGGAAAATT
ATTTAATCAAAATTTCAAAAAACAAGAATGATGCTAAAGTAAGTTTATTATTGAATAATTG
TGATGCTGAATATTCAAAATATTGTGATTGTAAACATACTACTACTCTCGTTAAAAGCGTT
TTAAATGGTAACGACAATACAATTAAGGAAAAGCGTGAACATATTGATTTAGATGATTTT
TCTAAATTTGGATGTGATAAAAATTCCGTTGATACAAACACAAAGGTGTGGGAATGTAAA
AAACCTTATAAATTATCCACTAAAGATGTATGTGTACCTCCGAGGAGGCAAGAATTATGT
CTTGGAAACATTGATAGAATATACGATAAAAACCTATTAATGATAAAAGAGCATATTCTT
GCTATTGCAATATATGAATCAAGAATATTGAAACGAAAATATAAGAATAAAGATGATAA
AGAAGTTTGTAAAATCATAAATAAAACTTTCGCTGATATAAGAGATATTATAGGAGGTAC
TGATTATTGGAATGATTTGAGCAATAGAAAATTAGTAGGAAAAATTAACACAAATTCAAA
TTATGTTCACAGGAATAAACAAAATGATAAGCTTTTTCGTGATGAGTGGTGGAAAGTTAT
TAAAAAAGATGTATGGAATGTGATATCATGGGTATTCAAGGATAAAACTGTTTGTAAAGA
AGATGATATTGAAAATATACCACAATTCTTCAGATGGTTTAGTGAATGGGGTGATGATTA
TTGCCAGGATAAAACAAAAATG

>Benin Ben62_EBA-175KJ419504.1
TATGTATGTATTCCTGATCGTAGAATCCAATTATGCATTGTTAATCTTAGCATTATTAAAA
CATATACAAAAGAGACCATGAAGGATCATTTCATTGAAGCCTCTAAAAAAGAATCTCAA
CTTTTGCTTAAAAAAAAATGATAACAAATATAATTCTAAATTTTGTAATGATTTGAAGAATA
GTTTTTTAGATTATGGACATCTTGCTATGGGAAATGATATGGATTTTGGAGGTTATTCAAC
TAAGGCAGAAAACAAAATTCAAGAAGTTTTTAAAGGGGCTCATGGGGAAATAAGTGAAC
ATGAAATTAAAAATTTTAGAAAAAAATGGTGGAATGAATTTAGAGAGAAACTTTGGGAA
GCTATGTTATCTGAGCATAAAAATAATATAAATAATTGTAAAAATATTCCCCAAGAAGAA
TTACAAATTACTCAATGGATAAAAGAATGGCATGGAGAATTTTTGCTTGAAAGAGATAAT
AGATCAAAATTGCCAAAAAGTAAATGTAAAAATAATACATTATATGAAGCATGTGAGAA
GGAATGTATTGATCCATGTATGAAATATAGAGATTGGATTATTAGAAGTAAATTTGAATG
GCATACGTTATCGAAAGAATATGAAACTCAAAAAGTTTCAAAGGAAAATGCGGAAAATT
ATTTAATCAAAATTTCAGAAAACAAGAATGATGCTAAAGTAAGTTTATTATTGAATAATT
GTGATGCTGAATATTCAAAATATTGTGATTGTAAACATACTACTACTCTCGTTAAAAGCGT
TTTAAATGGTAACGACAATACAATTAAGGAAAAGCGTGAACATATTGATTTAGATGATTT
TTCTAAATTTGGATGTGATAAAAATTCCGTTGATACAAACACAAAGGTGTGGGAATGTAA
AAAACCTTATATATTATCCACTAAAGATGTATGTGTACCTCCGAGGAGGCAAGAATTATG
TCTTGGAAACATTGATAGAATATACGATAAAAACCTATTAATGATAAAAGAGCATATTCT
TGCTATTGCAATATATGAATCAAGAATATTGAAACGAAAATATAAGAATAAAGATGATAA
AGAAGTTTGTAAAATCATAAATAAAACTTTCGCTGATATAAGAGATATTATAGGAGGTAC
TGATTATTGGAATGATTTGAGCAATAGAAAATTAGTAGGAAAAATTAACACAAATTCAAA
ATATGTTCACAGGAATAAAAAAAATGATAAGCTTTTTCGTGATGAGTGGTGGAAAGTTAT
TAAAAAAGATGTATGGAATGTGATATCATGGGTATTCAAGGATAAAACTGTTTGTAAAGA
AGATGATATTGAAAATATACCACAATTCTTCAGATGGTTTAGTGAATGGGGTGATGATTA
TTGCCAGGATAAAACAAAAATG
>Madagascar Mad7_EBA-175KJ419505.1
TATGTATGTATTCCTGATCGTAGAATCCAATTATGCATTGTTAATCTTAGCATTATTAAAA
CATATACAAAAGAGACCATGAAGGATCATTTCATTGAAGCCTCTAAAAAAGAATCTCAA
CTTTTGCTTAAAAAAAAATGATAACAAATATAATTCTAAATTTTGTAATGATTTGAAGAATA
GTTTTTTAGATTATGGACATCTTGCTATGGGAAATGATATGGATTTTGGAGGTTATTCAAC
TAAGGCAGAAAACAAAATTCAAGAAGTTTTTAAAGGGGCTCATGGGGAAATAAGTGAAC
ATGAAATTAAAAATTTTAGAAAAAAATGGTGGAATGAATTTAGAGAGAAACTTTGGGAA
GCTATGTTATCTGAGCATAAAAATAATATAAATAATTGTAAAAATATTCCCCAAGAAGAA
TTACAAATTACTCAATGGATAAAAGAATGGCATGGAGAATTTTTGCTTGAAAGAGATAAT
AGATCAAAATTGCCAAAAAGTAAATGTAAAAATAATACATTATATGAAGCATGTGAGAA
GGAATGTATTGATCCATGTATGAAATATAGAGATTGGATTATTAGAAGTAAATTTGAATG
GCATACGTTATCGAAAGAATATGAAACTCAAAATGTTTCAAAGGAAAATGCGGAAAATT
ATTTAATCAAAATTTCAAAAAACAAGAATGATGCTAAAGTAAGTTTATTATTGAATAATTG
TGATGCTGAATATTCAAAATATTGTGATTGTAAACATACTACTACTCTCGTTAAAAGCGTT
TTAAATGGTAACGACAATACAATTAAGGAAAAGCGTGAACATATTGATTTAGATGATTTT
TCTAAATTTGGATGTGATAAAAATTCCGTTGATACAAACACAAAGGTGTGGGAATGTAAA
AAACCTTATATATTATCCACTAAAGATGTATGTGTACCTCCGAGGAGGCAAGAATTATGT
CTTGGAAACATTGATAGAATATACGATAAAAACCTATTAATGATAAAAGAGCATATTCTT
GCTATTGCAATATATGAATCAAGAATATTGAAACGAAAATATAAGAATAAAGATGATAA

AGAAGTTTGTAAAATCATAAATAAAACTTTCGCTGATATAAGAGATATTATAGGAGGTAC
TGATTATTGGAATGATTTGAGCAATAGAAAATTAGTAGGAAAAATTAACACAAATTCAAA
TTATGTTCACAGGAATAAACAAAATGATAAGCTTTTTCGTGATGAGTGGTGGAAAGTTAT
TAAAAAAGATGTATGGAATGTGATATCATGGGTATTCAAGGATAAAACTGTTTGTAAAGA
AGATGATATTGAAAATATACCACAATTCTTCAGATGGTTTAGTGAATGGGGTGATGATTA
TTGCCAGGATAAAACAAAAATG
>Madagascar Mad20_EBA-175KJ419506.1
TATGTATGTATTCCTGATCGTAGAATCCAATTATGCATTGTTAATCTTAGCATTATTAAAA
CATATACAAAAGAGACCATGAAGGATCATTTCATTGAAGCCTCTAAAAAAGAATCTCAA
CTTTTGCTTAAAAAAAAATGATAACGAATATAATTCTAAATTTTGTAATGATTTGAAGAATA
GTTTTTTAGATTATGGACATCTTGCTATGGGAAATGATATGGATTTTGGAGGTTATTCAAC
TAAGGCAGAAAACAAAATTCAAGAAGTTTTTAAAGGGGCTCATGGGGAAATAAGTGAAC
ATAAAATTAAAAATTTTAGAAAAGAATGGTGGAATGAATTTAGAGAGAAACTTTGGGAA
GCTATGTTATCTGAGCATAAAAATAATATAAATAATTGTAAAAATATTCCCCAAGAAGAA
TTACAAATTACTCAATGGATAAAAGAATGGCATGGAGAATTTTTGCTTGAAAGAGATAAT
AGATCAAAATTGCCAAAAAGTAAATGTAAAAATAATACATTATATGAAGCATGTGAGAA
GGAATGTATTGATCCATGTATGAAATATAGAGATTGGATTATTAGAAGTAAATTTGAATG
GCATACGTTATCGAAAGAATATGAAACTCAAAATGTTTCAAAGGAAAATGCGGAAAATT
ATTTAATCAAAATTTCAAAAAACAAGAATGATGCTAAAGTAAGTTTATTATTGAATAATTG
TGATGCTGAATATTCAAAATATTGTGATTGTAAACATACTACTACTCTCGTTAAAAGCGTT
TTAAATGGTAACGACAATACAATTAAGGAAAAGCGTGAACATATTGATTTAGATGATTTT
TCTAAATTTGGATGTGATAAAAATTCCGTTGATACAAACACAAAGGTGTGGGAATGTAAA
AAACCTTATAAATTATCCACTAAAGATGTATGTGTACCTCCGAGGAGGCAAGAATTATGT
CTTGGAAACATTGATAGAATATACGATAAAAACCTATTAATGATAAAAGAGCATATTCTT
GCTATTGCAATATATGAATCAAGAATATTGAAACGAAAATATAAGAATAAAGATGATAA
AGAAGTTTGTAAAATCATAAATAAAACTTTCGCTGATATAAGAGATATTATAGGAGGTAC
TGATTATTGGAATGATTTGAGCAATAGAAAATTAGTAGGAAAAATTAACACAAATTCAAA
TTATGTTCACAGGAATAAACAAAATGATAAGCTTTTTCGTGATGAGTGGTGGAAAGTTAT
TAAAAAAGATGTATGGAATGTGATATCATGGGTATTCAAGGATAAAACTGTTTGTAAAGA
AGATGATATTGAAAATATACCACAATTCTTCAGATGGTTTAGTGAATGGGGTGATGATTA
TTGCCAGGATAAAACAAAAATG
>Madagascar Mad25_EBA-175KJ419507.1
TATGTATGTATTCCTGATCGTAGAATCCAATTATGCATTGTTAATCTTAGCATTATTAAAA
CATATACAAAAGAGACCATGAAGGATCATTTCATTGAAGCCTCTAAAAAAGAATCTCAA
CTTTTGCTTAAAAAAAAATGATAACAAATATAATTCTAAATTTTGTAATGATTTGAAGAATA
GTTTTTTAGATTATGGACATCTTGCTATGGGAAATGATATGGATTTTGGAGGTTATTCAAC
TAAGGCAGAAAACAAAATTCAAGAAGTTTTTAAAGGGGCTCATGGGAAAATAAGTGAAC
ATGAAATTAAAAATTTTAGAAAAAAATGGTGGAATGAATTTAGAGAGAAACTTTGGGAA
GCTATGTTATCTGAGCATAAAAATAATATAAATAATTGTAAAAATATTCCCCAAGAAGAA
TTACAAATTACTCAATGGATAAAAGAATGGCATGGAGAATTTTTGCTTGAAAGAGATAAT
AGATCAAAATTGCCAAAAAGTAAATGTAAAAATAATACATTATATGAAGCATGTGAGAA
GGAATGTATTGATCCATGTATGAAATATAGAGATTGGATTATTAGAAGTAAATTTGAATG
GCATACGTTATCGAAAGAATATGAAACTCAAAATGTTTCAAAGGAAAATGCGGAAAATT
ATTTAATCAAAATTTCAAAAAACAAGAATGATGCTAAAGTAAGTTTATTATTGAATAATTG

TGATGCTGAATATTCAAAATATTGTGATTGTAAACATACTACTACTCTCGTTAAAAGCGTT
TTAAATGGTAACGACAATACAATTAAGGAAAAGCGTGAACATATTGATTTAGATGATTTT
TCTAAATTTGGATGTGATAAAAATTCCGTTGATACAAACACAAAGGTGTGGGAATGTAAA
AACCCTTATATATTATCCACTAAAGATGTATGTGTACCTCCGAGGAGGCAAGAATTATGT
CTTGGAAACATTGATAGAATATACGATAAAAACCTATTAATGATAAAAGAGCATATTCTT
GCTATTGCAATATATGAATCAAGAATATTGAAACGAAAATATAAGAATAAAGATGATAA
AGAAGTTTGTAAAATCATAAATAAAACTTTCGCTGATATAAGAGATATTATAGGAGGTAC
TGATTATTGGAATGATTTGAGCAATAGAAAATTAGTAGGAAAAATTAACACAAATTCAAA
TTATGTTCACAGGAATAAAAAAAATGATAAGCTTTTTCGTGATGAGTGGTGGAAAGTTAT
TAAAAAAGATGTATGGAATGTGATATCATGGGTATTCAAGGATAAAACTGTTTGTAAAGA
AGATGATATTGAAAATATACCACAATTCTTCAGATGGTTTAGTGAATGGGGTGATGATTA
TTGCCAGGATAAAACAAAAATG
>Madagascar Mad54_EBA-175KJ419508.1
TATGTATGTATTCCTGATCGTAGAATCCAATTATGCATTGTTAATCTTAGCATTATTAAAA
CATATACAAAAGAGACCATGAAGGATCATTTCATTGAAGCCTCTAAAAAAGAATCTCAA
CTTTTGCTTAAAAAAAAATGATAACAAATATAATTCTAAATTTTGTAATGATTTGAAGAATA
GTTTTTTAGATTATGGACATCTTGCTATGGGAAATGATATGGATTTTGGAGGTTATTCAAC
TAAGGCAGAAAACAAAATTCAAGAAGTTTTTAAAGGGGCTCATGGGGAAATAAGTGAAC
ATAAAATTAAAAATTTTAGAAAAGAATGGTGGAATGAATTTAGAGAGAAACTTTGGGAA
GCTATGTTATCTGAGCATAAAAATAATATAAATAATTGTAAAAATATTCCCCAAGAAGAA
TTACAAATTACTCAATGGATAAAAGAATGGCATGGAGAATTTTTGCTTGAAAGAGATAAT
AGATCAAAATTGCCAAAAAGTAAATGTAAAAATAATACATTATATGAAGCATGTGAGAA
GGAATGTATTGATCCATGTATGAAATATAGAGATTGGATTATTAGAAGTAAATTTGAATG
GCATACGTTATCGAAAGAATATGAAACTCAAAAAGTTCCAAAGGAAAATGCGGAAAATT
ATTTAATCAAAATTTCAGAAAACAAGAATGATGCTAAAGTAAGTTTATTATTGAATAATT
GTGATGCTGAATATTCAAAATATTGTGATTGTAAACATACTACTACTCTCGTTAAAAGCGT
TTTAAATGGTAACGACAATACAATTAAGGAAAAGCGTGAACATATTGATTTAGATGATTT
TTCTAAATTTGGATGTGATAAAAATTCCGTTGATACAAACACAAAGGTGTGGGAATGTAA
AAACCCTTATATATTATCCACTAAAGATGTATGTGTACCTCCGAGGAGGCAAGAATTATG
TCTTGGAAACATTGATAGAATATACGATAAAAACCTATTAATGATAAAAGAGCATATTCT
TGCTATTGCAATATATGAATCAAGAATATTGAAACGAAAATATAAGAATAAAGATGATAA
AGAAGTTTGTAAAATCATAAATAAAACTTTCGCTGATATAAGAGATATTATAGGAGGTAC
TGATTATTGGAATGATTTGAGCAATAGAAAATTAGTAGGAAAAATTAACACAAATTCAAA
TTATGTTCACAGGAATAAAAAAAATGATAAGCTTTTTCGTGATGAGTGGTGGAAAGTTAT
TAAAAAAGATGTATGGAATGTGATATCATGGGTATTCAAGGATAAAACTGTTTGTAAAGA
AGATGATATTGAAAATATACCACAATTCTTCAGATGGTTTAGTGAATGGGGTGATGATTA
TTGCCAGGATAAAACAAAAATG
>Madagascar Mad55_EBA-175KJ419509.1
TATGTATGTATTCCTGATCGTAGAATCCAATTATGCATTGTTAATCTTAGCATTATTAAAA
CATATACAAAAGAGACCATGAAGGATCATTTCATTGAAGCCTCTAAAAAAGAATCTCAA
CTTTTGCTTAAAAAAAAATGATAACAAATATAATTCTAAATTTTGTAATGATTTGAAGAATA
GTTTTTTAGATTATGGACATCTTGCTATGGGAAATGATATGGATTTTGGAGGTTATTCAAC
TAAGGCAGAAAACAAAATTCAAGAAGTTTTTAAAGGGGCTCATGGGGAAATAAGTGAAC
ATGAAATTAAAAATTTTAGAAAAAAATGGTGGAATGAATTTAGAGAGAAACTTTGGGAA

GCTATGTTATCTGAGCATAAAAATAATATAAATAATTGTAAAAATATTCCCCAAGAAGAA
TTACAAATTACTCAATGGATAAAAGAATGGCATGGAGAATTTTTGCTTGAAAGAGATAAT
AGATCAAAATTGCCAAAAAGTAAATGTAAAAATAATACATTATATGAAGCATGTGAGAA
GGAATGTATTGATCCATGTATGAAATATAGAGATTGGATTATTAGAAGTAAATTTGAATG
GCATACGTTATCGAAAGAATATGAAACTCAAAATGTTTCAAAGGAAAATGCGGAAAATT
ATTTAATCAAAATTTCAAAAAACAAGAATGATGCTAAAGTAAGTTTATTATTGAATAATTG
TGATGCTGAATATTCAAAATATTGTGATTGTAAACATACTACTACTCTCGTTAAAAGCGTT
TTAAATGGTAACGACAATACAATTAAGGAAAAGCGTGAACATATTGATTTAGATGATTTT
TCTAAATTTGGATGTGATAAAAATTCCGTTGATACAAACACAAAGGTGTGGGAATGTAAA
AACCCTTATATATTATCCACTAAAGATGTATGTGTACCTCCGAGGAGGCAAGAATTATGT
CTTGGAAACATTGATAGAATATACGATAAAAACCTATTAATGATAAAAGAGCATATTCTT
GCTATTGCAATATATGAATCAAGAATATTGAAACGAAAATATAAGAATAAAGATGATAA
AGAAGTTTGTAAAATCATAAATAAAACTTTCGCTGATATAAGAGATATTATAGGAGGTAC
TGATTATTGGAATGATTTGAGCAATAGAAAATTAGTAGGAAAAATTAACACAAATTCAAA
TTATGTTCACAGGAATAAAAAAAAATGATAAGCTTTTTCGTGATGAGTGGTGGAAAGTTAT
TAAAAAAGATGTATGGAATGTGATATCATGGGTATTCAAGGATAAAACTGTTTGTAAAGA
AGATGATATTGAAAATATACCACAATTCTTCAGATGGTTTAGTGAATGGGGTGATGATTA
TTGCCAGGATAAAACAAAAATG
>Madagascar Mad56_EBA-175KJ419510.1
TATGTATGTATTCCTGATCGTAGAATCCAATTATGCATTGTTAATCTTAGCATTATTAAAA
CATATACAAAAGAGACCATGAAGGATCATTTCATTGAAGCCTCTAAAAAAGAATCTCAA
CTTTTGCTTAAAAAAAAATGATAACGAATATAATTCTAAATTTTGTAATGATTTGAAGAATA
GTTTTTTAGATTATGGACATCTTGCTATGGGAAATGATATGGATTTTGGAGGTTATTCAAC
TAAGGCAGAAAACAAAATTCAAGAAGTTTTTAAAGGGGCTCATGGGGAAATAAGTGAAC
ATAAAATTAAAAATTTTAGAAAAGAATGGTGGAATGAATTTAGAGAGAAACTTTGGGAA
GCTATGTTATCTGAGCATAAAAATAATATAAATAATTGTAAAAATATTCCCCAAGAAGAA
TTACAAATTACTCAATGGATAAAAGAATGGCATGGAGAATTTTTGCTTGAAAGAGATAAT
AGATCAAAATTGCCAAAAAGTAAATGTAAAAATAATACATTATATGAAGCATGTGAGAA
GGAATGTATTGATCCATGTATGAAATATAGAGATTGGATTATTAGAAGTAAATTTGAATG
GCATACGTTATCGAAAGAATATGAAACTCAAAAAGTTCCAAAGGAAAATGCGGAAAATT
ATTTAATCAAAATTTCAGAAAACAAGAATGATGCTAAAGTAAGTTTATTATTGAATAATT
GTGATGCTGAATATTCAAAATATTGTGATTGTAAACATACTACTACTCTCGTTAAAAGCGT
TTTAAATGGTAACGACAATACAATTAAGGAAAAGCGTGAACATATTGATTTAGATGATTT
TTCTAAATTTGGATGTGATAAAAATTCCGTTGATACAAACACAAAGGTGTGGGAATGTAA
AAAACCTTATAAATTATCCACTAAAGATGTATGTGTACCTCCGAGGAGGCAAGAATTATG
TCTTGGAAACATTGATAGAATATACGATAAAAACCTATTAATGATAAAAGAGCATATTCT
TGCTATTGCAATATATGAATCAAGAATATTGAAACGAAAATATAAGAATAAAGATGATAA
AGAAGTTTGTAAAATCATAAATAAAACTTTCGCTGATATAAGAGATATTATAGGAGGTAC
TGATTATTGGAATGATTTGAGCAATAGAAAATTAGTAGGAAAAATTAACACAAATTCAAA
TTATGTTCACAGGAATAAAGAAAATGATAAGCTTTTTCGTGATGCGTGGTGGAAAGTTAT
TAAAAAAGATGTATGGAATGTGATATCATGGGTATTCAAGGATAAAACTGTTTGTAAAGA
AGATGATATTGAAAATATACCACAATTCTTCAGATGGTTTAGTGAATGGGGTGATGATTA
TTGCCAGGATAAAACAAAAATG
>Madagascar Mad56_EBA-175KJ419511.1

TATGTATGTATTCCTGATCGTAGAATCCAATTATGCATTGTTAATCTTAGCATTATTAAAA
CATATACAAAAGAGACCATGAAGGATCATTTCATTGAAGCCTCTAAAAAAGAATCTCAA
CTTTTGCTTAAAAAAAAATGATAACAAATATAATTCTAAATTTTGTAATGATTTGAAGAATA
GTTTTTTAGATTATGGACATCTTGCTATGGGAAATGATATGGATTTTGGAGGTTATTCAAC
TAAGGCAGAAAACAAAATTCAAGAAGTTTTTAAAGGGGCTCATGGGGAAATAAGTGAAC
ATAAAATTAAAAATTTTAGAAAAGAATGGTGGAATGAATTTAGAGAGAAACTTTGGGAA
GCTATGTTATCTGAGCATAAAAATAATATAAATAATTGTAAAAATATTCCCCAAGAAGAA
TTACAAATTACTCAATGGATAAAAGAATGGCATGGAGAATTTTTGCTTGAAAGAGATAAT
AGATCAAAATTGCCAAAAAGTAAATGTAAAAATAATACATTATATGAAGCATGTGAGAA
GGAATGTATTGATCCATGTATGAAATATAGAGATTGGATTATTAGAAGTAAATTTGAATG
GCATACGTTATCGAAAGAATATGAAACTCAAAAAGTTCCAAAGGAAAATGCGGAAAATT
ATTTAATCAAAATTTCAGAAAACAAGAATGATGCTAAAGTAAGTTTATTATTGAATAATT
GTGATGCTGAATATTCAAAATATTGTGATTGTAAACATACTACTACTCTCGTTAAAAGCGT
TTTAAATGGTAACGACAATACAATTAAGGAAAAGCGTGAACATATTGATTTAGATGATTT
TTCTAAATTTGGATGTGATAAAAATTCCGTTGATACAAACACAAAGGTGTGGGAATGTAA
AAAACCTTATAAATTATCCACTAAAGATGTATGTGTACCTCCGAGGAGGCAAGAATTATG
TCTTGGAAACATTGATAGAATATACGATAAAAACCTATTAATGATAAAAGAGCATATTCT
TGCTATTGCAATATATGAATCAAGAATATTGAAACGAAAATATAAGAATAAAGATGATAA
AGAAGTTTGTAAAATCATAAATAAAACTTTCGCTGATATAAGAGATATTATAGGAGGTAC
TGATTATTGGAATGATTTGAGCAATAGAAAATTAGTAGGAAAAATTAACACAAATTCAAA
TTATGTTCACAGGAATAAACAAAATGATAAGCTTTTTCGTGATGAGTGGTGGAAAGTTAT
TAAAAAAGATGTATGGAATGTGATATCATGGGTATTCAAGGATAAAACTGTTTGTAAAGA
AGATGATATTGAAAATATACCACAATTCTTCAGATGGTTTAGTGAATGGGGTGATGATTA
TTGCCAGGATAAAACAAAAATG
>Colombia Col1_EBA-175KJ419512.1
TATGTATGTATTCCTGATCGTAGAATCCAATTATGCATTGTTAATCTTAGCATTATTAAAA
CATATACAAAAGAGACCATGAAGGATCATTTCATTGAAGCCTCTAAAAAAGAATCTCAA
CTTTTGCTTAAAAAAAAATGATAACAAATATAATTCTAAATTTTGTAATGATTTGAAGAATA
GTTTTTTAGATTATGGACATCTTGCTATGGGAAATGATATGGATTTTGGAGGTTATTCAAC
TAAGGCAGAAAACAAAATTCAAGAAGTTTTTAAAGGGGCTCATGGGGAAATAAGTGAAC
ATGAAATTAAAAATTTTAGAAAAAAATGGTGGAATGAATTTAGAGAGAAACTTTGGGAA
GCTATGTTATCTGAGCATAAAAATAATATAAATAATTGTAAAAATATTCCCCAAGAAGAA
TTACAAATTACTCAATGGATAAAAGAATGGCATGGAGAATTTTTGCTTGAAAGAGATAAT
AGATCAAAATTGCCAAAAAGTAAATGTAAAAATAATACATTATATGAAGCATGTGAGAA
GGAATGTATTGATCCATGTATGAAATATAGAGATTGGATTATTAGAAGTAAATTTGAATG
GCATACGTTATCGAAAGAATATGAAACTCAAAAAGTTTCAAAGGAAAATGCGGAAAATT
ATTTAATCAAAATTTCAGAAAACAAGAATGATGCTAAAGTAAGTTTATTATTGAATAATT
GTGATGCTGAATATTCAAAATATTGTGATTGTAAACATACTACTACTCTCGTTAAAAGCGT
TTTAAATGGTAACGACAATACAATTAAGGAAAAGCGTGAACATATTGATTTAGATGATTT
TTCTAAATTTGGATGTGATAAAAATTCCGTTGATACAAACACAAAGGTGTGGGAATGTAA
AAAACCTTATATATTATCCACTAAAGATGTATGTGTACCTCCGAGGAGGCAAGAATTATG
TCTTGGAAACATTGATAGAATATACGATAAAAACCTATTAATGATAAAAGAGCATATTCT
TGCTATTGCAATATATGAATCAAGAATATTGAAACGAAAATATAAGAATAAAGATGATAA
AGAAGTTTGTAAAATCATAAATAAAACTTTCGCTGATATAAGAGATATTATAGGAGGTAC

TGATTATTGGAATGATTTGAGCAATAGAAAATTAGTAGGAAAAATTAACACAAATTCAAA
ATATGTTCACAGGAATAAAAAAAATGATAAGCTTTTTCGTGATGAGTGGTGGAAAGTTAT
TAAAAAAGATGTATGGAATGTGATATCATGGGTATTCAAGGATAAAACTGTTTGTAAAGA
AGATGATATTGAAAATATACCACAATTCTTCAGATGGTTTAGTGAATGGGGTGATGATTA
TTGCCAGGATAAAACAAAAATG
>Colombia Col2_EBA-175KJ419513.1
TATGTATGTATTCCTGATCGTAGAATCCAATTATGCATTGTTAATCTTAGCATTATTAAAA
CATATACAAAAGAGACCATGAAGGATCATTTCATTGAAGCCTCTAAAAAAGAATCTCAA
CTTTTGCTTAAAAAAAATGATAACAAATATAATTCTAAATTTTGTAATGATTTGAAGAATA
GTTTTTTAGATTATGGACATCTTGCTATGGGAAATGATATGGATTTTGGAGGTTATTCAAC
TAAGGCAGAAAACAAAATTCAAGAAGTTTTTAAAGGGGCTCATGGGGAAATAAGTGAAC
ATGAAATTAAAAATTTTAGAAAAAAATGGTGGAATGAATTTAGAGAGAAACTTTGGGAA
GCTATGTTATCTGAGCATAAAAATAATATAAATAATTGTAAAAATATTCCCCAAGAAGAA
TTACAAATTACTCAATGGATAAAAGAATGGCATGGAGAATTTTTGCTTGAAAGAGATAAT
AGATCAAAATTGCCAAAAAGTAAATGTAAAAATAATACATTATATGAAGCATGTGAGAA
GGAATGTATTGATCCATGTATGAAATATAGAGATTGGATTATTAGAAGTAAATTTGAATG
GCATACGTTATCGAAAGAATATGAAACTCAAAAAGTTTCAAAGGAAAATGCGGAAAATT
ATTTAATCAAAATTTCAGAAAACAAGAATGATGCTAAAGTAAGTTTATTATTGAATAATT
GTGATGCTGAATATTCAAAATATTGTGATTGTAAACATACTACTACTCTCGTTAAAAGCGT
TTTAAATGGTAACGACAATACAATTAAGGAAAAGCGTGAACATATTGATTTAGATGATTT
TTCTAAATTTGGATGTGATAAAAATTCCGTTGATACAAACACAAAGGTGTGGGAATGTAA
AAAACCTTATATATTATCCACTAAAGATGTATGTGTACCTCCGAGGAGGCAAGAATTATG
TCTTGGAAACATTGATAGAATATACGATAAAAACCTATTAATGATAAAAGAGCATATTCT
TGCTATTGCAATATATGAATCAAGAATATTGAAACGAAAATATAAGAATAAAGATGATAA
AGAAGTTTGTAAAATCATAAATAAAACTTTCGCTGATATAAGAGATATTATAGGAGGTAC
TGATTATTGGAATGATTTGAGCAATAGAAAATTAGTAGGAAAAATTAACACAAATTCAAA
ATATGTTCACAGGAATAAAAAAAATGATAAGCTTTTTCGTGATGAGTGGTGGAAAGTTAT
TAAAAAAGATGTATGGAATGTGATATCATGGGTATTCAAGGATAAAACTGTTTGTAAAGA
AGATGATATTGAAAATATACCACAATTCTTCAGATGGTTTAGTGAATGGGGTGATGATTA
TTGCCAGGATAAAACAAAAATG
>Colombia Col3_EBA-175KJ419514.1
TATGTATGTATTCCTGATCGTAGAATCCAATTATGCATTGTTAATCTTAGCATTATTAAAA
CATATACAAAAGAGACCATGAAGGATCATTTCATTGAAGCCTCTAAAAAAGAATCTCAA
CTTTTGCTTAAAAAAAATGATAACAAATATAATTCTAAATTTTGTAATGATTTGAAGAATA
GTTTTTTAGATTATGGACATCTTGCTATGGGAAATGATATGGATTTTGGAGGTTATTCAAC
TAAGGCAGAAAACAAAATTCAAGAAGTTTTTAAAGGGGCTCATGGGGAAATAAGTGAAC
ATGAAATTAAAAATTTTAGAAAAAAATGGTGGAATGAATTTAGAGAGAAACTTTGGGAA
GCTATGTTATCTGAGCATAAAAATAATATAAATAATTGTAAAAATATTCCCCAAGAAGAA
TTACAAATTACTCAATGGATAAAAGAATGGCATGGAGAATTTTTGCTTGAAAGAGATAAT
AGATCAAAATTGCCAAAAAGTAAATGTAAAAATAATACATTATATGAAGCATGTGAGAA
GGAATGTATTGATCCATGTATGAAATATAGAGATTGGATTATTAGAAGTAAATTTGAATG
GCATACGTTATCGAAAGAATATGAAACTCAAAAAGTTTCAAAGGAAAATGCGGAAAATT
ATTTAATCAAAATTTCAGAAAACAAGAATGATGCTAAAGTAAGTTTATTATTGAATAATT
GTGATGCTGAATATTCAAAATATTGTGATTGTAAACATACTACTACTCTCGTTAAAAGCGT

TTTAAATGGTAACGACAATACAATTAAGGAAAAGCGTGAACATATTGATTTAGATGATTT
TTCTAAATTTGGATGTGATAAAAATTCCGTTGATACAAACACAAAGGTGTGGGAATGTAA
AAAACCTTATATATTATCCACTAAAGATGTATGTGTACCTCCGAGGAGGCAAGAATTATG
TCTTGGAAACATTGATAGAATATACGATAAAAACCTATTAATGATAAAAGAGCATATTCT
TGCTATTGCAATATATGAATCAAGAATATTGAAACGAAAATATAAGAATAAAGATGATAA
AGAAGTTTGTAAAATCATAAATAAAACTTTCGCTGATATAAGAGATATTATAGGAGGTAC
TGATTATTGGAATGATTTGAGCAATAGAAAATTAGTAGGAAAAATTAACACAAATTCAAA
ATATGTTCACAGGAATAAAAAAAATGATAAGCTTTTTCGTGATGAGTGGTGGAAAGTTAT
TAAAAAAGATGTATGGAATGTGATATCATGGGTATTCAAGGATAAAACTGTTTGTAAAGA
AGATGATATTGAAAATATACCACAATTCTTCAGATGGTTTAGTGAATGGGGTGATGATTA
TTGCCAGGATAAAACAAAAATG
>Colombia Col4_EBA-175KJ419515.1
TATGTATGTATTCCTGATCGTAGAATCCAATTATGCATTGTTAATCTTAGCATTATTAAAA
CATATACAAAAGAGACCATGAAGGATCATTTCATTGAAGCCTCTAAAAAAGAATCTCAA
CTTTTGCTTAAAAAAAAATGATAACAAATATAATTCTAAATTTTGTAATGATTTGAAGAATA
GTTTTTTAGATTATGGACATCTTGCTATGGGAAATGATATGGATTTTGGAGGTTATTCAAC
TAAGGCAGAAAACAAAATTCAAGAAGTTTTTAAAGGGGCTCATGGGGAAATAAGTGAAC
ATAAAATTAAAAATTTTAGAAAAGAATGGTGGAATGAATTTAGAGAGAAACTTTGGGAA
GCTATGTTATCTGAGCATAAAAATAATATAAATAATTGTAAAAATATTCCCCAAGAAGAA
TTACAAATTACTCAATGGATAAAAGAATGGCATGGAGAATTTTTGCTTGAAAGAGATAAT
AGATCAAAATTGCCAAAAAGTAAATGTAAAAATAATACATTATATGAAGCATGTGAGAA
GGAATGTATTGATCCATGTATGAAATATAGAGATTGGATTATTAGAAGTAAATTTGAATG
GCATACGTTATCGAAAGAATATGAAACTCAAAAAGTTCCAAAGGAAAATGCGGAAAATT
ATTTAATCAAAATTTCAGAAAACAAGAATGATGCTAAAGTAAGTTTATTATTGAATAATT
GTGATGCTGAATATTCAAAATATTGTGATTGTAAACATACTACTACTCTCGTTAAAAGCGT
TTTAAATGGTAACGACAATACAATTAAGGAAAAGCGTGAACATATTGATTTAGATGATTT
TTCTAAATTTGGATGTGATAAAAATTCCGTTGATACAAACACAAAGGTGTGGGAATGTAA
AAAACCTTATAAATTATCCACTAAAGATGTATGTGTACCTCCGAGGAGGCAAGAATTATG
TCTTGGAAACATTGATAGAATATACGATAAAAACCTATTAATGATAAAAGAGCATATTCT
TGCTATTGCAATATATGAATCAAGAATATTGAAACGAAAATATAAGAATAAAGATGATAA
AGAAGTTTGTAAAATCATAAATAAAACTTTCGCTGATATAAGAGATATTATAGGAGGTAC
TGATTATTGGAATGATTTGAGCAATAGAAAATTAGTAGGAAAAATTAACACAAATTCAAA
TTATGTTCACAGGAATAAACAAAATGATAAGCTTTTTCGTGATGAGTGGTGGAAAGTTAT
TAAAAAAGATGTATGGAATGTGATATCATGGGTATTCAAGGATAAAACTGTTTGTAAAGA
AGATGATATTGAAAATATACCACAATTCTTCAGATGGTTTAGTGAATGGGGTGATGATTA
TTGCCAGGATAAAACAAAAATG
>Colombia Col5_EBA-175KJ419516.1
TATGTATGTATTCCTGATCGTAGAATCCAATTATGCATTGTTAATCTTAGCATTATTAAAA
CATATACAAAAGAGACCATGAAGGATCATTTCATTGAAGCCTCTAAAAAAGAATCTCAA
CTTTTGCTTAAAAAAAAATGATAACAAATATAATTCTAAATTTTGTAATGATTTGAAGAATA
GTTTTTTAGATTATGGACATCTTGCTATGGGAAATGATATGGATTTTGGAGGTTATTCAAC
TAAGGCAGAAAACAAAATTCAAGAAGTTTTTAAAGGGGCTCATGGGGAAATAAGTGAAC
ATAAAATTAAAAATTTTAGAAAAGAATGGTGGAATGAATTTAGAGAGAAACTTTGGGAA
GCTATGTTATCTGAGCATAAAAATAATATAAATAATTGTAAAAATATTCCCCAAGAAGAA

TTACAAATTACTCAATGGATAAAAGAATGGCATGGAGAATTTTTGCTTGAAAGAGATAAT
AGATCAAAATTGCCAAAAAGTAAATGTAAAAATAATACATTATATGAAGCATGTGAGAA
GGAATGTATTGATCCATGTATGAAATATAGAGATTGGATTATTAGAAGTAAATTTGAATG
GCATACGTTATCGAAAGAATATGAAACTCAAAAAGTTCCAAAGGAAAATGCGGAAAATT
ATTTAATCAAAATTTCAGAAAACAAGAATGATGCTAAAGTAAGTTTATTATTGAATAATT
GTGATGCTGAATATTCAAAATATTGTGATTGTAAACATACTACTACTCTCGTTAAAAGCGT
TTTAAATGGTAACGACAATACAATTAAGGAAAAGCGTGAACATATTGATTTAGATGATTT
TTCTAAATTTGGATGTGATAAAAATTCCGTTGATACAAACACAAAGGTGTGGGAATGTAA
AAAACCTTATAAATTATCCACTAAAGATGTATGTGTACCTCCGAGGAGGCAAGAATTATG
TCTTGGAAACATTGATAGAATATACGATAAAAACCTATTAATGATAAAAGAGCATATTCT
TGCTATTGCAATATATGAATCAAGAATATTGAAACGAAAATATAAGAATAAAGATGATAA
AGAAGTTTGTAAAATCATAAATAAAACTTTCGCTGATATAAGAGATATTATAGGAGGTAC
TGATTATTGGAATGATTTGAGCAATAGAAAATTAGTAGGAAAAATTAACACAAATTCAAA
TTATGTTCACAGGAATAAACAAAATGATAAGCTTTTTCGTGATGAGTGGTGGAAAGTTAT
TAAAAAAGATGTATGGAATGTGATATCATGGGTATTCAAGGATAAAACTGTTTGTAAAGA
AGATGATATTGAAAATATACCACAATTCTTCAGATGGTTTAGTGAATGGGGTGATGATTA
TTGCCAGGATAAAACAAAAATG
>Colombia Col7_EBA-175KJ419517.1
TATGTATGTATTCCTGATCGTAGAATCCAATTATGCATTGTTAATCTTAGCATTATTAAAA
CATATACAAAAGAGACCATGAAGGATCATTTCATTGAAGCCTCTAAAAAAGAATCTCAA
CTTTTGCTTAAAAAAAAATGATAACAAATATAATTCTAAATTTTGTAATGATTTGAAGAATA
GTTTTTTAGATTATGGACATCTTGCTATGGGAAATGATATGGATTTTGGAGGTTATTCAAC
TAAGGCAGAAAACAAAATTCAAGAAGTTTTTAAAGGGGCTCATGGGAAAATAAGTGAAC
ATGAAATTAAAAATTTTAGAAAAAAAATGGTGGAATGAATTTAGAGAGAAACTTTGGGAA
GCTATGTTATCTGAGCATAAAAATAATATAAATAATTGTAAAAATATTCCCCAAGAAGAA
TTACAAATTACTCAATGGATAAAAGAATGGCATGGAGAATTTTTGCTTGAAAGATATAAT
AGATCAAAATTGCCAAAAAGTAAATGTAAAAATAATACATTATATGAAGCATGTGAGAA
GGAATGTATTGATCCATGTATGAAATATAGAGATTGGATTATTAGAAGTAAATTTGAATG
GCATACGTTATCGAAAGAATATGAAACTCAAAATGTTTCAAAGGAAAATGCGGAAAATT
ATTTAATCAAAATTTCAAAAAACAAGAATGATGCTAAAGTAAGTTTATTATTGAATAATTG
TGATGCTGAATATTCAAAATATTGTGATTGTAAACATACTACTACTCTCGTTAAAAGCGTT
TTAAATGGTAACGACAATACAATTAAGGAAAAGCGTGAACATATTGATTTAGATGATTTT
TCTAAATTTGGATGTGATAAAAATTCCGTTGATACAAACACAAAGGTGTGGGAATGTAAA
AAACCTTATATATTATCCACTAAAGATGTATGTGTACCTCCGAGGAGGCAAGAATTATGT
CTTGGAAACATTGATAGAATATACGATAAAAACCTATTAATGATAAAAGAGCATATTCTT
GCTATTGCAATATATGAATCAAGAATATTGAAACGAAAATATAAGAATAAAGATGATAA
AGAAGTTTGTAAAATCATAAATAAAACTTTCGCTGATATAAGAGATATTATAGGAGGTAC
TGATTATTGGAATGATTTGAGCAATAGAAAATTAGTAGGAAAAATTAACACAAATTCAAA
ATATGCTCACAGGAATAAAAAAAATGATAAGCTTTTTCGTGATGAGTGGTGGAAAGTTAT
TAAAAAAGATGTATGGAATGTGATATCATGGGTATTCAAGGATAAAACTGTTTGTAAAGA
AGATGATATTGAAAATATACCACAATTCTTCAGATGGTTTAGTGAATGGGGTGATGATTA
TTGCCAGGATAAAACAAAAATG
>Colombia Col8_EBA-175KJ419518.1
TATGTATGTATTCCTGATCGTAGAATCCAATTATGCATTGTTAATCTTAGCATTATTAAAA

CATATACAAAAGAGACCATGAAGGATCATTTCATTGAAGCCTCTAAAAAAGAATCTCAA
CTTTTGCTTAAAAAAAAATGATAACAAATATAATTCTAAATTTTGTAATGATTTGAAGAATA
GTTTTTTAGATTATGGACATCTTGCTATGGGAAATGATATGGATTTTGGAGGTTATTCAAC
TAAGGCAGAAAACAAAATTCAAGAAGTTTTTAAAGGGGCTCATGGGGAAATAAGTGAAC
ATAAAATTAAAAATTTTAGAAAAGAATGGTGGAATGAATTTAGAGAGAAACTTTGGGAA
GCTATGTTATCTGAGCATAAAAATAATATAAATAATTGTAAAAATATTCCCCAAGAAGAA
TTACAAATTACTCAATGGATAAAAGAATGGCATGGAGAATTTTTGCTTGAAAGAGATAAT
AGATCAAAATTGCCAAAAAGTAAATGTAAAAATAATACATTATATGAAGCATGTGAGAA
GGAATGTATTGATCCATGTATGAAATATAGAGATTGGATTATTAGAAGTAAATTTGAATG
GCATACGTTATCGAAAGAATATGAAACTCAAAAAGTTCCAAAGGAAAATGCGGAAAATT
ATTTAATCAAAATTTCAGAAAACAAGAATGATGCTAAAGTAAGTTTATTATTGAATAATT
GTGATGCTGAATATTCAAAATATTGTGATTGTAAACATACTACTACTCTCGTTAAAAGCGT
TTTAAATGGTAACGACAATACAATTAAGGAAAAGCGTGAACATATTGATTTAGATGATTT
TTCTAAATTTGGATGTGATAAAAATTCCGTTGATACAAACACAAAGGTGTGGGAATGTAA
AAAACCTTATAAATTATCCACTAAAGATGTATGTGTACCTCCGAGGAGGCAAGAATTATG
TCTTGGAAACATTGATAGAATATACGATAAAAACCTATTAATGATAAAAGAGCATATTCT
TGCTATTGCAATATATGAATCAAGAATATTGAAACGAAAATATAAGAATAAAGATGATAA
AGAAGTTTGTAAAATCATAAATAAAACTTTCGCTGATATAAGAGATATTATAGGAGGTAC
TGATTATTGGAATGATTTGAGCAATAGAAAATTAGTAGGAAAAATTAACACAAATTCAAA
TTATGTTCACAGGAATAAACAAAATGATAAGCTTTTTCGTGATGAGTGGTGGAAAGTTAT
TAAAAAAGATGTATGGAATGTGATATCATGGGTATTCAAGGATAAAACTGTTTGTAAAGA
AGATGATATTGAAAATATACCACAATTCTTCAGATGGTTTAGTGAATGGGGTGATGATTA
TTGCCAGGATAAAACAAAAATG
>Colombia Col9_EBA-175KJ419519.1
TATGTATGTATTCCTGATCGTAGAATCCAATTATGCATTGTTAATCTTAGCATTATTAAAA
CATATACAAAAGAGACCATGAAGGATCATTTCATTGAAGCCTCTAAAAAAGAATCTCAA
CTTTTGCTTAAAAAAAAATGATAACAAATATAATTCTAAATTTTGTAATGATTTGAAGAATA
GTTTTTTAGATTATGGACATCTTGCTATGGGAAATGATATGGATTTTGGAGGTTATTCAAC
TAAGGCAGAAAACAAAATTCAAGAAGTTTTTAAAGGGGCTCATGGGGAAATAAGTGAAC
ATAAAATTAAAAATTTTAGAAAAGAATGGTGGAATGAATTTAGAGAGAAACTTTGGGAA
GCTATGTTATCTGAGCATAAAAATAATATAAATAATTGTAAAAATATTCCCCAAGAAGAA
TTACAAATTACTCAATGGATAAAAGAATGGCATGGAGAATTTTTGCTTGAAAGAGATAAT
AGATCAAAATTGCCAAAAAGTAAATGTAAAAATAATACATTATATGAAGCATGTGAGAA
GGAATGTATTGATCCATGTATGAAATATAGAGATTGGATTATTAGAAGTAAATTTGAATG
GCATACGTTATCGAAAGAATATGAAACTCAAAAAGTTCCAAAGGAAAATGCGGAAAATT
ATTTAATCAAAATTTCAGAAAACAAGAATGATGCTAAAGTAAGTTTATTATTGAATAATT
GTGATGCTGAATATTCAAAATATTGTGATTGTAAACATACTACTACTCTCGTTAAAAGCGT
TTTAAATGGTAACGACAATACAATTAAGGAAAAGCGTGAACATATTGATTTAGATGATTT
TTCTAAATTTGGATGTGATAAAAATTCCGTTGATACAAACACAAAGGTGTGGGAATGTAA
AAAACCTTATAAATTATCCACTAAAGATGTATGTGTACCTCCGAGGAGGCAAGAATTATG
TCTTGGAAACATTGATAGAATATACGATAAAAACCTATTAATGATAAAAGAGCATATTCT
TGCTATTGCAATATATGAATCAAGAATATTGAAACGAAAATATAAGAATAAAGATGATAA
AGAAGTTTGTAAAATCATAAATAAAACTTTCGCTGATATAAGAGATATTATAGGAGGTAC
TGATTATTGGAATGATTTGAGCAATAGAAAATTAGTAGGAAAAATTAACACAAATTCAAA

TTATGTTCACAGGAATAAACAAAATGATAAGCTTTTTCGTGATGAGTGGTGGAAAGTTAT
TAAAAAAGATGTATGGAATGTGATATCATGGGTATTCAAGGATAAAACTGTTTGTAAAGA
AGATGATATTGAAAATATACCACAATTCTTCAGATGGTTTAGTGAATGGGGTGATGATTA
TTGCCAGGATAAAACAAAAATG
>Colombia Col10_EBA-175KJ419520.1
TATGTATGTATTCCTGATCGTAGAATCCAATTATGCATTGTTAATCTTAGCATTATTAAAA
CATATACAAAAGAGACCATGAAGGATCATTTCATTGAAGCCTCTAAAAAAGAATCTCAA
CTTTTGCTTAAAAAAAAATGATAACAAATATAATTCTAAATTTTGTAATGATTTGAAGAATA
GTTTTTTAGATTATGGACATCTTGCTATGGGAAATGATATGGATTTTGGAGGTTATTCAAC
TAAGGCAGAAAACAAAATTCAAGAAGTTTTTAAAGGGGCTCATGGGGAAATAAGTGAAC
ATAAAATTAAAAATTTTAGAAAAGAATGGTGGAATGAATTTAGAGAGAAACTTTGGGAA
GCTATGTTATCTGAGCATAAAAATAATATAAATAATTGTAAAAATATTCCCCAAGAAGAA
TTACAAATTACTCAATGGATAAAAGAATGGCATGGAGAATTTTTGCTTGAAAGAGATAAT
AGATCAAAATTGCCAAAAAGTAAATGTAAAAATAATACATTATATGAAGCATGTGAGAA
GGAATGTATTGATCCATGTATGAAATATAGAGATTGGATTATTAGAAGTAAATTTGAATG
GCATACGTTATCGAAAGAATATGAAACTCAAAAAGTTCCAAAGGAAAATGCGGAAAATT
ATTTAATCAAAATTTCAGAAAACAAGAATGATGCTAAAGTAAGTTTATTATTGAATAATT
GTGATGCTGAATATTCAAAATATTGTGATTGTAAACATACTACTACTCTCGTTAAAAGCGT
TTTAAATGGTAACGACAATACAATTAAGGAAAAGCGTGAACATATTGATTTAGATGATTT
TTCTAAATTTGGATGTGATAAAAAATTCCGTTGATACAAACACAAAGGTGTGGGAATGTAA
AAAACCTTATAAATTATCCACTAAAGATGTATGTGTACCTCCGAGGAGGCAAGAATTATG
TCTTGGAAACATTGATAGAATATACGATAAAAACCTATTAATGATAAAAGAGCATATTCT
TGCTATTGCAATATATGAATCAAGAATATTGAAACGAAAATATAAGAATAAAGATGATAA
AGAAGTTTGTAAAATCATAAATAAAACTTTCGCTGATATAAGAGATATTATAGGAGGTAC
TGATTATTGGAATGATTTGAGCAATAGAAAATTAGTAGGAAAAATTAACACAAATTCAAA
TTATGTTCACAGGAATAAACAAAATGATAAGCTTTTTCGTGATGAGTGGTGGAAAGTTAT
TAAAAAAGATGTATGGAATGTGATATCATGGGTATTCAAGGATAAAACTGTTTGTAAAGA
AGATGATATTGAAAATATACCACAATTCTTCAGATGGTTTAGTGAATGGGGTGATGATTA
TTGCCAGGATAAAACAAAAATG
>Colombia Col11_EBA-175KJ419521.1
TATGTATGTATTCCTGATCGTAGAATCCAATTATGCATTGTTAATCTTAGCATTATTAAAA
CATATACAAAAGAGACCATGAAGGATCATTTCATTGAAGCCTCTAAAAAAGAATCTCAA
CTTTTGCTTAAAAAAAAATGATAACAAATATAATTCTAAATTTTGTAATGATTTGAAGAATA
GTTTTTTAGATTATGGACATCTTGCTATGGGAAATGATATGGATTTTGGAGGTTATTCAAC
TAAGGCAGAAAACAAAATTCAAGAAGTTTTTAAAGGGGCTCATGGGGAAATAAGTGAAC
ATAAAATTAAAAATTTTAGAAAAGAATGGTGGAATGAATTTAGAGAGAAACTTTGGGAA
GCTATGTTATCTGAGCATAAAAATAATATAAATAATTGTAAAAATATTCCCCAAGAAGAA
TTACAAATTACTCAATGGATAAAAGAATGGCATGGAGAATTTTTGCTTGAAAGAGATAAT
AGATCAAAATTGCCAAAAAGTAAATGTAAAAATAATACATTATATGAAGCATGTGAGAA
GGAATGTATTGATCCATGTATGAAATATAGAGATTGGATTATTAGAAGTAAATTTGAATG
GCATACGTTATCGAAAGAATATGAAACTCAAAAAGTTCCAAAGGAAAATGCGGAAAATT
ATTTAATCAAAATTTCAGAAAACAAGAATGATGCTAAAGTAAGTTTATTATTGAATAATT
GTGATGCTGAATATTCAAAATATTGTGATTGTAAACATACTACTACTCTCGTTAAAAGCGT
TTTAAATGGTAACGACAATACAATTAAGGAAAAGCGTGAACATATTGATTTAGATGATTT

TTCTAAATTTGGATGTGATAAAAATTCCGTTGATACAAACACAAAGGTGTGGGAATGTAA
AAAACCTTATAAATTATCCACTAAAGATGTATGTGTACCTCCGAGGAGGCAAGAATTATG
TCTTGGAAACATTGATAGAATATACGATAAAAACCTATTAATGATAAAAGAGCATATTCT
TGCTATTGCAATATATGAATCAAGAATATTGAAACGAAAATATAAGAATAAAGATGATAA
AGAAGTTTGTAAAATCATAAATAAAACTTTCGCTGATATAAGAGATATTATAGGAGGTAC
TGATTATTGGAATGATTTGAGCAATAGAAAATTAGTAGGAAAAATTAACACAAATTCAAA
TTATGTTCACAGGAATAAACAAAATGATAAGCTTTTTCGTGATGAGTGGTGGAAAGTTAT
TAAAAAAGATGTATGGAATGTGATATCATGGGTATTCAAGGATAAAACTGTTTGTAAAGA
AGATGATATTGAAAATATACCACAATTCTTCAGATGGTTTAGTGAATGGGGTGATGATTA
TTGCCAGGATAAAACAAAAATG
>Colombia Col13_EBA-175KJ419522.1
TATGTATGTATTCCTGATCGTAGAATCCAATTATGCATTGTTAATCTTAGCATTATTAAAA
CATATACAAAAGAGACCATGAAGGATCATTTCATTGAAGCCTCTAAAAAAGAATCTCAA
CTTTTGCTTAAAAAAAAATGATAACAAATATAATTCTAAATTTTGTAATGATTTGAAGAATA
GTTTTTTAGATTATGGACATCTTGCTATGGGAAATGATATGGATTTTGGAGGTTATTCAAC
TAAGGCAGAAAACAAAATTCAAGAAGTTTTTAAAGGGGCTCATGGGGAAATAAGTGAAC
ATAAAATTAAAAAATTTTAGAAAAGAATGGTGGAATGAATTTAGAGAGAAACTTTGGGAA
GCTATGTTATCTGAGCATAAAAATAATATAAATAATTGTAAAAATATTCCCCAAGAAGAA
TTACAAATTACTCAATGGATAAAAGAATGGCATGGAGAATTTTTGCTTGAAAGAGATAAT
AGATCAAAATTGCCAAAAAGTAAATGTAAAAATAATACATTATATGAAGCATGTGAGAA
GGAATGTATTGATCCATGTATGAAATATAGAGATTGGATTATTAGAAGTAAATTTGAATG
GCATACGTTATCGAAAGAATATGAAACTCAAAAAGTTCCAAAGGAAAATGCGGAAAATT
ATTTAATCAAAATTTCAGAAAACAAGAATGATGCTAAAGTAAGTTTATTATTGAATAATT
GTGATGCTGAATATTCAAAATATTGTGATTGTAAACATACTACTACTCTCGTTAAAAGCGT
TTTAAATGGTAACGACAATACAATTAAGGAAAAGCGTGAACATATTGATTTAGATGATTT
TTCTAAATTTGGATGTGATAAAAATTCCGTTGATACAAACACAAAGGTGTGGGAATGTAA
AAAACCTTATAAATTATCCACTAAAGATGTATGTGTACCTCCGAGGAGGCAAGAATTATG
TCTTGGAAACATTGATAGAATATACGATAAAAACCTATTAATGATAAAAGAGCATATTCT
TGCTATTGCAATATATGAATCAAGAATATTGAAACGAAAATATAAGAATAAAGATGATAA
AGAAGTTTGTAAAATCATAAATAAAACTTTCGCTGATATAAGAGATATTATAGGAGGTAC
TGATTATTGGAATGATTTGAGCAATAGAAAATTAGTAGGAAAAATTAACACAAATTCAAA
TTATGTTCACAGGAATAAACAAAATGATAAGCTTTTTCGTGATGAGTGGTGGAAAGTTAT
TAAAAAAGATGTATGGAATGTGATATCATGGGTATTCAAGGATAAAACTGTTTGTAAAGA
AGATGATATTGAAAATATACCACAATTCTTCAGATGGTTTAGTGAATGGGGTGATGATTA
TTGCCAGGATAAAACAAAAATG
>Colombia Col14_EBA-175KJ419523.1
TATGTATGTATTCCTGATCGTAGAATCCAATTATGCATTGTTAATCTTAGCATTATTAAAA
CATATACAAAAGAGACCATGAAGGATCATTTCATTGAAGCCTCTAAAAAAGAATCTCAA
CTTTTGCTTAAAAAAAAATGATAACAAATATAATTCTAAATTTTGTAATGATTTGAAGAATA
GTTTTTTAGATTATGGACATCTTGCTATGGGAAATGATATGGATTTTGGAGGTTATTCAAC
TAAGGCAGAAAACAAAATTCAAGAAGTTTTTAAAGGGGCTCATGGGGAAATAAGTGAAC
ATAAAATTAAAAAATTTTAGAAAAGAATGGTGGAATGAATTTAGAGAGAAACTTTGGGAA
GCTATGTTATCTGAGCATAAAAATAATATAAATAATTGTAAAAATATTCCCCAAGAAGAA
TTACAAATTACTCAATGGATAAAAGAATGGCATGGAGAATTTTTGCTTGAAAGAGATAAT

AGATCAAAATTGCCAAAAAGTAAATGTAAAAATAATACATTATATGAAGCATGTGAGAA
GGAATGTATTGATCCATGTATGAAATATAGAGATTGGATTATTAGAAGTAAATTTGAATG
GCATACGTTATCGAAAGAATATGAAACTCAAAAAGTTCCAAAGGAAAATGCGGAAAATT
ATTTAATCAAAATTTCAGAAAACAAGAATGATGCTAAAGTAAGTTTATTATTGAATAATT
GTGATGCTGAATATTCAAAATATTGTGATTGTAAACATACTACTACTCTCGTTAAAAGCGT
TTTAAATGGTAACGACAATACAATTAAGGAAAAGCGTGAACATATTGATTTAGATGATTT
TTCTAAATTTGGATGTGATAAAAATTCCGTTGATACAAACACAAAGGTGTGGGAATGTAA
AAAACCTTATAAATTATCCACTAAAGATGTATGTGTACCTCCGAGGAGGCAAGAATTATG
TCTTGGAAACATTGATAGAATATACGATAAAAACCTATTAATGATAAAAGAGCATATTCT
TGCTATTGCAATATATGAATCAAGAATATTGAAACGAAAATATAAGAATAAAGATGATAA
AGAAGTTTGTAAAATCATAAATAAAACTTTCGCTGATATAAGAGATATTATAGGAGGTAC
TGATTATTGGAATGATTTGAGCAATAGAAAATTAGTAGGAAAAATTAACACAAATTCAAA
TTATGTTCACAGGAATAAACAAAATGATAAGCTTTTTCGTGATGAGTGGTGGAAAGTTAT
TAAAAAAGATGTATGGAATGTGATATCATGGGTATTCAAGGATAAAACTGTTTGTAAAGA
AGATGATATTGAAAATATACCACAATTCTTCAGATGGTTTAGTGAATGGGGTGATGATTA
TTGCCAGGATAAAACAAAAATG
>Colombia Col15_EBA-175KJ419524.1
TATGTATGTATTCCTGATCGTAGAATCCAATTATGCATTGTTAATCTTAGCATTATTAAAA
CATATACAAAAGAGACCATGAAGGATCATTTCATTGAAGCCTCTAAAAAAGAATCTCAA
CTTTTGCTTAAAAAAAATGATAACAAATATAATTCTAAATTTTGTAATGATTTGAAGAATA
GTTTTTTAGATTATGGACATCTTGCTATGGGAAATGATATGGATTTTGGAGGTTATTCAAC
TAAGGCAGAAAACAAAATTCAAGAAGTTTTTAAAGGGGCTCATGGGGAAATAAGTGAAC
ATGAAATTAAAAATTTTAGAAAAAAAATGGTGGAATGAATTTAGAGAGAAACTTTGGGAA
GCTATGTTATCTGAGCATAAAAATAATATAAATAATTGTAAAAATATTCCCCAAGAAGAA
TTACAAATTACTCAATGGATAAAAGAATGGCATGGAGAATTTTTGCTTGAAAGAGATAAT
AGATCAAAATTGCCAAAAAGTAAATGTAAAAATAATACATTATATGAAGCATGTGAGAA
GGAATGTATTGATCCATGTATGAAATATAGAGATTGGATTATTAGAAGTAAATTTGAATG
GCATACGTTATCGAAAGAATATGAAACTCAAAAAGTTTCAAAGGAAAATGCGGAAAATT
ATTTAATCAAAATTTCAGAAAACAAGAATGATGCTAAAGTAAGTTTATTATTGAATAATT
GTGATGCTGAATATTCAAAATATTGTGATTGTAAACATACTACTACTCTCGTTAAAAGCGT
TTTAAATGGTAACGACAATACAATTAAGGAAAAGCGTGAACATATTGATTTAGATGATTT
TTCTAAATTTGGATGTGATAAAAATTCCGTTGATACAAACACAAAGGTGTGGGAATGTAA
AAAACCTTATATATTATCCACTAAAGATGTATGTGTACCTCCGAGGAGGCAAGAATTATG
TCTTGGAAACATTGATAGAATATACGATAAAAACCTATTAATGATAAAAGAGCATATTCT
TGCTATTGCAATATATGAATCAAGAATATTGAAACGAAAATATAAGAATAAAGATGATAA
AGAAGTTTGTAAAATCATAAATAAAACTTTCGCTGATATAAGAGATATTATAGGAGGTAC
TGATTATTGGAATGATTTGAGCAATAGAAAATTAGTAGGAAAAATTAACACAAATTCAAA
ATATGTTCACAGGAATAAAAAAAAATGATAAGCTTTTTCGTGATGAGTGGTGGAAAGTTAT
TAAAAAAGATGTATGGAATGTGATATCATGGGTATTCAAGGATAAAACTGTTTGTAAAGA
AGATGATATTGAAAATATACCACAATTCTTCAGATGGTTTAGTGAATGGGGTGATGATTA
TTGCCAGGATAAAACAAAAATG
>Colombia Col17_EBA-175KJ419525.1
TATGTATGTATTCCTGATCGTAGAATCCAATTATGCATTGTTAATCTTAGCATTATTAAAA
CATATACAAAAGAGACCATGAAGGATCATTTCATTGAAGCCTCTAAAAAAGAATCTCAA

CTTTTGCTTAAAAAAAATGATAACAAATATAATTCTAAATTTTGTAATGATTTGAAGAATA
GTTTTTTAGATTATGGACATCTTGCTATGGGAAATGATATGGATTTTGGAGGTTATTCAAC
TAAGGCAGAAAACAAAATTCAAGAAGTTTTTAAAGGGGCTCATGGGAAAATAAGTGAAC
ATGAAATTAAAAATTTTAGAAAAAAATGGTGGAATGAATTTAGAGAGAAACTTTGGGAA
GCTATGTTATCTGAGCATAAAAATAATATAAATAATTGTAAAAATATTCCCCAAGAAGAA
TTACAAATTACTCAATGGATAAAAGAATGGCATGGAGAATTTTTGCTTGAAAGATATAAT
AGATCAAAATTGCCAAAAAGTAAATGTAAAAATAATACATTATATGAAGCATGTGAGAA
GGAATGTATTGATCCATGTATGAAATATAGAGATTGGATTATTAGAAGTAAATTTGAATG
GCATACGTTATCGAAAGAATATGAAACTCAAAATGTTTCAAAGGAAAATGCGGAAAATT
ATTTAATCAAAATTTCAAAAAACAAGAATGATGCTAAAGTAAGTTTATTATTGAATAATTG
TGATGCTGAATATTCAAAATATTGTGATTGTAAACATACTACTACTCTCGTTAAAAGCGTT
TTAAATGGTAACGACAATACAATTAAGGAAAAGCGTGAACATATTGATTTAGATGATTTT
TCTAAATTTGGATGTGATAAAAATTCCGTTGATACAAACACAAAGGTGTGGGAATGTAAA
AAACCTTATATATTATCCACTAAAGATGTATGTGTACCTCCGAGGAGGCAAGAATTATGT
CTTGGAAACATTGATAGAATATACGATAAAAACCTATTAATGATAAAAGAGCATATTCTT
GCTATTGCAATATATGAATCAAGAATATTGAAACGAAAATATAAGAATAAAGATGATAA
AGAAGTTTGTAAAATCATAAATAAAACTTTCGCTGATATAAGAGATATTATAGGAGGTAC
TGATTATTGGAATGATTTGAGCAATAGAAAATTAGTAGGAAAAATTAACACAAATTCAAA
ATATGCTCACAGGAATAAAAAAAATGATAAGCTTTTTCGTGATGAGTGGTGGAAAGTTAT
TAAAAAAGATGTATGGAATGTGATATCATGGGTATTCAAGGATAAAACTGTTTGTAAAGA
AGATGATATTGAAAATATACCACAATTCTTCAGATGGTTTAGTGAATGGGGTGATGATTA
TTGCCAGGATAAAACAAAAATG
>Colombia Col18_EBA-175KJ419526.1
TATGTATGTATTCCTGATCGTAGAATCCAATTATGCATTGTTAATCTTAGCATTATTAAAA
CATATACAAAAGAGACCATGAAGGATCATTTCATTGAAGCCTCTAAAAAAGAATCTCAA
CTTTTGCTTAAAAAAAATGATAACAAATATAATTCTAAATTTTGTAATGATTTGAAGAATA
GTTTTTTAGATTATGGACATCTTGCTATGGGAAATGATATGGATTTTGGAGGTTATTCAAC
TAAGGCAGAAAACAAAATTCAAGAAGTTTTTAAAGGGGCTCATGGGGAAATAAGTGAAC
ATAAAATTAAAAATTTTAGAAAAGAATGGTGGAATGAATTTAGAGAGAAACTTTGGGAA
GCTATGTTATCTGAGCATAAAAATAATATAAATAATTGTAAAAATATTCCCCAAGAAGAA
TTACAAATTACTCAATGGATAAAAGAATGGCATGGAGAATTTTTGCTTGAAAGAGATAAT
AGATCAAAATTGCCAAAAAGTAAATGTAAAAATAATACATTATATGAAGCATGTGAGAA
GGAATGTATTGATCCATGTATGAAATATAGAGATTGGATTATTAGAAGTAAATTTGAATG
GCATACGTTATCGAAAGAATATGAAACTCAAAAAGTTCCAAAGGAAAATGCGGAAAATT
ATTTAATCAAAATTTCAGAAAACAAGAATGATGCTAAAGTAAGTTTATTATTGAATAATT
GTGATGCTGAATATTCAAAATATTGTGATTGTAAACATACTACTACTCTCGTTAAAAGCGT
TTTAAATGGTAACGACAATACAATTAAGGAAAAGCGTGAACATATTGATTTAGATGATTT
TTCTAAATTTGGATGTGATAAAAATTCCGTTGATACAAACACAAAGGTGTGGGAATGTAA
AAAACCTTATAAATTATCCACTAAAGATGTATGTGTACCTCCGAGGAGGCAAGAATTATG
TCTTGGAAACATTGATAGAATATACGATAAAAACCTATTAATGATAAAAGAGCATATTCT
TGCTATTGCAATATATGAATCAAGAATATTGAAACGAAAATATAAGAATAAAGATGATAA
AGAAGTTTGTAAAATCATAAATAAAACTTTCGCTGATATAAGAGATATTATAGGAGGTAC
TGATTATTGGAATGATTTGAGCAATAGAAAATTAGTAGGAAAAATTAACACAAATTCAAA
TTATGTTCACAGGAATAAACAAAATGATAAGCTTTTTCGTGATGAGTGGTGGAAAGTTAT

TAAAAAAGATGTATGGAATGTGATATCATGGGTATTCAAGGATAAAACTGTTTGTAAAGA
AGATGATATTGAAAATATACCACAATTCTTCAGATGGTTTAGTGAATGGGGTGATGATTA
TTGCCAGGATAAAACAAAAATG
>Colombia Col22_EBA-175KJ419527.1
TATGTATGTATTCCTGATCGTAGAATCCAATTATGCATTGTTAATCTTAGCATTATTAAAA
CATATACAAAAGAGACCATGAAGGATCATTTCATTGAAGCCTCTAAAAAAGAATCTCAA
CTTTTGCTTAAAAAAAAATGATAACAAATATAATTCTAAATTTTGTAATGATTTGAAGAATA
GTTTTTTAGATTATGGACATCTTGCTATGGGAAATGATATGGATTTTGGAGGTTATTCAAC
TAAGGCAGAAAACAAAATTCAAGAAGTTTTTAAAGGGGCTCATGGGGAAATAAGTGAAC
ATGAAATTAAAAATTTTAGAAAAAAATGGTGGAATGAATTTAGAGAGAAACTTTGGGAA
GCTATGTTATCTGAGCATAAAAATAATATAAATAATTGTAAAAATATTCCCCAAGAAGAA
TTACAAATTACTCAATGGATAAAAGAATGGCATGGAGAATTTTTGCTTGAAAGAGATAAT
AGATCAAAATTGCCAAAAAGTAAATGTAAAAATAATACATTATATGAAGCATGTGAGAA
GGAATGTATTGATCCATGTATGAAATATAGAGATTGGATTATTAGAAGTAAATTTGAATG
GCATACGTTATCGAAAGAATATGAAACTCAAAAAGTTTCAAAGGAAAATGCGGAAAATT
ATTTAATCAAAATTTCAGAAAACAAGAATGATGCTAAAGTAAGTTTATTATTGAATAATT
GTGATGCTGAATATTCAAAATATTGTGATTGTAAACATACTACTACTCTCGTTAAAAGCGT
TTTAAATGGTAACGACAATACAATTAAGGAAAAGCGTGAACATATTGATTTAGATGATTT
TTCTAAATTTGGATGTGATAAAAATTCCGTTGATACAAACACAAAGGTGTGGGAATGTAA
AAAACCTTATATATTATCCACTAAAGATGTATGTGTACCTCCGAGGAGGCAAGAATTATG
TCTTGGAAACATTGATAGAATATACGATAAAAACCTATTAATGATAAAAGAGCATATTCT
TGCTATTGCAATATATGAATCAAGAATATTGAAACGAAAATATAAGAATAAAGATGATAA
AGAAGTTTGTAAAATCATAAATAAAACTTTCGCTGATATAAGAGATATTATAGGAGGTAC
TGATTATTGGAATGATTTGAGCAATAGAAAATTAGTAGGAAAAATTAACACAAATTCAAA
ATATGTTCACAGGAATAAAAAAAATGATAAGCTTTTTCGTGATGAGTGGTGGAAAGTTAT
TAAAAAAGATGTATGGAATGTGATATCATGGGTATTCAAGGATAAAACTGTTTGTAAAGA
AGATGATATTGAAAATATACCACAATTCTTCAGATGGTTTAGTGAATGGGGTGATGATTA
TTGCCAGGATAAAACAAAAATG
>Colombia Col21_EBA-175KJ419528.1
TATGTATGTATTCCTGATCGTAGAATCCAATTATGCATTGTTAATCTTAGCATTATTAAAA
CATATACAAAAGAGACCATGAAGGATCATTTCATTGAAGCCTCTAAAAAAGAATCTCAA
CTTTTGCTTAAAAAAAAATGATAACAAATATAATTCTAAATTTTGTAATGATTTGAAGAATA
GTTTTTTAGATTATGGACATCTTGCTATGGGAAATGATATGGATTTTGGAGGTTATTCAAC
TAAGGCAGAAAACAAAATTCAAGAAGTTTTTAAAGGGGCTCATGGGGAAATAAGTGAAC
ATAAAATTAAAAATTTTAGAAAAGAATGGTGGAATGAATTTAGAGAGAAACTTTGGGAA
GCTATGTTATCTGAGCATAAAAATAATATAAATAATTGTAAAAATATTCCCCAAGAAGAA
TTACAAATTACTCAATGGATAAAAGAATGGCATGGAGAATTTTTGCTTGAAAGAGATAAT
AGATCAAAATTGCCAAAAAGTAAATGTAAAAATAATACATTATATGAAGCATGTGAGAA
GGAATGTATTGATCCATGTATGAAATATAGAGATTGGATTATTAGAAGTAAATTTGAATG
GCATACGTTATCGAAAGAATATGAAACTCAAAAAGTTCCAAAGGAAAATGCGGAAAATT
ATTTAATCAAAATTTCAGAAAACAAGAATGATGCTAAAGTAAGTTTATTATTGAATAATT
GTGATGCTGAATATTCAAAATATTGTGATTGTAAACATACTACTACTCTCGTTAAAAGCGT
TTTAAATGGTAACGACAATACAATTAAGGAAAAGCGTGAACATATTGATTTAGATGATTT
TTCTAAATTTGGATGTGATAAAAATTCCGTTGATACAAACACAAAGGTGTGGGAATGTAA

AAAACCTTATAAATTATCCACTAAAGATGTATGTGTACCTCCGAGGAGGCAAGAATTATG
TCTTGGAAACATTGATAGAATATACGATAAAAACCTATTAATGATAAAAGAGCATATTCT
TGCTATTGCAATATATGAATCAAGAATATTGAAACGAAAATATAAGAATAAAGATGATAA
AGAAGTTTGTAAAATCATAAATAAAACTTTCGCTGATATAAGAGATATTATAGGAGGTAC
TGATTATTGGAATGATTTGAGCAATAGAAAATTAGTAGGAAAAATTAACACAAATTCAAA
TTATGTTCACAGGAATAAACAAAATGATAAGCTTTTTCGTGATGAGTGGTGGAAAGTTAT
TAAAAAAGATGTATGGAATGTGATATCATGGGTATTCAAGGATAAAACTGTTTGTAAAGA
AGATGATATTGAAAATATACCACAATTCTTCAGATGGTTTAGTGAATGGGGTGATGATTA
TTGCCAGGATAAAACAAAAATG
>Colombia Col24_EBA-175KJ419529.1
TATGTATGTATTCCTGATCGTAGAATCCAATTATGCATTGTTAATCTTAGCATTATTAAAA
CATATACAAAAGAGACCATGAAGGATCATTTCATTGAAGCCTCTAAAAAAGAATCTCAA
CTTTTGCTTAAAAAAAAATGATAACAAATATAATTCTAAATTTTGTAATGATTTGAAGAATA
GTTTTTTAGATTATGGACATCTTGCTATGGGAAATGATATGGATTTTGGAGGTTATTCAAC
TAAGGCAGAAAACAAAATTCAAGAAGTTTTTAAAGGGGCTCATGGGGAAATAAGTGAAC
ATAAAATTAAAAATTTTAGAAAAGAATGGTGGAATGAATTTAGAGAGAAACTTTGGGAA
GCTATGTTATCTGAGCATAAAAATAATATAAATAATTGTAAAAATATTCCCCAAGAAGAA
TTACAAATTACTCAATGGATAAAAGAATGGCATGGAGAATTTTTGCTTGAAAGAGATAAT
AGATCAAAATTGCCAAAAAGTAAATGTAAAAATAATACATTATATGAAGCATGTGAGAA
GGAATGTATTGATCCATGTATGAAATATAGAGATTGGATTATTAGAAGTAAATTTGAATG
GCATACGTTATCGAAAGAATATGAAACTCAAAAAGTTCCAAAGGAAAATGCGGAAAATT
ATTTAATCAAAATTTCAGAAAACAAGAATGATGCTAAAGTAAGTTTATTATTGAATAATT
GTGATGCTGAATATTCAAAATATTGTGATTGTAAACATACTACTACTCTCGTTAAAAGCGT
TTTAAATGGTAACGACAATACAATTAAGGAAAAGCGTGAACATATTGATTTAGATGATTT
TTCTAAATTTGGATGTGATAAAAATTCCGTTGATACAAACACAAAGGTGTGGGAATGTAA
AAAACCTTATAAATTATCCACTAAAGATGTATGTGTACCTCCGAGGAGGCAAGAATTATG
TCTTGGAAACATTGATAGAATATACGATAAAAACCTATTAATGATAAAAGAGCATATTCT
TGCTATTGCAATATATGAATCAAGAATATTGAAACGAAAATATAAGAATAAAGATGATAA
AGAAGTTTGTAAAATCATAAATAAAACTTTCGCTGATATAAGAGATATTATAGGAGGTAC
TGATTATTGGAATGATTTGAGCAATAGAAAATTAGTAGGAAAAATTAACACAAATTCAAA
TTATGTTCACAGGAATAAACAAAATGATAAGCTTTTTCGTGATGAGTGGTGGAAAGTTAT
TAAAAAAGATGTATGGAATGTGATATCATGGGTATTCAAGGATAAAACTGTTTGTAAAGA
AGATGATATTGAAAATATACCACAATTCTTCAGATGGTTTAGTGAATGGGGTGATGATTA
TTGCCAGGATAAAACAAAAATG
>Colombia Col25_EBA-175KJ419530.1
TATGTATGTATTCCTGATCGTAGAATCCAATTATGCATTGTTAATCTTAGCATTATTAAAA
CATATACAAAAGAGACCATGAAGGATCATTTCATTGAAGCCTCTAAAAAAGAATCTCAA
CTTTTGCTTAAAAAAAAATGATAACAAATATAATTCTAAATTTTGTAATGATTTGAAGAATA
GTTTTTTAGATTATGGACATCTTGCTATGGGAAATGATATGGATTTTGGAGGTTATTCAAC
TAAGGCAGAAAACAAAATTCAAGAAGTTTTTAAAGGGGCTCATGGGGAAATAAGTGAAC
ATAAAATTAAAAATTTTAGAAAAGAATGGTGGAATGAATTTAGAGAGAAACTTTGGGAA
GCTATGTTATCTGAGCATAAAAATAATATAAATAATTGTAAAAATATTCCCCAAGAAGAA
TTACAAATTACTCAATGGATAAAAGAATGGCATGGAGAATTTTTGCTTGAAAGAGATAAT
AGATCAAAATTGCCAAAAAGTAAATGTAAAAATAATACATTATATGAAGCATGTGAGAA

GGAATGTATTGATCCATGTATGAAATATAGAGATTGGATTATTAGAAGTAAATTTGAATG
GCATACGTTATCGAAAGAATATGAAACTCAAAAAGTTCCAAAGGAAAATGCGGAAAATT
ATTTAATCAAAATTTCAGAAAACAAGAATGATGCTAAAGTAAGTTTATTATTGAATAATT
GTGATGCTGAATATTCAAAATATTGTGATTGTAAACATACTACTACTCTCGTTAAAAGCGT
TTTAAATGGTAACGACAATACAATTAAGGAAAAGCGTGAACATATTGATTTAGATGATTT
TTCTAAATTTGGATGTGATAAAAATTCCGTTGATACAAACACAAAGGTGTGGGAATGTAA
AAAACCTTATAAATTATCCACTAAAGATGTATGTGTACCTCCGAGGAGGCAAGAATTATG
TCTTGGAAACATTGATAGAATATACGATAAAAACCTATTAATGATAAAAGAGCATATTCT
TGCTATTGCAATATATGAATCAAGAATATTGAAACGAAAATATAAGAATAAAGATGATAA
AGAAGTTTGTAAAATCATAAATAAAACTTTCGCTGATATAAGAGATATTATAGGAGGTAC
TGATTATTGGAATGATTTGAGCAATAGAAAATTAGTAGGAAAAATTAACACAAATTCAAA
TTATGTTCACAGGAATAAACAAAATGATAAGCTTTTTCGTGATGAGTGGTGGAAAGTTAT
TAAAAAAGATGTATGGAATGTGATATCATGGGTATTCAAGGATAAAACTGTTTGTAAAGA
AGATGATATTGAAAATATACCACAATTCTTCAGATGGTTTAGTGAATGGGGTGATGATTA
TTGCCAGGATAAAACAAAAATG
>Colombia Col30_EBA-175KJ419531.1
TATGTATGTATTCCTGATCGTAGAATCCAATTATGCATTGTTAATCTTAGCATTATTAAAA
CATATACAAAAGAGACCATGAAGGATCATTTCATTGAAGCCTCTAAAAAAGAATCTCAA
CTTTTGCTTAAAAAAAAATGATAACAAATATAATTCTAAATTTTGTAATGATTTGAAGAATA
GTTTTTTAGATTATGGACATCTTGCTATGGGAAATGATATGGATTTTGGAGGTTATTCAAC
TAAGGCAGAAAACAAAATTCAAGAAGTTTTTAAAGGGGCTCATGGGGAAATAAGTGAAC
ATGAAATTAAAAATTTTAGAAAAAAATGGTGGAATGAATTTAGAGAGAAACTTTGGGAA
GCTATGTTATCTGAGCATAAAAATAATATAAATAATTGTAAAAATATTCCCCAAGAAGAA
TTACAAATTACTCAATGGATAAAAGAATGGCATGGAGAATTTTTGCTTGAAAGAGATAAT
AGATCAAAATTGCCAAAAAGTAAATGTAAAAATAATACATTATATGAAGCATGTGAGAA
GGAATGTATTGATCCATGTATGAAATATAGAGATTGGATTATTAGAAGTAAATTTGAATG
GCATACGTTATCGAAAGAATATGAAACTCAAAAAGTTTCAAAGGAAAATGCGGAAAATT
ATTTAATCAAAATTTCAGAAAACAAGAATGATGCTAAAGTAAGTTTATTATTGAATAATT
GTGATGCTGAATATTCAAAATATTGTGATTGTAAACATACTACTACTCTCGTTAAAAGCGT
TTTAAATGGTAACGACAATACAATTAAGGAAAAGCGTGAACATATTGATTTAGATGATTT
TTCTAAATTTGGATGTGATAAAAATTCCGTTGATACAAACACAAAGGTGTGGGAATGTAA
AAAACCTTATATATTATCCACTAAAGATGTATGTGTACCTCCGAGGAGGCAAGAATTATG
TCTTGGAAACATTGATAGAATATACGATAAAAACCTATTAATGATAAAAGAGCATATTCT
TGCTATTGCAATATATGAATCAAGAATATTGAAACGAAAATATAAGAATAAAGATGATAA
AGAAGTTTGTAAAATCATAAATAAAACTTTCGCTGATATAAGAGATATTATAGGAGGTAC
TGATTATTGGAATGATTTGAGCAATAGAAAATTAGTAGGAAAAATTAACACAAATTCAAA
ATATGTTCACAGGAATAAAAAAAATGATAAGCTTTTTCGTGATGAGTGGTGGAAAGTTAT
TAAAAAAGATGTATGGAATGTGATATCATGGGTATTCAAGGATAAAACTGTTTGTAAAGA
AGATGATATTGAAAATATACCACAATTCTTCAGATGGTTTAGTGAATGGGGTGATGATTA
TTGCCAGGATAAAACAAAAATG
>French Guiana (Maripasoula) Mar025_EBA-175KJ419532.1
TATGTATGTATTCCTGATCGTAGAATCCAATTATGCATTGTTAATCTTAGCATTATTAAAA
CATATACAAAAGAGACCATGAAGGATCATTTCATTGAAGCCTCTAAAAAAGAATCTCAA
CTTTTGCTTAAAAAAAAATGATAACAAATATAATTCTAAATTTTGTAATGATTTGAAGAATA

GTTTTTTAGATTATGGACATCTTGCTATGGGAAATGATATGGATTTTGGAGGTTATTCAAC
TAAGGCAGAAAACAAAATTCAAGAAGTTTTTAAAGGGGCTCATGGGGAAATAAGTGAAC
ATAAAATTAAAAATTTTAGAAAAGAATGGTGGAATGAATTTAGAGAGAAACTTTGGGAA
GCTATGTTATCTGAGCATAAAAATAATATAAATAATTGTAAAAATATTCCCCAAGAAGAA
TTACAAATTACTCAATGGATAAAAGAATGGCATGGAGAATTTTTGCTTGAAAGAGATAAT
AGATCAAAATTGCCAAAAAGTAAATGTAAAAATAATACATTATATGAAGCATGTGAGAA
GGAATGTATTGATCCATGTATGAAATATAGAGATTGGATTATTAGAAGTAAATTTGAATG
GCATACGTTATCGAAAGAATATGAAACTCAAAAAGTTCCAAAGGAAAATGCGGAAAATT
ATTTAATCAAAATTTCAGAAAACAAGAATGATGCTAAAGTAAGTTTATTATTGAATAATT
GTGATGCTGAATATTCAAAATATTGTGATTGTAAACATACTACTACTCTCGTTAAAAGCGT
TTTAAATGGTAACGACAATACAATTAAGGAAAAGCGTGAACATATTGATTTAGATGATTT
TTCTAAATTTGGATGTGATAAAAATTCCGTTGATACAAACACAAAGGTGTGGGAATGTAA
AAAACCTTATAAATTATCCACTAAAGATGTATGTGTACCTCCGAGGAGGCAAGAATTATG
TCTTGGAAACATTGATAGAATATACGATAAAAACCTATTAATGATAAAAGAGCATATTCT
TGCTATTGCAATATATGAATCAAGAATATTGAAACGAAAATATAAGAATAAAGATGATAA
AGAAGTTTGTAAAATCATAAATAAAACTTTCGCTGATATAAGAGATATTATAGGAGGTAC
TGATTATTGGAATGATTTGAGCAATAGAAAATTAGTAGGAAAAATTAACACAAATTCAAA
TTATGTTCACAGGAATAAACAAAATGATAAGCTTTTTCGTGATGAGTGGTGGAAAGTTAT
TAAAAAAGATGTATGGAATGTGATATCATGGGTATTCAAGGATAAAACTGTTTGTAAAGA
AGATGATATTGAAAATATACCACAATTCTTCAGATGGTTTAGTGAATGGGGTGATGATTA
TTGCCAGGATAAAACAAAAATG
>French Guiana (Maripasoula) Mar026_EBA-175KJ419533.1
TATGTATGTATTCCTGATCGTAGAATCCAATTATGCATTGTTAATCTTAGCATTATTAAAA
CATATACAAAAGAGACCATGAAGGATCATTTCATTGAAGCCTCTAAAAAAGAATCTCAA
CTTTTGCTTAAAAAAAAATGATAACAAATATAATTCTAAATTTTGTAATGATTTGAAGAATA
GTTTTTTAGATTATGGACATCTTGCTATGGGAAATGATATGGATTTTGGAGGTTATTCAAC
TAAGGCAGAAAACAAAATTCAAGAAGTTTTTAAAGGGGCTCATGGGAAAATAAGTGAAC
ATGAAATTAAAAATTTTAGAAAAAAAATGGTGGAATGAATTTAGAGAGAAACTTTGGGAA
GCTATGTTATCTGAGCATAAAAATAATATAAATAATTGTAAAAATATTCCCCAAGAAGAA
TTACAAATTACTCAATGGATAAAAGAATGGCATGGAGAATTTTTGCTTGAAAGAGATAAT
AGATCAAAATTGCCAAAAAGTAAATGTAAAAATAATACATTATATGAAGCATGTGAGAA
GGAATGTATTGATCCATGTATGAAATATAGAGATTGGATTATTAGAAGTAAATTTGAATG
GCATACGTTATCGAAAGAATATGAAACTCAAAAAGTTTCAAAGGAAAATGCGGAAAATT
ATTTAATCAAAATTTCAGAAAACAAGAATGATGCTAAAGTAAGTTTATTATTGAATAATT
GTGATGCTGAATATTCAAAATATTGTGATTGTAAACATACTACTACTCTCGTTAAAAGCGT
TTTAAATGGTAACGACAATACAATTAAGGAAAAGCGTGAACATATTGATTTAGATGATTT
TTCTAAATTTGGATGTGATAAAAATTCCGTTGATACAAACACAAAGGTGTGGGAATGTAA
AAACCCTTATATATTATCCACTAAAGATGTATGTGTACCTCCGAGGAGGCAAGAATTATG
TCTTGGAAACATTGATAGAATATACGATAAAAACCTATTAATGATAAAAGAGCATATTCT
TGCTATTGCAATATATGAATCAAGAATATTGAAACGAAAATATAAGAATAAAGATGATAA
AGAAGTTTGTAAAATCATAAATAAAACTTTCGCTGATATAAGAGATATTATAGGAGGTAC
TGATTATTGGAATGATTTGAGCAATAGAAAATTAGTAGGAAAAATTAACACAAATTCAAA
ATATGTTCACAGGAATAAAAAAAATGATAAGCTTTTTCGTGATGAGTGGTGGAAAGTTAT
TAAAAAAGATGTATGGAATGTGATATCATGGGTATTCAAGGATAAAACTGTTTGTAAAGA

AGATGATATTGAAAATATACCACAATTCTTCAGATGGTTTAGTGAATGGGGTGATGATTA
TTGCCAGGATAAAACAAAAATG
>French Guiana (Maripasoula) Mar027_EBA-175KJ419534.1
TATGTATGTATTCCTGATCGTAGAATCCAATTATGCATTGTTAATCTTAGCATTATTAAAA
CATATACAAAAGAGACCATGAAGGATCATTTCATTGAAGCCTCTAAAAAAGAATCTCAA
CTTTTGCTTAAAAAAAATGATAACAAATATAATTCTAAATTTTGTAATGATTTGAAGAATA
GTTTTTTAGATTATGGACATCTTGCTATGGGAAATGATATGGATTTTGGAGGTTATTCAAC
TAAGGCAGAAAACAAAATTCAAGAAGTTTTTAAAGGGGCTCATGGGAAAATAAGTGAAC
ATGAAATTAAAAATTTTAGAAAAAAATGGTGGAATGAATTTAGAGAGAAACTTTGGGAA
GCTATGTTATCTGAGCATAAAAATAATATAAATAATTGTAAAAATATTCCCCAAGAAGAA
TTACAAATTACTCAATGGATAAAAGAATGGCATGGAGAATTTTTGCTTGAAAGAGATAAT
AGATCAAAATTGCCAAAAAGTAAATGTAAAAATAATACATTATATGAAGCATGTGAGAA
GGAATGTATTGATCCATGTATGAAATATAGAGATTGGATTATTAGAAGTAAATTTGAATG
GCATACGTTATCGAAAGAATATGAAACTCAAAAAGTTTCAAAGGAAAATGCGGAAAATT
ATTTAATCAAAATTTCAGAAAACAAGAATGATGCTAAAGTAAGTTTATTATTGAATAATT
GTGATGCTGAATATTCAAAATATTGTGATTGTAAACATACTACTACTCTCGTTAAAAGCGT
TTTAAATGGTAACGACAATACAATTAAGGAAAAGCGTGAACATATTGATTTAGATGATTT
TTCTAAATTTGGATGTGATAAAAATTCCGTTGATACAAACACAAAGGTGTGGGAATGTAA
AAACCCTTATATATTATCCACTAAAGATGTATGTGTACCTCCGAGGAGGCAAGAATTATG
TCTTGGAAACATTGATAGAATATACGATAAAAACCTATTAATGATAAAAGAGCATATTCT
TGCTATTGCAATATATGAATCAAGAATATTGAAACGAAAATATAAGAATAAAGATGATAA
AGAAGTTTGTAAAATCATAAATAAAACTTTCGCTGATATAAGAGATATTATAGGAGGTAC
TGATTATTGGAATGATTTGAGCAATAGAAAATTAGTAGGAAAAATTAACACAAATTCAAA
ATATGTTCACAGGAATAAAAAAAATGATAAGCTTTTTCGTGATGAGTGGTGGAAAGTTAT
TAAAAAAGATGTATGGAATGTGATATCATGGGTATTCAAGGATAAAACTGTTTGTAAAGA
AGATGATATTGAAAATATACCACAATTCTTCAGATGGTTTAGTGAATGGGGTGATGATTA
TTGCCAGGATAAAACAAAAATG
>French Guiana (Maripasoula) Mar035_EBA-175KJ419535.1
TATGTATGTATTCCTGATCGTAGAATCCAATTATGCATTGTTAATCTTAGCATTATTAAAA
CATATACAAAAGAGACCATGAAGGATCATTTCATTGAAGCCTCTAAAAAAGAATCTCAA
CTTTTGCTTAAAAAAAATGATAACAAATATAATTCTAAATTTTGTAATGATTTGAAGAATA
GTTTTTTAGATTATGGACATCTTGCTATGGGAAATGATATGGATTTTGGAGGTTATTCAAC
TAAGGCAGAAAACAAAATTCAAGAAGTTTTTAAAGGGGCTCATGGGGAAATAAGTGAAC
ATAAAATTAAAAATTTTAGAAAAGAATGGTGGAATGAATTTAGAGAGAAACTTTGGGAA
GCTATGTTATCTGAGCATAAAAATAATATAAATAATTGTAAAAATATTCCCCAAGAAGAA
TTACAAATTACTCAATGGATAAAAGAATGGCATGGAGAATTTTTGCTTGAAAGAGATAAT
AGATCAAAATTGCCAAAAAGTAAATGTAAAAATAATACATTATATGAAGCATGTGAGAA
GGAATGTATTGATCCATGTATGAAATATAGAGATTGGATTATTAGAAGTAAATTTGAATG
GCATACGTTATCGAAAGAATATGAAACTCAAAAAGTTCCAAAGGAAAATGCGGAAAATT
ATTTAATCAAAATTTCAGAAAACAAGAATGATGCTAAAGTAAGTTTATTATTGAATAATT
GTGATGCTGAATATTCAAAATATTGTGATTGTAAACATACTACTACTCTCGTTAAAAGCGT
TTTAAATGGTAACGACAATACAATTAAGGAAAAGCGTGAACATATTGATTTAGATGATTT
TTCTAAATTTGGATGTGATAAAAATTCCGTTGATACAAACACAAAGGTGTGGGAATGTAA
AAAACCTTATAAATTATCCACTAAAGATGTATGTGTACCTCCGAGGAGGCAAGAATTATG

TCTTGGAAACATTGATAGAATATACGATAAAAACCTATTAATGATAAAAGAGCATATTCT
TGCTATTGCAATATATGAATCAAGAATATTGAAACGAAAATATAAGAATAAAGATGATAA
AGAAGTTTGTAAAATCATAAATAAAACTTTCGCTGATATAAGAGATATTATAGGAGGTAC
TGATTATTGGAATGATTTGAGCAATAGAAAATTAGTAGGAAAAATTAACACAAATTCAAA
TTATGTTCACAGGAATAAACAAAATGATAAGCTTTTTCGTGATGAGTGGTGGAAAGTTAT
TAAAAAAGATGTATGGAATGTGATATCATGGGTATTCAAGGATAAAACTGTTTGTAAAGA
AGATGATATTGAAAATATACCACAATTCTTCAGATGGTTTAGTGAATGGGGTGATGATTA
TTGCCAGGATAAAACAAAAATG
>French Guiana (Maripasoula) Mar036_EBA-175KJ419536.1
TATGTATGTATTCCTGATCGTAGAATCCAATTATGCATTGTTAATCTTAGCATTATTAAAA
CATATACAAAAGAGACCATGAAGGATCATTTCATTGAAGCCTCTAAAAAAGAATCTCAA
CTTTTGCTTAAAAAAAATGATAACAAATATAATTCTAAATTTTGTAATGATTTGAAGAATA
GTTTTTTAGATTATGGACATCTTGCTATGGGAAATGATATGGATTTTGGAGGTTATTCAAC
TAAGGCAGAAAACAAAATTCAAGAAGTTTTTAAAGGGGCTCATGGGAAAATAAGTGAAC
ATGAAATTAAAAATTTTAGAAAAAAATGGTGGAATGAATTTAGAGAGAAACTTTGGGAA
GCTATGTTATCTGAGCATAAAAATAATATAAATAATTGTAAAAATATTCCCCAAGAAGAA
TTACAAATTACTCAATGGATAAAAGAATGGCATGGAGAATTTTTGCTTGAAAGAGATAAT
AGATCAAAATTGCCAAAAAGTAAATGTAAAAATAATACATTATATGAAGCATGTGAGAA
GGAATGTATTGATCCATGTATGAAATATAGAGATTGGATTATTAGAAGTAAATTTGAATG
GCATACGTTATCGAAAGAATATGAAACTCAAAAAGTTTCAAAGGAAAATGCGGAAAATT
ATTTAATCAAAATTTCAGAAAACAAGAATGATGCTAAAGTAAGTTTATTATTGAATAATT
GTGATGCTGAATATTCAAAATATTGTGATTGTAAACATACTACTACTCTCGTTAAAAGCGT
TTTAAATGGTAACGACAATACAATTAAGGAAAAGCGTGAACATATTGATTTAGATGATTT
TTCTAAATTTGGATGTGATAAAAATTCCGTTGATACAAACACAAAGGTGTGGGAATGTAA
AAACCCTTATATATTATCCACTAAAGATGTATGTGTACCTCCGAGGAGGCAAGAATTATG
TCTTGGAAACATTGATAGAATATACGATAAAAACCTATTAATGATAAAAGAGCATATTCT
TGCTATTGCAATATATGAATCAAGAATATTGAAACGAAAATATAAGAATAAAGATGATAA
AGAAGTTTGTAAAATCATAAATAAAACTTTCGCTGATATAAGAGATATTATAGGAGGTAC
TGATTATTGGAATGATTTGAGCAATAGAAAATTAGTAGGAAAAATTAACACAAATTCAAA
ATATGTTCACAGGAATAAAAAAAATGATAAGCTTTTTCGTGATGAGTGGTGGAAAGTTAT
TAAAAAAGATGTATGGAATGTGATATCATGGGTATTCAAGGATAAAACTGTTTGTAAAGA
AGATGATATTGAAAATATACCACAATTCTTCAGATGGTTTAGTGAATGGGGTGATGATTA
TTGCCAGGATAAAACAAAAATG
>French Guiana (Maripasoula) Mar074_EBA-175KJ419537.1
TATGTATGTATTCCTGATCGTAGAATCCAATTATGCATTGTTAATCTTAGCATTATTAAAA
CATATACAAAAGAGACCATGAAGGATCATTTCATTGAAGCCTCTAAAAAAGAATCTCAA
CTTTTGCTTAAAAAAAATGATAACAAATATAATTCTAAATTTTGTAATGATTTGAAGAATA
GTTTTTTAGATTATGGACATCTTGCTATGGGAAATGATATGGATTTTGGAGGTTATTCAAC
TAAGGCAGAAAACAAAATTCAAGAAGTTTTTAAAGGGGCTCATGGGAAAATAAGTGAAC
ATGAAATTAAAAATTTTAGAAAAAAATGGTGGAATGAATTTAGAGAGAAACTTTGGGAA
GCTATGTTATCTGAGCATAAAAATAATATAAATAATTGTAAAAATATTCCCCAAGAAGAA
TTACAAATTACTCAATGGATAAAAGAATGGCATGGAGAATTTTTGCTTGAAAGAGATAAT
AGATCAAAATTGCCAAAAAGTAAATGTAAAAATAATACATTATATGAAGCATGTGAGAA
GGAATGTATTGATCCATGTATGAAATATAGAGATTGGATTATTAGAAGTAAATTTGAATG

GCATACGTTATCGAAAGAATATGAAACTCAAAAAGTTTCAAAGGAAAATGCGGAAAATT
ATTTAATCAAAATTTCAGAAAACAAGAATGATGCTAAAGTAAGTTTATTATTGAATAATT
GTGATGCTGAATATTCAAAATATTGTGATTGTAAACATACTACTACTCTCGTTAAAAGCGT
TTTAAATGGTAACGACAATACAATTAAGGAAAAGCGTGAACATATTGATTTAGATGATTT
TTCTAAATTTGGATGTGATAAAAATTCCGTTGATACAAACACAAAGGTGTGGGAATGTAA
AAACCCTTATATATTATCCACTAAAGATGTATGTGTACCTCCGAGGAGGCAAGAATTATG
TCTTGGAAACATTGATAGAATATACGATAAAAACCTATTAATGATAAAAGAGCATATTCT
TGCTATTGCAATATATGAATCAAGAATATTGAAACGAAAATATAAGAATAAAGATGATAA
AGAAGTTTGTAAAATCATAAATAAAACTTTCGCTGATATAAGAGATATTATAGGAGGTAC
TGATTATTGGAATGATTTGAGCAATAGAAAATTAGTAGGAAAAATTAACACAAATTCAAA
ATATGTTCACAGGAATAAAAAAAATGATAAGCTTTTTCGTGATGAGTGGTGGAAAGTTAT
TAAAAAAGATGTATGGAATGTGATATCATGGGTATTCAAGGATAAAACTGTTTGTAAAGA
AGATGATATTGAAAATATACCACAATTCTTCAGATGGTTTAGTGAATGGGGTGATGATTA
TTGCCAGGATAAAACAAAAATG
>French Guiana (Maripasoula) Mar075_EBA-175KJ419538.1
TATGTATGTATTCCTGATCGTAGAATCCAATTATGCATTGTTAATCTTAGCATTATTAAAA
CATATACAAAAGAGACCATGAAGGATCATTTCATTGAAGCCTCTAAAAAAGAATCTCAA
CTTTTGCTTAAAAAAAATGATAACAAATATAATTCTAAATTTTGTAATGATTTGAAGAATA
GTTTTTTAGATTATGGACATCTTGCTATGGGAAATGATATGGATTTTGGAGGTTATTCAAC
TAAGGCAGAAAACAAAATTCAAGAAGTTTTTAAAGGGGCTCATGGGAAAATAAGTGAAC
ATGAAATTAAAAATTTTAGAAAAAAATGGTGGAATGAATTTAGAGAGAAACTTTGGGAA
GCTATGTTATCTGAGCATAAAAATAATATAAATAATTGTAAAAATATTCCCCAAGAAGAA
TTACAAATTACTCAATGGATAAAAGAATGGCATGGAGAATTTTTGCTTGAAAGAGATAAT
AGATCAAAATTGCCAAAAAGTAAATGTAAAAATAATACATTATATGAAGCATGTGAGAA
GGAATGTATTGATCCATGTATGAAATATAGAGATTGGATTATTAGAAGTAAATTTGAATG
GCATACGTTATCGAAAGAATATGAAACTCAAAAAGTTTCAAAGGAAAATGCGGAAAATT
ATTTAATCAAAATTTCAGAAAACAAGAATGATGCTAAAGTAAGTTTATTATTGAATAATT
GTGATGCTGAATATTCAAAATATTGTGATTGTAAACATACTACTACTCTCGTTAAAAGCGT
TTTAAATGGTAACGACAATACAATTAAGGAAAAGCGTGAACATATTGATTTAGATGATTT
TTCTAAATTTGGATGTGATAAAAATTCCGTTGATACAAACACAAAGGTGTGGGAATGTAA
AAACCCTTATATATTATCCACTAAAGATGTATGTGTACCTCCGAGGAGGCAAGAATTATG
TCTTGGAAACATTGATAGAATATACGATAAAAACCTATTAATGATAAAAGAGCATATTCT
TGCTATTGCAATATATGAATCAAGAATATTGAAACGAAAATATAAGAATAAAGATGATAA
AGAAGTTTGTAAAATCATAAATAAAACTTTCGCTGATATAAGAGATATTATAGGAGGTAC
TGATTATTGGAATGATTTGAGCAATAGAAAATTAGTAGGAAAAATTAACACAAATTCAAA
ATATGTTCACAGGAATAAAAAAAATGATAAGCTTTTTCGTGATGAGTGGTGGAAAGTTAT
TAAAAAAGATGTATGGAATGTGATATCATGGGTATTCAAGGATAAAACTGTTTGTAAAGA
AGATGATATTGAAAATATACCACAATTCTTCAGATGGTTTAGTGAATGGGGTGATGATTA
TTGCCAGGATAAAACAAAAATG
>French Guiana (Maripasoula) Mar076_EBA-175KJ419539.1
TATGTATGTATTCCTGATCGTAGAATCCAATTATGCATTGTTAATCTTAGCATTATTAAAA
CATATACAAAAGAGACCATGAAGGATCATTTCATTGAAGCCTCTAAAAAAGAATCTCAA
CTTTTGCTTAAAAAAAATGATAACAAATATAATTCTAAATTTTGTAATGATTTGAAGAATA
GTTTTTTAGATTATGGACATCTTGCTATGGGAAATGATATGGATTTTGGAGGTTATTCAAC

TAAGGCAGAAAACAAAATTCAAGAAGTTTTTAAAGGGGCTCATGGGAAAATAAGTGAAC
ATGAAATTAAAAATTTTAGAAAAAAAATGGTGGAATGAATTTAGAGAGAAACTTTGGGAA
GCTATGTTATCTGAGCATAAAAATAATATAAATAATTGTAAAAATATTCCCCAAGAAGAA
TTACAAATTACTCAATGGATAAAAGAATGGCATGGAGAATTTTTGCTTGAAAGAGATAAT
AGATCAAAATTGCCAAAAAGTAAATGTAAAAATAATACATTATATGAAGCATGTGAGAA
GGAATGTATTGATCCATGTATGAAATATAGAGATTGGATTATTAGAAGTAAATTTGAATG
GCATACGTTATCGAAAGAATATGAAACTCAAAAAGTTTCAAAGGAAAATGCGGAAAATT
ATTTAATCAAAATTTCAGAAAACAAGAATGATGCTAAAGTAAGTTTATTATTGAATAATT
GTGATGCTGAATATTCAAAATATTGTGATTGTAAACATACTACTACTCTCGTTAAAAGCGT
TTTAAATGGTAACGACAATACAATTAAGGAAAAGCGTGAACATATTGATTTAGATGATTT
TTCTAAATTTGGATGTGATAAAAATTCCGTTGATACAAACACAAAGGTGTGGGAATGTAA
AAACCCTTATATATTATCCACTAAAGATGTATGTGTACCTCCGAGGAGGCAAGAATTATG
TCTTGGAAACATTGATAGAATATACGATAAAAACCTATTAATGATAAAAGAGCATATTCT
TGCTATTGCAATATATGAATCAAGAATATTGAAACGAAAATATAAGAATAAAGATGATAA
AGAAGTTTGTAAAATCATAAATAAAACTTTCGCTGATATAAGAGATATTATAGGAGGTAC
TGATTATTGGAATGATTTGAGCAATAGAAAATTAGTAGGAAAAATTAACACAAATTCAAA
ATATGTTCACAGGAATAAAAAAAATGATAAGCTTTTTCGTGATGAGTGGTGGAAAGTTAT
TAAAAAAGATGTATGGAATGTGATATCATGGGTATTCAAGGATAAAACTGTTTGTAAAGA
AGATGATATTGAAAATATACCACAATTCTTCAGATGGTTTAGTGAATGGGGTGATGATTA
TTGCCAGGATAAAACAAAAATG
>French Guiana (Maripasoula) Mar080_EBA-175KJ419540.1
TATGTATGTATTCCTGATCGTAGAATCCAATTATGCATTGTTAATCTTAGCATTATTAAAA
CATATACAAAAGAGACCATGAAGGATCATTTCATTGAAGCCTCTAAAAAAGAATCTCAA
CTTTTGCTTAAAAAAAAATGATAACGAATATAATTCTAAATTTTGTAATGATTTGAAGAATA
GTTTTTTAGATTATGGACATCTTGCTATGGGAAATGATATGGATTTTGGAGGTTATTCAAC
TAAGGCAGAAAACAAAATTCAAGAAGTTTTTAAAGGGGCTCATGGGGAAATAAGTGAAC
ATAAAATTAAAAATTTTAGAAAAGAATGGTGGAATGAATTTAGAGAGAAACTTTGGGAA
GCTATGTTATCTGAGCATAAAAATAATATAAATAATTGTAAAAATATTCCCCAAGAAGAA
TTACAAATTACTCAATGGATAAAAGAATGGCATGGAGAATTTTTGCTTGAAAGATATAAT
AGATCAAAATTGCCAAAAAGTAAATGTAAAAATAATACATTATATGAAGCATGTGAGAA
GGAATGTATTGATCCATGTATGAAATATAGAGATTGGATTATTAGAAGTAAATTTGAATG
GCATACGTTATCGAAAGAATATGAAACTCAAAAAGTTTCAAAGGAAAATGCGGAAAATT
ATTTAATCAAAATTTCAGAAAACAAGAATGATGCTAAAGTAAGTTTATTATTGAATAATT
GTGATGCTGAATATTCAAAATATTGTGATTGTAAACATACTACTACTCTCGTTAAAAGCGT
TTTAAATGGTAACGACAATACAATTAAGGAAAAGCGTGAACATATTGATTTAGATGATTT
TTCTAAATTTGGATGTGATAAAAATTCCGTTGATACAAACACAAAGGTGTGGGAATGTAA
AAAACCTTATATATTATCCACTAAAGATGTATGTGTACCTCCGAGGAGGCAAGAATTATG
TCTTGGAAACATTGATAGAATATACGATAAAAACCTATTAATGATAAAAGAGCATATTCT
TGCTATTGCAATATATGAATCAAGAATATTGAAACGAAAATATAAGAATAAAGATGATAA
AGAAGTTTGTAAAATCATAAATAAAACTTTCGCTGATATAAGAGATATTATAGGAGGTAC
TGATTATTGGAATGATTTGAGCAATAGAAAATTAGTAGGAAAAATTAACACAAATTCAAA
ATATGTTCACAGGAATAAAAAAAATGATAAGCTTTTTCGTGATGAGTGGTGGAAAGTTAT
TAAAAAAGATGTATGGAATGTGATATCATGGGTATTCAAGGATAAAACTGTTTGTAAAGA
AGATGATATTGAAAATATACCACAATTCTTCAGATGGTTTAGTGAATGGGGTGATGATTA

TTGCCAGGATAAAACAAAAATG
>French Guiana (Maripasoula) Mar085_EBA-175KJ419541.1
TATGTATGTATTCCTGATCGTAGAATCCAATTATGCATTGTTAATCTTAGCATTATTAAAA
CATATACAAAAGAGACCATGAAGGATCATTTCATTGAAGCCTCTAAAAAAGAATCTCAA
CTTTTGCTTAAAAAAAAATGATAACAAATATAATTCTAAATTTTGTAATGATTTGAAGAATA
GTTTTTTAGATTATGGACATCTTGCTATGGGAAATGATATGGATTTTGGAGGTTATTCAAC
TAAGGCAGAAAACAAAATTCAAGAAGTTTTTAAAGGGGCTCATGGGAAAATAAGTGAAC
ATGAAATTAAAAATTTTAGAAAAAAATGGTGGAATGAATTTAGAGAGAAACTTTGGGAA
GCTATGTTATCTGAGCATAAAAATAATATAAATAATTGTAAAAATATTCCCCAAGAAGAA
TTACAAATTACTCAATGGATAAAAGAATGGCATGGAGAATTTTTGCTTGAAAGAGATAAT
AGATCAAAATTGCCAAAAAGTAAATGTAAAAATAATACATTATATGAAGCATGTGAGAA
GGAATGTATTGATCCATGTATGAAATATAGAGATTGGATTATTAGAAGTAAATTTGAATG
GCATACGTTATCGAAAGAATATGAAACTCAAAAAGTTTCAAAGGAAAATGCGGAAAATT
ATTTAATCAAAATTTCAGAAAACAAGAATGATGCTAAAGTAAGTTTATTATTGAATAATT
GTGATGCTGAATATTCAAAATATTGTGATTGTAAACATACTACTACTCTCGTTAAAAGCGT
TTTAAATGGTAACGACAATACAATTAAGGAAAAGCGTGAACATATTGATTTAGATGATTT
TTCTAAATTTGGATGTGATAAAAATTCCGTTGATACAAACACAAAGGTGTGGGAATGTAA
AAACCCTTATATATTATCCACTAAAGATGTATGTGTACCTCCGAGGAGGCAAGAATTATG
TCTTGGAAACATTGATAGAATATACGATAAAAACCTATTAATGATAAAAGAGCATATTCT
TGCTATTGCAATATATGAATCAAGAATATTGAAACGAAAATATAAGAATAAAGATGATAA
AGAAGTTTGTAAAATCATAAATAAAACTTTCGCTGATATAAGAGATATTATAGGAGGTAC
TGATTATTGGAATGATTTGAGCAATAGAAAATTAGTAGGAAAAATTAACACAAATTCAAA
ATATGTTCACAGGAATAAAAAAAATGATAAGCTTTTTCGTGATGAGTGGTGGAAAGTTAT
TAAAAAAGATGTATGGAATGTGATATCATGGGTATTCAAGGATAAAACTGTTTGTAAAGA
AGATGATATTGAAAATATACCACAATTCTTCAGATGGTTTAGTGAATGGGGTGATGATTA
TTGCCAGGATAAAACAAAAATG
>French Guiana (Maripasoula) Mar086_EBA-175KJ419542.1
TATGTATGTATTCCTGATCGTAGAATCCAATTATGCATTGTTAATCTTAGCATTATTAAAA
CATATACAAAAGAGACCATGAAGGATCATTTCATTGAAGCCTCTAAAAAAGAATCTCAA
CTTTTGCTTAAAAAAAAATGATAACGAATATAATTCTAAATTTTGTAATGATTTGAAGAATA
GTTTTTTAGATTATGGACATCTTGCTATGGGAAATGATATGGATTTTGGAGGTTATTCAAC
TAAGGCAGAAAACAAAATTCAAGAAGTTTTTAAAGGGGCTCATGGGGAAATAAGTGAAC
ATAAAATTAAAAATTTTAGAAAAGAATGGTGGAATGAATTTAGAGAGAAACTTTGGGAA
GCTATGTTATCTGAGCATAAAAATAATATAAATAATTGTAAAAATATTCCCCAAGAAGAA
TTACAAATTACTCAATGGATAAAAGAATGGCATGGAGAATTTTTGCTTGAAAGATATAAT
AGATCAAAATTGCCAAAAAGTAAATGTAAAAATAATACATTATATGAAGCATGTGAGAA
GGAATGTATTGATCCATGTATGAAATATAGAGATTGGATTATTAGAAGTAAATTTGAATG
GCATACGTTATCGAAAGAATATGAAACTCAAAAAGTTTCAAAGGAAAATGCGGAAAATT
ATTTAATCAAAATTTCAGAAAACAAGAATGATGCTAAAGTAAGTTTATTATTGAATAATT
GTGATGCTGAATATTCAAAATATTGTGATTGTAAACATACTACTACTCTCGTTAAAAGCGT
TTTAAATGGTAACGACAATACAATTAAGGAAAAGCGTGAACATATTGATTTAGATGATTT
TTCTAAATTTGGATGTGATAAAAATTCCGTTGATACAAACACAAAGGTGTGGGAATGTAA
AAAACCTTATATATTATCCACTAAAGATGTATGTGTACCTCCGAGGAGGCAAGAATTATG
TCTTGGAAACATTGATAGAATATACGATAAAAACCTATTAATGATAAAAGAGCATATTCT

TGCTATTGCAATATATGAATCAAGAATATTGAAACGAAAATATAAGAATAAAGATGATAA
AGAAGTTTGTAAAATCATAAATAAAACTTTCGCTGATATAAGAGATATTATAGGAGGTAC
TGATTATTGGAATGATTTGAGCAATAGAAAATTAGTAGGAAAAATTAACACAAATTCAAA
ATATGTTCACAGGAATAAAAAAAATGATAAGCTTTTTCGTGATGAGTGGTGGAAAGTTAT
TAAAAAAGATGTATGGAATGTGATATCATGGGTATTCAAGGATAAAACTGTTTGTAAAGA
AGATGATATTGAAAATATACCACAATTCTTCAGATGGTTTAGTGAATGGGGTGATGATTA
TTGCCAGGATAAAACAAAAATG
>French Guiana (Maripasoula) Mar093_EBA-175KJ419543.1
TATGTATGTATTCCTGATCGTAGAATCCAATTATGCATTGTTAATCTTAGCATTATTAAAA
CATATACAAAAGAGACCATGAAGGATCATTTCATTGAAGCCTCTAAAAAAGAATCTCAA
CTTTTGCTTAAAAAAAATGATAACAAATATAATTCTAAATTTTGTAATGATTTGAAGAATA
GTTTTTTAGATTATGGACATCTTGCTATGGGAAATGATATGGATTTTGGAGGTTATTCAAC
TAAGGCAGAAAACAAAATTCAAGAAGTTTTTAAAGGGGCTCATGGGAAAATAAGTGAAC
ATGAAATTAAAAATTTTAGAAAAAAATGGTGGAATGAATTTAGAGAGAAACTTTGGGAA
GCTATGTTATCTGAGCATAAAAATAATATAAATAATTGTAAAAATATTCCCCAAGAAGAA
TTACAAATTACTCAATGGATAAAAGAATGGCATGGAGAATTTTTGCTTGAAAGAGATAAT
AGATCAAAATTGCCAAAAAGTAAATGTAAAAATAATACATTATATGAAGCATGTGAGAA
GGAATGTATTGATCCATGTATGAAATATAGAGATTGGATTATTAGAAGTAAATTTGAATG
GCATACGTTATCGAAAGAATATGAAACTCAAAAAGTTTCAAAGGAAAATGCGGAAAATT
ATTTAATCAAAATTTCAGAAAACAAGAATGATGCTAAAGTAAGTTTATTATTGAATAATT
GTGATGCTGAATATTCAAAATATTGTGATTGTAAACATACTACTACTCTCGTTAAAAGCGT
TTTAAATGGTAACGACAATACAATTAAGGAAAAGCGTGAACATATTGATTTAGATGATTT
TTCTAAATTTGGATGTGATAAAAATTCCGTTGATACAAACACAAAGGTGTGGGAATGTAA
AAACCCTTATATATTATCCACTAAAGATGTATGTGTACCTCCGAGGAGGCAAGAATTATG
TCTTGGAAACATTGATAGAATATACGATAAAAACCTATTAATGATAAAAGAGCATATTCT
TGCTATTGCAATATATGAATCAAGAATATTGAAACGAAAATATAAGAATAAAGATGATAA
AGAAGTTTGTAAAATCATAAATAAAACTTTCGCTGATATAAGAGATATTATAGGAGGTAC
TGATTATTGGAATGATTTGAGCAATAGAAAATTAGTAGGAAAAATTAACACAAATTCAAA
ATATGTTCACAGGAATAAAAAAAATGATAAGCTTTTTCGTGATGAGTGGTGGAAAGTTAT
TAAAAAAGATGTATGGAATGTGATATCATGGGTATTCAAGGATAAAACTGTTTGTAAAGA
AGATGATATTGAAAATATACCACAATTCTTCAGATGGTTTAGTGAATGGGGTGATGATTA
TTGCCAGGATAAAACAAAAATG
>French Guiana (Maripasoula) Mar094_EBA-175KJ419544.1
TATGTATGTATTCCTGATCGTAGAATCCAATTATGCATTGTTAATCTTAGCATTATTAAAA
CATATACAAAAGAGACCATGAAGGATCATTTCATTGAAGCCTCTAAAAAAGAATCTCAA
CTTTTGCTTAAAAAAAATGATAACGAATATAATTCTAAATTTTGTAATGATTTGAAGAATA
GTTTTTTAGATTATGGACATCTTGCTATGGGAAATGATATGGATTTTGGAGGTTATTCAAC
TAAGGCAGAAAACAAAATTCAAGAAGTTTTTAAAGGGGCTCATGGGGAAATAAGTGAAC
ATAAAATTAAAAATTTTAGAAAAGAATGGTGGAATGAATTTAGAGAGAAACTTTGGGAA
GCTATGTTATCTGAGCATAAAAATAATATAAATAATTGTAAAAATATTCCCCAAGAAGAA
TTACAAATTACTCAATGGATAAAAGAATGGCATGGAGAATTTTTGCTTGAAAGATATAAT
AGATCAAAATTGCCAAAAAGTAAATGTAAAAATAATACATTATATGAAGCATGTGAGAA
GGAATGTATTGATCCATGTATGAAATATAGAGATTGGATTATTAGAAGTAAATTTGAATG
GCATACGTTATCGAAAGAATATGAAACTCAAAAAGTTTCAAAGGAAAATGCGGAAAATT

ATTTAATCAAAATTTCAGAAAACAAGAATGATGCTAAAGTAAGTTTATTATTGAATAATT
GTGATGCTGAATATTCAAAATATTGTGATTGTAAACATACTACTACTCTCGTTAAAAGCGT
TTTAAATGGTAACGACAATACAATTAAGGAAAAGCGTGAACATATTGATTTAGATGATTT
TTCTAAATTTGGATGTGATAAAAATTCCGTTGATACAAACACAAAGGTGTGGGAATGTAA
AAAACCTTATATATTATCCACTAAAGATGTATGTGTACCTCCGAGGAGGCAAGAATTATG
TCTTGGAAACATTGATAGAATATACGATAAAAACCTATTAATGATAAAAGAGCATATTCT
TGCTATTGCAATATATGAATCAAGAATATTGAAACGAAAATATAAGAATAAAGATGATAA
AGAAGTTTGTAAAATCATAAATAAAACTTTCGCTGATATAAGAGATATTATAGGAGGTAC
TGATTATTGGAATGATTTGAGCAATAGAAAATTAGTAGGAAAAATTAACACAAATTCAAA
ATATGTTCACAGGAATAAAAAAAATGATAAGCTTTTTCGTGATGAGTGGTGGAAAGTTAT
TAAAAAAGATGTATGGAATGTGATATCATGGGTATTCAAGGATAAAACTGTTTGTAAAGA
AGATGATATTGAAAATATACCACAATTCTTCAGATGGTTTAGTGAATGGGGTGATGATTA
TTGCCAGGATAAAACAAAAATG
>French Guiana (Maripasoula) Mar095_EBA-175KJ419545.1
TATGTATGTATTCCTGATCGTAGAATCCAATTATGCATTGTTAATCTTAGCATTATTAAAA
CATATACAAAAGAGACCATGAAGGATCATTTCATTGAAGCCTCTAAAAAAGAATCTCAA
CTTTTGCTTAAAAAAAAATGATAACAAATATAATTCTAAATTTTGTAATGATTTGAAGAATA
GTTTTTTAGATTATGGACATCTTGCTATGGGAAATGATATGGATTTTGGAGGTTATTCAAC
TAAGGCAGAAAACAAAATTCAAGAAGTTTTTAAAGGGGCTCATGGGAAAATAAGTGAAC
ATGAAATTAAAAATTTTAGAAAAAAATGGTGGAATGAATTTAGAGAGAAACTTTGGGAA
GCTATGTTATCTGAGCATAAAAATAATATAAATAATTGTAAAAATATTCCCCAAGAAGAA
TTACAAATTACTCAATGGATAAAAGAATGGCATGGAGAATTTTTGCTTGAAAGAGATAAT
AGATCAAAATTGCCAAAAAGTAAATGTAAAAATAATACATTATATGAAGCATGTGAGAA
GGAATGTATTGATCCATGTATGAAATATAGAGATTGGATTATTAGAAGTAAATTTGAATG
GCATACGTTATCGAAAGAATATGAAACTCAAAAAGTTTCAAAGGAAAATGCGGAAAATT
ATTTAATCAAAATTTCAGAAAACAAGAATGATGCTAAAGTAAGTTTATTATTGAATAATT
GTGATGCTGAATATTCAAAATATTGTGATTGTAAACATACTACTACTCTCGTTAAAAGCGT
TTTAAATGGTAACGACAATACAATTAAGGAAAAGCGTGAACATATTGATTTAGATGATTT
TTCTAAATTTGGATGTGATAAAAATTCCGTTGATACAAACACAAAGGTGTGGGAATGTAA
AAACCCTTATATATTATCCACTAAAGATGTATGTGTACCTCCGAGGAGGCAAGAATTATG
TCTTGGAAACATTGATAGAATATACGATAAAAACCTATTAATGATAAAAGAGCATATTCT
TGCTATTGCAATATATGAATCAAGAATATTGAAACGAAAATATAAGAATAAAGATGATAA
AGAAGTTTGTAAAATCATAAATAAAACTTTCGCTGATATAAGAGATATTATAGGAGGTAC
TGATTATTGGAATGATTTGAGCAATAGAAAATTAGTAGGAAAAATTAACACAAATTCAAA
ATATGTTCACAGGAATAAAAAAAATGATAAGCTTTTTCGTGATGAGTGGTGGAAAGTTAT
TAAAAAAGATGTATGGAATGTGATATCATGGGTATTCAAGGATAAAACTGTTTGTAAAGA
AGATGATATTGAAAATATACCACAATTCTTCAGATGGTTTAGTGAATGGGGTGATGATTA
TTGCCAGGATAAAACAAAAATG
>French Guiana (Maripasoula) Mar096_EBA-175KJ419546.1
TATGTATGTATTCCTGATCGTAGAATCCAATTATGCATTGTTAATCTTAGCATTATTAAAA
CATATACAAAAGAGACCATGAAGGATCATTTCATTGAAGCCTCTAAAAAAGAATCTCAA
CTTTTGCTTAAAAAAAAATGATAACAAATATAATTCTAAATTTTGTAATGATTTGAAGAATA
GTTTTTTAGATTATGGACATCTTGCTATGGGAAATGATATGGATTTTGGAGGTTATTCAAC
TAAGGCAGAAAACAAAATTCAAGAAGTTTTTAAAGGGGCTCATGGGGAAATAAGTGAAC

ATAAAATTAAAAATTTTAGAAAAGAATGGTGGAATGAATTTAGAGAGAAACTTTGGGAA
GCTATGTTATCTGAGCATAAAAATAATATAAATAATTGTAAAAATATTCCCCAAGAAGAA
TTACAAATTACTCAATGGATAAAAGAATGGCATGGAGAATTTTTGCTTGAAAGAGATAAT
AGATCAAAATTGCCAAAAAGTAAATGTAAAAATAATACATTATATGAAGCATGTGAGAA
GGAATGTATTGATCCATGTATGAAATATAGAGATTGGATTATTAGAAGTAAATTTGAATG
GCATACGTTATCGAAAGAATATGAAACTCAAAAAGTTCCAAAGGAAAATGCGGAAAATT
ATTTAATCAAAATTTCAGAAAACAAGAATGATGCTAAAGTAAGTTTATTATTGAATAATT
GTGATGCTGAATATTCAAAATATTGTGATTGTAAACATACTACTACTCTCGTTAAAAGCGT
TTTAAATGGTAACGACAATACAATTAAGGAAAAGCGTGAACATATTGATTTAGATGATTT
TTCTAAATTTGGATGTGATAAAAATTCCGTTGATACAAACACAAAGGTGTGGGAATGTAA
AAAACCTTATAAATTATCCACTAAAGATGTATGTGTACCTCCGAGGAGGCAAGAATTATG
TCTTGGAAACATTGATAGAATATACGATAAAAACCTATTAATGATAAAAGAGCATATTCT
TGCTATTGCAATATATGAATCAAGAATATTGAAACGAAAATATAAGAATAAAGATGATAA
AGAAGTTTGTAAAATCATAAATAAAACTTTCGCTGATATAAGAGATATTATAGGAGGTAC
TGATTATTGGAATGATTTGAGCAATAGAAAATTAGTAGGAAAAATTAACACAAATTCAAA
TTATGTTCACAGGAATAAACAAAATGATAAGCTTTTTCGTGATGAGTGGTGGAAAGTTAT
TAAAAAAGATGTATGGAATGTGATATCATGGGTATTCAAGGATAAAACTGTTTGTAAAGA
AGATGATATTGAAAATATACCACAATTCTTCAGATGGTTTAGTGAATGGGGTGATGATTA
TTGCCAGGATAAAACAAAAATG
>Peru Per6_EBA-175KJ419547.1
TATGTATGTATTCCTGATCGTAGAATCCAATTATGCATTGTTAATCTTAGCATTATTAAAA
CATATACAAAAGAGACCATGAAGGATCATTTCATTGAAGCCTCTAAAAAAGAATCTCAA
CTTTTGCTTAAAAAAAAATGATAACAAATATAATTCTAAATTTTGTAATGATTTGAAGAATA
GTTTTTTAGATTATGGACATCTTGCTATGGGAAATGATATGGATTTTGGAGGTTATTCAAC
TAAGGCAGAAAACAAAATTCAAGAAGTTTTTAAAGGGGCTCATGGGAAAATAAGTGAAC
ATGAAATTAAAAATTTTAGAAAAAAAATGGTGGAATGAATTTAGAGAGAAACTTTGGGAA
GCTATGTTATCTGAGCATAAAAATAATATAAATAATTGTAAAAATATTCCCCAAGAAGAA
TTACAAATTACTCAATGGATAAAAGAATGGCATGGAGAATTTTTGCTTGAAAGAGATAAT
AGATCAAAATTGCCAAAAAGTAAATGTAAAAATAATACATTATATGAAGCATGTGAGAA
GGAATGTATTGATCCATGTATGAAATATAGAGATTGGATTATTAGAAGTAAATTTGAATG
GCATACGTTATCGAAAGAATATGAAACTCAAAAAGTTCCAAAGGAAAATGCGGAAAATT
ATTTAATCAAAATTTCAAAAAAACAAGAATGATGCTAAAGTAAGTTTATTATTGAATAATTG
TGATGCTGAATATTCAAAATATTGTGATTGTAAACATACTACTACTCTCGTTAAAAGCGTT
TTAAATGGTAACGACAATACAATTAAGGAAAAGCGTGAACATATTGATTTAGATGATTTT
TCTAAATTTGGATGTGATAAAAATTCCGTTGATACAAACACAAAGGTGTGGGAATGTAAA
AAACCTTATAAATTATCCACTAAAGATGTATGTGTACCTCCGAGGAGGCAAGAATTATGT
CTTGGAAACATTGATAGAATATACGATAAAAACCTATTAATGATAAAAGAGCATATTCTT
GCTATTGCAATATATGAATCAAGAATATTGAAACGAAAATATAAGAATAAAGATGATAA
AGAAGTTTGTAAAATCATAAATAAAACTTTCGCTGATATAAGAGATATTATAGGAGGTAC
TGATTATTGGAATGATTTGAGCAATAGAAAATTAGTAGGAAAAATTAACACAAATTCAAA
TTATGTTCACAGGAATAAACAAAATGATAAGCTTTTTCGTGATGAGTGGTGGAAAGTTAT
TAAAAAAGATGTATGGAATGTGATATCATGGGTATTCAAGGATAAAACTGTTTGTAAAGA
AGATGATATTGAAAATATACCACAATTCTTCAGATGGTTTAGTGAATGGGGTGATGATTA
TTGCCAGGATAAAACAAAAATG

>Peru Per8_EBA-175KJ419548.1
TATGTATGTATTCCTGATCGTAGAATCCAATTATGCATTGTTAATCTTAGCATTATTAAAA
CATATACAAAAGAGACCATGAAGGATCATTTCATTGAAGCCTCTAAAAAAGAATCTCAA
CTTTTGCTTAAAAAAAAATGATAACAAATATAATTCTAAATTTTGTAATGATTTGAAGAATA
GTTTTTTAGATTATGGACATCTTGCTATGGGAAATGATATGGATTTTGGAGGTTATTCAAC
TAAGGCAGAAAACAAAATTCAAGAAGTTTTTAAAGGGGCTCATGGGAAAATAAGTGAAC
ATGAAATTAAAAATTTTAGAAAAAAATGGTGGAATGAATTTAGAGAGAAACTTTGGGAA
GCTATGTTATCTGAGCATAAAAATAATATAAATAATTGTAAAAATATTCCCCAAGAAGAA
TTACAAATTACTCAATGGATAAAAGAATGGCATGGAGAATTTTTGCTTGAAAGAGATAAT
AGATCAAAATTGCCAAAAAGTAAATGTAAAAATAATACATTATATGAAGCATGTGAGAA
GGAATGTATTGATCCATGTATGAAATATAGAGATTGGATTATTAGAAGTAAATTTGAATG
GCATACGTTATCGAAAGAATATGAAACTCAAAAAGTTCCAAAGGAAAATGCGGAAAATT
ATTTAATCAAAATTTCAAAAAACAAGAATGATGCTAAAGTAAGTTTATTATTGAATAATTG
TGATGCTGAATATTCAAAATATTGTGATTGTAAACATACTACTACTCTCGTTAAAAGCGTT
TTAAATGGTAACGACAATACAATTAAGGAAAAGCGTGAACATATTGATTTAGATGATTTT
TCTAAATTTGGATGTGATAAAAATTCCGTTGATACAAACACAAAGGTGTGGGAATGTAAA
AAACCTTATAAATTATCCACTAAAGATGTATGTGTACCTCCGAGGAGGCAAGAATTATGT
CTTGGAAACATTGATAGAATATACGATAAAAACCTATTAATGATAAAAGAGCATATTCTT
GCTATTGCAATATATGAATCAAGAATATTGAAACGAAAATATAAGAATAAAGATGATAA
AGAAGTTTGTAAAATCATAAATAAAACTTTCGCTGATATAAGAGATATTATAGGAGGTAC
TGATTATTGGAATGATTTGAGCAATAGAAAATTAGTAGGAAAAATTAACACAAATTCAAA
TTATGTTCACAGGAATAAACAAAATGATAAGCTTTTTCGTGATGAGTGGTGGAAAGTTAT
TAAAAAAGATGTATGGAATGTGATATCATGGGTATTCAAGGATAAAACTGTTTGTAAAGA
AGATGATATTGAAAATATACCACAATTCTTCAGATGGTTTAGTGAATGGGGTGATGATTA
TTGCCAGGATAAAACAAAAATG
>Peru Per9_EBA-175KJ419549.1
TATGTATGTATTCCTGATCGTAGAATCCAATTATGCATTGTTAATCTTAGCATTATTAAAA
CATATACAAAAGAGACCATGAAGGATCATTTCATTGAAGCCTCTAAAAAAGAATCTCAA
CTTTTGCTTAAAAAAAAATGATAACAAATATAATTCTAAATTTTGTAATGATTTGAAGAATA
GTTTTTTAGATTATGGACATCTTGCTATGGGAAATGATATGGATTTTGGAGGTTATTCAAC
TAAGGCAGAAAACAAAATTCAAGAAGTTTTTAAAGGGGCTCATGGGAAAATAAGTGAAC
ATGAAATTAAAAATTTTAGAAAAAAATGGTGGAATGAATTTAGAGAGAAACTTTGGGAA
GCTATGTTATCTGAGCATAAAAATAATATAAATAATTGTAAAAATATTCCCCAAGAAGAA
TTACAAATTACTCAATGGATAAAAGAATGGCATGGAGAATTTTTGCTTGAAAGAGATAAT
AGATCAAAATTGCCAAAAAGTAAATGTAAAAATAATACATTATATGAAGCATGTGAGAA
GGAATGTATTGATCCATGTATGAAATATAGAGATTGGATTATTAGAAGTAAATTTGAATG
GCATACGTTATCGAAAGAATATGAAACTCAAAAAGTTTCAAAGGAAAATGCGGAAAATT
ATTTAATCAAAATTTCAGAAAACAAGAATGATGCTAAAGTAAGTTTATTATTGAATAATT
GTGATGCTGAATATTCAAAATATTGTGATTGTAAACATACTACTACTCTCGTTAAAAGCGT
TTTAAATGGTAACGACAATACAATTAAGGAAAAGCGTGAACATATTGATTTAGATGATTT
TTCTAAATTTGGATGTGATAAAAATTCCGTTGATACAAACACAAAGGTGTGGGAATGTAA
AAACCCTTATATATTATCCACTAAAGATGTATGTGTACCTCCGAGGAGGCAAGAATTATG
TCTTGGAAACATTGATAGAATATACGATAAAAACCTATTAATGATAAAAGAGCATATTCT
TGCTATTGCAATATATGAATCAAGAATATTGAAACGAAAATATAAGAATAAAGATGATAA

AGAAGTTTGTAAAATCATAAATAAAACTTTCGCTGATATAAGAGATATTATAGGAGGTAC
TGATTATTGGAATGATTTGAGCAATAGAAAATTAGTAGGAAAAATTAACACAAATTCAAA
ATATGTTCACAGGAATAAAAAAAATGATAAGCTTTTTCGTGATGAGTGGTGGAAAGTTAT
TAAAAAAGATGTATGGAATGTGATATCATGGGTATTCAAGGATAAAACTGTTTGTAAAGA
AGATGATATTGAAAATATACCACAATTCTTCAGATGGTTTAGTGAATGGGGTGATGATTA
TTGCCAGGATAAAACAAAAATG
>Peru Per10_EBA-175KJ419550.1
TATGTATGTATTCCTGATCGTAGAATCCAATTATGCATTGTTAATCTTAGCATTATTAAAA
CATATACAAAAGAGACCATGAAGGATCATTTCATTGAAGCCTCTAAAAAAGAATCTCAA
CTTTTGCTTAAAAAAAAATGATAACAAATATAATTCTAAATTTTGTAATGATTTGAAGAATA
GTTTTTTAGATTATGGACATCTTGCTATGGGAAATGATATGGATTTTGGAGGTTATTCAAC
TAAGGCAGAAAACAAAATTCAAGAAGTTTTTAAAGGGGCTCATGGGAAAATAAGTGAAC
ATGAAATTAAAAATTTTAGAAAAAAATGGTGGAATGAATTTAGAGAGAAACTTTGGGAA
GCTATGTTATCTGAGCATAAAAATAATATAAATAATTGTAAAAATATTCCCCAAGAAGAA
TTACAAATTACTCAATGGATAAAAGAATGGCATGGAGAATTTTTGCTTGAAAGAGATAAT
AGATCAAAATTGCCAAAAAGTAAATGTAAAAATAATACATTATATGAAGCATGTGAGAA
GGAATGTATTGATCCATGTATGAAATATAGAGATTGGATTATTAGAAGTAAATTTGAATG
GCATACGTTATCGAAAGAATATGAAACTCAAAAAGTTTCAAAGGAAAATGCGGAAAATT
ATTTAATCAAAATTTCAGAAAACAAGAATGATGCTAAAGTAAGTTTATTATTGAATAATT
GTGATGCTGAATATTCAAAATATTGTGATTGTAAACATACTACTACTCTCGTTAAAAGCGT
TTTAAATGGTAACGACAATACAATTAAGGAAAAGCGTGAACATATTGATTTAGATGATTT
TTCTAAATTTGGATGTGATAAAAATTCCGTTGATACAAACACAAAGGTGTGGGAATGTAA
AAACCCTTATATATTATCCACTAAAGATGTATGTGTACCTCCGAGGAGGCAAGAATTATG
TCTTGGAAACATTGATAGAATATACGATAAAAACCTATTAATGATAAAAGAGCATATTCT
TGCTATTGCAATATATGAATCAAGAATATTGAAACGAAAATATAAGAATAAAGATGATAA
AGAAGTTTGTAAAATCATAAATAAAACTTTCGCTGATATAAGAGATATTATAGGAGGTAC
TGATTATTGGAATGATTTGAGCAATAGAAAATTAGTAGGAAAAATTAACACAAATTCAAA
ATATGTTCACAGGAATAAAAAAAATGATAAGCTTTTTCGTGATGAGTGGTGGAAAGTTAT
TAAAAAAGATGTATGGAATGTGATATCATGGGTATTCAAGGATAAAACTGTTTGTAAAGA
AGATGATATTGAAAATATACCACAATTCTTCAGATGGTTTAGTGAATGGGGTGATGATTA
TTGCCAGGATAAAACAAAAATG
>Peru Per16_EBA-175KJ419551.1
TATGTATGTATTCCTGATCGTAGAATCCAATTATGCATTGTTAATCTTAGCATTATTAAAA
CATATACAAAAGAGACCATGAAGGATCATTTCATTGAAGCCTCTAAAAAAGAATCTCAA
CTTTTGCTTAAAAAAAAATGATAACAAATATAATTCTAAATTTTGTAATGATTTGAAGAATA
GTTTTTTAGATTATGGACATCTTGCTATGGGAAATGATATGGATTTTGGAGGTTATTCAAC
TAAGGCAGAAAACAAAATTCAAGAAGTTTTTAAAGGGGCTCATGGGAAAATAAGTGAAC
ATGAAATTAAAAATTTTAGAAAAAAATGGTGGAATGAATTTAGAGAGAAACTTTGGGAA
GCTATGTTATCTGAGCATAAAAATAATATAAATAATTGTAAAAATATTCCCCAAGAAGAA
TTACAAATTACTCAATGGATAAAAGAATGGCATGGAGAATTTTTGCTTGAAAGAGATAAT
AGATCAAAATTGCCAAAAAGTAAATGTAAAAATAATACATTATATGAAGCATGTGAGAA
GGAATGTATTGATCCATGTATGAAATATAGAGATTGGATTATTAGAAGTAAATTTGAATG
GCATACGTTATCGAAAGAATATGAAACTCAAAAAGTTTCAAAGGAAAATGCGGAAAATT
ATTTAATCAAAATTTCAGAAAACAAGAATGATGCTAAAGTAAGTTTATTATTGAATAATT

GTGATGCTGAATATTCAAAATATTGTGATTGTAAACATACTACTACTCTCGTTAAAAGCGT
TTTAAATGGTAACGACAATACAATTAAGGAAAAGCGTGAACATATTGATTTAGATGATTT
TTCTAAATTTGGATGTGATAAAAATTCCGTTGATACAAACACAAAGGTGTGGGAATGTAA
AAACCCTTATATATTATCCACTAAAGATGTATGTGTACCTCCGAGGAGGCAAGAATTATG
TCTTGGAAACATTGATAGAATATACGATAAAAACCTATTAATGATAAAAGAGCATATTCT
TGCTATTGCAATATATGAATCAAGAATATTGAAACGAAAATATAAGAATAAAGATGATAA
AGAAGTTTGTAAAATCATAAATAAAACTTTCGCTGATATAAGAGATATTATAGGAGGTAC
TGATTATTGGAATGATTTGAGCAATAGAAAATTAGTAGGAAAAATTAACACAAATTCAAA
ATATGTTCACAGGAATAAAAAAAATGATAAGCTTTTTCGTGATGAGTGGTGGAAAGTTAT
TAAAAAAGATGTATGGAATGTGATATCATGGGTATTCAAGGATAAAACTGTTTGTAAAGA
AGATGATATTGAAAATATACCACAATTCTTCAGATGGTTTAGTGAATGGGGTGATGATTA
TTGCCAGGATAAAACAAAAATG
>Peru Per14_EBA-175KJ419552.1
TATGTATGTATTCCTGATCGTAGAATCCAATTATGCATTGTTAATCTTAGCATTATTAAAA
CATATACAAAAGAGACCATGAAGGATCATTTCATTGAAGCCTCTAAAAAAGAATCTCAA
CTTTTGCTTAAAAAAAATGATAACAAATATAATTCTAAATTTTGTAATGATTTGAAGAATA
GTTTTTTAGATTATGGACATCTTGCTATGGGAAATGATATGGATTTTGGAGGTTATTCAAC
TAAGGCAGAAAACAAAATTCAAGAAGTTTTTAAAGGGGCTCATGGGAAAATAAGTGAAC
ATGAAATTAAAAATTTTAGAAAAAAATGGTGGAATGAATTTAGAGAGAAACTTTGGGAA
GCTATGTTATCTGAGCATAAAAATAATATAAATAATTGTAAAAATATTCCCCAAGAAGAA
TTACAAATTACTCAATGGATAAAAGAATGGCATGGAGAATTTTTGCTTGAAAGAGATAAT
AGATCAAAATTGCCAAAAAGTAAATGTAAAAATAATACATTATATGAAGCATGTGAGAA
GGAATGTATTGATCCATGTATGAAATATAGAGATTGGATTATTAGAAGTAAATTTGAATG
GCATACGTTATCGAAAGAATATGAAACTCAAAAAGTTCCAAAGGAAAATGCGGAAAATT
ATTTAATCAAAATTTCAAAAAACAAGAATGATGCTAAAGTAAGTTTATTATTGAATAATTG
TGATGCTGAATATTCAAAATATTGTGATTGTAAACATACTACTACTCTCGTTAAAAGCGTT
TTAAATGGTAACGACAATACAATTAAGGAAAAGCGTGAACATATTGATTTAGATGATTTT
TCTAAATTTGGATGTGATAAAAATTCCGTTGATACAAACACAAAGGTGTGGGAATGTAAA
AAACCTTATAAATTATCCACTAAAGATGTATGTGTACCTCCGAGGAGGCAAGAATTATGT
CTTGGAAACATTGATAGAATATACGATAAAAACCTATTAATGATAAAAGAGCATATTCTT
GCTATTGCAATATATGAATCAAGAATATTGAAACGAAAATATAAGAATAAAGATGATAA
AGAAGTTTGTAAAATCATAAATAAAACTTTCGCTGATATAAGAGATATTATAGGAGGTAC
TGATTATTGGAATGATTTGAGCAATAGAAAATTAGTAGGAAAAATTAACACAAATTCAAA
TTATGTTCACAGGAATAAACAAAATGATAAGCTTTTTCGTGATGAGTGGTGGAAAGTTAT
TAAAAAAGATGTATGGAATGTGATATCATGGGTATTCAAGGATAAAACTGTTTGTAAAGA
AGATGATATTGAAAATATACCACAATTCTTCAGATGGTTTAGTGAATGGGGTGATGATTA
TTGCCAGGATAAAACAAAAATG
>Peru Per18_EBA-175KJ419553.1
TATGTATGTATTCCTGATCGTAGAATCCAATTATGCATTGTTAATCTTAGCATTATTAAAA
CATATACAAAAGAGACCATGAAGGATCATTTCATTGAAGCCTCTAAAAAAGAATCTCAA
CTTTTGCTTAAAAAAAATGATAACAAATATAATTCTAAATTTTGTAATGATTTGAAGAATA
GTTTTTTAGATTATGGACATCTTGCTATGGGAAATGATATGGATTTTGGAGGTTATTCAAC
TAAGGCAGAAAACAAAATTCAAGAAGTTTTTAAAGGGGCTCATGGGAAAATAAGTGAAC
ATGAAATTAAAAATTTTAGAAAAAAATGGTGGAATGAATTTAGAGAGAAACTTTGGGAA

GCTATGTTATCTGAGCATAAAAATAATATAAATAATTGTAAAAATATTCCCCAAGAAGAA
TTACAAATTACTCAATGGATAAAAGAATGGCATGGAGAATTTTTGCTTGAAAGAGATAAT
AGATCAAAATTGCCAAAAAGTAAATGTAAAAATAATACATTATATGAAGCATGTGAGAA
GGAATGTATTGATCCATGTATGAAATATAGAGATTGGATTATTAGAAGTAAATTTGAATG
GCATACGTTATCGAAAGAATATGAAACTCAAAAAGTTTCAAAGGAAAATGCGGAAAATT
ATTTAATCAAAATTTCAGAAAACAAGAATGATGCTAAAGTAAGTTTATTATTGAATAATT
GTGATGCTGAATATTCAAAATATTGTGATTGTAAACATACTACTACTCTCGTTAAAAGCGT
TTTAAATGGTAACGACAATACAATTAAGGAAAAGCGTGAACATATTGATTTAGATGATTT
TTCTAAATTTGGATGTGATAAAAATTCCGTTGATACAAACACAAAGGTGTGGGAATGTAA
AAACCCTTATATATTATCCACTAAAGATGTATGTGTACCTCCGAGGAGGCAAGAATTATG
TCTTGGAAACATTGATAGAATATACGATAAAAACCTATTAATGATAAAAGAGCATATTCT
TGCTATTGCAATATATGAATCAAGAATATTGAAACGAAAATATAAGAATAAAGATGATAA
AGAAGTTTGTAAAATCATAAATAAAACTTTCGCTGATATAAGAGATATTATAGGAGGTAC
TGATTATTGGAATGATTTGAGCAATAGAAAATTAGTAGGAAAAATTAACACAAATTCAAA
ATATGTTCACAGGAATAAAAAAAATGATAAGCTTTTTCGTGATGAGTGGTGGAAAGTTAT
TAAAAAAGATGTATGGAATGTGATATCATGGGTATTCAAGGATAAAACTGTTTGTAAAGA
AGATGATATTGAAAATATACCACAATTCTTCAGATGGTTTAGTGAATGGGGTGATGATTA
TTGCCAGGATAAAACAAAAATG
>Peru Per19_EBA-175KJ419554.1
TATGTATGTATTCCTGATCGTAGAATCCAATTATGCATTGTTAATCTTAGCATTATTAAAA
CATATACAAAAGAGACCATGAAGGATCATTTCATTGAAGCCTCTAAAAAAGAATCTCAA
CTTTTGCTTAAAAAAAAATGATAACAAATATAATTCTAAATTTTGTAATGATTTGAAGAATA
GTTTTTTAGATTATGGACATCTTGCTATGGGAAATGATATGGATTTTGGAGGTTATTCAAC
TAAGGCAGAAAACAAAATTCAAGAAGTTTTTAAAGGGGCTCATGGGAAAATAAGTGAAC
ATGAAATTAAAAATTTTAGAAAAAAATGGTGGAATGAATTTAGAGAGAAACTTTGGGAA
GCTATGTTATCTGAGCATAAAAATAATATAAATAATTGTAAAAATATTCCCCAAGAAGAA
TTACAAATTACTCAATGGATAAAAGAATGGCATGGAGAATTTTTGCTTGAAAGAGATAAT
AGATCAAAATTGCCAAAAAGTAAATGTAAAAATAATACATTATATGAAGCATGTGAGAA
GGAATGTATTGATCCATGTATGAAATATAGAGATTGGATTATTAGAAGTAAATTTGAATG
GCATACGTTATCGAAAGAATATGAAACTCAAAAAGTTTCAAAGGAAAATGCGGAAAATT
ATTTAATCAAAATTTCAGAAAACAAGAATGATGCTAAAGTAAGTTTATTATTGAATAATT
GTGATGCTGAATATTCAAAATATTGTGATTGTAAACATACTACTACTCTCGTTAAAAGCGT
TTTAAATGGTAACGACAATACAATTAAGGAAAAGCGTGAACATATTGATTTAGATGATTT
TTCTAAATTTGGATGTGATAAAAATTCCGTTGATACAAACACAAAGGTGTGGGAATGTAA
AAACCCTTATATATTATCCACTAAAGATGTATGTGTACCTCCGAGGAGGCAAGAATTATG
TCTTGGAAACATTGATAGAATATACGATAAAAACCTATTAATGATAAAAGAGCATATTCT
TGCTATTGCAATATATGAATCAAGAATATTGAAACGAAAATATAAGAATAAAGATGATAA
AGAAGTTTGTAAAATCATAAATAAAACTTTCGCTGATATAAGAGATATTATAGGAGGTAC
TGATTATTGGAATGATTTGAGCAATAGAAAATTAGTAGGAAAAATTAACACAAATTCAAA
ATATGTTCACAGGAATAAAAAAAATGATAAGCTTTTTCGTGATGAGTGGTGGAAAGTTAT
TAAAAAAGATGTATGGAATGTGATATCATGGGTATTCAAGGATAAAACTGTTTGTAAAGA
AGATGATATTGAAAATATACCACAATTCTTCAGATGGTTTAGTGAATGGGGTGATGATTA
TTGCCAGGATAAAACAAAAATG
>Peru Per22_EBA-175KJ419555.1

TATGTATGTATTCCTGATCGTAGAATCCAATTATGCATTGTTAATCTTAGCATTATTAAAA
CATATACAAAAGAGACCATGAAGGATCATTTCATTGAAGCCTCTAAAAAAGAATCTCAA
CTTTTGCTTAAAAAAAAATGATAACAAATATAATTCTAAATTTTGTAATGATTTGAAGAATA
GTTTTTTAGATTATGGACATCTTGCTATGGGAAATGATATGGATTTTGGAGGTTATTCAAC
TAAGGCAGAAAACAAAATTCAAGAAGTTTTTAAAGGGGCTCATGGGAAAATAAGTGAAC
ATGAAATTAAAAATTTTAGAAAAAAATGGTGGAATGAATTTAGAGAGAAACTTTGGGAA
GCTATGTTATCTGAGCATAAAAATAATATAAATAATTGTAAAAATATTCCCCAAGAAGAA
TTACAAATTACTCAATGGATAAAAGAATGGCATGGAGAATTTTTGCTTGAAAGAGATAAT
AGATCAAAATTGCCAAAAAGTAAATGTAAAAATAATACATTATATGAAGCATGTGAGAA
GGAATGTATTGATCCATGTATGAAATATAGAGATTGGATTATTAGAAGTAAATTTGAATG
GCATACGTTATCGAAAGAATATGAAACTCAAAAAGTTTCAAAGGAAAATGCGGAAAATT
ATTTAATCAAAATTTCAGAAAACAAGAATGATGCTAAAGTAAGTTTATTATTGAATAATT
GTGATGCTGAATATTCAAAATATTGTGATTGTAAACATACTACTACTCTCGTTAAAAGCGT
TTTAAATGGTAACGACAATACAATTAAGGAAAAGCGTGAACATATTGATTTAGATGATTT
TTCTAAATTTGGATGTGATAAAAATTCCGTTGATACAAACACAAAGGTGTGGGAATGTAA
AAACCCTTATATATTATCCACTAAAGATGTATGTGTACCTCCGAGGAGGCAAGAATTATG
TCTTGGAAACATTGATAGAATATACGATAAAAACCTATTAATGATAAAAGAGCATATTCT
TGCTATTGCAATATATGAATCAAGAATATTGAAACGAAAATATAAGAATAAAGATGATAA
AGAAGTTTGTAAAATCATAAATAAAACTTTCGCTGATATAAGAGATATTATAGGAGGTAC
TGATTATTGGAATGATTTGAGCAATAGAAAATTAGTAGGAAAAATTAACACAAATTCAAA
ATATGTTCACAGGAATAAAAAAAATGATAAGCTTTTTCGTGATGAGTGGTGGAAAGTTAT
TAAAAAAGATGTATGGAATGTGATATCATGGGTATTCAAGGATAAAACTGTTTGTAAAGA
AGATGATATTGAAAATATACCACAATTCTTCAGATGGTTTAGTGAATGGGGTGATGATTA
TTGCCAGGATAAAACAAAAATG
>Peru Per24_EBA-175KJ419556.1
TATGTATGTATTCCTGATCGTAGAATCCAATTATGCATTGTTAATCTTAGCATTATTAAAA
CATATACAAAAGAGACCATGAAGGATCATTTCATTGAAGCCTCTAAAAAAGAATCTCAA
CTTTTGCTTAAAAAAAAATGATAACAAATATAATTCTAAATTTTGTAATGATTTGAAGAATA
GTTTTTTAGATTATGGACATCTTGCTATGGGAAATGATATGGATTTTGGAGGTTATTCAAC
TAAGGCAGAAAACAAAATTCAAGAAGTTTTTAAAGGGGCTCATGGGAAAATAAGTGAAC
ATGAAATTAAAAATTTTAGAAAAAAATGGTGGAATGAATTTAGAGAGAAACTTTGGGAA
GCTATGTTATCTGAGCATAAAAATAATATAAATAATTGTAAAAATATTCCCCAAGAAGAA
TTACAAATTACTCAATGGATAAAAGAATGGCATGGAGAATTTTTGCTTGAAAGAGATAAT
AGATCAAAATTGCCAAAAAGTAAATGTAAAAATAATACATTATATGAAGCATGTGAGAA
GGAATGTATTGATCCATGTATGAAATATAGAGATTGGATTATTAGAAGTAAATTTGAATG
GCATACGTTATCGAAAGAATATGAAACTCAAAAAGTTTCAAAGGAAAATGCGGAAAATT
ATTTAATCAAAATTTCAGAAAACAAGAATGATGCTAAAGTAAGTTTATTATTGAATAATT
GTGATGCTGAATATTCAAAATATTGTGATTGTAAACATACTACTACTCTCGTTAAAAGCGT
TTTAAATGGTAACGACAATACAATTAAGGAAAAGCGTGAACATATTGATTTAGATGATTT
TTCTAAATTTGGATGTGATAAAAATTCCGTTGATACAAACACAAAGGTGTGGGAATGTAA
AAACCCTTATATATTATCCACTAAAGATGTATGTGTACCTCCGAGGAGGCAAGAATTATG
TCTTGGAAACATTGATAGAATATACGATAAAAACCTATTAATGATAAAAGAGCATATTCT
TGCTATTGCAATATATGAATCAAGAATATTGAAACGAAAATATAAGAATAAAGATGATAA
AGAAGTTTGTAAAATCATAAATAAAACTTTCGCTGATATAAGAGATATTATAGGAGGTAC

TGATTATTGGAATGATTTGAGCAATAGAAAATTAGTAGGAAAAATTAACACAAATTCAAA
ATATGTTCACAGGAATAAAAAAAATGATAAGCTTTTTCGTGATGAGTGGTGGAAAGTTAT
TAAAAAAGATGTATGGAATGTGATATCATGGGTATTCAAGGATAAAACTGTTTGTAAAGA
AGATGATATTGAAAATATACCACAATTCTTCAGATGGTTTAGTGAATGGGGTGATGATTA
TTGCCAGGATAAAACAAAAATG
>Peru Per25_EBA-175KJ419557.1
TATGTATGTATTCCTGATCGTAGAATCCAATTATGCATTGTTAATCTTAGCATTATTAAAA
CATATACAAAAGAGACCATGAAGGATCATTTCATTGAAGCCTCTAAAAAAGAATCTCAA
CTTTTGCTTAAAAAAAATGATAACAAATATAATTCTAAATTTTGTAATGATTTGAAGAATA
GTTTTTTAGATTATGGACATCTTGCTATGGGAAATGATATGGATTTTGGAGGTTATTCAAC
TAAGGCAGAAAACAAAATTCAAGAAGTTTTTAAAGGGGCTCATGGGAAAATAAGTGAAC
ATGAAATTAAAAATTTTAGAAAAAAATGGTGGAATGAATTTAGAGAGAAACTTTGGGAA
GCTATGTTATCTGAGCATAAAAATAATATAAATAATTGTAAAAATATTCCCCAAGAAGAA
TTACAAATTACTCAATGGATAAAAGAATGGCATGGAGAATTTTTGCTTGAAAGAGATAAT
AGATCAAAATTGCCAAAAAGTAAATGTAAAAATAATACATTATATGAAGCATGTGAGAA
GGAATGTATTGATCCATGTATGAAATATAGAGATTGGATTATTAGAAGTAAATTTGAATG
GCATACGTTATCGAAAGAATATGAAACTCAAAAAGTTTCAAAGGAAAATGCGGAAAATT
ATTTAATCAAAATTTCAGAAAACAAGAATGATGCTAAAGTAAGTTTATTATTGAATAATT
GTGATGCTGAATATTCAAAATATTGTGATTGTAAACATACTACTACTCTCGTTAAAAGCGT
TTTAAATGGTAACGACAATACAATTAAGGAAAAGCGTGAACATATTGATTTAGATGATTT
TTCTAAATTTGGATGTGATAAAAATTCCGTTGATACAAACACAAAGGTGTGGGAATGTAA
AAACCCTTATATATTATCCACTAAAGATGTATGTGTACCTCCGAGGAGGCAAGAATTATG
TCTTGGAAACATTGATAGAATATACGATAAAAACCTATTAATGATAAAAGAGCATATTCT
TGCTATTGCAATATATGAATCAAGAATATTGAAACGAAAATATAAGAATAAAGATGATAA
AGAAGTTTGTAAAATCATAAATAAAACTTTCGCTGATATAAGAGATATTATAGGAGGTAC
TGATTATTGGAATGATTTGAGCAATAGAAAATTAGTAGGAAAAATTAACACAAATTCAAA
ATATGTTCACAGGAATAAAAAAAATGATAAGCTTTTTCGTGATGAGTGGTGGAAAGTTAT
TAAAAAAGATGTATGGAATGTGATATCATGGGTATTCAAGGATAAAACTGTTTGTAAAGA
AGATGATATTGAAAATATACCACAATTCTTCAGATGGTTTAGTGAATGGGGTGATGATTA
TTGCCAGGATAAAACAAAAATG
>Peru Per27_EBA-175KJ419558.1
TATGTATGTATTCCTGATCGTAGAATCCAATTATGCATTGTTAATCTTAGCATTATTAAAA
CATATACAAAAGAGACCATGAAGGATCATTTCATTGAAGCCTCTAAAAAAGAATCTCAA
CTTTTGCTTAAAAAAAATGATAACAAATATAATTCTAAATTTTGTAATGATTTGAAGAATA
GTTTTTTAGATTATGGACATCTTGCTATGGGAAATGATATGGATTTTGGAGGTTATTCAAC
TAAGGCAGAAAACAAAATTCAAGAAGTTTTTAAAGGGGCTCATGGGAAAATAAGTGAAC
ATGAAATTAAAAATTTTAGAAAAAAATGGTGGAATGAATTTAGAGAGAAACTTTGGGAA
GCTATGTTATCTGAGCATAAAAATAATATAAATAATTGTAAAAATATTCCCCAAGAAGAA
TTACAAATTACTCAATGGATAAAAGAATGGCATGGAGAATTTTTGCTTGAAAGAGATAAT
AGATCAAAATTGCCAAAAAGTAAATGTAAAAATAATACATTATATGAAGCATGTGAGAA
GGAATGTATTGATCCATGTATGAAATATAGAGATTGGATTATTAGAAGTAAATTTGAATG
GCATACGTTATCGAAAGAATATGAAACTCAAAAAGTTTCAAAGGAAAATGCGGAAAATT
ATTTAATCAAAATTTCAGAAAACAAGAATGATGCTAAAGTAAGTTTATTATTGAATAATT
GTGATGCTGAATATTCAAAATATTGTGATTGTAAACATACTACTACTCTCGTTAAAAGCGT

TTTAAATGGTAACGACAATACAATTAAGGAAAAGCGTGAACATATTGATTTAGATGATTT
TTCTAAATTTGGATGTGATAAAAATTCCGTTGATACAAACACAAAGGTGTGGGAATGTAA
AAACCCTTATATATTATCCACTAAAGATGTATGTGTACCTCCGAGGAGGCAAGAATTATG
TCTTGGAAACATTGATAGAATATACGATAAAAACCTATTAATGATAAAAGAGCATATTCT
TGCTATTGCAATATATGAATCAAGAATATTGAAACGAAAATATAAGAATAAAGATGATAA
AGAAGTTTGTAAAATCATAAATAAAACTTTCGCTGATATAAGAGATATTATAGGAGGTAC
TGATTATTGGAATGATTTGAGCAATAGAAAATTAGTAGGAAAAATTAACACAAATTCAAA
ATATGTTCACAGGAATAAAAAAAATGATAAGCTTTTTCGTGATGAGTGGTGGAAAGTTAT
TAAAAAAGATGTATGGAATGTGATATCATGGGTATTCAAGGATAAAACTGTTTGTAAAGA
AGATGATATTGAAAATATACCACAATTCTTCAGATGGTTTAGTGAATGGGGTGATGATTA
TTGCCAGGATAAAACAAAAATG
>Peru Per31_EBA-175KJ419559.1
TATGTATGTATTCCTGATCGTAGAATCCAATTATGCATTGTTAATCTTAGCATTATTAAAA
CATATACAAAAGAGACCATGAAGGATCATTTCATTGAAGCCTCTAAAAAAGAATCTCAA
CTTTTGCTTAAAAAAAAATGATAACAAATATAATTCTAAATTTTGTAATGATTTGAAGAATA
GTTTTTTAGATTATGGACATCTTGCTATGGGAAATGATATGGATTTTGGAGGTTATTCAAC
TAAGGCAGAAAACAAAATTCAAGAAGTTTTTAAAGGGGCTCATGGGAAAATAAGTGAAC
ATGAAATTAAAAATTTTAGAAAAAAATGGTGGAATGAATTTAGAGAGAAACTTTGGGAA
GCTATGTTATCTGAGCATAAAAATAATATAAATAATTGTAAAAATATTCCCCAAGAAGAA
TTACAAATTACTCAATGGATAAAAGAATGGCATGGAGAATTTTTGCTTGAAAGAGATAAT
AGATCAAAATTGCCAAAAAGTAAATGTAAAAATAATACATTATATGAAGCATGTGAGAA
GGAATGTATTGATCCATGTATGAAATATAGAGATTGGATTATTAGAAGTAAATTTGAATG
GCATACGTTATCGAAAGAATATGAAACTCAAAAAGTTTCAAAGGAAAATGCGGAAAATT
ATTTAATCAAAATTTCAGAAAACAAGAATGATGCTAAAGTAAGTTTATTATTGAATAATT
GTGATGCTGAATATTCAAAATATTGTGATTGTAAACATACTACTACTCTCGTTAAAAGCGT
TTTAAATGGTAACGACAATACAATTAAGGAAAAGCGTGAACATATTGATTTAGATGATTT
TTCTAAATTTGGATGTGATAAAAATTCCGTTGATACAAACACAAAGGTGTGGGAATGTAA
AAACCCTTATATATTATCCACTAAAGATGTATGTGTACCTCCGAGGAGGCAAGAATTATG
TCTTGGAAACATTGATAGAATATACGATAAAAACCTATTAATGATAAAAGAGCATATTCT
TGCTATTGCAATATATGAATCAAGAATATTGAAACGAAAATATAAGAATAAAGATGATAA
AGAAGTTTGTAAAATCATAAATAAAACTTTCGCTGATATAAGAGATATTATAGGAGGTAC
TGATTATTGGAATGATTTGAGCAATAGAAAATTAGTAGGAAAAATTAACACAAATTCAAA
ATATGTTCACAGGAATAAAAAAAATGATAAGCTTTTTCGTGATGAGTGGTGGAAAGTTAT
TAAAAAAGATGTATGGAATGTGATATCATGGGTATTCAAGGATAAAACTGTTTGTAAAGA
AGATGATATTGAAAATATACCACAATTCTTCAGATGGTTTAGTGAATGGGGTGATGATTA
TTGCCAGGATAAAACAAAAATG
>Peru Per32_EBA-175KJ419560.1
TATGTATGTATTCCTGATCGTAGAATCCAATTATGCATTGTTAATCTTAGCATTATTAAAA
CATATACAAAAGAGACCATGAAGGATCATTTCATTGAAGCCTCTAAAAAAGAATCTCAA
CTTTTGCTTAAAAAAAAATGATAACAAATATAATTCTAAATTTTGTAATGATTTGAAGAATA
GTTTTTTAGATTATGGACATCTTGCTATGGGAAATGATATGGATTTTGGAGGTTATTCAAC
TAAGGCAGAAAACAAAATTCAAGAAGTTTTTAAAGGGGCTCATGGGAAAATAAGTGAAC
ATGAAATTAAAAATTTTAGAAAAAAATGGTGGAATGAATTTAGAGAGAAACTTTGGGAA
GCTATGTTATCTGAGCATAAAAATAATATAAATAATTGTAAAAATATTCCCCAAGAAGAA

TTACAAATTACTCAATGGATAAAAGAATGGCATGGAGAATTTTTGCTTGAAAGAGATAAT
AGATCAAAATTGCCAAAAAGTAAATGTAAAAATAATACATTATATGAAGCATGTGAGAA
GGAATGTATTGATCCATGTATGAAATATAGAGATTGGATTATTAGAAGTAAATTTGAATG
GCATACGTTATCGAAAGAATATGAAACTCAAAAAGTTTCAAAGGAAAATGCGGAAAATT
ATTTAATCAAAATTTCAGAAAACAAGAATGATGCTAAAGTAAGTTTATTATTGAATAATT
GTGATGCTGAATATTCAAAATATTGTGATTGTAAACATACTACTACTCTCGTTAAAAGCGT
TTTAAATGGTAACGACAATACAATTAAGGAAAAGCGTGAACATATTGATTTAGATGATTT
TTCTAAATTTGGATGTGATAAAAAATTCCGTTGATACAAACACAAAGGTGTGGGAATGTAA
AAACCCTTATATATTATCCACTAAAGATGTATGTGTACCTCCGAGGAGGCAAGAATTATG
TCTTGGAAACATTGATAGAATATACGATAAAAACCTATTAATGATAAAAGAGCATATTCT
TGCTATTGCAATATATGAATCAAGAATATTGAAACGAAAATATAAGAATAAAGATGATAA
AGAAGTTTGTAAAATCATAAATAAAACTTTCGCTGATATAAGAGATATTATAGGAGGTAC
TGATTATTGGAATGATTTGAGCAATAGAAAATTAGTAGGAAAAAATTAACACAAATTCAAA
ATATGTTCACAGGAATAAAAAAAAATGATAAGCTTTTTCGTGATGAGTGGTGGAAAGTTAT
TAAAAAAGATGTATGGAATGTGATATCATGGGTATTCAAGGATAAAACTGTTTGTAAAGA
AGATGATATTGAAAATATACCACAATTCTTCAGATGGTTTAGTGAATGGGGTGATGATTA
TTGCCAGGATAAAACAAAAATG
>Peru Per45_EBA-175KJ419561.1
TATGTATGTATTCCTGATCGTAGAATCCAATTATGCATTGTTAATCTTAGCATTATTAAAA
CATATACAAAAGAGACCATGAAGGATCATTTCATTGAAGCCTCTAAAAAAGAATCTCAA
CTTTTGCTTAAAAAAAAATGATAACAAATATAATTCTAAATTTTGTAATGATTTGAAGAATA
GTTTTTTAGATTATGGACATCTTGCTATGGGAAATGATATGGATTTTGGAGGTTATTCAAC
TAAGGCAGAAAACAAAATTCAAGAAGTTTTTAAAGGGGCTCATGGGAAAATAAGTGAAC
ATGAAATTAAAAATTTTAGAAAAAAATGGTGGAATGAATTTAGAGAGAAACTTTGGGAA
GCTATGTTATCTGAGCATAAAAATAATATAAATAATTGTAAAAATATTCCCCAAGAAGAA
TTACAAATTACTCAATGGATAAAAGAATGGCATGGAGAATTTTTGCTTGAAAGAGATAAT
AGATCAAAATTGCCAAAAAGTAAATGTAAAAATAATACATTATATGAAGCATGTGAGAA
GGAATGTATTGATCCATGTATGAAATATAGAGATTGGATTATTAGAAGTAAATTTGAATG
GCATACGTTATCGAAAGAATATGAAACTCAAAAAGTTCCAAAGGAAAATGCGGAAAATT
ATTTAATCAAAATTTCAAAAAACAAGAATGATGCTAAAGTAAGTTTATTATTGAATAATTG
TGATGCTGAATATTCAAAATATTGTGATTGTAAACATACTACTACTCTCGTTAAAAGCGTT
TTAAATGGTAACGACAATACAATTAAGGAAAAGCGTGAACATATTGATTTAGATGATTTT
TCTAAATTTGGATGTGATAAAAAATTCCGTTGATACAAACACAAAGGTGTGGGAATGTAAA
AAACCTTATAAATTATCCACTAAAGATGTATGTGTACCTCCGAGGAGGCAAGAATTATGT
CTTGGAAACATTGATAGAATATACGATAAAAACCTATTAATGATAAAAGAGCATATTCTT
GCTATTGCAATATATGAATCAAGAATATTGAAACGAAAATATAAGAATAAAGATGATAA
AGAAGTTTGTAAAATCATAAATAAAACTTTCGCTGATATAAGAGATATTATAGGAGGTAC
TGATTATTGGAATGATTTGAGCAATAGAAAATTAGTAGGAAAAAATTAACACAAATTCAAA
TTATGTTCACAGGAATAAACAAAATGATAAGCTTTTTCGTGATGAGTGGTGGAAAGTTAT
TAAAAAAGATGTATGGAATGTGATATCATGGGTATTCAAGGATAAAACTGTTTGTAAAGA
AGATGATATTGAAAATATACCACAATTCTTCAGATGGTTTAGTGAATGGGGTGATGATTA
TTGCCAGGATAAAACAAAAATG
>Peru Per46_EBA-175KJ419562.1
TATGTATGTATTCCTGATCGTAGAATCCAATTATGCATTGTTAATCTTAGCATTATTAAAA

CATATACAAAAGAGACCATGAAGGATCATTTCATTGAAGCCTCTAAAAAAGAATCTCAA
CTTTTGCTTAAAAAAAATGATAACAAATATAATTCTAAATTTTGTAATGATTTGAAGAATA
GTTTTTTAGATTATGGACATCTTGCTATGGGAAATGATATGGATTTTGGAGGTTATTCAAC
TAAGGCAGAAAACAAAATTCAAGAAGTTTTTAAAGGGGCTCATGGGAAAATAAGTGAAC
ATGAAATTAAAAATTTTAGAAAAAAATGGTGGAATGAATTTAGAGAGAAACTTTGGGAA
GCTATGTTATCTGAGCATAAAAATAATATAAATAATTGTAAAAATATTCCCCAAGAAGAA
TTACAAATTACTCAATGGATAAAAGAATGGCATGGAGAATTTTTGCTTGAAAGAGATAAT
AGATCAAAATTGCCAAAAAGTAAATGTAAAAATAATACATTATATGAAGCATGTGAGAA
GGAATGTATTGATCCATGTATGAAATATAGAGATTGGATTATTAGAAGTAAATTTGAATG
GCATACGTTATCGAAAGAATATGAAACTCAAAAAGTTCCAAAGGAAAATGCGGAAAATT
ATTTAATCAAAATTTCAAAAAACAAGAATGATGCTAAAGTAAGTTTATTATTGAATAATTG
TGATGCTGAATATTCAAAATATTGTGATTGTAAACATACTACTACTCTCGTTAAAAGCGTT
TTAAATGGTAACGACAATACAATTAAGGAAAAGCGTGAACATATTGATTTAGATGATTTT
TCTAAATTTGGATGTGATAAAAATTCCGTTGATACAAACACAAAGGTGTGGGAATGTAAA
AAACCTTATAAATTATCCACTAAAGATGTATGTGTACCTCCGAGGAGGCAAGAATTATGT
CTTGGAAACATTGATAGAATATACGATAAAAACCTATTAATGATAAAAGAGCATATTCTT
GCTATTGCAATATATGAATCAAGAATATTGAAACGAAAATATAAGAATAAAGATGATAA
AGAAGTTTGTAAAATCATAAATAAAACTTTCGCTGATATAAGAGATATTATAGGAGGTAC
TGATTATTGGAATGATTTGAGCAATAGAAAATTAGTAGGAAAAATTAACACAAATTCAAA
TTATGTTCACAGGAATAAACAAAATGATAAGCTTTTTCGTGATGAGTGGTGGAAAGTTAT
TAAAAAAGATGTATGGAATGTGATATCATGGGTATTCAAGGATAAAACTGTTTGTAAAGA
AGATGATATTGAAAATATACCACAATTCTTCAGATGGTTTAGTGAATGGGGTGATGATTA
TTGCCAGGATAAAACAAAAATG
>Peru Per50_EBA-175KJ419563.1
TATGTATGTATTCCTGATCGTAGAATCCAATTATGCATTGTTAATCTTAGCATTATTAAAA
CATATACAAAAGAGACCATGAAGGATCATTTCATTGAAGCCTCTAAAAAAGAATCTCAA
CTTTTGCTTAAAAAAAATGATAACAAATATAATTCTAAATTTTGTAATGATTTGAAGAATA
GTTTTTTAGATTATGGACATCTTGCTATGGGAAATGATATGGATTTTGGAGGTTATTCAAC
TAAGGCAGAAAACAAAATTCAAGAAGTTTTTAAAGGGGCTCATGGGAAAATAAGTGAAC
ATGAAATTAAAAATTTTAGAAAAAAATGGTGGAATGAATTTAGAGAGAAACTTTGGGAA
GCTATGTTATCTGAGCATAAAAATAATATAAATAATTGTAAAAATATTCCCCAAGAAGAA
TTACAAATTACTCAATGGATAAAAGAATGGCATGGAGAATTTTTGCTTGAAAGAGATAAT
AGATCAAAATTGCCAAAAAGTAAATGTAAAAATAATACATTATATGAAGCATGTGAGAA
GGAATGTATTGATCCATGTATGAAATATAGAGATTGGATTATTAGAAGTAAATTTGAATG
GCATACGTTATCGAAAGAATATGAAACTCAAAAAGTTCCAAAGGAAAATGCGGAAAATT
ATTTAATCAAAATTTCAAAAAACAAGAATGATGCTAAAGTAAGTTTATTATTGAATAATTG
TGATGCTGAATATTCAAAATATTGTGATTGTAAACATACTACTACTCTCGTTAAAAGCGTT
TTAAATGGTAACGACAATACAATTAAGGAAAAGCGTGAACATATTGATTTAGATGATTTT
TCTAAATTTGGATGTGATAAAAATTCCGTTGATACAAACACAAAGGTGTGGGAATGTAAA
AAACCTTATAAATTATCCACTAAAGATGTATGTGTACCTCCGAGGAGGCAAGAATTATGT
CTTGGAAACATTGATAGAATATACGATAAAAACCTATTAATGATAAAAGAGCATATTCTT
GCTATTGCAATATATGAATCAAGAATATTGAAACGAAAATATAAGAATAAAGATGATAA
AGAAGTTTGTAAAATCATAAATAAAACTTTCGCTGATATAAGAGATATTATAGGAGGTAC
TGATTATTGGAATGATTTGAGCAATAGAAAATTAGTAGGAAAAATTAACACAAATTCAAA

TTATGTTCACAGGAATAAACAAAATGATAAGCTTTTTCGTGATGAGTGGTGGAAAGTTAT
TAAAAAAGATGTATGGAATGTGATATCATGGGTATTCAAGGATAAAACTGTTTGTAAAGA
AGATGATATTGAAAATATACCACAATTCTTCAGATGGTTTAGTGAATGGGGTGATGATTA
TTGCCAGGATAAAACAAAAATG
>Peru Per52_EBA-175KJ419564.1
TATGTATGTATTCCTGATCGTAGAATCCAATTATGCATTGTTAATCTTAGCATTATTAAAA
CATATACAAAAGAGACCATGAAGGATCATTTCATTGAAGCCTCTAAAAAAGAATCTCAA
CTTTTGCTTAAAAAAAAATGATAACAAATATAATTCTAAATTTTGTAATGATTTGAAGAATA
GTTTTTTAGATTATGGACATCTTGCTATGGGAAATGATATGGATTTTGGAGGTTATTCAAC
TAAGGCAGAAAACAAAATTCAAGAAGTTTTTAAAGGGGCTCATGGGAAAATAAGTGAAC
ATGAAATTAAAAATTTTAGAAAAAAATGGTGGAATGAATTTAGAGAGAAACTTTGGGAA
GCTATGTTATCTGAGCATAAAAATAATATAAATAATTGTAAAAATATTCCCCAAGAAGAA
TTACAAATTACTCAATGGATAAAAGAATGGCATGGAGAATTTTTGCTTGAAAGAGATAAT
AGATCAAAATTGCCAAAAAGTAAATGTAAAAATAATACATTATATGAAGCATGTGAGAA
GGAATGTATTGATCCATGTATGAAATATAGAGATTGGATTATTAGAAGTAAATTTGAATG
GCATACGTTATCGAAAGAATATGAAACTCAAAAAGTTCCAAAGGAAAATGCGGAAAATT
ATTTAATCAAAATTTCAAAAAACAAGAATGATGCTAAAGTAAGTTTATTATTGAATAATTG
TGATGCTGAATATTCAAAATATTGTGATTGTAAACATACTACTACTCTCGTTAAAAGCGTT
TTAAATGGTAACGACAATACAATTAAGGAAAAGCGTGAACATATTGATTTAGATGATTTT
TCTAAATTTGGATGTGATAAAAATTCCGTTGATACAAACACAAAGGTGTGGGAATGTAAA
AAACCTTATAAATTATCCACTAAAGATGTATGTGTACCTCCGAGGAGGCAAGAATTATGT
CTTGGAAACATTGATAGAATATACGATAAAAACCTATTAATGATAAAAGAGCATATTCTT
GCTATTGCAATATATGAATCAAGAATATTGAAACGAAAATATAAGAATAAAGATGATAA
AGAAGTTTGTAAAATCATAAATAAAACTTTCGCTGATATAAGAGATATTATAGGAGGTAC
TGATTATTGGAATGATTTGAGCAATAGAAAATTAGTAGGAAAAATTAACACAAATTCAAA
TTATGTTCACAGGAATAAACAAAATGATAAGCTTTTTCGTGATGAGTGGTGGAAAGTTAT
TAAAAAAGATGTATGGAATGTGATATCATGGGTATTCAAGGATAAAACTGTTTGTAAAGA
AGATGATATTGAAAATATACCACAATTCTTCAGATGGTTTAGTGAATGGGGTGATGATTA
TTGCCAGGATAAAACAAAAATG
>Peru Per59_EBA-175KJ419565.1
TATGTATGTATTCCTGATCGTAGAATCCAATTATGCATTGTTAATCTTAGCATTATTAAAA
CATATACAAAAGAGACCATGAAGGATCATTTCATTGAAGCCTCTAAAAAAGAATCTCAA
CTTTTGCTTAAAAAAAAATGATAACAAATATAATTCTAAATTTTGTAATGATTTGAAGAATA
GTTTTTTAGATTATGGACATCTTGCTATGGGAAATGATATGGATTTTGGAGGTTATTCAAC
TAAGGCAGAAAACAAAATTCAAGAAGTTTTTAAAGGGGCTCATGGGAAAATAAGTGAAC
ATGAAATTAAAAATTTTAGAAAAAAATGGTGGAATGAATTTAGAGAGAAACTTTGGGAA
GCTATGTTATCTGAGCATAAAAATAATATAAATAATTGTAAAAATATTCCCCAAGAAGAA
TTACAAATTACTCAATGGATAAAAGAATGGCATGGAGAATTTTTGCTTGAAAGAGATAAT
AGATCAAAATTGCCAAAAAGTAAATGTAAAAATAATACATTATATGAAGCATGTGAGAA
GGAATGTATTGATCCATGTATGAAATATAGAGATTGGATTATTAGAAGTAAATTTGAATG
GCATACGTTATCGAAAGAATATGAAACTCAAAAAGTTCCAAAGGAAAATGCGGAAAATT
ATTTAATCAAAATTTCAAAAAACAAGAATGATGCTAAAGTAAGTTTATTATTGAATAATTG
TGATGCTGAATATTCAAAATATTGTGATTGTAAACATACTACTACTCTCGTTAAAAGCGTT
TTAAATGGTAACGACAATACAATTAAGGAAAAGCGTGAACATATTGATTTAGATGATTTT

TCTAAATTTGGATGTGATAAAAATTCCGTTGATACAAACACAAAGGTGTGGGAATGTAAA
AAACCTTATAAATTATCCACTAAAGATGTATGTGTACCTCCGAGGAGGCAAGAATTATGT
CTTGGAAACATTGATAGAATATACGATAAAAACCTATTAATGATAAAAGAGCATATTCTT
GCTATTGCAATATATGAATCAAGAATATTGAAACGAAAATATAAGAATAAAGATGATAA
AGAAGTTTGTAAAATCATAAATAAAACTTTCGCTGATATAAGAGATATTATAGGAGGTAC
TGATTATTGGAATGATTTGAGCAATAGAAAATTAGTAGGAAAAATTAACACAAATTCAAA
TTATGTTCACAGGAATAAACAAAATGATAAGCTTTTTCGTGATGAGTGGTGGAAAGTTAT
TAAAAAAGATGTATGGAATGTGATATCATGGGTATTCAAGGATAAAACTGTTTGTAAAGA
AGATGATATTGAAAATATACCACAATTCTTCAGATGGTTTAGTGAATGGGGTGATGATTA
TTGCCAGGATAAAACAAAAATG
>Peru Per55_EBA-175KJ419566.1
TATGTATGTATTCCTGATCGTAGAATCCAATTATGCATTGTTAATCTTAGCATTATTAAAA
CATATACAAAAGAGACCATGAAGGATCATTTCATTGAAGCCTCTAAAAAAGAATCTCAA
CTTTTGCTTAAAAAAAAATGATAACAAATATAATTCTAAATTTTGTAATGATTTGAAGAATA
GTTTTTTAGATTATGGACATCTTGCTATGGGAAATGATATGGATTTTGGAGGTTATTCAAC
TAAGGCAGAAAACAAAATTCAAGAAGTTTTTAAAGGGGCTCATGGGAAAATAAGTGAAC
ATGAAATTAAAAATTTTAGAAAAAAATGGTGGAATGAATTTAGAGAGAAACTTTGGGAA
GCTATGTTATCTGAGCATAAAAATAATATAAATAATTGTAAAAATATTCCCCAAGAAGAA
TTACAAATTACTCAATGGATAAAAGAATGGCATGGAGAATTTTTGCTTGAAAGAGATAAT
AGATCAAAATTGCCAAAAAGTAAATGTAAAAATAATACATTATATGAAGCATGTGAGAA
GGAATGTATTGATCCATGTATGAAATATAGAGATTGGATTATTAGAAGTAAATTTGAATG
GCATACGTTATCGAAAGAATATGAAACTCAAAAAGTTCCAAAGGAAAATGCGGAAAATT
ATTTAATCAAAATTTCAAAAAACAAGAATGATGCTAAAGTAAGTTTATTATTGAATAATTG
TGATGCTGAATATTCAAAATATTGTGATTGTAAACATACTACTACTCTCGTTAAAAGCGTT
TTAAATGGTAACGACAATACAATTAAGGAAAAGCGTGAACATATTGATTTAGATGATTTT
TCTAAATTTGGATGTGATAAAAATTCCGTTGATACAAACACAAAGGTGTGGGAATGTAAA
AAACCTTATAAATTATCCACTAAAGATGTATGTGTACCTCCGAGGAGGCAAGAATTATGT
CTTGGAAACATTGATAGAATATACGATAAAAACCTATTAATGATAAAAGAGCATATTCTT
GCTATTGCAATATATGAATCAAGAATATTGAAACGAAAATATAAGAATAAAGATGATAA
AGAAGTTTGTAAAATCATAAATAAAACTTTCGCTGATATAAGAGATATTATAGGAGGTAC
TGATTATTGGAATGATTTGAGCAATAGAAAATTAGTAGGAAAAATTAACACAAATTCAAA
TTATGTTCACAGGAATAAACAAAATGATAAGCTTTTTCGTGATGAGTGGTGGAAAGTTAT
TAAAAAAGATGTATGGAATGTGATATCATGGGTATTCAAGGATAAAACTGTTTGTAAAGA
AGATGATATTGAAAATATACCACAATTCTTCAGATGGTTTAGTGAATGGGGTGATGATTA
TTGCCAGGATAAAACAAAAATG
>Peru Per56_EBA-175KJ419567.1
TATGTATGTATTCCTGATCGTAGAATCCAATTATGCATTGTTAATCTTAGCATTATTAAAA
CATATACAAAAGAGACCATGAAGGATCATTTCATTGAAGCCTCTAAAAAAGAATCTCAA
CTTTTGCTTAAAAAAAAATGATAACAAATATAATTCTAAATTTTGTAATGATTTGAAGAATA
GTTTTTTAGATTATGGACATCTTGCTATGGGAAATGATATGGATTTTGGAGGTTATTCAAC
TAAGGCAGAAAACAAAATTCAAGAAGTTTTTAAAGGGGCTCATGGGAAAATAAGTGAAC
ATGAAATTAAAAATTTTAGAAAAAAATGGTGGAATGAATTTAGAGAGAAACTTTGGGAA
GCTATGTTATCTGAGCATAAAAATAATATAAATAATTGTAAAAATATTCCCCAAGAAGAA
TTACAAATTACTCAATGGATAAAAGAATGGCATGGAGAATTTTTGCTTGAAAGAGATAAT

AGATCAAAATTGCCAAAAAGTAAATGTAAAAATAATACATTATATGAAGCATGTGAGAA
GGAATGTATTGATCCATGTATGAAATATAGAGATTGGATTATTAGAAGTAAATTTGAATG
GCATACGTTATCGAAAGAATATGAAACTCAAAAAGTTCCAAAGGAAAATGCGGAAAATT
ATTTAATCAAAATTTCAAAAAACAAGAATGATGCTAAAGTAAGTTTATTATTGAATAATTG
TGATGCTGAATATTCAAAATATTGTGATTGTAAACATACTACTACTCTCGTTAAAAGCGTT
TTAAATGGTAACGACAATACAATTAAGGAAAAGCGTGAACATATTGATTTAGATGATTTT
TCTAAATTTGGATGTGATAAAAATTCCGTTGATACAAACACAAAGGTGTGGGAATGTAAA
AAACCTTATAAATTATCCACTAAAGATGTATGTGTACCTCCGAGGAGGCAAGAATTATGT
CTTGGAAACATTGATAGAATATACGATAAAAACCTATTAATGATAAAAGAGCATATTCTT
GCTATTGCAATATATGAATCAAGAATATTGAAACGAAAATATAAGAATAAAGATGATAA
AGAAGTTTGTAAAATCATAAATAAAACTTTCGCTGATATAAGAGATATTATAGGAGGTAC
TGATTATTGGAATGATTTGAGCAATAGAAAATTAGTAGGAAAAATTAACACAAATTCAAA
TTATGTTCACAGGAATAAACAAAATGATAAGCTTTTTCGTGATGAGTGGTGGAAAGTTAT
TAAAAAAGATGTATGGAATGTGATATCATGGGTATTCAAGGATAAAACTGTTTGTAAAGA
AGATGATATTGAAAATATACCACAATTCTTCAGATGGTTTAGTGAATGGGGTGATGATTA
TTGCCAGGATAAAACAAAAATG
>Peru Per57_EBA-175KJ419568.1
TATGTATGTATTCCTGATCGTAGAATCCAATTATGCATTGTTAATCTTAGCATTATTAAAA
CATATACAAAAGAGACCATGAAGGATCATTTCATTGAAGCCTCTAAAAAAGAATCTCAA
CTTTTGCTTAAAAAAAATGATAACAAATATAATTCTAAATTTTGTAATGATTTGAAGAATA
GTTTTTTAGATTATGGACATCTTGCTATGGGAAATGATATGGATTTTGGAGGTTATTCAAC
TAAGGCAGAAAACAAAATTCAAGAAGTTTTTAAAGGGGCTCATGGGGAAATAAGTGAAC
ATAAAATTAAAAATTTTAGAAAAGAATGGTGGAATGAATTTAGAGAGAAACTTTGGGAA
GCTATGTTATCTGAGCATAAAAATAATATAAATAATTGTAAAAATATTCCCCAAGAAGAA
TTACAAATTACTCAATGGATAAAAGAATGGCATGGAGAATTTTTGCTTGAAAGAGATAAT
AGATCAAAATTGCCAAAAAGTAAATGTAAAAATAATACATTATATGAAGCATGTGAGAA
GGAATGTATTGATCCATGTATGAAATATAGAGATTGGATTATTAGAAGTAAATTTGAATG
GCATACGTTATCGAAAGAATATGAAACTCAAAAAGTTCCAAAGGAAAATGCGGAAAATT
ATTTAATCAAAATTTCAGAAAACAAGAATGATGCTAAAGTAAGTTTATTATTGAATAATT
GTGATGCTGAATATTCAAAATATTGTGATTGTAAACATACTACTACTCTCGTTAAAAGCGT
TTTAAATGGTAACGACAATACAATTAAGGAAAAGCGTGAACATATTGATTTAGATGATTT
TTCTAAATTTGGATGTGATAAAAATTCCGTTGATACAAACACAAAGGTGTGGGAATGTAA
AAAACCTTATAAATTATCCACTAAAGATGTATGTGTACCTCCGAGGAGGCAAGAATTATG
TCTTGGAAACATTGATAGAATATACGATAAAAACCTATTAATGATAAAAGAGCATATTCT
TGCTATTGCAATATATGAATCAAGAATATTGAAACGAAAATATAAGAATAAAGATGATAA
AGAAGTTTGTAAAATCATAAATAAAACTTTCGCTGATATAAGAGATATTATAGGAGGTAC
TGATTATTGGAATGATTTGAGCAATAGAAAATTAGTAGGAAAAATTAACACAAATTCAAA
TTATGTTCACAGGAATAAACAAAATGATAAGCTTTTTCGTGATGAGTGGTGGAAAGTTAT
TAAAAAAGATGTATGGAATGTGATATCATGGGTATTCAAGGATAAAACTGTTTGTAAAGA
AGATGATATTGAAAATATACCACAATTCTTCAGATGGTTTAGTGAATGGGGTGATGATTA
TTGCCAGGATAAAACAAAAATG
>Peru Per58_EBA-175KJ419569.1
TATGTATGTATTCCTGATCGTAGAATCCAATTATGCATTGTTAATCTTAGCATTATTAAAA
CATATACAAAAGAGACCATGAAGGATCATTTCATTGAAGCCTCTAAAAAAGAATCTCAA

CTTTTGCTTAAAAAAAAATGATAACAAATATAATTCTAAATTTTGTAATGATTTGAAGAATA
GTTTTTTAGATTATGGACATCTTGCTATGGGAAATGATATGGATTTTGGAGGTTATTCAAC
TAAGGCAGAAAACAAAATTCAAGAAGTTTTTAAAGGGGCTCATGGGAAAATAAGTGAAC
ATGAAATTAAAAATTTTAGAAAAAAAATGGTGGAATGAATTTAGAGAGAAACTTTGGGAA
GCTATGTTATCTGAGCATAAAAATAATATAAATAATTGTAAAAATATTCCCCAAGAAGAA
TTACAAATTACTCAATGGATAAAAGAATGGCATGGAGAATTTTTGCTTGAAAGAGATAAT
AGATCAAAATTGCCAAAAAGTAAATGTAAAAATAATACATTATATGAAGCATGTGAGAA
GGAATGTATTGATCCATGTATGAAATATAGAGATTGGATTATTAGAAGTAAATTTGAATG
GCATACGTTATCGAAAGAATATGAAACTCAAAAAGTTCCAAAGGAAAATGCGGAAAATT
ATTTAATCAAAATTTCAAAAAACAAGAATGATGCTAAAGTAAGTTTATTATTGAATAATTG
TGATGCTGAATATTCAAAATATTGTGATTGTAAACATACTACTACTCTCGTTAAAAGCGTT
TTAAATGGTAACGACAATACAATTAAGGAAAAGCGTGAACATATTGATTTAGATGATTTT
TCTAAATTTGGATGTGATAAAAATTCCGTTGATACAAACACAAAGGTGTGGGAATGTAAA
AAACCTTATAAATTATCCACTAAAGATGTATGTGTACCTCCGAGGAGGCAAGAATTATGT
CTTGGAAACATTGATAGAATATACGATAAAAACCTATTAATGATAAAAGAGCATATTCTT
GCTATTGCAATATATGAATCAAGAATATTGAAACGAAAATATAAGAATAAAGATGATAA
AGAAGTTTGTAAAATCATAAATAAAACTTTCGCTGATATAAGAGATATTATAGGAGGTAC
TGATTATTGGAATGATTTGAGCAATAGAAAATTAGTAGGAAAAATTAACACAAATTCAAA
TTATGTTCACAGGAATAAACAAAATGATAAGCTTTTTCGTGATGAGTGGTGGAAAGTTAT
TAAAAAAGATGTATGGAATGTGATATCATGGGTATTCAAGGATAAAACTGTTTGTAAAGA
AGATGATATTGAAAATATACCACAATTCTTCAGATGGTTTAGTGAATGGGGTGATGATTA
TTGCCAGGATAAAACAAAAATG
>Peru Per62_EBA-175KJ419570.1
TATGTATGTATTCCTGATCGTAGAATCCAATTATGCATTGTTAATCTTAGCATTATTAAAA
CATATACAAAAGAGACCATGAAGGATCATTTCATTGAAGCCTCTAAAAAAGAATCTCAA
CTTTTGCTTAAAAAAAAATGATAACAAATATAATTCTAAATTTTGTAATGATTTGAAGAATA
GTTTTTTAGATTATGGACATCTTGCTATGGGAAATGATATGGATTTTGGAGGTTATTCAAC
TAAGGCAGAAAACAAAATTCAAGAAGTTTTTAAAGGGGCTCATGGGGAAATAAGTGAAC
ATAAAATTAAAAATTTTAGAAAAGAATGGTGGAATGAATTTAGAGAGAAACTTTGGGAA
GCTATGTTATCTGAGCATAAAAATAATATAAATAATTGTAAAAATATTCCCCAAGAAGAA
TTACAAATTACTCAATGGATAAAAGAATGGCATGGAGAATTTTTGCTTGAAAGAGATAAT
AGATCAAAATTGCCAAAAAGTAAATGTAAAAATAATACATTATATGAAGCATGTGAGAA
GGAATGTATTGATCCATGTATGAAATATAGAGATTGGATTATTAGAAGTAAATTTGAATG
GCATACGTTATCGAAAGAATATGAAACTCAAAAAGTTCCAAAGGAAAATGCGGAAAATT
ATTTAATCAAAATTTCAGAAAACAAGAATGATGCTAAAGTAAGTTTATTATTGAATAATT
GTGATGCTGAATATTCAAAATATTGTGATTGTAAACATACTACTACTCTCGTTAAAAGCGT
TTTAAATGGTAACGACAATACAATTAAGGAAAAGCGTGAACATATTGATTTAGATGATTT
TTCTAAATTTGGATGTGATAAAAATTCCGTTGATACAAACACAAAGGTGTGGGAATGTAA
AAAACCTTATAAATTATCCACTAAAGATGTATGTGTACCTCCGAGGAGGCAAGAATTATG
TCTTGGAAACATTGATAGAATATACGATAAAAACCTATTAATGATAAAAGAGCATATTCT
TGCTATTGCAATATATGAATCAAGAATATTGAAACGAAAATATAAGAATAAAGATGATAA
AGAAGTTTGTAAAATCATAAATAAAACTTTCGCTGATATAAGAGATATTATAGGAGGTAC
TGATTATTGGAATGATTTGAGCAATAGAAAATTAGTAGGAAAAATTAACACAAATTCAAA
TTATGTTCACAGGAATAAACAAAATGATAAGCTTTTTCGTGATGAGTGGTGGAAAGTTAT

TAAAAAAGATGTATGGAATGTGATATCATGGGTATTCAAGGATAAAACTGTTTGTAAAGA
AGATGATATTGAAAATATACCACAATTCTTCAGATGGTTTAGTGAATGGGGTGATGATTA
TTGCCAGGATAAAACAAAAATG
>Peru Per64_EBA-175KJ419571.1
TATGTATGTATTCCTGATCGTAGAATCCAATTATGCATTGTTAATCTTAGCATTATTAAAA
CATATACAAAAGAGACCATGAAGGATCATTTCATTGAAGCCTCTAAAAAAGAATCTCAA
CTTTTGCTTAAAAAAAAATGATAACAAATATAATTCTAAATTTTGTAATGATTTGAAGAATA
GTTTTTTAGATTATGGACATCTTGCTATGGGAAATGATATGGATTTTGGAGGTTATTCAAC
TAAGGCAGAAAACAAAATTCAAGAAGTTTTTAAAGGGGCTCATGGGGAAATAAGTGAAC
ATGAAATTAAAAATTTTAGAAAAAAATGGTGGAATGAATTTAGAGAGAAACTTTGGGAA
GCTATGTTATCTGAGCATAAAAATAATATAAATAATTGTAAAAATATTCCCCAAGAAGAA
TTACAAATTACTCAATGGATAAAAGAATGGCATGGAGAATTTTTGCTTGAAAGAGATAAT
AGATCAAAATTGCCAAAAAGTAAATGTAAAAATAATACATTATATGAAGCATGTGAGAA
GGAATGTATTGATCCATGTATGAAATATAGAGATTGGATTATTAGAAGTAAATTTGAATG
GCATACGTTATCGAAAGAATATGAAACTCAAAAAGTTTCAAAGGAAAATGCGGAAAATT
ATTTAATCAAAATTTCAGAAAACAAGAATGATGCTAAAGTAAGTTTATTATTGAATAATT
GTGATGCTGAATATTCAAAATATTGTGATTGTAAACATACTACTACTCTCGTTAAAAGCGT
TTTAAATGGTAACGACAATACAATTAAGGAAAAGCGTGAACATATTGATTTAGATGATTT
TTCTAAATTTGGATGTGATAAAAATTCCGTTGATACAAACACAAAGGTGTGGGAATGTAA
AAAACCTTATATATTATCCACTAAAGATGTATGTGTACCTCCGAGGAGGCAAGAATTATG
TCTTGGAAACATTGATAGAATATACGATAAAAACCTATTAATGATAAAAGAGCATATTCT
TGCTATTGCAATATATGAATCAAGAATATTGAAACGAAAATATAAGAATAAAGATGATAA
AGAAGTTTGTAAAATCATAAATAAAACTTTCGCTGATATAAGAGATATTATAGGAGGTAC
TGATTATTGGAATGATTTGAGCAATAGAAAATTAGTAGGAAAAATTAACACAAATTCAAA
ATATGTTCACAGGAATAAAAAAAATGATAAGCTTTTTCGTGATGAGTGGTGGAAAGTTAT
TAAAAAAGATGTATGGAATGTGATATCATGGGTATTCAAGGATAAAACTGTTTGTAAAGA
AGATGATATTGAAAATATACCACAATTCTTCAGATGGTTTAGTGAATGGGGTGATGATTA
TTGCCAGGATAAAACAAAAATG
>Peru Per65_EBA-175KJ419572.1
TATGTATGTATTCCTGATCGTAGAATCCAATTATGCATTGTTAATCTTAGCATTATTAAAA
CATATACAAAAGAGACCATGAAGGATCATTTCATTGAAGCCTCTAAAAAAGAATCTCAA
CTTTTGCTTAAAAAAAAATGATAACAAATATAATTCTAAATTTTGTAATGATTTGAAGAATA
GTTTTTTAGATTATGGACATCTTGCTATGGGAAATGATATGGATTTTGGAGGTTATTCAAC
TAAGGCAGAAAACAAAATTCAAGAAGTTTTTAAAGGGGCTCATGGGGAAATAAGTGAAC
ATGAAATTAAAAATTTTAGAAAAAAATGGTGGAATGAATTTAGAGAGAAACTTTGGGAA
GCTATGTTATCTGAGCATAAAAATAATATAAATAATTGTAAAAATATTCCCCAAGAAGAA
TTACAAATTACTCAATGGATAAAAGAATGGCATGGAGAATTTTTGCTTGAAAGAGATAAT
AGATCAAAATTGCCAAAAAGTAAATGTAAAAATAATACATTATATGAAGCATGTGAGAA
GGAATGTATTGATCCATGTATGAAATATAGAGATTGGATTATTAGAAGTAAATTTGAATG
GCATACGTTATCGAAAGAATATGAAACTCAAAAAGTTCCAAAGGAAAATGCGGAAAATT
ATTTAATCAAAATTTCAAAAAACAAGAATGATGCTAAAGTAAGTTTATTATTGAATAATTG
TGATGCTGAATATTCAAAATATTGTGATTGTAAACATACTACTACTCTCGTTAAAAGCGTT
TTAAATGGTAACGACAATACAATTAAGGAAAAGCGTGAACATATTGATTTAGATGATTTT
TCTAAATTTGGATGTGATAAAAATTCCGTTGATACAAACACAAAGGTGTGGGAATGTAAA

AAACCTTATAAATTATCCACTAAAGATGTATGTGTACCTCCGAGGAGGCAAGAATTATGT
CTTGGAAACATTGATAGAATATACGATAAAAACCTATTAATGATAAAAGAGCATATTCTT
GCTATTGCAATATATGAATCAAGAATATTGAAACGAAAATATAAGAATAAAGATGATAA
AGAAGTTTGTAAAATCATAAATAAAACTTTCGCTGATATAAGAGATATTATAGGAGGTAC
TGATTATTGGAATGATTTGAGCAATAGAAAATTAGTAGGAAAAATTAACACAAATTCAAA
TTATGTTCACAGGAATAAACAAAATGATAAGCTTTTTCGTGATGAGTGGTGGAAAGTTAT
TAAAAAAGATGTATGGAATGTGATATCATGGGTATTCAAGGATAAAACTGTTTGTAAAGA
AGATGATATTGAAAATATACCACAATTCTTCAGATGGTTTAGTGAATGGGGTGATGATTA
TTGCCAGGATAAAACAAAAATG
>Peru Per66_EBA-175KJ419573.1
TATGTATGTATTCCTGATCGTAGAATCCAATTATGCATTGTTAATCTTAGCATTATTAAAA
CATATACAAAAGAGACCATGAAGGATCATTTCATTGAAGCCTCTAAAAAAGAATCTCAA
CTTTTGCTTAAAAAAAAATGATAACAAATATAATTCTAAATTTTGTAATGATTTGAAGAATA
GTTTTTTAGATTATGGACATCTTGCTATGGGAAATGATATGGATTTTGGAGGTTATTCAAC
TAAGGCAGAAAACAAAATTCAAGAAGTTTTTAAAGGGGCTCATGGGAAAATAAGTGAAC
ATGAAATTAAAAATTTTAGAAAAAAATGGTGGAATGAATTTAGAGAGAAACTTTGGGAA
GCTATGTTATCTGAGCATAAAAATAATATAAATAATTGTAAAAATATTCCCCAAGAAGAA
TTACAAATTACTCAATGGATAAAAGAATGGCATGGAGAATTTTTGCTTGAAAGAGATAAT
AGATCAAAATTGCCAAAAAGTAAATGTAAAAATAATACATTATATGAAGCATGTGAGAA
GGAATGTATTGATCCATGTATGAAATATAGAGATTGGATTATTAGAAGTAAATTTGAATG
GCATACGTTATCGAAAGAATATGAAACTCAAAAAGTTCCAAAGGAAAATGCGGAAAATT
ATTTAATCAAAATTTCAAAAAACAAGAATGATGCTAAAGTAAGTTTATTATTGAATAATTG
TGATGCTGAATATTCAAAATATTGTGATTGTAAACATACTACTACTCTCGTTAAAAGCGTT
TTAAATGGTAACGACAATACAATTAAGGAAAAGCGTGAACATATTGATTTAGATGATTTT
TCTAAATTTGGATGTGATAAAAATTCCGTTGATACAAACACAAAGGTGTGGGAATGTAAA
AAACCTTATAAATTATCCACTAAAGATGTATGTGTACCTCCGAGGAGGCAAGAATTATGT
CTTGGAAACATTGATAGAATATACGATAAAAACCTATTAATGATAAAAGAGCATATTCTT
GCTATTGCAATATATGAATCAAGAATATTGAAACGAAAATATAAGAATAAAGATGATAA
AGAAGTTTGTAAAATCATAAATAAAACTTTCGCTGATATAAGAGATATTATAGGAGGTAC
TGATTATTGGAATGATTTGAGCAATAGAAAATTAGTAGGAAAAATTAACACAAATTCAAA
TTATGTTCACAGGAATAAACAAAATGATAAGCTTTTTCGTGATGAGTGGTGGAAAGTTAT
TAAAAAAGATGTATGGAATGTGATATCATGGGTATTCAAGGATAAAACTGTTTGTAAAGA
AGATGATATTGAAAATATACCACAATTCTTCAGATGGTTTAGTGAATGGGGTGATGATTA
TTGCCAGGATAAAACAAAAATG
>Peru Per68_EBA-175KJ419574.1
TATGTATGTATTCCTGATCGTAGAATCCAATTATGCATTGTTAATCTTAGCATTATTAAAA
CATATACAAAAGAGACCATGAAGGATCATTTCATTGAAGCCTCTAAAAAAGAATCTCAA
CTTTTGCTTAAAAAAAAATGATAACAAATATAATTCTAAATTTTGTAATGATTTGAAGAATA
GTTTTTTAGATTATGGACATCTTGCTATGGGAAATGATATGGATTTTGGAGGTTATTCAAC
TAAGGCAGAAAACAAAATTCAAGAAGTTTTTAAAGGGGCTCATGGGAAAATAAGTGAAC
ATGAAATTAAAAATTTTAGAAAAAAATGGTGGAATGAATTTAGAGAGAAACTTTGGGAA
GCTATGTTATCTGAGCATAAAAATAATATAAATAATTGTAAAAATATTCCCCAAGAAGAA
TTACAAATTACTCAATGGATAAAAGAATGGCATGGAGAATTTTTGCTTGAAAGAGATAAT
AGATCAAAATTGCCAAAAAGTAAATGTAAAAATAATACATTATATGAAGCATGTGAGAA

GGAATGTATTGATCCATGTATGAAATATAGAGATTGGATTATTAGAAGTAAATTTGAATG
GCATACGTTATCGAAAGAATATGAAACTCAAAAAGTTCCAAAGGAAAATGCGGAAAATT
ATTTAATCAAAATTTCAAAAAACAAGAATGATGCTAAAGTAAGTTTATTATTGAATAATTG
TGATGCTGAATATTCAAAATATTGTGATTGTAAACATACTACTACTCTCGTTAAAAGCGTT
TTAAATGGTAACGACAATACAATTAAGGAAAAGCGTGAACATATTGATTTAGATGATTTT
TCTAAATTTGGATGTGATAAAAATTCCGTTGATACAAACACAAAGGTGTGGGAATGTAAA
AAACCTTATAAATTATCCACTAAAGATGTATGTGTACCTCCGAGGAGGCAAGAATTATGT
CTTGGAAACATTGATAGAATATACGATAAAAACCTATTAATGATAAAAGAGCATATTCTT
GCTATTGCAATATATGAATCAAGAATATTGAAACGAAAATATAAGAATAAAGATGATAA
AGAAGTTTGTAAAATCATAAATAAAACTTTCGCTGATATAAGAGATATTATAGGAGGTAC
TGATTATTGGAATGATTTGAGCAATAGAAAATTAGTAGGAAAAATTAACACAAATTCAAA
TTATGTTCACAGGAATAAACAAAATGATAAGCTTTTTCGTGATGAGTGGTGGAAAGTTAT
TAAAAAAGATGTATGGAATGTGATATCATGGGTATTCAAGGATAAAACTGTTTGTAAAGA
AGATGATATTGAAAATATACCACAATTCTTCAGATGGTTTAGTGAATGGGGTGATGATTA
TTGCCAGGATAAAACAAAAATG
>Peru Per69_EBA-175KJ419575.1
TATGTATGTATTCCTGATCGTAGAATCCAATTATGCATTGTTAATCTTAGCATTATTAAAA
CATATACAAAAGAGACCATGAAGGATCATTTCATTGAAGCCTCTAAAAAAGAATCTCAA
CTTTTGCTTAAAAAAAAATGATAACAAATATAATTCTAAATTTTGTAATGATTTGAAGAATA
GTTTTTTAGATTATGGACATCTTGCTATGGGAAATGATATGGATTTTGGAGGTTATTCAAC
TAAGGCAGAAAACAAAATTCAAGAAGTTTTTAAAGGGGCTCATGGGAAAATAAGTGAAC
ATGAAATTAAAAATTTTAGAAAAAAATGGTGGAATGAATTTAGAGAGAAACTTTGGGAA
GCTATGTTATCTGAGCATAAAAATAATATAAATAATTGTAAAAATATTCCCCAAGAAGAA
TTACAAATTACTCAATGGATAAAAGAATGGCATGGAGAATTTTTGCTTGAAAGAGATAAT
AGATCAAAATTGCCAAAAAGTAAATGTAAAAATAATACATTATATGAAGCATGTGAGAA
GGAATGTATTGATCCATGTATGAAATATAGAGATTGGATTATTAGAAGTAAATTTGAATG
GCATACGTTATCGAAAGAATATGAAACTCAAAAAGTTCCAAAGGAAAATGCGGAAAATT
ATTTAATCAAAATTTCAAAAAACAAGAATGATGCTAAAGTAAGTTTATTATTGAATAATTG
TGATGCTGAATATTCAAAATATTGTGATTGTAAACATACTACTACTCTCGTTAAAAGCGTT
TTAAATGGTAACGACAATACAATTAAGGAAAAGCGTGAACATATTGATTTAGATGATTTT
TCTAAATTTGGATGTGATAAAAATTCCGTTGATACAAACACAAAGGTGTGGGAATGTAAA
AAACCTTATAAATTATCCACTAAAGATGTATGTGTACCTCCGAGGAGGCAAGAATTATGT
CTTGGAAACATTGATAGAATATACGATAAAAACCTATTAATGATAAAAGAGCATATTCTT
GCTATTGCAATATATGAATCAAGAATATTGAAACGAAAATATAAGAATAAAGATGATAA
AGAAGTTTGTAAAATCATAAATAAAACTTTCGCTGATATAAGAGATATTATAGGAGGTAC
TGATTATTGGAATGATTTGAGCAATAGAAAATTAGTAGGAAAAATTAACACAAATTCAAA
TTATGTTCACAGGAATAAACAAAATGATAAGCTTTTTCGTGATGAGTGGTGGAAAGTTAT
TAAAAAAGATGTATGGAATGTGATATCATGGGTATTCAAGGATAAAACTGTTTGTAAAGA
AGATGATATTGAAAATATACCACAATTCTTCAGATGGTTTAGTGAATGGGGTGATGATTA
TTGCCAGGATAAAACAAAAATG
>Peru Per70_EBA-175KJ419576.1
TATGTATGTATTCCTGATCGTAGAATCCAATTATGCATTGTTAATCTTAGCATTATTAAAA
CATATACAAAAGAGACCATGAAGGATCATTTCATTGAAGCCTCTAAAAAAGAATCTCAA
CTTTTGCTTAAAAAAAAATGATAACAAATATAATTCTAAATTTTGTAATGATTTGAAGAATA

GTTTTTTAGATTATGGACATCTTGCTATGGGAAATGATATGGATTTTGGAGGTTATTCAAC
TAAGGCAGAAAACAAAATTCAAGAAGTTTTTAAAGGGGCTCATGGGAAAATAAGTGAAC
ATGAAATTAAAAATTTTAGAAAAAAATGGTGGAATGAATTTAGAGAGAAACTTTGGGAA
GCTATGTTATCTGAGCATAAAAATAATATAAATAATTGTAAAAATATTCCCCAAGAAGAA
TTACAAATTACTCAATGGATAAAAGAATGGCATGGAGAATTTTTGCTTGAAAGAGATAAT
AGATCAAAATTGCCAAAAAGTAAATGTAAAAATAATACATTATATGAAGCATGTGAGAA
GGAATGTATTGATCCATGTATGAAATATAGAGATTGGATTATTAGAAGTAAATTTGAATG
GCATACGTTATCGAAAGAATATGAAACTCAAAAAGTTCCAAAGGAAAATGCGGAAAATT
ATTTAATCAAAATTTCAAAAAACAAGAATGATGCTAAAGTAAGTTTATTATTGAATAATTG
TGATGCTGAATATTCAAAATATTGTGATTGTAAACATACTACTACTCTCGTTAAAAGCGTT
TTAAATGGTAACGACAATACAATTAAGGAAAAGCGTGAACATATTGATTTAGATGATTTT
TCTAAATTTGGATGTGATAAAAATTCCGTTGATACAAACACAAAGGTGTGGGAATGTAAA
AAACCTTATAAATTATCCACTAAAGATGTATGTGTACCTCCGAGGAGGCAAGAATTATGT
CTTGGAAACATTGATAGAATATACGATAAAAACCTATTAATGATAAAAGAGCATATTCTT
GCTATTGCAATATATGAATCAAGAATATTGAAACGAAAATATAAGAATAAAGATGATAA
AGAAGTTTGTAAAATCATAAATAAAACTTTCGCTGATATAAGAGATATTATAGGAGGTAC
TGATTATTGGAATGATTTGAGCAATAGAAAATTAGTAGGAAAAATTAACACAAATTCAAA
TTATGTTCACAGGAATAAACAAAATGATAAGCTTTTTCGTGATGAGTGGTGGAAAGTTAT
TAAAAAAGATGTATGGAATGTGATATCATGGGTATTCAAGGATAAAACTGTTTGTAAAGA
AGATGATATTGAAAATATACCACAATTCTTCAGATGGTTTAGTGAATGGGGTGATGATTA
TTGCCAGGATAAAACAAAAATG
>Venezuela Ven2_EBA-175KJ419577.1
TATGTATGTATTCCTGATCGTAGAATCCAATTATGCATTGTTAATCTTAGCATTATTAAAA
CATATACAAAAGAGACCATGAAGGATCATTTCATTGAAGCCTCTAAAAAAGAATCTCAA
CTTTTGCTTAAAAAAAATGATAACGAATATAATTCTAAATTTTGTAATGATTTGAAGAATA
GTTTTTTAGATTATGGACATCTTGCTATGGGAAATGATATGGATTTTGGAGGTTATTCAAC
TAAGGCAGAAAACAAAATTCAAGAAGTTTTTAAAGGGGCTCATGGGGAAATAAGTGAAC
ATAAAATTAAAAATTTTAGAAAAGAATGGTGGAATGAATTTAGAGAGAAACTTTGGGAA
GCTATGTTATCTGAGCATAAAAATAATATAAATAATTGTAAAAATATTCCCCAAGAAGAA
TTACAAATTACTCAATGGATAAAAGAATGGCATGGAGAATTTTTGCTTGAAAGATATAAT
AGATCAAAATTGCCAAAAAGTAAATGTAAAAATAATACATTATATGAAGCATGTGAGAA
GGAATGTATTGATCCATGTATGAAATATAGAGATTGGATTATTAGAAGTAAATTTGAATG
GCATACGTTATCGAAAGAATATGAAACTCAAAAAGTTTCAAAGGAAAATGCGGAAAATT
ATTTAATCAAAATTTCAGAAAACAAGAATGATGCTAAAGTAAGTTTATTATTGAATAATT
GTGATGCTGAATATTCAAAATATTGTGATTGTAAACATACTACTACTCTCGTTAAAAGCGT
TTTAAATGGTAACGACAATACAATTAAGGAAAAGCGTGAACATATTGATTTAGATGATTT
TTCTAAATTTGGATGTGATAAAAATTCCGTTGATACAAACACAAAGGTGTGGGAATGTAA
AAAACCTTATATATTATCCACTAAAGATGTATGTGTACCTCCGAGGAGGCAAGAATTATG
TCTTGGAAACATTGATAGAATATACGATAAAAACCTATTAATGATAAAAGAGCATATTCT
TGCTATTGCAATATATGAATCAAGAATATTGAAACGAAAATATAAGAATAAAGATGATAA
AGAAGTTTGTAAAATCATAAATAAAACTTTCGCTGATATAAGAGATATTATAGGAGGTAC
TGATTATTGGAATGATTTGAGCAATAGAAAATTAGTAGGAAAAATTAACACAAATTCAAA
ATATGTTCACAGGAATAAAAAAAAATGATAAGCTTTTTCGTGATGAGTGGTGGAAAGTTAT
TAAAAAAGATGTATGGAATGTGATATCATGGGTATTCAAGGATAAAACTGTTTGTAAAGA

AGATGATATTGAAAATATACCACAATTCTTCAGATGGTTTAGTGAATGGGGTGATGATTA
TTGCCAGGATAAAACAAAAATG
>Venezuela Ven4_EBA-175KJ419578.1
TATGTATGTATTCCTGATCGTAGAATCCAATTATGCATTGTTAATCTTAGCATTATTAAAA
CATATACAAAAGAGACCATGAAGGATCATTTCATTGAAGCCTCTAAAAAAGAATCTCAA
CTTTTGCTTAAAAAAAATGATAACAAATATAATTCTAAATTTTGTAATGATTTGAAGAATA
GTTTTTTAGATTATGGACATCTTGCTATGGGAAATGATATGGATTTTGGAGGTTATTCAAC
TAAGGCAGAAAACAAAATTCAAGAAGTTTTTAAAGGGGCTCATGGGAAAATAAGTGAAC
ATGAAATTAAAAATTTTAGAAAAAAATGGTGGAATGAATTTAGAGAGAAACTTTGGGAA
GCTATGTTATCTGAGCATAAAAATAATATAAATAATTGTAAAAATATTCCCCAAGAAGAA
TTACAAATTACTCAATGGATAAAAGAATGGCATGGAGAATTTTTGCTTGAAAGAGATAAT
AGATCAAAATTGCCAAAAAGTAAATGTAAAAATAATACATTATATGAAGCATGTGAGAA
GGAATGTATTGATCCATGTATGAAATATAGAGATTGGATTATTAGAAGTAAATTTGAATG
GCATACGTTATCGAAAGAATATGAAACTCAAAAAGTTCCAAAGGAAAATGCGGAAAATT
ATTTAATCAAAATTTCAAAAAACAAGAATGATGCTAAAGTAAGTTTATTATTGAATAATTG
TGATGCTGAATATTCAAAATATTGTGATTGTAAACATACTACTACTCTCGTTAAAAGCGTT
TTAAATGGTAACGACAATACAATTAAGGAAAAGCGTGAACATATTGATTTAGATGATTTT
TCTAAATTTGGATGTGATAAAAATTCCGTTGATACAAACACAAAGGTGTGGGAATGTAAA
AAACCTTATAAATTATCCACTAAAGATGTATGTGTACCTCCGAGGAGGCAAGAATTATGT
CTTGGAAACATTGATAGAATATACGATAAAAACCTATTAATGATAAAAGAGCATATTCTT
GCTATTGCAATATATGAATCAAGAATATTGAAACGAAAATATAAGAATAAAGATGATAA
AGAAGTTTGTAAAATCATAAATAAAACTTTCGCTGATATAAGAGATATTATAGGAGGTAC
TGATTATTGGAATGATTTGAGCAATAGAAAATTAGTAGGAAAAATTAACACAAATTCAAA
TTATGTTCACAGGAATAAACAAAATGATAAGCTTTTTCGTGATGAGTGGTGGAAAGTTAT
TAAAAAAGATGTATGGAATGTGATATCATGGGTATTCAAGGATAAAACTGTTTGTAAAGA
AGATGATATTGAAAATATACCACAATTCTTCAGATGGTTTAGTGAATGGGGTGATGATTA
TTGCCAGGATAAAACAAAAATG
>Venezuela Ven6_EBA-175KJ419579.1
TATGTATGTATTCCTGATCGTAGAATCCAATTATGCATTGTTAATCTTAGCATTATTAAAA
CATATACAAAAGAGACCATGAAGGATCATTTCATTGAAGCCTCTAAAAAAGAATCTCAA
CTTTTGCTTAAAAAAAATGATAACAAATATAATTCTAAATTTTGTAATGATTTGAAGAATA
GTTTTTTAGATTATGGACATCTTGCTATGGGAAATGATATGGATTTTGGAGGTTATTCAAC
TAAGGCAGAAAACAAAATTCAAGAAGTTTTTAAAGGGGCTCATGGGGAAATAAGTGAAC
ATAAAATTAAAAATTTTAGAAAAGAATGGTGGAATGAATTTAGAGAGAAACTTTGGGAA
GCTATGTTATCTGAGCATAAAAATAATATAAATAATTGTAAAAATATTCCCCAAGAAGAA
TTACAAATTACTCAATGGATAAAAGAATGGCATGGAGAATTTTTGCTTGAAAGAGATAAT
AGATCAAAATTGCCAAAAAGTAAATGTAAAAATAATACATTATATGAAGCATGTGAGAA
GGAATGTATTGATCCATGTATGAAATATAGAGATTGGATTATTAGAAGTAAATTTGAATG
GCATACGTTATCGAAAGAATATGAAACTCAAAAAGTTCCAAAGGAAAATGCGGAAAATT
ATTTAATCAAAATTTCAGAAAACAAGAATGATGCTAAAGTAAGTTTATTATTGAATAATT
GTGATGCTGAATATTCAAAATATTGTGATTGTAAACATACTACTACTCTCGTTAAAAGCGT
TTTAAATGGTAACGACAATACAATTAAGGAAAAGCGTGAACATATTGATTTAGATGATTT
TTCTAAATTTGGATGTGATAAAAATTCCGTTGATACAAACACAAAGGTGTGGGAATGTAA
AAAACCTTATAAATTATCCACTAAAGATGTATGTGTACCTCCGAGGAGGCAAGAATTATG

TCTTGGAAACATTGATAGAATATACGATAAAAACCTATTAATGATAAAAGAGCATATTCT
TGCTATTGCAATATATGAATCAAGAATATTGAAACGAAAATATAAGAATAAAGATGATAA
AGAAGTTTGTAAAATCATAAATAAAACTTTCGCTGATATAAGAGATATTATAGGAGGTAC
TGATTATTGGAATGATTTGAGCAATAGAAAATTAGTAGGAAAAATTAACACAAATTCAAA
TTATGTTCACAGGAATAAACAAAATGATAAGCTTTTTCGTGATGAGTGGTGGAAAGTTAT
TAAAAAAGATGTATGGAATGTGATATCATGGGTATTCAAGGATAAAACTGTTTGTAAAGA
AGATGATATTGAAAATATACCACAATTCTTCAGATGGTTTAGTGAATGGGGTGATGATTA
TTGCCAGGATAAAACAAAAATG
>Venezuela Ven7_EBA-175KJ419580.1
TATGTATGTATTCCTGATCGTAGAATCCAATTATGCATTGTTAATCTTAGCATTATTAAAA
CATATACAAAAGAGACCATGAAGGATCATTTCATTGAAGCCTCTAAAAAAGAATCTCAA
CTTTTGCTTAAAAAAAAATGATAACAAATATAATTCTAAATTTTGTAATGATTTGAAGAATA
GTTTTTTAGATTATGGACATCTTGCTATGGGAAATGATATGGATTTTGGAGGTTATTCAAC
TAAGGCAGAAAACAAAATTCAAGAAGTTTTTAAAGGGGCTCATGGGAAAATAAGTGAAC
ATGAAATTAAAAATTTTAGAAAAAAATGGTGGAATGAATTTAGAGAGAAACTTTGGGAA
GCTATGTTATCTGAGCATAAAAATAATATAAATAATTGTAAAAATATTCCCCAAGAAGAA
TTACAAATTACTCAATGGATAAAAGAATGGCATGGAGAATTTTTGCTTGAAAGAGATAAT
AGATCAAAATTGCCAAAAAGTAAATGTAAAAATAATACATTATATGAAGCATGTGAGAA
GGAATGTATTGATCCATGTATGAAATATAGAGATTGGATTATTAGAAGTAAATTTGAATG
GCATACGTTATCGAAAGAATATGAAACTCAAAAAGTTCCAAAGGAAAATGCGGAAAATT
ATTTAATCAAAATTTCAGAAAACAAGAATGATGCTAAAGTAAGTTTATTATTGAATAATT
GTGATGCTGAATATTCAAAATATTGTGATTGTAAACATACTACTACTCTCGTTAAAAGCGT
TTTAAATGGTAACGACAATACAATTAAGGAAAAGCGTGAACATATTGATTTAGATGATTT
TTCTAAATTTGGATGTGATAAAAATTCCGTTGATACAAACACAAAGGTGTGGGAATGTAA
AAACCCTTATATATTATCCACTAAAGATGTATGTGTACCTCCGAGGAGGCAAGAATTATG
TCTTGGAAACATTGATAGAATATACGATAAAAACCTATTAATGATAAAAGAGCATATTCT
TGCTATTGCAATATATGAATCAAGAATATTGAAACGAAAATATAAGAATAAAGATGATAA
AGAAGTTTGTAAAATCATAAATAAAACTTTCGCTGATATAAGAGATATTATAGGAGGTAC
TGATTATTGGAATGATTTGAGCAATAGAAAATTAGTAGGAAAAATTAACACAAATTCAAA
ATATGTTCACAGGAATAAAAAAAAATGATAAGCTTTTTCGTGATGAGTGGTGGAAAGTTAT
TAAAAAAGATGTATGGAATGTGATATCATGGGTATTCAAGGATAAAACTGTTTGTAAAGA
AGATGATATTGAAAATATACCACAATTCTTCAGATGGTTTAGTGAATGGGGTGATGATTA
TTGCCAGGATAAAACAAAAATG
>Venezuela Ven9_EBA-175KJ419581.1
TATGTATGTATTCCTGATCGTAGAATCCAATTATGCATTGTTAATCTTAGCATTATTAAAA
CATATACAAAAGAGACCATGAAGGATCATTTCATTGAAGCCTCTAAAAAAGAATCTCAA
CTTTTGCTTAAAAAAAAATGATAACAAATATAATTCTAAATTTTGTAATGATTTGAAGAATA
GTTTTTTAGATTATGGACATCTTGCTATGGGAAATGATATGGATTTTGGAGGTTATTCAAC
TAAGGCAGAAAACAAAATTCAAGAAGTTTTTAAAGGGGCTCATGGGAAAATAAGTGAAC
ATGAAATTAAAAATTTTAGAAAAAAATGGTGGAATGAATTTAGAGAGAAACTTTGGGAA
GCTATGTTATCTGAGCATAAAAATAATATAAATAATTGTAAAAATATTCCCCAAGAAGAA
TTACAAATTACTCAATGGATAAAAGAATGGCATGGAGAATTTTTGCTTGAAAGATATAAT
AGATCAAAATTGCCAAAAAGTAAATGTAAAAATAATACATTATATGAAGCATGTGAGAA
GGAATGTATTGATCCATGTATGAAATATAGAGATTGGATTATTAGAAGTAAATTTGAATG

GCATACGTTATCGAAAGAATATGAAACTCAAAATGTTTCAAAGGAAAATGCGGAAAATT
ATTTAATCAAAATTTCAAAAAACAAGAATGATGCTAAAGTAAGTTTATTATTGAATAATTG
TGATGCTGAATATTCAAAATATTGTGATTGTAAACATACTACTACTCTCGTTAAAAGCGTT
TTAAATGGTAACGACAATACAATTAAGGAAAAGCGTGAACATATTGATTTAGATGATTTT
TCTAAATTTGGATGTGATAAAAATTCCGTTGATACAAACACAAAGGTGTGGGAATGTAAA
AAACCTTATATATTATCCACTAAAGATGTATGTGTACCTCCGAGGAGGCAAGAATTATGT
CTTGGAAACATTGATAGAATATACGATAAAAACCTATTAATGATAAAAGAGCATATTCTT
GCTATTGCAATATATGAATCAAGAATATTGAAACGAAAATATAAGAATAAAGATGATAA
AGAAGTTTGTAAAATCATAAATAAAACTTTCGCTGATATAAGAGATATTATAGGAGGTAC
TGATTATTGGAATGATTTGAGCAATAGAAAATTAGTAGGAAAAATTAACACAAATTCAAA
ATATGCTCACAGGAATAAAAAAAATGATAAGCTTTTTCGTGATGAGTGGTGGAAAGTTAT
TAAAAAAGATGTATGGAATGTGATATCATGGGTATTCAAGGATAAAACTGTTTGTAAAGA
AGATGATATTGAAAATATACCACAATTCTTCAGATGGTTTAGTGAATGGGGTGATGATTA
TTGCCAGGATAAAACAAAAATG
>Venezuela Ven12_EBA-175KJ419582.1
TATGTATGTATTCCTGATCGTAGAATCCAATTATGCATTGTTAATCTTAGCATTATTAAAA
CATATACAAAAGAGACCATGAAGGATCATTTCATTGAAGCCTCTAAAAAAGAATCTCAA
CTTTTGCTTAAAAAAAATGATAACAAATATAATTCTAAATTTTGTAATGATTTGAAGAATA
GTTTTTTAGATTATGGACATCTTGCTATGGGAAATGATATGGATTTTGGAGGTTATTCAAC
TAAGGCAGAAAACAAAATTCAAGAAGTTTTTAAAGGGGCTCATGGGAAAATAAGTGAAC
ATGAAATTAAAAATTTTAGAAAAAAATGGTGGAATGAATTTAGAGAGAAACTTTGGGAA
GCTATGTTATCTGAGCATAAAAATAATATAAATAATTGTAAAAATATTCCCCAAGAAGAA
TTACAAATTACTCAATGGATAAAAGAATGGCATGGAGAATTTTTGCTTGAAAGAGATAAT
AGATCAAAATTGCCAAAAAGTAAATGTAAAAATAATACATTATATGAAGCATGTGAGAA
GGAATGTATTGATCCATGTATGAAATATAGAGATTGGATTATTAGAAGTAAATTTGAATG
GCATACGTTATCGAAAGAATATGAAACTCAAAAAGTTCCAAAGGAAAATGCGGAAAATT
ATTTAATCAAAATTTCAGAAAACAAGAATGATGCTAAAGTAAGTTTATTATTGAATAATT
GTGATGCTGAATATTCAAAATATTGTGATTGTAAACATACTACTACTCTCGTTAAAAGCGT
TTTAAATGGTAACGACAATACAATTAAGGAAAAGCGTGAACATATTGATTTAGATGATTT
TTCTAAATTTGGATGTGATAAAAATTCCGTTGATACAAACACAAAGGTGTGGGAATGTAA
AAACCCTTATATATTATCCACTAAAGATGTATGTGTACCTCCGAGGAGGCAAGAATTATG
TCTTGGAAACATTGATAGAATATACGATAAAAACCTATTAATGATAAAAGAGCATATTCT
TGCTATTGCAATATATGAATCAAGAATATTGAAACGAAAATATAAGAATAAAGATGATAA
AGAAGTTTGTAAAATCATAAATAAAACTTTCGCTGATATAAGAGATATTATAGGAGGTAC
TGATTATTGGAATGATTTGAGCAATAGAAAATTAGTAGGAAAAATTAACACAAATTCAAA
ATATGTTCACAGGAATAAAAAAAATGATAAGCTTTTTCGTGATGAGTGGTGGAAAGTTAT
TAAAAAAGATGTATGGAATGTGATATCATGGGTATTCAAGGATAAAACTGTTTGTAAAGA
AGATGATATTGAAAATATACCACAATTCTTCAGATGGTTTAGTGAATGGGGTGATGATTA
TTGCCAGGATAAAACAAAAATG
>Venezuela Ven13_EBA-175KJ419583.1
TATGTATGTATTCCTGATCGTAGAATCCAATTATGCATTGTTAATCTTAGCATTATTAAAA
CATATACAAAAGAGACCATGAAGGATCATTTCATTGAAGCCTCTAAAAAAGAATCTCAA
CTTTTGCTTAAAAAAAATGATAACAAATATAATTCTAAATTTTGTAATGATTTGAAGAATA
GTTTTTTAGATTATGGACATCTTGCTATGGGAAATGATATGGATTTTGGAGGTTATTCAAC

TAAGGCAGAAAACAAAATTCAAGAAGTTTTTAAAGGGGCTCATGGGAAAATAAGTGAAC
ATGAAATTAAAAATTTTAGAAAAAAATGGTGGAATGAATTTAGAGAGAAACTTTGGGAA
GCTATGTTATCTGAGCATAAAAATAATATAAATAATTGTAAAAATATTCCCCAAGAAGAA
TTACAAATTACTCAATGGATAAAAGAATGGCATGGAGAATTTTTGCTTGAAAGAGATAAT
AGATCAAAATTGCCAAAAAGTAAATGTAAAAATAATACATTATATGAAGCATGTGAGAA
GGAATGTATTGATCCATGTATGAAATATAGAGATTGGATTATTAGAAGTAAATTTGAATG
GCATACGTTATCGAAAGAATATGAAACTCAAAAAGTTCCAAAGGAAAATGCGGAAAATT
ATTTAATCAAAATTTCAGAAAACAAGAATGATGCTAAAGTAAGTTTATTATTGAATAATT
GTGATGCTGAATATTCAAAATATTGTGATTGTAAACATACTACTACTCTCGTTAAAAGCGT
TTTAAATGGTAACGACAATACAATTAAGGAAAAGCGTGAACATATTGATTTAGATGATTT
TTCTAAATTTGGATGTGATAAAAATTCCGTTGATACAAACACAAAGGTGTGGGAATGTAA
AAACCCTTATATATTATCCACTAAAGATGTATGTGTACCTCCGAGGAGGCAAGAATTATG
TCTTGGAAACATTGATAGAATATACGATAAAAACCTATTAATGATAAAAGAGCATATTCT
TGCTATTGCAATATATGAATCAAGAATATTGAAACGAAAATATAAGAATAAAGATGATAA
AGAAGTTTGTAAAATCATAAATAAAACTTTCGCTGATATAAGAGATATTATAGGAGGTAC
TGATTATTGGAATGATTTGAGCAATAGAAAATTAGTAGGAAAAATTAACACAAATTCAAA
ATATGTTCACAGGAATAAAAAAAATGATAAGCTTTTTCGTGATGAGTGGTGGAAAGTTAT
TAAAAAAGATGTATGGAATGTGATATCATGGGTATTCAAGGATAAAACTGTTTGTAAAGA
AGATGATATTGAAAATATACCACAATTCTTCAGATGGTTTAGTGAATGGGGTGATGATTA
TTGCCAGGATAAAACAAAAATG
>Venezuela Ven14_EBA-175KJ419584.1
TATGTATGTATTCCTGATCGTAGAATCCAATTATGCATTGTTAATCTTAGCATTATTAAAA
CATATACAAAAGAGACCATGAAGGATCATTTCATTGAAGCCTCTAAAAAAGAATCTCAA
CTTTTGCTTAAAAAAAAATGATAACAAATATAATTCTAAATTTTGTAATGATTTGAAGAATA
GTTTTTTAGATTATGGACATCTTGCTATGGGAAATGATATGGATTTTGGAGGTTATTCAAC
TAAGGCAGAAAACAAAATTCAAGAAGTTTTTAAAGGGGCTCATGGGAAAATAAGTGAAC
ATGAAATTAAAAATTTTAGAAAAAAATGGTGGAATGAATTTAGAGAGAAACTTTGGGAA
GCTATGTTATCTGAGCATAAAAATAATATAAATAATTGTAAAAATATTCCCCAAGAAGAA
TTACAAATTACTCAATGGATAAAAGAATGGCATGGAGAATTTTTGCTTGAAAGAGATAAT
AGATCAAAATTGCCAAAAAGTAAATGTAAAAATAATACATTATATGAAGCATGTGAGAA
GGAATGTATTGATCCATGTATGAAATATAGAGATTGGATTATTAGAAGTAAATTTGAATG
GCATACGTTATCGAAAGAATATGAAACTCAAAAAGTTTCAAAGGAAAATGCGGAAAATT
ATTTAATCAAAATTTCAGAAAACAAGAATGATGCTAAAGTAAGTTTATTATTGAATAATT
GTGATGCTGAATATTCAAAATATTGTGATTGTAAACATACTACTACTCTCGTTAAAAGCGT
TTTAAATGGTAACGACAATACAATTAAGGAAAAGCGTGAACATATTGATTTAGATGATTT
TTCTAAATTTGGATGTGATAAAAATTCCGTTGATACAAACACAAAGGTGTGGGAATGTAA
AAACCCTTATATATTATCCACTAAAGATGTATGTGTACCTCCGAGGAGGCAAGAATTATG
TCTTGGAAACATTGATAGAATATACGATAAAAACCTATTAATGATAAAAGAGCATATTCT
TGCTATTGCAATATATGAATCAAGAATATTGAAACGAAAATATAAGAATAAAGATGATAA
AGAAGTTTGTAAAATCATAAATAAAACTTTCGCTGATATAAGAGATATTATAGGAGGTAC
TGATTATTGGAATGATTTGAGCAATAGAAAATTAGTAGGAAAAATTAACACAAATTCAAA
ATATGTTCACAGGAATAAAAAAAATGATAAGCTTTTTCGTGATGAGTGGTGGAAAGTTAT
TAAAAAAGATGTATGGAATGTGATATCATGGGTATTCAAGGATAAAACTGTTTGTAAAGA
AGATGATATTGAAAATATACCACAATTCTTCAGATGGTTTAGTGAATGGGGTGATGATTA

TTGCCAGGATAAAACAAAAATG
>Venezuela Ven15_EBA-175KJ419585.1
TATGTATGTATTCCTGATCGTAGAATCCAATTATGCATTGTTAATCTTAGCATTATTAAAA
CATATACAAAAGAGACCATGAAGGATCATTTCATTGAAGCCTCTAAAAAAGAATCTCAA
CTTTTGCTTAAAAAAAAATGATAACAAATATAATTCTAAATTTTGTAATGATTTGAAGAATA
GTTTTTTAGATTATGGACATCTTGCTATGGGAAATGATATGGATTTTGGAGGTTATTCAAC
TAAGGCAGAAAACAAAATTCAAGAAGTTTTTAAAGGGGCTCATGGGAAAATAAGTGAAC
ATGAAATTAAAAATTTTAGAAAAAAATGGTGGAATGAATTTAGAGAGAAACTTTGGGAA
GCTATGTTATCTGAGCATAAAAATAATATAAATAATTGTAAAAATATTCCCCAAGAAGAA
TTACAAATTACTCAATGGATAAAAGAATGGCATGGAGAATTTTTGCTTGAAAGAGATAAT
AGATCAAAATTGCCAAAAAGTAAATGTAAAAATAATACATTATATGAAGCATGTGAGAA
GGAATGTATTGATCCATGTATGAAATATAGAGATTGGATTATTAGAAGTAAATTTGAATG
GCATACGTTATCGAAAGAATATGAAACTCAAAAAGTTCCAAAGGAAAATGCGGAAAATT
ATTTAATCAAAATTTCAAAAAACAAGAATGATGCTAAAGTAAGTTTATTATTGAATAATTG
TGATGCTGAATATTCAAAATATTGTGATTGTAAACATACTACTACTCTCGTTAAAAGCGTT
TTAAATGGTAACGACAATACAATTAAGGAAAAGCGTGAACATATTGATTTAGATGATTTT
TCTAAATTTGGATGTGATAAAAATTCCGTTGATACAAACACAAAGGTGTGGGAATGTAAA
AAACCTTATAAATTATCCACTAAAGATGTATGTGTACCTCCGAGGAGGCAAGAATTATGT
CTTGGAAACATTGATAGAATATACGATAAAAACCTATTAATGATAAAAGAGCATATTCTT
GCTATTGCAATATATGAATCAAGAATATTGAAACGAAAATATAAGAATAAAGATGATAA
AGAAGTTTGTAAAATCATAAATAAAACTTTCGCTGATATAAGAGATATTATAGGAGGTAC
TGATTATTGGAATGATTTGAGCAATAGAAAATTAGTAGGAAAAATTAACACAAATTCAAA
TTATGTTCACAGGAATAAACAAAATGATAAGCTTTTTCGTGATGAGTGGTGGAAAGTTAT
TAAAAAAGATGTATGGAATGTGATATCATGGGTATTCAAGGATAAAACTGTTTGTAAAGA
AGATGATATTGAAAATATACCACAATTCTTCAGATGGTTTAGTGAATGGGGTGATGATTA
TTGCCAGGATAAAACAAAAATG
>French Guiana (Camopi) Cmp1_EBA-175KJ419586.1
TATGTATGTATTCCTGATCGTAGAATCCAATTATGCATTGTTAATCTTAGCATTATTAAAA
CATATACAAAAGAGACCATGAAGGATCATTTCATTGAAGCCTCTAAAAAAGAATCTCAA
CTTTTGCTTAAAAAAAAATGATAACAAATATAATTCTAAATTTTGTAATGATTTGAAGAATA
GTTTTTTAGATTATGGACATCTTGCTATGGGAAATGATATGGATTTTGGAGGTTATTCAAC
TAAGGCAGAAAACAAAATTCAAGAAGTTTTTAAAGGGGCTCATGGGAAAATAAGTGAAC
ATGAAATTAAAAATTTTAGAAAAAAATGGTGGAATGAATTTAGAGAGAAACTTTGGGAA
GCTATGTTATCTGAGCATAAAAATAATATAAATAATTGTAAAAATATTCCCCAAGAAGAA
TTACAAATTACTCAATGGATAAAAGAATGGCATGGAGAATTTTTGCTTGAAAGAGATAAT
AGATCAAAATTGCCAAAAAGTAAATGTAAAAATAATACATTATATGAAGCATGTGAGAA
GGAATGTATTGATCCATGTATGAAATATAGAGATTGGATTATTAGAAGTAAATTTGAATG
GCATACGTTATCGAAAGAATATGAAACTCAAAATGTTTCAAAGGAAAATGCGGAAAATT
ATTTAATCAAAATTTCAAAAAACAAGAATGATGCTAAAGTAAGTTTATTATTGAATAATTG
TGATGCTGAATATTCAAAATATTGTGATTGTAAACATACTACTACTCTCGTTAAAAGCGTT
TTAAATGGTAACGACAATACAATTAAGGAAAAGCGTGAACATATTGATTTAGATGATTTT
TCTAAATTTGGATGTGATAAAAATTCCGTTGATACAAACACAAAGGTGTGGGAATGTAAA
AAACCTTATAAATTATCCACTAAAGATGTATGTGTACCTCCGAGGAGGCAAGAATTATGT
CTTGGAAACATTGATAGAATATACGATAAAAACCTATTAATGATAAAAGAGCATATTCTT

GCTATTGCAATATATGAATCAAGAATATTGAAACGAAAATATAAGAATAAAGATGATAA
AGAAGTTTGTAAAATCATAAATAAAACTTTCGCTGATATAAGAGATATTATAGGAGGTAC
TGATTATTGGAATGATTTGAGCAATAGAAAATTAGTAGGAAAAATTAACACAAATTCAAA
TTATGTTCACAGGAATAAACAAAATGATAAGCTTTTTCGTGATGAGTGGTGGAAAGTTAT
TAAAAAAGATGTATGGAATGTGATATCATGGGTATTCAAGGATAAAACTGTTTGTAAAGA
AGATGATATTGAAAATATACCACAATTCTTCAGATGGTTTAGTGAATGGGGTGATGATTA
TTGCCAGGATAAAACAAAAATG
>French Guiana (Camopi) Cmp2_EBA-175KJ419587.1
TATGTATGTATTCCTGATCGTAGAATCCAATTATGCATTGTTAATCTTAGCATTATTAAAA
CATATACAAAAGAGACCATGAAGGATCATTTCATTGAAGCCTCTAAAAAAGAATCTCAA
CTTTTGCTTAAAAAAAAATGATAACAAATATAATTCTAAATTTTGTAATGATTTGAAGAATA
GTTTTTTAGATTATGGACATCTTGCTATGGGAAATGATATGGATTTTGGAGGTTATTCAAC
TAAGGCAGAAAACAAAATTCAAGAAGTTTTTAAAGGGGCTCATGGGAAAATAAGTGAAC
ATGAAATTAAAAATTTTAGAAAAAAATGGTGGAATGAATTTAGAGAGAAACTTTGGGAA
GCTATGTTATCTGAGCATAAAAATAATATAAATAATTGTAAAAATATTCCCCAAGAAGAA
TTACAAATTACTCAATGGATAAAAGAATGGCATGGAGAATTTTTGCTTGAAAGAGATAAT
AGATCAAAATTGCCAAAAAGTAAATGTAAAAATAATACATTATATGAAGCATGTGAGAA
GGAATGTATTGATCCATGTATGAAATATAGAGATTGGATTATTAGAAGTAAATTTGAATG
GCATACGTTATCGAAAGAATATGAAACTCAAAAAGTTTCAAAGGAAAATGCGGAAAATT
ATTTAATCAAAATTTCAGAAAACAAGAATGATGCTAAAGTAAGTTTATTATTGAATAATT
GTGATGCTGAATATTCAAAATATTGTGATTGTAAACATACTACTACTCTCGTTAAAAGCGT
TTTAAATGGTAACGACAATACAATTAAGGAAAAGCGTGAACATATTGATTTAGATGATTT
TTCTAAATTTGGATGTGATAAAAATTCCGTTGATACAAACACAAAGGTGTGGGAATGTAA
AAACCCTTATATATTATCCACTAAAGATGTATGTGTACCTCCGAGGAGGCAAGAATTATG
TCTTGGAAACATTGATAGAATATACGATAAAAACCTATTAATGATAAAAGAGCATATTCT
TGCTATTGCAATATATGAATCAAGAATATTGAAACGAAAATATAAGAATAAAGATGATAA
AGAAGTTTGTAAAATCATAAATAAAACTTTCGCTGATATAAGAGATATTATAGGAGGTAC
TGATTATTGGAATGATTTGAGCAATAGAAAATTAGTAGGAAAAATTAACACAAATTCAAA
ATATGTTCACAGGAATAAAAAAAAATGATAAGCTTTTTCGTGATGAGTGGTGGAAAGTTAT
TAAAAAAGATGTATGGAATGTGATATCATGGGTATTCAAGGATAAAACTGTTTGTAAAGA
AGATGATATTGAAAATATACCACAATTCTTCAGATGGTTTAGTGAATGGGGTGATGATTA
TTGCCAGGATAAAACAAAAATG
>French Guiana (Camopi) Cmp5_EBA-175KJ419588.1
TATGTATGTATTCCTGATCGTAGAATCCAATTATGCATTGTTAATCTTAGCATTATTAAAA
CATATACAAAAGAGACCATGAAGGATCATTTCATTGAAGCCTCTAAAAAAGAATCTCAA
CTTTTGCTTAAAAAAAAATGATAACGAATATAATTCTAAATTTTGTAATGATTTGAAGAATA
GTTTTTTAGATTATGGACATCTTGCTATGGGAAATGATATGGATTTTGGAGGTTATTCAAC
TAAGGCAGAAAACAAAATTCAAGAAGTTTTTAAAGGGGCTCATGGGGAAATAAGTGAAC
ATAAAATTAAAAATTTTAGAAAAGAATGGTGGAATGAATTTAGAGAGAAACTTTGGGAA
GCTATGTTATCTGAGCATAAAAATAATATAAATAATTGTAAAAATATTCCCCAAGAAGAA
TTACAAATTACTCAATGGATAAAAGAATGGCATGGAGAATTTTTGCTTGAAAGATATAAT
AGATCAAAATTGCCAAAAAGTAAATGTAAAAATAATACATTATATGAAGCATGTGAGAA
GGAATGTATTGATCCATGTATGAAATATAGAGATTGGATTATTAGAAGTAAATTTGAATG
GCATACGTTATCGAAAGAATATGAAACTCAAAAAGTTTCAAAGGAAAATGCGGAAAATT

ATTTAATCAAAATTTCAGAAAACAAGAATGATGCTAAAGTAAGTTTATTATTGAATAATT
GTGATGCTGAATATTCAAAATATTGTGATTGTAAACATACTACTACTCTCGTTAAAAGCGT
TTTAAATGGTAACGACAATACAATTAAGGAAAAGCGTGAACATATTGATTTAGATGATTT
TTCTAAATTTGGATGTGATAAAAATTCCGTTGATACAAACACAAAGGTGTGGGAATGTAA
AAAACCTTATATATTATCCACTAAAGATGTATGTGTACCTCCGAGGAGGCAAGAATTATG
TCTTGGAAACATTGATAGAATATACGATAAAAACCTATTAATGATAAAAGAGCATATTCT
TGCTATTGCAATATATGAATCAAGAATATTGAAACGAAAATATAAGAATAAAGATGATAA
AGAAGTTTGTAAAATCATAAATAAAACTTTCGCTGATATAAGAGATATTATAGGAGGTAC
TGATTATTGGAATGATTTGAGCAATAGAAAATTAGTAGGAAAAATTAACACAAATTCAAA
ATATGTTCACAGGAATAAAAAAAATGATAAGCTTTTTCGTGATGAGTGGTGGAAAGTTAT
TAAAAAAGATGTATGGAATGTGATATCATGGGTATTCAAGGATAAAACTGTTTGTAAAGA
AGATGATATTGAAAATATACCACAATTCTTCAGATGGTTTAGTGAATGGGGTGATGATTA
TTGCCAGGATAAAACAAAAATG
>French Guiana (Camopi) Cmp6_EBA-175KJ419589.1
TATGTATGTATTCCTGATCGTAGAATCCAATTATGCATTGTTAATCTTAGCATTATTAAAA
CATATACAAAAGAGACCATGAAGGATCATTTCATTGAAGCCTCTAAAAAAGAATCTCAA
CTTTTGCTTAAAAAAAAATGATAACGAATATAATTCTAAATTTTGTAATGATTTGAAGAATA
GTTTTTTAGATTATGGACATCTTGCTATGGGAAATGATATGGATTTTGGAGGTTATTCAAC
TAAGGCAGAAAACAAAATTCAAGAAGTTTTTAAAGGGGCTCATGGGGAAATAAGTGAAC
ATAAAATTAAAAATTTTAGAAAAGAATGGTGGAATGAATTTAGAGAGAAACTTTGGGAA
GCTATGTTATCTGAGCATAAAAATAATATAAATAATTGTAAAAATATTCCCCAAGAAGAA
TTACAAATTACTCAATGGATAAAAGAATGGCATGGAGAATTTTTGCTTGAAAGATATAAT
AGATCAAAATTGCCAAAAAGTAAATGTAAAAATAATACATTATATGAAGCATGTGAGAA
GGAATGTATTGATCCATGTATGAAATATAGAGATTGGATTATTAGAAGTAAATTTGAATG
GCATACGTTATCGAAAGAATATGAAACTCAAAAAGTTTCAAAGGAAAATGCGGAAAATT
ATTTAATCAAAATTTCAGAAAACAAGAATGATGCTAAAGTAAGTTTATTATTGAATAATT
GTGATGCTGAATATTCAAAATATTGTGATTGTAAACATACTACTACTCTCGTTAAAAGCGT
TTTAAATGGTAACGACAATACAATTAAGGAAAAGCGTGAACATATTGATTTAGATGATTT
TTCTAAATTTGGATGTGATAAAAATTCCGTTGATACAAACACAAAGGTGTGGGAATGTAA
AAAACCTTATATATTATCCACTAAAGATGTATGTGTACCTCCGAGGAGGCAAGAATTATG
TCTTGGAAACATTGATAGAATATACGATAAAAACCTATTAATGATAAAAGAGCATATTCT
TGCTATTGCAATATATGAATCAAGAATATTGAAACGAAAATATAAGAATAAAGATGATAA
AGAAGTTTGTAAAATCATAAATAAAACTTTCGCTGATATAAGAGATATTATAGGAGGTAC
TGATTATTGGAATGATTTGAGCAATAGAAAATTAGTAGGAAAAATTAACACAAATTCAAA
ATATGTTCACAGGAATAAAAAAAATGATAAGCTTTTTCGTGATGAGTGGTGGAAAGTTAT
TAAAAAAGATGTATGGAATGTGATATCATGGGTATTCAAGGATAAAACTGTTTGTAAAGA
AGATGATATTGAAAATATACCACAATTCTTCAGATGGTTTAGTGAATGGGGTGATGATTA
TTGCCAGGATAAAACAAAAATG
>French Guiana (Camopi) Cmp7_EBA-175KJ419590.1
TATGTATGTATTCCTGATCGTAGAATCCAATTATGCATTGTTAATCTTAGCATTATTAAAA
CATATACAAAAGAGACCATGAAGGATCATTTCATTGAAGCCTCTAAAAAAGAATCTCAA
CTTTTGCTTAAAAAAAAATGATAACAAATATAATTCTAAATTTTGTAATGATTTGAAGAATA
GTTTTTTAGATTATGGACATCTTGCTATGGGAAATGATATGGATTTTGGAGGTTATTCAAC
TAAGGCAGAAAACAAAATTCAAGAAGTTTTTAAAGGGGCTCATGGGAAAATAAGTGAAC

ATGAAATTAAAAATTTTAGAAAAAAATGGTGGAATGAATTTAGAGAGAAACTTTGGGAA
GCTATGTTATCTGAGCATAAAAATAATATAAATAATTGTAAAAATATTCCCCAAGAAGAA
TTACAAATTACTCAATGGATAAAAGAATGGCATGGAGAATTTTTGCTTGAAAGAGATAAT
AGATCAAAATTGCCAAAAAGTAAATGTAAAAATAATACATTATATGAAGCATGTGAGAA
GGAATGTATTGATCCATGTATGAAATATAGAGATTGGATTATTAGAAGTAAATTTGAATG
GCATACGTTATCGAAAGAATATGAAACTCAAAAAGTTTCAAAGGAAAATGCGGAAAATT
ATTTAATCAAAATTTCAGAAAACAAGAATGATGCTAAAGTAAGTTTATTATTGAATAATT
GTGATGCTGAATATTCAAAATATTGTGATTGTAAACATACTACTACTCTCGTTAAAAGCGT
TTTAAATGGTAACGACAATACAATTAAGGAAAAGCGTGAACATATTGATTTAGATGATTT
TTCTAAATTTGGATGTGATAAAAATTCCGTTGATACAAACACAAAGGTGTGGGAATGTAA
AAACCCTTATATATTATCCACTAAAGATGTATGTGTACCTCCGAGGAGGCAAGAATTATG
TCTTGGAAACATTGATAGAATATACGATAAAAACCTATTAATGATAAAAGAGCATATTCT
TGCTATTGCAATATATGAATCAAGAATATTGAAACGAAAATATAAGAATAAAGATGATAA
AGAAGTTTGTAAAATCATAAATAAAACTTTCGCTGATATAAGAGATATTATAGGAGGTAC
TGATTATTGGAATGATTTGAGCAATAGAAAATTAGTAGGAAAAATTAACACAAATTCAAA
ATATGTTCACAGGAATAAAAAAAATGATAAGCTTTTTCGTGATGAGTGGTGGAAAGTTAT
TAAAAAAGATGTATGGAATGTGATATCATGGGTATTCAAGGATAAAACTGTTTGTAAAGA
AGATGATATTGAAAATATACCACAATTCTTCAGATGGTTTAGTGAATGGGGTGATGATTA
TTGCCAGGATAAAACAAAAATG
>French Guiana (Camopi) Cmp8_EBA-175KJ419591.1
TATGTATGTATTCCTGATCGTAGAATCCAATTATGCATTGTTAATCTTAGCATTATTAAAA
CATATACAAAAGAGACCATGAAGGATCATTTCATTGAAGCCTCTAAAAAAGAATCTCAA
CTTTTGCTTAAAAAAAATGATAACGAATATAATTCTAAATTTTGTAATGATTTGAAGAATA
GTTTTTTAGATTATGGACATCTTGCTATGGGAAATGATATGGATTTTGGAGGTTATTCAAC
TAAGGCAGAAAACAAAATTCAAGAAGTTTTTAAAGGGGCTCATGGGGAAATAAGTGAAC
ATAAAATTAAAAATTTTAGAAAAGAATGGTGGAATGAATTTAGAGAGAAACTTTGGGAA
GCTATGTTATCTGAGCATAAAAATAATATAAATAATTGTAAAAATATTCCCCAAGAAGAA
TTACAAATTACTCAATGGATAAAAGAATGGCATGGAGAATTTTTGCTTGAAAGATATAAT
AGATCAAAATTGCCAAAAAGTAAATGTAAAAATAATACATTATATGAAGCATGTGAGAA
GGAATGTATTGATCCATGTATGAAATATAGAGATTGGATTATTAGAAGTAAATTTGAATG
GCATACGTTATCGAAAGAATATGAAACTCAAAAAGTTTCAAAGGAAAATGCGGAAAATT
ATTTAATCAAAATTTCAGAAAACAAGAATGATGCTAAAGTAAGTTTATTATTGAATAATT
GTGATGCTGAATATTCAAAATATTGTGATTGTAAACATACTACTACTCTCGTTAAAAGCGT
TTTAAATGGTAACGACAATACAATTAAGGAAAAGCGTGAACATATTGATTTAGATGATTT
TTCTAAATTTGGATGTGATAAAAATTCCGTTGATACAAACACAAAGGTGTGGGAATGTAA
AAAACCTTATATATTATCCACTAAAGATGTATGTGTACCTCCGAGGAGGCAAGAATTATG
TCTTGGAAACATTGATAGAATATACGATAAAAACCTATTAATGATAAAAGAGCATATTCT
TGCTATTGCAATATATGAATCAAGAATATTGAAACGAAAATATAAGAATAAAGATGATAA
AGAAGTTTGTAAAATCATAAATAAAACTTTCGCTGATATAAGAGATATTATAGGAGGTAC
TGATTATTGGAATGATTTGAGCAATAGAAAATTAGTAGGAAAAATTAACACAAATTCAAA
ATATGTTCACAGGAATAAAAAAAATGATAAGCTTTTTCGTGATGAGTGGTGGAAAGTTAT
TAAAAAAGATGTATGGAATGTGATATCATGGGTATTCAAGGATAAAACTGTTTGTAAAGA
AGATGATATTGAAAATATACCACAATTCTTCAGATGGTTTAGTGAATGGGGTGATGATTA
TTGCCAGGATAAAACAAAAATG

>French Guiana (Camopi) Cmp11_EBA-175KJ419592.1
TATGTATGTATTCCTGATCGTAGAATCCAATTATGCATTGTTAATCTTAGCATTATTAAAA
CATATACAAAAGAGACCATGAAGGATCATTTCATTGAAGCCTCTAAAAAAGAATCTCAA
CTTTTGCTTAAAAAAAAATGATAACAAATATAATTCTAAATTTTGTAATGATTTGAAGAATA
GTTTTTTAGATTATGGACATCTTGCTATGGGAAATGATATGGATTTTGGAGGTTATTCAAC
TAAGGCAGAAAACAAAATTCAAGAAGTTTTTAAAGGGGCTCATGGGAAAATAAGTGAAC
ATGAAATTAAAAATTTTAGAAAAAAATGGTGGAATGAATTTAGAGAGAAACTTTGGGAA
GCTATGTTATCTGAGCATAAAAATAATATAAATAATTGTAAAAATATTCCCCAAGAAGAA
TTACAAATTACTCAATGGATAAAAGAATGGCATGGAGAATTTTTGCTTGAAAGAGATAAT
AGATCAAAATTGCCAAAAAGTAAATGTAAAAATAATACATTATATGAAGCATGTGAGAA
GGAATGTATTGATCCATGTATGAAATATAGAGATTGGATTATTAGAAGTAAATTTGAATG
GCATACGTTATCGAAAGAATATGAAACTCAAAAAGTTTCAAAGGAAAATGCGGAAAATT
ATTTAATCAAAATTTCAGAAAACAAGAATGATGCTAAAGTAAGTTTATTATTGAATAATT
GTGATGCTGAATATTCAAAATATTGTGATTGTAAACATACTACTACTCTCGTTAAAAGCGT
TTTAAATGGTAACGACAATACAATTAAGGAAAAGCGTGAACATATTGATTTAGATGATTT
TTCTAAATTTGGATGTGATAAAAATTCCGTTGATACAAACACAAAGGTGTGGGAATGTAA
AAACCCTTATATATTATCCACTAAAGATGTATGTGTACCTCCGAGGAGGCAAGAATTATG
TCTTGGAAACATTGATAGAATATACGATAAAAACCTATTAATGATAAAAGAGCATATTCT
TGCTATTGCAATATATGAATCAAGAATATTGAAACGAAAATATAAGAATAAAGATGATAA
AGAAGTTTGTAAAATCATAAATAAAACTTTCGCTGATATAAGAGATATTATAGGAGGTAC
TGATTATTGGAATGATTTGAGCAATAGAAAATTAGTAGGAAAAATTAACACAAATTCAAA
ATATGTTCACAGGAATAAAAAAAATGATAAGCTTTTTCGTGATGAGTGGTGGAAAGTTAT
TAAAAAAGATGTATGGAATGTGATATCATGGGTATTCAAGGATAAAACTGTTTGTAAAGA
AGATGATATTGAAAATATACCACAATTCTTCAGATGGTTTAGTGAATGGGGTGATGATTA
TTGCCAGGATAAAACAAAAATG
>French Guiana (Camopi) Cmp14_EBA-175KJ419593.1
TATGTATGTATTCCTGATCGTAGAATCCAATTATGCATTGTTAATCTTAGCATTATTAAAA
CATATACAAAAGAGACCATGAAGGATCATTTCATTGAAGCCTCTAAAAAAGAATCTCAA
CTTTTGCTTAAAAAAAAATGATAACAAATATAATTCTAAATTTTGTAATGATTTGAAGAATA
GTTTTTTAGATTATGGACATCTTGCTATGGGAAATGATATGGATTTTGGAGGTTATTCAAC
TAAGGCAGAAAACAAAATTCAAGAAGTTTTTAAAGGGGCTCATGGGAAAATAAGTGAAC
ATGAAATTAAAAATTTTAGAAAAAAATGGTGGAATGAATTTAGAGAGAAACTTTGGGAA
GCTATGTTATCTGAGCATAAAAATAATATAAATAATTGTAAAAATATTCCCCAAGAAGAA
TTACAAATTACTCAATGGATAAAAGAATGGCATGGAGAATTTTTGCTTGAAAGAGATAAT
AGATCAAAATTGCCAAAAAGTAAATGTAAAAATAATACATTATATGAAGCATGTGAGAA
GGAATGTATTGATCCATGTATGAAATATAGAGATTGGATTATTAGAAGTAAATTTGAATG
GCATACGTTATCGAAAGAATATGAAACTCAAAAAGTTTCAAAGGAAAATGCGGAAAATT
ATTTAATCAAAATTTCAGAAAACAAGAATGATGCTAAAGTAAGTTTATTATTGAATAATT
GTGATGCTGAATATTCAAAATATTGTGATTGTAAACATACTACTACTCTCGTTAAAAGCGT
TTTAAATGGTAACGACAATACAATTAAGGAAAAGCGTGAACATATTGATTTAGATGATTT
TTCTAAATTTGGATGTGATAAAAATTCCGTTGATACAAACACAAAGGTGTGGGAATGTAA
AAACCCTTATATATTATCCACTAAAGATGTATGTGTACCTCCGAGGAGGCAAGAATTATG
TCTTGGAAACATTGATAGAATATACGATAAAAACCTATTAATGATAAAAGAGCATATTCT
TGCTATTGCAATATATGAATCAAGAATATTGAAACGAAAATATAAGAATAAAGATGATAA

AGAAGTTTGTAAAATCATAAATAAAACTTTCGCTGATATAAGAGATATTATAGGAGGTAC
TGATTATTGGAATGATTTGAGCAATAGAAAATTAGTAGGAAAAATTAACACAAATTCAAA
ATATGTTCACAGGAATAAAAAAAATGATAAGCTTTTTCGTGATGAGTGGTGGAAAGTTAT
TAAAAAAGATGTATGGAATGTGATATCATGGGTATTCAAGGATAAAACTGTTTGTAAAGA
AGATGATATTGAAAATATACCACAATTCTTCAGATGGTTTAGTGAATGGGGTGATGATTA
TTGCCAGGATAAAACAAAAATG
>French Guiana (Camopi) Cmp15_EBA-175KJ419594.1
TATGTATGTATTCCTGATCGTAGAATCCAATTATGCATTGTTAATCTTAGCATTATTAAAA
CATATACAAAAGAGACCATGAAGGATCATTTCATTGAAGCCTCTAAAAAAGAATCTCAA
CTTTTGCTTAAAAAAAAATGATAACGAATATAATTCTAAATTTTGTAATGATTTGAAGAATA
GTTTTTTAGATTATGGACATCTTGCTATGGGAAATGATATGGATTTTGGAGGTTATTCAAC
TAAGGCAGAAAACAAAATTCAAGAAGTTTTTAAAGGGGCTCATGGGGAAATAAGTGAAC
ATAAAATTAAAAATTTTAGAAAAGAATGGTGGAATGAATTTAGAGAGAAACTTTGGGAA
GCTATGTTATCTGAGCATAAAAATAATATAAATAATTGTAAAAATATTCCCCAAGAAGAA
TTACAAATTACTCAATGGATAAAAGAATGGCATGGAGAATTTTTGCTTGAAAGATATAAT
AGATCAAAATTGCCAAAAAGTAAATGTAAAAATAATACATTATATGAAGCATGTGAGAA
GGAATGTATTGATCCATGTATGAAATATAGAGATTGGATTATTAGAAGTAAATTTGAATG
GCATACGTTATCGAAAGAATATGAAACTCAAAAAGTTTCAAAGGAAAATGCGGAAAATT
ATTTAATCAAAATTTCAGAAAACAAGAATGATGCTAAAGTAAGTTTATTATTGAATAATT
GTGATGCTGAATATTCAAAATATTGTGATTGTAAACATACTACTACTCTCGTTAAAAGCGT
TTTAAATGGTAACGACAATACAATTAAGGAAAAGCGTGAACATATTGATTTAGATGATTT
TTCTAAATTTGGATGTGATAAAAATTCCGTTGATACAAACACAAAGGTGTGGGAATGTAA
AAAACCTTATATATTATCCACTAAAGATGTATGTGTACCTCCGAGGAGGCAAGAATTATG
TCTTGGAAACATTGATAGAATATACGATAAAAACCTATTAATGATAAAAGAGCATATTCT
TGCTATTGCAATATATGAATCAAGAATATTGAAACGAAAATATAAGAATAAAGATGATAA
AGAAGTTTGTAAAATCATAAATAAAACTTTCGCTGATATAAGAGATATTATAGGAGGTAC
TGATTATTGGAATGATTTGAGCAATAGAAAATTAGTAGGAAAAATTAACACAAATTCAAA
ATATGTTCACAGGAATAAAAAAAATGATAAGCTTTTTCGTGATGAGTGGTGGAAAGTTAT
TAAAAAAGATGTATGGAATGTGATATCATGGGTATTCAAGGATAAAACTGTTTGTAAAGA
AGATGATATTGAAAATATACCACAATTCTTCAGATGGTTTAGTGAATGGGGTGATGATTA
TTGCCAGGATAAAACAAAAATG
>French Guiana (Camopi) Cmp19_EBA-175KJ419595.1
TATGTATGTATTCCTGATCGTAGAATCCAATTATGCATTGTTAATCTTAGCATTATTAAAA
CATATACAAAAGAGACCATGAAGGATCATTTCATTGAAGCCTCTAAAAAAGAATCTCAA
CTTTTGCTTAAAAAAAAATGATAACAAATATAATTCTAAATTTTGTAATGATTTGAAGAATA
GTTTTTTAGATTATGGACATCTTGCTATGGGAAATGATATGGATTTTGGAGGTTATTCAAC
TAAGGCAGAAAACAAAATTCAAGAAGTTTTTAAAGGGGCTCATGGGAAAATAAGTGAAC
ATGAAATTAAAAATTTTAGAAAAAAATGGTGGAATGAATTTAGAGAGAAACTTTGGGAA
GCTATGTTATCTGAGCATAAAAATAATATAAATAATTGTAAAAATATTCCCCAAGAAGAA
TTACAAATTACTCAATGGATAAAAGAATGGCATGGAGAATTTTTGCTTGAAAGAGATAAT
AGATCAAAATTGCCAAAAAGTAAATGTAAAAATAATACATTATATGAAGCATGTGAGAA
GGAATGTATTGATCCATGTATGAAATATAGAGATTGGATTATTAGAAGTAAATTTGAATG
GCATACGTTATCGAAAGAATATGAAACTCAAAAAGTTTCAAAGGAAAATGCGGAAAATT
ATTTAATCAAAATTTCAGAAAACAAGAATGATGCTAAAGTAAGTTTATTATTGAATAATT

GTGATGCTGAATATTCAAAATATTGTGATTGTAAACATACTACTACTCTCGTTAAAAGCGT
TTTAAATGGTAACGACAATACAATTAAGGAAAAGCGTGAACATATTGATTTAGATGATTT
TTCTAAATTTGGATGTGATAAAAATTCCGTTGATACAAACACAAAGGTGTGGGAATGTAA
AAACCCTTATATATTATCCACTAAAGATGTATGTGTACCTCCGAGGAGGCAAGAATTATG
TCTTGGAAACATTGATAGAATATACGATAAAAACCTATTAATGATAAAAGAGCATATTCT
TGCTATTGCAATATATGAATCAAGAATATTGAAACGAAAATATAAGAATAAAGATGATAA
AGAAGTTTGTAAAATCATAAATAAAACTTTCGCTGATATAAGAGATATTATAGGAGGTAC
TGATTATTGGAATGATTTGAGCAATAGAAAATTAGTAGGAAAAATTAACACAAATTCAAA
ATATGTTCACAGGAATAAAAAAAATGATAAGCTTTTTCGTGATGAGTGGTGGAAAGTTAT
TAAAAAAGATGTATGGAATGTGATATCATGGGTATTCAAGGATAAAACTGTTTGTAAAGA
AGATGATATTGAAAATATACCACAATTCTTCAGATGGTTTAGTGAATGGGGTGATGATTA
TTGCCAGGATAAAACAAAAATG
>French Guiana (Camopi) Cmp20_EBA-175KJ419596.1
TATGTATGTATTCCTGATCGTAGAATCCAATTATGCATTGTTAATCTTAGCATTATTAAAA
CATATACAAAAGAGACCATGAAGGATCATTTCATTGAAGCCTCTAAAAAAGAATCTCAA
CTTTTGCTTAAAAAAAATGATAACAAATATAATTCTAAATTTTGTAATGATTTGAAGAATA
GTTTTTTAGATTATGGACATCTTGCTATGGGAAATGATATGGATTTTGGAGGTTATTCAAC
TAAGGCAGAAAACAAAATTCAAGAAGTTTTTAAAGGGGCTCATGGGAAAATAAGTGAAC
ATGAAATTAAAAATTTTAGAAAAAAATGGTGGAATGAATTTAGAGAGAAACTTTGGGAA
GCTATGTTATCTGAGCATAAAAATAATATAAATAATTGTAAAAATATTCCCCAAGAAGAA
TTACAAATTACTCAATGGATAAAAGAATGGCATGGAGAATTTTTGCTTGAAAGAGATAAT
AGATCAAAATTGCCAAAAAGTAAATGTAAAAATAATACATTATATGAAGCATGTGAGAA
GGAATGTATTGATCCATGTATGAAATATAGAGATTGGATTATTAGAAGTAAATTTGAATG
GCATACGTTATCGAAAGAATATGAAACTCAAAAAGTTTCAAAGGAAAATGCGGAAAATT
ATTTAATCAAAATTTCAGAAAACAAGAATGATGCTAAAGTAAGTTTATTATTGAATAATT
GTGATGCTGAATATTCAAAATATTGTGATTGTAAACATACTACTACTCTCGTTAAAAGCGT
TTTAAATGGTAACGACAATACAATTAAGGAAAAGCGTGAACATATTGATTTAGATGATTT
TTCTAAATTTGGATGTGATAAAAATTCCGTTGATACAAACACAAAGGTGTGGGAATGTAA
AAACCCTTATATATTATCCACTAAAGATGTATGTGTACCTCCGAGGAGGCAAGAATTATG
TCTTGGAAACATTGATAGAATATACGATAAAAACCTATTAATGATAAAAGAGCATATTCT
TGCTATTGCAATATATGAATCAAGAATATTGAAACGAAAATATAAGAATAAAGATGATAA
AGAAGTTTGTAAAATCATAAATAAAACTTTCGCTGATATAAGAGATATTATAGGAGGTAC
TGATTATTGGAATGATTTGAGCAATAGAAAATTAGTAGGAAAAATTAACACAAATTCAAA
ATATGTTCACAGGAATAAAAAAAATGATAAGCTTTTTCGTGATGAGTGGTGGAAAGTTAT
TAAAAAAGATGTATGGAATGTGATATCATGGGTATTCAAGGATAAAACTGTTTGTAAAGA
AGATGATATTGAAAATATACCACAATTCTTCAGATGGTTTAGTGAATGGGGTGATGATTA
TTGCCAGGATAAAACAAAAATG
>French Guiana (Camopi) Cmp21_EBA-175KJ419597.1
TATGTATGTATTCCTGATCGTAGAATCCAATTATGCATTGTTAATCTTAGCATTATTAAAA
CATATACAAAAGAGACCATGAAGGATCATTTCATTGAAGCCTCTAAAAAAGAATCTCAA
CTTTTGCTTAAAAAAAATGATAACAAATATAATTCTAAATTTTGTAATGATTTGAAGAATA
GTTTTTTAGATTATGGACATCTTGCTATGGGAAATGATATGGATTTTGGAGGTTATTCAAC
TAAGGCAGAAAACAAAATTCAAGAAGTTTTTAAAGGGGCTCATGGGAAAATAAGTGAAC
ATGAAATTAAAAATTTTAGAAAAAAATGGTGGAATGAATTTAGAGAGAAACTTTGGGAA

GCTATGTTATCTGAGCATAAAAATAATATAAATAATTGTAAAAATATTCCCCAAGAAGAA
TTACAAATTACTCAATGGATAAAAGAATGGCATGGAGAATTTTTGCTTGAAAGAGATAAT
AGATCAAAATTGCCAAAAAGTAAATGTAAAAATAATACATTATATGAAGCATGTGAGAA
GGAATGTATTGATCCATGTATGAAATATAGAGATTGGATTATTAGAAGTAAATTTGAATG
GCATACGTTATCGAAAGAATATGAAACTCAAAAAGTTTCAAAGGAAAATGCGGAAAATT
ATTTAATCAAAATTTCAGAAAACAAGAATGATGCTAAAGTAAGTTTATTATTGAATAATT
GTGATGCTGAATATTCAAAATATTGTGATTGTAAACATACTACTACTCTCGTTAAAAGCGT
TTTAAATGGTAACGACAATACAATTAAGGAAAAGCGTGAACATATTGATTTAGATGATTT
TTCTAAATTTGGATGTGATAAAAATTCCGTTGATACAAACACAAAGGTGTGGGAATGTAA
AAACCCTTATATATTATCCACTAAAGATGTATGTGTACCTCCGAGGAGGCAAGAATTATG
TCTTGGAAACATTGATAGAATATACGATAAAAACCTATTAATGATAAAAGAGCATATTCT
TGCTATTGCAATATATGAATCAAGAATATTGAAACGAAAATATAAGAATAAAGATGATAA
AGAAGTTTGTAAAATCATAAATAAAACTTTCGCTGATATAAGAGATATTATAGGAGGTAC
TGATTATTGGAATGATTTGAGCAATAGAAAATTAGTAGGAAAAATTAACACAAATTCAAA
ATATGTTCACAGGAATAAAAAAAATGATAAGCTTTTTCGTGATGAGTGGTGGAAAGTTAT
TAAAAAAGATGTATGGAATGTGATATCATGGGTATTCAAGGATAAAACTGTTTGTAAAGA
AGATGATATTGAAAATATACCACAATTCTTCAGATGGTTTAGTGAATGGGGTGATGATTA
TTGCCAGGATAAAACAAAAATG
>French Guiana (Camopi) Cmp29_EBA-175KJ419598.1
TATGTATGTATTCCTGATCGTAGAATCCAATTATGCATTGTTAATCTTAGCATTATTAAAA
CATATACAAAAGAGACCATGAAGGATCATTTCATTGAAGCCTCTAAAAAAGAATCTCAA
CTTTTGCTTAAAAAAAATGATAACAAATATAATTCTAAATTTTGTAATGATTTGAAGAATA
GTTTTTTAGATTATGGACATCTTGCTATGGGAAATGATATGGATTTTGGAGGTTATTCAAC
TAAGGCAGAAAACAAAATTCAAGAAGTTTTTAAAGGGGCTCATGGGAAAATAAGTGAAC
ATGAAATTAAAAATTTTAGAAAAAAATGGTGGAATGAATTTAGAGAGAAACTTTGGGAA
GCTATGTTATCTGAGCATAAAAATAATATAAATAATTGTAAAAATATTCCCCAAGAAGAA
TTACAAATTACTCAATGGATAAAAGAATGGCATGGAGAATTTTTGCTTGAAAGAGATAAT
AGATCAAAATTGCCAAAAAGTAAATGTAAAAATAATACATTATATGAAGCATGTGAGAA
GGAATGTATTGATCCATGTATGAAATATAGAGATTGGATTATTAGAAGTAAATTTGAATG
GCATACGTTATCGAAAGAATATGAAACTCAAAAAGTTTCAAAGGAAAATGCGGAAAATT
ATTTAATCAAAATTTCAGAAAACAAGAATGATGCTAAAGTAAGTTTATTATTGAATAATT
GTGATGCTGAATATTCAAAATATTGTGATTGTAAACATACTACTACTCTCGTTAAAAGCGT
TTTAAATGGTAACGACAATACAATTAAGGAAAAGCGTGAACATATTGATTTAGATGATTT
TTCTAAATTTGGATGTGATAAAAATTCCGTTGATACAAACACAAAGGTGTGGGAATGTAA
AAACCCTTATATATTATCCACTAAAGATGTATGTGTACCTCCGAGGAGGCAAGAATTATG
TCTTGGAAACATTGATAGAATATACGATAAAAACCTATTAATGATAAAAGAGCATATTCT
TGCTATTGCAATATATGAATCAAGAATATTGAAACGAAAATATAAGAATAAAGATGATAA
AGAAGTTTGTAAAATCATAAATAAAACTTTCGCTGATATAAGAGATATTATAGGAGGTAC
TGATTATTGGAATGATTTGAGCAATAGAAAATTAGTAGGAAAAATTAACACAAATTCAAA
ATATGTTCACAGGAATAAAAAAAATGATAAGCTTTTTCGTGATGAGTGGTGGAAAGTTAT
TAAAAAAGATGTATGGAATGTGATATCATGGGTATTCAAGGATAAAACTGTTTGTAAAGA
AGATGATATTGAAAATATACCACAATTCTTCAGATGGTTTAGTGAATGGGGTGATGATTA
TTGCCAGGATAAAACAAAAATG
>French Guiana (Camopi) Cmp30_EBA-175KJ419599.1

TATGTATGTATTCCTGATCGTAGAATCCAATTATGCATTGTTAATCTTAGCATTATTAAAA
CATATACAAAAGAGACCATGAAGGATCATTTCATTGAAGCCTCTAAAAAAGAATCTCAA
CTTTTGCTTAAAAAAAAATGATAACAAATATAATTCTAAATTTTGTAATGATTTGAAGAATA
GTTTTTTAGATTATGGACATCTTGCTATGGGAAATGATATGGATTTTGGAGGTTATTCAAC
TAAGGCAGAAAACAAAATTCAAGAAGTTTTTAAAGGGGCTCATGGGAAAATAAGTGAAC
ATGAAATTAAAAATTTTAGAAAAAAATGGTGGAATGAATTTAGAGAGAAACTTTGGGAA
GCTATGTTATCTGAGCATAAAAATAATATAAATAATTGTAAAAATATTCCCCAAGAAGAA
TTACAAATTACTCAATGGATAAAAGAATGGCATGGAGAATTTTTGCTTGAAAGAGATAAT
AGATCAAAATTGCCAAAAAGTAAATGTAAAAATAATACATTATATGAAGCATGTGAGAA
GGAATGTATTGATCCATGTATGAAATATAGAGATTGGATTATTAGAAGTAAATTTGAATG
GCATACGTTATCGAAAGAATATGAAACTCAAAAAGTTTCAAAGGAAAATGCGGAAAATT
ATTTAATCAAAATTTCAGAAAACAAGAATGATGCTAAAGTAAGTTTATTATTGAATAATT
GTGATGCTGAATATTCAAAATATTGTGATTGTAAACATACTACTACTCTCGTTAAAAGCGT
TTTAAATGGTAACGACAATACAATTAAGGAAAAGCGTGAACATATTGATTTAGATGATTT
TTCTAAATTTGGATGTGATAAAAATTCCGTTGATACAAACACAAAGGTGTGGGAATGTAA
AAACCCTTATATATTATCCACTAAAGATGTATGTGTACCTCCGAGGAGGCAAGAATTATG
TCTTGGAAACATTGATAGAATATACGATAAAAACCTATTAATGATAAAAGAGCATATTCT
TGCTATTGCAATATATGAATCAAGAATATTGAAACGAAAATATAAGAATAAAGATGATAA
AGAAGTTTGTAAAATCATAAATAAAACTTTCGCTGATATAAGAGATATTATAGGAGGTAC
TGATTATTGGAATGATTTGAGCAATAGAAAATTAGTAGGAAAAATTAACACAAATTCAAA
ATATGTTCACAGGAATAAAAAAAATGATAAGCTTTTTCGTGATGAGTGGTGGAAAGTTAT
TAAAAAAGATGTATGGAATGTGATATCATGGGTATTCAAGGATAAAACTGTTTGTAAAGA
AGATGATATTGAAAATATACCACAATTCTTCAGATGGTTTAGTGAATGGGGTGATGATTA
TTGCCAGGATAAAACAAAAATG
>French Guiana (Camopi) Cmp35_EBA-175KJ419600.1
TATGTATGTATTCCTGATCGTAGAATCCAATTATGCATTGTTAATCTTAGCATTATTAAAA
CATATACAAAAGAGACCATGAAGGATCATTTCATTGAAGCCTCTAAAAAAGAATCTCAA
CTTTTGCTTAAAAAAAAATGATAACAAATATAATTCTAAATTTTGTAATGATTTGAAGAATA
GTTTTTTAGATTATGGACATCTTGCTATGGGAAATGATATGGATTTTGGAGGTTATTCAAC
TAAGGCAGAAAACAAAATTCAAGAAGTTTTTAAAGGGGCTCATGGGAAAATAAGTGAAC
ATGAAATTAAAAATTTTAGAAAAAAATGGTGGAATGAATTTAGAGAGAAACTTTGGGAA
GCTATGTTATCTGAGCATAAAAATAATATAAATAATTGTAAAAATATTCCCCAAGAAGAA
TTACAAATTACTCAATGGATAAAAGAATGGCATGGAGAATTTTTGCTTGAAAGAGATAAT
AGATCAAAATTGCCAAAAAGTAAATGTAAAAATAATACATTATATGAAGCATGTGAGAA
GGAATGTATTGATCCATGTATGAAATATAGAGATTGGATTATTAGAAGTAAATTTGAATG
GCATACGTTATCGAAAGAATATGAAACTCAAAAAGTTTCAAAGGAAAATGCGGAAAATT
ATTTAATCAAAATTTCAGAAAACAAGAATGATGCTAAAGTAAGTTTATTATTGAATAATT
GTGATGCTGAATATTCAAAATATTGTGATTGTAAACATACTACTACTCTCGTTAAAAGCGT
TTTAAATGGTAACGACAATACAATTAAGGAAAAGCGTGAACATATTGATTTAGATGATTT
TTCTAAATTTGGATGTGATAAAAATTCCGTTGATACAAACACAAAGGTGTGGGAATGTAA
AAACCCTTATATATTATCCACTAAAGATGTATGTGTACCTCCGAGGAGGCAAGAATTATG
TCTTGGAAACATTGATAGAATATACGATAAAAACCTATTAATGATAAAAGAGCATATTCT
TGCTATTGCAATATATGAATCAAGAATATTGAAACGAAAATATAAGAATAAAGATGATAA
AGAAGTTTGTAAAATCATAAATAAAACTTTCGCTGATATAAGAGATATTATAGGAGGTAC

TGATTATTGGAATGATTTGAGCAATAGAAAATTAGTAGGAAAAATTAACACAAATTCAAA
ATATGTTCACAGGAATAAAAAAAATGATAAGCTTTTTCGTGATGAGTGGTGGAAAGTTAT
TAAAAAAGATGTATGGAATGTGATATCATGGGTATTCAAGGATAAAACTGTTTGTAAAGA
AGATGATATTGAAAATATACCACAATTCTTCAGATGGTTTAGTGAATGGGGTGATGATTA
TTGCCAGGATAAAACAAAAATG
>French Guiana (Camopi) Cmp36_EBA-175KJ419601.1
TATGTATGTATTCCTGATCGTAGAATCCAATTATGCATTGTTAATCTTAGCATTATTAAAA
CATATACAAAAGAGACCATGAAGGATCATTTCATTGAAGCCTCTAAAAAAGAATCTCAA
CTTTTGCTTAAAAAAAAATGATAACAAATATAATTCTAAATTTTGTAATGATTTGAAGAATA
GTTTTTTAGATTATGGACATCTTGCTATGGGAAATGATATGGATTTTGGAGGTTATTCAAC
TAAGGCAGAAAACAAAATTCAAGAAGTTTTTAAAGGGGCTCATGGGAAAATAAGTGAAC
ATGAAATTAAAAATTTTAGAAAAAAATGGTGGAATGAATTTAGAGAGAAACTTTGGGAA
GCTATGTTATCTGAGCATAAAAATAATATAAATAATTGTAAAAATATTCCCCAAGAAGAA
TTACAAATTACTCAATGGATAAAAGAATGGCATGGAGAATTTTTGCTTGAAAGAGATAAT
AGATCAAAATTGCCAAAAAGTAAATGTAAAAATAATACATTATATGAAGCATGTGAGAA
GGAATGTATTGATCCATGTATGAAATATAGAGATTGGATTATTAGAAGTAAATTTGAATG
GCATACGTTATCGAAAGAATATGAAACTCAAAAAGTTTCAAAGGAAAATGCGGAAAATT
ATTTAATCAAAATTTCAGAAAACAAGAATGATGCTAAAGTAAGTTTATTATTGAATAATT
GTGATGCTGAATATTCAAAATATTGTGATTGTAAACATACTACTACTCTCGTTAAAAGCGT
TTTAAATGGTAACGACAATACAATTAAGGAAAAGCGTGAACATATTGATTTAGATGATTT
TTCTAAATTTGGATGTGATAAAAATTCCGTTGATACAAACACAAAGGTGTGGGAATGTAA
AAACCCTTATATATTATCCACTAAAGATGTATGTGTACCTCCGAGGAGGCAAGAATTATG
TCTTGGAAACATTGATAGAATATACGATAAAAACCTATTAATGATAAAAGAGCATATTCT
TGCTATTGCAATATATGAATCAAGAATATTGAAACGAAAATATAAGAATAAAGATGATAA
AGAAGTTTGTAAAATCATAAATAAAACTTTCGCTGATATAAGAGATATTATAGGAGGTAC
TGATTATTGGAATGATTTGAGCAATAGAAAATTAGTAGGAAAAATTAACACAAATTCAAA
ATATGTTCACAGGAATAAAAAAAATGATAAGCTTTTTCGTGATGAGTGGTGGAAAGTTAT
TAAAAAAGATGTATGGAATGTGATATCATGGGTATTCAAGGATAAAACTGTTTGTAAAGA
AGATGATATTGAAAATATACCACAATTCTTCAGATGGTTTAGTGAATGGGGTGATGATTA
TTGCCAGGATAAAACAAAAATG
>French Guiana (Camopi) Cmp37_EBA-175KJ419602.1
TATGTATGTATTCCTGATCGTAGAATCCAATTATGCATTGTTAATCTTAGCATTATTAAAA
CATATACAAAAGAGACCATGAAGGATCATTTCATTGAAGCCTCTAAAAAAGAATCTCAA
CTTTTGCTTAAAAAAAAATGATAACAAATATAATTCTAAATTTTGTAATGATTTGAAGAATA
GTTTTTTAGATTATGGACATCTTGCTATGGGAAATGATATGGATTTTGGAGGTTATTCAAC
TAAGGCAGAAAACAAAATTCAAGAAGTTTTTAAAGGGGCTCATGGGAAAATAAGTGAAC
ATGAAATTAAAAATTTTAGAAAAAAATGGTGGAATGAATTTAGAGAGAAACTTTGGGAA
GCTATGTTATCTGAGCATAAAAATAATATAAATAATTGTAAAAATATTCCCCAAGAAGAA
TTACAAATTACTCAATGGATAAAAGAATGGCATGGAGAATTTTTGCTTGAAAGAGATAAT
AGATCAAAATTGCCAAAAAGTAAATGTAAAAATAATACATTATATGAAGCATGTGAGAA
GGAATGTATTGATCCATGTATGAAATATAGAGATTGGATTATTAGAAGTAAATTTGAATG
GCATACGTTATCGAAAGAATATGAAACTCAAAAAGTTTCAAAGGAAAATGCGGAAAATT
ATTTAATCAAAATTTCAGAAAACAAGAATGATGCTAAAGTAAGTTTATTATTGAATAATT
GTGATGCTGAATATTCAAAATATTGTGATTGTAAACATACTACTACTCTCGTTAAAAGCGT

TTTAAATGGTAACGACAATACAATTAAGGAAAAGCGTGAACATATTGATTTAGATGATTT
TTCTAAATTTGGATGTGATAAAAATTCCGTTGATACAAACACAAAGGTGTGGGAATGTAA
AAACCCTTATATATTATCCACTAAAGATGTATGTGTACCTCCGAGGAGGCAAGAATTATG
TCTTGGAAACATTGATAGAATATACGATAAAAACCTATTAATGATAAAAGAGCATATTCT
TGCTATTGCAATATATGAATCAAGAATATTGAAACGAAAATATAAGAATAAAGATGATAA
AGAAGTTTGTAAAATCATAAATAAAACTTTCGCTGATATAAGAGATATTATAGGAGGTAC
TGATTATTGGAATGATTTGAGCAATAGAAAATTAGTAGGAAAAATTAACACAAATTCAAA
ATATGTTCACAGGAATAAAAAAAATGATAAGCTTTTTCGTGATGAGTGGTGGAAAGTTAT
TAAAAAAGATGTATGGAATGTGATATCATGGGTATTCAAGGATAAAACTGTTTGTAAAGA
AGATGATATTGAAAATATACCACAATTCTTCAGATGGTTTAGTGAATGGGGTGATGATTA
TTGCCAGGATAAAACAAAAATG
>French Guiana (Camopi) Cmp43_EBA-175KJ419603.1
TATGTATGTATTCCTGATCGTAGAATCCAATTATGCATTGTTAATCTTAGCATTATTAAAA
CATATACAAAAGAGACCATGAAGGATCATTTCATTGAAGCCTCTAAAAAAGAATCTCAA
CTTTTGCTTAAAAAAAAATGATAACAAATATAATTCTAAATTTTGTAATGATTTGAAGAATA
GTTTTTTAGATTATGGACATCTTGCTATGGGAAATGATATGGATTTTGGAGGTTATTCAAC
TAAGGCAGAAAACAAAATTCAAGAAGTTTTTAAAGGGGCTCATGGGAAAATAAGTGAAC
ATGAAATTAAAAATTTTAGAAAAAAATGGTGGAATGAATTTAGAGAGAAACTTTGGGAA
GCTATGTTATCTGAGCATAAAAATAATATAAATAATTGTAAAAATATTCCCCAAGAAGAA
TTACAAATTACTCAATGGATAAAAGAATGGCATGGAGAATTTTTGCTTGAAAGAGATAAT
AGATCAAAATTGCCAAAAAGTAAATGTAAAAATAATACATTATATGAAGCATGTGAGAA
GGAATGTATTGATCCATGTATGAAATATAGAGATTGGATTATTAGAAGTAAATTTGAATG
GCATACGTTATCGAAAGAATATGAAACTCAAAAAGTTCCAAAGGAAAATGCGGAAAATT
ATTTAATCAAAATTTCAGAAAACAAGAATGATGCTAAAGTAAGTTTATTATTGAATAATT
GTGATGCTGAATATTCAAAATATTGTGATTGTAAACATACTACTACTCTCGTTAAAAGCGT
TTTAAATGGTAACGACAATACAATTAAGGAAAAGCGTGAACATATTGATTTAGATGATTT
TTCTAAATTTGGATGTGATAAAAATTCCGTTGATACAAACACAAAGGTGTGGGAATGTAA
AAACCCTTATATATTATCCACTAAAGATGTATGTGTACCTCCGAGGAGGCAAGAATTATG
TCTTGGAAACATTGATAGAATATACGATAAAAACCTATTAATGATAAAAGAGCATATTCT
TGCTATTGCAATATATGAATCAAGAATATTGAAACGAAAATATAAGAATAAAGATGATAA
AGAAGTTTGTAAAATCATAAATAAAACTTTCGCTGATATAAGAGATATTATAGGAGGTAC
TGATTATTGGAATGATTTGAGCAATAGAAAATTAGTAGGAAAAATTAACACAAATTCAAA
ATATGTTCACAGGAATAAAAAAAATGATAAGCTTTTTCGTGATGAGTGGTGGAAAGTTAT
TAAAAAAGATGTATGGAATGTGATATCATGGGTATTCAAGGATAAAACTGTTTGTAAAGA
AGATGATATTGAAAATATACCACAATTCTTCAGATGGTTTAGTGAATGGGGTGATGATTA
TTGCCAGGATAAAACAAAAATG
>French Guiana (Camopi) Cmp44_EBA-175KJ419604.1
TATGTATGTATTCCTGATCGTAGAATCCAATTATGCATTGTTAATCTTAGCATTATTAAAA
CATATACAAAAGAGACCATGAAGGATCATTTCATTGAAGCCTCTAAAAAAGAATCTCAA
CTTTTGCTTAAAAAAAAATGATAACAAATATAATTCTAAATTTTGTAATGATTTGAAGAATA
GTTTTTTAGATTATGGACATCTTGCTATGGGAAATGATATGGATTTTGGAGGTTATTCAAC
TAAGGCAGAAAACAAAATTCAAGAAGTTTTTAAAGGGGCTCATGGGAAAATAAGTGAAC
ATGAAATTAAAAATTTTAGAAAAAAATGGTGGAATGAATTTAGAGAGAAACTTTGGGAA
GCTATGTTATCTGAGCATAAAAATAATATAAATAATTGTAAAAATATTCCCCAAGAAGAA

TTACAAATTACTCAATGGATAAAAGAATGGCATGGAGAATTTTTGCTTGAAAGAGATAAT
AGATCAAAATTGCCAAAAAGTAAATGTAAAAATAATACATTATATGAAGCATGTGAGAA
GGAATGTATTGATCCATGTATGAAATATAGAGATTGGATTATTAGAAGTAAATTTGAATG
GCATACGTTATCGAAAGAATATGAAACTCAAAAAGTTTCAAAGGAAAATGCGGAAAATT
ATTTAATCAAAATTTCAGAAAACAAGAATGATGCTAAAGTAAGTTTATTATTGAATAATT
GTGATGCTGAATATTCAAAATATTGTGATTGTAAACATACTACTACTCTCGTTAAAAGCGT
TTTAAATGGTAACGACAATACAATTAAGGAAAAGCGTGAACATATTGATTTAGATGATTT
TTCTAAATTTGGATGTGATAAAAAATTCCGTTGATACAAACACAAAGGTGTGGGAATGTAA
AAACCCTTATATATTATCCACTAAAGATGTATGTGTACCTCCGAGGAGGCAAGAATTATG
TCTTGGAAACATTGATAGAATATACGATAAAAACCTATTAATGATAAAAGAGCATATTCT
TGCTATTGCAATATATGAATCAAGAATATTGAAACGAAAATATAAGAATAAAGATGATAA
AGAAGTTTGTAAAATCATAAATAAAACTTTCGCTGATATAAGAGATATTATAGGAGGTAC
TGATTATTGGAATGATTTGAGCAATAGAAAATTAGTAGGAAAAATTAACACAAATTCAAA
ATATGTTCACAGGAATAAAAAAAATGATAAGCTTTTTCGTGATGAGTGGTGGAAAGTTAT
TAAAAAAGATGTATGGAATGTGATATCATGGGTATTCAAGGATAAAACTGTTTGTAAAGA
AGATGATATTGAAAATATACCACAATTCTTCAGATGGTTTAGTGAATGGGGTGATGATTA
TTGCCAGGATAAAACAAAAATG
>French Guiana (Camopi) Cmp47_EBA-175KJ419605.1
TATGTATGTATTCCTGATCGTAGAATCCAATTATGCATTGTTAATCTTAGCATTATTAAAA
CATATACAAAAGAGACCATGAAGGATCATTTCATTGAAGCCTCTAAAAAAGAATCTCAA
CTTTTGCTTAAAAAAAAATGATAACAAATATAATTCTAAATTTTGTAATGATTTGAAGAATA
GTTTTTTAGATTATGGACATCTTGCTATGGGAAATGATATGGATTTTGGAGGTTATTCAAC
TAAGGCAGAAAACAAAATTCAAGAAGTTTTTAAAGGGGCTCATGGGAAAATAAGTGAAC
ATGAAATTAAAAATTTTAGAAAAAAATGGTGGAATGAATTTAGAGAGAAACTTTGGGAA
GCTATGTTATCTGAGCATAAAAATAATATAAATAATTGTAAAAATATTCCCCAAGAAGAA
TTACAAATTACTCAATGGATAAAAGAATGGCATGGAGAATTTTTGCTTGAAAGAGATAAT
AGATCAAAATTGCCAAAAAGTAAATGTAAAAATAATACATTATATGAAGCATGTGAGAA
GGAATGTATTGATCCATGTATGAAATATAGAGATTGGATTATTAGAAGTAAATTTGAATG
GCATACGTTATCGAAAGAATATGAAACTCAAAAAGTTTCAAAGGAAAATGCGGAAAATT
ATTTAATCAAAATTTCAGAAAACAAGAATGATGCTAAAGTAAGTTTATTATTGAATAATT
GTGATGCTGAATATTCAAAATATTGTGATTGTAAACATACTACTACTCTCGTTAAAAGCGT
TTTAAATGGTAACGACAATACAATTAAGGAAAAGCGTGAACATATTGATTTAGATGATTT
TTCTAAATTTGGATGTGATAAAAAATTCCGTTGATACAAACACAAAGGTGTGGGAATGTAA
AAACCCTTATATATTATCCACTAAAGATGTATGTGTACCTCCGAGGAGGCAAGAATTATG
TCTTGGAAACATTGATAGAATATACGATAAAAACCTATTAATGATAAAAGAGCATATTCT
TGCTATTGCAATATATGAATCAAGAATATTGAAACGAAAATATAAGAATAAAGATGATAA
AGAAGTTTGTAAAATCATAAATAAAACTTTCGCTGATATAAGAGATATTATAGGAGGTAC
TGATTATTGGAATGATTTGAGCAATAGAAAATTAGTAGGAAAAATTAACACAAATTCAAA
ATATGTTCACAGGAATAAAAAAAATGATAAGCTTTTTCGTGATGAGTGGTGGAAAGTTAT
TAAAAAAGATGTATGGAATGTGATATCATGGGTATTCAAGGATAAAACTGTTTGTAAAGA
AGATGATATTGAAAATATACCACAATTCTTCAGATGGTTTAGTGAATGGGGTGATGATTA
TTGCCAGGATAAAACAAAAATG
>French Guiana (Camopi) Cmp51_EBA-175KJ419606.1
TATGTATGTATTCCTGATCGTAGAATCCAATTATGCATTGTTAATCTTAGCATTATTAAAA

CATATACAAAAGAGACCATGAAGGATCATTTCATTGAAGCCTCTAAAAAAGAATCTCAA
CTTTTGCTTAAAAAAAAATGATAACAAATATAATTCTAAATTTTGTAATGATTTGAAGAATA
GTTTTTTAGATTATGGACATCTTGCTATGGGAAATGATATGGATTTTGGAGGTTATTCAAC
TAAGGCAGAAAACAAAATTCAAGAAGTTTTTAAAGGGGCTCATGGGAAAATAAGTGAAC
ATGAAATTAAAAATTTTAGAAAAAAATGGTGGAATGAATTTAGAGAGAAACTTTGGGAA
GCTATGTTATCTGAGCATAAAAATAATATAAATAATTGTAAAAATATTCCCCAAGAAGAA
TTACAAATTACTCAATGGATAAAAGAATGGCATGGAGAATTTTTGCTTGAAAGAGATAAT
AGATCAAAATTGCCAAAAAGTAAATGTAAAAATAATACATTATATGAAGCATGTGAGAA
GGAATGTATTGATCCATGTATGAAATATAGAGATTGGATTATTAGAAGTAAATTTGAATG
GCATACGTTATCGAAAGAATATGAAACTCAAAAAGTTTCAAAGGAAAATGCGGAAAATT
ATTTAATCAAAATTTCAGAAAACAAGAATGATGCTAAAGTAAGTTTATTATTGAATAATT
GTGATGCTGAATATTCAAAATATTGTGATTGTAAACATACTACTACTCTCGTTAAAAGCGT
TTTAAATGGTAACGACAATACAATTAAGGAAAAGCGTGAACATATTGATTTAGATGATTT
TTCTAAATTTGGATGTGATAAAAATTCCGTTGATACAAACACAAAGGTGTGGGAATGTAA
AAACCCTTATATATTATCCACTAAAGATGTATGTGTACCTCCGAGGAGGCAAGAATTATG
TCTTGGAAACATTGATAGAATATACGATAAAAACCTATTAATGATAAAAGAGCATATTCT
TGCTATTGCAATATATGAATCAAGAATATTGAAACGAAAATATAAGAATAAAGATGATAA
AGAAGTTTGTAAAATCATAAATAAAACTTTCGCTGATATAAGAGATATTATAGGAGGTAC
TGATTATTGGAATGATTTGAGCAATAGAAAATTAGTAGGAAAAATTAACACAAATTCAAA
ATATGTTCACAGGAATAAAAAAAATGATAAGCTTTTTCGTGATGAGTGGTGGAAAGTTAT
TAAAAAAGATGTATGGAATGTGATATCATGGGTATTCAAGGATAAAACTGTTTGTAAAGA
AGATGATATTGAAAATATACCACAATTCTTCAGATGGTTTAGTGAATGGGGTGATGATTA
TTGCCAGGATAAAACAAAAATG
>French Guiana (Camopi) Cmp52_EBA-175KJ419607.1
TATGTATGTATTCCTGATCGTAGAATCCAATTATGCATTGTTAATCTTAGCATTATTAAAA
CATATACAAAAGAGACCATGAAGGATCATTTCATTGAAGCCTCTAAAAAAGAATCTCAA
CTTTTGCTTAAAAAAAAATGATAACAAATATAATTCTAAATTTTGTAATGATTTGAAGAATA
GTTTTTTAGATTATGGACATCTTGCTATGGGAAATGATATGGATTTTGGAGGTTATTCAAC
TAAGGCAGAAAACAAAATTCAAGAAGTTTTTAAAGGGGCTCATGGGAAAATAAGTGAAC
ATGAAATTAAAAATTTTAGAAAAAAATGGTGGAATGAATTTAGAGAGAAACTTTGGGAA
GCTATGTTATCTGAGCATAAAAATAATATAAATAATTGTAAAAATATTCCCCAAGAAGAA
TTACAAATTACTCAATGGATAAAAGAATGGCATGGAGAATTTTTGCTTGAAAGAGATAAT
AGATCAAAATTGCCAAAAAGTAAATGTAAAAATAATACATTATATGAAGCATGTGAGAA
GGAATGTATTGATCCATGTATGAAATATAGAGATTGGATTATTAGAAGTAAATTTGAATG
GCATACGTTATCGAAAGAATATGAAACTCAAAAAGTTTCAAAGGAAAATGCGGAAAATT
ATTTAATCAAAATTTCAGAAAACAAGAATGATGCTAAAGTAAGTTTATTATTGAATAATT
GTGATGCTGAATATTCAAAATATTGTGATTGTAAACATACTACTACTCTCGTTAAAAGCGT
TTTAAATGGTAACGACAATACAATTAAGGAAAAGCGTGAACATATTGATTTAGATGATTT
TTCTAAATTTGGATGTGATAAAAATTCCGTTGATACAAACACAAAGGTGTGGGAATGTAA
AAACCCTTATATATTATCCACTAAAGATGTATGTGTACCTCCGAGGAGGCAAGAATTATG
TCTTGGAAACATTGATAGAATATACGATAAAAACCTATTAATGATAAAAGAGCATATTCT
TGCTATTGCAATATATGAATCAAGAATATTGAAACGAAAATATAAGAATAAAGATGATAA
AGAAGTTTGTAAAATCATAAATAAAACTTTCGCTGATATAAGAGATATTATAGGAGGTAC
TGATTATTGGAATGATTTGAGCAATAGAAAATTAGTAGGAAAAATTAACACAAATTCAAA

ATATGTTCACAGGAATAAAAAAAATGATAAGCTTTTTCGTGATGAGTGGTGGAAAGTTAT
TAAAAAAGATGTATGGAATGTGATATCATGGGTATTCAAGGATAAAACTGTTTGTAAAGA
AGATGATATTGAAAATATACCACAATTCTTCAGATGGTTTAGTGAATGGGGTGATGATTA
TTGCCAGGATAAAACAAAAATG
>French Guiana (Camopi) Cmp55_EBA-175KJ419608.1
TATGTATGTATTCCTGATCGTAGAATCCAATTATGCATTGTTAATCTTAGCATTATTAAAA
CATATACAAAAGAGACCATGAAGGATCATTTCATTGAAGCCTCTAAAAAAGAATCTCAA
CTTTTGCTTAAAAAAAATGATAACAAATATAATTCTAAATTTTGTAATGATTTGAAGAATA
GTTTTTTAGATTATGGACATCTTGCTATGGGAAATGATATGGATTTTGGAGGTTATTCAAC
TAAGGCAGAAAACAAAATTCAAGAAGTTTTTAAAGGGGCTCATGGGAAAATAAGTGAAC
ATGAAATTAAAAATTTTAGAAAAAAATGGTGGAATGAATTTAGAGAGAAACTTTGGGAA
GCTATGTTATCTGAGCATAAAAATAATATAAATAATTGTAAAAATATTCCCCAAGAAGAA
TTACAAATTACTCAATGGATAAAAGAATGGCATGGAGAATTTTTGCTTGAAAGAGATAAT
AGATCAAAATTGCCAAAAAGTAAATGTAAAAATAATACATTATATGAAGCATGTGAGAA
GGAATGTATTGATCCATGTATGAAATATAGAGATTGGATTATTAGAAGTAAATTTGAATG
GCATACGTTATCGAAAGAATATGAAACTCAAAAAGTTCCAAAGGAAAATGCGGAAAATT
ATTTAATCAAAATTTCAGAAAACAAGAATGATGCTAAAGTAAGTTTATTATTGAATAATT
GTGATGCTGAATATTCAAAATATTGTGATTGTAAACATACTACTACTCTCGTTAAAAGCGT
TTTAAATGGTAACGACAATACAATTAAGGAAAAGCGTGAACATATTGATTTAGATGATTT
TTCTAAATTTGGATGTGATAAAAATTCCGTTGATACAAACACAAAGGTGTGGGAATGTAA
AAACCCTTATATATTATCCACTAAAGATGTATGTGTACCTCCGAGGAGGCAAGAATTATG
TCTTGGAAACATTGATAGAATATACGATAAAAACCTATTAATGATAAAAGAGCATATTCT
TGCTATTGCAATATATGAATCAAGAATATTGAAACGAAAATATAAGAATAAAGATGATAA
AGAAGTTTGTAAAATCATAAATAAAACTTTCGCTGATATAAGAGATATTATAGGAGGTAC
TGATTATTGGAATGATTTGAGCAATAGAAAATTAGTAGGAAAAATTAACACAAATTCAAA
ATATGTTCACAGGAATAAAAAAAATGATAAGCTTTTTCGTGATGAGTGGTGGAAAGTTAT
TAAAAAAGATGTATGGAATGTGATATCATGGGTATTCAAGGATAAAACTGTTTGTAAAGA
AGATGATATTGAAAATATACCACAATTCTTCAGATGGTTTAGTGAATGGGGTGATGATTA
TTGCCAGGATAAAACAAAAATG
>Nigeria NIG34_EBA175AJ438799.1
TATGTATGTATTCCTGATCGTAGAATCCAATTATGCATTGTTAATCTTAGCATTATTAAAA
CATATACAAAAGAGACCATGAAGGATCATTTCATTGAAGCCTCTAAAAAAGAATCTCAA
CTTTTGCTTAAAAAAAATGATAACAAATATAATTCTAAATTTTGTAATGATTTGAAGAATA
GTTTTTTAGATTATGGACATCTTGCTATGGGAAATGATATGGATTTTGGAGGTTATTCAAC
TAAGGCAGAAAACAAAATTCAAGAAGTTTTTAAAGGGGCTCATGGGGAAATAAGTGAAC
ATGAAATTAAAAATTTTAGAAAAAAATGGTGGAATGAATTTAGAGAGAAACTTTGGGAA
GCTATGTTATCTGAGCATAAAAATAATATAAATAATTGTAAAAATATTCCCCAAGAAGAA
TTACAAATTACTCAATGGATAAAAGAATGGCATGGAGAATTTTTGCTTGAAAGAGATAAT
AGATCAAAATTGCCAAAAAGTAAATGTAAAAATAATACATTATATGAAGCATGTGAGAA
GGAATGTATTGATCCATGTATGAAATATAGAGATTGGATTATTAGAAGTAAATTTGAATG
GCATACGTTATCGAAAGAATATGAAACTCAAAAAGTTTCAAAGGAAAATGCGGAAAATT
ATTTAATCAAAATTTCAGAAAACAAGAATGATGCTAAAGTAAGTTTATTATTGAATAATT
GTGATGCTGAATATTCAAAATATTGTGATTGTAAACATACTACTACTCTCGTTAAAAGCGT
TTTAAATGGTAACGACAATACAATTAAGGAAAAGCGTGAACATATTGATTTAGATGATTT

TTCTAAATTTGGATGTGATAAAAATTCCGTTGATACAAACACAAAGGTGTGGGAATGTAA
AAAACCTTATATATTATCCACTAAAGATGTATGTGTACCTCCGAGGAGGCAAGAATTATG
TCTTGGAAACATTGATAGAATATACGATAAAAACCTATTAATGATAAAAGAGCATATTCT
TGCTATTGCAATATATGAATCAAGAATATTGAAACGAAAATATAAGAATAAAGATGATAA
AGAAGTTTGTAAAATCATAAATAAAACTTTCGCTGATATAAGAGATATTATAGGAGGTAC
TGATTATTGGAATGATTTGAGCAATAGAAAATTAGTAGGAAAAATTAACACAAATTCAAA
ATATGTTCACAGGAATAAAAAAAATGATAAGCTTTTTCGTGATGAGTGGTGGAAAGTTAT
TAAAAAAGATGTATGGAATGTGATATCATGGGTATTCAAGGATAAAACTGTTTGTAAAGA
AGATGATATTGAAAATATACCACAATTCTTCAGATGGTTTAGTGAATGGGGTGATGATTA
TTGCCAGGATAAAACAAAAATG
>Nigeria NIG210_EBA175AJ438828.1
TATGTATGTATTCCTGATCGTAGAATCCAATTATGCATTGTTAATCTTAGCATTATTAAAA
CATATACAAAAGAGACCATGAAGGATCATTTCATTGAAGCCTCTAAAAAAGAATCTCAA
CTTTTGCTTAAAAAAAATGATAACAAATATAATTCTAAATTTTGTAATGATTTGAAGAATA
GTTTTTTAGATTATGGACATCTTGCTATGGGAAATGATATGGATTTTGGAGGTTATTCAAC
TAAGGCAGAAAACAAAATTCAAGAAGTTTTTAAAGGGGCTCATGGGAAAATAAGTGAAC
ATGAAATTAAAAATTTTAGAAAAAAATGGTGGAATGAATTTAGAGAGAAACTTTGGGAA
GCTATGTTATCTGAGCATAAAAATAATATAAATAATTGTAAAAATATTCCCCAAGAAGAA
TTACAAATTACTCAATGGATAAAAGAATGGCATGGAGAATTTTTGCTTGAAAGAGATAAT
AGATCAAAATTGCCAAAAAGTAAATGTAAAAATAATACATTATATGAAGCATGTGAGAA
GGAATGTATTGATCCATGTATGAAATATAGAGATTGGATTATTAGAAGTAAATTTGAATG
GCATACGTTATCGAAAGAATATGAAACTCAAAATGTTTCAAAGGAAAATGCGGAAAATT
ATTTAATCAAAATTTCAGAAAAAATGAATGATGCTAAAGTAAGTTTATTATTGAATAATTG
TGATGCTGAATATTCAAAATATTGTGATTGTAAACATACTACTACTCTCGTTAAAAGCGTT
TTAAATGGTAACGACAATACAATTAAGGAAAAGCGTGAACATATTGATTTAGATGATTTT
TCTAAATTTGGATGTGATAAAAATTCCGTTGATACAAACACAAAGGTGTGGGAATGTAAA
AAACCTTATAAATTATCCACTAAAGATGTATGTGTACCTCCGAGGAGGCAAGAATTATGT
CTTGGAAACATTGATAGAATATACGATAAAAACCTATTAATGATAAAAGAGCATATTCTT
GCTATTGCAATATATGAATCAAGAATATTGAAACGAAAATATAAGAATAAAGATGATAA
AGAAGTTTGTAAAATCATAAATAAAACTTTCGCTGATATAAGAGATATTATAGGAGGTAC
TGATTATTGGAATGATTTGAGCAATAGAAAATTAGTAGGAAAAATTAACACAAATTCAAA
TTATGTTCACAGGAATAAAGAAAATGATAAGCTTTTTCGTGATGAGTGGTGGAAAGTTAT
TAAAAAAGATGTATGGAATGTGATATCATGGGTATTCAAGGATAAAACTGTTTGTAAAGA
AGATGATATTGAAAATATACCACAATTCTTCAGATGGTTTAGTGAATGGGGTGATGATTA
TTGCCAGGATAAAACAAAAATG
>Nigeria NIG203_EBA175AJ438827.1
TATGTATGTATTCCTGATCGTAGAATCCAATTATGCATTGTTAATCTTAGCATTATTAAAA
CATATACAAAAGAGACCATGAAGGATCATTTCATTGAAGCCTCTAAAAAAGAATCTCAA
CTTTTGCTTAAAAAAAATGATAACAAATATAATTCTAAATTTTGTAATGATTTGAAGAATA
GTTTTTTAGATTATGGACATCTTGCTATGGGAAATGATATGGATTTTGGAGGTTATTCAAC
TAAGGCAGAAAACAAAATTCAAGAAGTTTTTAAAGGGGCTCATGGGGAAATAAGTGAAC
ATGAAATTAAAAATTTTAGAAAAAAATGGTGGAATGAATTTAGAGAGAAACTTTGGGAA
GCTATGTTATCTGAGCATAAAAATAATATAAATAATTGTAAAAATATTCCCCAAGAAGAA
TTACAAATTACTCAATGGATAAAAGAATGGCATGGAGAATTTTTGCTTGAAAGAGATAAT

AGATCAAAATTGCCAAAAAGTAAATGTAAAAATAATACATTATATGAAGCATGTGAGAA
GGAATGTATTGATCCATGTATGAAATATAGAGATTGGATTATTAGAAGTAAATTTGAATG
GCATACGTTATCGAAAGAATATGAAACTCAAAAAGTTTCAAAGGAAAATGCGGAAAATT
ATTTAATCAAAATTTCAGAAAACAAGAATGATGCTAAAGTAAGTTTATTATTGAATAATT
GTGATGCTGAATATTCAAAATATTGTGATTGTAAACATACTACTACTCTCGTTAAAAGCGT
TTTAAATGGTAACGACAATACAATTAAGGAAAAGCGTGAACATATTGATTTAGATGATTT
TTCTAAATTTGGATGTGATAAAAATTCCGTTGATACAAACACAAAGGTGTGGGAATGTAA
AAAACCTTATATATTATCCACTAAAGATGTATGTGTACCTCCGAGGAGGCAAGAATTATG
TCTTGGAAACATTGATAGAATATACGATAAAAACCTATTAATGATAAAAGAGCATATTCT
TGCTATTGCAATATATGAATCAAGAATATTGAAACGAAAATATAAGAATAAAGATGATAA
AGAAGTTTGTAAAATCATAAATAAAACTTTCGCTGATATAAGAGATATTATAGGAGGTAC
TGATTATTGGAATGATTTGAGCAATAGAAAATTAGTAGGAAAAATTAACACAAATTCAAA
ATATGTTCACAGGAATAAAAAAAATGATAAGCTTTTTCGTGATGAGTGGTGGAAAGTTAT
TAAAAAAGATGTATGGAATGTGATATCATGGGTATTCAAGGATAAAACTGTTTGTAAAGA
AGATGATATTGAAAATATACCACAATTCTTCAGATGGTTTAGTGAATGGGGTGATGATTA
TTGCCAGGATAAAACAAAAATG
>Nigeria NIG199_EBA175AJ438826.1
TATGTATGTATTCCTGATCGTAGAATCCAATTATGCATTGTTAATCTTAGCATTATTAAAA
CATATACAAAAGAGACCATGAAGGATCATTTCATTGAAGCCTCTAAAAAAGAATCTCAA
CTTTTGCTTAAAAAAAATGATAACAAATATAATTCTAAATTTTGTAATGATTTGAAGAATA
GTTTTTTAGATTATGGACATCTTGCTATGGGAAATGATATGGATTTTGGAGGTTATTCAAC
TAAGGCAGAAAACAAAATTCAAGAAGTTTTTAAAGGGGCTCATGGGGAAATAAGTGAAC
ATGAAATTAAAAATTTTAGAAAAAAATGGTGGAATGAATTTAGAGAGAAACTTTGGGAA
GCTATGTTATCTGAGCATAAAAATAATATAAATAATTGTAAAAATATTCCCCAAGAAGAA
TTACAAATTACTCAATGGATAAAAGAATGGCATGGAGAATTTTTGCTTGAAAGAGATAAT
AGATCAAAATTGCCAAAAAGTAAATGTAAAAATAATACATTATATGAAGCATGTGAGAA
GGAATGTATTGATCCATGTATGAAATATAGAGATTGGATTATTAGAAGTAAATTTGAATG
GCATACGTTATCGAAAGAATATGAAACTCAAAAAGTTTCAAAGGAAAATGCGGAAAATT
ATTTAATCAAAATTTCAGAAAACAAGAATGATGCTAAAGTAAGTTTATTATTGAATAATT
GTGATGCTGAATATTCAAAATATTGTGATTGTAAACATACTACTACTCTCGTTAAAAGCGT
TTTAAATGGTAACGACAATACAATTAAGGAAAAGCGTGAACATATTGATTTAGATGATTT
TTCTAAATTTGGATGTGATAAAAATTCCGTTGATACAAACACAAAGGTGTGGGAATGTAA
AAAACCTTATATATTATCCACTAAAGATGTATGTGTACCTCCGAGGAGGCAAGAATTATG
TCTTGGAAACATTGATAGAATATACGATAAAAACCTATTAATGATAAAAGAGCATATTCT
TGCTATTGCAATATATGAATCAAGAATATTGAAACGAAAATATAAGAATAAAGATGATAA
AGAAGTTTGTAAAATCATAAATAAAACTTTCGCTGATATAAGAGATATTATAGGAGGTAC
TGATTATTGGAATGATTTGAGCAATAGAAAATTAGTAGGAAAAATTAACACAAATTCAAA
ATATGTTCACAGGAATAAAAAAAATGATAAGCTTTTTCGTGATGAGTGGTGGAAAGTTAT
TAAAAAAGATGTATGGAATGTGATATCATGGGTATTCAAGGATAAAACTGTTTGTAAAGA
AGATGATATTGAAAATATACCACAATTCTTCAGATGGTTTAGTGAATGGGGTGATGATTA
TTGCCAGGATAAAACAAAAATG
>Nigeria NIG198_EBA175AJ438825.1
TATGTATGTATTCCTGATCGTAGAATCCAATTATGCATTGTTAATCTTAGCATTATTAAAA
CATATACAAAAGAGACCATGAAGGATCATTTCATTGAAGCCTCTAAAAAAGAATCTCAA

CTTTTGCTTAAAAAAAAATGATAACAAATATAATTCTAAATTTTGTAATGATTTGAAGAATA
GTTTTTTAGATTATGGACATCTTGCTATGGGAAATGATATGGATTTTGGAGGTTATTCAAC
TAAGGCAGAAAACAAAATTCAAGAAGTTTTTAAAGGGGCTCATGGGGAAATAAGTGAAC
ATGAAATTAAAAATTTTAGAAAAAAATGGTGGAATGAATTTAGAGAGAAACTTTGGGAA
GCTATGTTATCTGAGCATAAAAATAATATAAATAATTGTAAAAATATTCCCCAAGAAGAA
TTACAAATTACTCAATGGATAAAAGAATGGCATGGAGAATTTTTGCTTGAAAGAGATAAT
AGATCAAAATTGCCAAAAAGTAAATGTAAAAATAATACATTATATGAAGCATGTGAGAA
GGAATGTATTGATCCATGTATGAAATATAGAGATTGGATTATTAGAAGTAAATTTGAATG
GCATACGTTATCGAAAGAATATGAAACTCAAAAAGTTTCAAAGGAAAATGCGGAAAATT
ATTTAATCAAAATTTCAGAAAACAAGAATGATGCTAAAGTAAGTTTATTATTGAATAATT
GTGATGCTGAATATTCAAAATATTGTGATTGTAAACATACTACTACTCTCGTTAAAAGCGT
TTTAAATGGTAACGACAATACAATTAAGGAAAAGCGTGAACATATTGATTTAGATGATTT
TTCTAAATTTGGATGTGATAAAAATTCCGTTGATACAAACACAAAGGTGTGGGAATGTAA
AAAACCTTATATATTATCCACTAAAGATGTATGTGTACCTCCGAGGAGGCAAGAATTATG
TCTTGGAAACATTGATAGAATATACGATAAAAACCTATTAATGATAAAAGAGCATATTCT
TGCTATTGCAATATATGAATCAAGAATATTGAAACGAAAATATAAGAATAAAGATGATAA
AGAAGTTTGTAAAATCATAAATAAAACTTTCGCTGATATAAGAGATATTATAGGAGGTAC
TGATTATTGGAATGATTTGAGCAATAGAAAATTAGTAGGAAAAATTAACACAAATTCAAA
ATATGTTCACAGGAATAAAAAAAATGATAAGCTTTTTCGTGATGAGTGGTGGAAAGTTAT
TAAAAAAGATGTATGGAATGTGATATCATGGGTATTCAAGGATAAAACTGTTTGTAAAGA
AGATGATATTGAAAATATACCACAATTCTTCAGATGGTTTAGTGAATGGGGTGATGATTA
TTGCCAGGATAAAACAAAAATG
>Nigeria NIG196_EBA175AJ438824.1
TATGTATGTATTCCTGATCGTAGAATCCAATTATGCATTGTTAATCTTAGCATTATTAAAA
CATATACAAAAGAGACCATGAAGGATCATTTCATTGAAGCCTCTAAAAAAGAATCTCAA
CTTTTGCTTAAAAAAAAATGATAACAAATATAATTCTAAATTTTGTAATGATTTGAAGAATA
GTTTTTTAGATTATGGACATCTTGCTATGGGAAATGATATGGATTTTGGAGGTTATTCAAC
TAAGGCAGAAAACAAAATTCAAGAAGTTTTTAAAGGGGCTCATGGGAAAATAAGTGAAC
ATGAAATTAAAAATTTTAGAAAAGAATGGTGGAATGAATTTAGAGAGAAACTTTGGGAA
GCTATGTTATCTGAGCATAAAAATAATATAAATAATTGTAAAAATATTCCCCAAGAAGAA
TTACAAATTACTCAATGGATAAAAGAATGGCATGGAGAATTTTTGCTTGAAAGAGATAAT
AGATCAAAATTGCCAAAAAGTAAATGTAAAAATAATACATTATATGAAGCATGTGAGAA
GGAATGTATTGATCCATGTATGAAATATAGAGATTGGATTATTAGAAGTAAATTTGAATG
GCATACGTTATCGAAAGAATATGAAACTCAAAATGTTTCAAAGGAAAATGCGGAAAATT
ATTTAATCAAAATTTCAGAAAAAATGAATGATGCTAAAGTAAGTTTATTATTGAATAATTG
TGATGCTGAATATTCAAAATATTGTGATTGTAAACATACTACTACTCTCGTTAAAAGCGTT
TTAAATGGTAACGACAATACAATTAAGGAAAAGCGTGAACATATTGATTTAGATGATTTT
TCTAAATTTGGATGTGATAAAAATTCCGTTGATACAAACACAAAGGTGTGGGAATGTAAA
AAACCTTATAAATTATCCACTAAAGATGTATGTGTACCTCCGAGGAGGCAAGAATTATGT
CTTGGAAACATTGATAGAATATACGATAAAAACCTATTAATGATAAAAGAGCATATTCTT
GCTATTGCAATATATGAATCAAGAATATTGAAACGAAAATATAAGAATAAAGATGATAA
AGAAGTTTGTAAAATCATAAATAAAACTTTCGCTGATATAAGAGATATTATAGGAGGTAC
TGATTATTGGAATGATTTGAGCAATAGAAAATTAGTAGGAAAAATTAACACAAATTCAAA
TTATGTTCACAGGAATAAACAAAATGATAAGCTTTTTCGTGATGCGTGGTGGAAAGTTAT

TAAAAAAGATGTATGGAATGTGATATCATGGGTATTCAAGGATAAAACTGTTTGTAAAGA
AGATGATATTGAAAATATACCACAATTCTTCAGATGGTTTAGTGAATGGGGTGATGATTA
TTGCCAGGATAAAACAAAAATG
>Nigeria NIG195_EBA175AJ438823.1
TATGTATGTATTCCTGATCGTAGAATCCAATTATGCATTGTTAATCTTAGCATTATTAAAA
CATATACAAAAGAGACCATGAAGGATCATTTCATTGAAGCCTCTAAAAAAGAATCTCAA
CTTTTGCTTAAAAAAAAATGATAACAAATATAATTCTAAATTTTGTAATGATTTGAAGAATA
GTTTTTTAGATTATGGACATCTTGCTATGGGAAATGATATGGATTTTGGAGGTTATTCAAC
TAAGGCAGAAAACAAAATTCAAGAAGTTTTTAAAGGGGCTCATGGGAAAATAAGTGAAC
ATGAAATTAAAAATTTTAGAAAAAAATGGTGGAATGAATTTAGAGAGAAACTTTGGGAA
GCTATGTTATCTGAGCATAAAAATAATATAAATAATTGTAAAAATATTCCCCAAGAAGAA
TTACAAATTACTCAATGGATAAAAGAATGGCATGGAGAATTTTTGCTTGAAAGAGATAAT
AGATCAAAATTGCCAAAAAGTAAATGTAAAAATAATACATTATATGAAGCATGTGAGAA
GGAATGTATTGATCCATGTATGAAATATAGAGATTGGATTATTAGAAGTAAATTTGAATG
GCATACGTTATCGAAAGAATATGAAACTCAAAAAGTTCCAAAGGAAAATGCGGAAAATT
ATTTAATCAAAATTTCAGAAAACAAGAATGATGCTAAAGTAAGTTTATTATTGAATAATT
GTGATGCTGAATATTCAAAATATTGTGATTGTAAACATACTACTACTCTCGTTAAAAGCGT
TTTAAATGGTAACGACAATACAATTAAGGAAAAGCGTGAACATATTGATTTAGATGATTT
TTCTAAATTTGGATGTGATAAAAATTCCGTTGATACAAACACAAAGGTGTGGGAATGTAA
AAAACCTTATAAATTATCCACTAAAGATGTATGTGTACCTCCGAGGAGGCAAGAATTATG
TCTTGGAAACATTGATAGAATATACGATAAAAACCTATTAATGATAAAAGAGCATATTCT
TGCTATTGCAATATATGAATCAAGAATATTGAAACGAAAATATAAGAATAAAGATGATAA
AGAAGTTTGTAAAATCATAAATAAAACTTTCGCTGATATAAGAGATATTATAGGAGGTAC
TGATTATTGGAATGATTTGAGCAATAGAAAATTAGTAGGAAAAATTAACACAAATTCAAA
TTATGTTCACAGGAATAAACAAAATGATAAGCTTTTTCGTGATGAGTGGTGGAAAGTTAT
TAAAAAAGATGTATGGAATGTGATATCATGGGTATTCAAGGATAAAACTGTTTGTAAAGA
AGATGATATTGAAAATATACCACAATTCTTCAGATGGTTTAGTGAATGGGGTGATGATTA
TTGCCAGGATAAAACAAAAATG
>Nigeria NIG193_EBA175AJ438822.1
TATGTATGTATTCCTGATCGTAGAATCCAATTATGCATTGTTAATCTTAGCATTATTAAAA
CATATACAAAAGAGACCATGAAGGATCATTTCATTGAAGCCTCTAAAAAAGAATCTCAA
CTTTTGCTTAAAAAAAAATGATAACGAATATAATTCTAAATTTTGTAATGATTTGAAGAATA
GTTTTTTAGATTATGGACATCTTGCTATGGGAAATGATATGGATTTTGGAGGTTATTCAAC
TAAGGCAGAAAACAAAATTCAAGAAGTTTTTAAAGGGGCTCATGGGGAAATAAGTGAAC
ATAAAATTAAAAATTTTAGAAAAGAATGGTGGAATGAATTTAGAGAGAAACTTTGGGAA
GCTATGTTATCTGAGCATAAAAATAATATAAATAATTGTAAAAATATTCCCCAAGAAGAA
TTACAAATTACTCAATGGATAAAAGAATGGCATGGAGAATTTTTGCTTGAAAGAGATAAT
AGATCAAAATTGCCAAAAAGTAAATGTAAAAATAATACATTATATGAAGCATGTGAGAA
GGAATGTATTGATCCATGTATGAAATATAGAGATTGGATTATTAGAAGTAAATTTGAATG
GCATACGTTATCGAAAGAATATGAAACTCAAAAAGTTCCAAAGGAAAATGCGGAAAATT
ATTTAATCAAAATTTCAGAAAACAAGAATGATGCTAAAGTAAGTTTATTATTGAATAATT
GTGATGCTGAATATTCAAAATATTGTGATTGTAAACATACTACTACTCTCGTTAAAAGCGT
TTTAAATGGTAACGACAATACAATTAAGGAAAAGCGTGAACATATTGATTTAGATGATTT
TTCTAAATTTGGATGTGATAAAAATTCCGTTGATACAAACACAAAGGTGTGGGAATGTAA

AAACCCTTATATATTATCCACTAAAGATGTATGTGTACCTCCGAGGAGGCAAGAATTATG
TCTTGGAAACATTGATAGAATATACGATAAAAACCTATTAATGATAAAAGAGCATATTCT
TGCTATTGCAATATATGAATCAAGAATATTGAAACGAAAATATAAGAATAAAGATGATAA
AGAAGTTTGTAAAATCATAAATAAAACTTTCGCTGATATAAGAGATATTATAGGAGGTAC
TGATTATTGGAATGATTTGAGCAATAGAAAATTAGTAGGAAAAATTAACACAAATTCAAA
ATATGTTCACAGGAATAAAAAAAATGATAAGCTTTTTCGTGATGAGTGGTGGAAAGTTAT
TAAAAAAGATGTATGGAATGTGATATCATGGGTATTCAAGGATAAAACTGTTTGTAAAGA
AGATGATATTGAAAATATACCACAATTCTTCAGATGGTTTAGTGAATGGGGTGATGATTA
TTGCCAGGATAAAACAAAAATG
>Nigeria NIG192_EBA175AJ438821.1
TATGTATGTATTCCTGATCGTAGAATCCAATTATGCATTGTTAATCTTAGCATTATTAAAA
CATATACAAAAGAGACCATGAAGGATCATTTCATTGAAGCCTCTAAAAAAGAATCTCAA
CTTTTGCTTAAAAAAAAATGATAACAAATATAATTCTAAATTTTGTAATGATTTGAAGAATA
GTTTTTTAGATTATGGACATCTTGCTATGGGAAATGATATGGATTTTGGAGGTTATTCAAC
TAAGGCAGAAAACAAAATTCAAGAAGTTTTTAAAGGGGCTCATGGGGAAATAAGTGAAC
ATGAAATTAAAAATTTTAGAAAAAAATGGTGGAATGAATTTAGAGAGAAACTTTGGGAA
GCTATGTTATCTGAGCATAAAAATAATATAAATAATTGTAAAAATATTCCCCAAGAAGAA
TTACAAATTACTCAATGGATAAAAGAATGGCATGGAGAATTTTTGCTTGAAAGAGATAAT
AGATCAAAATTGCCAAAAAGTAAATGTAAAAATAATACATTATATGAAGCATGTGAGAA
GGAATGTATTGATCCATGTATGAAATATAGAGATTGGATTATTAGAAGTAAATTTGAATG
GCATACGTTATCGAAAGAATATGAAACTCAAAAAGTTTCAAAGGAAAATGCGGAAAATT
ATTTAATCAAAATTTCAGAAAACAAGAATGATGCTAAAGTAAGTTTATTATTGAATAATT
GTGATGCTGAATATTCAAAATATTGTGATTGTAAACATACTACTACTCTCGTTAAAAGCGT
TTTAAATGGTAACGACAATACAATTAAGGAAAAGCGTGAACATATTGATTTAGATGATTT
TTCTAAATTTGGATGTGATAAAAATTCCGTTGATACAAACACAAAGGTGTGGGAATGTAA
AAAACCTTATATATTATCCACTAAAGATGTATGTGTACCTCCGAGGAGGCAAGAATTATG
TCTTGGAAACATTGATAGAATATACGATAAAAACCTATTAATGATAAAAGAGCATATTCT
TGCTATTGCAATATATGAATCAAGAATATTGAAACGAAAATATAAGAATAAAGATGATAA
AGAAGTTTGTAAAATCATAAATAAAACTTTCGCTGATATAAGAGATATTATAGGAGGTAC
TGATTATTGGAATGATTTGAGCAATAGAAAATTAGTAGGAAAAATTAACACAAATTCAAA
ATATGTTCACAGGAATAAAAAAAATGATAAGCTTTTTCGTGATGAGTGGTGGAAAGTTAT
TAAAAAAGATGTATGGAATGTGATATCATGGGTATTCAAGGATAAAACTGTTTGTAAAGA
AGATGATATTGAAAATATACCACAATTCTTCAGATGGTTTAGTGAATGGGGTGATGATTA
TTGCCAGGATAAAACAAAAATG
>Nigeria NIG185_EBA175AJ438820.1
TATGTATGTATTCCTGATCGTAGAATCCAATTATGCATTGTTAATCTTAGCATTATTAAAA
CATATACAAAAGAGACCATGAAGGATCATTTCATTGAAGCCTCTAAAAAAGAATCTCAA
CTTTTGCTTAAAAAAAAATGATAACAAATATAATTCTAAATTTTGTAATGATTTGAAGAATA
GTTTTTTAGATTATGGACATCTTGCTATGGGAAATGATATGGATTTTGGAGGTTATTCAAC
TAAGGCAGAAAACAAAATTCAAGAAGTTTTTAAAGGGGCTCATGGGGAAATAAGTGAAC
ATAAAATTAAAAATTTTAGAAAAGAATGGTGGAATGAATTTAGAGAGAAACTTTGGGAA
GCTATGTTATCTGAGCATAAAAATAATATAAATAATTGTAAAAATATTCCCCAAGAAGAA
TTACAAATTACTCAATGGATAAAAGAATGGCATGGAGAATTTTTGCTTGAAAGAGATAAT
AGATCAAAATTGCCAAAAAGTAAATGTAAAAATAATACATTATATGAAGCATGTGAGAA

GGAATGTATTGATCCATGTATGAAATATAGAGATTGGATTATTAGAAGTAAATTTGAATG
GCATACGTTATCGAAAGAATATGAAACTCAAAAAGTTCCAAAGGAAAATGCGGAAAATT
ATTTAATCAAAATTTCAGAAAACAAGAATGATGCTAAAGTAAGTTTATTATTGAATAATT
GTGATGCTGAATATTCAAAATATTGTGATTGTAAACATACTACTACTCTCGTTAAAAGCGT
TTTAAATGGTAACGACAATACAATTAAGGAAAAGCGTGAACATATTGATTTAGATGATTT
TTCTAAATTTGGATGTGATAAAAATTCCGTTGATACAAACACAAAGGTGTGGGAATGTAA
AAAACCTTATAAATTATCCACTAAAGATGTATGTGTACCTCCGAGGAGGCAAGAATTATG
TCTTGGAAACATTGATAGAATATACGATAAAAACCTATTAATGATAAAAGAGCATATTCT
TGCTATTGCAATATATGAATCAAGAATATTGAAACGAAAATATAAGAATAAAGATGATAA
AGAAGTTTGTAAAATCATAAATAAAACTTTCGCTGATATAAGAGATATTATAGGAGGTAC
TGATTATTGGAATGATTTGAGCAATAGAAAATTAGTAGGAAAAATTAACACAAATTCAAA
TTATGTTCACAGGAATAAACAAAATGATAAGCTTTTTCGTGATGAGTGGTGGAAAGTTAT
TAAAAAAGATGTATGGAATGTGATATCATGGGTATTCAAGGATAAAACTGTTTGTAAAGA
AGATGATATTGAAAATATACCACAATTCTTCAGATGGTTTAGTGAATGGGGTGATGATTA
TTGCCAGGATAAAACAAAAATG
>Nigeria NIG171_EBA175AJ438819.1
TATGTATGTATTCCTGATCGTAGAATCCAATTATGCATTGTTAATCTTAGCATTATTAAAA
CATATACAAAAGAGACCATGAAGGATCATTTCATTGAAGCCTCTAAAAAAGAATCTCAA
CTTTTGCTTAAAAAAAAATGATAACAAATATAATTCTAAATTTTGTAATGATTTGAAGAATA
GTTTTTTAGATTATGGACATCTTGCTATGGGAAATGATATGGATTTTGGAGGTTATTCAAC
TAAGGCAGAAAACAAAATTCAAGAAGTTTTTAAAGGGGCTCATGGGGAAATAAGTGAAC
ATGAAATTAAAAATTTTAGAAAAAAATGGTGGAATGAATTTAGAGAGAAACTTTGGGAA
GCTATGTTATCTGAGCATAAAAATAATATAAATAATTGTAAAAATATTCCCCAAGAAGAA
TTACAAATTACTCAATGGATAAAAGAATGGCATGGAGAATTTTTGCTTGAAAGAGATAAT
AGATCAAAATTGCCAAAAAGTAAATGTAAAAATAATACATTATATGAAGCATGTGAGAA
GGAATGTATTGATCCATGTATGAAATATAGAGATTGGATTATTAGAAGTAAATTTGAATG
GCATACGTTATCGAAAGAATATGAAACTCAAAAAGTTCCAAAGGAAAATGCGGAAAATT
ATTTAATCAAAATTTCAGAAAACAAGAATGATGCTAAAGTAAGTTTATTATTGAATAATT
GTGATGCTGAATATTCAAAATATTGTGATTGTAAACATACTACTACTCTCGTTAAAAGCGT
TTTAAATGGTAACGACAATACAATTAAGGAAAAGCGTGAACATATTGATTTAGATGATTT
TTCTAAATTTGGATGTGATAAAAATTCCGTTGATACAAACACAAAGGTGTGGGAATGTAA
AAAACCTTATATATTATCCACTAAAGATGTATGTGTACCTCCGAGGAGGCAAGAATTATG
TCTTGGAAACATTGATAGAATATACGATAAAAACCTATTAATGATAAAAGAGCATATTCT
TGCTATTGCAATATATGAATCAAGAATATTGAAACGAAAATATAAGAATAAAGATGATAA
AGAAGTTTGTAAAATCATAAATAAAACTTTCGCTGATATAAGAGATATTATAGGAGGTAC
TGATTATTGGAATGATTTGAGCAATAGAAAATTAGTAGGAAAAATTAACACAAATTCAAA
TTATGTTCACAGGAATAAAAAAAAATGATAAGCTTTTTCGTGATGAGTGGTGGAAAGTTAT
TAAAAAAGATGTATGGAATGTGATATCATGGGTATTCAAGGATAAAACTGTTTGTAAAGA
AGATGATATTGAAAATATACCACAATTCTTCAGATGGTTTAGTGAATGGGGTGATGATTA
TTGCCAGGATAAAACAAAAATG
>Nigeria NIG170_EBA175AJ438818.1
TATGTATGTATTCCTGATCGTAGAATCCAATTATGCATTGTTAATCTTAGCATTATTAAAA
CATATACAAAAGAGACCATGAAGGATCATTTCATTGAAGCCTCTAAAAAAGAATCTCAA
CTTTTGCTTAAAAAAAAATGATAACAAATATAATTCTAAATTTTGTAATGATTTGAAGAATA

GTTTTTTAGATTATGGACATCTTGCTATGGGAAATGATATGGATTTTGGAGGTTATTCAAC
TAAGGCAGAAAACAAAATTCAAGAAGTTTTTAAAGGGGCTCATGGGAAAATAAGTGAAC
ATGAAATTAAAAATTTTAGAAAAGAATGGTGGAATGAATTTAGAGAGAAACTTTGGGAA
GCTATGTTATCTGAGCATAAAAATAATATAAATAATTGTAAAAATATTCCCCAAGAAGAA
TTACAAATTACTCAATGGATAAAAGAATGGCATGGAGAATTTTTGCTTGAAAGAGATAAT
AGATCAAAATTGCCAAAAAGTAAATGTAAAAATAATACATTATATGAAGCATGTGAGAA
GGAATGTATTGATCCATGTATGAAATATAGAGATTGGATTATTAGAAGTAAATTTGAATG
GCATACGTTATCGAAAGAATATGAAACTCAAAATGTTTCAAAGGAAAATGCGGAAAATT
ATTTAATCAAAATTTCAGAAAAAATGAATGATGCTAAAGTAAGTTTATTATTGAATAATTG
TGATGCTGAATATTCAAAATATTGTGATTGTAAACATACTACTACTCTCGTTAAAAGCGTT
TTAAATGGTAACGACAATACAATTAAGGAAAAGCGTGAACATATTGATTTAGATGATTTT
TCTAAATTTGGATGTGATAAAAATTCCGTTGATACAAACACAAAGGTGTGGGAATGTAAA
AAACCTTATAAATTATCCACTAAAGATGTATGTGTACCTCCGAGGAGGCAAGAATTATGT
CTTGGAAACATTGATAGAATATACGATAAAAACCTATTAATGATAAAAGAGCATATTCTT
GCTATTGCAATATATGAATCAAGAATATTGAAACGAAAATATAAGAATAAAGATGATAA
AGAAGTTTGTAAAATCATAAATAAAACTTTCGCTGATATAAGAGATATTATAGGAGGTAC
TGATTATTGGAATGATTTGAGCAATAGAAAATTAGTAGGAAAAATTAACACAAATTCAAA
TTATGTTCACAGGAATAAACAAAATGATAAGCTTTTTCGTGATGAGTGGTGGAAAGTTAT
TAAAAAAGATGTATGGAATGTGATATCATGGGTATTCAAGGATAAAACTGTTTGTAAAGA
AGATGATATTGAAAATATACCACAATTCTTCAGATGGTTTAGTGAATGGGGTGATGATTA
TTGCCAGGATAAAACAAAAATG
>Nigeria NIG167_EBA175AJ438817.1
TATGTATGTATTCCTGATCGTAGAATCCAATTATGCATTGTTAATCTTAGCATTATTAAAA
CATATACAAAAGAGACCATGAAGGATCATTTCATTGAAGCCTCTAAAAAAGAATCTCAA
CTTTTGCTTAAAAAAAATGATAACAAATATAATTCTAAATTTTGTAATGATTTGAAGAATA
GTTTTTTAGATTATGGACATCTTGCTATGGGAAATGATATGGATTTTGGAGGTTATTCAAC
TAAGGCAGAAAACAAAATTCAAGAAGTTTTTAAAGGGGCTCATGGGGAAATAAGTGAAC
ATGAAATTAAAAATTTTAGAAAAAAATGGTGGAATGAATTTAGAGAGAAACTTTGGGAA
GCTATGTTATCTGAGCATAAAAATAATATAAATAATTGTAAAAATATTCCCCAAGAAGAA
TTACAAATTACTCAATGGATAAAAGAATGGCATGGAGAATTTTTGCTTGAAAGAGATAAT
AGATCAAAATTGCCAAAAAGTAAATGTAAAAATAATACATTATATGAAGCATGTGAGAA
GGAATGTATTGATCCATGTATGAAATATAGAGATTGGATTATTAGAAGTAAATTTGAATG
GCATACGTTATCGAAAGAATATGAAACTCAAAAAGTTTCAAAGGAAAATGCGGAAAATT
ATTTAATCAAAATTTCAGAAAACAAGAATGATGCTAAAGTAAGTTTATTATTGAATAATT
GTGATGCTGAATATTCAAAATATTGTGATTGTAAACATACTACTACTCTCGTTAAAAGCGT
TTTAAATGGTAACGACAATACAATTAAGGAAAAGCGTGAACATATTGATTTAGATGATTT
TTCTAAATTTGGATGTGATAAAAATTCCGTTGATACAAACACAAAGGTGTGGGAATGTAA
AAAACCTTATATATTATCCACTAAAGATGTATGTGTACCTCCGAGGAGGCAAGAATTATG
TCTTGGAAACATTGATAGAATATACGATAAAAACCTATTAATGATAAAAGAGCATATTCT
TGCTATTGCAATATATGAATCAAGAATATTGAAACGAAAATATAAGAATAAAGATGATAA
AGAAGTTTGTAAAATCATAAATAAAACTTTCGCTGATATAAGAGATATTATAGGAGGTAC
TGATTATTGGAATGATTTGAGCAATAGAAAATTAGTAGGAAAAATTAACACAAATTCAAA
ATATGTTCACAGGAATAAAAAAAAATGATAAGCTTTTTCGTGATGAGTGGTGGAAAGTTAT
TAAAAAAGATGTATGGAATGTGATATCATGGGTATTCAAGGATAAAACTGTTTGTAAAGA

AGATGATATTGAAAATATACCACAATTCTTCAGATGGTTTAGTGAATGGGGTGATGATTA
TTGCCAGGATAAAACAAAAATG
>Nigeria NIG163_EBA175AJ438816.1
TATGTATGTATTCCTGATCGTAGAATCCAATTATGCATTGTTAATCTTAGCATTATTAAAA
CATATACAAAAGAGACCATGAAGGATCATTTCATTGAAGCCTCTAAAAAAGAATCTCAA
CTTTTGCTTAAAAAAAATGATAACAAATATAATTCTAAATTTTGTAATGATTTGAAGAATA
GTTTTTTAGATTATGGACATCTTGCTATGGGAAATGATATGGATTTTGGAGGTTATTCAAC
TAAGGCAGAAAACAAAATTCAAGAAGTTTTTAAAGGGGCTCATGGGGAAATAAGTGAAC
ATGAAATTAAAAATTTTAGAAAAAAATGGTGGAATGAATTTAGAGAGAAACTTTGGGAA
GCTATGTTATCTGAGCATAAAAATAATATAAATAATTGTAAAAATATTCCCCAAGAAGAA
TTACAAATTACTCAATGGATAAAAGAATGGCATGGAGAATTTTTGCTTGAAAGAGATAAT
AGATCAAAATTGCCAAAAAGTAAATGTAAAAATAATACATTATATGAAGCATGTGAGAA
GGAATGTATTGATCCATGTATGAAATATAGAGATTGGATTATTAGAAGTAAATTTGAATG
GCATACGTTATCGAAAGAATATGAAACTCAAAAAGTTTCAAAGGAAAATGCGGAAAATT
ATTTAATCAAAATTTCAGAAAACAAGAATGATGCTAAAGTAAGTTTATTATTGAATAATT
GTGATGCTGAATATTCAAAATATTGTGATTGTAAACATACTACTACTCTCGTTAAAAGCGT
TTTAAATGGTAACGACAATACAATTAAGGAAAAGCGTGAACATATTGATTTAGATGATTT
TTCTAAATTTGGATGTGATAAAAATTCCGTTGATACAAACACAAAGGTGTGGGAATGTAA
AAAACCTTATATATTATCCACTAAAGATGTATGTGTACCTCCGAGGAGGCAAGAATTATG
TCTTGGAAACATTGATAGAATATACGATAAAAACCTATTAATGATAAAAGAGCATATTCT
TGCTATTGCAATATATGAATCAAGAATATTGAAACGAAAATATAAGAATAAAGATGATAA
AGAAGTTTGTAAAATCATAAATAAAACTTTCGCTGATATAAGAGATATTATAGGAGGTAC
TGATTATTGGAATGATTTGAGCAATAGAAAATTAGTAGGAAAAATTAACACAAATTCAAA
ATATGTTCACAGGAATAAAAAAAATGATAAGCTTTTTCGTGATGAGTGGTGGAAAGTTAT
TAAAAAAGATGTATGGAATGTGATATCATGGGTATTCAAGGATAAAACTGTTTGTAAAGA
AGATGATATTGAAAATATACCACAATTCTTCAGATGGTTTAGTGAATGGGGTGATGATTA
TTGCCAGGATAAAACAAAAATG
>Nigeria NIG162_EBA175AJ438815.1
TATGTATGTATTCCTGATCGTAGAATCCAATTATGCATTGTTAATCTTAGCATTATTAAAA
CATATACAAAAGAGACCATGAAGGATCATTTCATTGAAGCCTCTAAAAAAGAATCTCAA
CTTTTGCTTAAAAAAAATGATAACAAATATAATTCTAAATTTTGTAATGATTTGAAGAATA
GTTTTTTAGATTATGGACATCTTGCTATGGGAAATGATATGGATTTTGGAGGTTATTCAAC
TAAGGCAGAAAACAAAATTCAAGAAGTTTTTAAAGGGGCTCATGGGGAAATAAGTGAAC
ATGAAATTAAAAATTTTAGAAAAAAATGGTGGAATGAATTTAGAGAGAAACTTTGGGAA
GCTATGTTATCTGAGCATAAAAATAATATAAATAATTGTAAAAATATTCCCCAAGAAGAA
TTACAAATTACTCAATGGATAAAAGAATGGCATGGAGAATTTTTGCTTGAAAGAGATAAT
AGATCAAAATTGCCAAAAAGTAAATGTAAAAATAATACATTATATGAAGCATGTGAGAA
GGAATGTATTGATCCATGTATGAAATATAGAGATTGGATTATTAGAAGTAAATTTGAATG
GCATACGTTATCGAAAGAATATGAAACTCAAAAAGTTCCAAAGGAAAATGCGGAAAATT
ATTTAATCAAAATTTCAGAAAACAAGAATGATGCTAAAGTAAGTTTATTATTGAATAATT
GTGATGCTGAATATTCAAAATATTGTGATTGTAAACATACTACTACTCTCGTTAAAAGCGT
TTTAAATGGTAACGACAATACAATTAAGGAAAAGCGTGAACATATTGATTTAGATGATTT
TTCTAAATTTGGATGTGATAAAAATTCCGTTGATACAAACACAAAGGTGTGGGAATGTAA
AAAACCTTATAAATTATCCACTAAAGATGTATGTGTACCTCCGAGGAGGCAAGAATTATG

TCTTGGAAACATTGATAGAATATACGATAAAAACCTATTAATGATAAAAGAGCATATTCT
TGCTATTGCAATATATGAATCAAGAATATTGAAACGAAAATATAAGAATAAAGATGATAA
AGAAGTTTGTAAAATCATAAATAAAACTTTCGCTGATATAAGAGATATTATAGGAGGTAC
TGATTATTGGAATGATTTGAGCAATAGAAAATTAGTAGGAAAAATTAACACAAATTCAAA
TTATGTTCACAGGAATAAACAAAATGATAAGCTTTTTCGTGATGAGTGGTGGAAAGTTAT
TAAAAAAGATGTATGGAATGTGATATCATGGGTATTCAAGGATAAAACTGTTTGTAAAGA
AGATGATATTGAAAATATACCACAATTCTTCAGATGGTTTAGTGAATGGGGTGATGATTA
TTGCCAGGATAAAACAAAAATG
>Nigeria NIG160_EBA175AJ438814.1
TATGTATGTATTCCTGATCGTAGAATCCAATTATGCATTGTTAATCTTAGCATTATTAAAA
CATATACAAAAGAGACCATGAAGGATCATTTCATTGAAGCCTCTAAAAAAGAATCTCAA
CTTTTGCTTAAAAAAAAATGATAACAAATATAATTCTAAATTTTGTAATGATTTGAAGAATA
GTTTTTTAGATTATGGACATCTTGCTATGGGAAATGATATGGATTTTGGAGGTTATTCAAC
TAAGGCAGAAAACAAAATTCAAGAAGTTTTTAAAGGGGCTCATGGGGAAATAAGTGAAC
ATAAAATTAAAAATTTTAGAAAAGAATGGTGGAATGAATTTAGAGAGAAACTTTGGGAA
GCTATGTTATCTGAGCATAAAAATAATATAAATAATTGTAAAAATATTCCCCAAGAAGAA
TTACAAATTACTCAATGGATAAAAGAATGGCATGGAGAATTTTTGCTTGAAAGAGATAAT
AGATCAAAATTGCCAAAAAGTAAATGTAAAAATAATACATTATATGAAGCATGTGAGAA
GGAATGTATTGATCCATGTATGAAATATAGAGATTGGATTATTAGAAGTAAATTTGAATG
GCATACGTTATCGAAAGAATATGAAACTCAAAAAGTTCCAAAGGAAAATGCGGAAAATT
ATTTAATCAAAATTTCAGAAAACAAGAATGATGCTAAAGTAAGTTTATTATTGAATAATT
GTGATGCTGAATATTCAAAATATTGTGATTGTAAACATACTACTACTCTCGTTAAAAGCGT
TTTAAATGGTAACGACAATACAATTAAGGAAAAGCGTGAACATATTGATTTAGATGATTT
TTCTAAATTTGGATGTGATAAAAATTCCGTTGATACAAACACAAAGGTGTGGGAATGTAA
AAAACCTTATAAATTATCCACTAAAGATGTATGTGTACCTCCGAGGAGGCAAGAATTATG
TCTTGGAAACATTGATAGAATATACGATAAAAACCTATTAATGATAAAAGAGCATATTCT
TGCTATTGCAATATATGAATCAAGAATATTGAAACGAAAATATAAGAATAAAGATGATAA
AGAAGTTTGTAAAATCATAAATAAAACTTTCGCTGATATAAGAGATATTATAGGAGGTAC
TGATTATTGGAATGATTTGAGCAATAGAAAATTAGTAGGAAAAATTAACACAAATTCAAA
TTATGTTCACAGGAATAAACAAAATGATAAGCTTTTTCGTGATGAGTGGTGGAAAGTTAT
TAAAAAAGATGTATGGAATGTGATATCATGGGTATTCAAGGATAAAACTGTTTGTAAAGA
AGATGATATTGAAAATATACCACAATTCTTCAGATGGTTTAGTGAATGGGGTGATGATTA
TTGCCAGGATAAAACAAAAATG
>Nigeria NIG157_EBA175AJ438813.1
TATGTATGTATTCCTGATCGTAGAATCCAATTATGCATTGTTAATCTTAGCATTATTAAAA
CATATACAAAAGAGACCATGAAGGATCATTTCATTGAAGCCTCTAAAAAAGAATCTCAA
CTTTTGCTTAAAAAAAAATGATAACAAATATAATTCTAAATTTTGTAATGATTTGAAGAATA
GTTTTTTAGATTATGGACATCTTGCTATGGGAAATGATATGGATTTTGGAGGTTATTCAAC
TAAGGCAGAAAACAAAATTCAAGAAGTTTTTAAAGGGGCTCATGGGGAAATAAGTGAAC
ATGAAATTAAAAATTTTAGAAAAAAAATGGTGGAATGAATTTAGAGAGAAACTTTGGGAA
GCTATGTTATCTGAGCATAAAAATAATATAAATAATTGTAAAAATATTCCCCAAGAAGAA
TTACAAATTACTCAATGGATAAAAGAATGGCATGGAGAATTTTTGCTTGAAAGAGATAAT
AGATCAAAATTGCCAAAAAGTAAATGTAAAAATAATACATTATATGAAGCATGTGAGAA
GGAATGTATTGATCCATGTATGAAATATAGAGATTGGATTATTAGAAGTAAATTTGAATG

GCATACGTTATCGAAAGAATATGAAACTCAAAAAGTTTCAAAGGAAAATGCGGAAAATT
ATTTAATCAAAATTTCAGAAAACAAGAATGATGCTAAAGTAAGTTTATTATTGAATAATT
GTGATGCTGAATATTCAAAATATTGTGATTGTAAACATACTACTACTCTCGTTAAAAGCGT
TTTAAATGGTAACGACAATACAATTAAGGAAAAGCGTGAACATATTGATTTAGATGATTT
TTCTAAATTTGGATGTGATAAAAATTCCGTTGATACAAACACAAAGGTGTGGGAATGTAA
AAAACCTTATATATTATCCACTAAAGATGTATGTGTACCTCCGAGGAGGCAAGAATTATG
TCTTGGAAACATTGATAGAATATACGATAAAAACCTATTAATGATAAAAGAGCATATTCT
TGCTATTGCAATATATGAATCAAGAATATTGAAACGAAAATATAAGAATAAAGATGATAA
AGAAGTTTGTAAAATCATAAATAAAACTTTCGCTGATATAAGAGATATTATAGGAGGTAC
TGATTATTGGAATGATTTGAGCAATAGAAAATTAGTAGGAAAAATTAACACAAATTCAAA
ATATGTTCACAGGAATAAAAAAAATGATAAGCTTTTTCGTGATGAGTGGTGGAAAGTTAT
TAAAAAAGATGTATGGAATGTGATATCATGGGTATTCAAGGATAAAACTGTTTGTAAAGA
AGATGATATTGAAAATATACCACAATTCTTCAGATGGTTTAGTGAATGGGGTGATGATTA
TTGCCAGGATAAAACAAAAATG
>Nigeria NIG151_EBA175AJ438812.1
TATGTATGTATTCCTGATCGTAGAATCCAATTATGCATTGTTAATCTTAGCATTATTAAAA
CATATACAAAAGAGACCATGAAGGATCATTTCATTGAAGCCTCTAAAAAAGAATCTCAA
CTTTTGCTTAAAAAAAATGATAACAAATATAATTCTAAATTTTGTAATGATTTGAAGAATA
GTTTTTTAGATTATGGACATCTTGCTATGGGAAATGATATGGATTTTGGAGGTTATTCAAC
TAAGGCAGAAAACAAAATTCAAGAAGTTTTTAAAGGGGCTCATGGGAAAATAAGTGAAC
ATGAAATTAAAAATTTTAGAAAAAAATGGTGGAATGAATTTAGAGAGAAACTTTGGGAA
GCTATGTTATCTGAGCATAAAAATAATATAAATAATTGTAAAAATATTCCCCAAGAAGAA
TTACAAATTACTCAATGGATAAAAGAATGGCATGGAGAATTTTTGCTTGAAAGAGATAAT
AGATCAAAATTGCCAAAAAGTAAATGTAAAAATAATACATTATATGAAGCATGTGAGAA
GGAATGTATTGATCCATGTATGAAATATAGAGATTGGATTATTAGAAGTAAATTTGAATG
GCATACGTTATCGAAAGAATATGAAACTCAAAATGTTTCAAAGGAAAATGCGGAAAATT
ATTTAATCAAAATTTCAAAAAACAAGAATGATGCTAAAGTAAGTTTATTATTGAATAATTG
TGATGCTGAATATTCAAAATATTGTGATTGTAAACATACTACTACTCTCGTTAAAAGCGTT
TTAAATGGTAACGACAATACAATTAAGGAAAAGCGTGAACATATTGATTTAGATGATTTT
TCTAAATTTGGATGTGATAAAAATTCCGTTGATACAAACACAAAGGTGTGGGAATGTAAA
AAACCTTATAAATTATCCACTAAAGATGTATGTGTACCTCCGAGGAGGCAAGAATTATGT
CTTGGAAACATTGATAGAATATACGATAAAAACCTATTAATGATAAAAGAGCATATTCTT
GCTATTGCAATATATGAATCAAGAATATTGAAACGAAAATATAAGAATAAAGATGATAA
AGAAGTTTGTAAAATCATAAATAAAACTTTCGCTGATATAAGAGATATTATAGGAGGTAC
TGATTATTGGAATGATTTGAGCAATAGAAAATTAGTAGGAAAAATTAACACAAATTCAAA
TTATGTTCACAGGAATAAACAAAATGATAAGCTTTTTCGTGATGAGTGGTGGAAAGTTAT
TAAAAAAGATGTATGGAATGTGATATCATGGGTATTCAAGGATAAAACTGTTTGTAAAGA
AGATGATATTGAAAATATACCACAATTCTTCAGATGGTTTAGTGAATGGGGTGATGATTA
TTGCCAGGATAAAACAAAAATG
>Nigeria NIG149_EBA175AJ438811.1
TATGTATGTATTCCTGATCGTAGAATCCAATTATGCATTGTTAATCTTAGCATTATTAAAA
CATATACAAAAGAGACCATGAAGGATCATTTCATTGAAGCCTCTAAAAAAGAATCTCAA
CTTTTGCTTAAAAAAAATGATAACAAATATAATTCTAAATTTTGTAATGATTTGAAGAATA
GTTTTTTAGATTATGGACATCTTGCTATGGGAAATGATATGGATTTTGGAGGTTATTCAAC

TAAGGCAGAAAACAAAATTCAAGAAGTTTTTAAAGGGGCTCATGGGGAAATAAGTGAAC
ATGAAATTAAAAATTTTAGAAAAAAAATGGTGGAATGAATTTAGAGAGAAACTTTGGGAA
GCTATGTTATCTGAGCATAAAAATAATATAAATAATTGTAAAAATATTCCCCAAGAAGAA
TTACAAATTACTCAATGGATAAAAGAATGGCATGGAGAATTTTTGCTTGAAAGAGATAAT
AGATCAAAATTGCCAAAAAGTAAATGTAAAAATAATACATTATATGAAGCATGTGAGAA
GGAATGTATTGATCCATGTATGAAATATAGAGATTGGATTATTAGAAGTAAATTTGAATG
GCATACGTTATCGAAAGAATATGAAACTCAAAATGTTTCAAAGGAAAATGCGGAAAATT
ATTTAATCAAAATTTCAAAAAACAAGAATGATGCTAAAGTAAGTTTATTATTGAATAATTG
TGATGCTGAATATTCAAAATATTGTGATTGTAAACATACTACTACTCTCGTTAAAAGCGTT
TTAAATGGTAACGACAATACAATTAAGGAAAAGCGTGAACATATTGATTTAGATGATTTT
TCTAAATTTGGATGTGATAAAAATTCCGTTGATACAAACACAAAGGTGTGGGAATGTAAA
AAACCTTATAAATTATCCACTAAAGATGTATGTGTACCTCCGAGGAGGCAAGAATTATGT
CTTGGAAACATTGATAGAATATACGATAAAAACCTATTAATGATAAAAGAGCATATTCTT
GCTATTGCAATATATGAATCAAGAATATTGAAACGAAAATATAAGAATAAAGATGATAA
AGAAGTTTGTAAAATCATAAATAAAACTTTCGCTGATATAAGAGATATTATAGGAGGTAC
TGATTATTGGAATGATTTGAGCAATAGAAAATTAGTAGGAAAAATTAACACAAATTCAAA
TTATGTTCACAGGAATAAACAAAATGATAAGCTTTTTCGTGATGAGTGGTGGAAAGTTAT
TAAAAAAGATGTATGGAATGTGATATCATGGGTATTCAAGGATAAAACTGTTTGTAAAGA
AGATGATATTGAAAATATACCACAATTCTTCAGATGGTTTAGTGAATGGGGTGATGATTA
TTGCCAGGATAAAACAAAAATG
>Nigeria NIG146_EBA175AJ438810.1
TATGTATGTATTCCTGATCGTAGAATCCAATTATGCATTGTTAATCTTAGCATTATTAAAA
CATATACAAAAGAGACCATGAAGGATCATTTCATTGAAGCCTCTAAAAAAGAATCTCAA
CTTTTGCTTAAAAAAAAATGATAACAAATATAATTCTAAATTTTGTAATGATTTGAAGAATA
GTTTTTTAGATTATGGACATCTTGCTATGGGAAATGATATGGATTTTGGAGGTTATTCAAC
TAAGGCAGAAAACAAAATTCAAGAAGTTTTTAAAGGGGCTCATGGGAAAATAAGTGAAC
ATGAAATTAAAAATTTTAGAAAAGAATGGTGGAATGAATTTAGAGAGAAACTTTGGGAA
GCTATGTTATCTGAGCATAAAAATAATATAAATAATTGTAAAAATATTCCCCAAGAAGAA
TTACAAATTACTCAATGGATAAAAGAATGGCATGGAGAATTTTTGCTTGAAAGAGATAAT
AGATCAAAATTGCCAAAAAGTAAATGTAAAAATAATACATTATATGAAGCATGTGAGAA
GGAATGTATTGATCCATGTATGAAATATAGAGATTGGATTATTAGAAGTAAATTTGAATG
GCATACGTTATCGAAAGAATATGAAACTCAAAATGTTTCAAAGGAAAATGCGGAAAATT
ATTTAATCAAAATTTCAGAAAACATGAATGATGCTAAAGTAAGTTTATTATTGAATAATTG
TGATGCTGAATATTCAAAATATTGTGATTGTAAACATACTACTACTCTCGTTAAAAGCGTT
TTAAATGGTAACGACAATACAATTAAGGAAAAGCGTGAACATATTGATTTAGATGATTTT
TCTAAATTTGGATGTGATAAAAATTCCGTTGATACAAACACAAAGGTGTGGGAATGTAAA
AAACCTTATAAATTATCCACTAAAGATGTATGTGTACCTCCGAGGAGGCAAGAATTATGT
CTTGGAAACATTGATAGAATATACGATAAAAACCTATTAATGATAAAAGAGCATATTCTT
GCTATTGCAATATATGAATCAAGAATATTGAAACGAAAATATAAGAATAAAGATGATAA
AGAAGTTTGTAAAATCATAAATAAAACTTTCGCTGATATAAGAGATATTATAGGAGGTAC
TGATTATTGGAATGATTTGAGCAATAGAAAATTAGTAGGAAAAATTAACACAAATTCAAA
TTATGTTCACAGGAATAAACAAAATGATAAGCTTTTTCGTGATGCGTGGTGGAAAGTTAT
TAAAAAAGATGTATGGAATGTGATATCATGGGTATTCAAGGATAAAACTGTTTGTAAAGA
AGATGATATTGAAAATATACCACAATTCTTCAGATGGTTTAGTGAATGGGGTGATGATTA

TTGCCAGGATAAAACAAAAATG
>Nigeria NIG140_EBA175AJ438809.1
TATGTATGTATTCCTGATCGTAGAATCCAATTATGCATTGTTAATCTTAGCATTATTAAAA
CATATACAAAAGAGACCATGAAGGATCATTTCATTGAAGCCTCTAAAAAAGAATCTCAA
CTTTTGCTTAAAAAAAAATGATAACAAATATAATTCTAAATTTTGTAATGATTTGAAGAATA
GTTTTTTAGATTATGGACATCTTGCTATGGGAAATGATATGGATTTTGGAGGTTATTCAAC
TAAGGCAGAAAACAAAATTCAAGAAGTTTTTAAAGGGGCTCATGGGGAAATAAGTGAAC
ATGAAATTAAAAATTTTAGAAAAAAAATGGTGGAATGAATTTAGAGAGAAACTTTGGGAA
GCTATGTTATCTGAGCATAAAAATAATATAAATAATTGTAAAAATATTCCCCAAGAAGAA
TTACAAATTACTCAATGGATAAAAGAATGGCATGGAGAATTTTTGCTTGAAAGAGATAAT
AGATCAAAATTGCCAAAAAGTAAATGTAAAAATAATACATTATATGAAGCATGTGAGAA
GGAATGTATTGATCCATGTATGAAATATAGAGATTGGATTATTAGAAGTAAATTTGAATG
GCATACGTTATCGAAAGAATATGAAACTCAAAAAGTTTCAAAGGAAAATGCGGAAAATT
ATTTAATCAAAATTTCAGAAAACAAGAATGATGCTAAAGTAAGTTTATTATTGAATAATT
GTGATGCTGAATATTCAAAATATTGTGATTGTAAACATACTACTACTCTCGTTAAAAGCGT
TTTAAATGGTAACGACAATACAATTAAGGAAAAGCGTGAACATATTGATTTAGATGATTT
TTCTAAATTTGGATGTGATAAAAATTCCGTTGATACAAACACAAAGGTGTGGGAATGTAA
AAAACCTTATATATTATCCACTAAAGATGTATGTGTACCTCCGAGGAGGCAAGAATTATG
TCTTGGAAACATTGATAGAATATACGATAAAAACCTATTAATGATAAAAGAGCATATTCT
TGCTATTGCAATATATGAATCAAGAATATTGAAACGAAAATATAAGAATAAAGATGATAA
AGAAGTTTGTAAAATCATAAATAAAACTTTCGCTGATATAAGAGATATTATAGGAGGTAC
TGATTATTGGAATGATTTGAGCAATAGAAAATTAGTAGGAAAAATTAACACAAATTCAAA
ATATGTTCACAGGAATAAAAAAAAATGATAAGCTTTTTCGTGATGAGTGGTGGAAAGTTAT
TAAAAAAGATGTATGGAATGTGATATCATGGGTATTCAAGGATAAAACTGTTTGTAAAGA
AGATGATATTGAAAATATACCACAATTCTTCAGATGGTTTAGTGAATGGGGTGATGATTA
TTGCCAGGATAAAACAAAAATG
>Nigeria NIG132_EBA175AJ438808.1
TATGTATGTATTCCTGATCGTAGAATCCAATTATGCATTGTTAATCTTAGCATTATTAAAA
CATATACAAAAGAGACCATGAAGGATCATTTCATTGAAGCCTCTAAAAAAGAATCTCAA
CTTTTGCTTAAAAAAAAATGATAACAAATATAATTCTAAATTTTGTAATGATTTGAAGAATA
GTTTTTTAGATTATGGACATCTTGCTATGGGAAATGATATGGATTTTGGAGGTTATTCAAC
TAAGGCAGAAAACAAAATTCAAGAAGTTTTTAAAGGGGCTCATGGGGAAATAAGTGAAC
ATAAAATTAAAAATTTTAGAAAAGAATGGTGGAATGAATTTAGAGAGAAACTTTGGGAA
GCTATGTTATCTGAGCATAAAAATAATATAAATAATTGTAAAAATATTCCCCAAGAAGAA
TTACAAATTACTCAATGGATAAAAGAATGGCATGGAGAATTTTTGCTTGAAAGAGATAAT
AGATCAAAATTGCCAAAAAGTAAATGTAAAAATAATACATTATATGAAGCATGTGAGAA
GGAATGTATTGATCCATGTATGAAATATAGAGATTGGATTATTAGAAGTAAATTTGAATG
GCATACGTTATCGAAAGAATATGAAACTCAAAAAGTTTCAAAGGAAAATGCGGAAAATT
ATTTAATCAAAATTTCAGAAAACAAGAATGATGCTAAAGTAAGTTTATTATTGAATAATT
GTGATGCTGAATATTCAAAATATTGTGATTGTAAACATACTACTACTCTCGTTAAAAGCGT
TTTAAATGGTAACGACAATACAATTAAGGAAAAGCGTGAACATATTGATTTAGATGATTT
TTCTAAATTTGGATGTGATAAAAATTCCGTTGATACAAACACAAAGGTGTGGGAATGTAA
AAAACCTTATAAATTATCCACTAAAGATGTATGTGTACCTCCGAGGAGGCAAGAATTATG
TCTTGGAAACATTGATAGAATATACGATAAAAACCTATTAATGATAAAAGAGCATATTCT

TGCTATTGCAATATATGAATCAAGAATATTGAAACGAAAATATAAGAATAAAGATGATAA
AGAAGTTTGTAAAATCATAAATAAAACTTTCGCTGATATAAGAGATATTATAGGAGGTAC
TGATTATTGGAATGATTTGAGCAATAGAAAATTAGTAGGAAAAATTAACACAAATTCAAA
TTATGTTCACAGGAATAAACAAAATGATAAGCTTTTTCGTGATGAGTGGTGGAAAGTTAT
TAAAAAAGATGTATGGAATGTGATATCATGGGTATTCAAGGATAAAACTGTTTGTAAAGA
AGATGATATTGAAAATATACCACAATTCTTCAGATGGTTTAGTGAATGGGGTGATGATTA
TTGCCAGGATAAAACAAAAATG
>Nigeria NIG113_EBA175AJ438807.1
TATGTATGTATTCCTGATCGTAGAATCCAATTATGCATTGTTAATCTTAGCATTATTAAAA
CATATACAAAAGAGACCATGAAGGATCATTTCATTGAAGCCTCTAAAAAAGAATCTCAA
CTTTTGCTTAAAAAAAAATGATAACAAATATAATTCTAAATTTTGTAATGATTTGAAGAATA
GTTTTTTAGATTATGGACATCTTGCTATGGGAAATGATATGGATTTTGGAGGTTATTCAAC
TAAGGCAGAAAACAAAATTCAAGAAGTTTTTAAAGGGGCTCATGGGGAAATAAGTGAAC
ATGAAATTAAAAATTTTAGAAAAAAATGGTGGAATGAATTTAGAGAGAAACTTTGGGAA
GCTATGTTATCTGAGCATAAAAATAATATAAATAATTGTAAAAATATTCCCCAAGAAGAA
TTACAAATTACTCAATGGATAAAAGAATGGCATGGAGAATTTTTGCTTGAAAGAGATAAT
AGATCAAAATTGCCAAAAAGTAAATGTAAAAATAATACATTATATGAAGCATGTGAGAA
GGAATGTATTGATCCATGTATGAAATATAGAGATTGGATTATTAGAAGTAAATTTGAATG
GCATACGTTATCGAAAGAATATGAAACTCAAAAAGTTTCAAAGGAAAATGCGGAAAATT
ATTTAATCAAAATTTCAGAAAACAAGAATGATGCTAAAGTAAGTTTATTATTGAATAATT
GTGATGCTGAATATTCAAAATATTGTGATTGTAAACATACTACTACTCTCGTTAAAAGCGT
TTTAAATGGTAACGACAATACAATTAAGGAAAAGCGTGAACATATTGATTTAGATGATTT
TTCTAAATTTGGATGTGATAAAAATTCCGTTGATACAAACACAAAGGTGTGGGAATGTAA
AAAACCTTATATATTATCCACTAAAGATGTATGTGTACCTCCGAGGAGGCAAGAATTATG
TCTTGGAAACATTGATAGAATATACGATAAAAACCTATTAATGATAAAAGAGCATATTCT
TGCTATTGCAATATATGAATCAAGAATATTGAAACGAAAATATAAGAATAAAGATGATAA
AGAAGTTTGTAAAATCATAAATAAAACTTTCGCTGATATAAGAGATATTATAGGAGGTAC
TGATTATTGGAATGATTTGAGCAATAGAAAATTAGTAGGAAAAATTAACACAAATTCAAA
ATATGTTCACAGGAATAAAAAAAATGATAAGCTTTTTCGTGATGAGTGGTGGAAAGTTAT
TAAAAAAGATGTATGGAATGTGATATCATGGGTATTCAAGGATAAAACTGTTTGTAAAGA
AGATGATATTGAAAATATACCACAATTCTTCAGATGGTTTAGTGAATGGGGTGATGATTA
TTGCCAGGATAAAACAAAAATG
>Nigeria NIG112_EBA175AJ438806.1
TATGTATGTATTCCTGATCGTAGAATCCAATTATGCATTGTTAATCTTAGCATTATTAAAA
CATATACAAAAGAGACCATGAAGGATCATTTCATTGAAGCCTCTAAAAAAGAATCTCAA
CTTTTGCTTAAAAAAAAATGATAACAAATATAATTCTAAATTTTGTAATGATTTGAAGAATA
GTTTTTTAGATTATGGACATCTTGCTATGGGAAATGATATGGATTTTGGAGGTTATTCAAC
TAAGGCAGAAAACAAAATTCAAGAAGTTTTTAAAGGGGCTCATGGGAAAATAAGTGAAC
ATGAAATTAAAAATTTTAGAAAAAAATGGTGGAATGAATTTAGAGAGAAACTTTGGGAA
GCTATGTTATCTGAGCATAAAAATAATATAAATAATTGTAAAAATATTCCCCAAGAAGAA
TTACAAATTACTCAATGGATAAAAGAATGGCATGGAGAATTTTTGCTTGAAAGAGATAAT
AGATCAAAATTGCCAAAAAGTAAATGTAAAAATAATACATTATATGAAGCATGTGAGAA
GGAATGTATTGATCCATGTATGAAATATAGAGATTGGATTATTAGAAGTAAATTTGAATG
GCATACGTTATCGAAAGAATATGAAACTCAAAAAGTTCCAAAGGAAAATGCGGAAAATT

ATTTAATCAAAATTTCAAAAAACAAGAATGATGCTAAAGTAAGTTTATTATTGAATAATTG
TGATGCTGAATATTCAAAATATTGTGATTGTAAACATACTACTACTCTCGTTAAAAGCGTT
TTAAATGGTAACGACAATACAATTAAGGAAAAGCGTGAACATATTGATTTAGATGATTTT
TCTAAATTTGGATGTGATAAAAATTCCGTTGATACAAACACAAAGGTGTGGGAATGTAAA
AAACCTTATAAATTATCCACTAAAGATGTATGTGTACCTCCGAGGAGGCAAGAATTATGT
CTTGGAAACATTGATAGAATATACGATAAAAACCTATTAATGATAAAAGAGCATATTCTT
GCTATTGCAATATATGAATCAAGAATATTGAAACGAAAATATAAGAATAAAGATGATAA
AGAAGTTTGTAAAATCATAAATAAAACTTTCGCTGATATAAGAGATATTATAGGAGGTAC
TGATTATTGGAATGATTTGAGCAATAGAAAATTAGTAGGAAAAATTAACACAAATTCAAA
TTATGTTCACAGGAATAAACAAAATGATAAGCTTTTTCGTGATGAGTGGTGGAAAGTTAT
TAAAAAAGATGTATGGAATGTGATATCATGGGTATTCAAGGATAAAACTGTTTGTAAAGA
AGATGATATTGAAAATATACCACAATTCTTCAGATGGTTTAGTGAATGGGGTGATGATTA
TTGCCAGGATAAAACAAAAATG
>Nigeria NIG109_EBA175AJ438805.1
TATGTATGTATTCCTGATCGTAGAATCCAATTATGCATTGTTAATCTTAGCATTATTAAAA
CATATACAAAAGAGACCATGAAGGATCATTTCATTGAAGCCTCTAAAAAAGAATCTCAA
CTTTTGCTTAAAAAAAAATGATAACAAATATAATTCTAAATTTTGTAATGATTTGAAGAATA
GTTTTTTAGATTATGGACATCTTGCTATGGGAAATGATATGGATTTTGGAGGTTATTCAAC
TAAGGCAGAAAACAAAATTCAAGAAGTTTTTAAAGGGGCTCATGGGGAAATAAGTGAAC
ATGAAATTAAAAATTTTAGAAAAAAATGGTGGAATGAATTTAGAGAGAAACTTTGGGAA
GCTATGTTATCTGAGCATAAAAATAATATAAATAATTGTAAAAATATTCCCCAAGAAGAA
TTACAAATTACTCAATGGATAAAAGAATGGCATGGAGAATTTTTGCTTGAAAGAGATAAT
AGATCAAAATTGCCAAAAAGTAAATGTAAAAATAATACATTATATGAAGCATGTGAGAA
GGAATGTATTGATCCATGTATGAAATATAGAGATTGGATTATTAGAAGTAAATTTGAATG
GCATACGTTATCGAAAGAATATGAAACTCAAAAAGTTTCAAAGGAAAATGCGGAAAATT
ATTTAATCAAAATTTCAGAAAACAAGAATGATGCTAAAGTAAGTTTATTATTGAATAATT
GTGATGCTGAATATTCAAAATATTGTGATTGTAAACATACTACTACTCTCGTTAAAAGCGT
TTTAAATGGTAACGACAATACAATTAAGGAAAAGCGTGAACATATTGATTTAGATGATTT
TTCTAAATTTGGATGTGATAAAAATTCCGTTGATACAAACACAAAGGTGTGGGAATGTAA
AAAACCTTATATATTATCCACTAAAGATGTATGTGTACCTCCGAGGAGGCAAGAATTATG
TCTTGGAAACATTGATAGAATATACGATAAAAACCTATTAATGATAAAAGAGCATATTCT
TGCTATTGCAATATATGAATCAAGAATATTGAAACGAAAATATAAGAATAAAGATGATAA
AGAAGTTTGTAAAATCATAAATAAAACTTTCGCTGATATAAGAGATATTATAGGAGGTAC
TGATTATTGGAATGATTTGAGCAATAGAAAATTAGTAGGAAAAATTAACACAAATTCAAA
ATATGTTCACAGGAATAAAAAAAATGATAAGCTTTTTCGTGATGAGTGGTGGAAAGTTAT
TAAAAAAGATGTATGGAATGTGATATCATGGGTATTCAAGGATAAAACTGTTTGTAAAGA
AGATGATATTGAAAATATACCACAATTCTTCAGATGGTTTAGTGAATGGGGTGATGATTA
TTGCCAGGATAAAACAAAAATG
>Nigeria NIG105_EBA175AJ438804.1
TATGTATGTATTCCTGATCGTAGAATCCAATTATGCATTGTTAATCTTAGCATTATTAAAA
CATATACAAAAGAGACCATGAAGGATCATTTCATTGAAGCCTCTAAAAAAGAATCTCAA
CTTTTGCTTAAAAAAAAATGATAACAAATATAATTCTAAATTTTGTAATGATTTGAAGAATA
GTTTTTTAGATTATGGACATCTTGCTATGGGAAATGATATGGATTTTGGAGGTTATTCAAC
TAAGGCAGAAAACAAAATTCAAGAAGTTTTTAAAGGGGCTCATGGGAAAATAAGTGAAC

ATGAAATTAAAAATTTTAGAAAAGAATGGTGGAATGAATTTAGAGAGAAACTTTGGGAA
GCTATGTTATCTGAGCATAAAAATAATATAAATAATTGTAAAAATATTCCCCAAGAAGAA
TTACAAATTACTCAATGGATAAAAGAATGGCATGGAGAATTTTTGCTTGAAAGAGATAAT
AGATCAAAATTGCCAAAAAGTAAATGTAAAAATAATACATTATATGAAGCATGTGAGAA
GGAATGTATTGATCCATGTATGAAATATAGAGATTGGATTATTAGAAGTAAATTTGAATG
GCATACGTTATCGAAAGAATATGAAACTCAAAATGTTTCAAAGGAAAATGCGGAAAATT
ATTTAATCAAAATTTCAGAAAAAATGAATGATGCTAAAGTAAGTTTATTATTGAATAATTG
TGATGCTGAATATTCAAAATATTGTGATTGTAAACATACTACTACTCTCGTTAAAAGCGTT
TTAAATGGTAACGACAATACAATTAAGGAAAAGCGTGAACATATTGATTTAGATGATTTT
TCTAAATTTGGATGTGATAAAAATTCCGTTGATACAAACACAAAGGTGTGGGAATGTAAA
AAACCTTATAAATTATCCACTAAAGATGTATGTGTACCTCCGAGGAGGCAAGAATTATGT
CTTGGAAACATTGATAGAATATACGATAAAAACCTATTAATGATAAAAGAGCATATTCTT
GCTATTGCAATATATGAATCAAGAATATTGAAACGAAAATATAAGAATAAAGATGATAA
AGAAGTTTGTAAAATCATAAATAAAACTTTCGCTGATATAAGAGATATTATAGGAGGTAC
TGATTATTGGAATGATTTGAGCAATAGAAAATTAGTAGGAAAAATTAACACAAATTCAAA
TTATGTTCACAGGAATAAACAAAATGATAAGCTTTTTCGTGATGAGTGGTGGAAAGTTAT
TAAAAAAGATGTATGGAATGTGATATCATGGGTATTCAAGGATAAAACTGTTTGTAAAGA
AGATGATATTGAAAATATACCACAATTCTTCAGATGGTTTAGTGAATGGGGTGATGATTA
TTGCCAGGATAAAACAAAAATG
>Nigeria NIG103_EBA175AJ438803.1
TATGTATGTATTCCTGATCGTAGAATCCAATTATGCATTGTTAATCTTAGCATTATTAAAA
CATATACAAAAGAGACCATGAAGGATCATTTCATTGAAGCCTCTAAAAAAGAATCTCAA
CTTTTGCTTAAAAAAAAATGATAACAAATATAATTCTAAATTTTGTAATGATTTGAAGAATA
GTTTTTTAGATTATGGACATCTTGCTATGGGAAATGATATGGATTTTGGAGGTTATTCAAC
TAAGGCAGAAAACAAAATTCAAGAAGTTTTTAAAGGGGCTCATGGGGAAATAAGTGAAC
ATGAAATTAAAAATTTTAGAAAAAAAATGGTGGAATGAATTTAGAGAGAAACTTTGGGAA
GCTATGTTATCTGAGCATAAAAATAATATAAATAATTGTAAAAATATTCCCCAAGAAGAA
TTACAAATTACTCAATGGATAAAAGAATGGCATGGAGAATTTTTGCTTGAAAGAGATAAT
AGATCAAAATTGCCAAAAAGTAAATGTAAAAATAATACATTATATGAAGCATGTGAGAA
GGAATGTATTGATCCATGTATGAAATATAGAGATTGGATTATTAGAAGTAAATTTGAATG
GCATACGTTATCGAAAGAATATGAAACTCAAAAAGTTTCAAAGGAAAATGCGGAAAATT
ATTTAATCAAAATTTCAGAAAACAAGAATGATGCTAAAGTAAGTTTATTATTGAATAATT
GTGATGCTGAATATTCAAAATATTGTGATTGTAAACATACTACTACTCTCGTTAAAAGCGT
TTTAAATGGTAACGACAATACAATTAAGGAAAAGCGTGAACATATTGATTTAGATGATTT
TTCTAAATTTGGATGTGATAAAAATTCCGTTGATACAAACACAAAGGTGTGGGAATGTAA
AAAACCTTATATATTATCCACTAAAGATGTATGTGTACCTCCGAGGAGGCAAGAATTATG
TCTTGGAAACATTGATAGAATATACGATAAAAACCTATTAATGATAAAAGAGCATATTCT
TGCTATTGCAATATATGAATCAAGAATATTGAAACGAAAATATAAGAATAAAGATGATAA
AGAAGTTTGTAAAATCATAAATAAAACTTTCGCTGATATAAGAGATATTATAGGAGGTAC
TGATTATTGGAATGATTTGAGCAATAGAAAATTAGTAGGAAAAATTAACACAAATTCAAA
ATATGTTCACAGGAATAAAAAAAATGATAAGCTTTTTCGTGATGAGTGGTGGAAAGTTAT
TAAAAAAGATGTATGGAATGTGATATCATGGGTATTCAAGGATAAAACTGTTTGTAAAGA
AGATGATATTGAAAATATACCACAATTCTTCAGATGGTTTAGTGAATGGGGTGATGATTA
TTGCCAGGATAAAACAAAAATG

>Nigeria NIG66_EBA175AJ438802.1
TATGTATGTATTCCTGATCGTAGAATCCAATTATGCATTGTTAATCTTAGCATTATTAAAA
CATATACAAAAGAGACCATGAAGGATCATTTCATTGAAGCCTCTAAAAAAGAATCTCAA
CTTTTGCTTAAAAAAAAATGATAACAAATATAATTCTAAATTTTGTAATGATTTGAAGAATA
GTTTTTTAGATTATGGACATCTTGCTATGGGAAATGATATGGATTTTGGAGGTTATTCAAC
TAAGGCAGAAAACAAAATTCAAGAAGTTTTTAAAGGGGCTCATGGGAAAATAAGTGAAC
ATGAAATTAAAAATTTTAGAAAAAAATGGTGGAATGAATTTAGAGAGAAACTTTGGGAA
GCTATGTTATCTGAGCATAAAAATAATATAAATAATTGTAAAAATATTCCCCAAGAAGAA
TTACAAATTACTCAATGGATAAAAGAATGGCATGGAGAATTTTTGCTTGAAAGAGATAAT
AGATCAAAATTGCCAAAAAGTAAATGTAAAAATAATACATTATATGAAGCATGTGAGAA
GGAATGTATTGATCCATGTATGAAATATAGAGATTGGATTATTAGAAGTAAATTTGAATG
GCATACGTTATCGAAAGAATATGAAACTCAAAAAGTTTCAAAGGAAAATGCGGAAAATT
ATTTAATCAAAATTTCAGAAAACAAGAATGATGCTAAAGTAAGTTTATTATTGAATAATT
GTGATGCTGAATATTCAAAATATTGTGATTGTAAACATACTACTACTCTCGTTAAAAGCGT
TTTAAATGGTAACGACAATACAATTAAGGAAAAGCGTGAACATATTGATTTAGATGATTT
TTCTAAATTTGGATGTGATAAAAATTCCGTTGATACAAACACAAAGGTGTGGGAATGTAA
AAAACCTTATAAATTATCCACTAAAGATGTATGTGTACCTCCGAGGAGGCAAGAATTATG
TCTTGGAAACATTGATAGAATATACGATAAAAACCTATTAATGATAAAAGAGCATATTCT
TGCTATTGCAATATATGAATCAAGAATATTGAAACGAAAATATAAGAATAAAGATGATAA
AGAAGTTTGTAAAATCATAAATAAAACTTTCGCTGATATAAGAGATATTATAGGAGGTAC
TGATTATTGGAATGATTTGAGCAATAGAAAATTAGTAGGAAAAATTAACACAAATTCAAA
ATATGTTCACAGGAATAAAAAAAATGATAAGCTTTTTCGTGATGAGTGGTGGAAAGTTAT
TAAAAAAGATGTATGGAATGTGATATCATGGGTATTCAAGGATAAAACTGTTTGTAAAGA
AGATGATATTGAAAATATACCACAATTCTTCAGATGGTTTAGTGAATGGGGTGATGATTA
TTGCCAGGATAAAACAAAAATG
>Nigeria NIG52_EBA175AJ438801.1
TATGTATGTATTCCTGATCGTAGAATCCAATTATGCATTGTTAATCTTAGCATTATTAAAA
CATATACAAAAGAGACCATGAAGGATCATTTCATTGAAGCCTCTAAAAAAGAATCTCAA
CTTTTGCTTAAAAAAAAATGATAACAAATATAATTCTAAATTTTGTAATGATTTGAAGAATA
GTTTTTTAGATTATGGACATCTTGCTATGGGAAATGATATGGATTTTGGAGGTTATTCAAC
TAAGGCAGAAAACAAAATTCAAGAAGTTTTTAAAGGGGCTCATGGGGAAATAAGTGAAC
ATGAAATTAAAAATTTTAGAAAAAAATGGTGGAATGAATTTAGAGAGAAACTTTGGGAA
GCTATGTTATCTGAGCATAAAAATAATATAAATAATTGTAAAAATATTCCCCAAGAAGAA
TTACAAATTACTCAATGGATAAAAGAATGGCATGGAGAATTTTTGCTTGAAAGAGATAAT
AGATCAAAATTGCCAAAAAGTAAATGTAAAAATAATACATTATATGAAGCATGTGAGAA
GGAATGTATTGATCCATGTATGAAATATAGAGATTGGATTATTAGAAGTAAATTTGAATG
GCATACGTTATCGAAAGAATATGAAACTCAAAAAGTTTCAAAGGAAAATGCGGAAAATT
ATTTAATCAAAATTTCAGAAAACAAGAATGATGCTAAAGTAAGTTTATTATTGAATAATT
GTGATGCTGAATATTCAAAATATTGTGATTGTAAACATACTACTACTCTCGTTAAAAGCGT
TTTAAATGGTAACGACAATACAATTAAGGAAAAGCGTGAACATATTGATTTAGATGATTT
TTCTAAATTTGGATGTGATAAAAATTCCGTTGATACAAACACAAAGGTGTGGGAATGTAA
AAAACCTTATATATTATCCACTAAAGATGTATGTGTACCTCCGAGGAGGCAAGAATTATG
TCTTGGAAACATTGATAGAATATACGATAAAAACCTATTAATGATAAAAGAGCATATTCT
TGCTATTGCAATATATGAATCAAGAATATTGAAACGAAAATATAAGAATAAAGATGATAA

AGAAGTTTGTAAAATCATAAATAAAACTTTCGCTGATATAAGAGATATTATAGGAGGTAC
TGATTATTGGAATGATTTGAGCAATAGAAAATTAGTAGGAAAAATTAACACAAATTCAAA
ATATGTTCACAGGAATAAAAAAAATGATAAGCTTTTTCGTGATGAGTGGTGGAAAGTTAT
TAAAAAAGATGTATGGAATGTGATATCATGGGTATTCAAGGATAAAACTGTTTGTAAAGA
AGATGATATTGAAAATATACCACAATTCTTCAGATGGTTTAGTGAATGGGGTGATGATTA
TTGCCAGGATAAAACAAAAATG
>Nigeria NIG39_EBA175AJ438800.1
TATGTATGTATTCCTGATCGTAGAATCCAATTATGCATTGTTAATCTTAGCATTATTAAAA
CATATACAAAAGAGACCATGAAGGATCATTTCATTGAAGCCTCTAAAAAAGAATCTCAA
CTTTTGCTTAAAAAAAAATGATAACGAATATAATTCTAAATTTTGTAATGATTTGAAGAATA
GTTTTTTAGATTATGGACATCTTGCTATGGGAAATGATATGGATTTTGGAGGTTATTCAAC
TAAGGCAGAAAACAAAATTCAAGAAGTTTTTAAAGGGGCTCATGGGGAAATAAGTGAAC
ATAAAATTAAAAATTTTAGAAAAGAATGGTGGAATGAATTTAGAGAGAAACTTTGGGAA
GCTATGTTATCTGAGCATAAAAATAATATAAATAATTGTAAAAATATTCCCCAAGAAGAA
TTACAAATTACTCAATGGATAAAAGAATGGCATGGAGAATTTTTGCTTGAAAGAGATAAT
AGATCAAAATTGCCAAAAAGTAAATGTAAAAATAATACATTATATGAAGCATGTGAGAA
GGAATGTATTGATCCATGTATGAAATATAGAGATTGGATTATTAGAAGTAAATTTGAATG
GCATACGTTATCGAAAGAATATGAAACTCAAAATGTTTCAAAGGAAAATGCGGAAAATT
ATTTAATCAAAATTTCAGAAAAAATGAATGATGCTAAAGTAAGTTTATTATTGAATAATTG
TGATGCTGAATATTCAAAATATTGTGATTGTAAACATACTACTACTCTCGTTAAAAGCGTT
TTAAATGGTAACGACAATACAATTAAGGAAAAGCGTGAACATATTGATTTAGATGATTTT
TCTAAATTTGGATGTGATAAAAATTCCGTTGATACAAACACAAAGGTGTGGGAATGTAAA
AAACCTTATATATTATCCACTAAAGATGTATGTGTACCTCCGAGGAGGCAAGAATTATGT
CTTGGAAACATTGATAGAATATACGATAAAAACCTATTAATGATAAAAGAGCATATTCTT
GCTATTGCAATATATGAATCAAGAATATTGAAACGAAAATATAAGAATAAAGATGATAA
AGAAGTTTGTAAAATCATAAATAAAACTTTCGCTGATATAAGAGATATTATAGGAGGTAC
TGATTATTGGAATGATTTGAGCAATAGAAAATTAGTAGGAAAAATTAACACAAATTCAAA
ATATGTTCACAGGAATAAAAAAAATGATAAGCTTTTTCGTGATGAGTGGTGGAAAGTTAT
TAAAAAAGATGTATGGAATGTGATATCATGGGTATTCAAGGATAAAACTGTTTGTAAAGA
AGATGATATTGAAAATATACCACAATTCTTCAGATGGTTTAGTGAATGGGGTGATGATTA
TTGCCAGGATAAAACAAAAATG
