# Supplementary material for: Population genetic analysis of the Plasmodium falciparum erythrocyte binding antigen-175 (EBA-175) gene in Equatorial Guinea
Source: Malar J. 2021 Sep 19;20:374. doi: 10.1186/s12936-021-03904-x (PMC8451130; doi:10.1186/s12936-021-03904-x)
Supplement: Supplementary file 4 — Additional file 4. Global PfEBA-175 region II acquired from NCBI and sequencing in the study. [file 12936_2021_3904_MOESM4_ESM.pptx]

## Slide 1
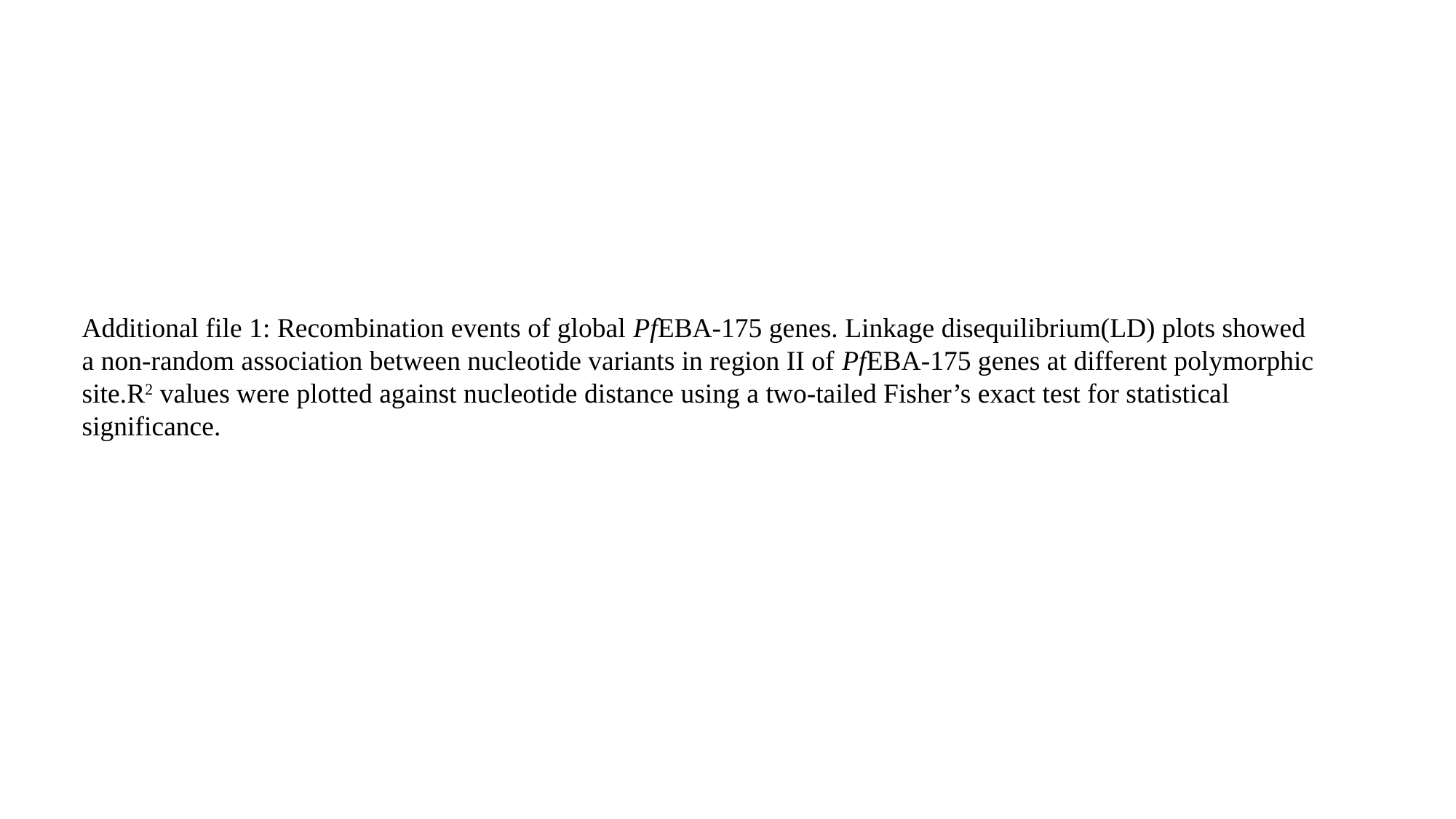

Additional file 1: Recombination events of global PfEBA-175 genes. Linkage disequilibrium(LD) plots showed a non-random association between nucleotide variants in region II of PfEBA-175 genes at different polymorphic site.R2 values were plotted against nucleotide distance using a two-tailed Fisher’s exact test for statistical significance.

## Slide 2
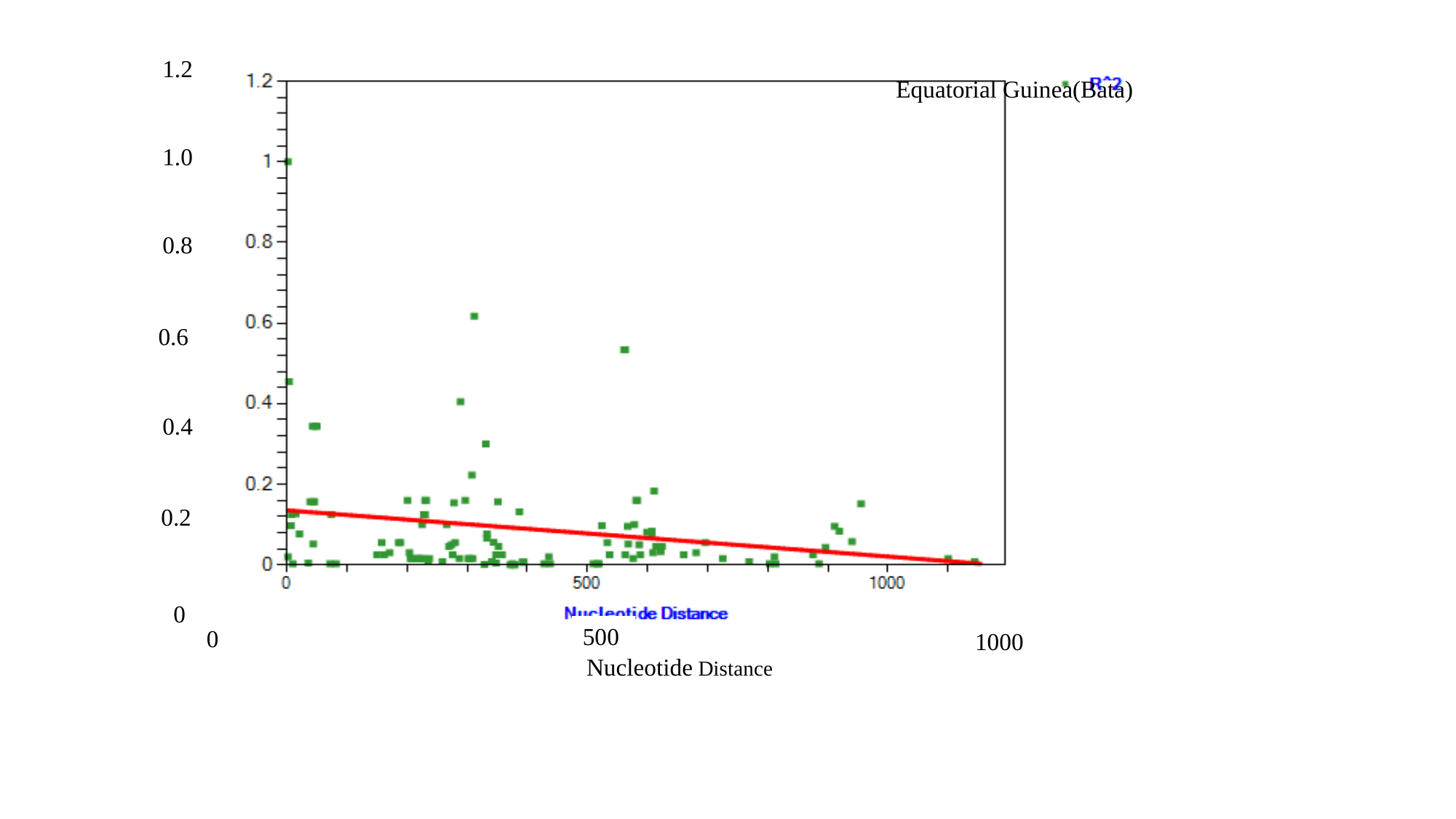

1.2
Equatorial Guinea(Bata)
1.0
0.8
0.6
0.4
0.2
0
500
0
1000
Nucleotide Distance

## Slide 3
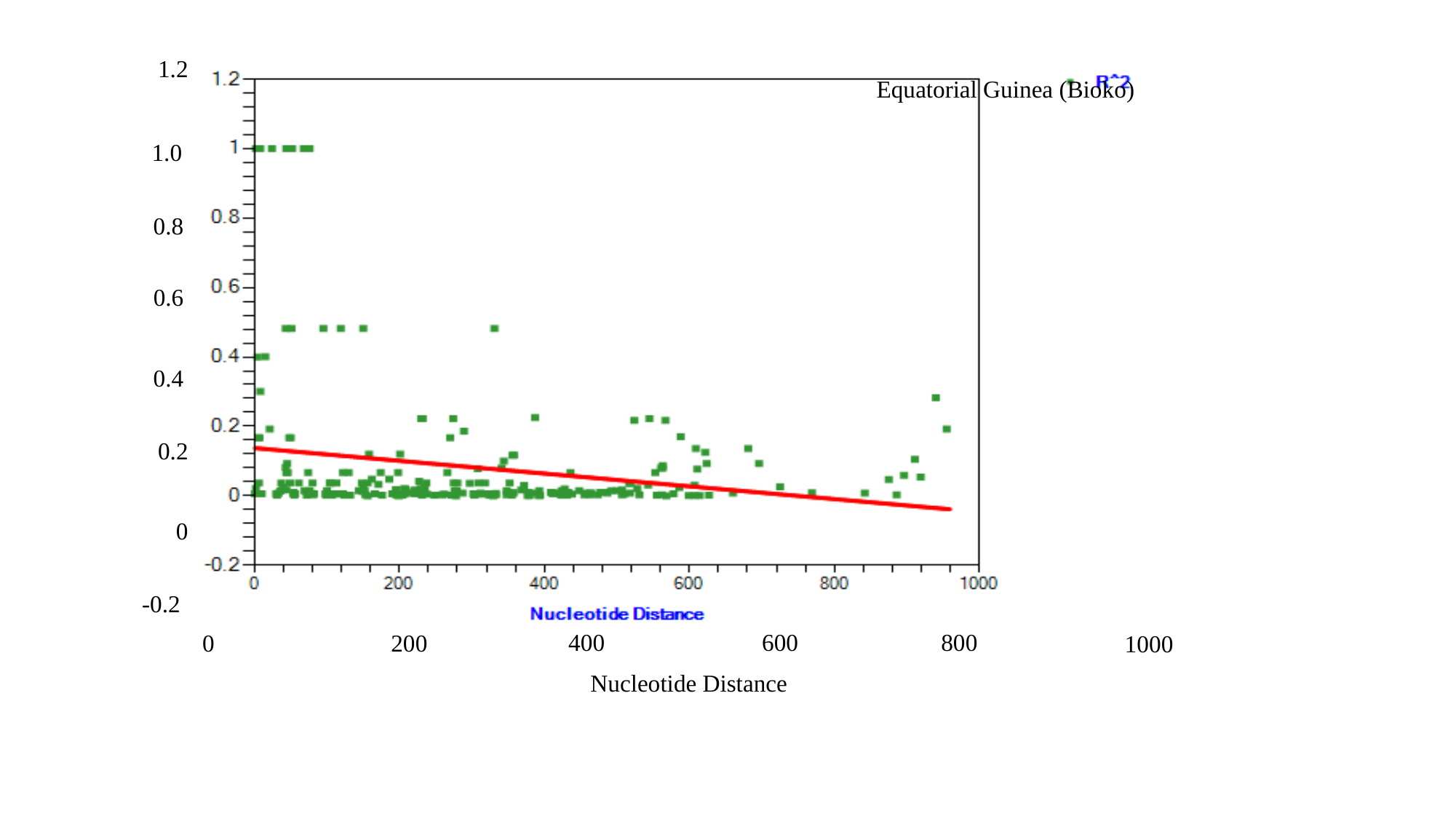

1.2
Equatorial Guinea (Bioko)
1.0
0.8
0.6
0.4
0.2
0
-0.2
400
600
800
0
200
1000
Nucleotide Distance

## Slide 4
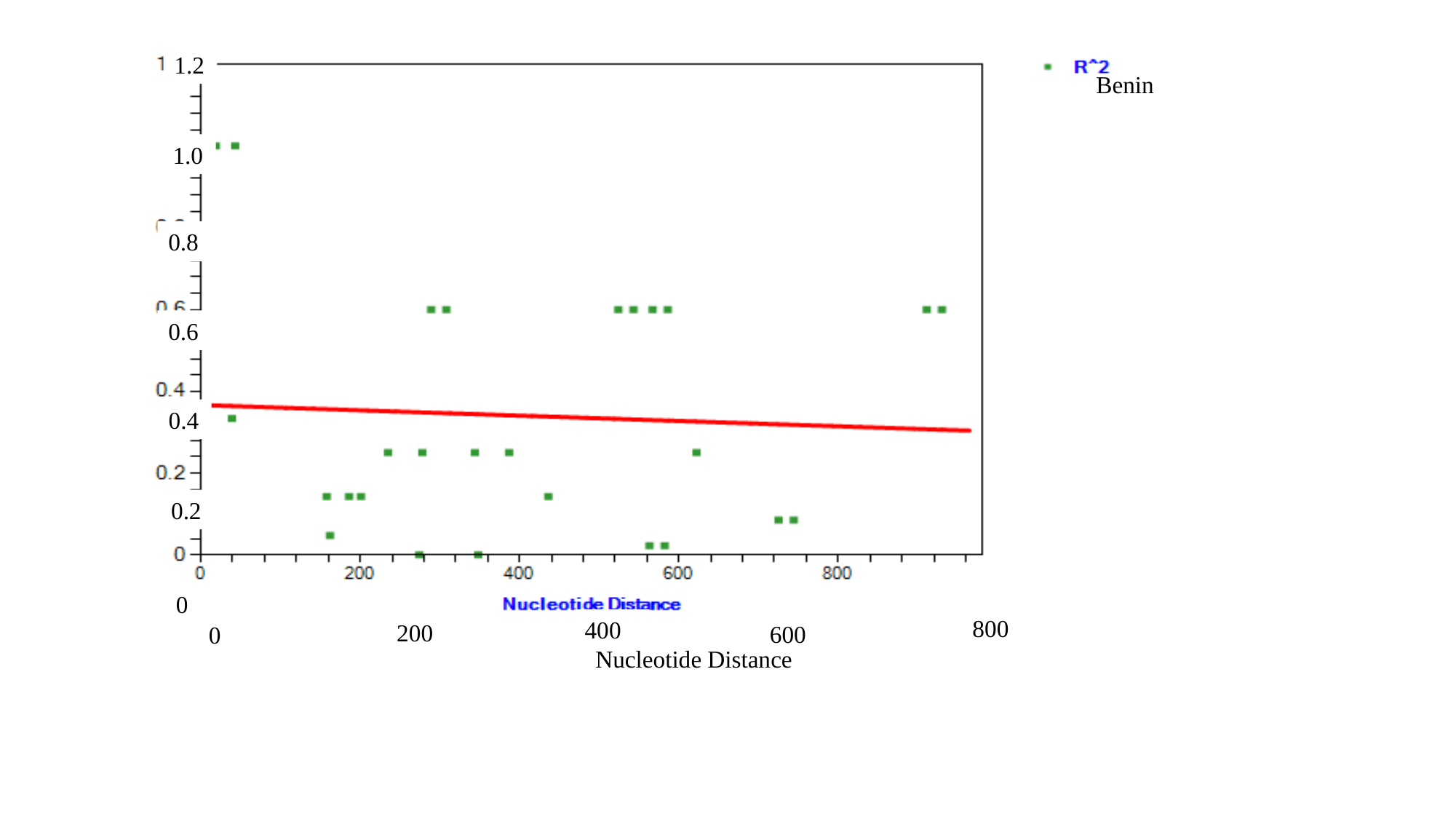

1.2
Benin
1.0
0.8
0.6
0.4
0.2
0
800
400
200
600
0
Nucleotide Distance

## Slide 5
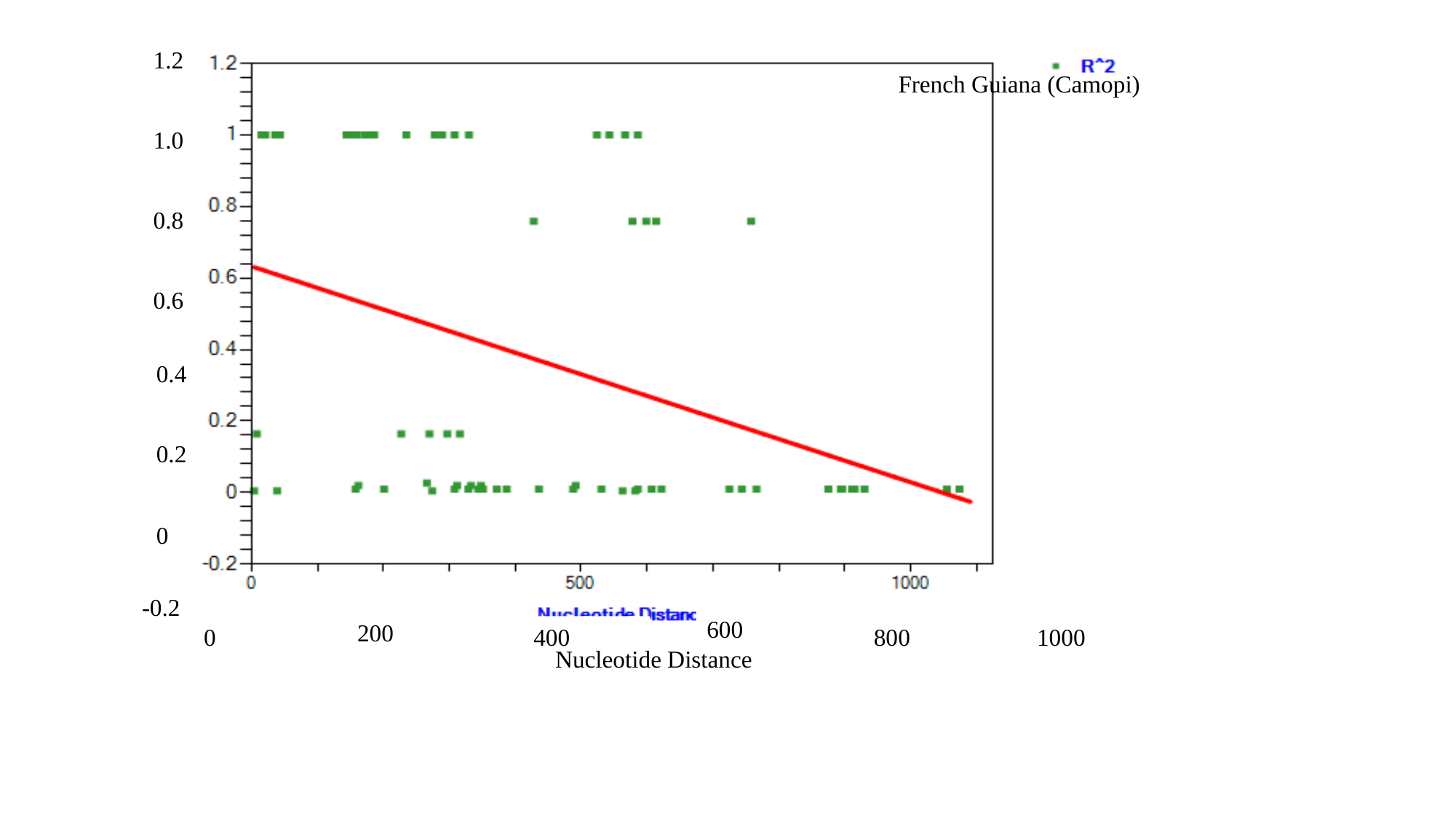

1.2
French Guiana (Camopi)
1.0
0.8
0.6
0.4
0.2
0
-0.2
600
200
0
400
800
1000
Nucleotide Distance

## Slide 6
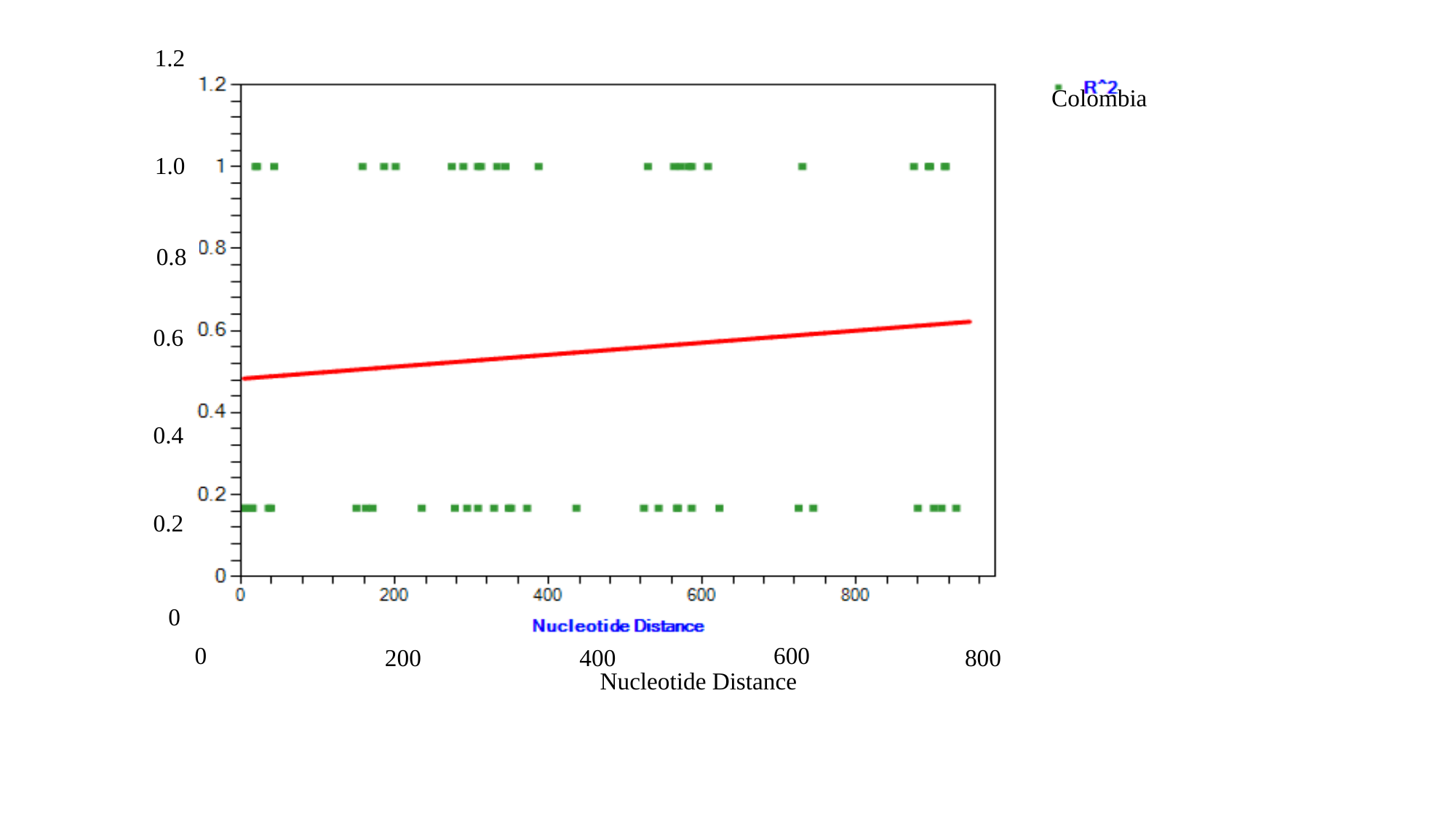

1.2
 Colombia
1.0
0.8
0.6
0.4
0.2
0
0
600
200
400
800
Nucleotide Distance

## Slide 7
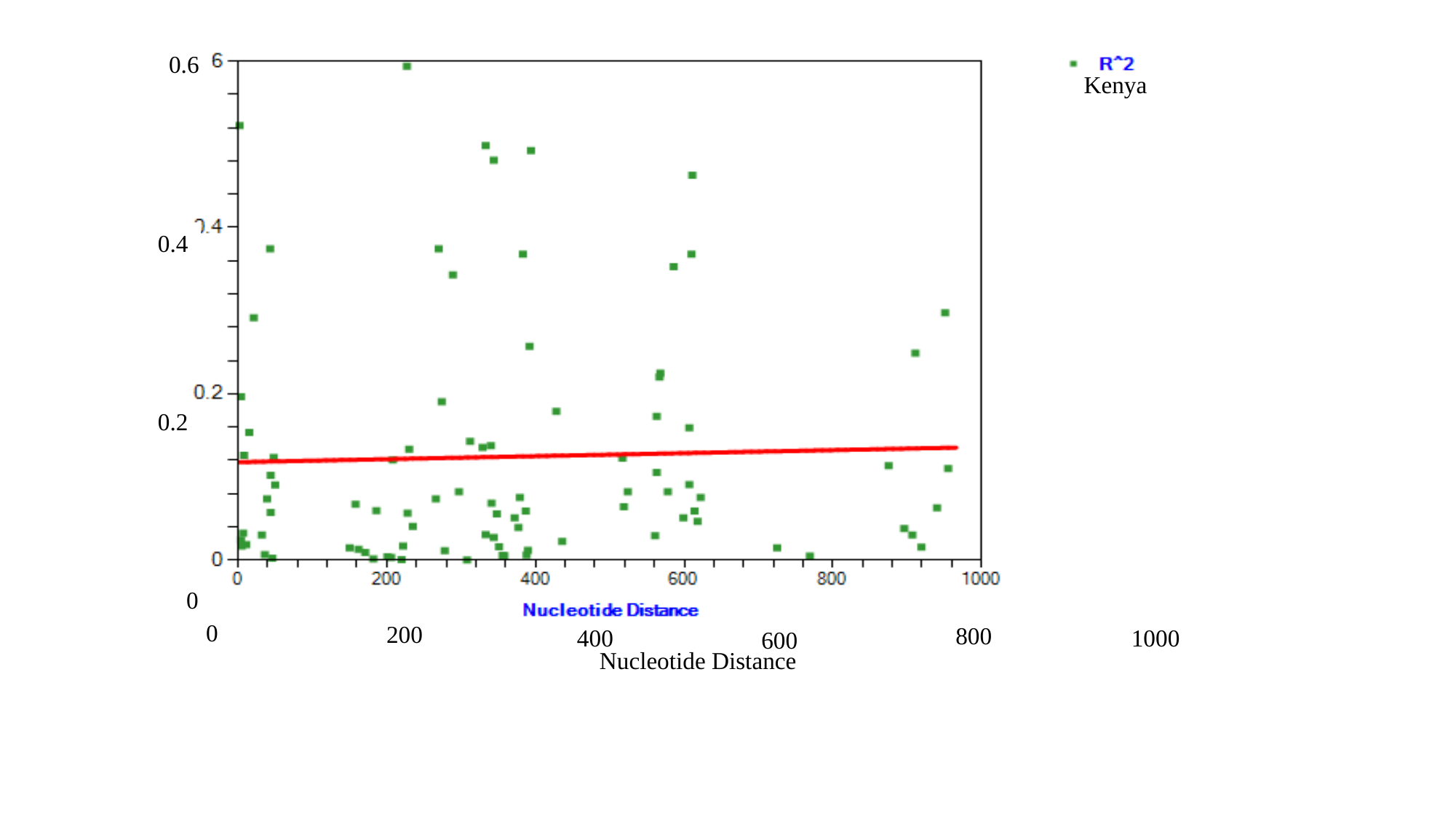

0.6
Kenya
0.4
0.2
0
0
200
800
400
1000
600
Nucleotide Distance

## Slide 8
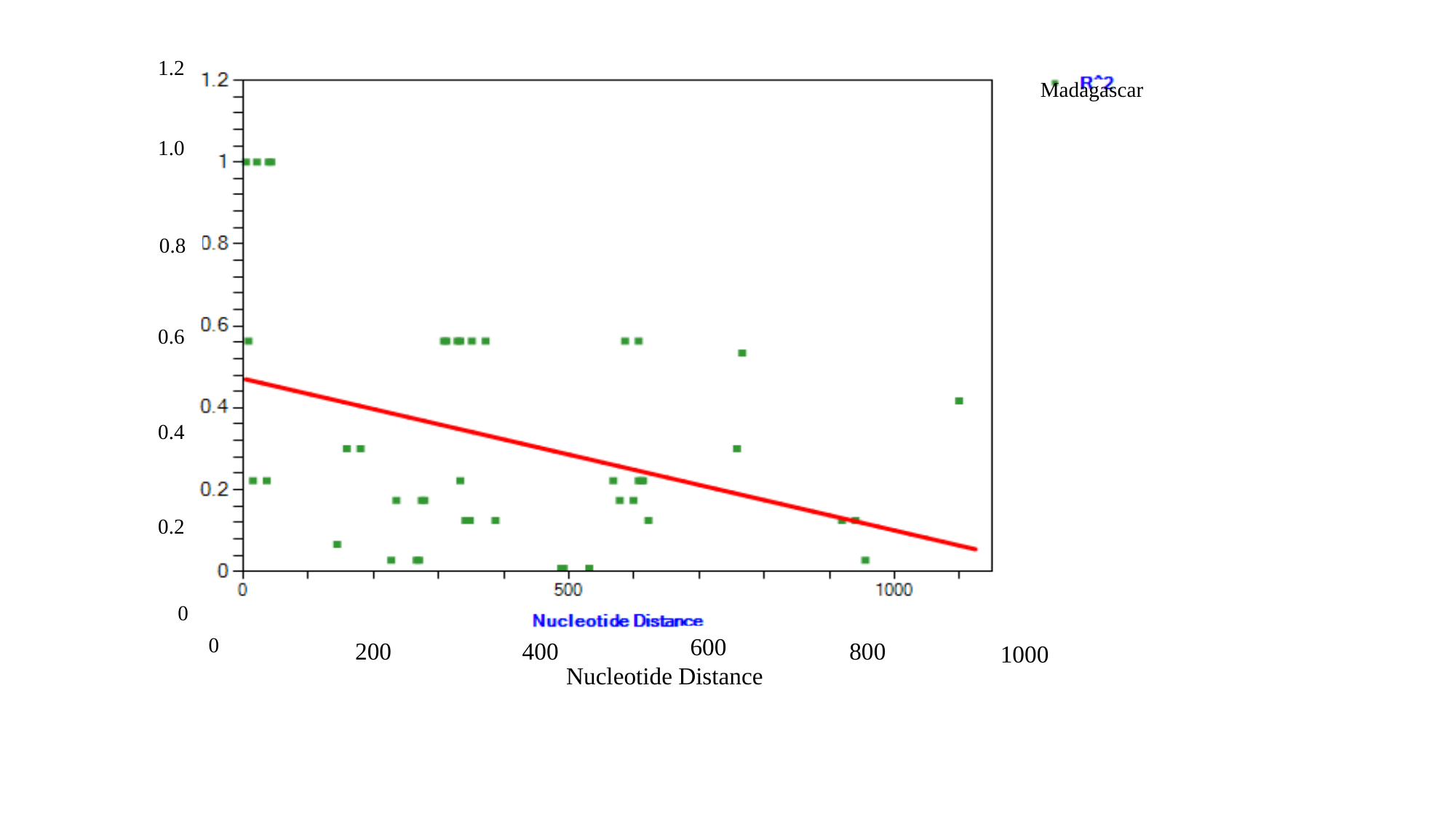

1.2
Madagascar
1.0
0.8
0.6
0.4
0.2
0
0
600
200
400
800
1000
Nucleotide Distance

## Slide 9
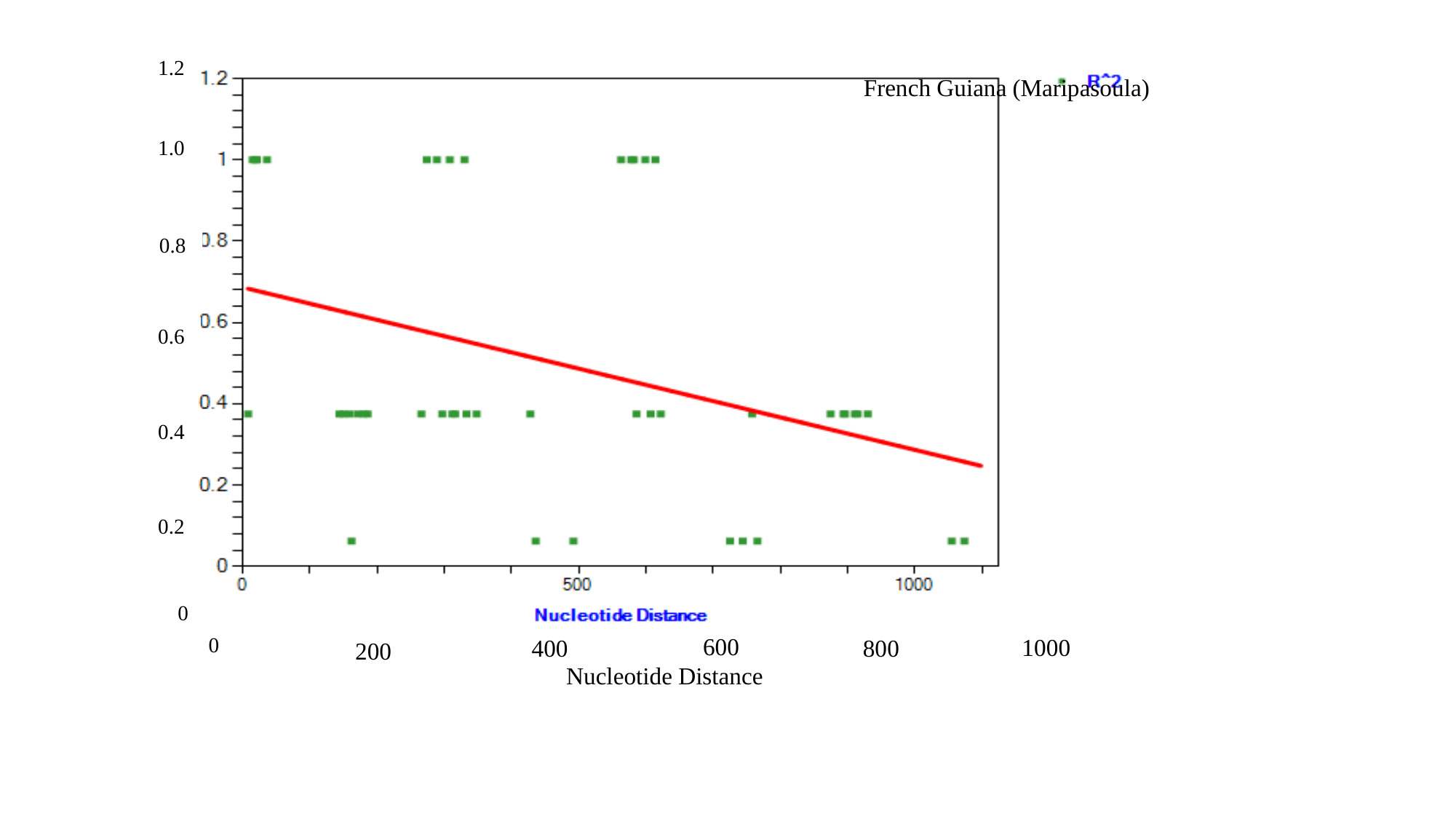

1.2
French Guiana (Maripasoula)
1.0
0.8
0.6
0.4
0.2
0
0
600
1000
400
800
200
Nucleotide Distance

## Slide 10
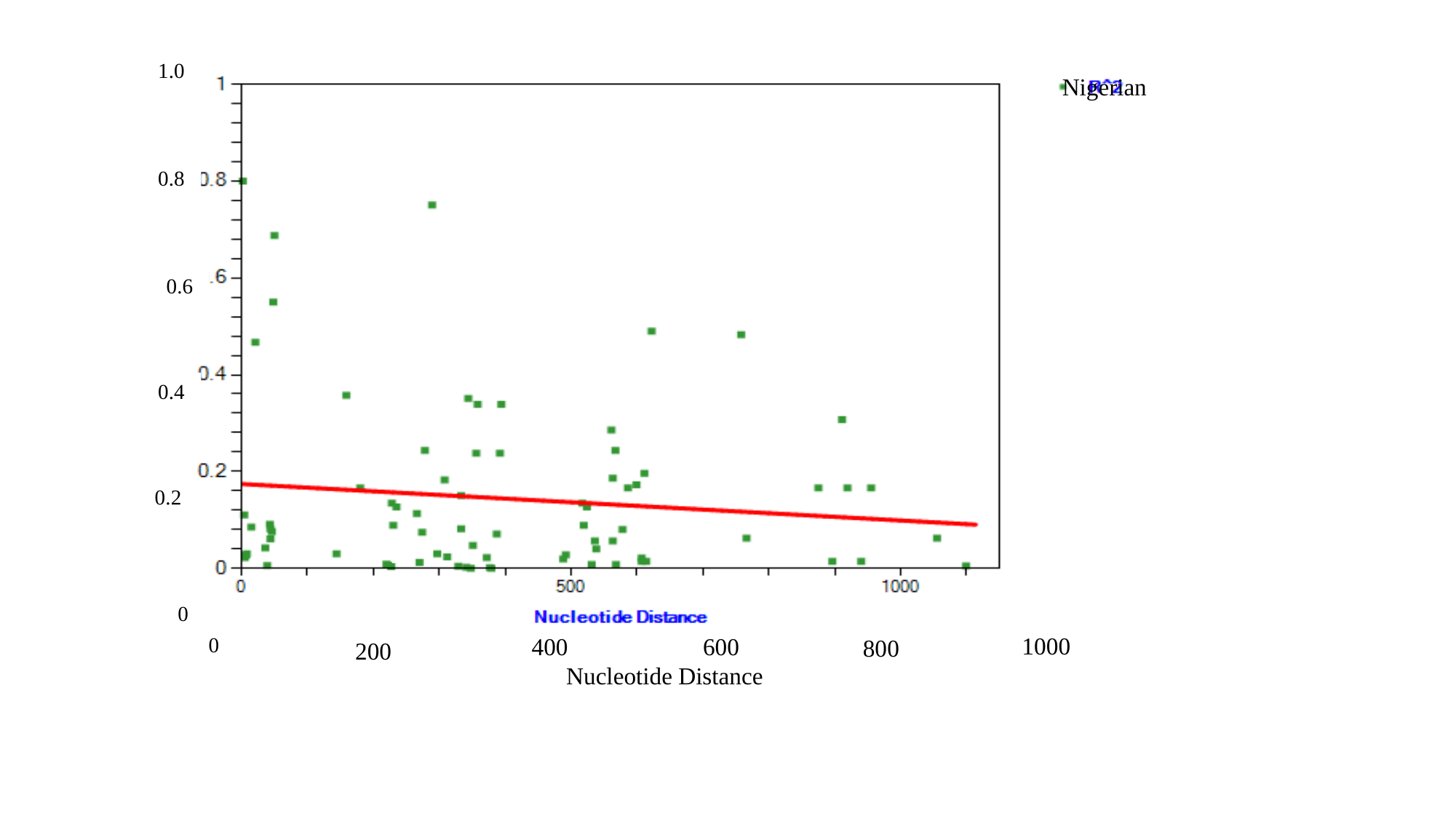

1.0
Nigerian
0.8
0.6
0.4
0.2
0
1000
0
600
400
800
200
Nucleotide Distance

## Slide 11
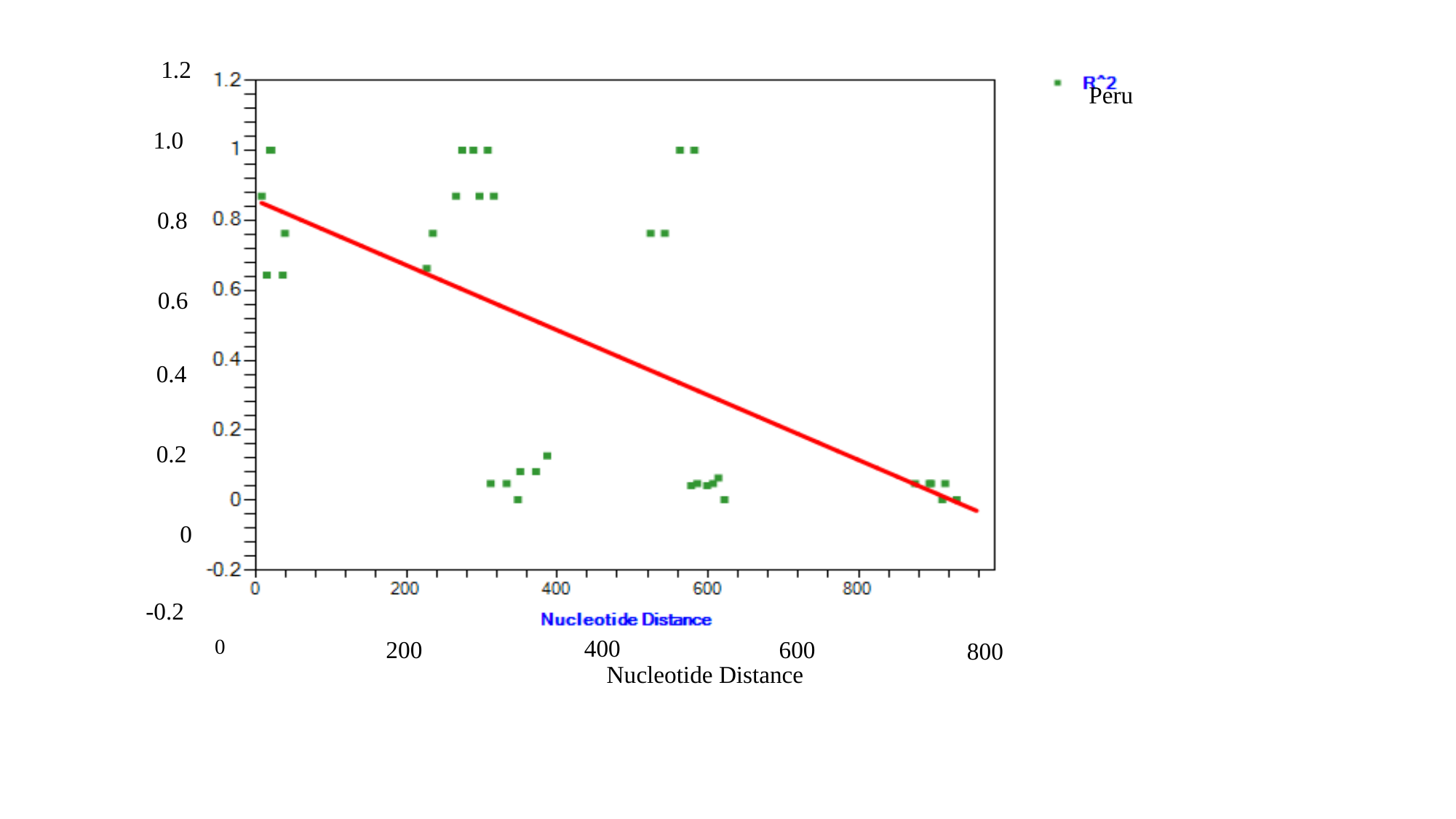

1.2
Peru
1.0
0.8
0.6
0.4
0.2
0
-0.2
0
400
200
600
800
Nucleotide Distance

## Slide 12
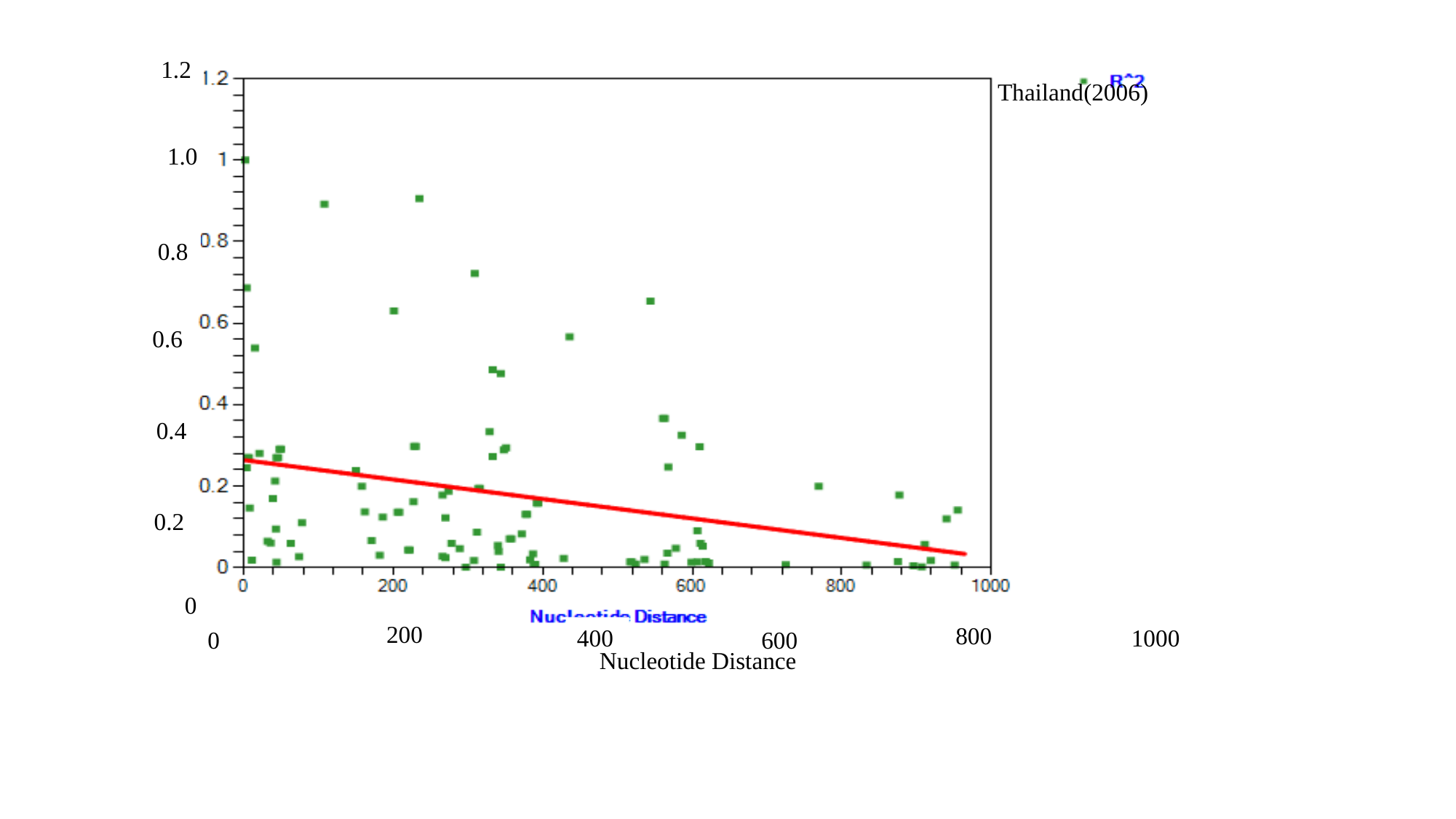

1.2
Thailand(2006)
1.0
0.8
0.6
0.4
0.2
0
200
800
400
1000
0
600
Nucleotide Distance

## Slide 13
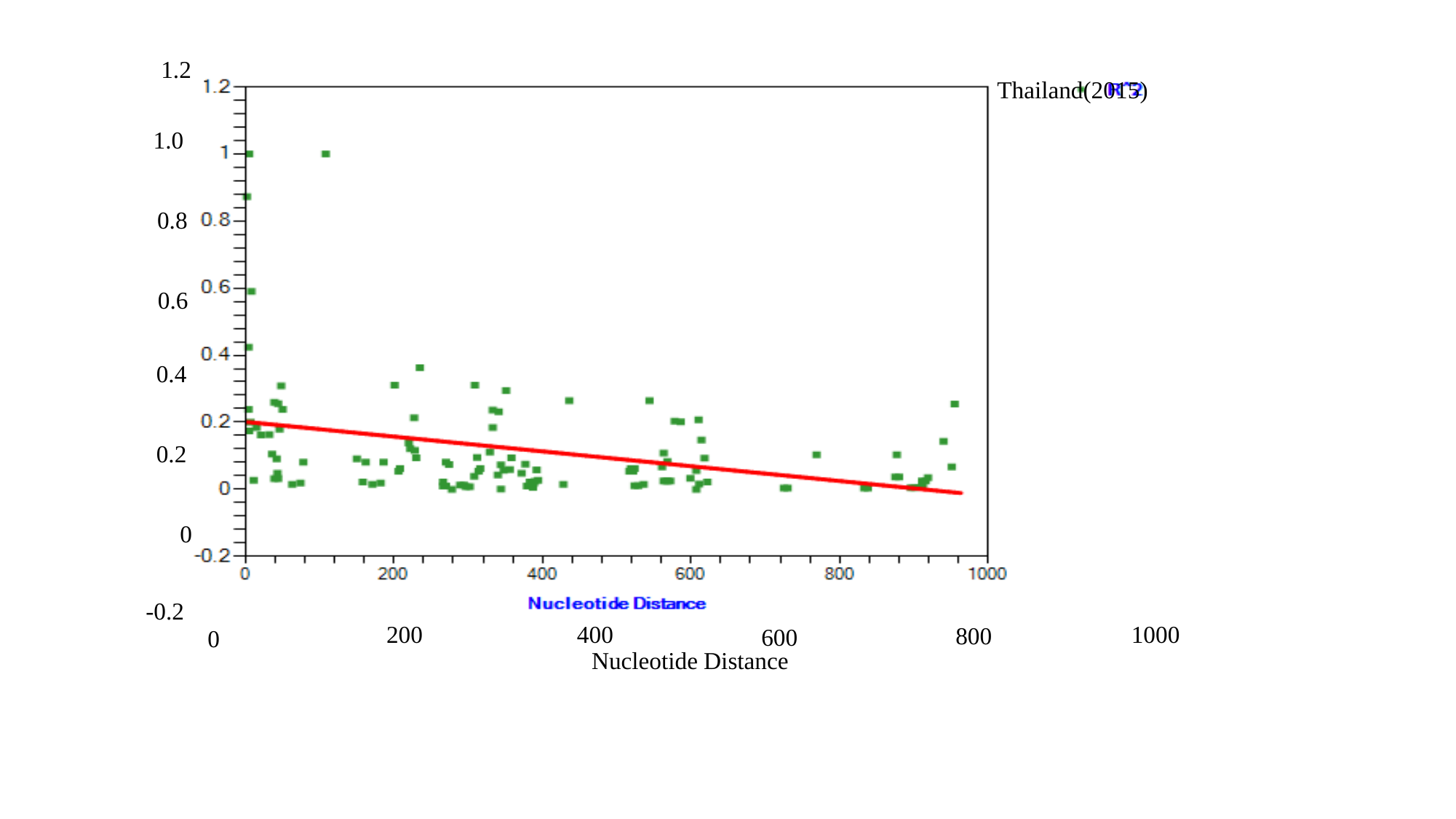

1.2
Thailand(2015)
1.0
0.8
0.6
0.4
0.2
0
-0.2
200
400
1000
800
600
0
Nucleotide Distance

## Slide 14
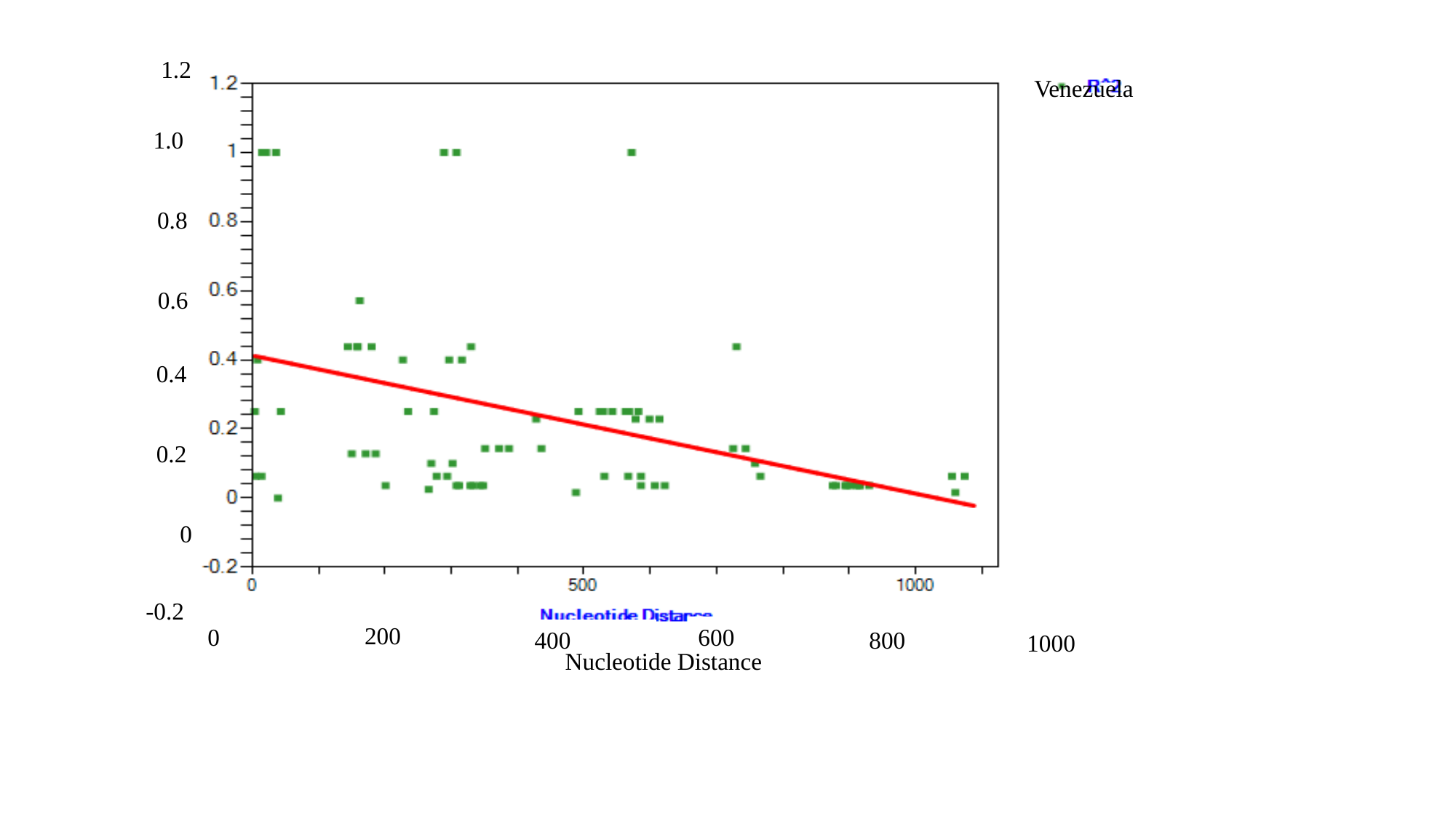

1.2
Venezuela
1.0
0.8
0.6
0.4
0.2
0
-0.2
200
0
600
400
800
1000
Nucleotide Distance
