# Supplementary material for: Population genetic analysis of the Plasmodium falciparum erythrocyte binding antigen-175 (EBA-175) gene in Equatorial Guinea
Source: Malar J. 2021 Sep 19;20:374. doi: 10.1186/s12936-021-03904-x (PMC8451130; doi:10.1186/s12936-021-03904-x)

## Slide 1
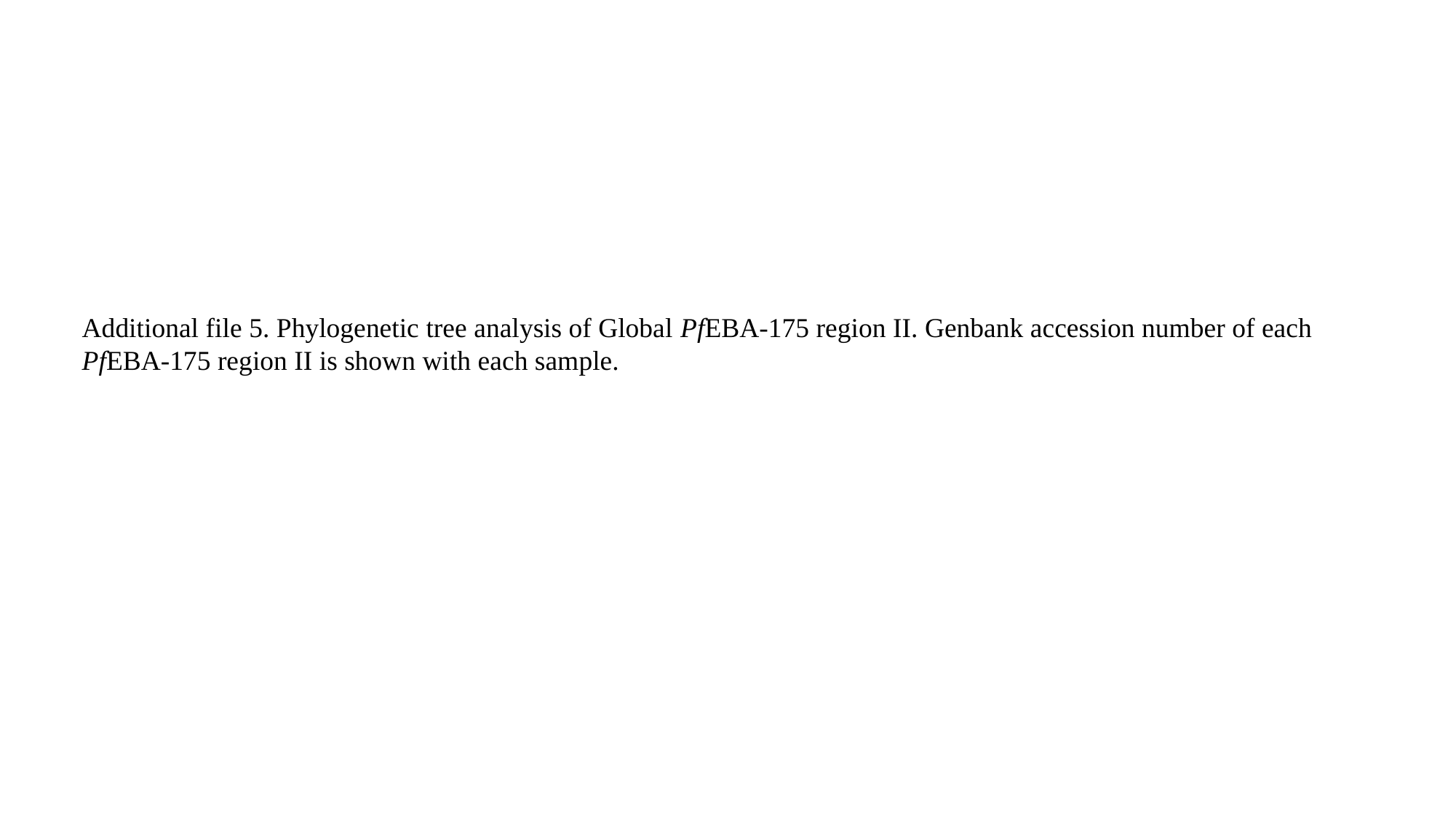

Additional file 5. Phylogenetic tree analysis of Global PfEBA-175 region II. Genbank accession number of each PfEBA-175 region II is shown with each sample.

## Slide 2
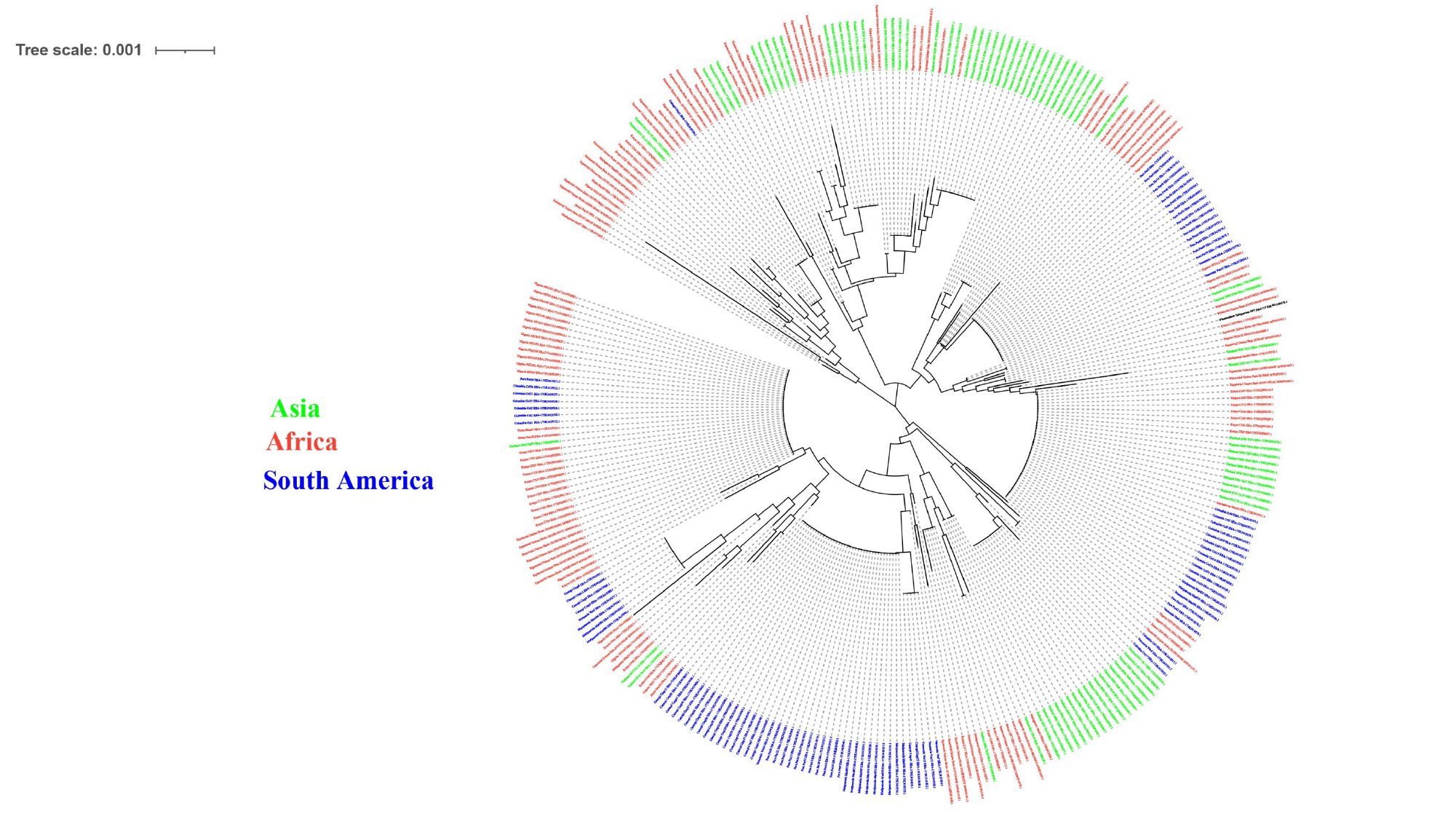

Supplement: Supplementary file 5 — Additional file 5. Phylogenetic tree analysis of Global PfEBA-175 region II. [file 12936_2021_3904_MOESM5_ESM.pptx]
